# Phylogenetic analyses suggest centipede venom arsenals were repeatedly stocked by horizontal gene transfer

**DOI:** 10.1038/s41467-021-21093-8

**Published:** 2021-02-05

**Authors:** Eivind A. B. Undheim, Ronald A. Jenner

**Affiliations:** 1 Centre for Biodiversity Dynamics, Department of Biology, NTNU, Trondheim, Norway; 2 Centre for Ecological and Evolutionary Synthesis, Department of Bioscience, University of Oslo, Blindern, Oslo Norway; 3 Centre for Advanced Imaging, University of Queensland, St Lucia, QLD Australia; 4 Department of Life Sciences, Natural History Museum, London, UK

**Keywords:** Molecular evolution, Phylogenetics

## Abstract

Venoms have evolved over a hundred times in animals. Venom toxins are thought to evolve mostly by recruitment of endogenous proteins with physiological functions. Here we report phylogenetic analyses of venom proteome-annotated venom gland transcriptome data, assisted by genomic analyses, to show that centipede venoms have recruited at least five gene families from bacterial and fungal donors, involving at least eight horizontal gene transfer events. These results establish centipedes as currently the only known animals with venoms used in predation and defence that contain multiple gene families derived from horizontal gene transfer. The results also provide the first evidence for the implication of horizontal gene transfer in the evolutionary origin of venom in an animal lineage. Three of the bacterial gene families encode virulence factors, suggesting that horizontal gene transfer can provide a fast track channel for the evolution of novelty by the exaptation of bacterial weapons into animal venoms.

## Abstract (web summary)

Animal venoms have evolved many times primarily by recruitment of endogenous proteins with physiological functions. Undheim and Jenner find that centipede venoms have recruited at least five gene families from bacterial and fungal donors, involving at least eight horizontal gene transfer events.

## Introduction

Horizontal gene transfer (HGT) between kingdoms and domains of life has contributed to the evolution of a diversity of novel adaptive traits in animals, including the ability of bdelloid rotifers to withstand desiccation, the ability of springtails to feed on decaying organic matter, and the ability of plant-parasitic nematodes to degrade plant cell walls^1–7^. HGT has also contributed to the evolution of venom, one of the most convergently evolved animal adaptations. Venoms are complex, typically proteinaceous, secretions that are used primarily for predation and defence by a wide phylogenetic range of animals. However, although animal venoms have evolved at least a hundred times independently^8^, the contribution of HGT to the evolution of venom arsenals has so far been shown to be minor.

HGT is a well-supported hypothesis for only three gene families present in arthropod and cnidarian venoms. Phylogenetic analyses, in some cases supported by genomic information, strongly suggest that bacteria were the source of type D phospholipases found in the venoms of sicariid spiders, scorpions, and ticks^9^, and of pore-forming toxins expressed in the venom glands of ticks as well as gland cells in the digestive system of cnidarians, although it is debated whether these should be considered part of the venom system or not^10^. Similarly, glycoside family 19 chitinases found in the venom of chalcidoid parasitoid wasps were probably transferred from parasitic fungi^11^. Other potential cases of HGT contributing to insect venoms currently lack phylogenetic support^12–14^, while the direction of HGT of neurotoxic α-latrotoxins present in the venom of theridiid spiders and bacteria remains uncertain^15^. Although HGT is currently not considered to be a major mechanism of venom evolution, venoms are nevertheless a promising research area given the existence of many tens of thousands of mostly unstudied venomous animal species. Many venoms also contain a substantial number of proteins with few or no known metazoan homologues^16–21^, and these may include HGT candidates.

One venomous lineage that contains a large diversity of unassignable venom proteins^22,23^ is centipedes (Chilopoda). Centipedes are one of the oldest terrestrial venomous lineages, with a fossil record going back 418 million years^24^. Living species belong to five orders: Scutigeromorpha (long-legged house centipedes), Lithobiomorpha (stone centipedes), Geophilomorpha (long-bodied earth centipedes), Scolopendromorpha (the most familiar centipedes, including large tropical species), and Craterostigmomorpha (two species from Tasmania and New Zealand). All of these have complex venoms that are used for predation and defence. While most of the protein families contained in centipede venoms were recruited from gene families that are widespread in animals, others have few or no metazoan homologues. This pattern suggests that the evolutionary origins of several centipede venom toxins could lie outside the animal kingdom.

We show that multiple HGTs have stocked centipede venom arsenals throughout their evolution. Phylogenetic analyses of venom gland transcriptome and venom proteome data assisted by genomic analyses identified seven gene families encoding centipede venom proteins and peptides that were horizontally transferred between bacteria, fungi, oomycetes, and centipedes. Our analyses reveal between 10 and 12 HGT events. At least eight HGTs involved five gene families that transferred from bacteria and fungi into centipede venoms, whereas the direction of two or three HGTs between centipedes and fungi and oomycetes remain uncertain. Three of the protein families in bacterial donor taxa are virulence factors involved in pathogenicity, suggesting that centipedes have repurposed bacterial weapons as venom components involved in predation and/or defence. Our findings suggest that HGT can be an important factor shaping the evolution of animal venoms.

## Results and discussion

### Overall support for HGT

Several methods are available for identifying HGT^25^. A combination of phylogenetic analyses of candidate HGT gene families including both potential donor and host sequences, and confirming their presence in host genomes is considered to be the most robust method. We used this approach to identify putative HGTs from non-metazoan sources into centipede venoms. Table 1 summarizes the support for all inferred HGTs that have contributed to centipede venom arsenals. The robustly supported phylogenetic nesting of clades of centipede sequences within paraphyletic backbones of non-metazoan donor sequences supports HGT for five of the seven gene families: β-pore-forming toxin (β-PFTx), centipede peptidylarginine deiminase (centiPAD), protein with a domain of unknown function (DUF3472), pesticidal crystal protein domain-containing protein-like protein (PCPDP-like), and uncharacterized protein family 5 (unchar05). The phylogenetic nesting of centipede geotoxin 2 (GEOTX02) within fungal sequences is less well supported, while the centipede sequences for uncharacterized protein family 16 (unchar16) group in a clade that is sister to a clade of oomycete sequences. Furthermore, by confirming that five of the genes map to protein-coding genes with introns in the genome of the geophilomorph centipede *Strigamia maritima*, which is the only published centipede genome^26^, we show that they are bona fide centipede genes rather than the result of contamination or symbionts. Importantly, a recent study^27^ that examined the presence of contamination in the genome of *S. maritima* confirms that none of our HGT candidates map to the only genomic scaffold for which there are signs of contamination (scaffold JH431684; C. M. Francois, pers. comm.).

**Table 1:** Summary of gene families horizontally transferred into centipede venoms.

Gene	HGT source	Number of HGT events^a^	Phylogenetic location of HGT	Phylogenetic location of recruitment into venom	Mapped to *Strigamia maritima* genome^b^
β-PFTx	Bacteria	2 (1)	Arthropoda or Chilopoda; within Lithobiomorpha	Chilopoda	SMAR004242, SMAR004243, SMAR012417
centiPAD	Bacteria	2	Within Scutigeromorpha; within Lithobiomorpha	Within Scutigeromorpha; within Lithobiomorpha	n/a
DUF3472	Bacteria	1 or 2 (1)	In the stem of Pleurostigmophora or Amalpighiata^c^; or in Epimorpha and within Lithobiomorpha	Within Scolopendromorpha	SMAR002991, SMAR002992, SMAR002993, SMAR008653
GEOTX02	Fungi^d^	1 or 2	Geophilomorpha	Geophilomorpha	(group 1: SMAR012843, SMAR003678, SMAR004759); (group 2: SMAR012429, SMAR005429); group 3: SMAR014279; (group 4: SMAR009615, SMAR004692, SMAR001285, SMAR007268, SMAR006394, SMAR009617, SMAR010233)
PCPDP-like	Bacteria	1	Lithobiomorpha	Lithobiomorpha	n/a
unchar05	Fungi	2	Geophilomorpha, within Lithobiomorpha	Geophilomorpha	SMAR002275, SMAR004333, SMAR005016, SMAR002277, SMAR015613
unchar16	Oomycetes^d^	1	Unknown	Craterostigmomorpha	SMAR001399, SMAR001400

*n/a* The absence of these genes from the genome of *S. maritima* is uninformative because the HGT events happened elsewhere in the tree.

^a^The number in parentheses shows the number of times the gene was recruited into the venom proteome if that differs from the number of HGT events^23^.

^b^The identity of all paralogous loci is given. All are protein-coding loci with introns. Different paralog groups are indicated in parentheses.

^c^Due to uncertainty about centipede phylogeny^52^ we cannot distinguish between a single HGT into Pleurostigmophora (non-scutigeromorph centipedes), followed by a loss in Craterostigmomorpha, or a HGT into Amalpighiata (Lithobiomorpha + Epimorpha). Both these hypotheses suggest a loss in henicopid lithobiomorphs.

^d^The direction of transfer is ambiguous.

We bolster our conclusions about HGT with three ancillary criteria. First, all seven putative HGT gene families are present in both centipede venom gland transcriptomes and milked venom proteomes, which argues against them being accidental contamination. Second, each putative HGT gene is consistently expressed in the venom glands of multiple species collected from disparate geographic locations and habitats, which would not be expected if the sequences derived from local contaminants. Third, putative HGT sequences from different centipede species that are contaminants would be expected to group with related non-centipede sequences in different places in gene trees, rather than cluster together in a single clade. The strong clustering of the centipede sequences into well-supported clades in our gene trees, and the lack of the haphazard interleaving of putative donor and centipede sequences in any of our trees strongly suggest that the putative HGT genes are bona fide centipede sequences. Fulfilment of these ancillary criteria in addition to the phylogenetic nesting of the centipede sequences within paraphyletic groups of donor sequences, and the presence of five of the seven genes in the genome of *S. maritima*, further decreases the probability that our results are due to contamination or symbionts. Below we will discuss the full support for our conclusions for each of the genes, and the possibility that the genes that could not be checked against the *S. maritima* genome (centiPAD and PCPDP-like) could be due to symbionts.

### Bacterial pore-forming toxins transferred twice into centipedes

**Fig. 1:**
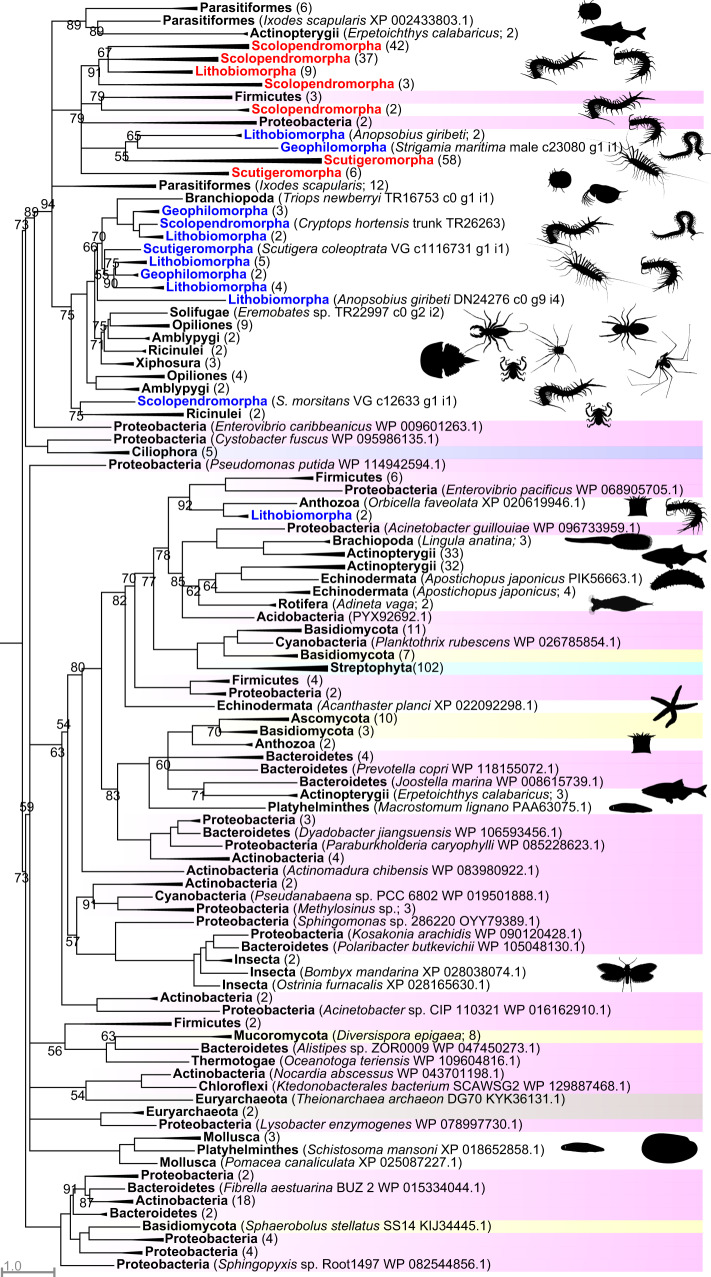
A maximum likelihood tree of β-PFTx sequences shows two clades of centipede β-PFTx sequences nested within a paraphyletic backbone of bacterial sequences. The tree shows that the centipede β-PFTxs originated from at least two bacterial HGTs, one along the centipede or arthropod stem lineage (represented by the clade at the top of the tree with 94% bootstrap support), and one within the lithobiomorph lineage (represented by the clade of two lithobiomorph sequences lower down the tree). Centipede sequences are coloured blue (present in transcriptomes) and red (present in transcriptomes and venom proteomes). Highlighted sequences are Bacteria (pink), Euryarchaeota (brown), Protozoa (purple), Fungi (yellow), and Streptophyta (cyan). Metazoan sequences are not highlighted. Collapsed clades have the number of included sequences indicated in parentheses. For the uncollapsed tree see Supplementary Fig. 1. The tree was reconstructed using the WAG + R7 model and is displayed as midpoint rooted. Bootstrap support values are shown for each clade, and clades with support <50% are collapsed into polytomies. Clades without bootstrap values have >95% support. Non-centipede images are sourced from Phylopic (www.phylopic.org; credit for the Opiliones image is with Gareth Monger: https://creativecommons.org/licenses/by/3.0/).

Centipede β-PFTxs were recruited into the ancestral centipede venom proteome, with subsequent losses from craterostigmomorph and geophilomorph venoms^23^. This gene family belongs to the bacterial aerolysin-like β-pore-forming toxin superfamily, which Moran et al.^10^ showed was transferred at least six times from bacteria to eukaryotes, including animals. We did not specifically design our phylogenetic dataset to provide a precise estimate of when and where all non-centipede HGTs occurred, but our findings agree with and extend their results. Although the structure of the gene tree is complex (Fig. 1; see Supplementary Fig. 1 for full tree), it shows that centipede β-PFTxs transferred twice from bacteria, once into the stem lineage of centipedes or arthropods (upper clade with 94% bootstrap support in Fig. 1), and once into the lithobiomorph lineage (located in the lower clade). This inference is supported by tree topology tests, which strongly reject monophyly of centipede β-PFTxs (see Supplementary Data 1). The structure of the tree, especially the complex interleaving pattern of bacterial, fungal, plant, and animal sequences in the lower clade of Fig. 1, suggests a complex history of multiple HGTs from bacteria to eukaryotes as well as losses of β-PFTx. For instance, an early transfer of β-PFTx into the arthropod stem lineage implies that it was lost in non-centipede myriapods and pancrustaceans, according to the current consensus on arthropod phylogeny^28^. However, the pronounced phylogenetic disjunction of the non-centipede animal sequences, and the lack of species from phyla with a strong representation in our custom (see “Methods”) and public sequence databases, such as arthropods, molluscs and nematodes, suggest that multiple HGTs have occurred from bacteria to animals. This interpretation is supported by tree topology tests that reject animal monophyly (see Supplementary Data 1).

The β-PFTxs of *S. maritima* map to three protein-coding paralogous genomic loci with introns (see Table 1). The phylogenetic distribution of these paralogs in three sub-clades of centipede sequences in the upper clade of Fig. 1 shows that the duplications that produced them happened early in the evolution of centipedes. However, β-PFTx and the other three protein families that were recruited into the ancestral centipede venom are absent from the venom proteome of *S. maritima*, which shows that streamlining of venom arsenals occurs alongside the recruitment and diversification of new components^23^.

The β-PFTxs produced by bacteria are virulence factors that contribute to pathogenicity by the lysing of host cells^29^. Interestingly, although they are not expressed in their tentacle venom, cnidarian β-PFTxs, which were horizontally transferred independently from those found in the venoms of centipedes and arachnids, are secreted into the pharynx and gut and aid digestion by disintegrating prey tissues, although their paralytic activity may also assist in prey immobilisation^10,30,31^. There is no experimental data for the role of β-PFTxs in centipedes, but they are believed to be at least in part responsible for the cytolytic activities of centipede venoms by the formation of transmembrane pores^32^. The great diversity of β-PFTx transcripts expressed in centipede venom proteomes, and the abundance of their expression^22,23,33,34^, suggest that β-PFTx likely plays important roles in prey immobilisation and processing.

### Bacterial exotoxins probable source of PCPDP-like proteins

**Fig. 2:**
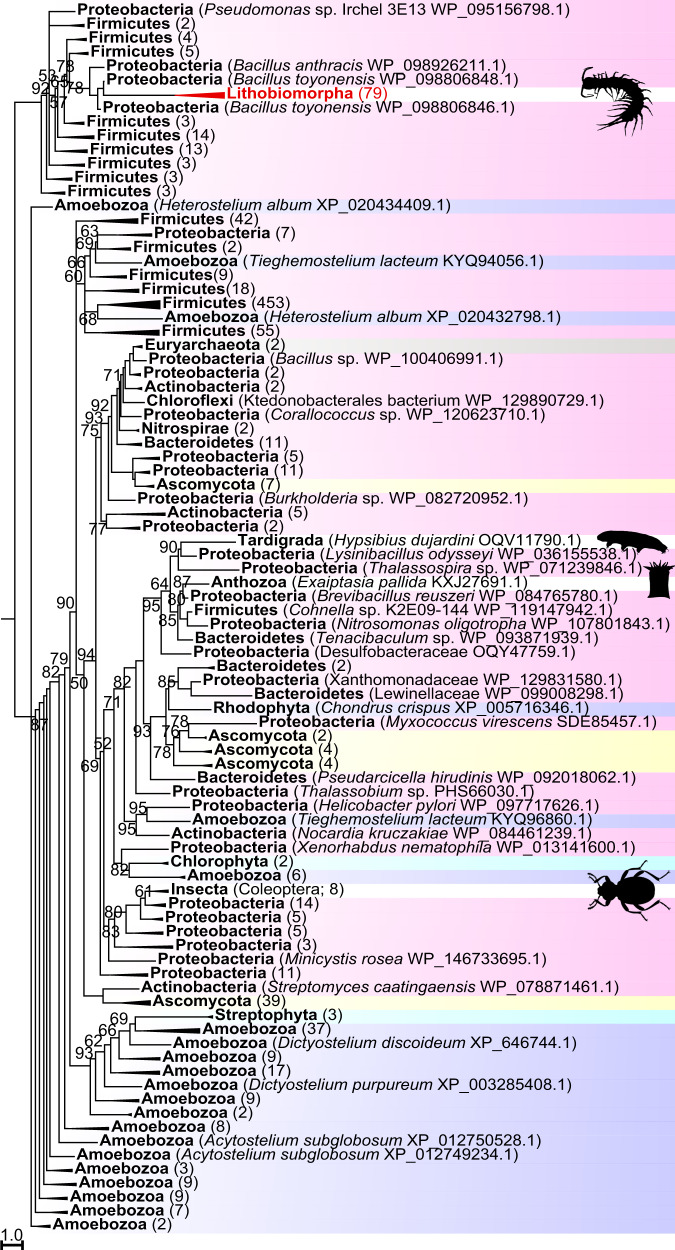
A maximum likelihood tree of PCPDP-like sequences shows a clade of centipede sequences nested within a paraphyletic backbone of bacterial sequences. It shows that the centipede sequences originated from a bacterial HGT into the lithobiomorph lineage. Centipede sequences are coloured red. Highlighted sequences are Bacteria (pink), Viridiplantae (cyan), Protozoa (purple), Euryarchaeota (brown), and Fungi (yellow). Metazoan sequences are not highlighted. Collapsed clades have the number of included sequences indicated in parentheses. For the uncollapsed tree see Supplementary Fig. 2. The tree was reconstructed using the VT + G4 model and is displayed as midpoint rooted. Bootstrap support values are shown for each clade, and clades with support <50% are collapsed into polytomies. Clades without bootstrap values have >95% support. Non-centipede images are sourced from Phylopic (www.phylopic.org).

We previously detected proteins with a pesticidal crystal protein domain (InterPro accession IPR036716) in the venom of *Lithobius forficatus*^23^. Homologous sequences are also present in transcriptomes of other centipedes from both lithobiomorph families (Lithobiidae: *L. forficatus*, *E. cavernicolus*; Henicopidae: *A. giribeti*, *P. validus*). All centipede PCPDP-like sequences cluster together in a strongly supported clade that is embedded in a paraphyletic backbone of bacterial PCPDP sequences (Fig. 2; see Supplementary Fig. 2 for full tree). The tree also shows that PCPDP-like proteins were independently transferred into beetles, a cnidarian and a tardigrade. This is supported by topological tree tests that strongly reject metazoan monophyly (see Supporting Data 1). The clade of centipede sequences includes species collected from the UK, Europe, North America, New Zealand, and Australia, and contains no interleaved bacterial sequences. This strongly suggests that the PCPDP-like sequences are bona fide centipede sequences rather than bacterial contaminants. Although on current evidence we cannot categorically reject the possibility that PCPDP-like protein is produced by symbionts, further evidence against this conclusion is that the centipede sequences are very distinct from their nearest bacterial relatives (see below), which is reflected by the relatively long branch leading to the centipede clade. Lastly, a morphological study of the venom system of *L. forficatus* found no evidence for bacterial symbionts in the venom producing and secreting tissues^35^.

The role of PCPDP-like proteins in centipede venom remains unknown, but our results suggest they evolved from bacterial insecticidal pore-forming toxins. The most intensely studied bacterial PCPDPs are pore-forming insecticidal endotoxins known as Cry toxins or δ-endotoxins, which are used widely in GM crops^36–39^. They are produced by *Bacillus* species in the *B. cereus* group^40,41^, especially *B. thuringiensis*, the entomopathogenic bacterium from which they were first described, and which feeds on the insects killed by the toxin^42^. Cry toxins consist of three conserved domains: an N-terminal domain of α-helices that is thought to be responsible for insertion into the cell membrane and pore formation, plus a middle and a C-terminal domain comprising β-sheets that are involved in receptor interactions, and which may confer host-specific toxicity^37,43,44^. Cry toxins are not secreted, but released as parasporal crystalline bodies through lysis of the spore-forming bacterial cell. The Cry toxin genes are located on plasmids, and plasmid transfer may explain why three-domain Cry proteins or genes have been found in several bacterial species outside the *B. cereus* group^37,41^.

In addition to three-domain Cry proteins our tree also contains sequences from a broad range of bacterial phyla that only contain a single Cry toxin domain, which in all cases is the pore-forming N-terminal domain. The centipede and other eukaryotic PCPDP-like sequences likewise only contain this N-terminal domain. A hint of how centipedes may have repurposed an insecticidal bacterial toxin into a venom protein is suggested by the most closely related bacterial sequences. All bacterial sequences that group together with the centipede sequences in the clade at the top of Fig. 2 also only contain the pore-forming N-terminal domain, and like the centipede sequences include a signal peptide region. This suggests that the bacterial proteins are exotoxins that are secreted from cells, like the centipede PCPDP-like proteins. Unlike the centipede sequences, the bacterial sequences in this clade also contain C-terminal cell wall-binding repeats (InterPro accession IPR018337), and/or a ricin B lectin domain (InterPro accession IPR000772). Cell wall-binding and ricin domains could help bind such putative exotoxins to bacterial or eukaryotic host cells, enabling the N-terminal perforating domain’s cytolytic action. The centipede PCPDP-like sequences may derive from such putative bacterial exotoxins, followed by loss of these target-binding domains. Alternatively, the centipede proteins may derive from a bacterial endotoxin, either a non-secreted single-Cry-toxin-domain protein, or a true three-domain Cry toxin, by adding a signal peptide. The low sequence similarity of the bacterial and centipede sequences makes it impossible to distinguish these possibilities. However, it is unlikely that only the N-terminal domain was transferred from bacteria and joined to a native centipede sequence because BLAST searches of the C-terminal region of the PCPDP-like sequences against centipede transcriptomes and the genome of *S. maritima* produce no hits.

### Two bacterial HGTs of centiPADS

**Fig. 3:**
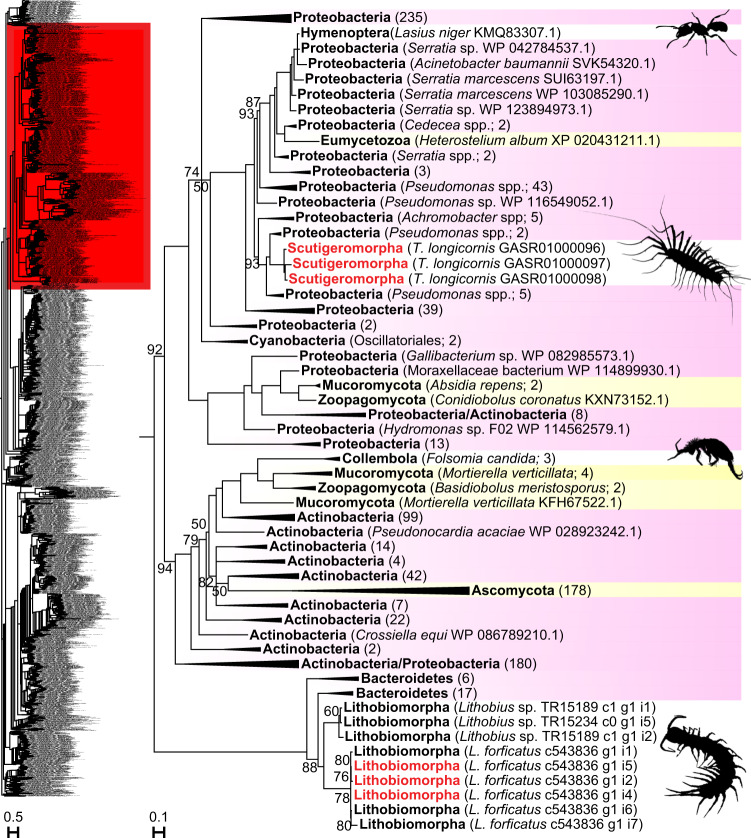
A maximum likelihood tree of PAD sequences shows two clades of centiPAD sequences nested within a paraphyletic backbone of bacterial sequences. The tree represents one clade nested within a larger tree (red highlight in inset) made up entirely of bacterial sequences. The tree shows that centiPADs originated from two bacterial HGTs, one within the lithobiomorph lineage, and one within the scutigeromorph lineage. Centipede sequences are in black (present in transcriptomes) and red (present in transcriptomes and venom proteomes). Highlighted sequences are Bacteria (pink) and Fungi (yellow). Metazoan sequences are not highlighted. Collapsed clades have the number of included sequences indicated in parentheses. For the uncollapsed tree see Supplementary Fig. 3. The tree was reconstructed using the WAG + G4 model and is displayed as midpoint rooted. Bootstrap support values are shown for each clade, and clades with support <50% are collapsed into polytomies. Clades without bootstrap values have >95% support. Collembolan image was sourced from Phylopic (www.phylopic.org; credit for the Collembola image is with Birgit Lang: https://creativecommons.org/licenses/by/3.0/).

We previously detected the enzyme peptidylarginine deiminase (PAD) in the venoms of two distantly related centipede species, *Thereuopoda longicornis* (order Scutigeromorpha), and *Lithobius forficatus* (order Lithobiomorpha)^22,23^. Our phylogenetic analysis shows that these sequences are positioned in different parts of the tree, separated by many strongly supported nodes. Hence, centiPADs are the result of two HGTs from different bacterial phyla. *T. longicornis* centiPAD derives from Gammaproteobacteria, while *L. forficatus* centiPAD derives from Bacteroidetes (Fig. 3; see Supplementary Fig. 3 for full tree). The centiPAD sequences are deeply nested within a large tree of bacterial sequences, confirming that human and bacterial PADs are evolutionarily unrelated^45,46^. Interestingly, the nesting of four fungal branches and a sequence derived from the black garden ant *Lasius niger* within the paraphyletic backbone of bacterial sequences suggest that PAD was transferred multiple times from bacteria to other eukaryotes as well.

We cannot categorically reject the possibility that centiPADs are produced by bacterial symbionts, which, if true, would be the second example of an animal venom component being produced by bacteria^47^. However, the balance of evidence suggests that centiPADs are a bona fide centipede gene family. CentiPAD is a prominent component of the venom proteome of *T. longicornis*^22^, which is incompatible with it being due to accidental bacterial contamination. The sequences of *T. longicornis* can be up to 78% similar to the most closely related bacterial PAD sequences, but they share unique features that separate them from all bacterial sequences grouped in the same clade. Compared to related PAD sequences derived from the gammaproteobacterial genera *Pseudomonas, Cedecea*, *Aeromonas*, *Serratia, Stenotrophomonas*, and *Acinetobacter*, as well as the betaproteobacterial genera *Achromobacter*, *Paucibacter*, and *Undibacterium*, the centiPAD sequences uniquely have a Met593 and a single amino acid deletion at position 606 (see alignment in Supplementary Data 2). These distinctive differences further support the conclusion that the *T. longicornis* centiPADs are bona fide centipede sequences.

The *Lithobius* centiPAD sequences group together in a strongly supported clade without interleaving bacterial sequences. This clade groups sequences from specimens collected in the UK, continental Europe, and North America^23,48,49^. This strongly suggests that they are bona fide centipede sequences, a conclusion in line with the lack of evidence for microorganisms in the venom system of *L. forficatus*^35^. The European sequences (represented by UK sequences; an identical German sequence was excluded) form a sister clade to the American sequences. Because the latter were not determined to species by the original collectors^48^, it is unclear if they are *L. forficatus*, which was imported from Europe to North America some time before the end of the 19^th^ century^50^. CentiPAD is absent from the transcriptomes of other lithobiomorph species: *Eupolybothrus cavernicolus*, *Paralamyctes validus*, and *Anopsobius giribeti*^51,52^. With the exception of *E. cavernicolus*, no venom glands were included in these transcriptomes, so these could be false negatives. However, the mean GC content of the UK centiPAD sequences is on the edge of the first quartile of all non-HGT venom protein sequences (0.385 vs. 0.384) from all centipede species analysed in our previous study^23^ (see Supplementary Data 3), which suggests that the HGT probably occurred relatively recently.

A recent transfer is also likely for the *T. longicornis* centiPADs. The mean GC content of the three *T. longicornis* centiPAD sequences (0.588) is extremely skewed in the other direction and falls outside the 99th percentile (0.557) of all non-HGT centipede venom protein sequences. This skew and the sequence similarity of the centipede and bacterial sequences indicate that this HGT may have happened relatively recently. The absence of centiPAD sequences from the transcriptomes of other scutigeromorphs (*Scutigerina weberi*, *Sphendononema guilgingii*, and *Scutigera coleoptrata*)^23,52^ provides further support for a relatively recent HGT. Since only the transcriptome of *S. coleoptrata* contains venom gland tissue the other two may be false negatives. We consider this unlikely, however, because they represent different scutigeromorph families, while *S. coleoptrata* and *T. longicornis* belong to the family Scutigeridae. The unique presence of centiPAD in *T. longicornis* therefore suggests that this gene was transferred after its lineage split off from that of *S. coleoptrata*, which is estimated to have happened by about 200 million years ago^53^.

Bacterial PAD converts peptidylarginine into citrulline residues, and the effects of this process have been most intensely investigated for the pathogenic bacterium *Porphyromonas gingivalis*. *Porphyromonas* PAD (PPAD) is a major virulence factor that causes inflammatory gum disease, and is a risk factor for rheumatoid arthritis^45,46,54,55^. How PPAD contributes to pathogenicity is an active area of research, and it may include defusing the host’s immune system and the formation of protective biofilms^55,56^. It is unknown what role centiPADs play in centipede venom but modulating the activity of other venom components through posttranslational modification is one possibility. The centiPAD sequences from both species have conserved the five catalytic residues responsible for PPAD’s enzymatic activity (Asp1372, His2321, Asp2323, Asn2928, Cys4010 in the PAD alignment in Supplementary Data 5), but they have changed two residues that determine substrate specificity of bacterial PADs^46^.

### One or two bacterial HGTs of DUF3472-domain proteins

**Fig. 4:**
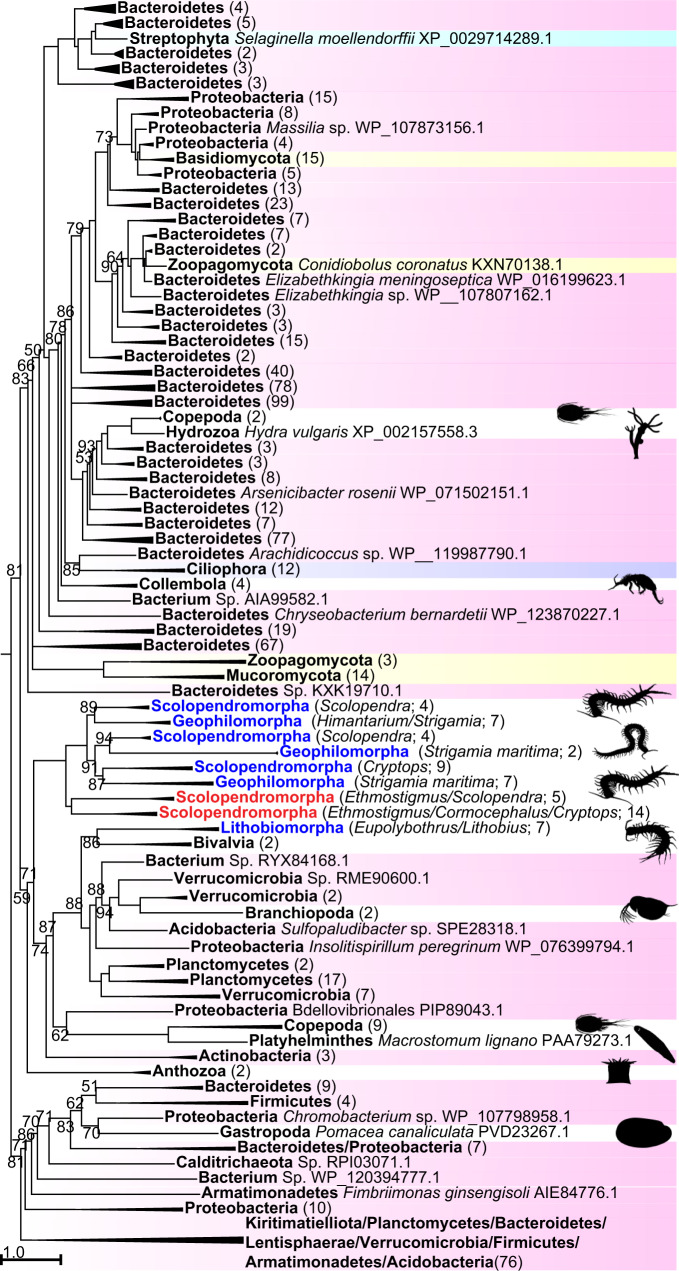
A maximum likelihood tree of sequences with DUF3472-domains shows two clades of centipede sequences nested within a paraphyletic backbone of bacterial sequences. This suggests that the centipede sequences may have originated from two bacterial HGTs, one into the epimorphan lineage, and one within the lithobiomorph lineage. However, tree topology tests cannot reject centipede monophyly (see Supplementary Data 1). Centipede sequences are coloured blue (present in transcriptomes) and red (present in transcriptomes and venom proteomes). Highlighted sequences are Bacteria (pink), Protozoa (purple), Streptophyta (cyan), and Fungi (yellow). Metazoan sequences are not highlighted. Collapsed clades have the number of included sequences indicated in parentheses. For the uncollapsed tree see Supplementary Fig. 4. The tree was reconstructed using the WAG + R10 model and is displayed as midpoint rooted. Bootstrap support values are shown for each clade, and clades with support <50% are collapsed into polytomies. Clades without bootstrap values have >95% support. Copepod image was sourced from Phylopic (www.phylopic.org; credit for the Collembola image is with Birgit Lang: https://creativecommons.org/licenses/by/3.0/).

Proteins with a domain of unknown function DUF3472 (InterPro accession IPR021862) are found in the venom of several species of scolopendromorph centipedes, as well as in geophilomorph and lithobiomorph venom gland and non-venom gland transcriptomes^23,33,34,57,58^. In addition, many of the sequences have an N-terminal DUF5077 domain (InterPro accession IPR031712). Our phylogenetic analysis places the centipede sequences into two clades separated by bacterial and metazoan sequences (Fig. 4; see Supplementary Fig. 4 for full tree). This suggests that DUF3472-domain proteins may have transferred twice from bacteria to centipedes, once into the lineage leading to Epimorpha (geophilomorphs and scolopendromorphs), and once into lithobiid lithobiomorphs. Topological tree tests cannot statistically reject centipede monophyly, but do reject metazoan monophyly (see Supplementary Data 1). This shows that DUF3472-domain proteins have been transferred from bacteria to animals multiple times, like β-PFTxs and PCPDP-like proteins. DUF3472-domain proteins from *S. maritima* map to four protein-coding genomic loci with introns (see Table 1), and the tree suggests that these and the multiple copies found in scolopendromorphs are the result of several rounds of gene duplication.

### Multiple HGTs between fungi, oomycetes and centipedes

**Fig. 5:**
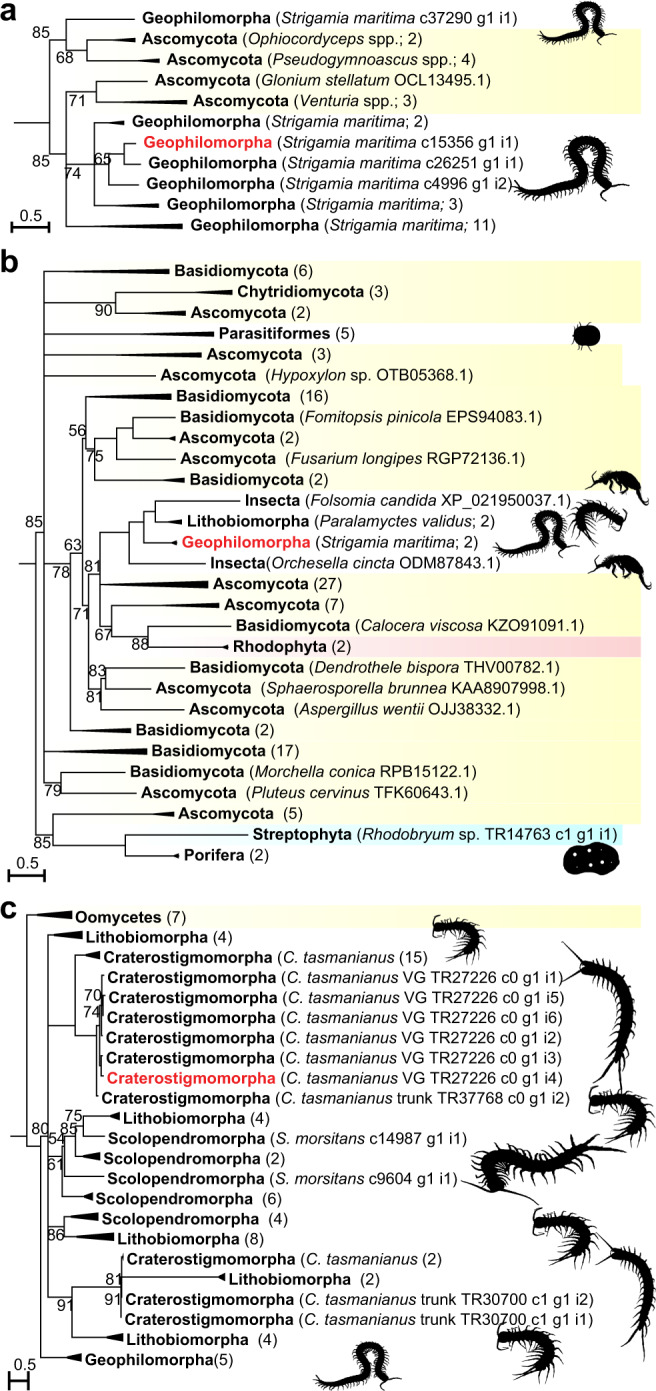
Maximum likelihood trees showing eukaryotic HGTs between fungi, oomycetes, and centipedes. **a** Tree of GEOTX02 homologues showing that the centipede sequences are distributed across two clades, and interleaved with ascomycete sequences. The direction and number of HGTs (one or two) is uncertain. The tree was reconstructed using the VT + I + G4 model and is midpoint rooted. For the uncollapsed tree see Supplementary Fig. 5. **b** Tree of unchar05 homologues showing the four centipede sequences grouping in a clade with two collembolan sequences, nested within a paraphyletic backbone of fungal sequences. The tree shows that the centipede sequences likely originated from two fungal HGTs, one into the geophilomorph lineage, and one within the lithobiomorph lineage. The tree was reconstructed using the WAG + R5 model and is midpoint rooted. For the uncollapsed tree see Supplementary Fig. 6. **c** Tree of unchar16 homologues showing a clade of centipede sequences that is the sister group to a clade of oomycete sequences. The direction of HGT is unclear. The tree was reconstructed using the VT + R3 model and is rooted with the oomycete sequences. For the uncollapsed tree see Supplementary Fig. 7. For each tree, bootstrap support values are shown for each clade and clades with support <50% are collapsed into polytomies. Clades without bootstrap values have >95% support. Centipede sequences are coloured black (present in transcriptomes) and red (present in transcriptomes and venom proteomes). Highlighted sequences are Fungi (yellow), Rhodophyta (reddish brown), and Streptophyta (cyan). Metazoan sequences are not highlighted. Collapsed clades have the number of included sequences indicated in parentheses. Non-centipede images are sourced from Phylopic (www.phylopic.org; credit for the Collembola images is with Birgit Lang: https://creativecommons.org/licenses/by/3.0/).

Centipedes not only express four gene families in their venoms that were horizontally transferred from bacteria, but also three gene families that find their nearest homologues in fungi and oomycetes (water molds). GEOTX02 is a peptide present in the venom of the geophilomorph *S. maritima*, and similar sequences with a corresponding cysteine framework are restricted to a few species of ascomycete fungi. The sequences exhibit two distinct cysteine patterns, with 8 or 10 cysteine residues in the mature domain of the peptide, with the latter being restricted to the top clade in the tree with 85% bootstrap support (Fig. 5a; see Supplementary Fig. 5 for full tree). The centipede sequences map to four paralogue groups of genes with introns in the genome of *S. maritima*, with the clade with 74% bootstrap support representing paralogue groups 1–3 and the collapsed clade of eleven *S. maritima* sequences representing paralogue group 4 (see Table 1). The tree suggests that the centipede sequences with the two different cysteine patterns may have resulted from two HGTs, although a tree topology test cannot reject centipede monophyly (see Supplementary Data 1), and the direction of these horizontal transfers remains uncertain. The ascomycetes included in the tree belong to two orders (Dothideomycetes and Sordariomycetes) and include species known to infect animals and plants. The transfers therefore possibly involved an arthropod-infecting ascomycete as either a donor or recipient of GEOTX02.

Unchar05 is another venom protein family that has been horizontally transferred between centipedes and fungi. Unchar05 is present in the venom of *S. maritima* but is also found in a trunk transcriptome of the lithobiomorph *Paralamyctes validus*. The two unchar05 transcripts identified in the venom proteome of *S. maritima* map to a protein-coding genomic locus with introns (SMAR002275), which is one of five paralogous loci (see Table 1), four of which are expressed as transcripts in the venom gland of *S. maritima*. Our phylogenetic analysis (Fig. 5b; see Supplementary Fig. 6 for full tree) shows that unchar05 was transferred into centipedes from fungal donors. The centipede sequences group in a clade with sequences from two species of springtails, *Folsomia candida* and *Orchesella cincta*, but neither the centipede nor the springtail sequences are monophyletic. This taxonomic interleaving of sequences and the phylogenetic disjunction between the centipede species suggest that unchar05 horizontally transferred twice into centipedes. This may also be true for the springtails, where unchar05 homologues are found in at least two different families, and whose genomes contain hundreds of genes of HGT origin^5,6^. Moreover, the tree also contains a well-supported clade of mite sequences that includes species that have also previously been shown to have received horizontally transferred fungal genes^3^.

Although a tree topology test cannot reject metazoan monophyly in our tree (see Supplementary Data 1), we consider the alternative hypothesis of a single early HGT of unchar05 into animals followed by rampant losses to be less convincing. To explain the large phylogenetic disjunction of the sequences on various levels—within centipedes, within insects, and within animals—would require an immense amount of gene loss throughout the animal kingdom to leave just this handful of metazoan homologues, several of which represent taxa already known to be recipients of horizontally transferred genes.

The third gene family that has probably undergone eukaryotic HGT is Unchar16. It encodes cysteine-rich proteins found in the venom gland and non-venom gland transcriptomes of pleurostigmophoran (non-scutigeromorph) centipedes, as well as in the venom of the craterostigmomorph *Craterostigmus tasmanianus*. Unchar16 maps to two protein-coding paralogous loci with introns in the genome of *S. maritima* (see Table 1). Our searches identified small secretory proteins from plant-parasitic oomycetes as homologues based upon sequence similarity and corresponding cysteine patterns. Unchar16 has undergone marked sequence evolution in centipedes, and all centipede sequences group in a well-supported clade when the tree is rooted with oomycetes (Fig. 5c; see Supplementary Fig. 7 for full tree). However, two different HGT scenarios may explain the data depending on how the tree is rooted.

Oomycetes originated at about the same time as centipedes, about 430 million years ago^59^, so a HGT between early oomycete and centipede lineages is possible if unchar16 was transferred from oomycetes into the stem lineage of pleurostigmophoran centipedes. However, the early evolutionary history of oomycetes and the taxonomic distribution of oomycete unchar16 homologues casts doubt on this scenario. Early diverging oomycete lineages are exclusively marine, with the exception of the genus *Haptoglossa*^60,61^. Moreover, the oomycete homologues of unchar16 that we identified belong to the predominantly terrestrial oomycete order Peronosporales, which is a lineage that evolved much later, in the early Mesozoic about 225-190 million years ago^59^. This suggests that unchar16 may have horizontally transferred much more recently from centipedes into the peronosporalean lineage of oomycetes—the reverse transfer would require independent HGTs into all four pleurostigmophoran centipede lineages. HGT is known to have contributed to the evolution of oomycete secretomes^62,63^, but which centipede lineage functioned as a donor of unchar16 in this scenario remains unclear given the lack of resolution in the tree. On the balance of available evidence, we prefer this second scenario, but hope that future research will shed further light on this tantalizing riddle.

### HGT is a potentially major mechanism of venom evolution

**Fig. 6:**
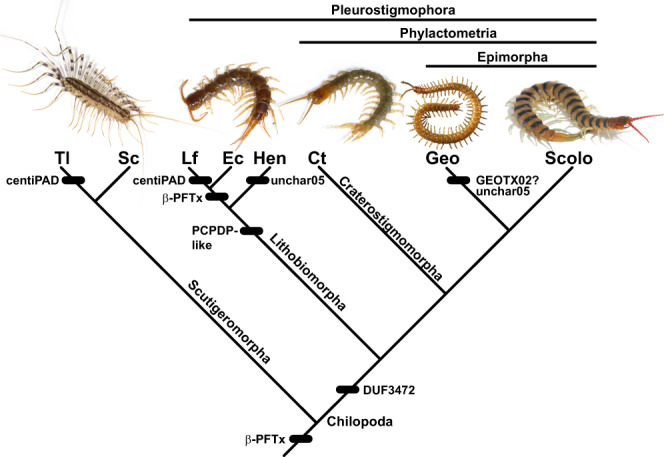
Phylogenetic distribution of centipede venom gene families horizontally transferred from bacteria and fungi. ‘?’ indicates uncertainty in the direction of transfer. Taxon abbreviations are as follows. Tl: *Thereuopoda longicornis*; Sc: *Scutigera coleoptrata*; Lf: *Lithobius forficatus*; Ec: *Eupolybothrus cavernicolus*; Hen: Henicopidae; Ct: *Craterostigmus tasmanianus*; Geo: Geophilomorpha; Scolo: Scolopendromorpha.

Our results suggest that HGT has been a key factor in the expansion and diversification of centipede venoms in all five orders throughout their evolutionary history (Fig. 6). Because genes were horizontally transferred from bacteria and fungi both deeply and repeatedly in the phylogeny of centipedes, we expect that the vast majority of centipede species produce venoms that include multiple horizontally transferred components. Because proteotranscriptomic venom profiles are currently available for only a small number of the more than 3,100 described species of centipedes, new insights into the full impact of HGT on centipede venom evolution are likely to emerge from future studies.

Our findings increase the number of animal venom protein families with well-supported HGT origins from three to at least eight, which increases the number of known HGT events stocking venom arsenals from five or six to at least thirteen. We show that centipedes are the first known animals with venoms used for predation and defence that contain multiple gene families derived from HGT. It is likely that HGT contributions to venom evolution are a much more widespread phenomenon. More than a hundred animal lineages have evolved venoms^8^, and recent proteotranscriptomic studies of venoms from a wide range of taxa have identified substantial numbers of protein families with few or no known metazoan homologues (e.g.^16–21^). Such gene families are especially promising for identifying new HGT candidates, but this requires a targeted approach, like the one adopted here, that goes beyond the standard BLAST-based annotation pipelines commonly used in venom profiling studies.

Our findings expand the insights generated by previous research into how HGT can increase the adaptive versatility of organisms^1,2^. Our results suggest that HGT can allow a venomous lineage to reap the immediate adaptive benefits of genes evolved in unrelated lineages if the gene products are preapted to a venom function. For instance, the incorporation of a cytolytic bacterial pore-forming toxin, such as β-PFTx, into the ancestral centipede venom may have conferred an immediate functional benefit, for example in prey immobilisation. In this scenario, the pore-forming activity of the bacterial protein is a preaptation that would have allowed the protein to take on this function in the centipede venom without first having to evolve modifications to gain a venom function. This parallels, for example, the use of detoxifying enzymes by herbivorous arthropods that were horizontally transferred from, and similarly used, by bacterial and fungal donors^64^. The selective benefit of the horizontal transfer of β-PFTx into the earliest centipede venom could have been substantial because it is just one of two putative toxins that could have been involved in prey immobilization. The other three protein families that we reconstructed as present in the ancestral centipede venom are metalloprotease family M12A, glycoside hydrolase family 18, and centipede CAP1 (*c*ysteine-rich secretory proteins, *a*ntigen 5 and *p*athogenesis-related protein family 1), which is the second putative venom toxin^23^. The recruitment of β-PFTx into the ancestral centipede venom represents the first known example of HGT contributing to the evolutionary origin of venom in a lineage. Horizontal transfer could therefore have been a crucial step in setting centipedes on the selective trajectory that eventually led to the complex venoms of modern species.

The fact that the centipede venom homologues of the three horizontally transferred bacterial virulence factors for which there are functional data have retained the structural domains involved in pore-formation (β-PFTx and PCPDP-like proteins), or conserved the catalytic sites involved in enzymatic action (centiPAD), is consistent with a continuity of function and adaptive value from donor to recipient taxa. Moreover, the gene duplications that have subsequently occurred in the genome-confirmed gene families underlines a commonly observed feature of the route to the functional consolidation and diversification of horizontally transferred genes^2^. Our results therefore show that HGT can provide a fast track channel for the evolution of novelty by the exaptation of bacterial weapons for new functions in animal venoms.

## Methods

### Initial identification of HGT candidates

We used the transcriptomic and proteomic data from Undheim et al.^22^ and Jenner et al.^23^ to identify HGT candidates expressed in centipede venom glands and venoms. Manual inspection of BLAST results generated for these studies for more than 90 venom protein families yielded sixteen protein families with either non-metazoan hits, and/or few or no metazoan hits (β-PFTx, centiPAD, CHILOTX01, DUF3472, GEOTX02, LTHTX01, LTHTX03, PCPDP-like, SCTX01, SCTX02, SLPTX02, SLPTX04, SLPTX06, SLPTX30, unchar05, and unchar16.). We performed a protein BLAST search of these HGT candidate sequences against a local version of the NCBI non-redundant (nr) database (downloaded from the NCBI FTP Server ftp://ftp.ncbi.nlm.nih.gov/ on 5 June 2019) with BLAST version 2.4.0, and an *E*-value cut off of e-3. Significant hits against non-metazoan sequences were found for β-PFTx, centiPAD, DUF3472, GEOTX02, PCPDP-like, unchar05, unchar16, and SLPTX02. These BLAST results were submitted to the Alienness web server (http://alienness.sophia.inra.fr/cgi/index.cgi), which is a tool designed to detect HGT candidates^65^. Alienness calculates an Alien Index for each query sequence based on the *E*-values of the best BLAST hits to putative candidate donors (non-metazoan) and recipient (metazoan) taxa. The following taxa and taxon codes were excluded from the Alien Index calculations as self-hits for the different protein families that generated positive Alien Indices: β-PFTx: *Cormocephalus westwoodi* (1096223), *Ethmostigmus rubripes* (62613), *Lithobius forficatus* (7552), *Scutigera coleoptrata* (29022), *Scolopendra alternans* (1329349), *Sco. morsitans* (943129), *Sco. subspinipes* (55038), *Thereuopoda longicornis* (353555), *Ixodes scapularis* (6945), *Limulus polyphemus* (6850), *Strigamia maritima* (126957), *Cryptops hortensis* (1268897), *Acuclavella merickeli* (703423), *Damon variegatus* (317683), *Cryptocellus becki* (1642531), *Lithobius* (7551), centipedes (7540); centiPAD: *L. forficatus* (7552), *T. longicornis* (353555), centipedes: 7540; DUF3472: *S. maritima* (126957), *E. rubripes* (62613), *Himantarium gabrielis* (241672), *Sco. morsitans* (943129), *Sco. subspinipes* (55038), *C. westwoodi* (1096223), *Cryptops hortensis* (1268897), *L. forficatus* (7552), centipedes (7540); GEOTX02: *S. maritima* (1256957), centipedes (7540); unchar05: *S. maritima* (126957), centipedes (7540); unchar16: *Craterostigmus tasmanianus* (60162), *Sco. morsitans* (943129), *L. forficatus* (7552), *S. maritima* (126957), centipedes (7540); SLPTX02: centipedes (7540), *L. forficatus* (7552), *Scu. coleoptrata* (29022), *C. tasmanianus* (60162), *C. hortensis* (1268897), *H. gabrielis* (241672), *Lithiobius* (7551), *Sco. Subspinipes* (55038), *E. rubripes* (62613). Results are summarized in Supplementary Data 7. The PCPDP-like gene family didn’t generate any BLAST hits. However, because the sequences contain an insecticidal delta-endotoxin domain known only from bacteria we included this gene family in our analyses as well. SLPTX02 was dropped from further consideration because the broad phylogenetic distribution of homologues suggests an ancient origin of this protein family.

### Construction of phylogenetic datasets

All analyses were performed on amino acid translations of the transcriptome sequences. We used HMMER v3.2.1 (http://hmmer.org) with default settings to generate Hidden Markov Models for each of the seven venom protein families with possible non-metazoan origins, and retained all hits above HMMER’s default inclusion threshold (per-sequence *E*-value of 0.01). Geneious version 11.1.5 (https://www.geneious.com) was used to construct alignments for training HMMER profiles, using the local paired iterative alignment method (L-INS-i) in MAFFT v7.450^66^ (see Supplementary Data 5 and 6 for the alignments and profiles). We included in these alignments all the full-length centipede sequences that we generated for these gene families in our previous studies^22,23^. For β-PFTxs and the PCPDP-like gene family, we additionally included a selection of outgroup taxa, and the PCPDP-like alignment was limited to the N-terminal Cry toxin domain. We used the HMMER profiles to search against a local fasta version of the nr database (downloaded from the NCBI FTP Server ftp://ftp.ncbi.nlm.nih.gov/ on 21 May 2019) for possible homologues of the centipede sequences. We also used these profiles to search a previously published^67^ custom database of 155 de novo assembled and translated transcriptomes obtained from the NCBI Sequence Read Archive (SRA), representing 134 animal species, with 121 arthropod species including eight millipede whole body and eight centipede whole body or trunk transcriptomes, as well as seven species of fungi, plants, and choanoflagellates (see Supplemental Table S2 in Dash et al.^67^). This database was supplemented with assembled transcriptomes for the centipedes *Paralamyctes validus*, *Anopsobius giribeti*, *Scutigerina weberi*, and *Sphendononema guildingii*^52^. Complementing these transcriptome-based sequence data we used the HMMER profiles to search for homologues in 25 metazoan Ensembl genomes (http://ensemblgenomes.org) representing these major lineages: Cnidaria: *Thelohanellus kitauei*, *Nematostella vectensis*; Placozoa: *Trichoplax adhaerens*; Ctenophora: *Mnemiopsis leidyi*; Deuterostomia: *Strongylocentrotus purpuratus*; Rotifera: *Adineta vaga*; Brachiopoda: *Lingula anatina*; Mollusca: *Octopus bimaculoides*, *Crassostrea gigas*, *Lottia gigantea*; Annelida: *Capitella teleta*, *Helobdella robusta*; Nematoda: *Pristionchus pacificus*, *Caenorhabditis elegans*; Arthropoda, Arachnida: *Ixodes scapularis*, *Sarcoptes scabiei*, *Tetranychus urticae*, *Stegodyphus mimosarum*; Arthropoda, Pancrustacea: *Daphnia pulex*, *Lepeophtheirus salmonis*, *Folsomia candida*, *Nasonia vitripennis*, *Apis mellifera*, *Megaselia scalaris*, *Anopheles gambiae*.

Once a comprehensive list of homologues was generated, we removed identical sequences using CD-HIT v4.6^68^, and examined and filtered false positives using CLC Main WorkBench v7 (Qiagen, Aarhus, Denmark) and Geneious v11.1.5 (https://www.geneious.com). In the case of β-PFTx, we also filtered the non-chilopod sequences with CD-HIT to only include sequences with <95% sequence identity due to a large number of identified unique homologues (2164 sequences). To create datasets of manageable size for PAD, PCPDP, and DUF3472, while retaining a broad net for capturing putative donor taxa and sampling metazoan homologues, the identified homologues were first sorted to Kingdom and then filtered with CD-HIT to include only sequences with <90% (bacteria, fungi, protists, and viruses) or 70% sequence identity (non-myriapod animals, Archaea, and plants). Due to the large number of PAD homologues still retained by this approach (6716 sequences), we then removed all sequences with a pairwise distance to any chilopod sequence >0.5.

The remaining sequences were aligned using the local paired iterative alignment method (L-INS-i) in MAFFT v7.304b^66^. For the alignment of GEOTX02, we first aligned the structurally important conserved cysteines^69^, and then used the MAFFT regional alignment ruby script to align the pre-, inter-, and post-cysteine regions by local paired iterative alignment method as above. All alignments are included in Supplementary Data 4. We used InterProScan^70^ as implemented in Geneious v11.1.5 (https://www.geneious.com) to generate protein domain annotations for all alignments (see Supplementary Data 8). The evolutionary history of each protein family was then reconstructed using a molecular phylogenetic approach. The most appropriate evolutionary model was determined using ModelFinder^71^, before using IQ-TREE v1.5.5^72^ to reconstruct molecular phylogenies by maximum likelihood, and estimating branch support values by ultrafast bootstrap using 10,000 replicates^73^. Because taxonomic outgroups could not be designated we used midpoint rooting to root the trees. Trees were visualised in Archaeopteryx v0.9921^74^.

### Tree topology tests

Likelihood ratio tests of constrained tree topologies were performed in IQ-TREE 2^75^, which implements several different tests. Each test compares support for the unconstrained optimal maximum likelihood tree with a tree that constrains the monophyly of selected taxa as a polytomy. Results are given in Supplementary Data 1. Mesquite 3.61 (build 927)^76^ was used to build constraint topologies.

### Mapping of genes against the *Strigamia maritima* genome

All sequences belonging to candidate HGT gene families present in the venom gland transcriptome and venom proteome of *S. maritima* were mapped against its genome using the TBLASTN search function with an E-value cut off of e-5 on the EnsemblMetazoa web portal at http://metazoa.ensembl.org/Strigamia_maritima/Info/Index (last accessed 1 April 2020).

### GC contents analyses

We used CLC Main WorkBench v7 (Qiagen, Aarhus, Denmark) to calculate GC frequencies of all nucleotide sequences encoding centipede venom proteins and peptides published by Jenner et al.^23^. Descriptive statistics were calculated with GraphPad Prism v8.4.1 (GraphPad Software, La Jolla California USA, www.graphpad.com), and are available as Supplementary Data 3.

### Reporting summary

Further information on research design is available in the Nature Research Reporting Summary linked to this article.

## Supplementary information

Supplementary Information

Peer Review File

Reporting Summary

Description of Additional Supplementary Files

Supplementary Data 1

Supplementary Data 2

Supplementary Data 3

Supplementary Data 4

Supplementary Data 5

Supplementary Data 6

Supplementary Data 7

Supplementary Data 8

## Data Availability

The transcriptomic custom database used in this study is available in the NIRD Research Data Archive with identifier 10.11582/2020.00067 [https://l.antigena.com/l/-XpFdcjUOuQ3kwVUGwNUCcawa65ouPHcGAU1UyZ4_G8tW7vXlL81qJ8DGsAVtkPIn4FKNoqN6enY799zIGURLtFK78EEeGN7Vjv6rkUj6QgiCaGMuFn2wNUwN3avmVFclTjxYAKWjK8PqF7hKgWurRu8L2F61L~640JO9Vwr1vwCQm]. The transcriptome data from Undheim et al.^22^ are available at the National Center for Biotechnology Information (NCBI) under bioprojects PRJNA200639, PRJNA200641, PRJNA200753, PRJNA200640, and PRJNA213032, while individually curated sequences are available in the Transcriptome Shotgun Assembly Sequence Database (https://www.ncbi.nlm.nih.gov/nuccore/) as GASI01000001–GASI01000195, GASL01000001–GASL01000050, GASK01000001–GASK01000051, GASH01000001–GASH01000185, and GASR01000001–GASR01000119. Undheim et al.’s proteomic evidence are available as supplementary files associated with the original publication. The assembled transcriptomes from Jenner et al.^23^ are available via the Natural History Museum’s Data Portal (https://data.nhm.ac.uk/dataset/evolution-of-centipede-venoms; last accessed 30 June 2020). 9), while the proteomic data are available in the ProteomeXchange Consortium via the PRIDE partner repository with the data set identifier PXD013356. In addition, we used the following databases: NCBI non-redundant (nr) database (https://www.ncbi.nlm.nih.gov), EnsemblMetazoa (https://metazoa.ensembl.org/index.html), and the databases in the InterPro Consortium (https://www.ebi.ac.uk/interpro/).
