# Supplementary material for: Phylogenetic analyses suggest centipede venom arsenals were repeatedly stocked by horizontal gene transfer
Source: Nat Commun. 2021 Feb 5;12:818. doi: 10.1038/s41467-021-21093-8 (PMC7864903; doi:10.1038/s41467-021-21093-8)
Supplement: Supplementary file 1 — Supplementary Information [file 41467_2021_21093_MOESM1_ESM.pdf]

This Supplementary Information contains Supplementary Figures 1-7. The full legend for each tree is located beneath it.

Supplementary Figure 1: Maximum likelihood tree of ß-PFTx sequences.
Supplementary Figure 2: Maximum likelihood tree of PCPDP sequences.
Supplementary Figure 3: Maximum likelihood tree of PAD sequences.
Supplementary Figure 4: Maximum likelihood tree of DUF3472 sequences.
Supplementary Figure 5: Maximum likelihood tree of GEOTX02 sequences.
Supplementary Figure 6: Maximum likelihood tree of unchar05 sequences.
Supplementary Figure 7: Maximum likelihood tree of unchar16 sequences.

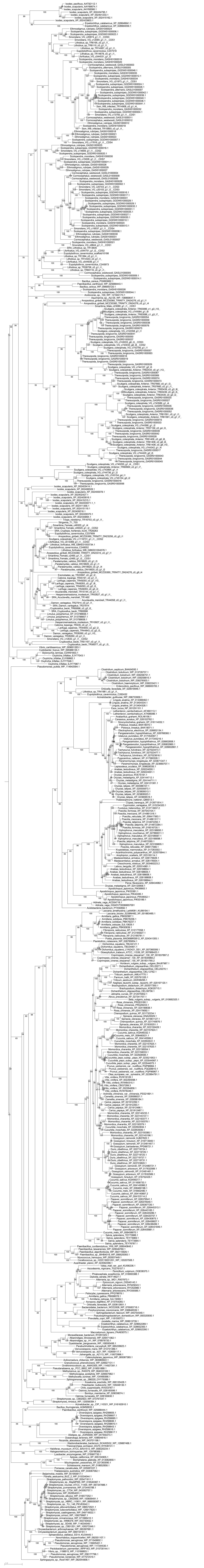

Supplementary Fig. 1. Maximum likelihood tree of ß-PFTx sequences. The tree was reconstructed using the WAG+R7 model and is displayed as midpoint rooted. Bootstrap support values are shown at each node. Nodes with support <50% are collapsed into polytomies.

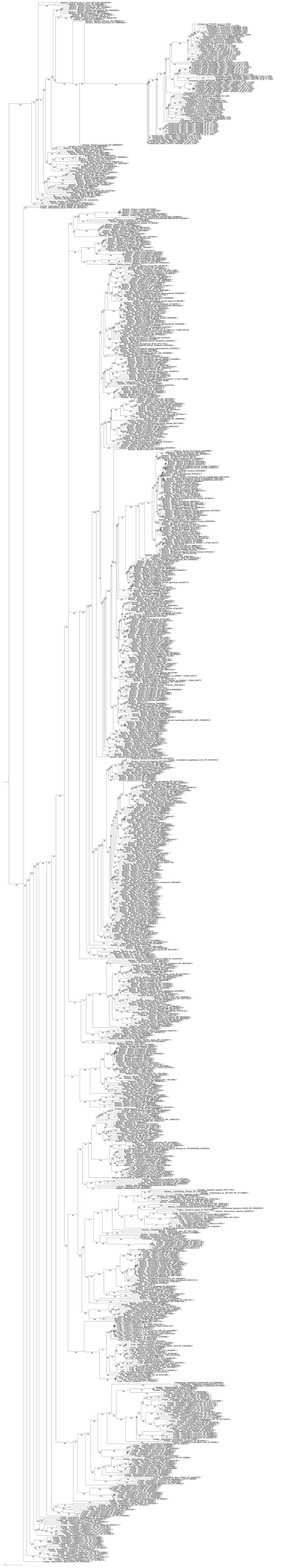

Supplementary Fig. 2. Maximum likelihood tree of PCPDP sequences. The tree was reconstructed using the VT+G4 model and is displayed as midpoint rooted. Bootstrap support values are shown at each node. Nodes with support <50% are collapsed into polytomies.

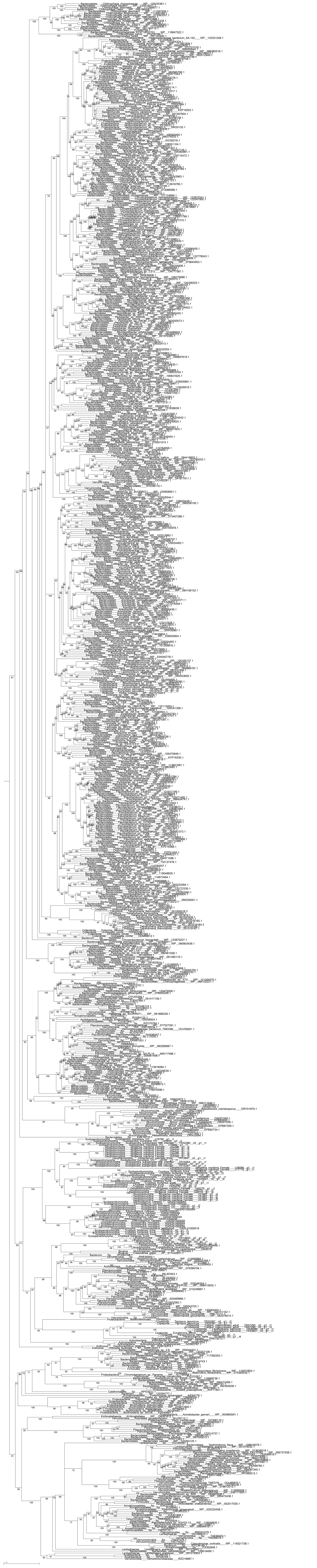

Supplementary Fig. 3. Maximum likelihood tree of PAD sequences. The tree was reconstructed using the WAG+G4 model and is displayed as midpoint rooted. Bootstrap support values are shown at each node. Nodes with support <50% are collapsed into polytomies.

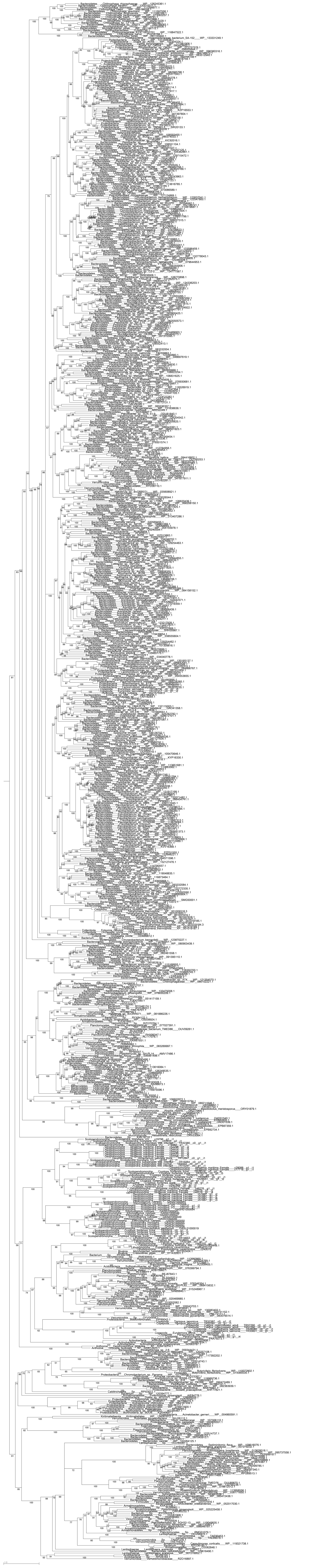

Supplementary Fig. 4. Maximum likelihood tree of DUF3472 sequences. The tree was reconstructed using the WAG+R10 model and is displayed as midpoint rooted. Bootstrap support values are shown at each node. Nodes with support <50% are collapsed into polytomies.

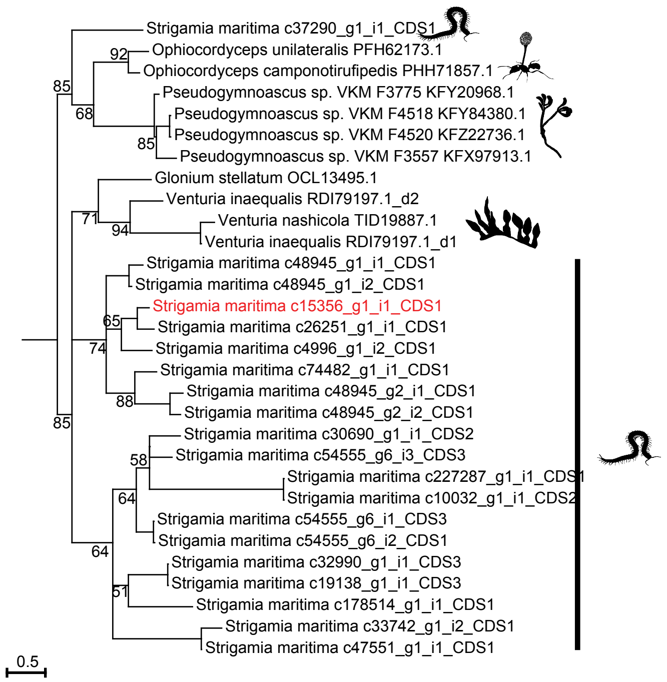

Strigamia maritima c37290_g1_i1_CDS1
Ophiocordyceps unilateralis PFH62173.1
Ophiocordyceps camponotirufipedis PHH71857.1
Pseudogymnoascus sp. VKM F3775 KFY20968.1
Pseudogymnoascus sp. VKM F4518 KFY84380.1
Pseudogymnoascus sp. VKM F4520 KFZ22736.1
Pseudogymnoascus sp. VKM F3557 KFX97913.1
Glonium stellatum OCL13495.1
Venturia inaequalis RDI79197.1_d2
Venturia nashicola TID19887.1
Venturia inaequalis RDI79197.1_d1
Strigamia maritima c48945_g1_i1_CDS1
Strigamia maritima c48945_g1_i2_CDS1
Strigamia maritima c15356_g1_i1_CDS1
Strigamia maritima c26251_g1_i1_CDS1
Strigamia maritima c4996_g1_i2_CDS1
Strigamia maritima c74482_g1_i1_CDS1
Strigamia maritima c48945_g2_i1_CDS1
Strigamia maritima c48945_g2_i2_CDS1
Strigamia maritima c30690_g1_i1_CDS2
Strigamia maritima c54555_g6_i3_CDS3
Strigamia maritima c227287_g1_i1_CDS1
Strigamia maritima c10032_g1_i1_CDS2
Strigamia maritima c54555_g6_i1_CDS3
Strigamia maritima c54555_g6_i2_CDS1
Strigamia maritima c32990_g1_i1_CDS3
Strigamia maritima c19138_g1_i1_CDS3
Strigamia maritima c178514_g1_i1_CDS1
Strigamia maritima c33742_g1_i2_CDS1
Strigamia maritima c47551_g1_i1_CDS1
0.5

Supplementary Fig. 5. Maximum likelihood tree of GEOTX02 sequences. The sequence in red is present in both the venom gland transcriptome and venom proteome. The tree was reconstructed using the VT+I+G4 model and is displayed as midpoint rooted. Bootstrap support values are shown at each node. Nodes with support <50% are collapsed into polytomies.

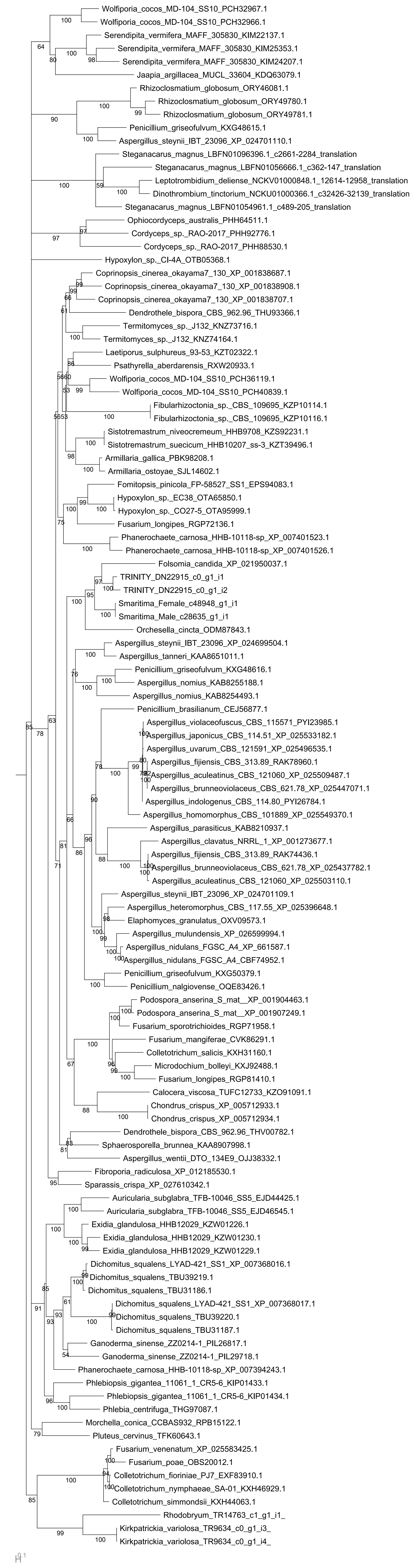
Wolfiporia_cocos_MD-104_SS10_PCH32967.1
Wolfiporia_cocos_MD-104_SS10_PCH32966.1
Serendipita_vermifera_MAFF_305830_KIM22137.1
Serendipita_vermifera_MAFF_305830_KIM25353.1
Serendipita_vermifera_MAFF_305830_KIM24207.1
Jaapia_argillacea_MUCL_33604_KDQ63079.1
Rhizoclosmatium_globosum_ORY46081.1
Rhizoclosmatium_globosum_ORY49780.1
Rhizoclosmatium_globosum_ORY49781.1
Penicillium_griseofulvum_KXG48615.1
Aspergillus_steynii_IBT_23096_XP_024701110.1
Steganacarus_magnus_LBFN01096396.1_c2661-2284_translation
Steganacarus_magnus_LBFN01056666.1_c362-147_translation
Leptotrombidium_deliense_NCKV01000848.1_12614-12958_translation
Dinothrombium_tinctorium_NCKU01000366.1_c32426-32139_translation
Steganacarus_magnus_LBFN01054961.1_c489-205_translation
Ophiocordyceps_australis_PHH64511.1
Cordyceps_sp._RAO-2017_PHH92776.1
Cordyceps_sp._RAO-2017_PHH88530.1
Hypoxylon_sp._CI-4A_OTB05368.1
Coprinopsis_cinerea_okayama7_130_XP_001838687.1
Coprinopsis_cinerea_okayama7_130_XP_001838908.1
Coprinopsis_cinerea_okayama7_130_XP_001838707.1
Dendrothele_bispora_CBS_962.96_THU93366.1
Termitomyces_sp._J132_KNZ73716.1
Termitomyces_sp._J132_KNZ74164.1
Laetiporus_sulphureus_93-53_KZT02322.1
Psathyrella_aberdarensis_RXW20933.1
Wolfiporia_cocos_MD-104_SS10_PCH36119.1
Wolfiporia_cocos_MD-104_SS10_PCH40839.1
Fibularhizoctonia_sp._CBS_109695_KZP10114.1
Fibularhizoctonia_sp._CBS_109695_KZP10116.1
Sistotremastrum_niveocremeum_HHB9708_KZS92231.1
Sistotremastrum_suecicum_HHB10207_ss-3_KZT39496.1
Armillaria_gallica_PBK98208.1
Armillaria_ostoyae_SJL14602.1
Fomitopsis_pinicola_FP-58527_SS1_EPS94083.1
Hypoxylon_sp._EC38_OTA65850.1
Hypoxylon_sp._CO27-5_OTA95999.1
Fusarium_longipes_RGP72136.1
Phanerochaete_carnosa_HHB-10118-sp_XP_007401523.1
Phanerochaete_carnosa_HHB-10118-sp_XP_007401526.1
Folsomia_candida_XP_021950037.1
TRINITY_DN22915_c0_g1_i1
TRINITY_DN22915_c0_g1_i2
Smaritima_Female_c48948_g1_i1
Smaritima_Male_c28635_g1_i1
Orchesella_cincta_ODM87843.1
Aspergillus_steynii_IBT_23096_XP_024699504.1
Aspergillus_tanneri_KAA8651011.1
Penicillium_griseofulvum_KXG48616.1
Aspergillus_nomius_KAB8255188.1
Aspergillus_nomius_KAB8254493.1
Penicillium_brasilianum_CEJ56877.1
Aspergillus_violaceofuscus_CBS_115571_PYI23985.1
Aspergillus_japonicus_CBS_114.51_XP_025533182.1
Aspergillus_uvarum_CBS_121591_XP_025496535.1
Aspergillus_fijiensis_CBS_313.89_RAK78960.1
Aspergillus_aculeatinus_CBS_121060_XP_025509487.1
Aspergillus_brunneoviolaceus_CBS_621.78_XP_025447071.1
Aspergillus_indologenus_CBS_114.80_PYI26784.1
Aspergillus_homomorphus_CBS_101889_XP_025549370.1
Aspergillus_parasiticus_KAB8210937.1
Aspergillus_clavatus_NRRL_1_XP_001273677.1
Aspergillus_fijiensis_CBS_313.89_RAK74436.1
Aspergillus_brunneoviolaceus_CBS_621.78_XP_025437782.1
Aspergillus_aculeatinus_CBS_121060_XP_025503110.1
Aspergillus_steynii_IBT_23096_XP_024701109.1
Aspergillus_heteromorphus_CBS_117.55_XP_025396648.1
Elaphomyces_granulatus_OXV09573.1
Aspergillus_mulundensis_XP_026599994.1
Aspergillus_nidulans_FGSC_A4_XP_661587.1
Aspergillus_nidulans_FGSC_A4_CBF74952.1
Penicillium_griseofulvum_KXG50379.1
Penicillium_nalgiovense_OQE83426.1
Podospora_anserina_S_mat__XP_001904463.1
Podospora_anserina_S_mat__XP_001907249.1
Fusarium_sporotrichioides_RGP71958.1
Fusarium_mangiferae_CVK86291.1
Colletotrichum_salicis_KXH31160.1
Microdochium_bolleyi_KXJ92488.1
Fusarium_longipes_RGP81410.1
Calocera_viscosa_TUFC12733_KZO91091.1
Chondrus_crispus_XP_005712933.1
Chondrus_crispus_XP_005712934.1
Dendrothele_bispora_CBS_962.96_THV00782.1
Sphaerosporella_brunnea_KAA8907998.1
Aspergillus_wentii_DTO_134E9_OJJ38332.1
Fibroporia_radiculosa_XP_012185530.1
Sparassis_crispa_XP_027610342.1
Auricularia_subglabra_TFB-10046_SS5_EJD44425.1
Auricularia_subglabra_TFB-10046_SS5_EJD46545.1
Exidia_glandulosa_HHB12029_KZW01226.1
Exidia_glandulosa_HHB12029_KZW01230.1
Exidia_glandulosa_HHB12029_KZW01229.1
Dichomitus_squalens_LYAD-421_SS1_XP_007368016.1
Dichomitus_squalens_TBU39219.1
Dichomitus_squalens_TBU31186.1
Dichomitus_squalens_LYAD-421_SS1_XP_007368017.1
Dichomitus_squalens_TBU39220.1
Dichomitus_squalens_TBU31187.1
Ganoderma_sinense_ZZ0214-1_PIL26817.1
Ganoderma_sinense_ZZ0214-1_PIL29718.1
Phanerochaete_carnosa_HHB-10118-sp_XP_007394243.1
Phlebiopsis_gigantea_11061_1_CR5-6_KIP01433.1
Phlebiopsis_gigantea_11061_1_CR5-6_KIP01434.1
Phlebia_centrifuga_THG97087.1
Morchella_conica_CCBAS932_RPB15122.1
Pluteus_cervinus_TFK60643.1
Fusarium_venenatum_XP_025583425.1
Fusarium_poae_OBS20012.1
Colletotrichum_fioriniae_PJ7_EXF83910.1
Colletotrichum_nymphaeae_SA-01_KXH46929.1
Colletotrichum_simmondsii_KXH44063.1
Rhodobryum_TR14763_c1_g1_i1_
Kirkpatrickia_variolosa_TR9634_c0_g1_i3_
Kirkpatrickia_variolosa_TR9634_c0_g1_i4_
0.1

Supplementary Fig. 6. Maximum likelihood tree of unchar05 sequences. The tree was reconstructed using the WAG+R5 model and is displayed as midpoint rooted. Bootstrap support values are shown at each node. Nodes with support <50% are collapsed into polytomies.

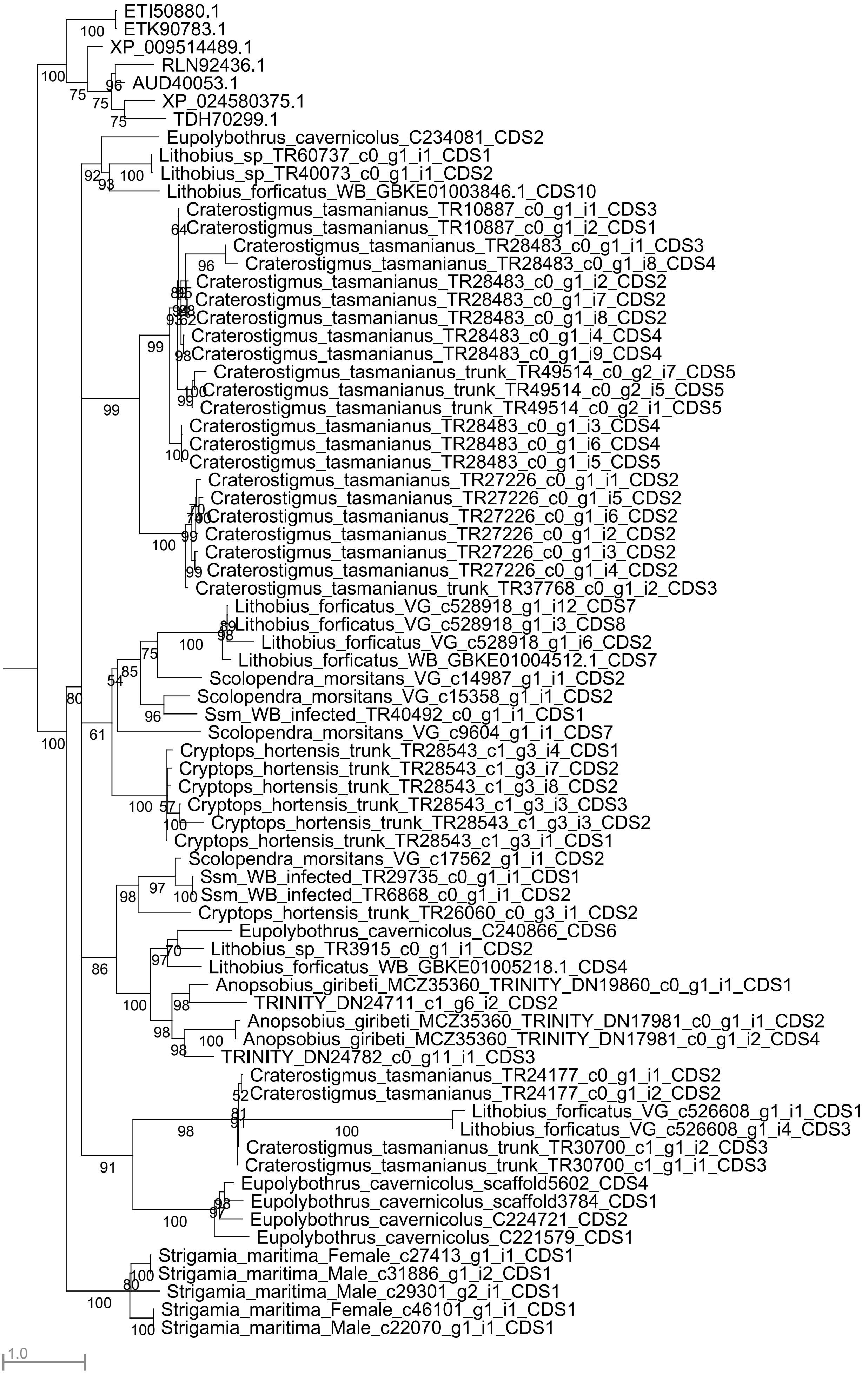
ETI50880.1
ETK90783.1
XP_009514489.1
RLN92436.1
AUD40053.1
XP_024580375.1
TDH70299.1
Eupolybothrus_cavernicolus_C234081_CDS2
Lithobius_sp_TR60737_c0_g1_i1_CDS1
Lithobius_sp_TR40073_c0_g1_i1_CDS2
Lithobius_forficatus_WB_GBKE01003846.1_CDS10
Craterostigmus_tasmanianus_TR10887_c0_g1_i1_CDS3
Craterostigmus_tasmanianus_TR10887_c0_g1_i2_CDS1
Craterostigmus_tasmanianus_TR28483_c0_g1_i1_CDS3
Craterostigmus_tasmanianus_TR28483_c0_g1_i8_CDS4
Craterostigmus_tasmanianus_TR28483_c0_g1_i2_CDS2
Craterostigmus_tasmanianus_TR28483_c0_g1_i7_CDS2
Craterostigmus_tasmanianus_TR28483_c0_g1_i8_CDS2
Craterostigmus_tasmanianus_TR28483_c0_g1_i4_CDS4
Craterostigmus_tasmanianus_TR28483_c0_g1_i9_CDS4
Craterostigmus_tasmanianus_trunk_TR49514_c0_g2_i7_CDS5
Craterostigmus_tasmanianus_trunk_TR49514_c0_g2_i5_CDS5
Craterostigmus_tasmanianus_trunk_TR49514_c0_g2_i1_CDS5
Craterostigmus_tasmanianus_TR28483_c0_g1_i3_CDS4
Craterostigmus_tasmanianus_TR28483_c0_g1_i6_CDS4
Craterostigmus_tasmanianus_TR28483_c0_g1_i5_CDS5
Craterostigmus_tasmanianus_TR27226_c0_g1_i1_CDS2
Craterostigmus_tasmanianus_TR27226_c0_g1_i5_CDS2
Craterostigmus_tasmanianus_TR27226_c0_g1_i6_CDS2
Craterostigmus_tasmanianus_TR27226_c0_g1_i2_CDS2
Craterostigmus_tasmanianus_TR27226_c0_g1_i3_CDS2
Craterostigmus_tasmanianus_TR27226_c0_g1_i4_CDS2
Craterostigmus_tasmanianus_trunk_TR37768_c0_g1_i2_CDS3
Lithobius_forficatus_VG_c528918_g1_i12_CDS7
Lithobius_forficatus_VG_c528918_g1_i3_CDS8
Lithobius_forficatus_VG_c528918_g1_i6_CDS2
Lithobius_forficatus_WB_GBKE01004512.1_CDS7
Scolopendra_morsitans_VG_c14987_g1_i1_CDS2
Scolopendra_morsitans_VG_c15358_g1_i1_CDS2
Ssm_WB_infected_TR40492_c0_g1_i1_CDS1
Scolopendra_morsitans_VG_c9604_g1_i1_CDS7
Cryptops_hortensis_trunk_TR28543_c1_g3_i4_CDS1
Cryptops_hortensis_trunk_TR28543_c1_g3_i7_CDS2
Cryptops_hortensis_trunk_TR28543_c1_g3_i8_CDS2
Cryptops_hortensis_trunk_TR28543_c1_g3_i3_CDS3
Cryptops_hortensis_trunk_TR28543_c1_g3_i3_CDS2
Cryptops_hortensis_trunk_TR28543_c1_g3_i1_CDS1
Scolopendra_morsitans_VG_c17562_g1_i1_CDS2
Ssm_WB_infected_TR29735_c0_g1_i1_CDS1
Ssm_WB_infected_TR6868_c0_g1_i1_CDS2
Cryptops_hortensis_trunk_TR26060_c0_g3_i1_CDS2
Eupolybothrus_cavernicolus_C240866_CDS6
Lithobius_sp_TR3915_c0_g1_i1_CDS2
Lithobius_forficatus_WB_GBKE01005218.1_CDS4
Anopsobius_giribeti_MCZ35360_TRINITY_DN19860_c0_g1_i1_CDS1
TRINITY_DN24711_c1_g6_i2_CDS2
Anopsobius_giribeti_MCZ35360_TRINITY_DN17981_c0_g1_i1_CDS2
Anopsobius_giribeti_MCZ35360_TRINITY_DN17981_c0_g1_i2_CDS4
TRINITY_DN24782_c0_g11_i1_CDS3
Craterostigmus_tasmanianus_TR24177_c0_g1_i1_CDS2
Craterostigmus_tasmanianus_TR24177_c0_g1_i2_CDS2
Lithobius_forficatus_VG_c526608_g1_i1_CDS1
Lithobius_forficatus_VG_c526608_g1_i4_CDS3
Craterostigmus_tasmanianus_trunk_TR30700_c1_g1_i2_CDS3
Craterostigmus_tasmanianus_trunk_TR30700_c1_g1_i1_CDS3
Eupolybothrus_cavernicolus_scaffold5602_CDS4
Eupolybothrus_cavernicolus_scaffold3784_CDS1
Eupolybothrus_cavernicolus_C224721_CDS2
Eupolybothrus_cavernicolus_C221579_CDS1
Strigamia_maritima_Female_c27413_g1_i1_CDS1
Strigamia_maritima_Male_c31886_g1_i2_CDS1
Strigamia_maritima_Male_c29301_g2_i1_CDS1
Strigamia_maritima_Female_c46101_g1_i1_CDS1
Strigamia_maritima_Male_c22070_g1_i1_CDS1
1.0

Supplementary Fig. 7. Maximum likelihood tree of unchar16 sequences. The tree was reconstructed using the VT+R3 model and is rooted with the oomycete sequences. Bootstrap support values are shown at each node. Nodes with support <50% are collapsed into polytomies.
