# Supplementary material for: Phylogenetic analyses suggest centipede venom arsenals were repeatedly stocked by horizontal gene transfer
Source: Nat Commun. 2021 Feb 5;12:818. doi: 10.1038/s41467-021-21093-8 (PMC7864903; doi:10.1038/s41467-021-21093-8)
Supplement: Supplementary file 3 — Reporting Summary [file 41467_2021_21093_MOESM3_ESM.pdf]

nature research

Corresponding author(s): *Double-blind peer review submissions: write DBPR and your manuscript number here instead of author names.*

Last updated by author(s): *YYYY-MM-DD*

Reporting Summary

Nature Research wishes to improve the reproducibility of the work that we publish. This form provides structure for consistency and transparency in reporting. For further information on Nature Research policies, see our Editorial Policies and the Editorial Policy Checklist.

Statistics

For all statistical analyses, confirm that the following items are present in the figure legend, table legend, main text, or Methods section.

n/a	Confirmed	
☐	☒	The exact sample size (*n*) for each experimental group/condition, given as a discrete number and unit of measurement
☒	☐	A statement on whether measurements were taken from distinct samples or whether the same sample was measured repeatedly
☒	☐	The statistical test(s) used AND whether they are one- or two-sided<br>*Only common tests should be described solely by name; describe more complex techniques in the Methods section.*
☒	☐	A description of all covariates tested
☒	☐	A description of any assumptions or corrections, such as tests of normality and adjustment for multiple comparisons
☒	☐	A full description of the statistical parameters including central tendency (e.g. means) or other basic estimates (e.g. regression coefficient) AND variation (e.g. standard deviation) or associated estimates of uncertainty (e.g. confidence intervals)
☒	☐	For null hypothesis testing, the test statistic (e.g. *F*, *t*, *r*) with confidence intervals, effect sizes, degrees of freedom and *P* value noted<br>*Give P values as exact values whenever suitable.*
☒	☐	For Bayesian analysis, information on the choice of priors and Markov chain Monte Carlo settings
☒	☐	For hierarchical and complex designs, identification of the appropriate level for tests and full reporting of outcomes
☒	☐	Estimates of effect sizes (e.g. Cohen's *d*, Pearson's *r*), indicating how they were calculated

Our web collection on statistics for biologists contains articles on many of the points above.

Software and code

Policy information about availability of computer code

Data collection	- BLAST+: used to search for genetic sequences in public databases (Camacho et al. 2009, BMC Bioinformatics 10: 421; https://blast.ncbi.nlm.nih.gov/Blast.cgi)<br>- HMMER v3.2.1: used to search for genetic sequences in public and custom databases (http://hmmer.org)
Data analysis	- Alienness version 1: used to identify putative HGT candidates (Rancurel et al. 2017, Genes 8: 248; http://alienness.sophia.inra.fr/cgi/index.cgi)<br>- Geneious v11.1.5: used to construct and annotate sequence alignments (https://www.geneious.com)<br>- MAFFT v7.304b and v7.450: used to produce sequence alignments (Katoh and Standley, 2013, Mol. Biol. Evol. 30: 772-780)<br>- MAFFT regional alignment ruby script: used to produce constrained sequence alignments (https://mafft.cbrc.jp/alignment/software/regionalrealignment.html)<br>- ModelFinder: used to determine optimal substitution model for phylogenetic analyses (Kalyaanamoorthy et al. 2017, Nature Methods 14: 587-589)<br>- IQ-TREE v1.5.5 and v2: used to construct phylogenetic trees and perform tree topology tests (Minh et al 2020, Mol. Biol. Evol. 37: 1530-1534)<br>- Archaeopteryx v0.9921: used to visualise phylogenetic trees (Han and Zmasek 2009, Bioinformatics 10: 356)<br>- Mesquite 3.61 (build 927): used to construct constraint tree topologies (http://www.mesquiteproject.org)<br>- CLC Main WorkBench v7: used to examine sequences and perform GC content calculations (Qiagen, Aarhus, Denmark)<br>- CD-HIT v4.6: used to filter sequences (Li and Godzik 2006, Bioinformatics 22: 1658-1659)<br>- GraphPad Prism v8.4.1: used to calculate descriptive statistics (http://www.graphpad.com)

For manuscripts utilizing custom algorithms or software that are central to the research but not yet described in published literature, software must be made available to editors and reviewers. We strongly encourage code deposition in a community repository (e.g. GitHub). See the Nature Research guidelines for submitting code & software for further information.

Data

Policy information about availability of data

All manuscripts must include a data availability statement. This statement should provide the following information, where applicable:

- Accession codes, unique identifiers, or web links for publicly available datasets
- A list of figures that have associated raw data
- A description of any restrictions on data availability

The transcriptomic custom database used in this study is available in the NIRD Research Data Archive with identifier 10.11582/2020.00067 [https://l.antigena.com/l/-XpFdcjUOuQ3kwVUGwNUCcawa65ouPHcGAU1UyZ4_G8tW7vXlL81qJ8DGsAVtkPIn4FKNoqN6enY799zIGURLtFK78EEeGN7Vjv6rkUj6QgiCaGMuFn2wNUwN3avmVFclTjxYAKWjK8PqF7hKgWurRu8L2F61L~640JO9Vwr1vwCQm]. The transcriptome data from Undheim et al. are available at the National Center for Biotechnology Information (NCBI) under bioprojects PRJNA200639 [https://www.ncbi.nlm.nih.gov/bioproject/PRJNA200639], PRJNA200641 [https://www.ncbi.nlm.nih.gov/bioproject/PRJNA200641], PRJNA200753 [https://www.ncbi.nlm.nih.gov/bioproject/PRJNA200753], PRJNA200640 [https://www.ncbi.nlm.nih.gov/bioproject/PRJNA200640], and PRJNA213032 [https://www.ncbi.nlm.nih.gov/bioproject/PRJNA213032], while individually curated sequences are available in the Transcriptome Shotgun Assembly Sequence Database (https://www.ncbi.nlm.nih.gov/nuccore/) as GASI01000001–GASI01000195, GASL01000001–GASL01000050, GASK01000001–GASK01000051, GASH01000001–GASH01000185, and GASR01000001–GASR01000119. Undheim et al.'s proteomic evidence are available as supplementary files associated with the original publication. The assembled transcriptomes from Jenner et al. are available via the Natural History Museum's Data Portal (https://data.nhm.ac.uk/dataset/evolution-of-centipede-venoms; last accessed 30 June 2020). 9), while the proteomic data are available in the ProteomeXchange Consortium via the PRIDE partner repository with the data set identifier PXD013356 [https://www.ebi.ac.uk/pride/archive/projects/PXD013356]. In addition we used the following databases: NCBI non-redundant (nr) database (https://www.ncbi.nlm.nih.gov), EnsemblMetazoa (https://metazoa.ensembl.org/index.html), and the databases in the InterPro Consortium (https://www.ebi.ac.uk/interpro/).

Field-specific reporting

Please select the one below that is the best fit for your research. If you are not sure, read the appropriate sections before making your selection.

☐ Life sciences ☐ Behavioural & social sciences ☒ Ecological, evolutionary & environmental sciences

For a reference copy of the document with all sections, see nature.com/documents/nr-reporting-summary-flat.pdf

Ecological, evolutionary & environmental sciences study design

All studies must disclose on these points even when the disclosure is negative.

Study description	The study reports phylogenetic analyses of previously published venom proteome-annotated transcriptome data from centipede venom glands, supplemented by mapping of sequence data to the genome of Strigamia maritima, and descriptive statistics of GC content of the sequence data.
Research sample	The primary data used in the paper encompassed proteomic and transcriptomic venom data for nine species of centipedes that were selected to represent all centipede orders.These data were previously published in Undheim et al. 2014, Mol. Biol. Evol. 31: 21-24-2148 and Jenner et al. 2019, Mol. Biol. Evol. 36: 2748-2763. The transcriptome data from Undheim et al. are available at the National Center for Biotechnology Information (NCBI) under bioprojects PRJNA200639 [https://www.ncbi.nlm.nih.gov/bioproject/PRJNA200639], PRJNA200641 [https://www.ncbi.nlm.nih.gov/bioproject/PRJNA200641], PRJNA200753 [https://www.ncbi.nlm.nih.gov/bioproject/PRJNA200753], PRJNA200640 [https://www.ncbi.nlm.nih.gov/bioproject/PRJNA200640], and PRJNA213032 [https://www.ncbi.nlm.nih.gov/bioproject/PRJNA213032], while individually curated sequences are available in the Transcriptome Shotgun Assembly Sequence Database (https://www.ncbi.nlm.nih.gov/nuccore/) as GASI01000001–GASI01000195, GASL01000001–GASL01000050, GASK01000001–GASK01000051, GASH01000001–GASH01000185, and GASR01000001–GASR01000119. Undheim et al.'s proteomic evidence are available as supplementary files associated with the original publication. The assembled transcriptomes from Jenner et al. are available via the Natural History Museum's Data Portal (https://data.nhm.ac.uk/dataset/evolution-of-centipede-venoms; last accessed 30 June 2020). 9), while the proteomic data are available in the ProteomeXchange Consortium via the PRIDE partner repository with the data set identifier PXD013356 [https://www.ebi.ac.uk/pride/archive/projects/PXD013356]. Our analyses further included sequences obtained from the following public sequence databases: NCBI non-redundant (nr) database (https://www.ncbi.nlm.nih.gov), EnsemblMetazoa (https://metazoa.ensembl.org/index.html), and the databases in the InterPro Consortium (https://www.ebi.ac.uk/interpro/). We also used a transcriptomic custom database that is available in the NIRD Research Data Archive with identifier 10.11582/2020.00067 [https://l.antigena.com/l/-XpFdcjUOuQ3kwVUGwNUCcawa65ouPHcGAU1UyZ4_G8tW7vXlL81qJ8DGsAVtkPIn4FKNoqN6enY799zIGURLtFK78EEeGN7Vjv6rkUj6QgiCaGMuFn2wNUwN3avmVFclTjxYAKWjK8PqF7hKgWurRu8L2F61L~640JO9Vwr1vwCQm].
Sampling strategy	The number of datasets reflects the number of species sampled in these two previous studies. These species were chosen to represent all major lineages of centipedes.
Data collection	The primary data were collected for two previous studies: Undheim et al. 2014, Mol. Biol. Evol. 31: 21-24-2148 and Jenner et al. 2019, Mol. Biol. Evol. 36: 2748-2763. Information about data collection is included in these papers.
Timing and spatial scale	See Undheim et al. 2014, Mol. Biol. Evol. 31: 21-24-2148 and Jenner et al. 2019, Mol. Biol. Evol. 36: 2748-2763.
Data exclusions	No data were excluded.
Reproducibility	The results can be reproduced by accessing and re-analysing all the data used in this study. All the relevant data are publicly available

as per the Data Availability section of the manuscript.

Randomization	The work done didn't involve any experiments or comparisons between experimental and control groups.
Blinding	The work done didn't involve any experiments or comparisons between experimental and control groups.

Did the study involve field work? ☐ Yes ☒ No

Reporting for specific materials, systems and methods

We require information from authors about some types of materials, experimental systems and methods used in many studies. Here, indicate whether each material, system or method listed is relevant to your study. If you are not sure if a list item applies to your research, read the appropriate section before selecting a response.

Materials & experimental systems

n/a	Involved in the study
☒	☐ Antibodies
☒	☐ Eukaryotic cell lines
☒	☐ Palaeontology and archaeology
☒	☐ Animals and other organisms
☒	☐ Human research participants
☒	☐ Clinical data
☒	☐ Dual use research of concern

Methods

n/a	Involved in the study
☒	☐ ChIP-seq
☒	☐ Flow cytometry
☒	☐ MRI-based neuroimaging
