# Supplementary material for: Phylogenetic analyses suggest centipede venom arsenals were repeatedly stocked by horizontal gene transfer
Source: Nat Commun. 2021 Feb 5;12:818. doi: 10.1038/s41467-021-21093-8 (PMC7864903; doi:10.1038/s41467-021-21093-8)
Supplement: Supplementary file 4 — Description of Additional Supplementary Files [file 41467_2021_21093_MOESM4_ESM.pdf]

Description of Additional Supplementary Files

Supplementary Data 1.

Results of the maximum likelihood ratio tree topology tests performed in IQ-TREE 2.

Supplementary Data 2.

Amino acid sequence alignment of Thereuopoda longicornis centiPAD sequences and their most closely related bacterial PAD sequences depicted in Fig. 3.

Supplementary Data 3.

GC content analysis of horizontally transferred centipede venom gene families.

Supplementary Data 4.

Amino acid sequence alignments of the horizontally transferred centipede venom gene families used to generate the trees in this paper.

Supplementary Data 5.

Amino acid sequence alignments used to train the HMMER profiles.

Supplementary Data 6.

HMMER profiles.

Supplementary Data 7.

Alienness results. Supplementary Data 8. Protein domain annotations for all gene family alignments.
