# Supplementary material for: Phylogenetic analyses suggest centipede venom arsenals were repeatedly stocked by horizontal gene transfer
Source: Nat Commun. 2021 Feb 5;12:818. doi: 10.1038/s41467-021-21093-8 (PMC7864903; doi:10.1038/s41467-021-21093-8)
Supplement: Supplementary file 11 — Supplementary Data 7 [file 41467_2021_21093_MOESM11_ESM.zip › centipad_index.html]

Index centipad


```
# Alienness results


Very likely HGT
Possible HGT
Likely contamination

  


| top Very likely HGT | | |
| --- | --- | --- |
| Lithobius_forficatus_VG_c543836_g1_i4_CDS6 | 126.67 | Bacteria |
| Lithobius_forficatus_VG_c543836_g1_i2_CDS7 | 79.07 | Bacteria |
| Lithobius_forficatus_VG_c543836_g1_i5_CDS3 | 77.83 | Bacteria |
| Lithobius_forficatus_VG_c543836_g1_i6_CDS9 | 54.09 | Bacteria |
| Lithobius_forficatus_VG_c543836_g1_i1_CDS6 | 50.43 | Bacteria |
| Lithobius_forficatus_VG_c543836_g1_i7_CDS1 | 37.21 | Bacteria |


| top Possible HGT | | |
| --- | --- | --- |
| Lithobius_forficatus_VG_c1536665_g1_i1_CDS1 | 12.60 | Bacteria |


| top Likely contamination | | |
| --- | --- | --- |
| Lithobius_sp_TR15234_c0_g1_i5_CDS2 | 120.57 | Bacteria |
| Lithobius_sp_TR15189_c1_g1_i2_CDS3 | 115.27 | Bacteria |
| Lithobius_sp_TR15189_c1_g1_i1_CDS3 | 95.51 | Bacteria |
| Thereuopoda_longicornis_GASR01000096 | 55.36 | Bacteria |
| Thereuopoda_longicornis_GASR01000097 | 54.52 | Bacteria |
| Thereuopoda_longicornis_GASR01000098 | 49.00 | Bacteria |
```
