# Supplementary material for: Phylogenetic analyses suggest centipede venom arsenals were repeatedly stocked by horizontal gene transfer
Source: Nat Commun. 2021 Feb 5;12:818. doi: 10.1038/s41467-021-21093-8 (PMC7864903; doi:10.1038/s41467-021-21093-8)
Supplement: Supplementary file 11 — Supplementary Data 7 [file 41467_2021_21093_MOESM11_ESM.zip › DUF3472_index.html]

Index DUF3472


```
# Alienness results


Very likely HGT
Possible HGT
Likely contamination

  


| top Very likely HGT | | |
| --- | --- | --- |
| Scolopendra_morsitans_GASH01000055 | 47.95 | Bacteria |
| Scolopendra_morsitans_GASH01000057 | 47.87 | Bacteria |
| Scolopendra_morsitans_GASH01000056 | 47.80 | Bacteria |
| Cryptops_hortensis_trunk_TR29376_c0_g5_i9_CDS3 | 47.04 | Bacteria |
| Scolopendra_morsitans_VG_c20737_g1_i1_CDS2 | 45.97 | Bacteria |
| Scolopendra_morsitans_GASH01000054 | 43.87 | Bacteria |
| Ethmostigmus_rubripes_GASI01000044 | 43.85 | Bacteria |
| Strigamia_maritima_Female_c51667_g1_i2_CDS1 | 40.12 | Bacteria |
| Strigamia_maritima_Female_c51667_g1_i3_CDS1 | 40.12 | Bacteria |
| Strigamia_maritima_Female_c51667_g1_i7_CDS1 | 40.12 | Bacteria |
| Strigamia_maritima_Female_c51667_g1_i6_CDS1 | 40.12 | Bacteria |
| Cryptops_hortensis_trunk_TR27822_c0_g1_i1_CDS2 | 36.63 | Bacteria |
| Cryptops_hortensis_trunk_TR10152_c0_g1_i1_CDS1 | 35.63 | Bacteria |
| Cryptops_hortensis_trunk_TR29376_c0_g5_i6_CDS3 | 34.27 | Bacteria |
| Scolopendra_morsitans_VG_c22453_g2_i1_CDS2 | 33.89 | Bacteria |


| top Possible HGT | | |
| --- | --- | --- |
| Scolopendra_morsitans_VG_c19796_g1_i1_CDS3 | 27.36 | Bacteria |
| Ethmostigmus_rubripes_GASI01000043 | 26.41 | Bacteria |
| Strigamia_maritima_Female_c52298_g1_i2_CDS3 | 26.24 | Bacteria |
| Strigamia_maritima_Female_c52298_g1_i3_CDS3 | 26.24 | Bacteria |
| Strigamia_maritima_Male_c42370_g1_i6_CDS8 | 26.24 | Bacteria |
| Strigamia_maritima_Male_c42370_g1_i1_CDS9 | 26.24 | Bacteria |
| Strigamia_maritima_Female_c52298_g1_i5_CDS3 | 26.24 | Bacteria |
| Strigamia_maritima_Male_c42370_g1_i5_CDS8 | 26.24 | Bacteria |
| Scolopendra_morsitans_VG_c9197_g1_i1_CDS2 | 25.94 | Bacteria |
| Cryptops_hortensis_trunk_TR29551_c0_g1_i1_CDS2 | 24.34 | Bacteria |
| Scolopendra_morsitans_VG_c18482_g1_i1_CDS3 | 23.25 | Bacteria |
| Strigamia_maritima_Female_c46276_g1_i2_CDS4 | 19.66 | Bacteria |
| Strigamia_maritima_Female_c46276_g1_i4_CDS3 | 19.66 | Bacteria |
| Ethmostigmus_rubripes_GASI01000046 | 18.48 | Bacteria |
| Strigamia_maritima_Female_c28088_g1_i1_CDS1 | 18.32 | Bacteria |
| Strigamia_maritima_Male_c17718_g1_i1_CDS1 | 18.32 | Bacteria |
| Strigamia_maritima_Female_c46276_g1_i1_CDS5 | 16.92 | Bacteria |
| Cryptops_hortensis_trunk_TR22915_c0_g1_i1_CDS1 | 16.35 | Bacteria |
| Scolopendra_subspinipes_WB_infected_TR15064_c0_g1_i1_CDS3 | 16.21 | Bacteria |
| Strigamia_maritima_Male_c42370_g1_i2_CDS1 | 16.18 | Bacteria |
| Strigamia_maritima_Male_c42370_g1_i4_CDS1 | 16.18 | Bacteria |
| Strigamia_maritima_Male_c42370_g1_i5_CDS1 | 16.18 | Bacteria |
| Strigamia_maritima_Male_c42370_g1_i3_CDS1 | 16.18 | Bacteria |
| Strigamia_maritima_Male_c42370_g1_i6_CDS1 | 16.18 | Bacteria |
| Strigamia_maritima_Female_c46276_g1_i4_CDS1 | 14.38 | Bacteria |
| Ethmostigmus_rubripes_GASI01000045 | 13.37 | Bacteria |
| Lithobius_forficatus_VG_c542310_g2_i2_CDS4 | 13.36 | Bacteria |
| Ethmostigmus_rubripes_GASI01000049 | 9.51 | Bacteria |
| Himantarium_gabrielis_trunk_TR12847_c0_g1_i1_CDS1 | 7.42 | Bacteria |
| Cormocephalus_westwoodi_GASL01000019 | 5.90 | Bacteria |
| Ethmostigmus_rubripes_GASI01000047 | 5.27 | Bacteria |
| Lithobius_sp_TR20185_c0_g1_i1_CDS2 | 3.32 | Bacteria |
| Ethmostigmus_rubripes_GASI01000048 | 0.70 | Bacteria |
| Ethmostigmus_rubripes_GASI01000050 | 0.67 | Bacteria |
```
