# Supplementary material for: Phylogenetic analyses suggest centipede venom arsenals were repeatedly stocked by horizontal gene transfer
Source: Nat Commun. 2021 Feb 5;12:818. doi: 10.1038/s41467-021-21093-8 (PMC7864903; doi:10.1038/s41467-021-21093-8)
Supplement: Supplementary file 11 — Supplementary Data 7 [file 41467_2021_21093_MOESM11_ESM.zip › geotx02_index.html]

Index geotx02


```
# Alienness results


Very likely HGT
Possible HGT
Likely contamination

  


| top Very likely HGT | | |
| --- | --- | --- |


| top Possible HGT | | |
| --- | --- | --- |
| Smaritima_Female_c48945_g1_i1_CDS1 | 7.75 | Eukaryota |
| Smaritima_Female_c48945_g1_i2_CDS1 | 7.04 | Eukaryota |
| Smaritima_Female_c37290_g1_i1_CDS1 | 4.59 | Eukaryota |
| Smaritima_Female_c37290_g1_i2_CDS1 | 4.59 | Eukaryota |
| Smaritima_Male_c30235_g1_i1_CDS2 | 4.59 | Eukaryota |
| Smaritima_Male_c30235_g1_i2_CDS2 | 4.59 | Eukaryota |
| Smaritima_Male_c29073_g1_i1_CDS1 | 3.65 | Eukaryota |
| Smaritima_Female_c48945_g2_i2_CDS1 | 3.43 | Eukaryota |
| Smaritima_Male_c36461_g1_i2_CDS2 | 3.43 | Eukaryota |
| Smaritima_Male_c31938_g1_i1_CDS1 | 2.47 | Eukaryota |
| Smaritima_Male_c4996_g1_i2_CDS1 | 1.70 | Eukaryota |
| Smaritima_Female_c32990_g1_i1_CDS3 | 1.50 | Eukaryota |
| Smaritima_Male_c19138_g1_i1_CDS3 | 1.50 | Eukaryota |
| Smaritima_Female_c54555_g6_i3_CDS3 | 1.15 | Bacteria |
| Smaritima_Female_c54555_g6_i4_CDS1 | 1.15 | Bacteria |
| Smaritima_Female_c54555_g6_i5_CDS3 | 1.15 | Bacteria |
| Smaritima_Female_c54555_g6_i6_CDS1 | 1.15 | Bacteria |
| Smaritima_Male_c37630_g1_i2_CDS2 | 1.15 | Bacteria |
| Smaritima_Male_c37630_g1_i3_CDS2 | 1.15 | Bacteria |
| Smaritima_Female_c54555_g6_i2_CDS1 | 1.03 | Eukaryota |
| Smaritima_Male_c37630_g1_i1_CDS3 | 1.03 | Eukaryota |
| Smaritima_Female_c54555_g6_i1_CDS3 | 1.02 | Eukaryota |
```
