# Supplementary material for: Phylogenetic analyses suggest centipede venom arsenals were repeatedly stocked by horizontal gene transfer
Source: Nat Commun. 2021 Feb 5;12:818. doi: 10.1038/s41467-021-21093-8 (PMC7864903; doi:10.1038/s41467-021-21093-8)
Supplement: Supplementary file 11 — Supplementary Data 7 [file 41467_2021_21093_MOESM11_ESM.zip › unchar16_index.html]

Index unchar16


```
# Alienness results


Very likely HGT
Possible HGT
Likely contamination

  


| top Very likely HGT | | |
| --- | --- | --- |


| top Possible HGT | | |
| --- | --- | --- |
| Strigamia_maritima_Male_c29301_g2_i1_CDS1 | 10.44 | Eukaryota |
| Craterostigmus_tasmanianus_TR28483_c0_g1_i1_CDS3 | 1.32 | Eukaryota |
| Craterostigmus_tasmanianus_TR28483_c0_g1_i7_CDS2 | 0.81 | Eukaryota |
| Scolopendra_morsitans_VG_c14987_g1_i1_CDS2 | 0.50 | Eukaryota |
| Craterostigmus_tasmanianus_TR28483_c0_g1_i2_CDS2 | 0.17 | Eukaryota |
```
