# Supplementary material for: Phylogenetic analyses suggest centipede venom arsenals were repeatedly stocked by horizontal gene transfer
Source: Nat Commun. 2021 Feb 5;12:818. doi: 10.1038/s41467-021-21093-8 (PMC7864903; doi:10.1038/s41467-021-21093-8)
Supplement: Supplementary file 11 — Supplementary Data 7 [file 41467_2021_21093_MOESM11_ESM.zip › slptx02_index.html]

Index slptx02


```
# Alienness results


Very likely HGT
Possible HGT
Likely contamination

  


| top Very likely HGT | | |
| --- | --- | --- |


| top Possible HGT | | |
| --- | --- | --- |
| Lithobius_sp_TR12495_c0_g1_i1_CDS4-D1 | 14.21 | Eukaryota |
| Lithobius_sp_TR12519_c0_g1_i2_CDS3-D1 | 14.12 | Eukaryota |
| Lithobius_sp_TR14096_c0_g1_i1_CDS2-D1 | 14.12 | Eukaryota |
| Lithobius_sp_TR12519_c0_g1_i4_CDS3-D1 | 11.91 | Eukaryota |
| Lithobius_sp_TR10019_c0_g1_i1_CDS1 | 9.76 | Eukaryota |
| Lithobius_forficatus_VG_c405449_g1_i1_CDS1 | 9.07 | Eukaryota |
| Lithobius_forficatus_VG_c513196_g1_i2_CDS2 | 2.12 | Eukaryota |
```
