# Supplementary material for: Phylogenetic analyses suggest centipede venom arsenals were repeatedly stocked by horizontal gene transfer
Source: Nat Commun. 2021 Feb 5;12:818. doi: 10.1038/s41467-021-21093-8 (PMC7864903; doi:10.1038/s41467-021-21093-8)
Supplement: Supplementary file 11 — Supplementary Data 7 [file 41467_2021_21093_MOESM11_ESM.zip › BPFTx_index.html]

Index BPFTx


```
# Alienness results


Very likely HGT
Possible HGT
Likely contamination

  


| top Very likely HGT | | |
| --- | --- | --- |
| XP_013788657.1 | 42.14 | Bacteria |
| Scoleoptrata_VG_c1116731_g1_i1__CDS2  length 171 aa, 513 bp, from 40..552 of VG_Scu_c1116731_g1_i1 | 40.92 | Bacteria |
| SRA_Acuclavella_merickeli_TR1492|c0_g2_i1__CDS1 | 38.32 | Bacteria |
| SRA_Acuclavella_merickeli_TR1492|c0_g1_i1__CDS1 | 38.30 | Bacteria |
| Smaritima_Male_c3755_g1_i1 | 37.21 | Bacteria |
| XP_013788656.1 | 36.03 | Bacteria |
| Strigamia_T1_TR-04 | 35.23 | Bacteria |
| Smaritima_Female_c49320_g1_i4 | 34.96 | Bacteria |
| Smaritima_Female_c49320_g1_i6__CDS2  length 286 aa, 861 bp, from 115..975 of Female_c49320_g1_i6 len=1037 path=[1:0-179 181:180-180 @412@!:181-510 @556@!:511-660 2857:661-663 1116:664-1036] | 34.43 | Bacteria |
| Smaritima_Female_c49320_g1_i3__CDS3  length 151 aa, 456 bp, from 40..495 of Female_c49320_g1_i3 len=557 path=[519:0-14 534:15-30 @556@!:31-180 2857:181-183 1116:184-556] | 33.88 | Bacteria |
| SRA_Cryptocellus_becki_TR60696|c0_g3_i3__CDS4 | 33.64 | Bacteria |


| top Possible HGT | | |
| --- | --- | --- |
| SRA_Cryptops_hortensis_trunk_TR26263|c0_g2_i1__CDS2 | 29.86 | Bacteria |
| SRA_Cryptops_hortensis_trunk_TR70476|c0_g1_i1__CDS2 | 26.32 | Bacteria |
| Strigamia_T1_T03 | 23.94 | Bacteria |
| XP_002405816.1 | 22.99 | Bacteria |
| Smaritima_Female_c6740_g1_i1__CDS1  length 95 aa, 288 bp, from complement(88..375) of Female_c6740_g1_i1 len=394 path=[99:0-55 25:56-79 49:80-393] | 22.94 | Bacteria |
| SRA_Damon_variegatus_TR27914|c0_g1_i1__CDS1 | 22.76 | Bacteria |
| Lforficatus_VG_c513369_g1_i1__CDS3  length 105 aa, 318 bp, from complement(133..450) of VG_Lit_c513369_g1_i1 | 21.32 | Bacteria |
| Smaritima_Male_c193596_g1_i1__CDS2  length 95 aa, 288 bp, from 20..307 of Male_c193596_g1_i1 len=392 path=[1:0-391] | 20.99 | Bacteria |
| Smaritima_Female_c49320_g1_i2__CDS2  length 217 aa, 654 bp, from 115..768 of Female_c49320_g1_i2 len=1033 path=[1:0-179 181:180-180 @412@!:181-510 940:511-521 534:522-537 @556@!:538-687 2857:688-690 2447:691-775 458:776-1032] | 20.20 | Bacteria |
| SRA_Lithobius_sp_TR13835|c0_g1_i1__CDS1 | 16.50 | Bacteria |
| Scoleoptrata_VG_c657933_g1_i1__CDS2  length 165 aa, 498 bp, from complement(210..707) of VG_Scu_c657933_g1_i1 | 12.76 | Bacteria |
| Cormocephalus_westwoodi_GASL01000012 | 10.44 | Bacteria |
| Cormocephalus_westwoodi_GASL01000011 | 9.73 | Bacteria |
| Scolopendra_morsitans_GASH01000018 | 9.57 | Bacteria |
| Ethmostigmus_rubripes_GASI01000029 | 8.41 | Bacteria |
| Scolopendra_morsitans_GASH01000028 | 8.00 | Bacteria |
| Scolopendra_subspinipes_GGDW01000025.1 | 7.58 | Bacteria |
| Cormocephalus_westwoodi_GASL01000008 | 7.51 | Bacteria |
| Scolopendra_alternans_GASL01000007 | 7.49 | Bacteria |
| Scolopendra_subspinipes_GGDW01000007.1 | 7.38 | Bacteria |
| Scolopendra_morsitans_GASH01000019 | 7.36 | Bacteria |
| Cormocephalus_westwoodi_GASL01000003 | 7.27 | Bacteria |
| Ethmostigmus_rubripes_GASI01000012 | 7.15 | Bacteria |
| Scolopendra_subspinipes_GGDW01000011.1 | 7.06 | Bacteria |
| Scolopendra_morsitans_GASH01000020 | 7.05 | Bacteria |
| Scolopendra_subspinipes_GGDW01000012.1 | 6.91 | Bacteria |
| Smorsitans_VG_c23283_g1_i1__CDS3  length 337 aa, 1014 bp, from 255..1268 of VG_Sm_c23283_g1_i1 | 6.91 | Bacteria |
| Smorsitans_VG_c12633_g1_i1__CDS2  length 321 aa, 966 bp, from complement(86..1051) of VG_Sm_c12633_g1_i1 | 5.95 | Bacteria |
| Scoleoptrata_VG_c744736_g1_i2 | 4.30 | Bacteria |
| Lforficatus_VG_c544751_g1_i2__CDS2  length 316 aa, 951 bp, from 5..955 of VG_Lit_c544751_g1_i2 | 4.06 | Bacteria |
| Smaritima_Female_c2465_g1_i1__CDS1  length 158 aa, 477 bp, from complement(84..560) of Female_c2465_g1_i1 len=613 path=[944:0-365 273:366-612] | 0.15 | Bacteria |


| top Likely contamination | | |
| --- | --- | --- |
| Thereuopoda_longicornis_GASR01000022 | 8.18 | Bacteria |
```
