# Supplementary material for: Phylogenetic analyses suggest centipede venom arsenals were repeatedly stocked by horizontal gene transfer
Source: Nat Commun. 2021 Feb 5;12:818. doi: 10.1038/s41467-021-21093-8 (PMC7864903; doi:10.1038/s41467-021-21093-8)
Supplement: Supplementary file 11 — Supplementary Data 7 [file 41467_2021_21093_MOESM11_ESM.zip › unchar05_index.html]

Index unchar05


```
# Alienness results


Very likely HGT
Possible HGT
Likely contamination

  


| top Very likely HGT | | |
| --- | --- | --- |


| top Possible HGT | | |
| --- | --- | --- |
| Smaritima_Female_c28610_g1_i1|CDS1 | 1.87 | Eukaryota |
```
