# Supplementary material for: Phylogenetic analyses suggest centipede venom arsenals were repeatedly stocked by horizontal gene transfer
Source: Nat Commun. 2021 Feb 5;12:818. doi: 10.1038/s41467-021-21093-8 (PMC7864903; doi:10.1038/s41467-021-21093-8)
Supplement: Supplementary file 12 — Supplementary Data 8 [file 41467_2021_21093_MOESM12_ESM.zip › Supplementary Data 8/centiPAD extraction.pdf]

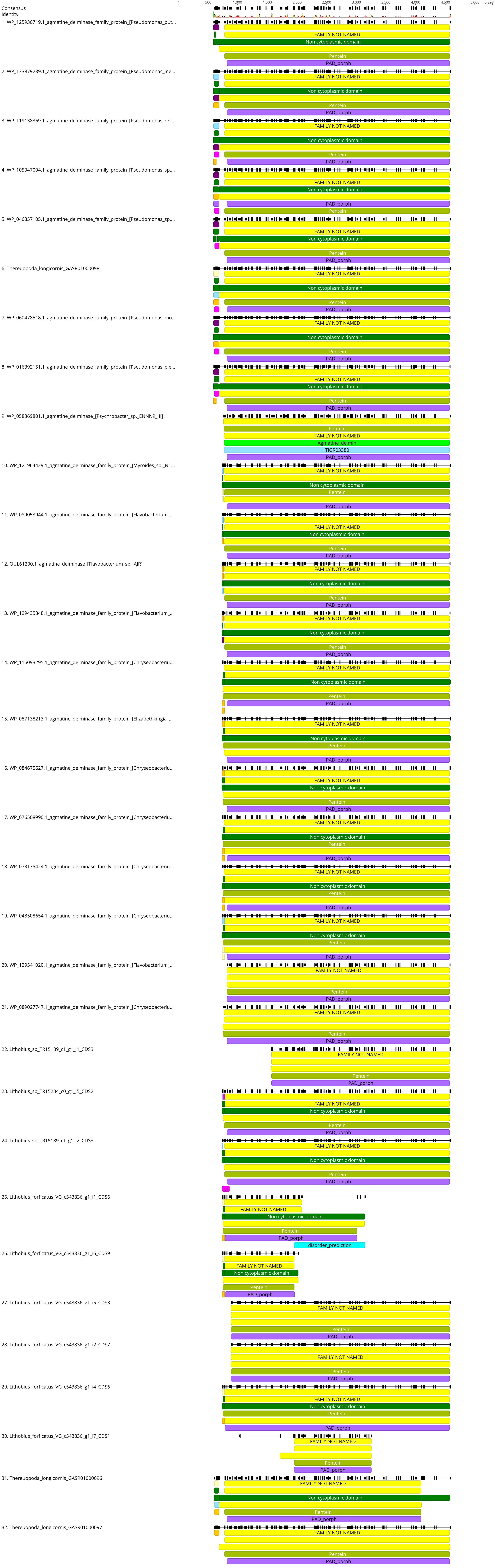
1
500
1,000
1,500
2,000
2,500
3,000
3,500
4,000
4,500
5,000
5,298
Consensus
Identity
1. WP_125930719.1_agmatine_deiminase_family_protein_[Pseudomonas_put...
FAMILY NOT NAMED
Non cytoplasmic domain
Pentein
PAD_porph
2. WP_133979289.1_agmatine_deiminase_family_protein_[Pseudomonas_ine...
FAMILY NOT NAMED
Non cytoplasmic domain
Pentein
PAD_porph
3. WP_119138369.1_agmatine_deiminase_family_protein_[Pseudomonas_rei...
FAMILY NOT NAMED
Non cytoplasmic domain
Pentein
PAD_porph
4. WP_105947004.1_agmatine_deiminase_family_protein_[Pseudomonas_sp....
FAMILY NOT NAMED
Non cytoplasmic domain
PAD_porph
Pentein
5. WP_046857105.1_agmatine_deiminase_family_protein_[Pseudomonas_sp....
FAMILY NOT NAMED
Non cytoplasmic domain
Pentein
PAD_porph
6. Thereuopoda_longicornis_GASR01000098
FAMILY NOT NAMED
Non cytoplasmic domain
Pentein
PAD_porph
7. WP_060478518.1_agmatine_deiminase_family_protein_[Pseudomonas_mo...
FAMILY NOT NAMED
Non cytoplasmic domain
Pentein
PAD_porph
8. WP_016392151.1_agmatine_deiminase_family_protein_[Pseudomonas_ple...
FAMILY NOT NAMED
Non cytoplasmic domain
Pentein
PAD_porph
9. WP_058369801.1_agmatine_deiminase_[Psychrobacter_sp._ENNN9_III]
Pentein
FAMILY NOT NAMED
Agmatine_deimin
TIGR03380
PAD_porph
10. WP_121964429.1_agmatine_deiminase_family_protein_[Myroides_sp._N1...
FAMILY NOT NAMED
Non cytoplasmic domain
Pentein
PAD_porph
11. WP_089053944.1_agmatine_deiminase_family_protein_[Flavobacterium_...
FAMILY NOT NAMED
Non cytoplasmic domain
Pentein
PAD_porph
12. OUL61200.1_agmatine_deiminase_[Flavobacterium_sp._AJR]
FAMILY NOT NAMED
Non cytoplasmic domain
Pentein
PAD_porph
13. WP_129435848.1_agmatine_deiminase_family_protein_[Flavobacterium_...
FAMILY NOT NAMED
Non cytoplasmic domain
Pentein
PAD_porph
14. WP_116093295.1_agmatine_deiminase_family_protein_[Chryseobacteriu...
FAMILY NOT NAMED
Non cytoplasmic domain
Pentein
PAD_porph
15. WP_087138213.1_agmatine_deiminase_family_protein_[Elizabethkingia_...
FAMILY NOT NAMED
Non cytoplasmic domain
Pentein
PAD_porph
16. WP_084675627.1_agmatine_deiminase_family_protein_[Chryseobacteriu...
FAMILY NOT NAMED
Non cytoplasmic domain
Pentein
PAD_porph
17. WP_076508990.1_agmatine_deiminase_family_protein_[Chryseobacteriu...
FAMILY NOT NAMED
Non cytoplasmic domain
Pentein
PAD_porph
18. WP_073175424.1_agmatine_deiminase_family_protein_[Chryseobacteriu...
FAMILY NOT NAMED
Non cytoplasmic domain
Pentein
PAD_porph
19. WP_048508654.1_agmatine_deiminase_family_protein_[Chryseobacteriu...
FAMILY NOT NAMED
Non cytoplasmic domain
Pentein
PAD_porph
20. WP_129541020.1_agmatine_deiminase_family_protein_[Flavobacterium_...
FAMILY NOT NAMED
Pentein
PAD_porph
21. WP_089027747.1_agmatine_deiminase_family_protein_[Chryseobacteriu...
FAMILY NOT NAMED
Pentein
PAD_porph
22. Lithobius_sp_TR15189_c1_g1_i1_CDS3
FAMILY NOT NAMED
Pentein
PAD_porph
23. Lithobius_sp_TR15234_c0_g1_i5_CDS2
FAMILY NOT NAMED
Non cytoplasmic domain
Pentein
PAD_porph
24. Lithobius_sp_TR15189_c1_g1_i2_CDS3
FAMILY NOT NAMED
Non cytoplasmic domain
Pentein
PAD_porph
25. Lithobius_forficatus_VG_c543836_g1_i1_CDS6
FAMILY NOT NAMED
Non cytoplasmic domain
Pentein
PAD_porph
disorder_prediction
26. Lithobius_forficatus_VG_c543836_g1_i6_CDS9
FAMILY NOT NAMED
Non cytoplasmic domain
Pentein
PAD_porph
27. Lithobius_forficatus_VG_c543836_g1_i5_CDS3
FAMILY NOT NAMED
Pentein
PAD_porph
28. Lithobius_forficatus_VG_c543836_g1_i2_CDS7
FAMILY NOT NAMED
Pentein
PAD_porph
29. Lithobius_forficatus_VG_c543836_g1_i4_CDS6
FAMILY NOT NAMED
Non cytoplasmic domain
Pentein
PAD_porph
30. Lithobius_forficatus_VG_c543836_g1_i7_CDS1
FAMILY NOT NAMED
Pentein
PAD_porph
31. Thereuopoda_longicornis_GASR01000096
FAMILY NOT NAMED
Non cytoplasmic domain
Pentein
PAD_porph
32. Thereuopoda_longicornis_GASR01000097
FAMILY NOT NAMED
Pentein
PAD_porph
