# Supplementary material for: Phylogenetic analyses suggest centipede venom arsenals were repeatedly stocked by horizontal gene transfer
Source: Nat Commun. 2021 Feb 5;12:818. doi: 10.1038/s41467-021-21093-8 (PMC7864903; doi:10.1038/s41467-021-21093-8)
Supplement: Supplementary file 12 — Supplementary Data 8 [file PCPDP.pdf › Supplementary Data 8/PCPDP.pdf]

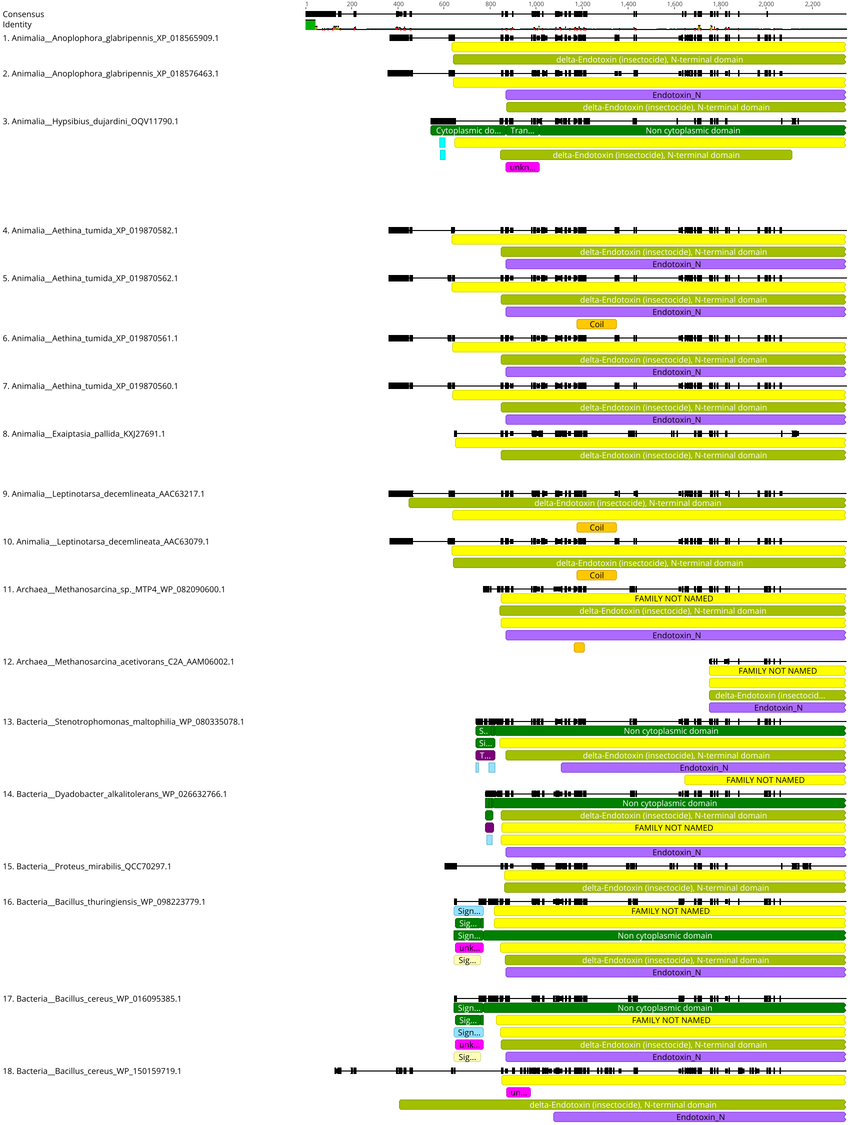
Consensus
Identity
1
200
400
600
800
1,000
1,200
1,400
1,600
1,800
2,000
2,200
1. Animalia__Anoplophora_glabripennis_XP_018565909.1
delta-Endotoxin (insectocide), N-terminal domain
2. Animalia__Anoplophora_glabripennis_XP_018576463.1
Endotoxin_N
delta-Endotoxin (insectocide), N-terminal domain
3. Animalia__Hypsibius_dujardini_OQV11790.1
Cytoplasmic do...
Tran...
Non cytoplasmic domain
delta-Endotoxin (insectocide), N-terminal domain
unkn...
4. Animalia__Aethina_tumida_XP_019870582.1
delta-Endotoxin (insectocide), N-terminal domain
Endotoxin_N
5. Animalia__Aethina_tumida_XP_019870562.1
delta-Endotoxin (insectocide), N-terminal domain
Endotoxin_N
Coil
6. Animalia__Aethina_tumida_XP_019870561.1
delta-Endotoxin (insectocide), N-terminal domain
Endotoxin_N
7. Animalia__Aethina_tumida_XP_019870560.1
delta-Endotoxin (insectocide), N-terminal domain
Endotoxin_N
8. Animalia__Exaiptasia_pallida_KXJ27691.1
delta-Endotoxin (insectocide), N-terminal domain
9. Animalia__Leptinotarsa_decemlineata_AAC63217.1
delta-Endotoxin (insectocide), N-terminal domain
Coil
10. Animalia__Leptinotarsa_decemlineata_AAC63079.1
delta-Endotoxin (insectocide), N-terminal domain
Coil
11. Archaea__Methanosarcina_sp._MTP4_WP_082090600.1
FAMILY NOT NAMED
delta-Endotoxin (insectocide), N-terminal domain
Endotoxin_N
12. Archaea__Methanosarcina_acetivorans_C2A_AAM06002.1
FAMILY NOT NAMED
delta-Endotoxin (insectocid...
Endotoxin_N
13. Bacteria__Stenotrophomonas_maltophilia_WP_080335078.1
S...
Non cytoplasmic domain
Si...
T...
delta-Endotoxin (insectocide), N-terminal domain
Endotoxin_N
FAMILY NOT NAMED
14. Bacteria__Dyadobacter_alkalitolerans_WP_026632766.1
Non cytoplasmic domain
delta-Endotoxin (insectocide), N-terminal domain
FAMILY NOT NAMED
Endotoxin_N
15. Bacteria__Proteus_mirabilis_QCC70297.1
delta-Endotoxin (insectocide), N-terminal domain
16. Bacteria__Bacillus_thuringiensis_WP_098223779.1
Sign...
FAMILY NOT NAMED
Sig...
Sign...
Non cytoplasmic domain
unk...
Sig...
delta-Endotoxin (insectocide), N-terminal domain
Endotoxin_N
17. Bacteria__Bacillus_cereus_WP_016095385.1
Sign...
Non cytoplasmic domain
Sig...
FAMILY NOT NAMED
Sign...
unk...
delta-Endotoxin (insectocide), N-terminal domain
Sig...
Endotoxin_N
18. Bacteria__Bacillus_cereus_WP_150159719.1
un...
delta-Endotoxin (insectocide), N-terminal domain
Endotoxin_N

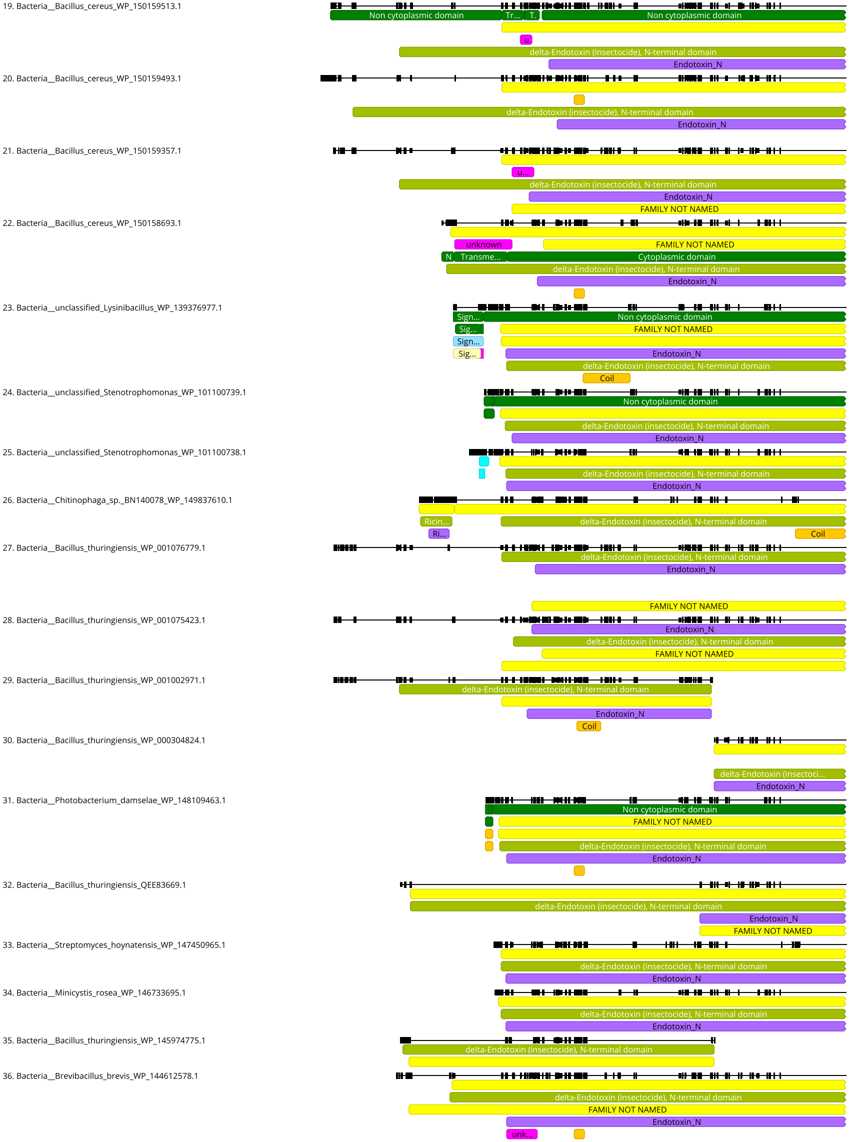
19. Bacteria__Bacillus_cereus_WP_150159513.1
Non cytoplasmic domain
Tr...
T.
Non cytoplasmic domain
u
delta-Endotoxin (insecticide), N-terminal domain
Endotoxin_N
20. Bacteria__Bacillus_cereus_WP_150159493.1
delta-Endotoxin (insecticide), N-terminal domain
Endotoxin_N
21. Bacteria__Bacillus_cereus_WP_150159357.1
u...
delta-Endotoxin (insecticide), N-terminal domain
Endotoxin_N
FAMILY NOT NAMED
22. Bacteria__Bacillus_cereus_WP_150158693.1
unknown
FAMILY NOT NAMED
N
Transme...
Cytoplasmic domain
delta-Endotoxin (insecticide), N-terminal domain
Endotoxin_N
23. Bacteria__unclassified_Lysinibacillus_WP_139376977.1
Sign...
Non cytoplasmic domain
Sig...
FAMILY NOT NAMED
Sign...
Sig...
Endotoxin_N
delta-Endotoxin (insecticide), N-terminal domain
Coil
24. Bacteria__unclassified_Stenotrophomonas_WP_101100739.1
Non cytoplasmic domain
delta-Endotoxin (insecticide), N-terminal domain
Endotoxin_N
25. Bacteria__unclassified_Stenotrophomonas_WP_101100738.1
delta-Endotoxin (insecticide), N-terminal domain
Endotoxin_N
26. Bacteria__Chitinophaga_sp._BN140078_WP_149837610.1
Ricin...
delta-Endotoxin (insecticide), N-terminal domain
Ri...
Coil
27. Bacteria__Bacillus_thuringiensis_WP_001076779.1
delta-Endotoxin (insecticide), N-terminal domain
Endotoxin_N
FAMILY NOT NAMED
28. Bacteria__Bacillus_thuringiensis_WP_001075423.1
Endotoxin_N
delta-Endotoxin (insecticide), N-terminal domain
FAMILY NOT NAMED
29. Bacteria__Bacillus_thuringiensis_WP_001002971.1
delta-Endotoxin (insecticide), N-terminal domain
Endotoxin_N
Coil
30. Bacteria__Bacillus_thuringiensis_WP_000304824.1
delta-Endotoxin (insectoci...
Endotoxin_N
31. Bacteria__Photobacterium_damselae_WP_148109463.1
Non cytoplasmic domain
FAMILY NOT NAMED
delta-Endotoxin (insecticide), N-terminal domain
Endotoxin_N
32. Bacteria__Bacillus_thuringiensis_QEE83669.1
delta-Endotoxin (insecticide), N-terminal domain
Endotoxin_N
FAMILY NOT NAMED
33. Bacteria__Streptomyces_hoynatensis_WP_147450965.1
delta-Endotoxin (insecticide), N-terminal domain
Endotoxin_N
34. Bacteria__Minicystis_rosea_WP_146733695.1
delta-Endotoxin (insecticide), N-terminal domain
Endotoxin_N
35. Bacteria__Bacillus_thuringiensis_WP_145974775.1
delta-Endotoxin (insecticide), N-terminal domain
36. Bacteria__Brevibacillus_brevis_WP_144612578.1
delta-Endotoxin (insecticide), N-terminal domain
FAMILY NOT NAMED
Endotoxin_N
unk...

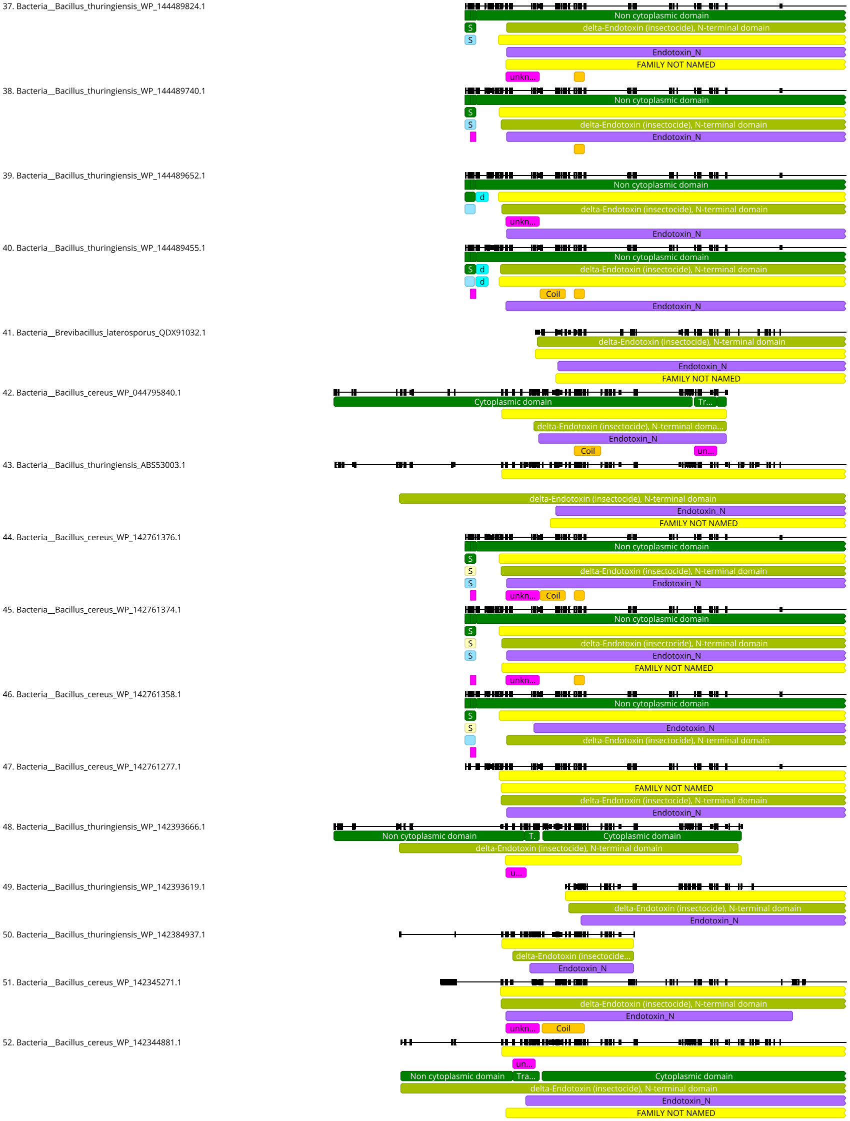
37. Bacteria__Bacillus_thuringiensis_WP_144489824.1
Non cytoplasmic domain
S
delta-Endotoxin (insectocide), N-terminal domain
S
Endotoxin_N
FAMILY NOT NAMED
unkn...
38. Bacteria__Bacillus_thuringiensis_WP_144489740.1
Non cytoplasmic domain
S
S
delta-Endotoxin (insectocide), N-terminal domain
Endotoxin_N
39. Bacteria__Bacillus_thuringiensis_WP_144489652.1
Non cytoplasmic domain
d
delta-Endotoxin (insectocide), N-terminal domain
unkn...
Endotoxin_N
40. Bacteria__Bacillus_thuringiensis_WP_144489455.1
Non cytoplasmic domain
S
d
delta-Endotoxin (insectocide), N-terminal domain
d
Coil
Endotoxin_N
41. Bacteria__Brevibacillus_laterosporus_QDX91032.1
delta-Endotoxin (insectocide), N-terminal domain
Endotoxin_N
FAMILY NOT NAMED
42. Bacteria__Bacillus_cereus_WP_044795840.1
Cytoplasmic domain
Tr...
delta-Endotoxin (insectocide), N-terminal doma...
Endotoxin_N
Coil
un...
43. Bacteria__Bacillus_thuringiensis_ABS53003.1
delta-Endotoxin (insectocide), N-terminal domain
Endotoxin_N
FAMILY NOT NAMED
44. Bacteria__Bacillus_cereus_WP_142761376.1
Non cytoplasmic domain
S
S
delta-Endotoxin (insectocide), N-terminal domain
S
Endotoxin_N
unkn...
Coil
45. Bacteria__Bacillus_cereus_WP_142761374.1
Non cytoplasmic domain
S
S
delta-Endotoxin (insectocide), N-terminal domain
S
Endotoxin_N
FAMILY NOT NAMED
unkn...
46. Bacteria__Bacillus_cereus_WP_142761358.1
Non cytoplasmic domain
S
S
Endotoxin_N
delta-Endotoxin (insectocide), N-terminal domain
47. Bacteria__Bacillus_cereus_WP_142761277.1
FAMILY NOT NAMED
delta-Endotoxin (insectocide), N-terminal domain
Endotoxin_N
48. Bacteria__Bacillus_thuringiensis_WP_142393666.1
Non cytoplasmic domain
T.
Cytoplasmic domain
delta-Endotoxin (insectocide), N-terminal domain
u...
49. Bacteria__Bacillus_thuringiensis_WP_142393619.1
delta-Endotoxin (insectocide), N-terminal domain
Endotoxin_N
50. Bacteria__Bacillus_thuringiensis_WP_142384937.1
delta-Endotoxin (insectocide...
Endotoxin_N
51. Bacteria__Bacillus_cereus_WP_142345271.1
delta-Endotoxin (insectocide), N-terminal domain
Endotoxin_N
unkn...
Coil
52. Bacteria__Bacillus_cereus_WP_142344881.1
un...
Non cytoplasmic domain
Tra...
Cytoplasmic domain
delta-Endotoxin (insectocide), N-terminal domain
Endotoxin_N
FAMILY NOT NAMED

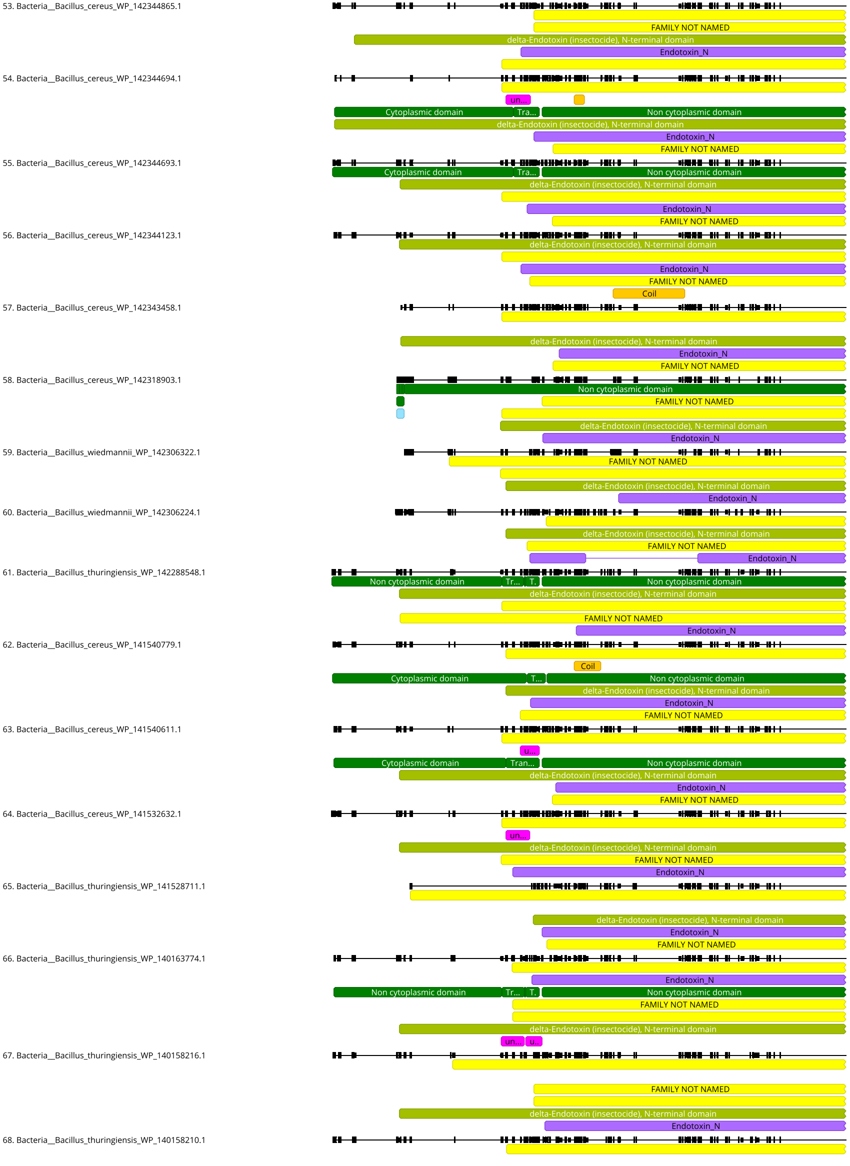
53. Bacteria__Bacillus_cereus_WP_142344865.1
FAMILY NOT NAMED
delta-Endotoxin (insecticide), N-terminal domain
Endotoxin_N
54. Bacteria__Bacillus_cereus_WP_142344694.1
un...
Cytoplasmic domain
Tra...
Non cytoplasmic domain
delta-Endotoxin (insecticide), N-terminal domain
Endotoxin_N
FAMILY NOT NAMED
55. Bacteria__Bacillus_cereus_WP_142344693.1
Cytoplasmic domain
Tra...
Non cytoplasmic domain
delta-Endotoxin (insecticide), N-terminal domain
Endotoxin_N
FAMILY NOT NAMED
56. Bacteria__Bacillus_cereus_WP_142344123.1
delta-Endotoxin (insecticide), N-terminal domain
Endotoxin_N
FAMILY NOT NAMED
Coil
57. Bacteria__Bacillus_cereus_WP_142343458.1
delta-Endotoxin (insecticide), N-terminal domain
Endotoxin_N
FAMILY NOT NAMED
58. Bacteria__Bacillus_cereus_WP_142318903.1
Non cytoplasmic domain
FAMILY NOT NAMED
delta-Endotoxin (insecticide), N-terminal domain
Endotoxin_N
59. Bacteria__Bacillus_wiedmannii_WP_142306322.1
FAMILY NOT NAMED
delta-Endotoxin (insecticide), N-terminal domain
Endotoxin_N
60. Bacteria__Bacillus_wiedmannii_WP_142306224.1
delta-Endotoxin (insecticide), N-terminal domain
FAMILY NOT NAMED
Endotoxin_N
61. Bacteria__Bacillus_thuringiensis_WP_142288548.1
Non cytoplasmic domain
Tr...
T.
Non cytoplasmic domain
delta-Endotoxin (insecticide), N-terminal domain
FAMILY NOT NAMED
Endotoxin_N
62. Bacteria__Bacillus_cereus_WP_141540779.1
Coil
Cytoplasmic domain
T...
Non cytoplasmic domain
delta-Endotoxin (insecticide), N-terminal domain
Endotoxin_N
FAMILY NOT NAMED
63. Bacteria__Bacillus_cereus_WP_141540611.1
u...
Cytoplasmic domain
Tran...
Non cytoplasmic domain
delta-Endotoxin (insecticide), N-terminal domain
Endotoxin_N
FAMILY NOT NAMED
64. Bacteria__Bacillus_cereus_WP_141532632.1
un...
delta-Endotoxin (insecticide), N-terminal domain
FAMILY NOT NAMED
Endotoxin_N
65. Bacteria__Bacillus_thuringiensis_WP_141528711.1
delta-Endotoxin (insecticide), N-terminal domain
Endotoxin_N
FAMILY NOT NAMED
66. Bacteria__Bacillus_thuringiensis_WP_140163774.1
Endotoxin_N
Non cytoplasmic domain
Tr...
T.
Non cytoplasmic domain
FAMILY NOT NAMED
delta-Endotoxin (insecticide), N-terminal domain
un...
u..
67. Bacteria__Bacillus_thuringiensis_WP_140158216.1
FAMILY NOT NAMED
delta-Endotoxin (insecticide), N-terminal domain
Endotoxin_N
68. Bacteria__Bacillus_thuringiensis_WP_140158210.1

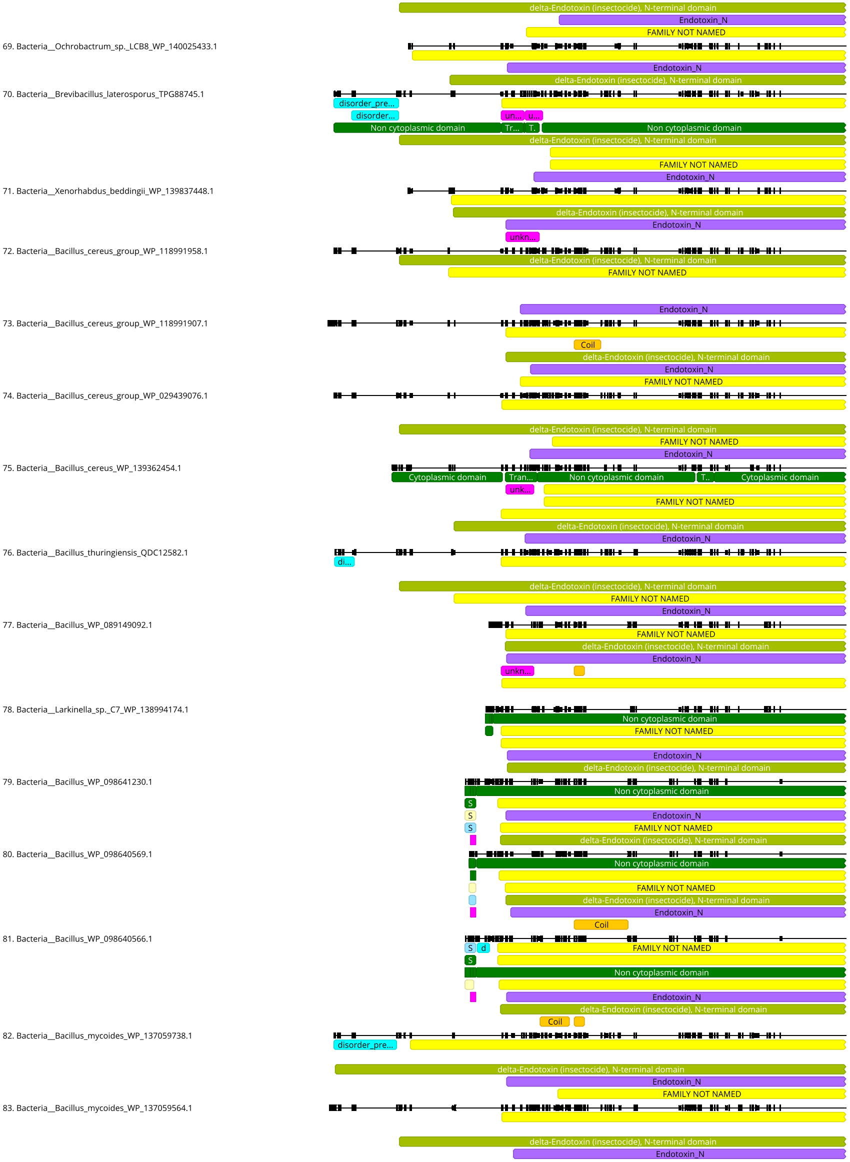

delta-Endotoxin (insectocide), N-terminal domain
Endotoxin_N
FAMILY NOT NAMED
69. Bacteria__Ochrobactrum_sp._LCB8_WP_140025433.1
Endotoxin_N
delta-Endotoxin (insectocide), N-terminal domain
70. Bacteria__Brevibacillus_laterosporus_TPG88745.1
disorder_pre...
disorder...
un...
u...
Non cytoplasmic domain
Tr...
T.
Non cytoplasmic domain
delta-Endotoxin (insectocide), N-terminal domain
FAMILY NOT NAMED
Endotoxin_N
71. Bacteria__Xenorhabdus_beddingii_WP_139837448.1
delta-Endotoxin (insectocide), N-terminal domain
Endotoxin_N
unkn...
72. Bacteria__Bacillus_cereus_group_WP_118991958.1
delta-Endotoxin (insectocide), N-terminal domain
FAMILY NOT NAMED
Endotoxin_N
73. Bacteria__Bacillus_cereus_group_WP_118991907.1
Coil
delta-Endotoxin (insectocide), N-terminal domain
Endotoxin_N
FAMILY NOT NAMED
74. Bacteria__Bacillus_cereus_group_WP_029439076.1
delta-Endotoxin (insectocide), N-terminal domain
FAMILY NOT NAMED
Endotoxin_N
75. Bacteria__Bacillus_cereus_WP_139362454.1
Cytoplasmic domain
Tran...
Non cytoplasmic domain
T..
Cytoplasmic domain
unk...
FAMILY NOT NAMED
delta-Endotoxin (insectocide), N-terminal domain
Endotoxin_N
76. Bacteria__Bacillus_thuringiensis_QDC12582.1
di...
delta-Endotoxin (insectocide), N-terminal domain
FAMILY NOT NAMED
Endotoxin_N
77. Bacteria__Bacillus_WP_089149092.1
FAMILY NOT NAMED
delta-Endotoxin (insectocide), N-terminal domain
Endotoxin_N
unkn...
78. Bacteria__Larkinella_sp._C7_WP_138994174.1
Non cytoplasmic domain
FAMILY NOT NAMED
Endotoxin_N
delta-Endotoxin (insectocide), N-terminal domain
79. Bacteria__Bacillus_WP_098641230.1
Non cytoplasmic domain
S
S
Endotoxin_N
S
FAMILY NOT NAMED
delta-Endotoxin (insectocide), N-terminal domain
80. Bacteria__Bacillus_WP_098640569.1
Non cytoplasmic domain
FAMILY NOT NAMED
delta-Endotoxin (insectocide), N-terminal domain
Endotoxin_N
Coil
81. Bacteria__Bacillus_WP_098640566.1
S
d
FAMILY NOT NAMED
S
Non cytoplasmic domain
Endotoxin_N
delta-Endotoxin (insectocide), N-terminal domain
Coil
82. Bacteria__Bacillus_mycoides_WP_137059738.1
disorder_pre...
delta-Endotoxin (insectocide), N-terminal domain
Endotoxin_N
FAMILY NOT NAMED
83. Bacteria__Bacillus_mycoides_WP_137059564.1
delta-Endotoxin (insectocide), N-terminal domain
Endotoxin_N

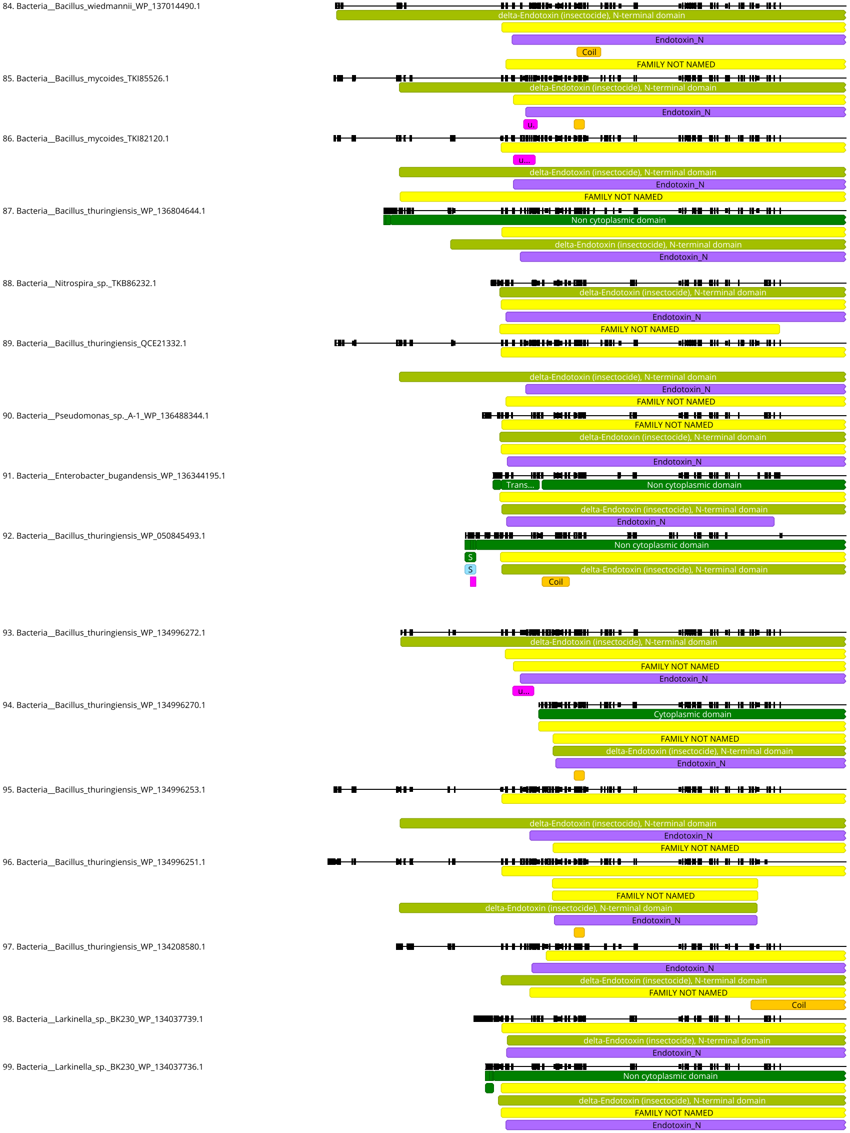

84. Bacteria__Bacillus_wiedmannii_WP_137014490.1
delta-Endotoxin (insectocide), N-terminal domain
Endotoxin_N
Coil
FAMILY NOT NAMED
85. Bacteria__Bacillus_mycoides_TKI85526.1
delta-Endotoxin (insectocide), N-terminal domain
Endotoxin_N
u.
86. Bacteria__Bacillus_mycoides_TKI82120.1
u...
delta-Endotoxin (insectocide), N-terminal domain
Endotoxin_N
FAMILY NOT NAMED
87. Bacteria__Bacillus_thuringiensis_WP_136804644.1
Non cytoplasmic domain
delta-Endotoxin (insectocide), N-terminal domain
Endotoxin_N
88. Bacteria__Nitrospira_sp._TKB86232.1
delta-Endotoxin (insectocide), N-terminal domain
Endotoxin_N
FAMILY NOT NAMED
89. Bacteria__Bacillus_thuringiensis_QCE21332.1
delta-Endotoxin (insectocide), N-terminal domain
Endotoxin_N
FAMILY NOT NAMED
90. Bacteria__Pseudomonas_sp._A-1_WP_136488344.1
FAMILY NOT NAMED
delta-Endotoxin (insectocide), N-terminal domain
Endotoxin_N
91. Bacteria__Enterobacter_bugandensis_WP_136344195.1
Trans...
Non cytoplasmic domain
delta-Endotoxin (insectocide), N-terminal domain
Endotoxin_N
92. Bacteria__Bacillus_thuringiensis_WP_050845493.1
Non cytoplasmic domain
S
S
delta-Endotoxin (insectocide), N-terminal domain
Coil
93. Bacteria__Bacillus_thuringiensis_WP_134996272.1
delta-Endotoxin (insectocide), N-terminal domain
FAMILY NOT NAMED
Endotoxin_N
u...
94. Bacteria__Bacillus_thuringiensis_WP_134996270.1
Cytoplasmic domain
FAMILY NOT NAMED
delta-Endotoxin (insectocide), N-terminal domain
Endotoxin_N
95. Bacteria__Bacillus_thuringiensis_WP_134996253.1
delta-Endotoxin (insectocide), N-terminal domain
Endotoxin_N
FAMILY NOT NAMED
96. Bacteria__Bacillus_thuringiensis_WP_134996251.1
FAMILY NOT NAMED
delta-Endotoxin (insectocide), N-terminal domain
Endotoxin_N
97. Bacteria__Bacillus_thuringiensis_WP_134208580.1
Endotoxin_N
delta-Endotoxin (insectocide), N-terminal domain
FAMILY NOT NAMED
Coil
98. Bacteria__Larkinella_sp._BK230_WP_134037739.1
delta-Endotoxin (insectocide), N-terminal domain
Endotoxin_N
99. Bacteria__Larkinella_sp._BK230_WP_134037736.1
Non cytoplasmic domain
delta-Endotoxin (insectocide), N-terminal domain
FAMILY NOT NAMED
Endotoxin_N

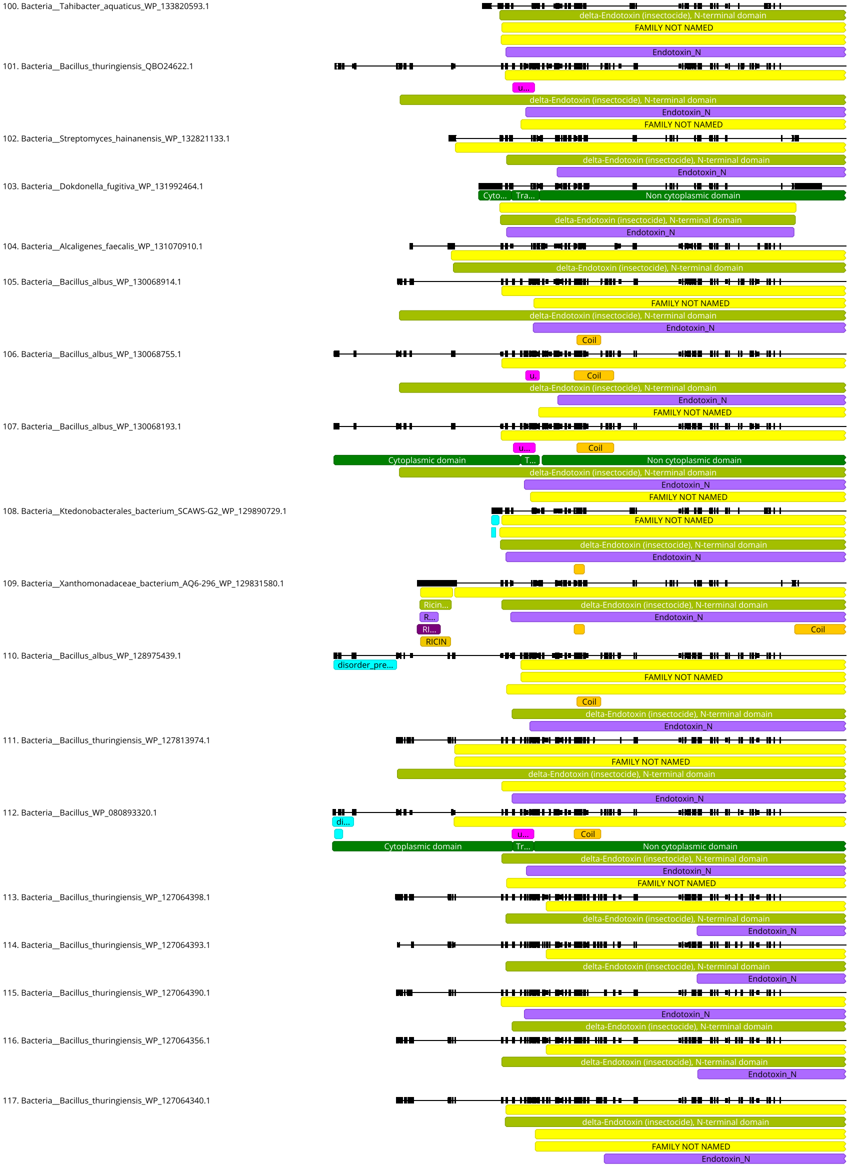

100. Bacteria__Tahibacter_aquaticus_WP_133820593.1
delta-Endotoxin (insectocide), N-terminal domain
FAMILY NOT NAMED
Endotoxin_N
101. Bacteria__Bacillus_thuringiensis_QBO24622.1
u...
delta-Endotoxin (insectocide), N-terminal domain
Endotoxin_N
FAMILY NOT NAMED
102. Bacteria__Streptomyces_hainanensis_WP_132821133.1
delta-Endotoxin (insectocide), N-terminal domain
Endotoxin_N
103. Bacteria__Dokdonella_fugitiva_WP_131992464.1
Cyto...
Tra...
Non cytoplasmic domain
delta-Endotoxin (insectocide), N-terminal domain
Endotoxin_N
104. Bacteria__Alcaligenes_faecalis_WP_131070910.1
delta-Endotoxin (insectocide), N-terminal domain
105. Bacteria__Bacillus_albus_WP_130068914.1
FAMILY NOT NAMED
delta-Endotoxin (insectocide), N-terminal domain
Endotoxin_N
Coil
106. Bacteria__Bacillus_albus_WP_130068755.1
u.
Coil
delta-Endotoxin (insectocide), N-terminal domain
Endotoxin_N
FAMILY NOT NAMED
107. Bacteria__Bacillus_albus_WP_130068193.1
u...
Coil
Cytoplasmic domain
T...
Non cytoplasmic domain
delta-Endotoxin (insectocide), N-terminal domain
Endotoxin_N
FAMILY NOT NAMED
108. Bacteria__Ktedonobacterales_bacterium_SCAWS-G2_WP_129890729.1
FAMILY NOT NAMED
delta-Endotoxin (insectocide), N-terminal domain
Endotoxin_N
109. Bacteria__Xanthomonadaceae_bacterium_AQ6-296_WP_129831580.1
Ricin...
delta-Endotoxin (insectocide), N-terminal domain
R...
Endotoxin_N
RI...
Coil
RICIN
110. Bacteria__Bacillus_albus_WP_128975439.1
disorder_pre...
FAMILY NOT NAMED
Coil
delta-Endotoxin (insectocide), N-terminal domain
Endotoxin_N
111. Bacteria__Bacillus_thuringiensis_WP_127813974.1
FAMILY NOT NAMED
delta-Endotoxin (insectocide), N-terminal domain
Endotoxin_N
112. Bacteria__Bacillus_WP_080893320.1
di...
u...
Coil
Cytoplasmic domain
Tr...
Non cytoplasmic domain
delta-Endotoxin (insectocide), N-terminal domain
Endotoxin_N
FAMILY NOT NAMED
113. Bacteria__Bacillus_thuringiensis_WP_127064398.1
delta-Endotoxin (insectocide), N-terminal domain
Endotoxin_N
114. Bacteria__Bacillus_thuringiensis_WP_127064393.1
delta-Endotoxin (insectocide), N-terminal domain
Endotoxin_N
115. Bacteria__Bacillus_thuringiensis_WP_127064390.1
Endotoxin_N
delta-Endotoxin (insectocide), N-terminal domain
116. Bacteria__Bacillus_thuringiensis_WP_127064356.1
delta-Endotoxin (insectocide), N-terminal domain
Endotoxin_N
117. Bacteria__Bacillus_thuringiensis_WP_127064340.1
delta-Endotoxin (insectocide), N-terminal domain
FAMILY NOT NAMED
Endotoxin_N

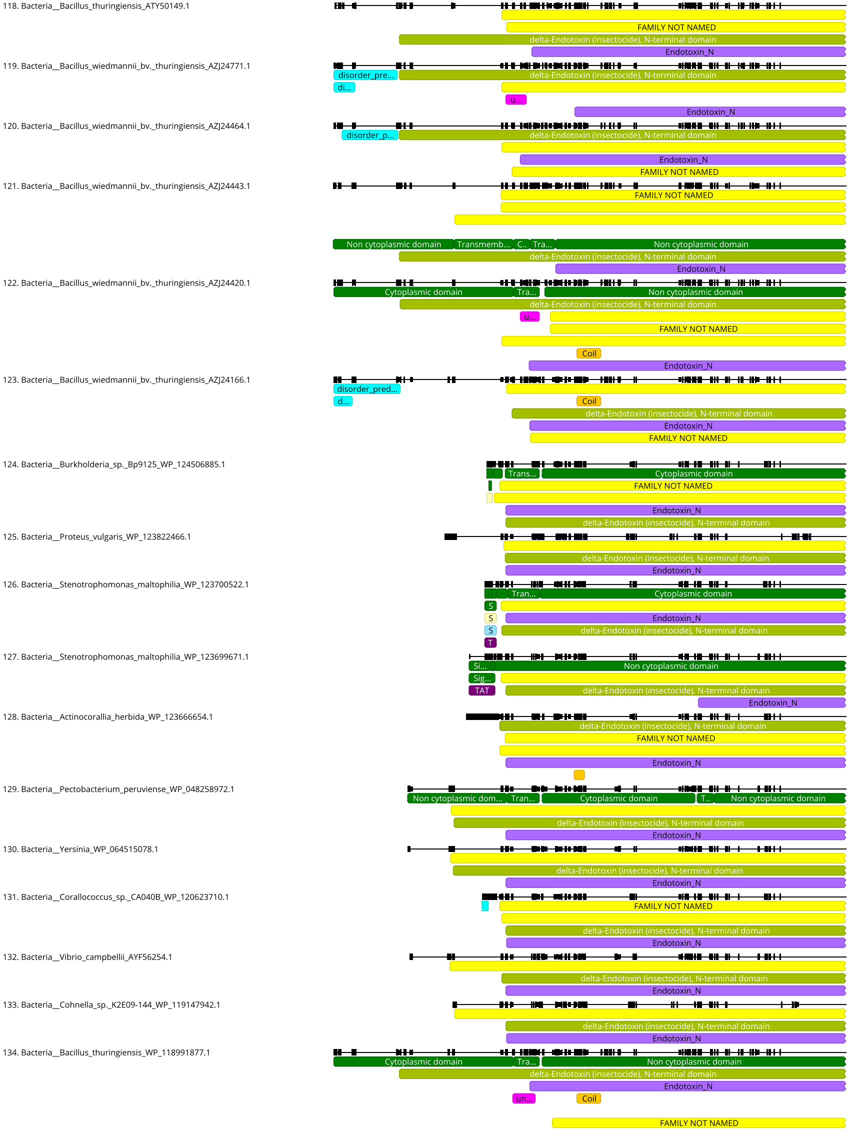
118. Bacteria__Bacillus_thuringiensis_ATY50149.1
FAMILY NOT NAMED
delta-Endotoxin (insectocide), N-terminal domain
Endotoxin_N
119. Bacteria__Bacillus_wiedmannii_bv._thuringiensis_AZJ24771.1
disorder_pre...
delta-Endotoxin (insectocide), N-terminal domain
di...
u...
Endotoxin_N
120. Bacteria__Bacillus_wiedmannii_bv._thuringiensis_AZJ24464.1
disorder_p...
delta-Endotoxin (insectocide), N-terminal domain
Endotoxin_N
FAMILY NOT NAMED
121. Bacteria__Bacillus_wiedmannii_bv._thuringiensis_AZJ24443.1
FAMILY NOT NAMED
Non cytoplasmic domain
Transmemb...
C...
Tra...
Non cytoplasmic domain
delta-Endotoxin (insectocide), N-terminal domain
Endotoxin_N
122. Bacteria__Bacillus_wiedmannii_bv._thuringiensis_AZJ24420.1
Cytoplasmic domain
Tra...
Non cytoplasmic domain
delta-Endotoxin (insectocide), N-terminal domain
u...
FAMILY NOT NAMED
Coil
Endotoxin_N
123. Bacteria__Bacillus_wiedmannii_bv._thuringiensis_AZJ24166.1
disorder_pred...
d...
Coil
delta-Endotoxin (insectocide), N-terminal domain
Endotoxin_N
FAMILY NOT NAMED
124. Bacteria__Burkholderia_sp._Bp9125_WP_124506885.1
Trans...
Cytoplasmic domain
FAMILY NOT NAMED
Endotoxin_N
delta-Endotoxin (insectocide), N-terminal domain
125. Bacteria__Proteus_vulgaris_WP_123822466.1
delta-Endotoxin (insectocide), N-terminal domain
Endotoxin_N
126. Bacteria__Stenotrophomonas_maltophilia_WP_123700522.1
Tran...
Cytoplasmic domain
S
S
Endotoxin_N
S
delta-Endotoxin (insectocide), N-terminal domain
T
127. Bacteria__Stenotrophomonas_maltophilia_WP_123699671.1
Si...
Non cytoplasmic domain
Sig...
TAT
delta-Endotoxin (insectocide), N-terminal domain
Endotoxin_N
128. Bacteria__Actinocorallia_herbida_WP_123666654.1
delta-Endotoxin (insectocide), N-terminal domain
FAMILY NOT NAMED
Endotoxin_N
129. Bacteria__Pectobacterium_peruviense_WP_048258972.1
Non cytoplasmic dom...
Tran...
Cytoplasmic domain
T...
Non cytoplasmic domain
delta-Endotoxin (insectocide), N-terminal domain
Endotoxin_N
130. Bacteria__Yersinia_WP_064515078.1
delta-Endotoxin (insectocide), N-terminal domain
Endotoxin_N
131. Bacteria__Corallococcus_sp._CA040B_WP_120623710.1
FAMILY NOT NAMED
delta-Endotoxin (insectocide), N-terminal domain
Endotoxin_N
132. Bacteria__Vibrio_campbellii_AYF56254.1
delta-Endotoxin (insectocide), N-terminal domain
Endotoxin_N
133. Bacteria__Cohnella_sp._K2E09-144_WP_119147942.1
delta-Endotoxin (insectocide), N-terminal domain
Endotoxin_N
134. Bacteria__Bacillus_thuringiensis_WP_118991877.1
Cytoplasmic domain
Tra...
Non cytoplasmic domain
delta-Endotoxin (insectocide), N-terminal domain
Endotoxin_N
un...
Coil
FAMILY NOT NAMED

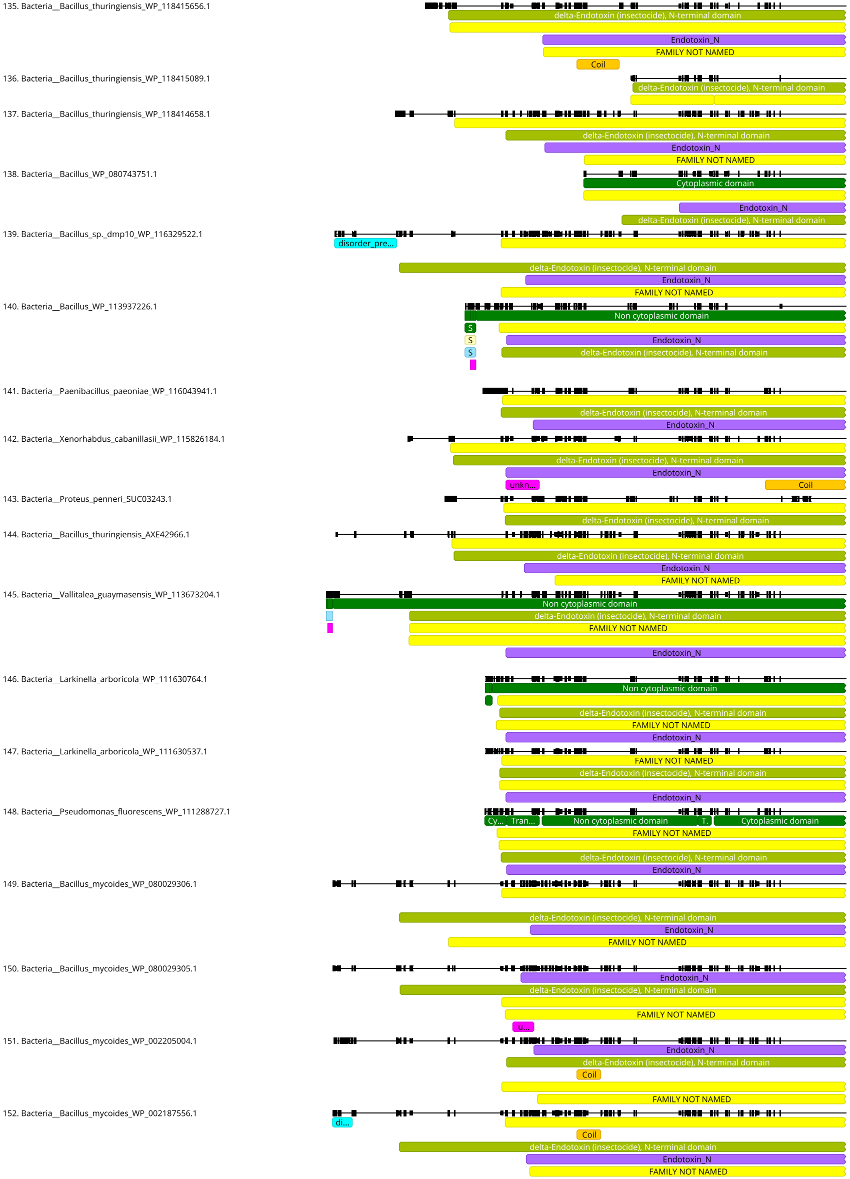
135. Bacteria__Bacillus_thuringiensis_WP_118415656.1
delta-Endotoxin (insectocide), N-terminal domain
Endotoxin_N
FAMILY NOT NAMED
Coil
136. Bacteria__Bacillus_thuringiensis_WP_118415089.1
delta-Endotoxin (insectocide), N-terminal domain
137. Bacteria__Bacillus_thuringiensis_WP_118414658.1
delta-Endotoxin (insectocide), N-terminal domain
Endotoxin_N
FAMILY NOT NAMED
138. Bacteria__Bacillus_WP_080743751.1
Cytoplasmic domain
Endotoxin_N
delta-Endotoxin (insectocide), N-terminal domain
139. Bacteria__Bacillus_sp._dmp10_WP_116329522.1
disorder_pre...
delta-Endotoxin (insectocide), N-terminal domain
Endotoxin_N
FAMILY NOT NAMED
140. Bacteria__Bacillus_WP_113937226.1
Non cytoplasmic domain
S
S
S
Endotoxin_N
delta-Endotoxin (insectocide), N-terminal domain
141. Bacteria__Paenibacillus_paeoniae_WP_116043941.1
delta-Endotoxin (insectocide), N-terminal domain
Endotoxin_N
142. Bacteria__Xenorhabdus_cabanillasii_WP_115826184.1
delta-Endotoxin (insectocide), N-terminal domain
Endotoxin_N
unkn...
Coil
143. Bacteria__Proteus_penneri_SUC03243.1
delta-Endotoxin (insectocide), N-terminal domain
144. Bacteria__Bacillus_thuringiensis_AXE42966.1
delta-Endotoxin (insectocide), N-terminal domain
Endotoxin_N
FAMILY NOT NAMED
145. Bacteria__Vallitalea_guaymasensis_WP_113673204.1
Non cytoplasmic domain
delta-Endotoxin (insectocide), N-terminal domain
FAMILY NOT NAMED
Endotoxin_N
146. Bacteria__Larkinella_arboricola_WP_111630764.1
Non cytoplasmic domain
delta-Endotoxin (insectocide), N-terminal domain
FAMILY NOT NAMED
Endotoxin_N
147. Bacteria__Larkinella_arboricola_WP_111630537.1
FAMILY NOT NAMED
delta-Endotoxin (insectocide), N-terminal domain
Endotoxin_N
148. Bacteria__Pseudomonas_fluorescens_WP_111288727.1
Cy...
Tran...
Non cytoplasmic domain
T.
Cytoplasmic domain
FAMILY NOT NAMED
delta-Endotoxin (insectocide), N-terminal domain
Endotoxin_N
149. Bacteria__Bacillus_mycoides_WP_080029306.1
delta-Endotoxin (insectocide), N-terminal domain
Endotoxin_N
FAMILY NOT NAMED
150. Bacteria__Bacillus_mycoides_WP_080029305.1
Endotoxin_N
delta-Endotoxin (insectocide), N-terminal domain
FAMILY NOT NAMED
u...
151. Bacteria__Bacillus_mycoides_WP_002205004.1
Endotoxin_N
delta-Endotoxin (insectocide), N-terminal domain
Coil
FAMILY NOT NAMED
152. Bacteria__Bacillus_mycoides_WP_002187556.1
di...
Coil
delta-Endotoxin (insectocide), N-terminal domain
Endotoxin_N
FAMILY NOT NAMED

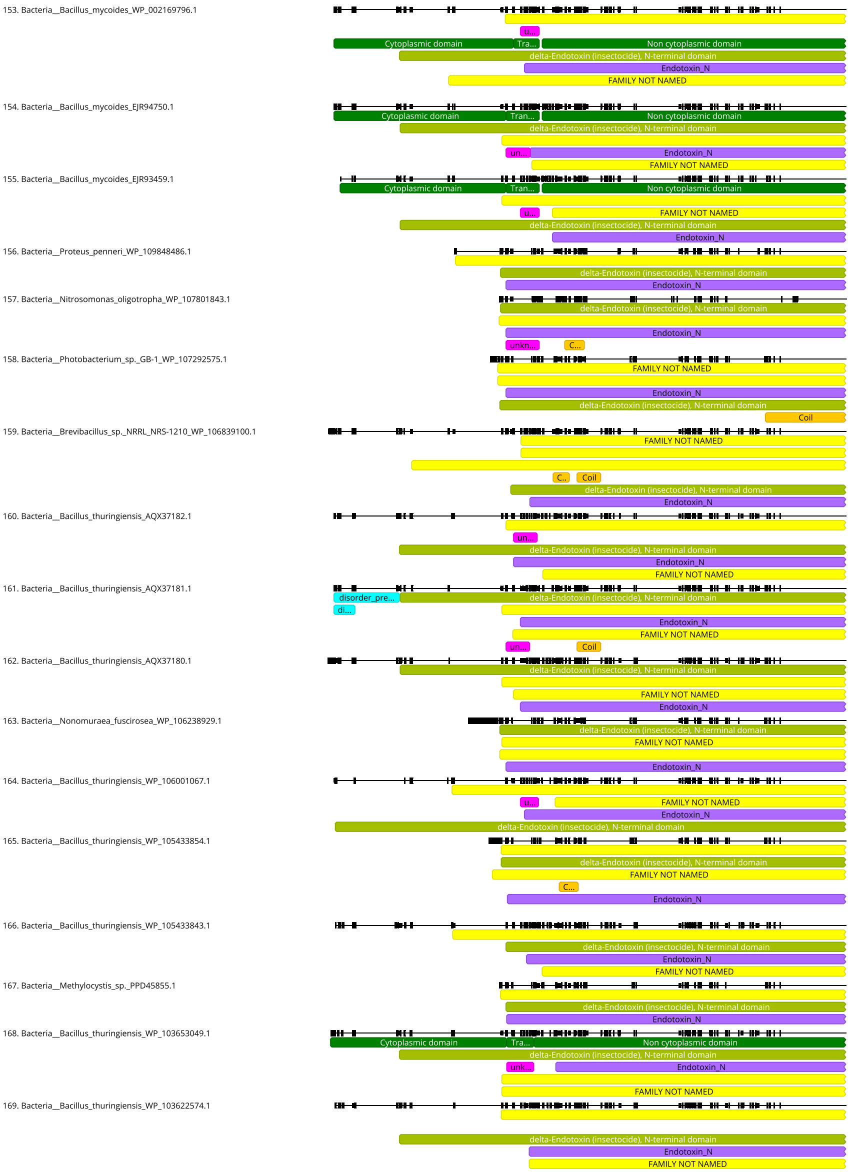
153. Bacteria__Bacillus_mycoides_WP_002169796.1
u...
Cytoplasmic domain
Tra...
Non cytoplasmic domain
delta-Endotoxin (insectocide), N-terminal domain
Endotoxin_N
FAMILY NOT NAMED
154. Bacteria__Bacillus_mycoides_EJR94750.1
Cytoplasmic domain
Tran...
Non cytoplasmic domain
delta-Endotoxin (insectocide), N-terminal domain
un...
Endotoxin_N
FAMILY NOT NAMED
155. Bacteria__Bacillus_mycoides_EJR93459.1
Cytoplasmic domain
Tran...
Non cytoplasmic domain
u...
FAMILY NOT NAMED
delta-Endotoxin (insectocide), N-terminal domain
Endotoxin_N
156. Bacteria__Proteus_penneri_WP_109848486.1
delta-Endotoxin (insectocide), N-terminal domain
Endotoxin_N
157. Bacteria__Nitrosomonas_oligotropha_WP_107801843.1
delta-Endotoxin (insectocide), N-terminal domain
Endotoxin_N
unkn...
C...
158. Bacteria__Photobacterium_sp._GB-1_WP_107292575.1
FAMILY NOT NAMED
Endotoxin_N
delta-Endotoxin (insectocide), N-terminal domain
Coil
159. Bacteria__Brevibacillus_sp._NRRL_NRS-1210_WP_106839100.1
FAMILY NOT NAMED
C..
Coil
delta-Endotoxin (insectocide), N-terminal domain
Endotoxin_N
160. Bacteria__Bacillus_thuringiensis_AQX37182.1
un...
delta-Endotoxin (insectocide), N-terminal domain
Endotoxin_N
FAMILY NOT NAMED
161. Bacteria__Bacillus_thuringiensis_AQX37181.1
disorder_pre...
delta-Endotoxin (insectocide), N-terminal domain
di...
Endotoxin_N
FAMILY NOT NAMED
un...
Coil
162. Bacteria__Bacillus_thuringiensis_AQX37180.1
delta-Endotoxin (insectocide), N-terminal domain
FAMILY NOT NAMED
Endotoxin_N
163. Bacteria__Nonomuraea_fuscirosea_WP_106238929.1
delta-Endotoxin (insectocide), N-terminal domain
FAMILY NOT NAMED
Endotoxin_N
164. Bacteria__Bacillus_thuringiensis_WP_106001067.1
u...
FAMILY NOT NAMED
Endotoxin_N
delta-Endotoxin (insectocide), N-terminal domain
165. Bacteria__Bacillus_thuringiensis_WP_105433854.1
delta-Endotoxin (insectocide), N-terminal domain
FAMILY NOT NAMED
C...
Endotoxin_N
166. Bacteria__Bacillus_thuringiensis_WP_105433843.1
delta-Endotoxin (insectocide), N-terminal domain
Endotoxin_N
FAMILY NOT NAMED
167. Bacteria__Methylocystis_sp._PPD45855.1
delta-Endotoxin (insectocide), N-terminal domain
Endotoxin_N
168. Bacteria__Bacillus_thuringiensis_WP_103653049.1
Cytoplasmic domain
Tra...
Non cytoplasmic domain
delta-Endotoxin (insectocide), N-terminal domain
unk...
Endotoxin_N
FAMILY NOT NAMED
169. Bacteria__Bacillus_thuringiensis_WP_103622574.1
delta-Endotoxin (insectocide), N-terminal domain
Endotoxin_N
FAMILY NOT NAMED

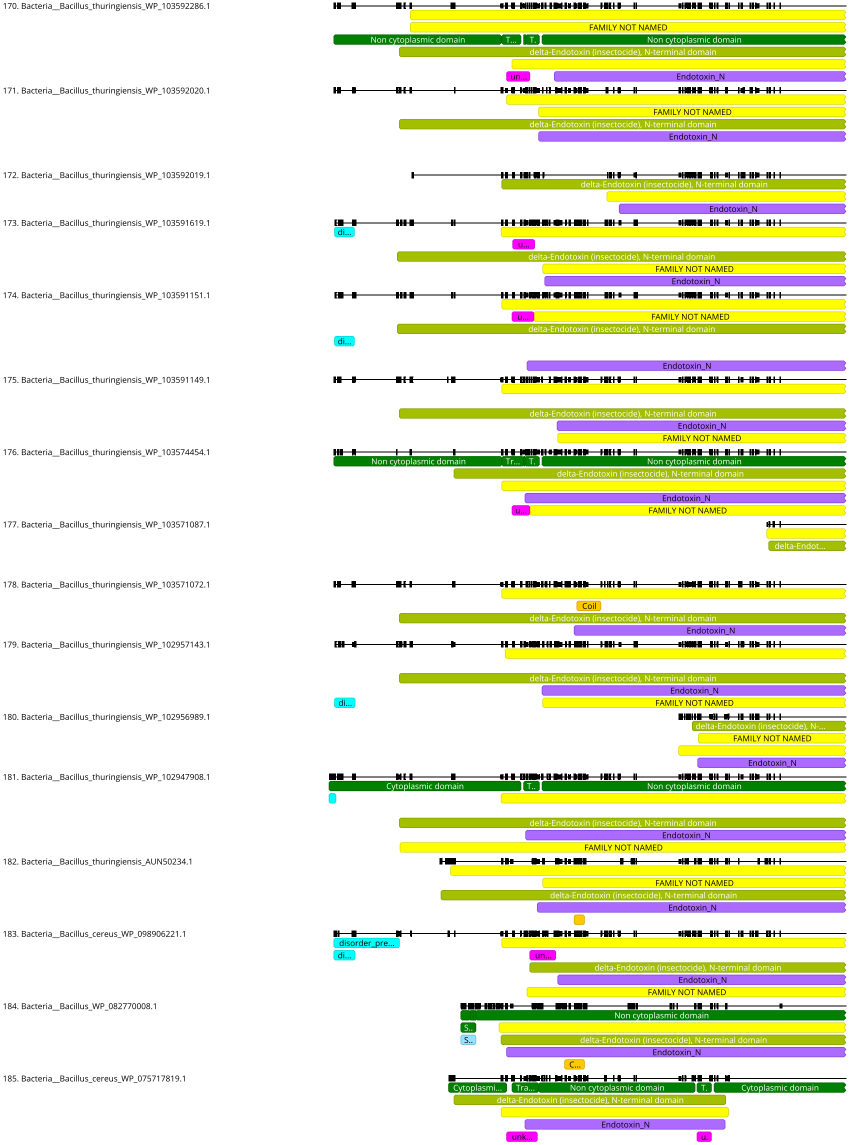

170. Bacteria__Bacillus_thuringiensis_WP_103592286.1
FAMILY NOT NAMED
Non cytoplasmic domain
T...
T.
Non cytoplasmic domain
delta-Endotoxin (insectocide), N-terminal domain
un...
Endotoxin_N
171. Bacteria__Bacillus_thuringiensis_WP_103592020.1
FAMILY NOT NAMED
delta-Endotoxin (insectocide), N-terminal domain
Endotoxin_N
172. Bacteria__Bacillus_thuringiensis_WP_103592019.1
delta-Endotoxin (insectocide), N-terminal domain
Endotoxin_N
173. Bacteria__Bacillus_thuringiensis_WP_103591619.1
di...
u...
delta-Endotoxin (insectocide), N-terminal domain
FAMILY NOT NAMED
Endotoxin_N
174. Bacteria__Bacillus_thuringiensis_WP_103591151.1
u...
FAMILY NOT NAMED
delta-Endotoxin (insectocide), N-terminal domain
di...
Endotoxin_N
175. Bacteria__Bacillus_thuringiensis_WP_103591149.1
delta-Endotoxin (insectocide), N-terminal domain
Endotoxin_N
FAMILY NOT NAMED
176. Bacteria__Bacillus_thuringiensis_WP_103574454.1
Non cytoplasmic domain
Tr...
T.
Non cytoplasmic domain
delta-Endotoxin (insectocide), N-terminal domain
Endotoxin_N
u...
FAMILY NOT NAMED
177. Bacteria__Bacillus_thuringiensis_WP_103571087.1
delta-Endot...
178. Bacteria__Bacillus_thuringiensis_WP_103571072.1
Coil
delta-Endotoxin (insectocide), N-terminal domain
Endotoxin_N
179. Bacteria__Bacillus_thuringiensis_WP_102957143.1
delta-Endotoxin (insectocide), N-terminal domain
Endotoxin_N
di...
FAMILY NOT NAMED
180. Bacteria__Bacillus_thuringiensis_WP_102956989.1
delta-Endotoxin (insectocide), N-...
FAMILY NOT NAMED
Endotoxin_N
181. Bacteria__Bacillus_thuringiensis_WP_102947908.1
Cytoplasmic domain
T..
Non cytoplasmic domain
delta-Endotoxin (insectocide), N-terminal domain
Endotoxin_N
FAMILY NOT NAMED
182. Bacteria__Bacillus_thuringiensis_AUN50234.1
FAMILY NOT NAMED
delta-Endotoxin (insectocide), N-terminal domain
Endotoxin_N
183. Bacteria__Bacillus_cereus_WP_098906221.1
disorder_pre...
di...
un...
delta-Endotoxin (insectocide), N-terminal domain
Endotoxin_N
FAMILY NOT NAMED
184. Bacteria__Bacillus_WP_082770008.1
Non cytoplasmic domain
S..
S..
delta-Endotoxin (insectocide), N-terminal domain
Endotoxin_N
C...
185. Bacteria__Bacillus_cereus_WP_075717819.1
Cytoplasmi...
Tra...
Non cytoplasmic domain
T.
Cytoplasmic domain
delta-Endotoxin (insectocide), N-terminal domain
Endotoxin_N
unk...
u.

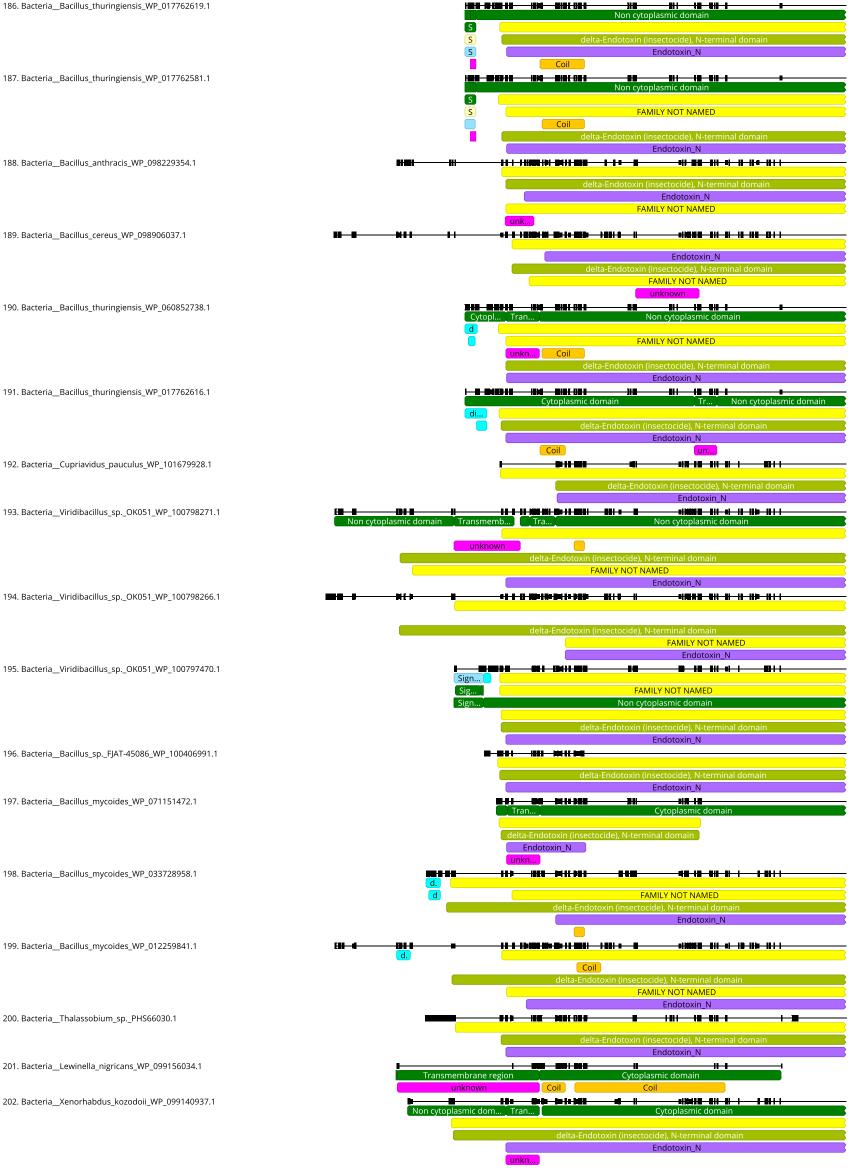
186. Bacteria__Bacillus_thuringiensis_WP_017762619.1
Non cytoplasmic domain
S
delta-Endotoxin (insectocide), N-terminal domain
S
Endotoxin_N
S
Coil
187. Bacteria__Bacillus_thuringiensis_WP_017762581.1
Non cytoplasmic domain
S
FAMILY NOT NAMED
S
Coil
delta-Endotoxin (insectocide), N-terminal domain
Endotoxin_N
188. Bacteria__Bacillus_anthracis_WP_098229354.1
delta-Endotoxin (insectocide), N-terminal domain
Endotoxin_N
FAMILY NOT NAMED
unk...
189. Bacteria__Bacillus_cereus_WP_098906037.1
Endotoxin_N
delta-Endotoxin (insectocide), N-terminal domain
FAMILY NOT NAMED
unknown
190. Bacteria__Bacillus_thuringiensis_WP_060852738.1
Cytopl...
Tran...
Non cytoplasmic domain
d
FAMILY NOT NAMED
unkn...
Coil
delta-Endotoxin (insectocide), N-terminal domain
Endotoxin_N
191. Bacteria__Bacillus_thuringiensis_WP_017762616.1
Cytoplasmic domain
Tr...
Non cytoplasmic domain
di...
delta-Endotoxin (insectocide), N-terminal domain
Endotoxin_N
Coil
un...
192. Bacteria__Cupriavidus_pauculus_WP_101679928.1
delta-Endotoxin (insectocide), N-terminal domain
Endotoxin_N
193. Bacteria__Viridibacillus_sp._OK051_WP_100798271.1
Non cytoplasmic domain
Transmemb...
Tra...
Non cytoplasmic domain
unknown
delta-Endotoxin (insectocide), N-terminal domain
FAMILY NOT NAMED
Endotoxin_N
194. Bacteria__Viridibacillus_sp._OK051_WP_100798266.1
delta-Endotoxin (insectocide), N-terminal domain
FAMILY NOT NAMED
Endotoxin_N
195. Bacteria__Viridibacillus_sp._OK051_WP_100797470.1
Sign...
Sig...
FAMILY NOT NAMED
Sign...
Non cytoplasmic domain
delta-Endotoxin (insectocide), N-terminal domain
Endotoxin_N
196. Bacteria__Bacillus_sp._FJAT-45086_WP_100406991.1
delta-Endotoxin (insectocide), N-terminal domain
Endotoxin_N
197. Bacteria__Bacillus_mycoides_WP_071151472.1
Tran...
Cytoplasmic domain
delta-Endotoxin (insectocide), N-terminal domain
Endotoxin_N
unkn...
198. Bacteria__Bacillus_mycoides_WP_033728958.1
d.
d
FAMILY NOT NAMED
delta-Endotoxin (insectocide), N-terminal domain
Endotoxin_N
199. Bacteria__Bacillus_mycoides_WP_012259841.1
d.
Coil
delta-Endotoxin (insectocide), N-terminal domain
FAMILY NOT NAMED
Endotoxin_N
200. Bacteria__Thalassobium_sp._PHS66030.1
delta-Endotoxin (insectocide), N-terminal domain
Endotoxin_N
201. Bacteria__Lewinella_nigricans_WP_099156034.1
Transmembrane region
Cytoplasmic domain
unknown
Coil
Coil
202. Bacteria__Xenorhabdus_kozodoii_WP_099140937.1
Non cytoplasmic dom...
Tran...
Cytoplasmic domain
delta-Endotoxin (insectocide), N-terminal domain
Endotoxin_N
unkn...

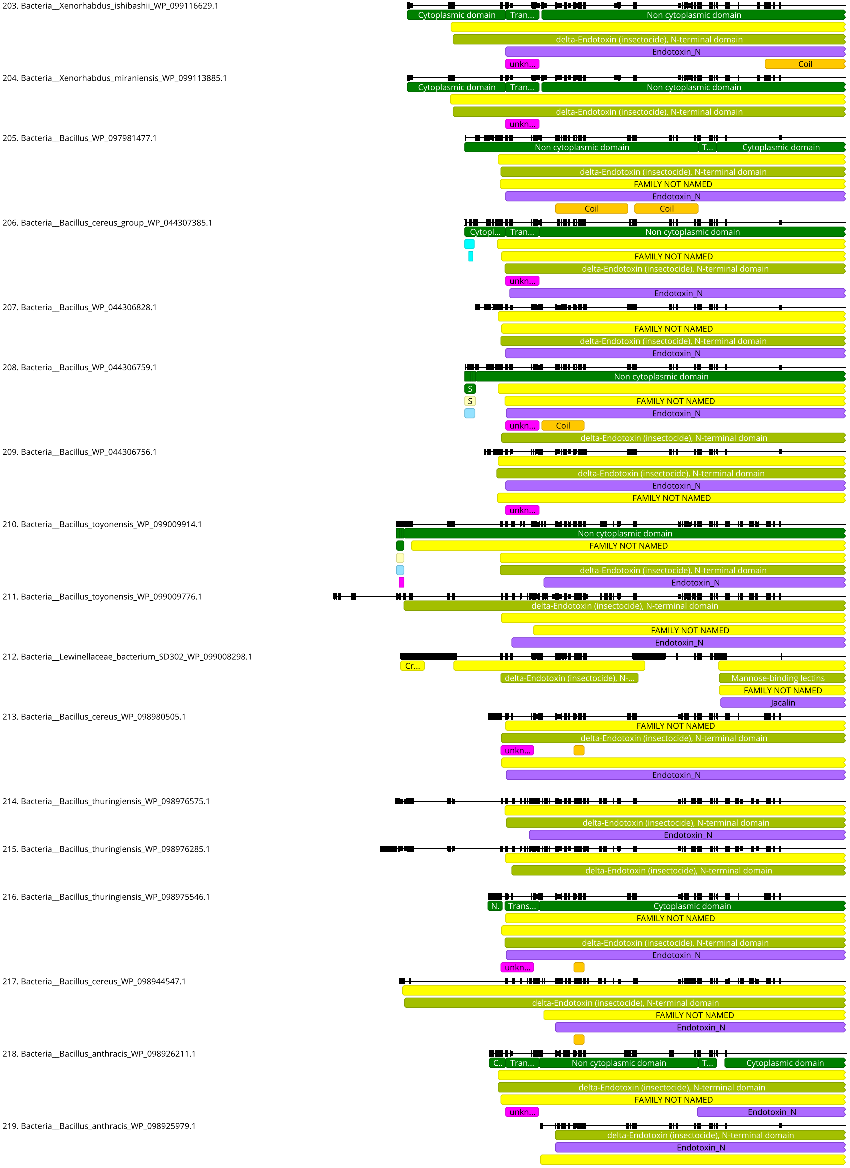
203. Bacteria__Xenorhabdus_ishibashii_WP_099116629.1
Cytoplasmic domain
Tran...
Non cytoplasmic domain
delta-Endotoxin (insecticide), N-terminal domain
Endotoxin_N
unkn...
Coil
204. Bacteria__Xenorhabdus_miraniensis_WP_099113885.1
Cytoplasmic domain
Tran...
Non cytoplasmic domain
delta-Endotoxin (insecticide), N-terminal domain
unkn...
205. Bacteria__Bacillus_WP_097981477.1
Non cytoplasmic domain
T...
Cytoplasmic domain
delta-Endotoxin (insecticide), N-terminal domain
FAMILY NOT NAMED
Endotoxin_N
Coil
Coil
206. Bacteria__Bacillus_cereus_group_WP_044307385.1
Cytopl...
Tran...
Non cytoplasmic domain
FAMILY NOT NAMED
delta-Endotoxin (insecticide), N-terminal domain
unkn...
Endotoxin_N
207. Bacteria__Bacillus_WP_044306828.1
FAMILY NOT NAMED
delta-Endotoxin (insecticide), N-terminal domain
Endotoxin_N
208. Bacteria__Bacillus_WP_044306759.1
Non cytoplasmic domain
S
S
FAMILY NOT NAMED
Endotoxin_N
unkn...
Coil
delta-Endotoxin (insecticide), N-terminal domain
209. Bacteria__Bacillus_WP_044306756.1
delta-Endotoxin (insecticide), N-terminal domain
Endotoxin_N
FAMILY NOT NAMED
unkn...
210. Bacteria__Bacillus_toyonensis_WP_099009914.1
Non cytoplasmic domain
FAMILY NOT NAMED
delta-Endotoxin (insecticide), N-terminal domain
Endotoxin_N
211. Bacteria__Bacillus_toyonensis_WP_099009776.1
delta-Endotoxin (insecticide), N-terminal domain
FAMILY NOT NAMED
Endotoxin_N
212. Bacteria__Lewinellaceae_bacterium_SD302_WP_099008298.1
Cr...
delta-Endotoxin (insecticide), N-...
Mannose-binding lectins
FAMILY NOT NAMED
Jacalin
213. Bacteria__Bacillus_cereus_WP_098980505.1
FAMILY NOT NAMED
delta-Endotoxin (insecticide), N-terminal domain
unkn...
Endotoxin_N
214. Bacteria__Bacillus_thuringiensis_WP_098976575.1
delta-Endotoxin (insecticide), N-terminal domain
Endotoxin_N
215. Bacteria__Bacillus_thuringiensis_WP_098976285.1
delta-Endotoxin (insecticide), N-terminal domain
216. Bacteria__Bacillus_thuringiensis_WP_098975546.1
N.
Trans...
Cytoplasmic domain
FAMILY NOT NAMED
delta-Endotoxin (insecticide), N-terminal domain
Endotoxin_N
unkn...
217. Bacteria__Bacillus_cereus_WP_098944547.1
delta-Endotoxin (insecticide), N-terminal domain
FAMILY NOT NAMED
Endotoxin_N
218. Bacteria__Bacillus_anthracis_WP_098926211.1
C..
Tran...
Non cytoplasmic domain
T...
Cytoplasmic domain
delta-Endotoxin (insecticide), N-terminal domain
FAMILY NOT NAMED
unkn...
Endotoxin_N
219. Bacteria__Bacillus_anthracis_WP_098925979.1
delta-Endotoxin (insecticide), N-terminal domain
Endotoxin_N

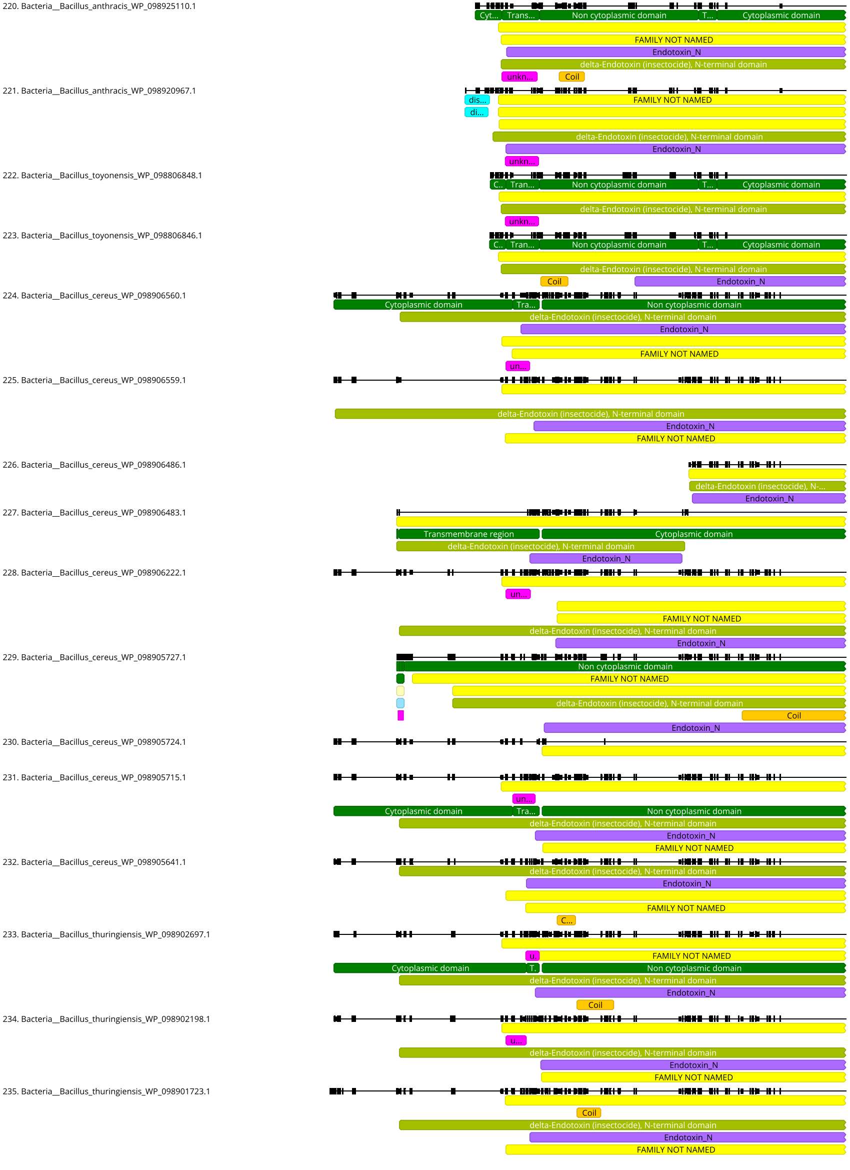
220. Bacteria__Bacillus_anthracis_WP_098925110.1
Cyt... Trans... Non cytoplasmic domain T... Cytoplasmic domain
FAMILY NOT NAMED
Endotoxin_N
delta-Endotoxin (insectocide), N-terminal domain
unkn... Coil
221. Bacteria__Bacillus_anthracis_WP_098920967.1
dis... FAMILY NOT NAMED
di...
delta-Endotoxin (insectocide), N-terminal domain
Endotoxin_N
unkn...
222. Bacteria__Bacillus_toyonensis_WP_098806848.1
C.. Tran... Non cytoplasmic domain T... Cytoplasmic domain
delta-Endotoxin (insectocide), N-terminal domain
unkn...
223. Bacteria__Bacillus_toyonensis_WP_098806846.1
C.. Tran... Non cytoplasmic domain T... Cytoplasmic domain
delta-Endotoxin (insectocide), N-terminal domain
Coil Endotoxin_N
224. Bacteria__Bacillus_cereus_WP_098906560.1
Cytoplasmic domain Tra... Non cytoplasmic domain
delta-Endotoxin (insectocide), N-terminal domain
Endotoxin_N
FAMILY NOT NAMED
un...
225. Bacteria__Bacillus_cereus_WP_098906559.1
delta-Endotoxin (insectocide), N-terminal domain
Endotoxin_N
FAMILY NOT NAMED
226. Bacteria__Bacillus_cereus_WP_098906486.1
delta-Endotoxin (insectocide), N-...
Endotoxin_N
227. Bacteria__Bacillus_cereus_WP_098906483.1
Transmembrane region Cytoplasmic domain
delta-Endotoxin (insectocide), N-terminal domain
Endotoxin_N
228. Bacteria__Bacillus_cereus_WP_098906222.1
un...
FAMILY NOT NAMED
delta-Endotoxin (insectocide), N-terminal domain
Endotoxin_N
229. Bacteria__Bacillus_cereus_WP_098905727.1
Non cytoplasmic domain
FAMILY NOT NAMED
delta-Endotoxin (insectocide), N-terminal domain
Coil
Endotoxin_N
230. Bacteria__Bacillus_cereus_WP_098905724.1
231. Bacteria__Bacillus_cereus_WP_098905715.1
un...
Cytoplasmic domain Tra... Non cytoplasmic domain
delta-Endotoxin (insectocide), N-terminal domain
Endotoxin_N
FAMILY NOT NAMED
232. Bacteria__Bacillus_cereus_WP_098905641.1
delta-Endotoxin (insectocide), N-terminal domain
Endotoxin_N
FAMILY NOT NAMED
C...
233. Bacteria__Bacillus_thuringiensis_WP_098902697.1
u. FAMILY NOT NAMED
Cytoplasmic domain T. Non cytoplasmic domain
delta-Endotoxin (insectocide), N-terminal domain
Endotoxin_N
Coil
234. Bacteria__Bacillus_thuringiensis_WP_098902198.1
u...
delta-Endotoxin (insectocide), N-terminal domain
Endotoxin_N
FAMILY NOT NAMED
235. Bacteria__Bacillus_thuringiensis_WP_098901723.1
Coil
delta-Endotoxin (insectocide), N-terminal domain
Endotoxin_N
FAMILY NOT NAMED

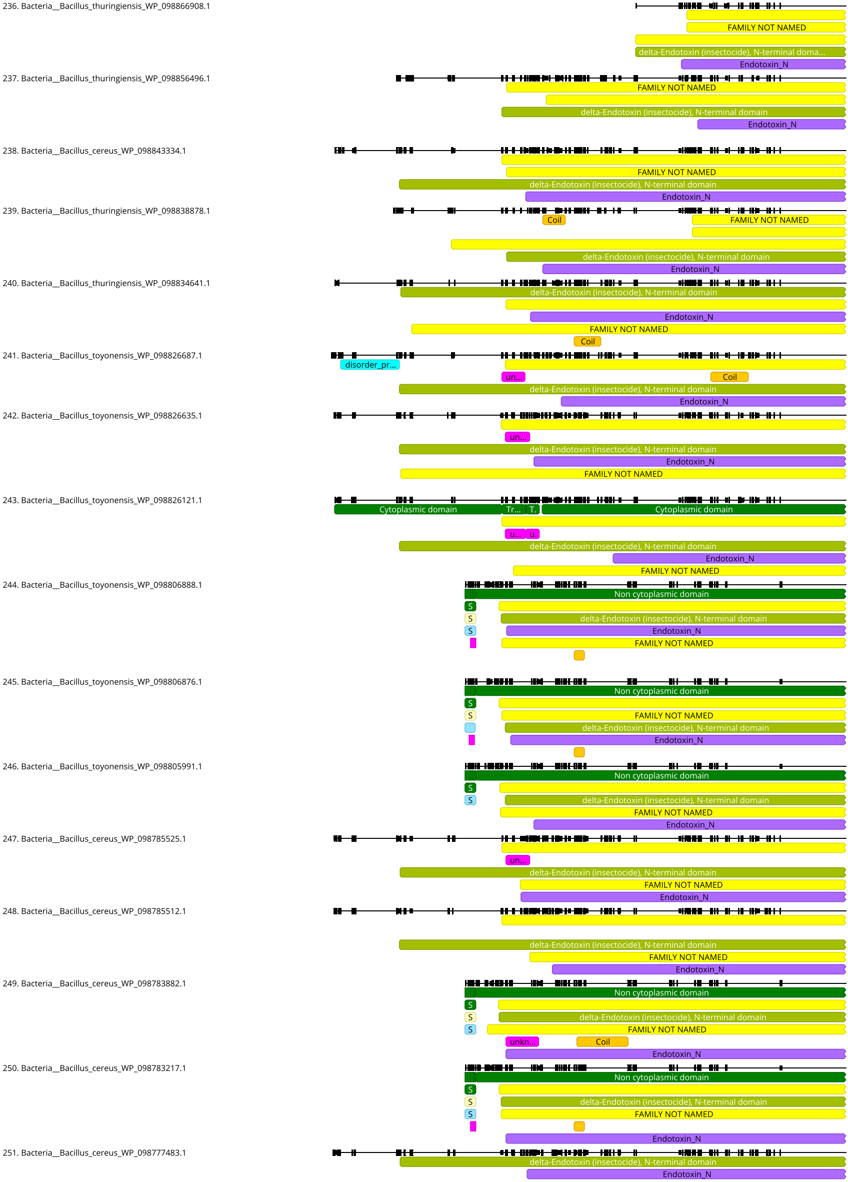
236. Bacteria__Bacillus_thuringiensis_WP_098866908.1
FAMILY NOT NAMED
delta-Endotoxin (insectocide), N-terminal doma...
Endotoxin_N
237. Bacteria__Bacillus_thuringiensis_WP_098856496.1
FAMILY NOT NAMED
delta-Endotoxin (insectocide), N-terminal domain
Endotoxin_N
238. Bacteria__Bacillus_cereus_WP_098843334.1
FAMILY NOT NAMED
delta-Endotoxin (insectocide), N-terminal domain
Endotoxin_N
239. Bacteria__Bacillus_thuringiensis_WP_098838878.1
Coil
FAMILY NOT NAMED
delta-Endotoxin (insectocide), N-terminal domain
Endotoxin_N
240. Bacteria__Bacillus_thuringiensis_WP_098834641.1
delta-Endotoxin (insectocide), N-terminal domain
Endotoxin_N
FAMILY NOT NAMED
Coil
241. Bacteria__Bacillus_toyonensis_WP_098826687.1
disorder_pr...
un...
Coil
delta-Endotoxin (insectocide), N-terminal domain
Endotoxin_N
242. Bacteria__Bacillus_toyonensis_WP_098826635.1
un...
delta-Endotoxin (insectocide), N-terminal domain
Endotoxin_N
FAMILY NOT NAMED
243. Bacteria__Bacillus_toyonensis_WP_098826121.1
Cytoplasmic domain
Tr...
T.
Cytoplasmic domain
u...
u.
delta-Endotoxin (insectocide), N-terminal domain
Endotoxin_N
FAMILY NOT NAMED
244. Bacteria__Bacillus_toyonensis_WP_098806888.1
Non cytoplasmic domain
S
S
delta-Endotoxin (insectocide), N-terminal domain
S
Endotoxin_N
FAMILY NOT NAMED
245. Bacteria__Bacillus_toyonensis_WP_098806876.1
Non cytoplasmic domain
S
S
FAMILY NOT NAMED
delta-Endotoxin (insectocide), N-terminal domain
Endotoxin_N
246. Bacteria__Bacillus_toyonensis_WP_098805991.1
Non cytoplasmic domain
S
S
delta-Endotoxin (insectocide), N-terminal domain
FAMILY NOT NAMED
Endotoxin_N
247. Bacteria__Bacillus_cereus_WP_098785525.1
un...
delta-Endotoxin (insectocide), N-terminal domain
FAMILY NOT NAMED
Endotoxin_N
248. Bacteria__Bacillus_cereus_WP_098785512.1
delta-Endotoxin (insectocide), N-terminal domain
FAMILY NOT NAMED
Endotoxin_N
249. Bacteria__Bacillus_cereus_WP_098783882.1
Non cytoplasmic domain
S
S
delta-Endotoxin (insectocide), N-terminal domain
S
FAMILY NOT NAMED
unkn...
Coil
Endotoxin_N
250. Bacteria__Bacillus_cereus_WP_098783217.1
Non cytoplasmic domain
S
S
delta-Endotoxin (insectocide), N-terminal domain
S
FAMILY NOT NAMED
Endotoxin_N
251. Bacteria__Bacillus_cereus_WP_098777483.1
delta-Endotoxin (insectocide), N-terminal domain
Endotoxin_N

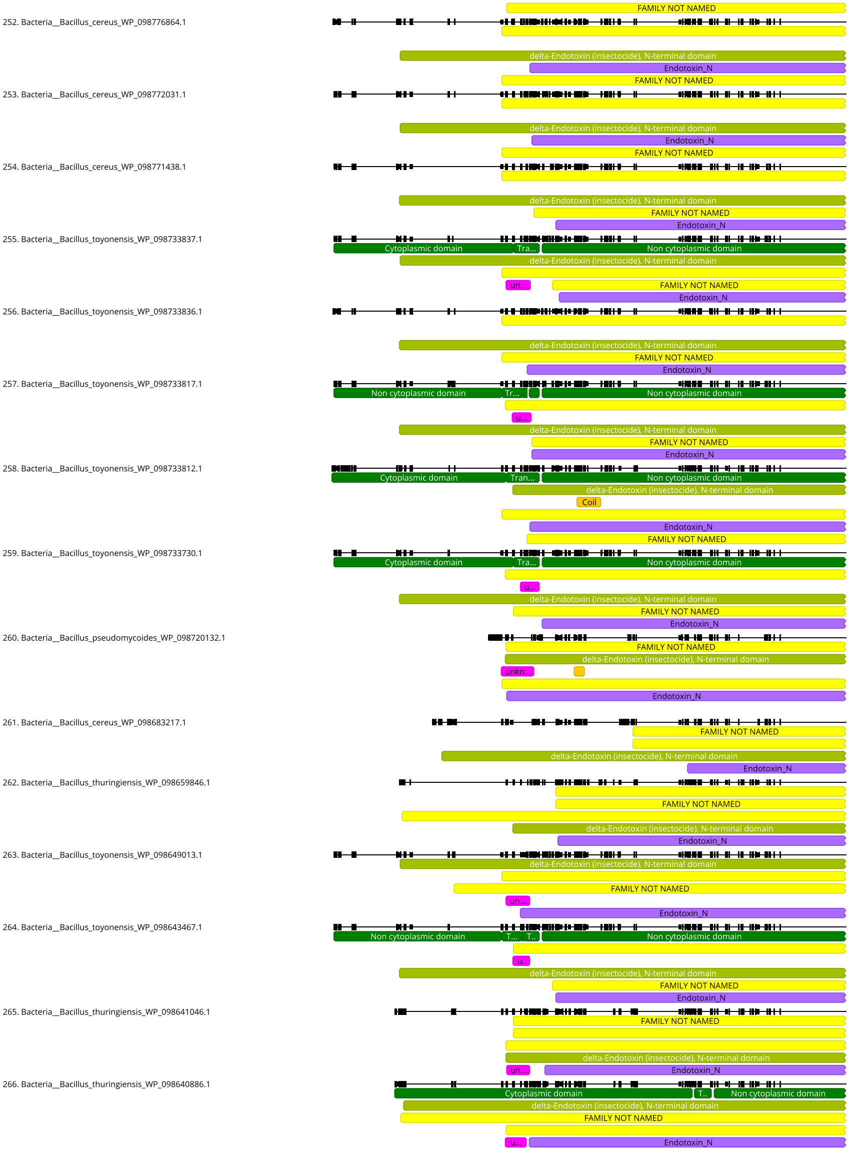
FAMILY NOT NAMED
252. Bacteria__Bacillus_cereus_WP_098776864.1
delta-Endotoxin (insectocide), N-terminal domain
Endotoxin_N
FAMILY NOT NAMED
253. Bacteria__Bacillus_cereus_WP_098772031.1
delta-Endotoxin (insectocide), N-terminal domain
Endotoxin_N
FAMILY NOT NAMED
254. Bacteria__Bacillus_cereus_WP_098771438.1
delta-Endotoxin (insectocide), N-terminal domain
FAMILY NOT NAMED
Endotoxin_N
255. Bacteria__Bacillus_toyonensis_WP_098733837.1
Cytoplasmic domain
Tra...
Non cytoplasmic domain
delta-Endotoxin (insectocide), N-terminal domain
un...
FAMILY NOT NAMED
Endotoxin_N
256. Bacteria__Bacillus_toyonensis_WP_098733836.1
delta-Endotoxin (insectocide), N-terminal domain
FAMILY NOT NAMED
Endotoxin_N
257. Bacteria__Bacillus_toyonensis_WP_098733817.1
Non cytoplasmic domain
Tr...
Non cytoplasmic domain
u...
delta-Endotoxin (insectocide), N-terminal domain
FAMILY NOT NAMED
Endotoxin_N
258. Bacteria__Bacillus_toyonensis_WP_098733812.1
Cytoplasmic domain
Tran...
Non cytoplasmic domain
delta-Endotoxin (insectocide), N-terminal domain
Coil
Endotoxin_N
FAMILY NOT NAMED
259. Bacteria__Bacillus_toyonensis_WP_098733730.1
Cytoplasmic domain
Tra...
Non cytoplasmic domain
u...
delta-Endotoxin (insectocide), N-terminal domain
FAMILY NOT NAMED
Endotoxin_N
260. Bacteria__Bacillus_pseudomycoides_WP_098720132.1
FAMILY NOT NAMED
delta-Endotoxin (insectocide), N-terminal domain
unkn...
Endotoxin_N
261. Bacteria__Bacillus_cereus_WP_098683217.1
FAMILY NOT NAMED
delta-Endotoxin (insectocide), N-terminal domain
Endotoxin_N
262. Bacteria__Bacillus_thuringiensis_WP_098659846.1
FAMILY NOT NAMED
delta-Endotoxin (insectocide), N-terminal domain
Endotoxin_N
263. Bacteria__Bacillus_toyonensis_WP_098649013.1
delta-Endotoxin (insectocide), N-terminal domain
FAMILY NOT NAMED
un...
Endotoxin_N
264. Bacteria__Bacillus_toyonensis_WP_098643467.1
Non cytoplasmic domain
T...
T..
Non cytoplasmic domain
u..
delta-Endotoxin (insectocide), N-terminal domain
FAMILY NOT NAMED
Endotoxin_N
265. Bacteria__Bacillus_thuringiensis_WP_098641046.1
FAMILY NOT NAMED
delta-Endotoxin (insectocide), N-terminal domain
un...
Endotoxin_N
266. Bacteria__Bacillus_thuringiensis_WP_098640886.1
Cytoplasmic domain
T..
Non cytoplasmic domain
delta-Endotoxin (insectocide), N-terminal domain
FAMILY NOT NAMED
u...
Endotoxin_N

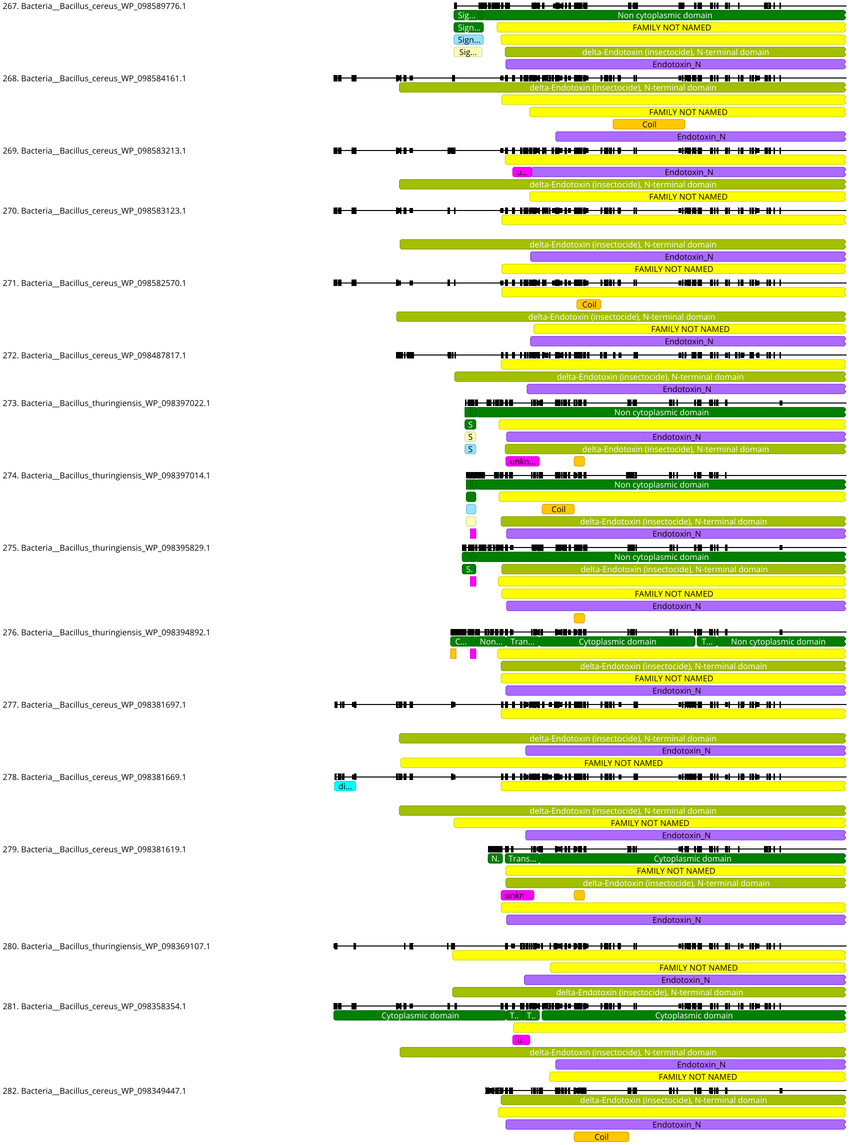
267. Bacteria__Bacillus_cereus_WP_098589776.1
Sig...
Non cytoplasmic domain
Sign...
FAMILY NOT NAMED
Sign...
Sig...
delta-Endotoxin (insectocide), N-terminal domain
Endotoxin_N
268. Bacteria__Bacillus_cereus_WP_098584161.1
delta-Endotoxin (insectocide), N-terminal domain
FAMILY NOT NAMED
Coil
Endotoxin_N
269. Bacteria__Bacillus_cereus_WP_098583213.1
u...
Endotoxin_N
delta-Endotoxin (insectocide), N-terminal domain
FAMILY NOT NAMED
270. Bacteria__Bacillus_cereus_WP_098583123.1
delta-Endotoxin (insectocide), N-terminal domain
Endotoxin_N
FAMILY NOT NAMED
271. Bacteria__Bacillus_cereus_WP_098582570.1
Coil
delta-Endotoxin (insectocide), N-terminal domain
FAMILY NOT NAMED
Endotoxin_N
272. Bacteria__Bacillus_cereus_WP_098487817.1
delta-Endotoxin (insectocide), N-terminal domain
Endotoxin_N
273. Bacteria__Bacillus_thuringiensis_WP_098397022.1
Non cytoplasmic domain
S
S
Endotoxin_N
S
delta-Endotoxin (insectocide), N-terminal domain
unkn...
274. Bacteria__Bacillus_thuringiensis_WP_098397014.1
Non cytoplasmic domain
Coil
delta-Endotoxin (insectocide), N-terminal domain
Endotoxin_N
275. Bacteria__Bacillus_thuringiensis_WP_098395829.1
Non cytoplasmic domain
S.
delta-Endotoxin (insectocide), N-terminal domain
FAMILY NOT NAMED
Endotoxin_N
276. Bacteria__Bacillus_thuringiensis_WP_098394892.1
C...
Non...
Tran...
Cytoplasmic domain
T...
Non cytoplasmic domain
delta-Endotoxin (insectocide), N-terminal domain
FAMILY NOT NAMED
Endotoxin_N
277. Bacteria__Bacillus_cereus_WP_098381697.1
delta-Endotoxin (insectocide), N-terminal domain
Endotoxin_N
FAMILY NOT NAMED
278. Bacteria__Bacillus_cereus_WP_098381669.1
di...
delta-Endotoxin (insectocide), N-terminal domain
FAMILY NOT NAMED
Endotoxin_N
279. Bacteria__Bacillus_cereus_WP_098381619.1
N.
Trans...
Cytoplasmic domain
FAMILY NOT NAMED
delta-Endotoxin (insectocide), N-terminal domain
unkn...
Endotoxin_N
280. Bacteria__Bacillus_thuringiensis_WP_098369107.1
FAMILY NOT NAMED
Endotoxin_N
delta-Endotoxin (insectocide), N-terminal domain
281. Bacteria__Bacillus_cereus_WP_098358354.1
Cytoplasmic domain
T..
T..
Cytoplasmic domain
u..
delta-Endotoxin (insectocide), N-terminal domain
Endotoxin_N
FAMILY NOT NAMED
282. Bacteria__Bacillus_cereus_WP_098349447.1
delta-Endotoxin (insectocide), N-terminal domain
Endotoxin_N
Coil

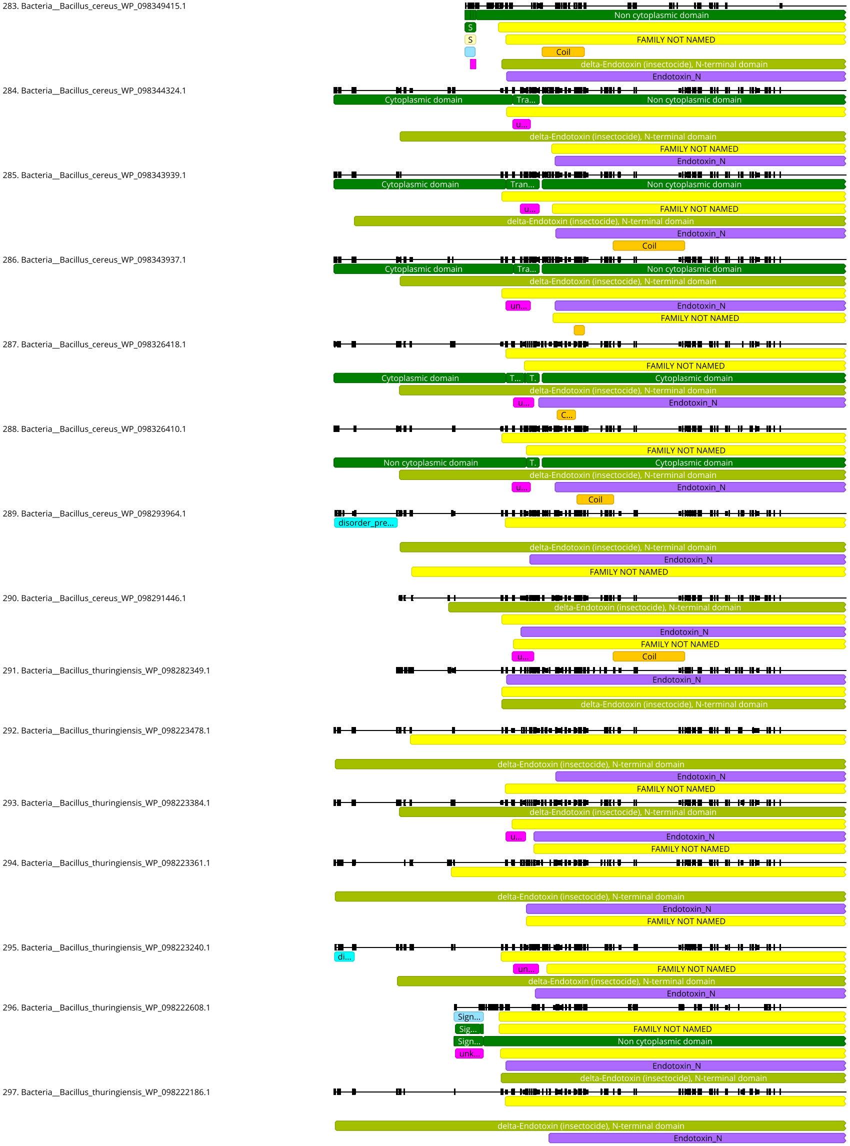
283. Bacteria__Bacillus_cereus_WP_098349415.1
Non cytoplasmic domain
S
S
FAMILY NOT NAMED
Coil
delta-Endotoxin (insecticide), N-terminal domain
Endotoxin_N
284. Bacteria__Bacillus_cereus_WP_098344324.1
Cytoplasmic domain
Tra...
Non cytoplasmic domain
u...
delta-Endotoxin (insecticide), N-terminal domain
FAMILY NOT NAMED
Endotoxin_N
285. Bacteria__Bacillus_cereus_WP_098343939.1
Cytoplasmic domain
Tran...
Non cytoplasmic domain
u...
FAMILY NOT NAMED
delta-Endotoxin (insecticide), N-terminal domain
Endotoxin_N
Coil
286. Bacteria__Bacillus_cereus_WP_098343937.1
Cytoplasmic domain
Tra...
Non cytoplasmic domain
delta-Endotoxin (insecticide), N-terminal domain
un...
Endotoxin_N
FAMILY NOT NAMED
287. Bacteria__Bacillus_cereus_WP_098326418.1
FAMILY NOT NAMED
Cytoplasmic domain
T...
T.
Cytoplasmic domain
delta-Endotoxin (insecticide), N-terminal domain
u...
Endotoxin_N
C...
288. Bacteria__Bacillus_cereus_WP_098326410.1
FAMILY NOT NAMED
Non cytoplasmic domain
T.
Cytoplasmic domain
delta-Endotoxin (insecticide), N-terminal domain
u...
Endotoxin_N
Coil
289. Bacteria__Bacillus_cereus_WP_098293964.1
disorder_pre...
delta-Endotoxin (insecticide), N-terminal domain
Endotoxin_N
FAMILY NOT NAMED
290. Bacteria__Bacillus_cereus_WP_098291446.1
delta-Endotoxin (insecticide), N-terminal domain
Endotoxin_N
FAMILY NOT NAMED
u...
Coil
291. Bacteria__Bacillus_thuringiensis_WP_098282349.1
Endotoxin_N
delta-Endotoxin (insecticide), N-terminal domain
292. Bacteria__Bacillus_thuringiensis_WP_098223478.1
delta-Endotoxin (insecticide), N-terminal domain
Endotoxin_N
FAMILY NOT NAMED
293. Bacteria__Bacillus_thuringiensis_WP_098223384.1
delta-Endotoxin (insecticide), N-terminal domain
u...
Endotoxin_N
FAMILY NOT NAMED
294. Bacteria__Bacillus_thuringiensis_WP_098223361.1
delta-Endotoxin (insecticide), N-terminal domain
Endotoxin_N
FAMILY NOT NAMED
295. Bacteria__Bacillus_thuringiensis_WP_098223240.1
di...
un...
FAMILY NOT NAMED
delta-Endotoxin (insecticide), N-terminal domain
Endotoxin_N
296. Bacteria__Bacillus_thuringiensis_WP_098222608.1
Sign...
Sig...
FAMILY NOT NAMED
Sign...
Non cytoplasmic domain
unk...
Endotoxin_N
delta-Endotoxin (insecticide), N-terminal domain
297. Bacteria__Bacillus_thuringiensis_WP_098222186.1
delta-Endotoxin (insecticide), N-terminal domain
Endotoxin_N

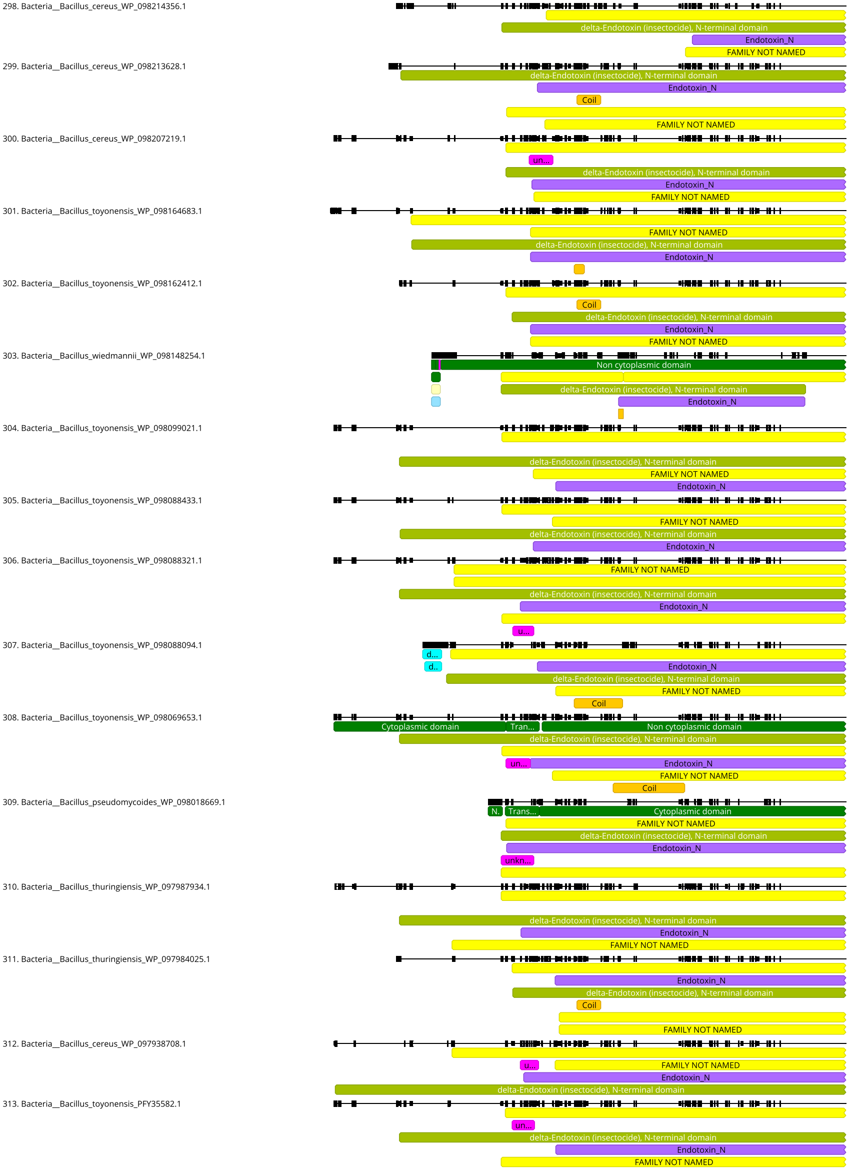
298. Bacteria__Bacillus_cereus_WP_098214356.1
delta-Endotoxin (insectocide), N-terminal domain
Endotoxin_N
FAMILY NOT NAMED
299. Bacteria__Bacillus_cereus_WP_098213628.1
delta-Endotoxin (insectocide), N-terminal domain
Endotoxin_N
Coil
FAMILY NOT NAMED
300. Bacteria__Bacillus_cereus_WP_098207219.1
un...
delta-Endotoxin (insectocide), N-terminal domain
Endotoxin_N
FAMILY NOT NAMED
301. Bacteria__Bacillus_toyonensis_WP_098164683.1
FAMILY NOT NAMED
delta-Endotoxin (insectocide), N-terminal domain
Endotoxin_N
302. Bacteria__Bacillus_toyonensis_WP_098162412.1
Coil
delta-Endotoxin (insectocide), N-terminal domain
Endotoxin_N
FAMILY NOT NAMED
303. Bacteria__Bacillus_wiedmannii_WP_098148254.1
Non cytoplasmic domain
delta-Endotoxin (insectocide), N-terminal domain
Endotoxin_N
304. Bacteria__Bacillus_toyonensis_WP_098099021.1
delta-Endotoxin (insectocide), N-terminal domain
FAMILY NOT NAMED
Endotoxin_N
305. Bacteria__Bacillus_toyonensis_WP_098088433.1
FAMILY NOT NAMED
delta-Endotoxin (insectocide), N-terminal domain
Endotoxin_N
306. Bacteria__Bacillus_toyonensis_WP_098088321.1
FAMILY NOT NAMED
delta-Endotoxin (insectocide), N-terminal domain
Endotoxin_N
u...
307. Bacteria__Bacillus_toyonensis_WP_098088094.1
d...
d..
Endotoxin_N
delta-Endotoxin (insectocide), N-terminal domain
FAMILY NOT NAMED
Coil
308. Bacteria__Bacillus_toyonensis_WP_098069653.1
Cytoplasmic domain
Tran...
Non cytoplasmic domain
delta-Endotoxin (insectocide), N-terminal domain
un...
Endotoxin_N
FAMILY NOT NAMED
Coil
309. Bacteria__Bacillus_pseudomycoides_WP_098018669.1
N.
Trans...
Cytoplasmic domain
FAMILY NOT NAMED
delta-Endotoxin (insectocide), N-terminal domain
Endotoxin_N
unkn...
310. Bacteria__Bacillus_thuringiensis_WP_097987934.1
delta-Endotoxin (insectocide), N-terminal domain
Endotoxin_N
FAMILY NOT NAMED
311. Bacteria__Bacillus_thuringiensis_WP_097984025.1
Endotoxin_N
delta-Endotoxin (insectocide), N-terminal domain
Coil
FAMILY NOT NAMED
312. Bacteria__Bacillus_cereus_WP_097938708.1
u...
FAMILY NOT NAMED
Endotoxin_N
delta-Endotoxin (insectocide), N-terminal domain
313. Bacteria__Bacillus_toyonensis_PFY35582.1
un...
delta-Endotoxin (insectocide), N-terminal domain
Endotoxin_N
FAMILY NOT NAMED

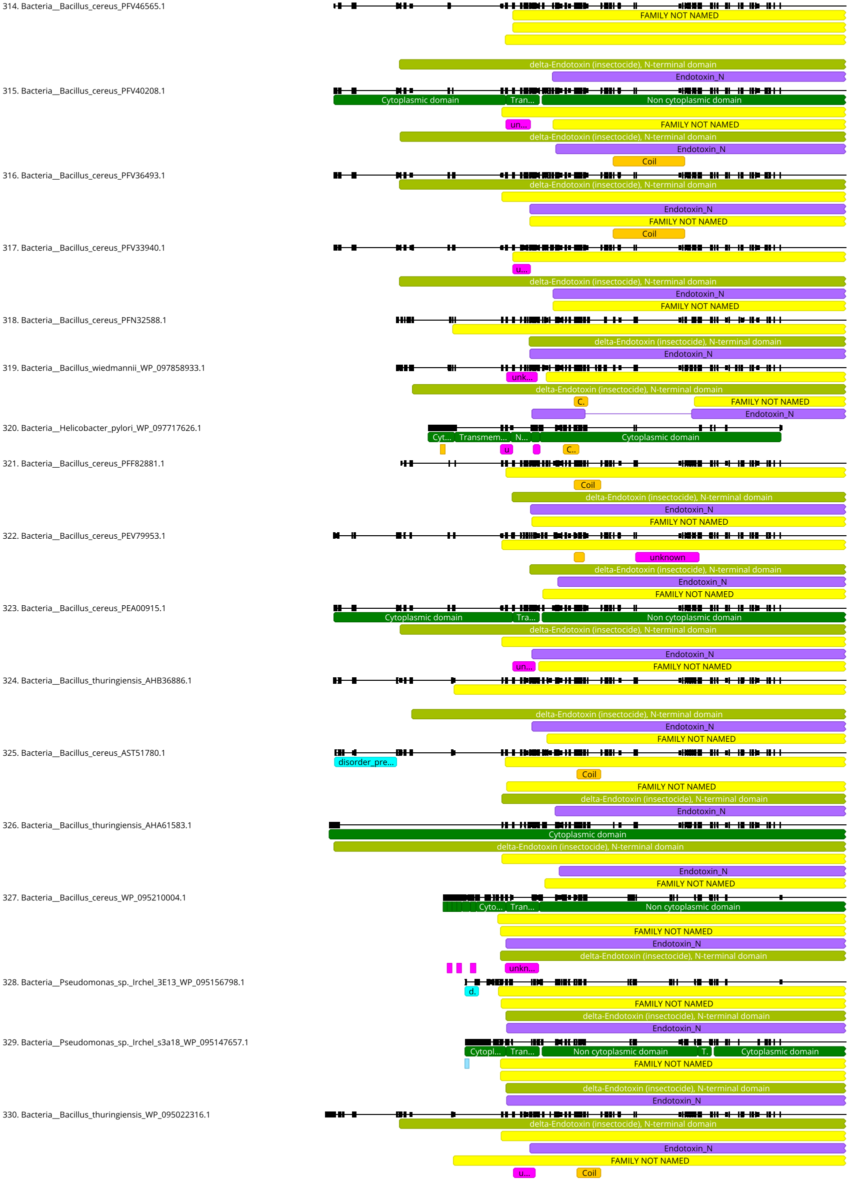
314. Bacteria__Bacillus_cereus_PFV46565.1
FAMILY NOT NAMED
delta-Endotoxin (insectocide), N-terminal domain
Endotoxin_N
315. Bacteria__Bacillus_cereus_PFV40208.1
Cytoplasmic domain
Tran...
Non cytoplasmic domain
un...
FAMILY NOT NAMED
delta-Endotoxin (insectocide), N-terminal domain
Endotoxin_N
Coil
316. Bacteria__Bacillus_cereus_PFV36493.1
delta-Endotoxin (insectocide), N-terminal domain
Endotoxin_N
FAMILY NOT NAMED
Coil
317. Bacteria__Bacillus_cereus_PFV33940.1
u...
delta-Endotoxin (insectocide), N-terminal domain
Endotoxin_N
FAMILY NOT NAMED
318. Bacteria__Bacillus_cereus_PFN32588.1
delta-Endotoxin (insectocide), N-terminal domain
Endotoxin_N
319. Bacteria__Bacillus_wiedmannii_WP_097858933.1
unk...
delta-Endotoxin (insectocide), N-terminal domain
C.
FAMILY NOT NAMED
Endotoxin_N
320. Bacteria__Helicobacter_pylori_WP_097717626.1
Cyt...
Transmem...
N...
Cytoplasmic domain
u
C..
321. Bacteria__Bacillus_cereus_PFF82881.1
Coil
delta-Endotoxin (insectocide), N-terminal domain
Endotoxin_N
FAMILY NOT NAMED
322. Bacteria__Bacillus_cereus_PEV79953.1
unknown
delta-Endotoxin (insectocide), N-terminal domain
Endotoxin_N
FAMILY NOT NAMED
323. Bacteria__Bacillus_cereus_PEA00915.1
Cytoplasmic domain
Tra...
Non cytoplasmic domain
delta-Endotoxin (insectocide), N-terminal domain
Endotoxin_N
un...
FAMILY NOT NAMED
324. Bacteria__Bacillus_thuringiensis_AHB36886.1
delta-Endotoxin (insectocide), N-terminal domain
Endotoxin_N
FAMILY NOT NAMED
325. Bacteria__Bacillus_cereus_AST51780.1
disorder_pre...
Coil
FAMILY NOT NAMED
delta-Endotoxin (insectocide), N-terminal domain
Endotoxin_N
326. Bacteria__Bacillus_thuringiensis_AHA61583.1
Cytoplasmic domain
delta-Endotoxin (insectocide), N-terminal domain
Endotoxin_N
FAMILY NOT NAMED
327. Bacteria__Bacillus_cereus_WP_095210004.1
Cyto...
Tran...
Non cytoplasmic domain
FAMILY NOT NAMED
Endotoxin_N
delta-Endotoxin (insectocide), N-terminal domain
unkn...
328. Bacteria__Pseudomonas_sp._Irchel_3E13_WP_095156798.1
d.
FAMILY NOT NAMED
delta-Endotoxin (insectocide), N-terminal domain
Endotoxin_N
329. Bacteria__Pseudomonas_sp._Irchel_s3a18_WP_095147657.1
Cytopl...
Tran...
Non cytoplasmic domain
T.
Cytoplasmic domain
FAMILY NOT NAMED
delta-Endotoxin (insectocide), N-terminal domain
Endotoxin_N
330. Bacteria__Bacillus_thuringiensis_WP_095022316.1
delta-Endotoxin (insectocide), N-terminal domain
Endotoxin_N
FAMILY NOT NAMED
u...
Coil

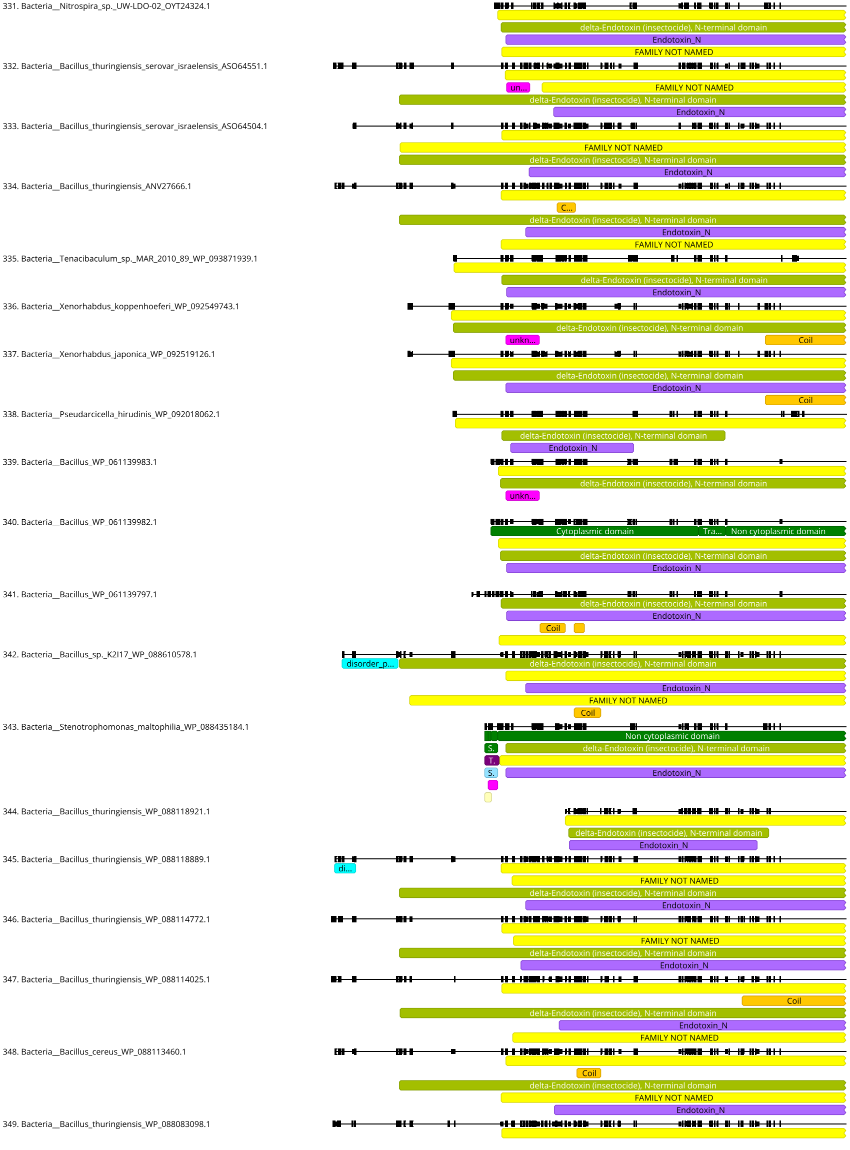
331. Bacteria__Nitrospira_sp._UW-LDO-02_OYT24324.1
delta-Endotoxin (insectocide), N-terminal domain
Endotoxin_N
FAMILY NOT NAMED
332. Bacteria__Bacillus_thuringiensis_serovar_israelensis_ASO64551.1
un...
FAMILY NOT NAMED
delta-Endotoxin (insectocide), N-terminal domain
Endotoxin_N
333. Bacteria__Bacillus_thuringiensis_serovar_israelensis_ASO64504.1
FAMILY NOT NAMED
delta-Endotoxin (insectocide), N-terminal domain
Endotoxin_N
334. Bacteria__Bacillus_thuringiensis_ANV27666.1
C...
delta-Endotoxin (insectocide), N-terminal domain
Endotoxin_N
FAMILY NOT NAMED
335. Bacteria__Tenacibaculum_sp._MAR_2010_89_WP_093871939.1
delta-Endotoxin (insectocide), N-terminal domain
Endotoxin_N
336. Bacteria__Xenorhabdus_koppenhoeferi_WP_092549743.1
delta-Endotoxin (insectocide), N-terminal domain
unkn...
Coil
337. Bacteria__Xenorhabdus_japonica_WP_092519126.1
delta-Endotoxin (insectocide), N-terminal domain
Endotoxin_N
Coil
338. Bacteria__Pseudarcicella_hirudinis_WP_092018062.1
delta-Endotoxin (insectocide), N-terminal domain
Endotoxin_N
339. Bacteria__Bacillus_WP_061139983.1
delta-Endotoxin (insectocide), N-terminal domain
unkn...
340. Bacteria__Bacillus_WP_061139982.1
Cytoplasmic domain
Tra...
Non cytoplasmic domain
delta-Endotoxin (insectocide), N-terminal domain
Endotoxin_N
341. Bacteria__Bacillus_WP_061139797.1
delta-Endotoxin (insectocide), N-terminal domain
Endotoxin_N
Coil
342. Bacteria__Bacillus_sp._K2I17_WP_088610578.1
disorder_p...
delta-Endotoxin (insectocide), N-terminal domain
Endotoxin_N
FAMILY NOT NAMED
Coil
343. Bacteria__Stenotrophomonas_maltophilia_WP_088435184.1
Non cytoplasmic domain
S.
delta-Endotoxin (insectocide), N-terminal domain
T.
S.
Endotoxin_N
344. Bacteria__Bacillus_thuringiensis_WP_088118921.1
delta-Endotoxin (insectocide), N-terminal domain
Endotoxin_N
345. Bacteria__Bacillus_thuringiensis_WP_088118889.1
di...
FAMILY NOT NAMED
delta-Endotoxin (insectocide), N-terminal domain
Endotoxin_N
346. Bacteria__Bacillus_thuringiensis_WP_088114772.1
FAMILY NOT NAMED
delta-Endotoxin (insectocide), N-terminal domain
Endotoxin_N
347. Bacteria__Bacillus_thuringiensis_WP_088114025.1
Coil
delta-Endotoxin (insectocide), N-terminal domain
Endotoxin_N
FAMILY NOT NAMED
348. Bacteria__Bacillus_cereus_WP_088113460.1
Coil
delta-Endotoxin (insectocide), N-terminal domain
FAMILY NOT NAMED
Endotoxin_N
349. Bacteria__Bacillus_thuringiensis_WP_088083098.1

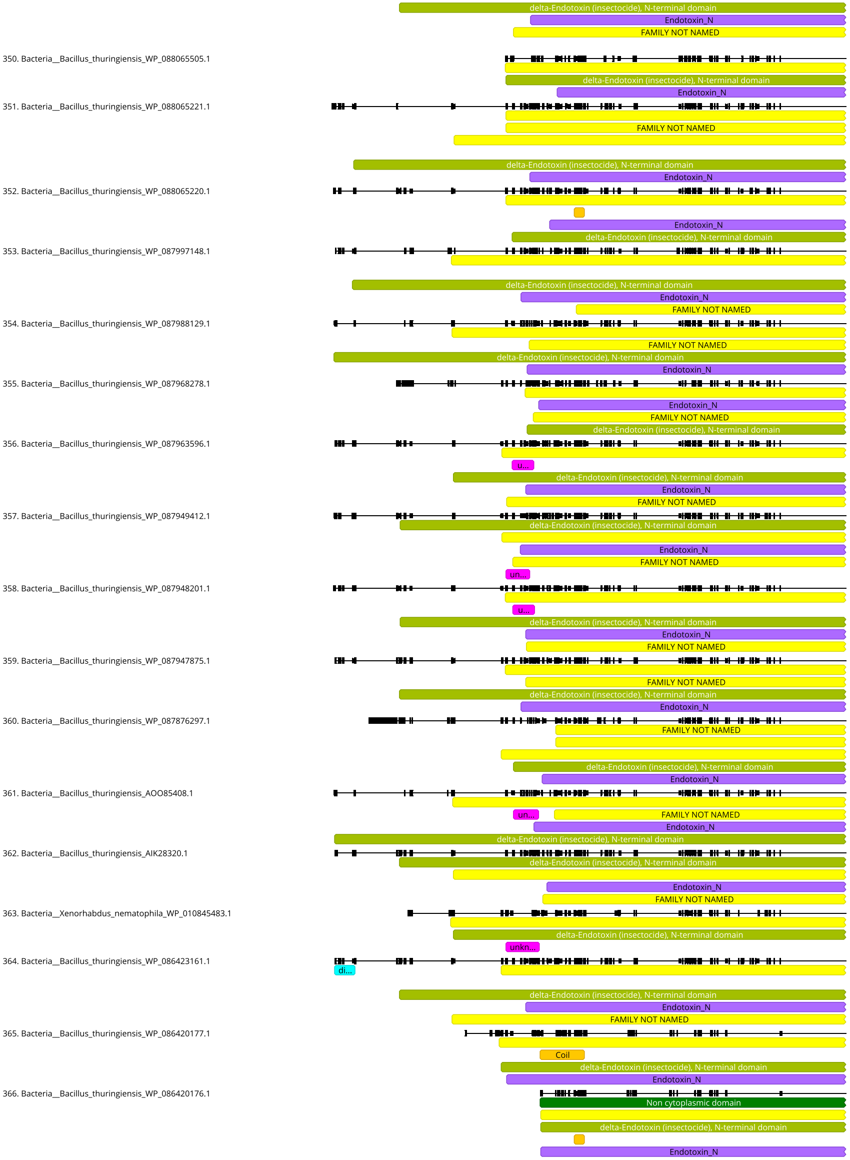

delta-Endotoxin (insectocide), N-terminal domain
Endotoxin_N
FAMILY NOT NAMED
350. Bacteria__Bacillus_thuringiensis_WP_088065505.1
delta-Endotoxin (insectocide), N-terminal domain
Endotoxin_N
351. Bacteria__Bacillus_thuringiensis_WP_088065221.1
FAMILY NOT NAMED
delta-Endotoxin (insectocide), N-terminal domain
Endotoxin_N
352. Bacteria__Bacillus_thuringiensis_WP_088065220.1
Endotoxin_N
delta-Endotoxin (insectocide), N-terminal domain
353. Bacteria__Bacillus_thuringiensis_WP_087997148.1
delta-Endotoxin (insectocide), N-terminal domain
Endotoxin_N
FAMILY NOT NAMED
354. Bacteria__Bacillus_thuringiensis_WP_087988129.1
FAMILY NOT NAMED
delta-Endotoxin (insectocide), N-terminal domain
Endotoxin_N
355. Bacteria__Bacillus_thuringiensis_WP_087968278.1
Endotoxin_N
FAMILY NOT NAMED
delta-Endotoxin (insectocide), N-terminal domain
356. Bacteria__Bacillus_thuringiensis_WP_087963596.1
u...
delta-Endotoxin (insectocide), N-terminal domain
Endotoxin_N
FAMILY NOT NAMED
357. Bacteria__Bacillus_thuringiensis_WP_087949412.1
delta-Endotoxin (insectocide), N-terminal domain
Endotoxin_N
FAMILY NOT NAMED
un...
358. Bacteria__Bacillus_thuringiensis_WP_087948201.1
u...
delta-Endotoxin (insectocide), N-terminal domain
Endotoxin_N
FAMILY NOT NAMED
359. Bacteria__Bacillus_thuringiensis_WP_087947875.1
FAMILY NOT NAMED
delta-Endotoxin (insectocide), N-terminal domain
Endotoxin_N
360. Bacteria__Bacillus_thuringiensis_WP_087876297.1
FAMILY NOT NAMED
delta-Endotoxin (insectocide), N-terminal domain
Endotoxin_N
361. Bacteria__Bacillus_thuringiensis_AOO85408.1
un...
FAMILY NOT NAMED
Endotoxin_N
delta-Endotoxin (insectocide), N-terminal domain
362. Bacteria__Bacillus_thuringiensis_AIK28320.1
delta-Endotoxin (insectocide), N-terminal domain
Endotoxin_N
FAMILY NOT NAMED
363. Bacteria__Xenorhabdus_nematophila_WP_010845483.1
delta-Endotoxin (insectocide), N-terminal domain
unkn...
364. Bacteria__Bacillus_thuringiensis_WP_086423161.1
di...
delta-Endotoxin (insectocide), N-terminal domain
Endotoxin_N
FAMILY NOT NAMED
365. Bacteria__Bacillus_thuringiensis_WP_086420177.1
Coil
delta-Endotoxin (insectocide), N-terminal domain
Endotoxin_N
366. Bacteria__Bacillus_thuringiensis_WP_086420176.1
Non cytoplasmic domain
delta-Endotoxin (insectocide), N-terminal domain
Endotoxin_N

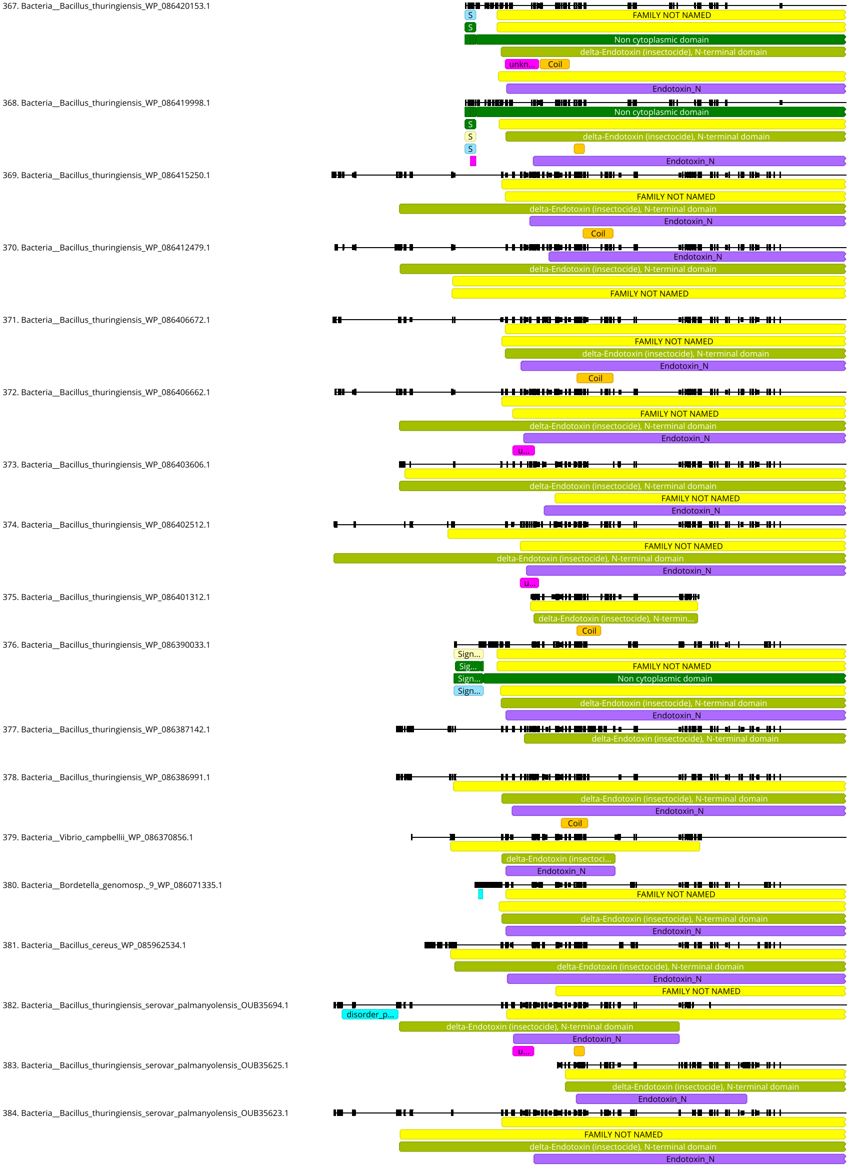
367. Bacteria__Bacillus_thuringiensis_WP_086420153.1
S
FAMILY NOT NAMED
S
Non cytoplasmic domain
delta-Endotoxin (insectocide), N-terminal domain
unkn...
Coil
Endotoxin_N
368. Bacteria__Bacillus_thuringiensis_WP_086419998.1
Non cytoplasmic domain
S
S
delta-Endotoxin (insectocide), N-terminal domain
S
Endotoxin_N
369. Bacteria__Bacillus_thuringiensis_WP_086415250.1
FAMILY NOT NAMED
delta-Endotoxin (insectocide), N-terminal domain
Endotoxin_N
Coil
370. Bacteria__Bacillus_thuringiensis_WP_086412479.1
Endotoxin_N
delta-Endotoxin (insectocide), N-terminal domain
FAMILY NOT NAMED
371. Bacteria__Bacillus_thuringiensis_WP_086406672.1
FAMILY NOT NAMED
delta-Endotoxin (insectocide), N-terminal domain
Endotoxin_N
Coil
372. Bacteria__Bacillus_thuringiensis_WP_086406662.1
FAMILY NOT NAMED
delta-Endotoxin (insectocide), N-terminal domain
Endotoxin_N
u...
373. Bacteria__Bacillus_thuringiensis_WP_086403606.1
delta-Endotoxin (insectocide), N-terminal domain
FAMILY NOT NAMED
Endotoxin_N
374. Bacteria__Bacillus_thuringiensis_WP_086402512.1
FAMILY NOT NAMED
delta-Endotoxin (insectocide), N-terminal domain
Endotoxin_N
u...
375. Bacteria__Bacillus_thuringiensis_WP_086401312.1
delta-Endotoxin (insectocide), N-termin...
Coil
376. Bacteria__Bacillus_thuringiensis_WP_086390033.1
Sign...
Sig...
FAMILY NOT NAMED
Sign...
Non cytoplasmic domain
Sign...
delta-Endotoxin (insectocide), N-terminal domain
Endotoxin_N
377. Bacteria__Bacillus_thuringiensis_WP_086387142.1
delta-Endotoxin (insectocide), N-terminal domain
378. Bacteria__Bacillus_thuringiensis_WP_086386991.1
delta-Endotoxin (insectocide), N-terminal domain
Endotoxin_N
Coil
379. Bacteria__Vibrio_campbellii_WP_086370856.1
delta-Endotoxin (insectoci...
Endotoxin_N
380. Bacteria__Bordetella_genomosp._9_WP_086071335.1
FAMILY NOT NAMED
delta-Endotoxin (insectocide), N-terminal domain
Endotoxin_N
381. Bacteria__Bacillus_cereus_WP_085962534.1
delta-Endotoxin (insectocide), N-terminal domain
Endotoxin_N
FAMILY NOT NAMED
382. Bacteria__Bacillus_thuringiensis_serovar_palmanyolensis_OUB35694.1
disorder_p...
delta-Endotoxin (insectocide), N-terminal domain
Endotoxin_N
u...
383. Bacteria__Bacillus_thuringiensis_serovar_palmanyolensis_OUB35625.1
delta-Endotoxin (insectocide), N-terminal domain
Endotoxin_N
384. Bacteria__Bacillus_thuringiensis_serovar_palmanyolensis_OUB35623.1
FAMILY NOT NAMED
delta-Endotoxin (insectocide), N-terminal domain
Endotoxin_N

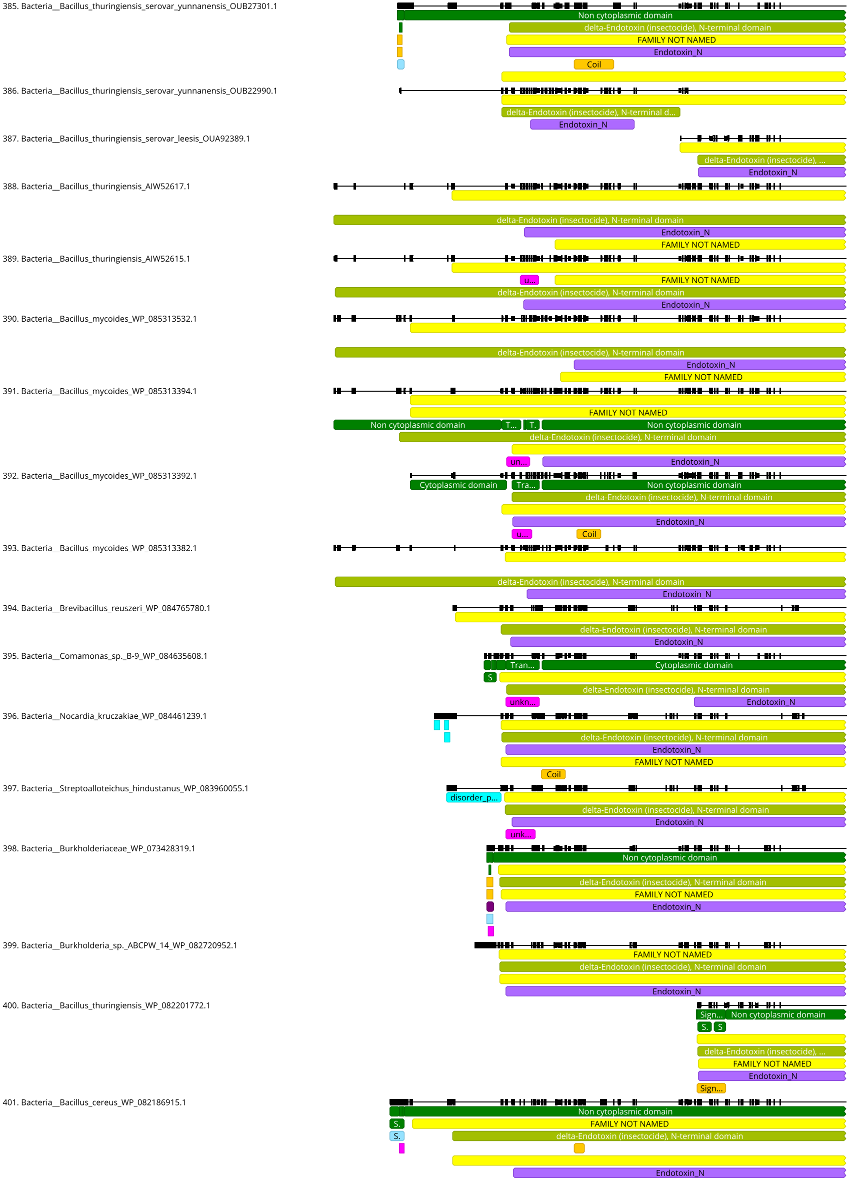
385. Bacteria__Bacillus_thuringiensis_serovar_yunnanensis_OUB27301.1
Non cytoplasmic domain
delta-Endotoxin (insectocide), N-terminal domain
FAMILY NOT NAMED
Endotoxin_N
Coil
386. Bacteria__Bacillus_thuringiensis_serovar_yunnanensis_OUB22990.1
delta-Endotoxin (insectocide), N-terminal d...
Endotoxin_N
387. Bacteria__Bacillus_thuringiensis_serovar_leesis_OUA92389.1
delta-Endotoxin (insectocide), ...
Endotoxin_N
388. Bacteria__Bacillus_thuringiensis_AIW52617.1
delta-Endotoxin (insectocide), N-terminal domain
Endotoxin_N
FAMILY NOT NAMED
389. Bacteria__Bacillus_thuringiensis_AIW52615.1
u...
FAMILY NOT NAMED
delta-Endotoxin (insectocide), N-terminal domain
Endotoxin_N
390. Bacteria__Bacillus_mycoides_WP_085313532.1
delta-Endotoxin (insectocide), N-terminal domain
Endotoxin_N
FAMILY NOT NAMED
391. Bacteria__Bacillus_mycoides_WP_085313394.1
FAMILY NOT NAMED
Non cytoplasmic domain
T...
T.
Non cytoplasmic domain
delta-Endotoxin (insectocide), N-terminal domain
un...
Endotoxin_N
392. Bacteria__Bacillus_mycoides_WP_085313392.1
Cytoplasmic domain
Tra...
Non cytoplasmic domain
delta-Endotoxin (insectocide), N-terminal domain
Endotoxin_N
u...
Coil
393. Bacteria__Bacillus_mycoides_WP_085313382.1
delta-Endotoxin (insectocide), N-terminal domain
Endotoxin_N
394. Bacteria__Brevibacillus_reuszeri_WP_084765780.1
delta-Endotoxin (insectocide), N-terminal domain
Endotoxin_N
395. Bacteria__Comamonas_sp._B-9_WP_084635608.1
Tran...
Cytoplasmic domain
S
delta-Endotoxin (insectocide), N-terminal domain
unkn...
Endotoxin_N
396. Bacteria__Nocardia_kruczakiae_WP_084461239.1
delta-Endotoxin (insectocide), N-terminal domain
Endotoxin_N
FAMILY NOT NAMED
Coil
397. Bacteria__Streptoalloteichus_hindustanus_WP_083960055.1
disorder_p...
delta-Endotoxin (insectocide), N-terminal domain
Endotoxin_N
unk...
398. Bacteria__Burkholderiaceae_WP_073428319.1
Non cytoplasmic domain
delta-Endotoxin (insectocide), N-terminal domain
FAMILY NOT NAMED
Endotoxin_N
399. Bacteria__Burkholderia_sp._ABCPW_14_WP_082720952.1
FAMILY NOT NAMED
delta-Endotoxin (insectocide), N-terminal domain
Endotoxin_N
400. Bacteria__Bacillus_thuringiensis_WP_082201772.1
Sign...
Non cytoplasmic domain
S.
S
delta-Endotoxin (insectocide), ...
FAMILY NOT NAMED
Endotoxin_N
Sign...
401. Bacteria__Bacillus_cereus_WP_082186915.1
Non cytoplasmic domain
S.
FAMILY NOT NAMED
S.
delta-Endotoxin (insectocide), N-terminal domain
Endotoxin_N

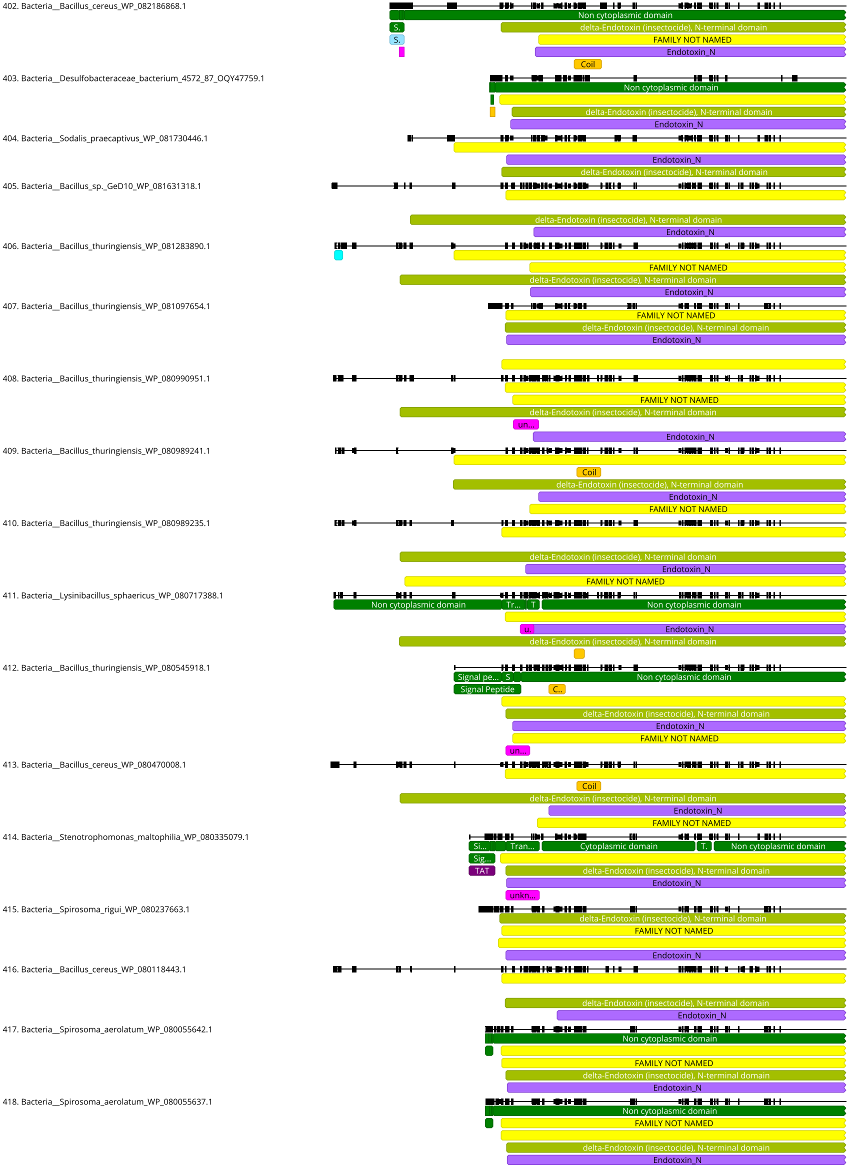

402. Bacteria__Bacillus_cereus_WP_082186868.1
Non cytoplasmic domain
S.
delta-Endotoxin (insectocide), N-terminal domain
S.
FAMILY NOT NAMED
Endotoxin_N
Coil
403. Bacteria__Desulfobacteraceae_bacterium_4572_87_OQY47759.1
Non cytoplasmic domain
delta-Endotoxin (insectocide), N-terminal domain
Endotoxin_N
404. Bacteria__Sodalis_praecaptivus_WP_081730446.1
Endotoxin_N
delta-Endotoxin (insectocide), N-terminal domain
405. Bacteria__Bacillus_sp._GeD10_WP_081631318.1
delta-Endotoxin (insectocide), N-terminal domain
Endotoxin_N
406. Bacteria__Bacillus_thuringiensis_WP_081283890.1
FAMILY NOT NAMED
delta-Endotoxin (insectocide), N-terminal domain
Endotoxin_N
407. Bacteria__Bacillus_thuringiensis_WP_081097654.1
FAMILY NOT NAMED
delta-Endotoxin (insectocide), N-terminal domain
Endotoxin_N
408. Bacteria__Bacillus_thuringiensis_WP_080990951.1
FAMILY NOT NAMED
delta-Endotoxin (insectocide), N-terminal domain
un...
Endotoxin_N
409. Bacteria__Bacillus_thuringiensis_WP_080989241.1
Coil
delta-Endotoxin (insectocide), N-terminal domain
Endotoxin_N
FAMILY NOT NAMED
410. Bacteria__Bacillus_thuringiensis_WP_080989235.1
delta-Endotoxin (insectocide), N-terminal domain
Endotoxin_N
FAMILY NOT NAMED
411. Bacteria__Lysinibacillus_sphaericus_WP_080717388.1
Non cytoplasmic domain
Tr...
T
Non cytoplasmic domain
u.
Endotoxin_N
delta-Endotoxin (insectocide), N-terminal domain
412. Bacteria__Bacillus_thuringiensis_WP_080545918.1
Signal pe...
S
Non cytoplasmic domain
Signal Peptide
C..
delta-Endotoxin (insectocide), N-terminal domain
Endotoxin_N
FAMILY NOT NAMED
un...
413. Bacteria__Bacillus_cereus_WP_080470008.1
Coil
delta-Endotoxin (insectocide), N-terminal domain
Endotoxin_N
FAMILY NOT NAMED
414. Bacteria__Stenotrophomonas_maltophilia_WP_080335079.1
Si...
Tran...
Cytoplasmic domain
T.
Non cytoplasmic domain
Sig...
TAT
delta-Endotoxin (insectocide), N-terminal domain
Endotoxin_N
unkn...
415. Bacteria__Spirosoma_rigui_WP_080237663.1
delta-Endotoxin (insectocide), N-terminal domain
FAMILY NOT NAMED
Endotoxin_N
416. Bacteria__Bacillus_cereus_WP_080118443.1
delta-Endotoxin (insectocide), N-terminal domain
Endotoxin_N
417. Bacteria__Spirosoma_aerolatum_WP_080055642.1
Non cytoplasmic domain
FAMILY NOT NAMED
delta-Endotoxin (insectocide), N-terminal domain
Endotoxin_N
418. Bacteria__Spirosoma_aerolatum_WP_080055637.1
Non cytoplasmic domain
FAMILY NOT NAMED
delta-Endotoxin (insectocide), N-terminal domain
Endotoxin_N

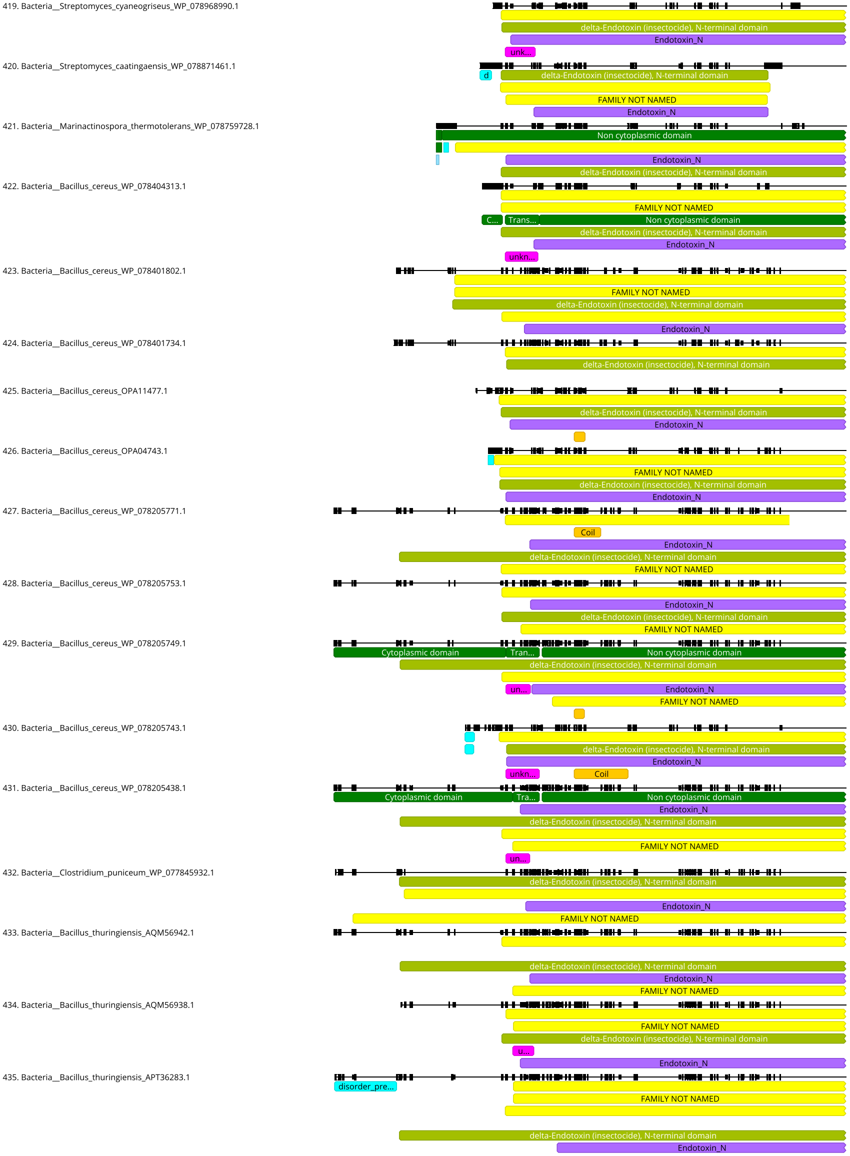
419. Bacteria__Streptomyces_cyaneogriseus_WP_078968990.1
delta-Endotoxin (insectocide), N-terminal domain
Endotoxin_N
unk...
420. Bacteria__Streptomyces_caatingaensis_WP_078871461.1
d
delta-Endotoxin (insectocide), N-terminal domain
FAMILY NOT NAMED
Endotoxin_N
421. Bacteria__Marinactinospora_thermotolerans_WP_078759728.1
Non cytoplasmic domain
Endotoxin_N
delta-Endotoxin (insectocide), N-terminal domain
422. Bacteria__Bacillus_cereus_WP_078404313.1
FAMILY NOT NAMED
C...
Trans...
Non cytoplasmic domain
delta-Endotoxin (insectocide), N-terminal domain
Endotoxin_N
unkn...
423. Bacteria__Bacillus_cereus_WP_078401802.1
FAMILY NOT NAMED
delta-Endotoxin (insectocide), N-terminal domain
Endotoxin_N
424. Bacteria__Bacillus_cereus_WP_078401734.1
delta-Endotoxin (insectocide), N-terminal domain
425. Bacteria__Bacillus_cereus_OPA11477.1
delta-Endotoxin (insectocide), N-terminal domain
Endotoxin_N
426. Bacteria__Bacillus_cereus_OPA04743.1
FAMILY NOT NAMED
delta-Endotoxin (insectocide), N-terminal domain
Endotoxin_N
427. Bacteria__Bacillus_cereus_WP_078205771.1
Coil
Endotoxin_N
delta-Endotoxin (insectocide), N-terminal domain
FAMILY NOT NAMED
428. Bacteria__Bacillus_cereus_WP_078205753.1
Endotoxin_N
delta-Endotoxin (insectocide), N-terminal domain
FAMILY NOT NAMED
429. Bacteria__Bacillus_cereus_WP_078205749.1
Cytoplasmic domain
Tran...
Non cytoplasmic domain
delta-Endotoxin (insectocide), N-terminal domain
un...
Endotoxin_N
FAMILY NOT NAMED
430. Bacteria__Bacillus_cereus_WP_078205743.1
delta-Endotoxin (insectocide), N-terminal domain
Endotoxin_N
unkn...
Coil
431. Bacteria__Bacillus_cereus_WP_078205438.1
Cytoplasmic domain
Tra...
Non cytoplasmic domain
Endotoxin_N
delta-Endotoxin (insectocide), N-terminal domain
FAMILY NOT NAMED
un...
432. Bacteria__Clostridium_puniceum_WP_077845932.1
delta-Endotoxin (insectocide), N-terminal domain
Endotoxin_N
FAMILY NOT NAMED
433. Bacteria__Bacillus_thuringiensis_AQM56942.1
delta-Endotoxin (insectocide), N-terminal domain
Endotoxin_N
FAMILY NOT NAMED
434. Bacteria__Bacillus_thuringiensis_AQM56938.1
FAMILY NOT NAMED
delta-Endotoxin (insectocide), N-terminal domain
u...
Endotoxin_N
435. Bacteria__Bacillus_thuringiensis_APT36283.1
disorder_pre...
FAMILY NOT NAMED
delta-Endotoxin (insectocide), N-terminal domain
Endotoxin_N

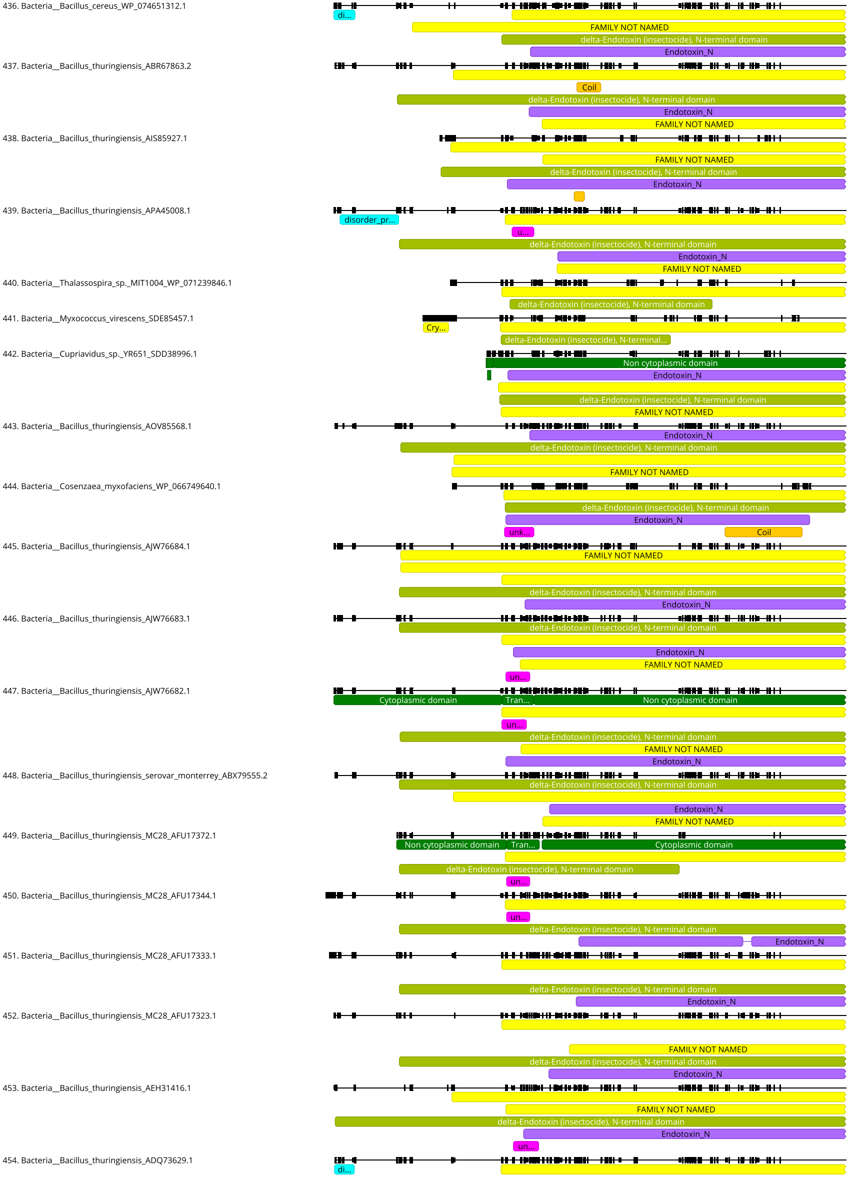
436. Bacteria__Bacillus_cereus_WP_074651312.1
di...
FAMILY NOT NAMED
delta-Endotoxin (insectocide), N-terminal domain
Endotoxin_N
437. Bacteria__Bacillus_thuringiensis_ABR67863.2
Coil
delta-Endotoxin (insectocide), N-terminal domain
Endotoxin_N
FAMILY NOT NAMED
438. Bacteria__Bacillus_thuringiensis_AIS85927.1
FAMILY NOT NAMED
delta-Endotoxin (insectocide), N-terminal domain
Endotoxin_N
439. Bacteria__Bacillus_thuringiensis_APA45008.1
disorder_pr...
u...
delta-Endotoxin (insectocide), N-terminal domain
Endotoxin_N
FAMILY NOT NAMED
440. Bacteria__Thalassospira_sp._MIT1004_WP_071239846.1
delta-Endotoxin (insectocide), N-terminal domain
441. Bacteria__Myxococcus_virescens_SDE85457.1
Cry...
delta-Endotoxin (insectocide), N-terminal...
442. Bacteria__Cupriavidus_sp._YR651_SDD38996.1
Non cytoplasmic domain
Endotoxin_N
delta-Endotoxin (insectocide), N-terminal domain
FAMILY NOT NAMED
443. Bacteria__Bacillus_thuringiensis_AOV85568.1
Endotoxin_N
delta-Endotoxin (insectocide), N-terminal domain
FAMILY NOT NAMED
444. Bacteria__Cosenzaea_myxofaciens_WP_066749640.1
delta-Endotoxin (insectocide), N-terminal domain
Endotoxin_N
unk...
Coil
445. Bacteria__Bacillus_thuringiensis_AJW76684.1
FAMILY NOT NAMED
delta-Endotoxin (insectocide), N-terminal domain
Endotoxin_N
446. Bacteria__Bacillus_thuringiensis_AJW76683.1
delta-Endotoxin (insectocide), N-terminal domain
Endotoxin_N
FAMILY NOT NAMED
un...
447. Bacteria__Bacillus_thuringiensis_AJW76682.1
Cytoplasmic domain
Tran...
Non cytoplasmic domain
un...
delta-Endotoxin (insectocide), N-terminal domain
FAMILY NOT NAMED
Endotoxin_N
448. Bacteria__Bacillus_thuringiensis_serovar_monterrey_ABX79555.2
delta-Endotoxin (insectocide), N-terminal domain
Endotoxin_N
FAMILY NOT NAMED
449. Bacteria__Bacillus_thuringiensis_MC28_AFU17372.1
Non cytoplasmic domain
Tran...
Cytoplasmic domain
delta-Endotoxin (insectocide), N-terminal domain
un...
450. Bacteria__Bacillus_thuringiensis_MC28_AFU17344.1
un...
delta-Endotoxin (insectocide), N-terminal domain
Endotoxin_N
451. Bacteria__Bacillus_thuringiensis_MC28_AFU17333.1
delta-Endotoxin (insectocide), N-terminal domain
Endotoxin_N
452. Bacteria__Bacillus_thuringiensis_MC28_AFU17323.1
FAMILY NOT NAMED
delta-Endotoxin (insectocide), N-terminal domain
Endotoxin_N
453. Bacteria__Bacillus_thuringiensis_AEH31416.1
FAMILY NOT NAMED
delta-Endotoxin (insectocide), N-terminal domain
Endotoxin_N
un...
454. Bacteria__Bacillus_thuringiensis_ADQ73629.1
di...

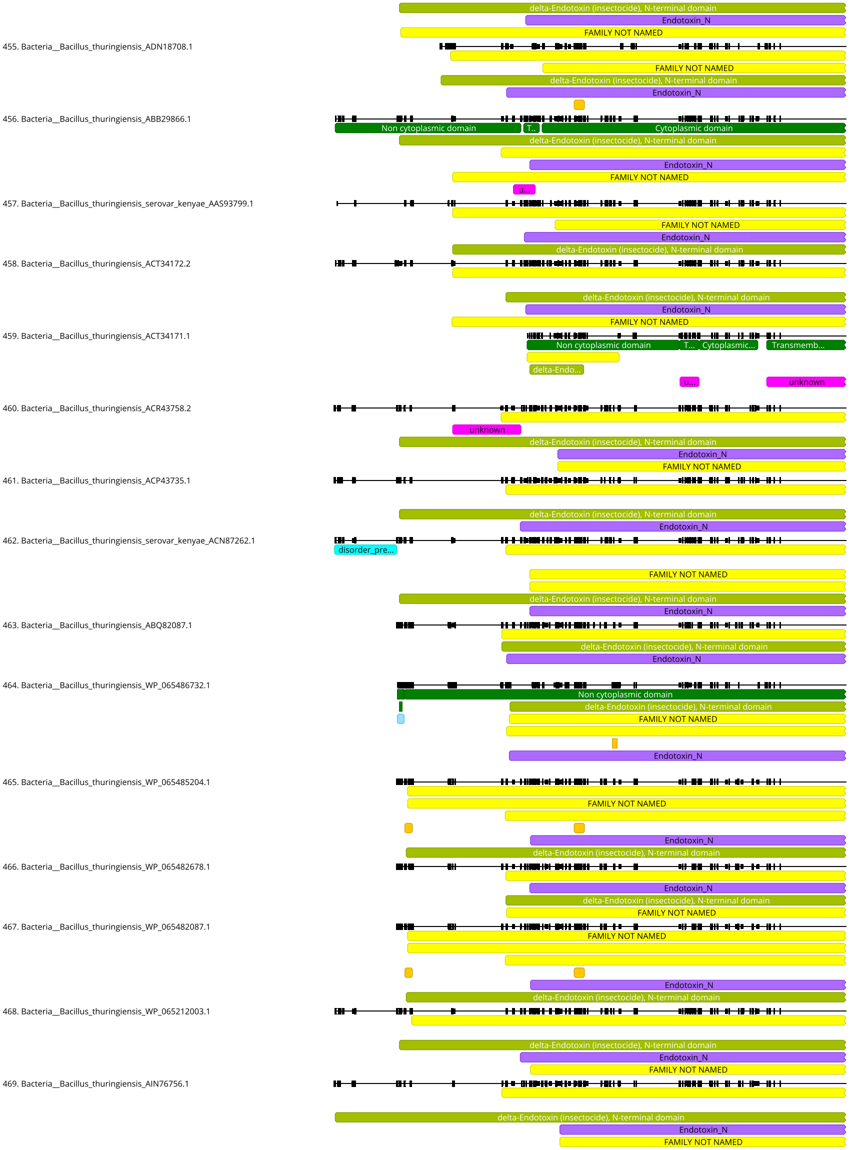

delta-Endotoxin (insectocide), N-terminal domain
Endotoxin_N
FAMILY NOT NAMED
455. Bacteria__Bacillus_thuringiensis_ADN18708.1
FAMILY NOT NAMED
delta-Endotoxin (insectocide), N-terminal domain
Endotoxin_N
456. Bacteria__Bacillus_thuringiensis_ABB29866.1
Non cytoplasmic domain
T...
Cytoplasmic domain
delta-Endotoxin (insectocide), N-terminal domain
Endotoxin_N
FAMILY NOT NAMED
u...
457. Bacteria__Bacillus_thuringiensis_serovar_kenyae_AAS93799.1
FAMILY NOT NAMED
Endotoxin_N
delta-Endotoxin (insectocide), N-terminal domain
458. Bacteria__Bacillus_thuringiensis_ACT34172.2
delta-Endotoxin (insectocide), N-terminal domain
Endotoxin_N
FAMILY NOT NAMED
459. Bacteria__Bacillus_thuringiensis_ACT34171.1
Non cytoplasmic domain
T...
Cytoplasmic...
Transmemb...
delta-Endo...
u...
unknown
460. Bacteria__Bacillus_thuringiensis_ACR43758.2
unknown
delta-Endotoxin (insectocide), N-terminal domain
Endotoxin_N
FAMILY NOT NAMED
461. Bacteria__Bacillus_thuringiensis_ACP43735.1
delta-Endotoxin (insectocide), N-terminal domain
Endotoxin_N
462. Bacteria__Bacillus_thuringiensis_serovar_kenyae_ACN87262.1
disorder_pre...
FAMILY NOT NAMED
delta-Endotoxin (insectocide), N-terminal domain
Endotoxin_N
463. Bacteria__Bacillus_thuringiensis_ABQ82087.1
delta-Endotoxin (insectocide), N-terminal domain
Endotoxin_N
464. Bacteria__Bacillus_thuringiensis_WP_065486732.1
Non cytoplasmic domain
delta-Endotoxin (insectocide), N-terminal domain
FAMILY NOT NAMED
Endotoxin_N
465. Bacteria__Bacillus_thuringiensis_WP_065485204.1
FAMILY NOT NAMED
Endotoxin_N
delta-Endotoxin (insectocide), N-terminal domain
466. Bacteria__Bacillus_thuringiensis_WP_065482678.1
Endotoxin_N
delta-Endotoxin (insectocide), N-terminal domain
FAMILY NOT NAMED
467. Bacteria__Bacillus_thuringiensis_WP_065482087.1
FAMILY NOT NAMED
Endotoxin_N
delta-Endotoxin (insectocide), N-terminal domain
468. Bacteria__Bacillus_thuringiensis_WP_065212003.1
delta-Endotoxin (insectocide), N-terminal domain
Endotoxin_N
FAMILY NOT NAMED
469. Bacteria__Bacillus_thuringiensis_AIN76756.1
delta-Endotoxin (insectocide), N-terminal domain
Endotoxin_N
FAMILY NOT NAMED

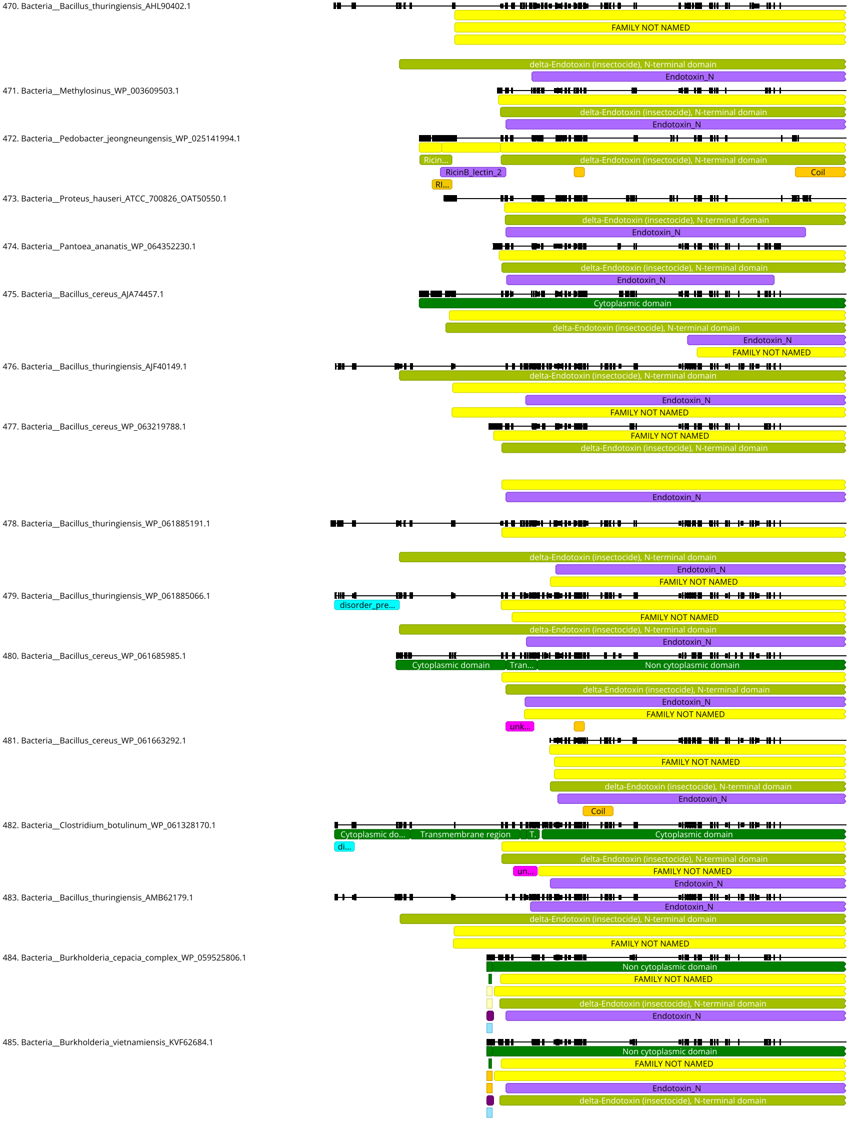
470. Bacteria__Bacillus_thuringiensis_AHL90402.1
FAMILY NOT NAMED
delta-Endotoxin (insectocide), N-terminal domain
Endotoxin_N
471. Bacteria__Methylosinus_WP_003609503.1
delta-Endotoxin (insectocide), N-terminal domain
Endotoxin_N
472. Bacteria__Pedobacter_jeongneungensis_WP_025141994.1
Ricin...
delta-Endotoxin (insectocide), N-terminal domain
RicinB_lectin_2
Coil
RI...
473. Bacteria__Proteus_hauseri_ATCC_700826_OAT50550.1
delta-Endotoxin (insectocide), N-terminal domain
Endotoxin_N
474. Bacteria__Pantoea_ananatis_WP_064352230.1
delta-Endotoxin (insectocide), N-terminal domain
Endotoxin_N
475. Bacteria__Bacillus_cereus_AJA74457.1
Cytoplasmic domain
delta-Endotoxin (insectocide), N-terminal domain
Endotoxin_N
FAMILY NOT NAMED
476. Bacteria__Bacillus_thuringiensis_AJF40149.1
delta-Endotoxin (insectocide), N-terminal domain
Endotoxin_N
FAMILY NOT NAMED
477. Bacteria__Bacillus_cereus_WP_063219788.1
FAMILY NOT NAMED
delta-Endotoxin (insectocide), N-terminal domain
Endotoxin_N
478. Bacteria__Bacillus_thuringiensis_WP_061885191.1
delta-Endotoxin (insectocide), N-terminal domain
Endotoxin_N
FAMILY NOT NAMED
479. Bacteria__Bacillus_thuringiensis_WP_061885066.1
disorder_pre...
FAMILY NOT NAMED
delta-Endotoxin (insectocide), N-terminal domain
Endotoxin_N
480. Bacteria__Bacillus_cereus_WP_061685985.1
Cytoplasmic domain
Tran...
Non cytoplasmic domain
delta-Endotoxin (insectocide), N-terminal domain
Endotoxin_N
FAMILY NOT NAMED
unk...
481. Bacteria__Bacillus_cereus_WP_061663292.1
FAMILY NOT NAMED
delta-Endotoxin (insectocide), N-terminal domain
Endotoxin_N
Coil
482. Bacteria__Clostridium_botulinum_WP_061328170.1
Cytoplasmic do...
Transmembrane region
T.
Cytoplasmic domain
di...
delta-Endotoxin (insectocide), N-terminal domain
un...
FAMILY NOT NAMED
Endotoxin_N
483. Bacteria__Bacillus_thuringiensis_AMB62179.1
Endotoxin_N
delta-Endotoxin (insectocide), N-terminal domain
FAMILY NOT NAMED
484. Bacteria__Burkholderia_cepacia_complex_WP_059525806.1
Non cytoplasmic domain
FAMILY NOT NAMED
delta-Endotoxin (insectocide), N-terminal domain
Endotoxin_N
485. Bacteria__Burkholderia_vietnamiensis_KVF62684.1
Non cytoplasmic domain
FAMILY NOT NAMED
Endotoxin_N
delta-Endotoxin (insectocide), N-terminal domain

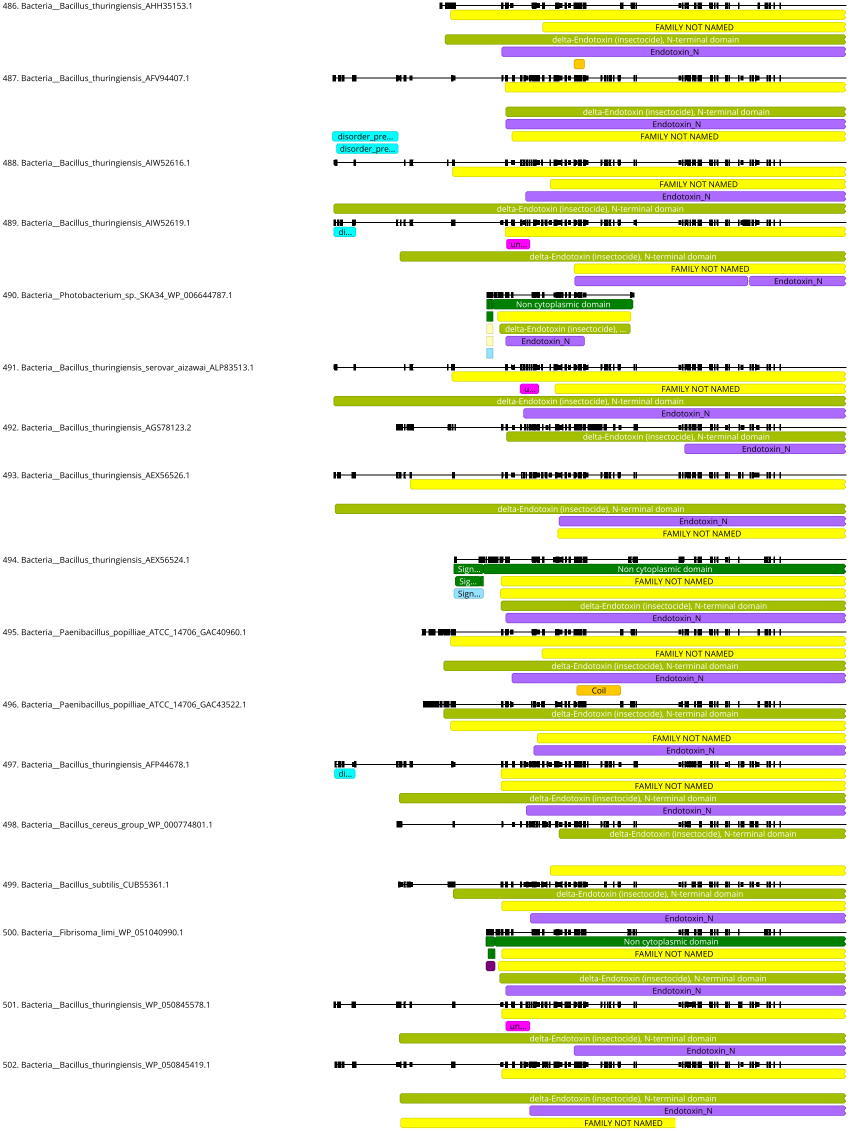
486. Bacteria__Bacillus_thuringiensis_AHH35153.1
FAMILY NOT NAMED
delta-Endotoxin (insectocide), N-terminal domain
Endotoxin_N
487. Bacteria__Bacillus_thuringiensis_AFV94407.1
delta-Endotoxin (insectocide), N-terminal domain
Endotoxin_N
disorder_pre...
FAMILY NOT NAMED
disorder_pre...
488. Bacteria__Bacillus_thuringiensis_AIW52616.1
FAMILY NOT NAMED
Endotoxin_N
delta-Endotoxin (insectocide), N-terminal domain
489. Bacteria__Bacillus_thuringiensis_AIW52619.1
di...
un...
delta-Endotoxin (insectocide), N-terminal domain
FAMILY NOT NAMED
Endotoxin_N
490. Bacteria__Photobacterium_sp._SKA34_WP_006644787.1
Non cytoplasmic domain
delta-Endotoxin (insectocide), ...
Endotoxin_N
491. Bacteria__Bacillus_thuringiensis_serovar_aizawai_ALP83513.1
u...
FAMILY NOT NAMED
delta-Endotoxin (insectocide), N-terminal domain
Endotoxin_N
492. Bacteria__Bacillus_thuringiensis_AGS78123.2
delta-Endotoxin (insectocide), N-terminal domain
Endotoxin_N
493. Bacteria__Bacillus_thuringiensis_AEX56526.1
delta-Endotoxin (insectocide), N-terminal domain
Endotoxin_N
FAMILY NOT NAMED
494. Bacteria__Bacillus_thuringiensis_AEX56524.1
Sign...
Non cytoplasmic domain
Sig...
FAMILY NOT NAMED
Sign...
delta-Endotoxin (insectocide), N-terminal domain
Endotoxin_N
495. Bacteria__Paenibacillus_popilliae_ATCC_14706_GAC40960.1
FAMILY NOT NAMED
delta-Endotoxin (insectocide), N-terminal domain
Endotoxin_N
Coil
496. Bacteria__Paenibacillus_popilliae_ATCC_14706_GAC43522.1
delta-Endotoxin (insectocide), N-terminal domain
FAMILY NOT NAMED
Endotoxin_N
497. Bacteria__Bacillus_thuringiensis_AFP44678.1
di...
FAMILY NOT NAMED
delta-Endotoxin (insectocide), N-terminal domain
Endotoxin_N
498. Bacteria__Bacillus_cereus_group_WP_000774801.1
delta-Endotoxin (insectocide), N-terminal domain
499. Bacteria__Bacillus_subtilis_CUB55361.1
delta-Endotoxin (insectocide), N-terminal domain
Endotoxin_N
500. Bacteria__Fibrisoma_limi_WP_051040990.1
Non cytoplasmic domain
FAMILY NOT NAMED
delta-Endotoxin (insectocide), N-terminal domain
Endotoxin_N
501. Bacteria__Bacillus_thuringiensis_WP_050845578.1
un...
delta-Endotoxin (insectocide), N-terminal domain
Endotoxin_N
502. Bacteria__Bacillus_thuringiensis_WP_050845419.1
delta-Endotoxin (insectocide), N-terminal domain
Endotoxin_N
FAMILY NOT NAMED

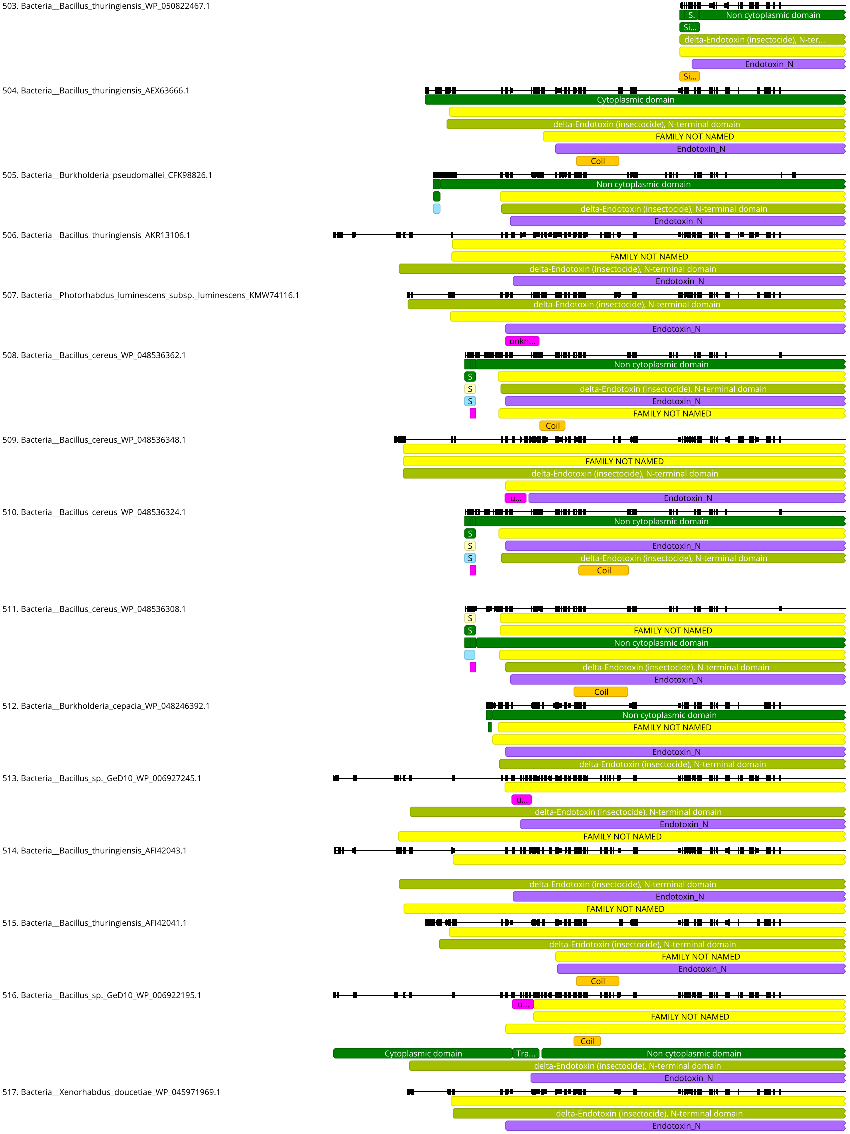
503. Bacteria__Bacillus_thuringiensis_WP_050822467.1
S.
Non cytoplasmic domain
Si...
delta-Endotoxin (insectocide), N-ter...
Endotoxin_N
Si...
504. Bacteria__Bacillus_thuringiensis_AEX63666.1
Cytoplasmic domain
delta-Endotoxin (insectocide), N-terminal domain
FAMILY NOT NAMED
Endotoxin_N
Coil
505. Bacteria__Burkholderia_pseudomallei_CFK98826.1
Non cytoplasmic domain
delta-Endotoxin (insectocide), N-terminal domain
Endotoxin_N
506. Bacteria__Bacillus_thuringiensis_AKR13106.1
FAMILY NOT NAMED
delta-Endotoxin (insectocide), N-terminal domain
Endotoxin_N
507. Bacteria__Photorhabdus_luminescens_subsp._luminescens_KMW74116.1
delta-Endotoxin (insectocide), N-terminal domain
Endotoxin_N
unkn...
508. Bacteria__Bacillus_cereus_WP_048536362.1
Non cytoplasmic domain
S
S
delta-Endotoxin (insectocide), N-terminal domain
S
Endotoxin_N
FAMILY NOT NAMED
Coil
509. Bacteria__Bacillus_cereus_WP_048536348.1
FAMILY NOT NAMED
delta-Endotoxin (insectocide), N-terminal domain
u...
Endotoxin_N
510. Bacteria__Bacillus_cereus_WP_048536324.1
Non cytoplasmic domain
S
S
Endotoxin_N
S
delta-Endotoxin (insectocide), N-terminal domain
Coil
511. Bacteria__Bacillus_cereus_WP_048536308.1
S
S
FAMILY NOT NAMED
Non cytoplasmic domain
delta-Endotoxin (insectocide), N-terminal domain
Endotoxin_N
Coil
512. Bacteria__Burkholderia_cepacia_WP_048246392.1
Non cytoplasmic domain
FAMILY NOT NAMED
Endotoxin_N
delta-Endotoxin (insectocide), N-terminal domain
513. Bacteria__Bacillus_sp._GeD10_WP_006927245.1
u...
delta-Endotoxin (insectocide), N-terminal domain
Endotoxin_N
FAMILY NOT NAMED
514. Bacteria__Bacillus_thuringiensis_AFI42043.1
delta-Endotoxin (insectocide), N-terminal domain
Endotoxin_N
FAMILY NOT NAMED
515. Bacteria__Bacillus_thuringiensis_AFI42041.1
delta-Endotoxin (insectocide), N-terminal domain
FAMILY NOT NAMED
Endotoxin_N
Coil
516. Bacteria__Bacillus_sp._GeD10_WP_006922195.1
u...
FAMILY NOT NAMED
Coil
Cytoplasmic domain
Tra...
Non cytoplasmic domain
delta-Endotoxin (insectocide), N-terminal domain
Endotoxin_N
517. Bacteria__Xenorhabdus_doucetiae_WP_045971969.1
delta-Endotoxin (insectocide), N-terminal domain
Endotoxin_N

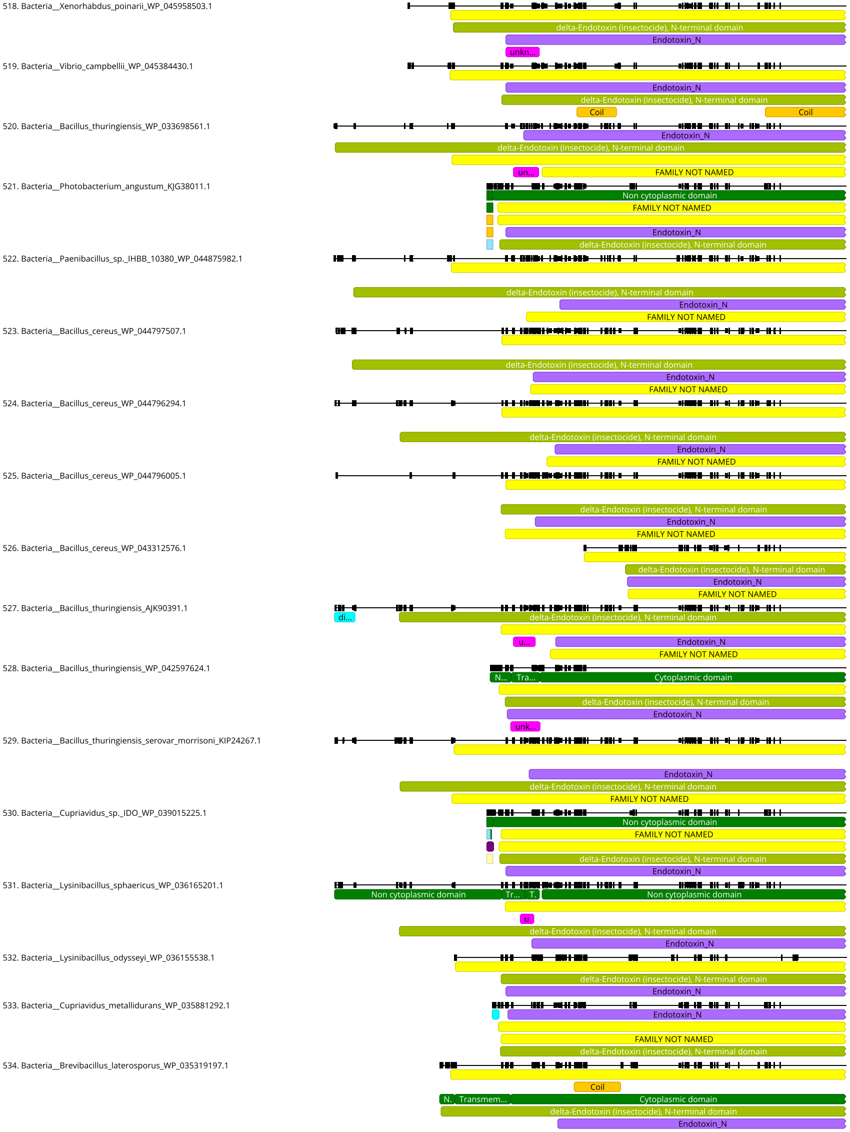
518. Bacteria__Xenorhabdus_poinarii_WP_045958503.1
delta-Endotoxin (insectocide), N-terminal domain
Endotoxin_N
unkn...
519. Bacteria__Vibrio_campbellii_WP_045384430.1
Endotoxin_N
delta-Endotoxin (insectocide), N-terminal domain
Coil
Coil
520. Bacteria__Bacillus_thuringiensis_WP_033698561.1
Endotoxin_N
delta-Endotoxin (insectocide), N-terminal domain
un...
FAMILY NOT NAMED
521. Bacteria__Photobacterium_angustum_KJG38011.1
Non cytoplasmic domain
FAMILY NOT NAMED
Endotoxin_N
delta-Endotoxin (insectocide), N-terminal domain
522. Bacteria__Paenibacillus_sp._IHBB_10380_WP_044875982.1
delta-Endotoxin (insectocide), N-terminal domain
Endotoxin_N
FAMILY NOT NAMED
523. Bacteria__Bacillus_cereus_WP_044797507.1
delta-Endotoxin (insectocide), N-terminal domain
Endotoxin_N
FAMILY NOT NAMED
524. Bacteria__Bacillus_cereus_WP_044796294.1
delta-Endotoxin (insectocide), N-terminal domain
Endotoxin_N
FAMILY NOT NAMED
525. Bacteria__Bacillus_cereus_WP_044796005.1
delta-Endotoxin (insectocide), N-terminal domain
Endotoxin_N
FAMILY NOT NAMED
526. Bacteria__Bacillus_cereus_WP_043312576.1
delta-Endotoxin (insectocide), N-terminal domain
Endotoxin_N
FAMILY NOT NAMED
527. Bacteria__Bacillus_thuringiensis_AJK90391.1
di...
delta-Endotoxin (insectocide), N-terminal domain
u...
Endotoxin_N
FAMILY NOT NAMED
528. Bacteria__Bacillus_thuringiensis_WP_042597624.1
N...
Tra...
Cytoplasmic domain
delta-Endotoxin (insectocide), N-terminal domain
Endotoxin_N
unk...
529. Bacteria__Bacillus_thuringiensis_serovar_morrisoni_KIP24267.1
Endotoxin_N
delta-Endotoxin (insectocide), N-terminal domain
FAMILY NOT NAMED
530. Bacteria__Cupriavidus_sp._IDO_WP_039015225.1
Non cytoplasmic domain
FAMILY NOT NAMED
delta-Endotoxin (insectocide), N-terminal domain
Endotoxin_N
531. Bacteria__Lysinibacillus_sphaericus_WP_036165201.1
Non cytoplasmic domain
Tr...
T.
Non cytoplasmic domain
u.
delta-Endotoxin (insectocide), N-terminal domain
Endotoxin_N
532. Bacteria__Lysinibacillus_odysseyi_WP_036155538.1
delta-Endotoxin (insectocide), N-terminal domain
Endotoxin_N
533. Bacteria__Cupriavidus_metallidurans_WP_035881292.1
Endotoxin_N
FAMILY NOT NAMED
delta-Endotoxin (insectocide), N-terminal domain
534. Bacteria__Brevibacillus_laterosporus_WP_035319197.1
Coil
N.
Transmem...
Cytoplasmic domain
delta-Endotoxin (insectocide), N-terminal domain
Endotoxin_N

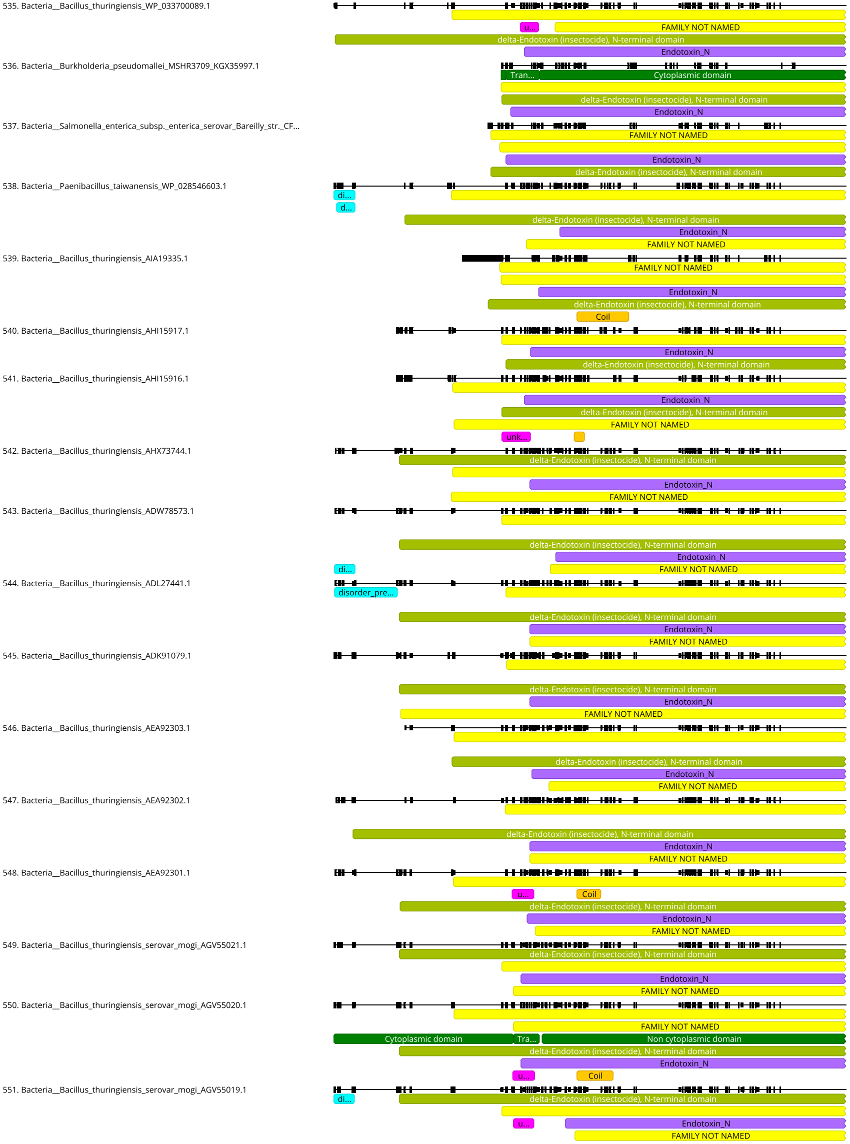
535. Bacteria__Bacillus_thuringiensis_WP_033700089.1
u...
FAMILY NOT NAMED
delta-Endotoxin (insectocide), N-terminal domain
Endotoxin_N
536. Bacteria__Burkholderia_pseudomallei_MSHR3709_KGX35997.1
Tran...
Cytoplasmic domain
delta-Endotoxin (insectocide), N-terminal domain
Endotoxin_N
537. Bacteria__Salmonella_enterica_subsp._enterica_serovar_Bareilly_str._CF...
FAMILY NOT NAMED
Endotoxin_N
delta-Endotoxin (insectocide), N-terminal domain
538. Bacteria__Paenibacillus_taiwanensis_WP_028546603.1
di...
d...
delta-Endotoxin (insectocide), N-terminal domain
Endotoxin_N
FAMILY NOT NAMED
539. Bacteria__Bacillus_thuringiensis_AIA19335.1
FAMILY NOT NAMED
Endotoxin_N
delta-Endotoxin (insectocide), N-terminal domain
Coil
540. Bacteria__Bacillus_thuringiensis_AHI15917.1
Endotoxin_N
delta-Endotoxin (insectocide), N-terminal domain
541. Bacteria__Bacillus_thuringiensis_AHI15916.1
Endotoxin_N
delta-Endotoxin (insectocide), N-terminal domain
FAMILY NOT NAMED
unk...
542. Bacteria__Bacillus_thuringiensis_AHX73744.1
delta-Endotoxin (insectocide), N-terminal domain
Endotoxin_N
FAMILY NOT NAMED
543. Bacteria__Bacillus_thuringiensis_ADW78573.1
delta-Endotoxin (insectocide), N-terminal domain
Endotoxin_N
di...
FAMILY NOT NAMED
544. Bacteria__Bacillus_thuringiensis_ADL27441.1
disorder_pre...
delta-Endotoxin (insectocide), N-terminal domain
Endotoxin_N
FAMILY NOT NAMED
545. Bacteria__Bacillus_thuringiensis_ADK91079.1
delta-Endotoxin (insectocide), N-terminal domain
Endotoxin_N
FAMILY NOT NAMED
546. Bacteria__Bacillus_thuringiensis_AEA92303.1
delta-Endotoxin (insectocide), N-terminal domain
Endotoxin_N
FAMILY NOT NAMED
547. Bacteria__Bacillus_thuringiensis_AEA92302.1
delta-Endotoxin (insectocide), N-terminal domain
Endotoxin_N
FAMILY NOT NAMED
548. Bacteria__Bacillus_thuringiensis_AEA92301.1
u...
Coil
delta-Endotoxin (insectocide), N-terminal domain
Endotoxin_N
FAMILY NOT NAMED
549. Bacteria__Bacillus_thuringiensis_serovar_mogi_AGV55021.1
delta-Endotoxin (insectocide), N-terminal domain
Endotoxin_N
FAMILY NOT NAMED
550. Bacteria__Bacillus_thuringiensis_serovar_mogi_AGV55020.1
FAMILY NOT NAMED
Cytoplasmic domain
Tra...
Non cytoplasmic domain
delta-Endotoxin (insectocide), N-terminal domain
Endotoxin_N
u...
Coil
551. Bacteria__Bacillus_thuringiensis_serovar_mogi_AGV55019.1
di...
delta-Endotoxin (insectocide), N-terminal domain
u...
Endotoxin_N
FAMILY NOT NAMED

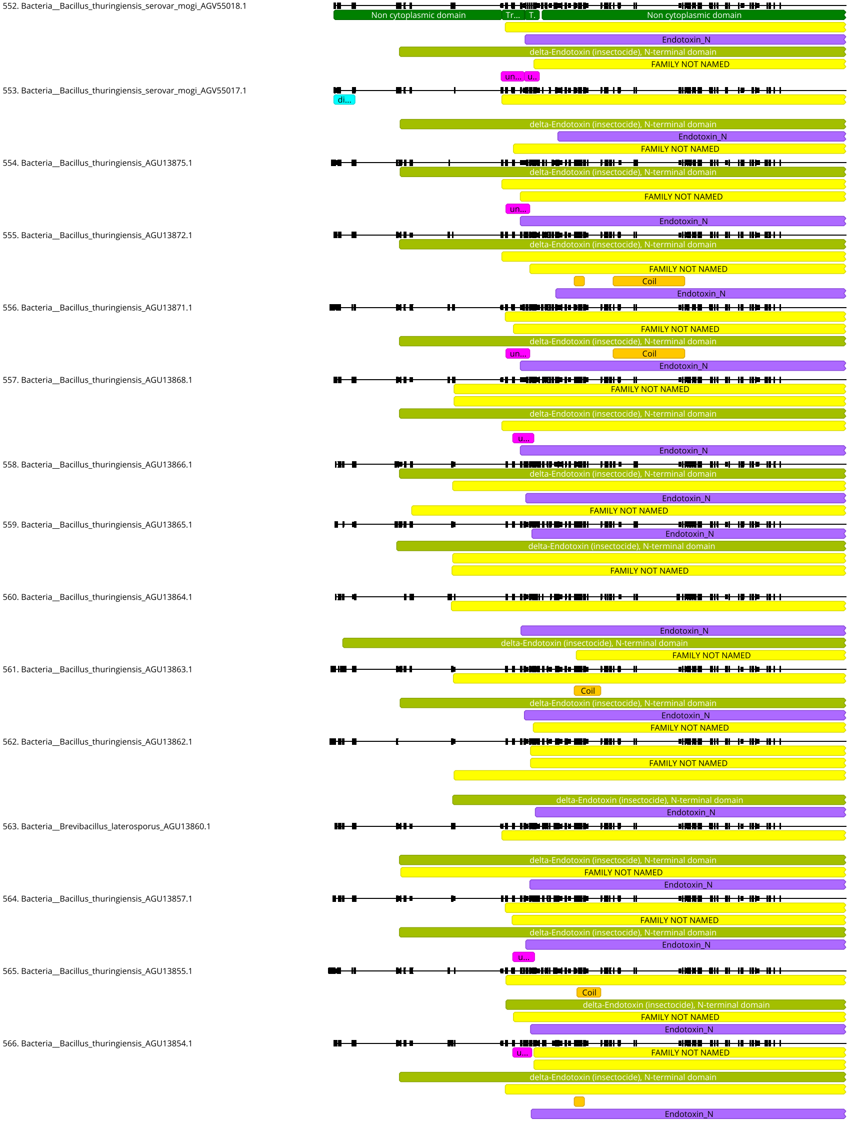
552. Bacteria__Bacillus_thuringiensis_serovar_mogi_AGV55018.1
Non cytoplasmic domain
Tr...
T.
Non cytoplasmic domain
Endotoxin_N
delta-Endotoxin (insectocide), N-terminal domain
FAMILY NOT NAMED
un...
u..
553. Bacteria__Bacillus_thuringiensis_serovar_mogi_AGV55017.1
di...
delta-Endotoxin (insectocide), N-terminal domain
Endotoxin_N
FAMILY NOT NAMED
554. Bacteria__Bacillus_thuringiensis_AGU13875.1
delta-Endotoxin (insectocide), N-terminal domain
FAMILY NOT NAMED
un...
Endotoxin_N
555. Bacteria__Bacillus_thuringiensis_AGU13872.1
delta-Endotoxin (insectocide), N-terminal domain
FAMILY NOT NAMED
Coil
Endotoxin_N
556. Bacteria__Bacillus_thuringiensis_AGU13871.1
FAMILY NOT NAMED
delta-Endotoxin (insectocide), N-terminal domain
un...
Coil
Endotoxin_N
557. Bacteria__Bacillus_thuringiensis_AGU13868.1
FAMILY NOT NAMED
delta-Endotoxin (insectocide), N-terminal domain
u...
Endotoxin_N
558. Bacteria__Bacillus_thuringiensis_AGU13866.1
delta-Endotoxin (insectocide), N-terminal domain
Endotoxin_N
FAMILY NOT NAMED
559. Bacteria__Bacillus_thuringiensis_AGU13865.1
Endotoxin_N
delta-Endotoxin (insectocide), N-terminal domain
FAMILY NOT NAMED
560. Bacteria__Bacillus_thuringiensis_AGU13864.1
Endotoxin_N
delta-Endotoxin (insectocide), N-terminal domain
FAMILY NOT NAMED
561. Bacteria__Bacillus_thuringiensis_AGU13863.1
Coil
delta-Endotoxin (insectocide), N-terminal domain
Endotoxin_N
FAMILY NOT NAMED
562. Bacteria__Bacillus_thuringiensis_AGU13862.1
FAMILY NOT NAMED
delta-Endotoxin (insectocide), N-terminal domain
Endotoxin_N
563. Bacteria__Brevibacillus_laterosporus_AGU13860.1
delta-Endotoxin (insectocide), N-terminal domain
FAMILY NOT NAMED
Endotoxin_N
564. Bacteria__Bacillus_thuringiensis_AGU13857.1
FAMILY NOT NAMED
delta-Endotoxin (insectocide), N-terminal domain
Endotoxin_N
u...
565. Bacteria__Bacillus_thuringiensis_AGU13855.1
Coil
delta-Endotoxin (insectocide), N-terminal domain
FAMILY NOT NAMED
Endotoxin_N
566. Bacteria__Bacillus_thuringiensis_AGU13854.1
u...
FAMILY NOT NAMED
delta-Endotoxin (insectocide), N-terminal domain
Endotoxin_N

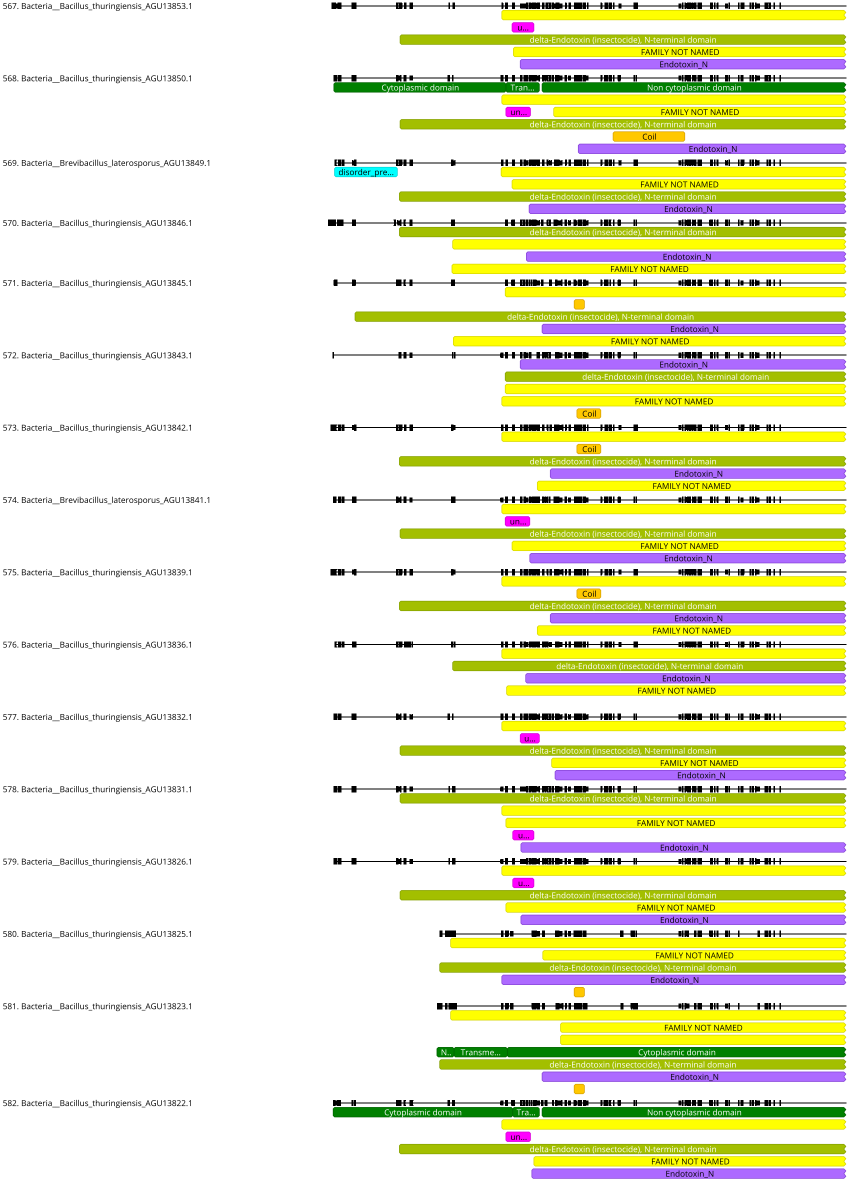
567. Bacteria__Bacillus_thuringiensis_AGU13853.1
u...
delta-Endotoxin (insectocide), N-terminal domain
FAMILY NOT NAMED
Endotoxin_N
568. Bacteria__Bacillus_thuringiensis_AGU13850.1
Cytoplasmic domain
Tran...
Non cytoplasmic domain
un...
FAMILY NOT NAMED
delta-Endotoxin (insectocide), N-terminal domain
Coil
Endotoxin_N
569. Bacteria__Brevibacillus_laterosporus_AGU13849.1
disorder_pre...
FAMILY NOT NAMED
delta-Endotoxin (insectocide), N-terminal domain
Endotoxin_N
570. Bacteria__Bacillus_thuringiensis_AGU13846.1
delta-Endotoxin (insectocide), N-terminal domain
Endotoxin_N
FAMILY NOT NAMED
571. Bacteria__Bacillus_thuringiensis_AGU13845.1
delta-Endotoxin (insectocide), N-terminal domain
Endotoxin_N
FAMILY NOT NAMED
572. Bacteria__Bacillus_thuringiensis_AGU13843.1
Endotoxin_N
delta-Endotoxin (insectocide), N-terminal domain
FAMILY NOT NAMED
Coil
573. Bacteria__Bacillus_thuringiensis_AGU13842.1
Coil
delta-Endotoxin (insectocide), N-terminal domain
Endotoxin_N
FAMILY NOT NAMED
574. Bacteria__Brevibacillus_laterosporus_AGU13841.1
un...
delta-Endotoxin (insectocide), N-terminal domain
FAMILY NOT NAMED
Endotoxin_N
575. Bacteria__Bacillus_thuringiensis_AGU13839.1
Coil
delta-Endotoxin (insectocide), N-terminal domain
Endotoxin_N
FAMILY NOT NAMED
576. Bacteria__Bacillus_thuringiensis_AGU13836.1
delta-Endotoxin (insectocide), N-terminal domain
Endotoxin_N
FAMILY NOT NAMED
577. Bacteria__Bacillus_thuringiensis_AGU13832.1
u...
delta-Endotoxin (insectocide), N-terminal domain
FAMILY NOT NAMED
Endotoxin_N
578. Bacteria__Bacillus_thuringiensis_AGU13831.1
delta-Endotoxin (insectocide), N-terminal domain
FAMILY NOT NAMED
u...
Endotoxin_N
579. Bacteria__Bacillus_thuringiensis_AGU13826.1
u...
delta-Endotoxin (insectocide), N-terminal domain
FAMILY NOT NAMED
Endotoxin_N
580. Bacteria__Bacillus_thuringiensis_AGU13825.1
FAMILY NOT NAMED
delta-Endotoxin (insectocide), N-terminal domain
Endotoxin_N
581. Bacteria__Bacillus_thuringiensis_AGU13823.1
FAMILY NOT NAMED
N...
Transme...
Cytoplasmic domain
delta-Endotoxin (insectocide), N-terminal domain
Endotoxin_N
582. Bacteria__Bacillus_thuringiensis_AGU13822.1
Cytoplasmic domain
Tra...
Non cytoplasmic domain
un...
delta-Endotoxin (insectocide), N-terminal domain
FAMILY NOT NAMED
Endotoxin_N

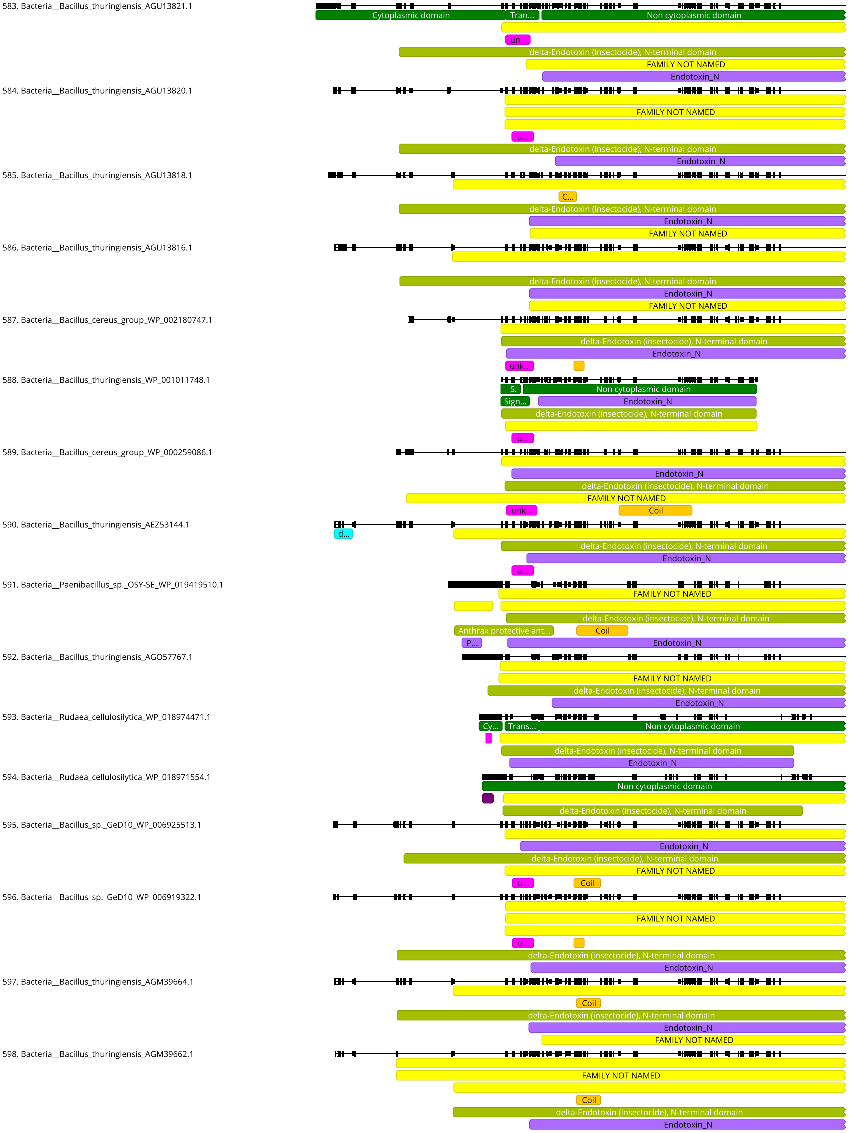
583. Bacteria__Bacillus_thuringiensis_AGU13821.1
Cytoplasmic domain
Tran...
Non cytoplasmic domain
un...
delta-Endotoxin (insectocide), N-terminal domain
FAMILY NOT NAMED
Endotoxin_N
584. Bacteria__Bacillus_thuringiensis_AGU13820.1
FAMILY NOT NAMED
u...
delta-Endotoxin (insectocide), N-terminal domain
Endotoxin_N
585. Bacteria__Bacillus_thuringiensis_AGU13818.1
C...
delta-Endotoxin (insectocide), N-terminal domain
Endotoxin_N
FAMILY NOT NAMED
586. Bacteria__Bacillus_thuringiensis_AGU13816.1
delta-Endotoxin (insectocide), N-terminal domain
Endotoxin_N
FAMILY NOT NAMED
587. Bacteria__Bacillus_cereus_group_WP_002180747.1
delta-Endotoxin (insectocide), N-terminal domain
Endotoxin_N
unk...
588. Bacteria__Bacillus_thuringiensis_WP_001011748.1
S.
Non cytoplasmic domain
Sign...
Endotoxin_N
delta-Endotoxin (insectocide), N-terminal domain
u...
589. Bacteria__Bacillus_cereus_group_WP_000259086.1
Endotoxin_N
delta-Endotoxin (insectocide), N-terminal domain
FAMILY NOT NAMED
unk...
Coil
590. Bacteria__Bacillus_thuringiensis_AEZ53144.1
d...
delta-Endotoxin (insectocide), N-terminal domain
Endotoxin_N
u...
591. Bacteria__Paenibacillus_sp._OSY-SE_WP_019419510.1
FAMILY NOT NAMED
delta-Endotoxin (insectocide), N-terminal domain
Anthrax protective ant...
Coil
P...
Endotoxin_N
592. Bacteria__Bacillus_thuringiensis_AGO57767.1
FAMILY NOT NAMED
delta-Endotoxin (insectocide), N-terminal domain
Endotoxin_N
593. Bacteria__Rudaea_cellulosilytica_WP_018974471.1
Cy...
Trans...
Non cytoplasmic domain
delta-Endotoxin (insectocide), N-terminal domain
Endotoxin_N
594. Bacteria__Rudaea_cellulosilytica_WP_018971554.1
Non cytoplasmic domain
delta-Endotoxin (insectocide), N-terminal domain
595. Bacteria__Bacillus_sp._GeD10_WP_006925513.1
Endotoxin_N
delta-Endotoxin (insectocide), N-terminal domain
FAMILY NOT NAMED
u...
Coil
596. Bacteria__Bacillus_sp._GeD10_WP_006919322.1
FAMILY NOT NAMED
u...
delta-Endotoxin (insectocide), N-terminal domain
Endotoxin_N
597. Bacteria__Bacillus_thuringiensis_AGM39664.1
Coil
delta-Endotoxin (insectocide), N-terminal domain
Endotoxin_N
FAMILY NOT NAMED
598. Bacteria__Bacillus_thuringiensis_AGM39662.1
FAMILY NOT NAMED
Coil
delta-Endotoxin (insectocide), N-terminal domain
Endotoxin_N

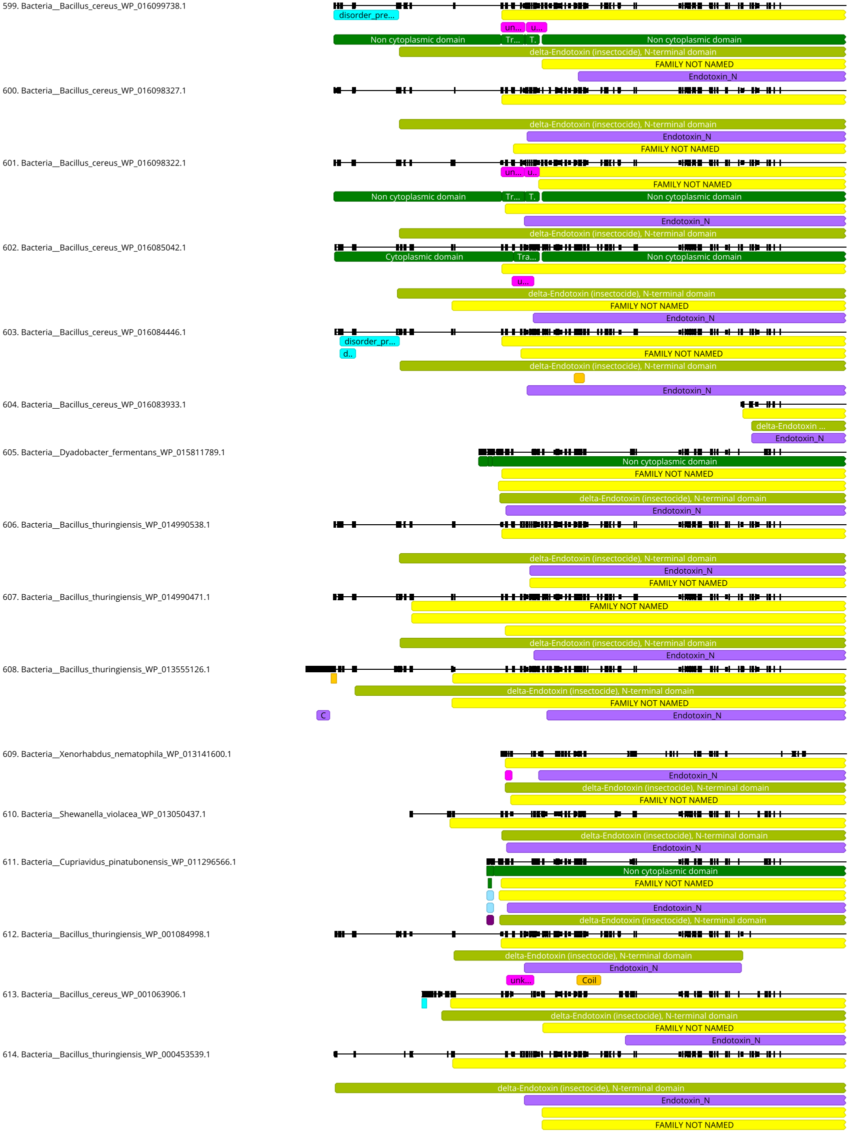
599. Bacteria__Bacillus_cereus_WP_016099738.1
disorder_pre...
un...
u...
Non cytoplasmic domain
Tr...
T.
Non cytoplasmic domain
delta-Endotoxin (insectocide), N-terminal domain
FAMILY NOT NAMED
Endotoxin_N
600. Bacteria__Bacillus_cereus_WP_016098327.1
delta-Endotoxin (insectocide), N-terminal domain
Endotoxin_N
FAMILY NOT NAMED
601. Bacteria__Bacillus_cereus_WP_016098322.1
un...
u..
FAMILY NOT NAMED
Non cytoplasmic domain
Tr...
T.
Non cytoplasmic domain
Endotoxin_N
delta-Endotoxin (insectocide), N-terminal domain
602. Bacteria__Bacillus_cereus_WP_016085042.1
Cytoplasmic domain
Tra...
Non cytoplasmic domain
u...
delta-Endotoxin (insectocide), N-terminal domain
FAMILY NOT NAMED
Endotoxin_N
603. Bacteria__Bacillus_cereus_WP_016084446.1
disorder_pr...
d..
FAMILY NOT NAMED
delta-Endotoxin (insectocide), N-terminal domain
Endotoxin_N
604. Bacteria__Bacillus_cereus_WP_016083933.1
delta-Endotoxin ...
Endotoxin_N
605. Bacteria__Dyadobacter_fermentans_WP_015811789.1
Non cytoplasmic domain
FAMILY NOT NAMED
delta-Endotoxin (insectocide), N-terminal domain
Endotoxin_N
606. Bacteria__Bacillus_thuringiensis_WP_014990538.1
delta-Endotoxin (insectocide), N-terminal domain
Endotoxin_N
FAMILY NOT NAMED
607. Bacteria__Bacillus_thuringiensis_WP_014990471.1
FAMILY NOT NAMED
delta-Endotoxin (insectocide), N-terminal domain
Endotoxin_N
608. Bacteria__Bacillus_thuringiensis_WP_013555126.1
delta-Endotoxin (insectocide), N-terminal domain
FAMILY NOT NAMED
C
Endotoxin_N
609. Bacteria__Xenorhabdus_nematophila_WP_013141600.1
Endotoxin_N
delta-Endotoxin (insectocide), N-terminal domain
FAMILY NOT NAMED
610. Bacteria__Shewanella_violacea_WP_013050437.1
delta-Endotoxin (insectocide), N-terminal domain
Endotoxin_N
611. Bacteria__Cupriavidus_pinatubonensis_WP_011296566.1
Non cytoplasmic domain
FAMILY NOT NAMED
Endotoxin_N
delta-Endotoxin (insectocide), N-terminal domain
612. Bacteria__Bacillus_thuringiensis_WP_001084998.1
delta-Endotoxin (insectocide), N-terminal domain
Endotoxin_N
unk...
Coil
613. Bacteria__Bacillus_cereus_WP_001063906.1
delta-Endotoxin (insectocide), N-terminal domain
FAMILY NOT NAMED
Endotoxin_N
614. Bacteria__Bacillus_thuringiensis_WP_000453539.1
delta-Endotoxin (insectocide), N-terminal domain
Endotoxin_N
FAMILY NOT NAMED

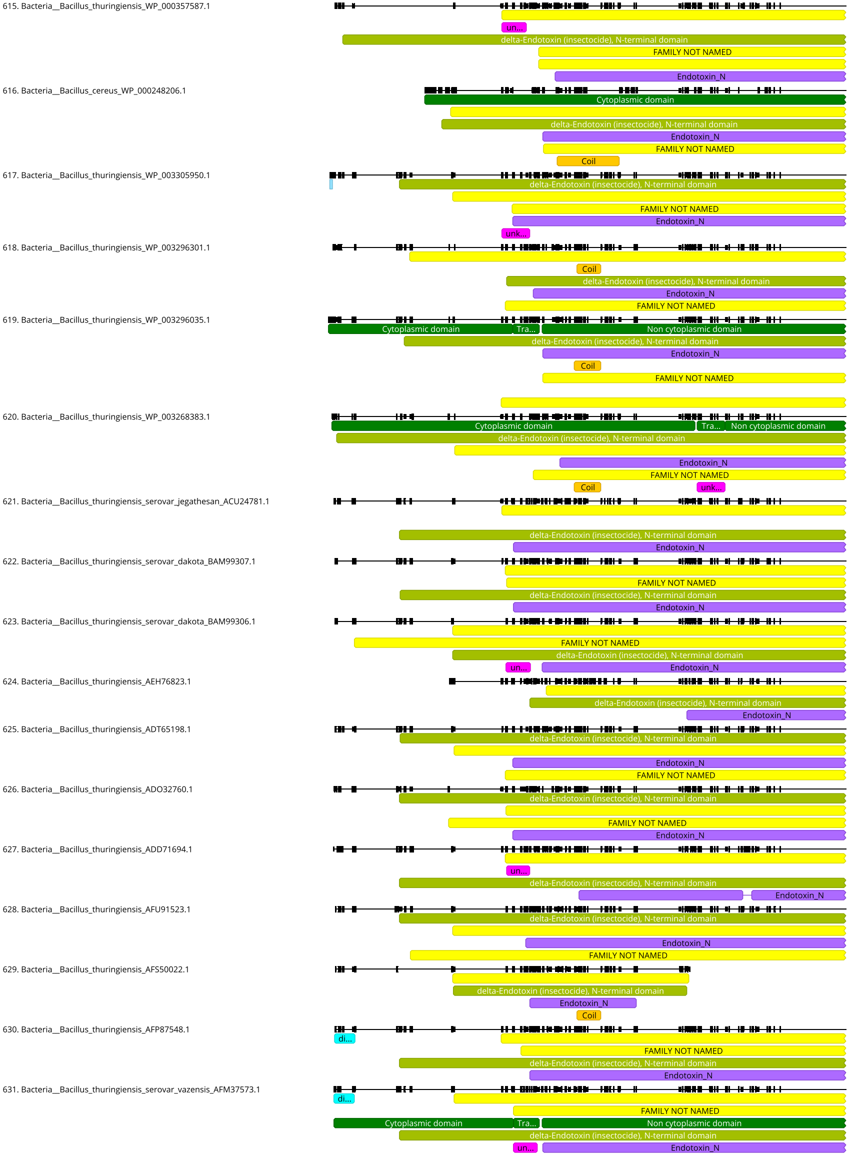
615. Bacteria__Bacillus_thuringiensis_WP_000357587.1
un...
delta-Endotoxin (insectocide), N-terminal domain
FAMILY NOT NAMED
Endotoxin_N
616. Bacteria__Bacillus_cereus_WP_000248206.1
Cytoplasmic domain
delta-Endotoxin (insectocide), N-terminal domain
Endotoxin_N
FAMILY NOT NAMED
Coil
617. Bacteria__Bacillus_thuringiensis_WP_003305950.1
delta-Endotoxin (insectocide), N-terminal domain
FAMILY NOT NAMED
Endotoxin_N
unk...
618. Bacteria__Bacillus_thuringiensis_WP_003296301.1
Coil
delta-Endotoxin (insectocide), N-terminal domain
Endotoxin_N
FAMILY NOT NAMED
619. Bacteria__Bacillus_thuringiensis_WP_003296035.1
Cytoplasmic domain
Tra...
Non cytoplasmic domain
delta-Endotoxin (insectocide), N-terminal domain
Endotoxin_N
Coil
FAMILY NOT NAMED
620. Bacteria__Bacillus_thuringiensis_WP_003268383.1
Cytoplasmic domain
Tra...
Non cytoplasmic domain
delta-Endotoxin (insectocide), N-terminal domain
Endotoxin_N
FAMILY NOT NAMED
Coil
unk...
621. Bacteria__Bacillus_thuringiensis_serovar_jegathesan_ACU24781.1
delta-Endotoxin (insectocide), N-terminal domain
Endotoxin_N
622. Bacteria__Bacillus_thuringiensis_serovar_dakota_BAM99307.1
FAMILY NOT NAMED
delta-Endotoxin (insectocide), N-terminal domain
Endotoxin_N
623. Bacteria__Bacillus_thuringiensis_serovar_dakota_BAM99306.1
FAMILY NOT NAMED
delta-Endotoxin (insectocide), N-terminal domain
un...
Endotoxin_N
624. Bacteria__Bacillus_thuringiensis_AEH76823.1
delta-Endotoxin (insectocide), N-terminal domain
Endotoxin_N
625. Bacteria__Bacillus_thuringiensis_ADT65198.1
delta-Endotoxin (insectocide), N-terminal domain
Endotoxin_N
FAMILY NOT NAMED
626. Bacteria__Bacillus_thuringiensis_ADO32760.1
delta-Endotoxin (insectocide), N-terminal domain
FAMILY NOT NAMED
Endotoxin_N
627. Bacteria__Bacillus_thuringiensis_ADD71694.1
un...
delta-Endotoxin (insectocide), N-terminal domain
Endotoxin_N
628. Bacteria__Bacillus_thuringiensis_AFU91523.1
delta-Endotoxin (insectocide), N-terminal domain
Endotoxin_N
FAMILY NOT NAMED
629. Bacteria__Bacillus_thuringiensis_AFS50022.1
delta-Endotoxin (insectocide), N-terminal domain
Endotoxin_N
Coil
630. Bacteria__Bacillus_thuringiensis_AFP87548.1
di...
FAMILY NOT NAMED
delta-Endotoxin (insectocide), N-terminal domain
Endotoxin_N
631. Bacteria__Bacillus_thuringiensis_serovar_vazensis_AFM37573.1
di...
FAMILY NOT NAMED
Cytoplasmic domain
Tra...
Non cytoplasmic domain
delta-Endotoxin (insectocide), N-terminal domain
un...
Endotoxin_N

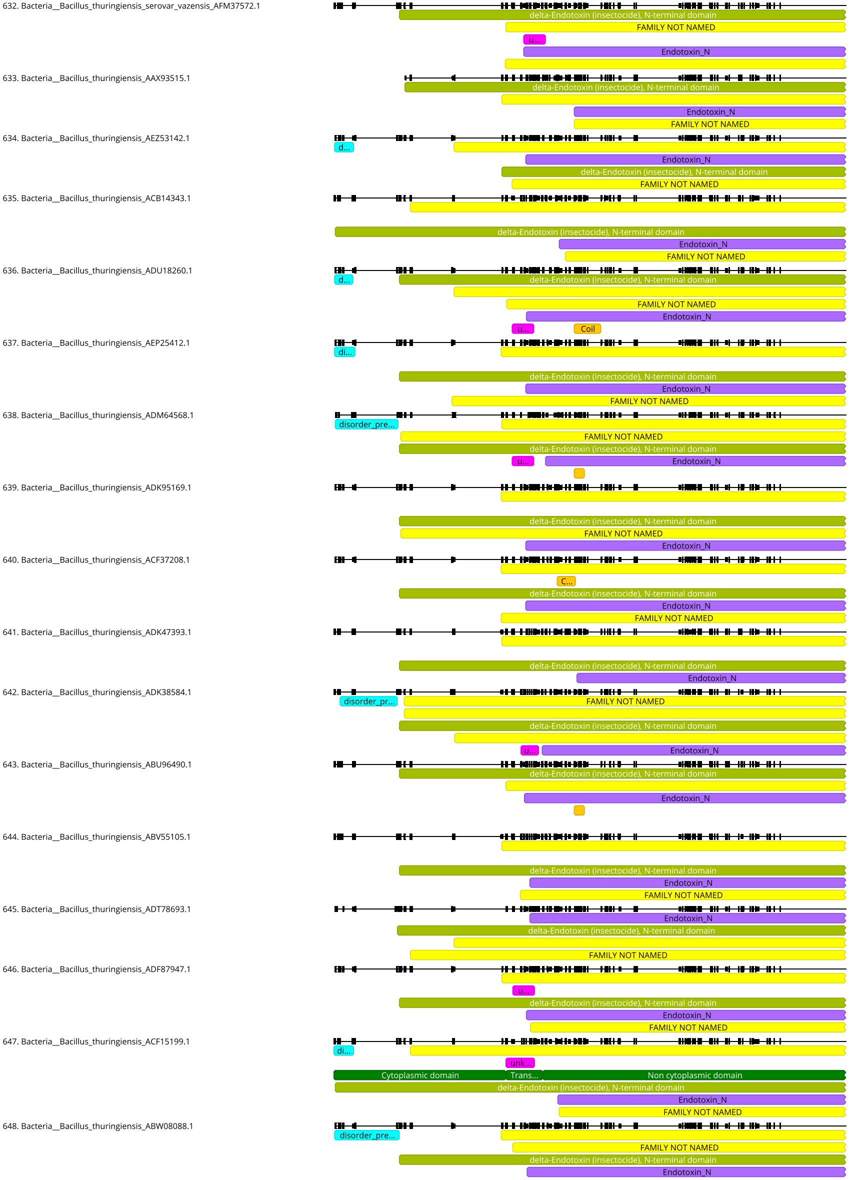
632. Bacteria__Bacillus_thuringiensis_serovar_vazensis_AFM37572.1
delta-Endotoxin (insectocide), N-terminal domain
FAMILY NOT NAMED
u...
Endotoxin_N
633. Bacteria__Bacillus_thuringiensis_AAX93515.1
delta-Endotoxin (insectocide), N-terminal domain
Endotoxin_N
FAMILY NOT NAMED
634. Bacteria__Bacillus_thuringiensis_AEZ53142.1
d...
Endotoxin_N
delta-Endotoxin (insectocide), N-terminal domain
FAMILY NOT NAMED
635. Bacteria__Bacillus_thuringiensis_ACB14343.1
delta-Endotoxin (insectocide), N-terminal domain
Endotoxin_N
FAMILY NOT NAMED
636. Bacteria__Bacillus_thuringiensis_ADU18260.1
d...
delta-Endotoxin (insectocide), N-terminal domain
FAMILY NOT NAMED
Endotoxin_N
u...
Coil
637. Bacteria__Bacillus_thuringiensis_AEP25412.1
di...
delta-Endotoxin (insectocide), N-terminal domain
Endotoxin_N
FAMILY NOT NAMED
638. Bacteria__Bacillus_thuringiensis_ADM64568.1
disorder_pre...
FAMILY NOT NAMED
delta-Endotoxin (insectocide), N-terminal domain
u...
Endotoxin_N
639. Bacteria__Bacillus_thuringiensis_ADK95169.1
delta-Endotoxin (insectocide), N-terminal domain
FAMILY NOT NAMED
Endotoxin_N
640. Bacteria__Bacillus_thuringiensis_ACF37208.1
C...
delta-Endotoxin (insectocide), N-terminal domain
Endotoxin_N
FAMILY NOT NAMED
641. Bacteria__Bacillus_thuringiensis_ADK47393.1
delta-Endotoxin (insectocide), N-terminal domain
Endotoxin_N
642. Bacteria__Bacillus_thuringiensis_ADK38584.1
disorder_pr...
FAMILY NOT NAMED
delta-Endotoxin (insectocide), N-terminal domain
u...
Endotoxin_N
643. Bacteria__Bacillus_thuringiensis_ABU96490.1
delta-Endotoxin (insectocide), N-terminal domain
Endotoxin_N
644. Bacteria__Bacillus_thuringiensis_ABV55105.1
delta-Endotoxin (insectocide), N-terminal domain
Endotoxin_N
FAMILY NOT NAMED
645. Bacteria__Bacillus_thuringiensis_ADT78693.1
Endotoxin_N
delta-Endotoxin (insectocide), N-terminal domain
FAMILY NOT NAMED
646. Bacteria__Bacillus_thuringiensis_ADF87947.1
u...
delta-Endotoxin (insectocide), N-terminal domain
Endotoxin_N
FAMILY NOT NAMED
647. Bacteria__Bacillus_thuringiensis_ACF15199.1
di...
unk...
Cytoplasmic domain
Trans...
Non cytoplasmic domain
delta-Endotoxin (insectocide), N-terminal domain
Endotoxin_N
FAMILY NOT NAMED
648. Bacteria__Bacillus_thuringiensis_ABW08088.1
disorder_pre...
FAMILY NOT NAMED
delta-Endotoxin (insectocide), N-terminal domain
Endotoxin_N

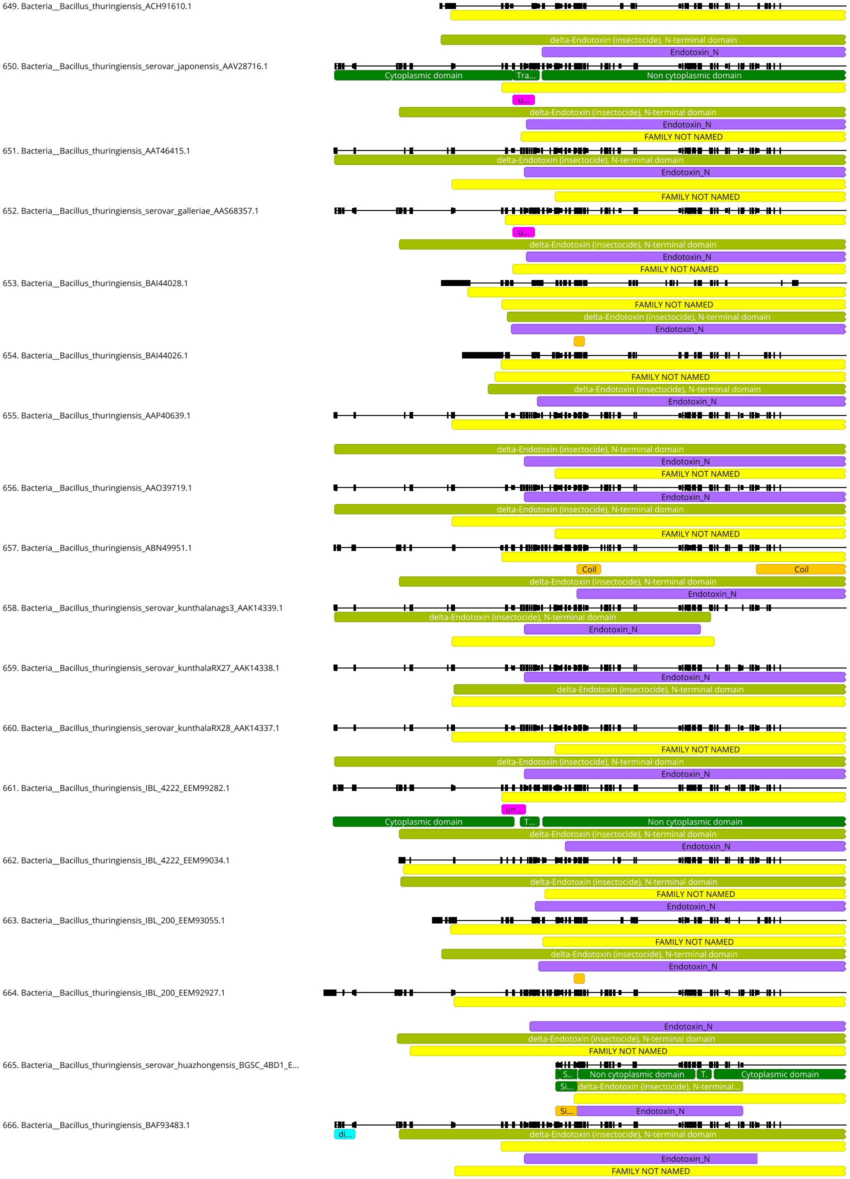
649. Bacteria__Bacillus_thuringiensis_ACH91610.1
delta-Endotoxin (insectocide), N-terminal domain
Endotoxin_N
650. Bacteria__Bacillus_thuringiensis_serovar_japonensis_AAV28716.1
Cytoplasmic domain
Tra...
Non cytoplasmic domain
u...
delta-Endotoxin (insectocide), N-terminal domain
Endotoxin_N
FAMILY NOT NAMED
651. Bacteria__Bacillus_thuringiensis_AAT46415.1
delta-Endotoxin (insectocide), N-terminal domain
Endotoxin_N
FAMILY NOT NAMED
652. Bacteria__Bacillus_thuringiensis_serovar_galleriae_AAS68357.1
u...
delta-Endotoxin (insectocide), N-terminal domain
Endotoxin_N
FAMILY NOT NAMED
653. Bacteria__Bacillus_thuringiensis_BAI44028.1
FAMILY NOT NAMED
delta-Endotoxin (insectocide), N-terminal domain
Endotoxin_N
654. Bacteria__Bacillus_thuringiensis_BAI44026.1
FAMILY NOT NAMED
delta-Endotoxin (insectocide), N-terminal domain
Endotoxin_N
655. Bacteria__Bacillus_thuringiensis_AAP40639.1
delta-Endotoxin (insectocide), N-terminal domain
Endotoxin_N
FAMILY NOT NAMED
656. Bacteria__Bacillus_thuringiensis_AAO39719.1
Endotoxin_N
delta-Endotoxin (insectocide), N-terminal domain
FAMILY NOT NAMED
657. Bacteria__Bacillus_thuringiensis_ABN49951.1
Coil
Coil
delta-Endotoxin (insectocide), N-terminal domain
Endotoxin_N
658. Bacteria__Bacillus_thuringiensis_serovar_kunthalanags3_AAK14339.1
delta-Endotoxin (insectocide), N-terminal domain
Endotoxin_N
659. Bacteria__Bacillus_thuringiensis_serovar_kunthalaRX27_AAK14338.1
Endotoxin_N
delta-Endotoxin (insectocide), N-terminal domain
660. Bacteria__Bacillus_thuringiensis_serovar_kunthalaRX28_AAK14337.1
FAMILY NOT NAMED
delta-Endotoxin (insectocide), N-terminal domain
Endotoxin_N
661. Bacteria__Bacillus_thuringiensis_IBL_4222_EEM99282.1
un...
Cytoplasmic domain
T...
Non cytoplasmic domain
delta-Endotoxin (insectocide), N-terminal domain
Endotoxin_N
662. Bacteria__Bacillus_thuringiensis_IBL_4222_EEM99034.1
delta-Endotoxin (insectocide), N-terminal domain
FAMILY NOT NAMED
Endotoxin_N
663. Bacteria__Bacillus_thuringiensis_IBL_200_EEM93055.1
FAMILY NOT NAMED
delta-Endotoxin (insectocide), N-terminal domain
Endotoxin_N
664. Bacteria__Bacillus_thuringiensis_IBL_200_EEM92927.1
Endotoxin_N
delta-Endotoxin (insectocide), N-terminal domain
FAMILY NOT NAMED
665. Bacteria__Bacillus_thuringiensis_serovar_huazhongensis_BGSC_4BD1_E...
S..
Non cytoplasmic domain
T.
Cytoplasmic domain
Si...
delta-Endotoxin (insectocide), N-terminal...
Si...
Endotoxin_N
666. Bacteria__Bacillus_thuringiensis_BAF93483.1
di...
delta-Endotoxin (insectocide), N-terminal domain
Endotoxin_N
FAMILY NOT NAMED

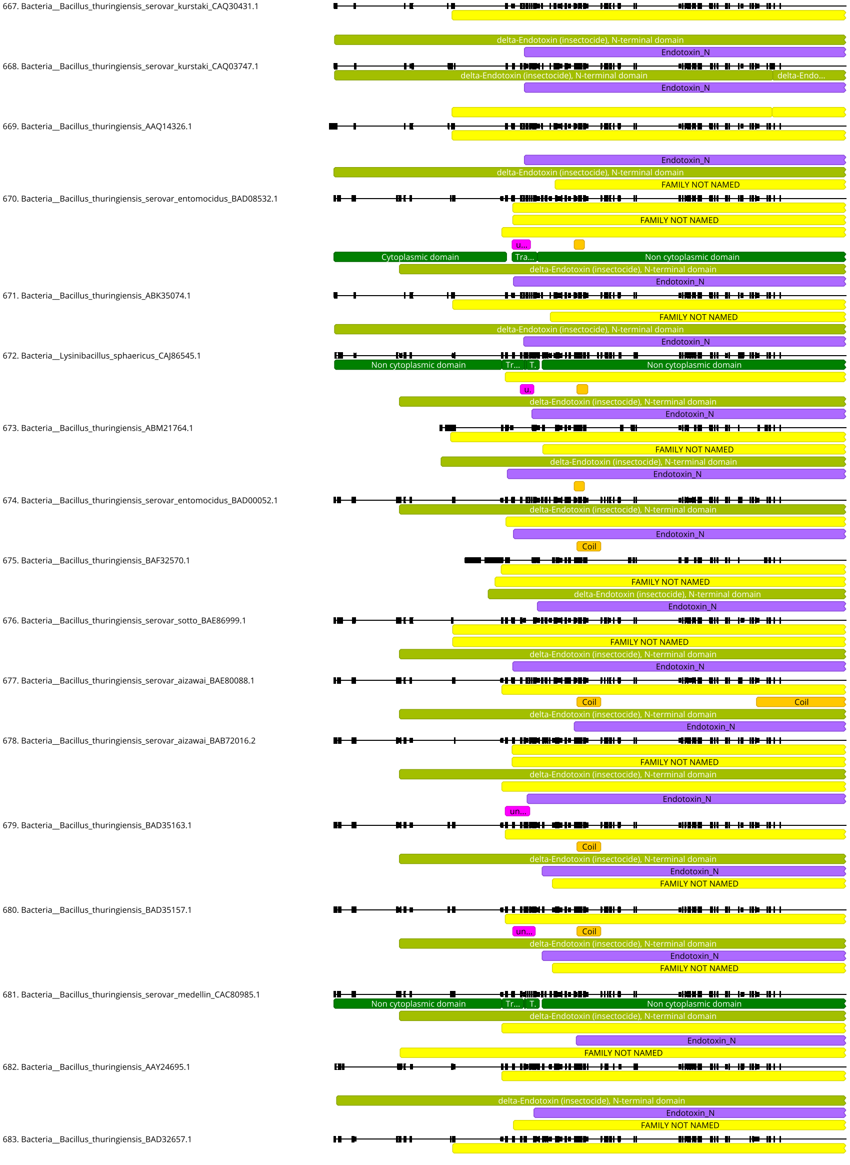

667. Bacteria__Bacillus_thuringiensis_serovar_kurstaki_CAQ30431.1
delta-Endotoxin (insectocide), N-terminal domain
Endotoxin_N
668. Bacteria__Bacillus_thuringiensis_serovar_kurstaki_CAQ03747.1
delta-Endotoxin (insectocide), N-terminal domain
delta-Endo...
Endotoxin_N
669. Bacteria__Bacillus_thuringiensis_AAQ14326.1
Endotoxin_N
delta-Endotoxin (insectocide), N-terminal domain
FAMILY NOT NAMED
670. Bacteria__Bacillus_thuringiensis_serovar_entomocidus_BAD08532.1
FAMILY NOT NAMED
u...
Cytoplasmic domain
Tra...
Non cytoplasmic domain
delta-Endotoxin (insectocide), N-terminal domain
Endotoxin_N
671. Bacteria__Bacillus_thuringiensis_ABK35074.1
FAMILY NOT NAMED
delta-Endotoxin (insectocide), N-terminal domain
Endotoxin_N
672. Bacteria__Lysinibacillus_sphaericus_CAJ86545.1
Non cytoplasmic domain
Tr...
T.
Non cytoplasmic domain
u.
delta-Endotoxin (insectocide), N-terminal domain
Endotoxin_N
673. Bacteria__Bacillus_thuringiensis_ABM21764.1
FAMILY NOT NAMED
delta-Endotoxin (insectocide), N-terminal domain
Endotoxin_N
674. Bacteria__Bacillus_thuringiensis_serovar_entomocidus_BAD00052.1
delta-Endotoxin (insectocide), N-terminal domain
Endotoxin_N
Coil
675. Bacteria__Bacillus_thuringiensis_BAF32570.1
FAMILY NOT NAMED
delta-Endotoxin (insectocide), N-terminal domain
Endotoxin_N
676. Bacteria__Bacillus_thuringiensis_serovar_sotto_BAE86999.1
FAMILY NOT NAMED
delta-Endotoxin (insectocide), N-terminal domain
Endotoxin_N
677. Bacteria__Bacillus_thuringiensis_serovar_aizawai_BAE80088.1
Coil
Coil
delta-Endotoxin (insectocide), N-terminal domain
Endotoxin_N
678. Bacteria__Bacillus_thuringiensis_serovar_aizawai_BAB72016.2
FAMILY NOT NAMED
delta-Endotoxin (insectocide), N-terminal domain
Endotoxin_N
un...
679. Bacteria__Bacillus_thuringiensis_BAD35163.1
Coil
delta-Endotoxin (insectocide), N-terminal domain
Endotoxin_N
FAMILY NOT NAMED
680. Bacteria__Bacillus_thuringiensis_BAD35157.1
un...
Coil
delta-Endotoxin (insectocide), N-terminal domain
Endotoxin_N
FAMILY NOT NAMED
681. Bacteria__Bacillus_thuringiensis_serovar_medellin_CAC80985.1
Non cytoplasmic domain
Tr...
T.
Non cytoplasmic domain
delta-Endotoxin (insectocide), N-terminal domain
Endotoxin_N
FAMILY NOT NAMED
682. Bacteria__Bacillus_thuringiensis_AAY24695.1
delta-Endotoxin (insectocide), N-terminal domain
Endotoxin_N
FAMILY NOT NAMED
683. Bacteria__Bacillus_thuringiensis_BAD32657.1

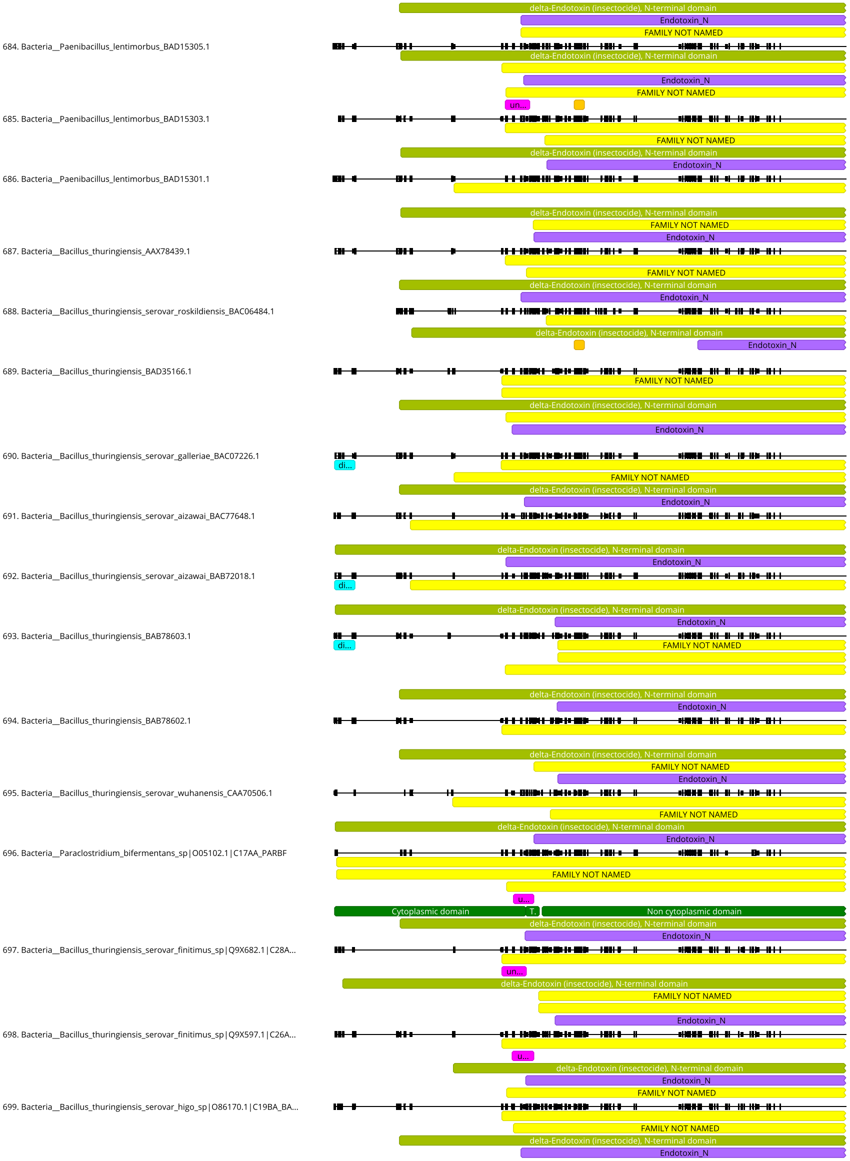

delta-Endotoxin (insectocide), N-terminal domain
Endotoxin_N
FAMILY NOT NAMED
684. Bacteria__Paenibacillus_lentimorbus_BAD15305.1
delta-Endotoxin (insectocide), N-terminal domain
Endotoxin_N
FAMILY NOT NAMED
un...
685. Bacteria__Paenibacillus_lentimorbus_BAD15303.1
FAMILY NOT NAMED
delta-Endotoxin (insectocide), N-terminal domain
Endotoxin_N
686. Bacteria__Paenibacillus_lentimorbus_BAD15301.1
delta-Endotoxin (insectocide), N-terminal domain
FAMILY NOT NAMED
Endotoxin_N
687. Bacteria__Bacillus_thuringiensis_AAX78439.1
FAMILY NOT NAMED
delta-Endotoxin (insectocide), N-terminal domain
Endotoxin_N
688. Bacteria__Bacillus_thuringiensis_serovar_roskildiensis_BAC06484.1
delta-Endotoxin (insectocide), N-terminal domain
Endotoxin_N
689. Bacteria__Bacillus_thuringiensis_BAD35166.1
FAMILY NOT NAMED
delta-Endotoxin (insectocide), N-terminal domain
Endotoxin_N
690. Bacteria__Bacillus_thuringiensis_serovar_galleriae_BAC07226.1
di...
FAMILY NOT NAMED
delta-Endotoxin (insectocide), N-terminal domain
Endotoxin_N
691. Bacteria__Bacillus_thuringiensis_serovar_aizawai_BAC77648.1
delta-Endotoxin (insectocide), N-terminal domain
Endotoxin_N
692. Bacteria__Bacillus_thuringiensis_serovar_aizawai_BAB72018.1
di...
delta-Endotoxin (insectocide), N-terminal domain
Endotoxin_N
693. Bacteria__Bacillus_thuringiensis_BAB78603.1
di...
FAMILY NOT NAMED
delta-Endotoxin (insectocide), N-terminal domain
Endotoxin_N
694. Bacteria__Bacillus_thuringiensis_BAB78602.1
delta-Endotoxin (insectocide), N-terminal domain
FAMILY NOT NAMED
Endotoxin_N
695. Bacteria__Bacillus_thuringiensis_serovar_wuhanensis_CAA70506.1
FAMILY NOT NAMED
delta-Endotoxin (insectocide), N-terminal domain
Endotoxin_N
696. Bacteria__Paraclostridium_bifermentans_sp|O05102.1|C17AA_PARBF
FAMILY NOT NAMED
u...
Cytoplasmic domain
T.
Non cytoplasmic domain
delta-Endotoxin (insectocide), N-terminal domain
Endotoxin_N
697. Bacteria__Bacillus_thuringiensis_serovar_finitimus_sp|Q9X682.1|C28A...
un...
delta-Endotoxin (insectocide), N-terminal domain
FAMILY NOT NAMED
Endotoxin_N
698. Bacteria__Bacillus_thuringiensis_serovar_finitimus_sp|Q9X597.1|C26A...
u...
delta-Endotoxin (insectocide), N-terminal domain
Endotoxin_N
FAMILY NOT NAMED
699. Bacteria__Bacillus_thuringiensis_serovar_higo_sp|O86170.1|C19BA_BA...
FAMILY NOT NAMED
delta-Endotoxin (insectocide), N-terminal domain
Endotoxin_N

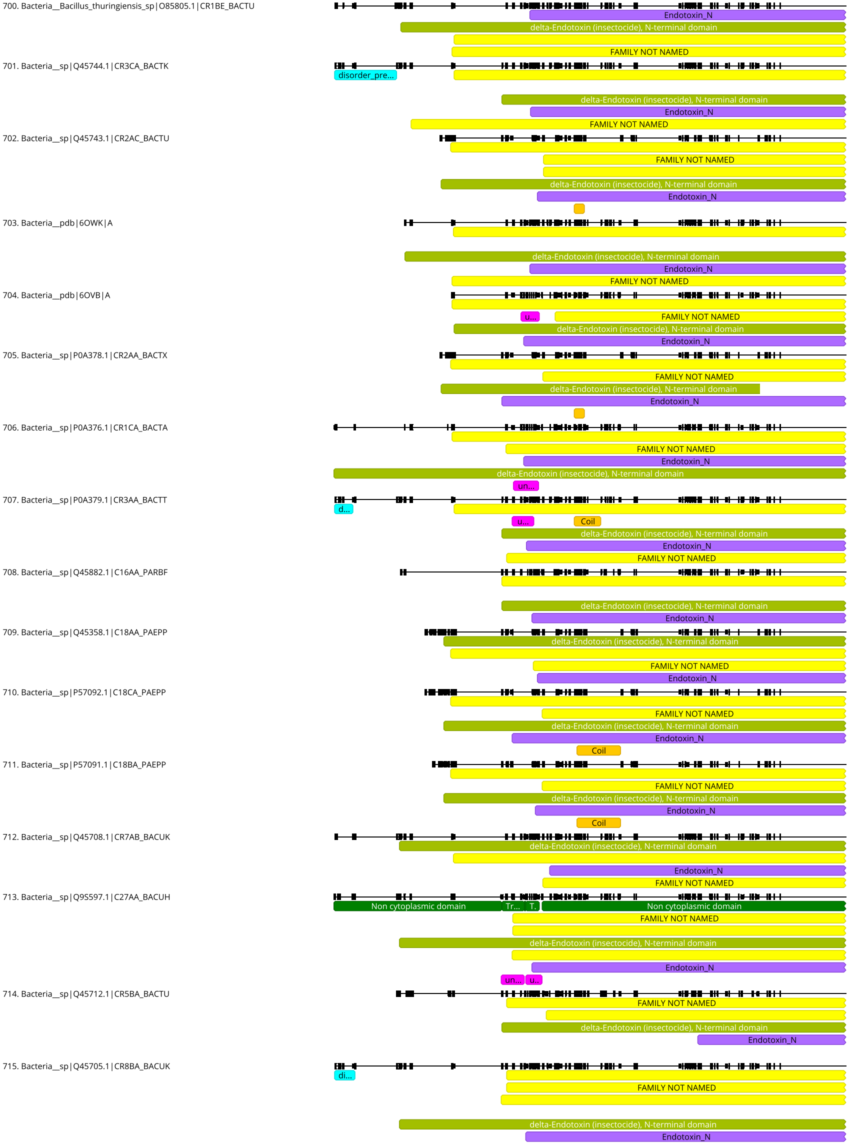
700. Bacteria__Bacillus_thuringiensis_sp|O85805.1|CR1BE_BACTU
Endotoxin_N
delta-Endotoxin (insecticide), N-terminal domain
FAMILY NOT NAMED
701. Bacteria__sp|Q45744.1|CR3CA_BACTK
disorder_pre...
delta-Endotoxin (insecticide), N-terminal domain
Endotoxin_N
FAMILY NOT NAMED
702. Bacteria__sp|Q45743.1|CR2AC_BACTU
FAMILY NOT NAMED
delta-Endotoxin (insecticide), N-terminal domain
Endotoxin_N
703. Bacteria__pdb|6OWK|A
delta-Endotoxin (insecticide), N-terminal domain
Endotoxin_N
FAMILY NOT NAMED
704. Bacteria__pdb|6OVB|A
u...
FAMILY NOT NAMED
delta-Endotoxin (insecticide), N-terminal domain
Endotoxin_N
705. Bacteria__sp|P0A378.1|CR2AA_BACTX
FAMILY NOT NAMED
delta-Endotoxin (insecticide), N-terminal domain
Endotoxin_N
706. Bacteria__sp|P0A376.1|CR1CA_BACTA
FAMILY NOT NAMED
Endotoxin_N
delta-Endotoxin (insecticide), N-terminal domain
un...
707. Bacteria__sp|P0A379.1|CR3AA_BACTT
d...
u...
Coil
delta-Endotoxin (insecticide), N-terminal domain
Endotoxin_N
FAMILY NOT NAMED
708. Bacteria__sp|Q45882.1|C16AA_PARBF
delta-Endotoxin (insecticide), N-terminal domain
Endotoxin_N
709. Bacteria__sp|Q45358.1|C18AA_PAEPP
delta-Endotoxin (insecticide), N-terminal domain
FAMILY NOT NAMED
Endotoxin_N
710. Bacteria__sp|P57092.1|C18CA_PAEPP
FAMILY NOT NAMED
delta-Endotoxin (insecticide), N-terminal domain
Endotoxin_N
Coil
711. Bacteria__sp|P57091.1|C18BA_PAEPP
FAMILY NOT NAMED
delta-Endotoxin (insecticide), N-terminal domain
Endotoxin_N
Coil
712. Bacteria__sp|Q45708.1|CR7AB_BACUK
delta-Endotoxin (insecticide), N-terminal domain
Endotoxin_N
FAMILY NOT NAMED
713. Bacteria__sp|Q9S597.1|C27AA_BACUH
Non cytoplasmic domain
Tr...
T.
Non cytoplasmic domain
FAMILY NOT NAMED
delta-Endotoxin (insecticide), N-terminal domain
Endotoxin_N
un...
u..
714. Bacteria__sp|Q45712.1|CR5BA_BACTU
FAMILY NOT NAMED
delta-Endotoxin (insecticide), N-terminal domain
Endotoxin_N
715. Bacteria__sp|Q45705.1|CR8BA_BACUK
di...
FAMILY NOT NAMED
delta-Endotoxin (insecticide), N-terminal domain
Endotoxin_N

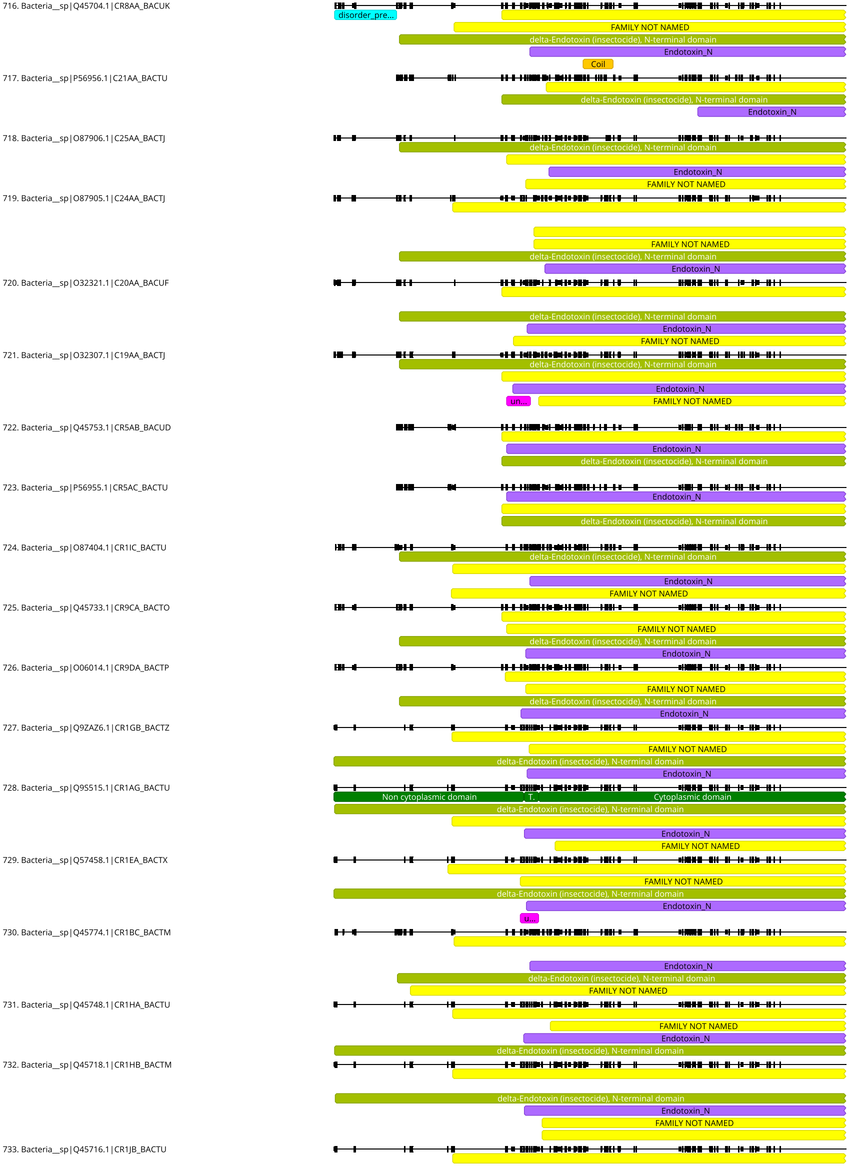
716. Bacteria__sp|Q45704.1|CR8AA_BACUK
disorder_pre...
FAMILY NOT NAMED
delta-Endotoxin (insecticide), N-terminal domain
Endotoxin_N
Coil
717. Bacteria__sp|P56956.1|C21AA_BACTU
delta-Endotoxin (insecticide), N-terminal domain
Endotoxin_N
718. Bacteria__sp|O87906.1|C25AA_BACTJ
delta-Endotoxin (insecticide), N-terminal domain
Endotoxin_N
FAMILY NOT NAMED
719. Bacteria__sp|O87905.1|C24AA_BACTJ
FAMILY NOT NAMED
delta-Endotoxin (insecticide), N-terminal domain
Endotoxin_N
720. Bacteria__sp|O32321.1|C20AA_BACUF
delta-Endotoxin (insecticide), N-terminal domain
Endotoxin_N
FAMILY NOT NAMED
721. Bacteria__sp|O32307.1|C19AA_BACTJ
delta-Endotoxin (insecticide), N-terminal domain
Endotoxin_N
un...
FAMILY NOT NAMED
722. Bacteria__sp|Q45753.1|CR5AB_BACUD
Endotoxin_N
delta-Endotoxin (insecticide), N-terminal domain
723. Bacteria__sp|P56955.1|CR5AC_BACTU
Endotoxin_N
delta-Endotoxin (insecticide), N-terminal domain
724. Bacteria__sp|O87404.1|CR1IC_BACTU
delta-Endotoxin (insecticide), N-terminal domain
Endotoxin_N
FAMILY NOT NAMED
725. Bacteria__sp|Q45733.1|CR9CA_BACTO
FAMILY NOT NAMED
delta-Endotoxin (insecticide), N-terminal domain
Endotoxin_N
726. Bacteria__sp|O06014.1|CR9DA_BACTP
FAMILY NOT NAMED
delta-Endotoxin (insecticide), N-terminal domain
Endotoxin_N
727. Bacteria__sp|Q9ZAZ6.1|CR1GB_BACTZ
FAMILY NOT NAMED
delta-Endotoxin (insecticide), N-terminal domain
Endotoxin_N
728. Bacteria__sp|Q9S515.1|CR1AG_BACTU
Non cytoplasmic domain
T.
Cytoplasmic domain
delta-Endotoxin (insecticide), N-terminal domain
Endotoxin_N
FAMILY NOT NAMED
729. Bacteria__sp|Q57458.1|CR1EA_BACTX
FAMILY NOT NAMED
delta-Endotoxin (insecticide), N-terminal domain
Endotoxin_N
u...
730. Bacteria__sp|Q45774.1|CR1BC_BACTM
Endotoxin_N
delta-Endotoxin (insecticide), N-terminal domain
FAMILY NOT NAMED
731. Bacteria__sp|Q45748.1|CR1HA_BACTU
FAMILY NOT NAMED
Endotoxin_N
delta-Endotoxin (insecticide), N-terminal domain
732. Bacteria__sp|Q45718.1|CR1HB_BACTM
delta-Endotoxin (insecticide), N-terminal domain
Endotoxin_N
FAMILY NOT NAMED
733. Bacteria__sp|Q45716.1|CR1JB_BACTU

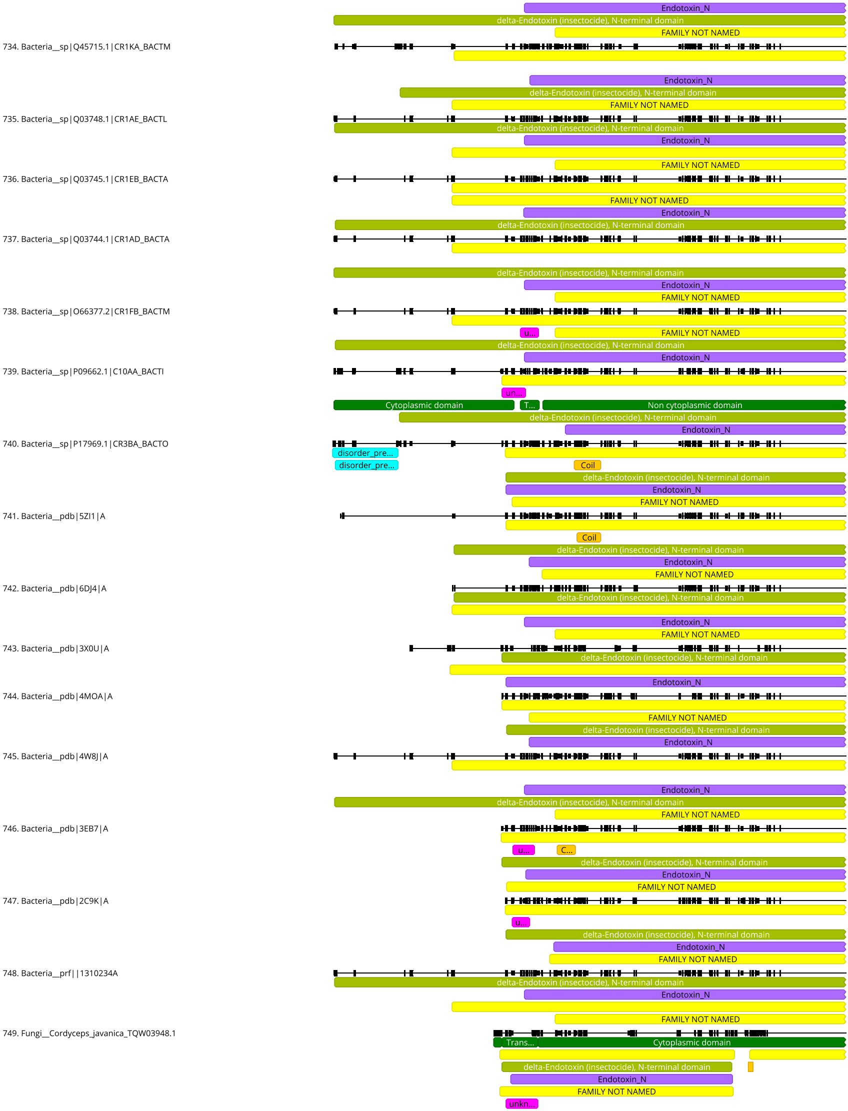

Endotoxin_N
delta-Endotoxin (insectocide), N-terminal domain
FAMILY NOT NAMED
734. Bacteria__sp|Q45715.1|CR1KA_BACTM
Endotoxin_N
delta-Endotoxin (insectocide), N-terminal domain
FAMILY NOT NAMED
735. Bacteria__sp|Q03748.1|CR1AE_BACTL
delta-Endotoxin (insectocide), N-terminal domain
Endotoxin_N
FAMILY NOT NAMED
736. Bacteria__sp|Q03745.1|CR1EB_BACTA
FAMILY NOT NAMED
Endotoxin_N
delta-Endotoxin (insectocide), N-terminal domain
737. Bacteria__sp|Q03744.1|CR1AD_BACTA
delta-Endotoxin (insectocide), N-terminal domain
Endotoxin_N
FAMILY NOT NAMED
738. Bacteria__sp|O66377.2|CR1FB_BACTM
u...
FAMILY NOT NAMED
delta-Endotoxin (insectocide), N-terminal domain
Endotoxin_N
739. Bacteria__sp|P09662.1|C10AA_BACTI
un...
Cytoplasmic domain
T...
Non cytoplasmic domain
delta-Endotoxin (insectocide), N-terminal domain
Endotoxin_N
740. Bacteria__sp|P17969.1|CR3BA_BACTO
disorder_pre...
disorder_pre...
Coil
delta-Endotoxin (insectocide), N-terminal domain
Endotoxin_N
FAMILY NOT NAMED
741. Bacteria__pdb|5ZI1|A
Coil
delta-Endotoxin (insectocide), N-terminal domain
Endotoxin_N
FAMILY NOT NAMED
742. Bacteria__pdb|6DJ4|A
delta-Endotoxin (insectocide), N-terminal domain
Endotoxin_N
FAMILY NOT NAMED
743. Bacteria__pdb|3X0U|A
delta-Endotoxin (insectocide), N-terminal domain
Endotoxin_N
744. Bacteria__pdb|4MOA|A
FAMILY NOT NAMED
delta-Endotoxin (insectocide), N-terminal domain
Endotoxin_N
745. Bacteria__pdb|4W8J|A
Endotoxin_N
delta-Endotoxin (insectocide), N-terminal domain
FAMILY NOT NAMED
746. Bacteria__pdb|3EB7|A
u...
C...
delta-Endotoxin (insectocide), N-terminal domain
Endotoxin_N
FAMILY NOT NAMED
747. Bacteria__pdb|2C9K|A
u...
delta-Endotoxin (insectocide), N-terminal domain
Endotoxin_N
FAMILY NOT NAMED
748. Bacteria__prf||1310234A
delta-Endotoxin (insectocide), N-terminal domain
Endotoxin_N
FAMILY NOT NAMED
749. Fungi__Cordyceps_javanica_TQW03948.1
Trans...
Cytoplasmic domain
delta-Endotoxin (insectocide), N-terminal domain
Endotoxin_N
FAMILY NOT NAMED
unkn...

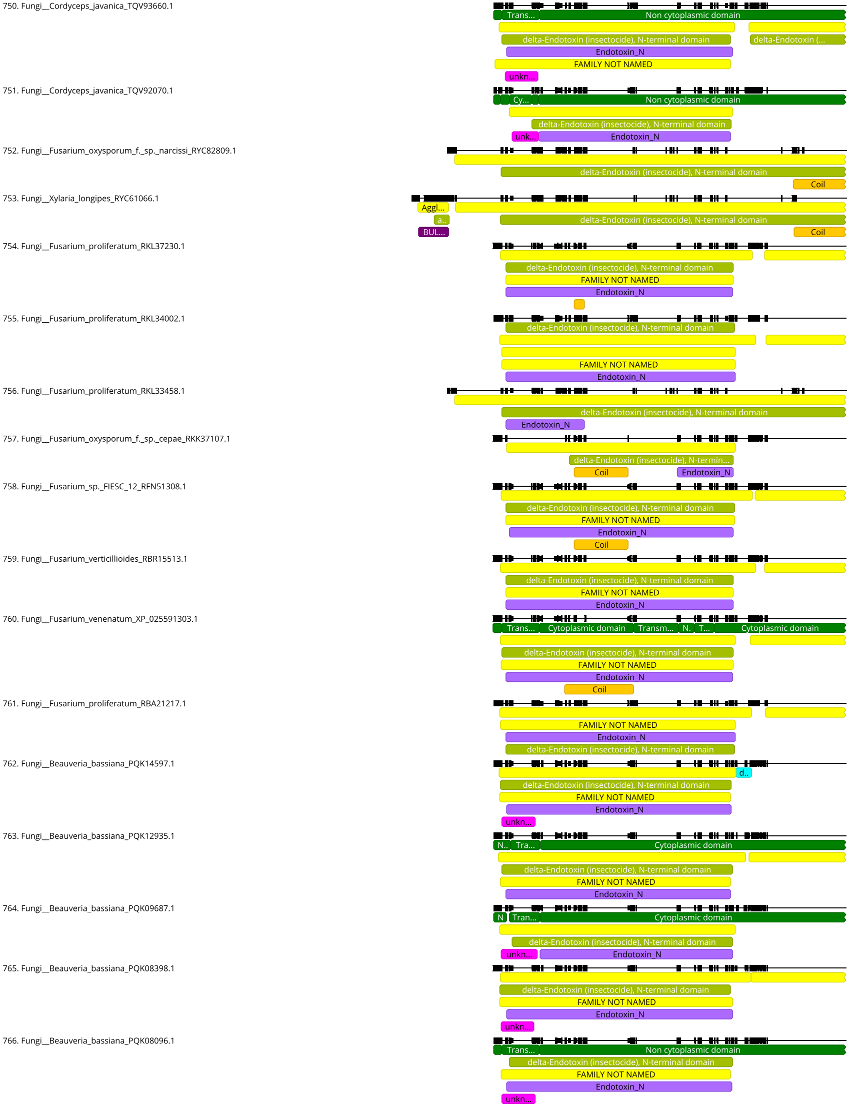
750. Fungi__Cordyceps_javanica_TQV93660.1
Trans...
Non cytoplasmic domain
delta-Endotoxin (insectocide), N-terminal domain
delta-Endotoxin (...
Endotoxin_N
FAMILY NOT NAMED
unkn...
751. Fungi__Cordyceps_javanica_TQV92070.1
Cy...
Non cytoplasmic domain
delta-Endotoxin (insectocide), N-terminal domain
unk...
Endotoxin_N
752. Fungi__Fusarium_oxysporum_f._sp._narcissi_RYC82809.1
delta-Endotoxin (insectocide), N-terminal domain
Coil
753. Fungi__Xylaria_longipes_RYC61066.1
Aggl...
a..
delta-Endotoxin (insectocide), N-terminal domain
BUL...
Coil
754. Fungi__Fusarium_proliferatum_RKL37230.1
delta-Endotoxin (insectocide), N-terminal domain
FAMILY NOT NAMED
Endotoxin_N
755. Fungi__Fusarium_proliferatum_RKL34002.1
delta-Endotoxin (insectocide), N-terminal domain
FAMILY NOT NAMED
Endotoxin_N
756. Fungi__Fusarium_proliferatum_RKL33458.1
delta-Endotoxin (insectocide), N-terminal domain
Endotoxin_N
757. Fungi__Fusarium_oxysporum_f._sp._cepae_RKK37107.1
delta-Endotoxin (insectocide), N-termin...
Coil
Endotoxin_N
758. Fungi__Fusarium_sp._FIESC_12_RFN51308.1
delta-Endotoxin (insectocide), N-terminal domain
FAMILY NOT NAMED
Endotoxin_N
Coil
759. Fungi__Fusarium_verticillioides_RBR15513.1
delta-Endotoxin (insectocide), N-terminal domain
FAMILY NOT NAMED
Endotoxin_N
760. Fungi__Fusarium_venenatum_XP_025591303.1
Trans...
Cytoplasmic domain
Transm...
N.
T...
Cytoplasmic domain
delta-Endotoxin (insectocide), N-terminal domain
FAMILY NOT NAMED
Endotoxin_N
Coil
761. Fungi__Fusarium_proliferatum_RBA21217.1
FAMILY NOT NAMED
Endotoxin_N
delta-Endotoxin (insectocide), N-terminal domain
762. Fungi__Beauveria_bassiana_PQK14597.1
d..
delta-Endotoxin (insectocide), N-terminal domain
FAMILY NOT NAMED
Endotoxin_N
unkn...
763. Fungi__Beauveria_bassiana_PQK12935.1
N..
Tra...
Cytoplasmic domain
delta-Endotoxin (insectocide), N-terminal domain
FAMILY NOT NAMED
Endotoxin_N
764. Fungi__Beauveria_bassiana_PQK09687.1
N
Tran...
Cytoplasmic domain
delta-Endotoxin (insectocide), N-terminal domain
unkn...
Endotoxin_N
765. Fungi__Beauveria_bassiana_PQK08398.1
delta-Endotoxin (insectocide), N-terminal domain
FAMILY NOT NAMED
Endotoxin_N
unkn...
766. Fungi__Beauveria_bassiana_PQK08096.1
Trans...
Non cytoplasmic domain
delta-Endotoxin (insectocide), N-terminal domain
FAMILY NOT NAMED
Endotoxin_N
unkn...

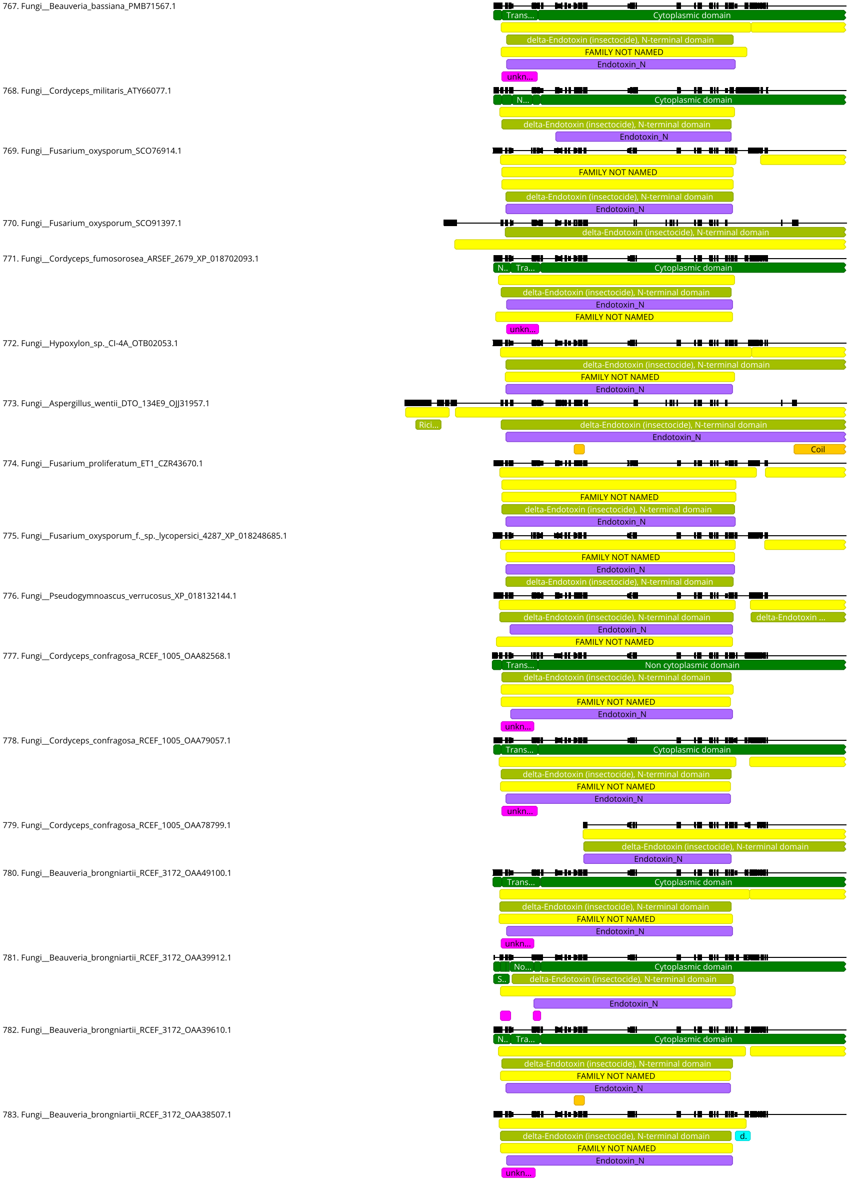
767. Fungi__Beauveria_bassiana_PMB71567.1
Trans...
Cytoplasmic domain
delta-Endotoxin (insectocide), N-terminal domain
FAMILY NOT NAMED
Endotoxin_N
unkn...
768. Fungi__Cordyceps_militaris_ATY66077.1
N...
Cytoplasmic domain
delta-Endotoxin (insectocide), N-terminal domain
Endotoxin_N
769. Fungi__Fusarium_oxysporum_SCO76914.1
FAMILY NOT NAMED
delta-Endotoxin (insectocide), N-terminal domain
Endotoxin_N
770. Fungi__Fusarium_oxysporum_SCO91397.1
delta-Endotoxin (insectocide), N-terminal domain
771. Fungi__Cordyceps_fumosorosea_ARSEF_2679_XP_018702093.1
N..
Tra...
Cytoplasmic domain
delta-Endotoxin (insectocide), N-terminal domain
Endotoxin_N
FAMILY NOT NAMED
unkn...
772. Fungi__Hypoxylon_sp._CI-4A_OTB02053.1
delta-Endotoxin (insectocide), N-terminal domain
FAMILY NOT NAMED
Endotoxin_N
773. Fungi__Aspergillus_wentii_DTO_134E9_OJJ31957.1
Rici...
delta-Endotoxin (insectocide), N-terminal domain
Endotoxin_N
Coil
774. Fungi__Fusarium_proliferatum_ET1_CZR43670.1
FAMILY NOT NAMED
delta-Endotoxin (insectocide), N-terminal domain
Endotoxin_N
775. Fungi__Fusarium_oxysporum_f._sp._lycopersici_4287_XP_018248685.1
FAMILY NOT NAMED
Endotoxin_N
delta-Endotoxin (insectocide), N-terminal domain
776. Fungi__Pseudogymnoascus_verrucosus_XP_018132144.1
delta-Endotoxin (insectocide), N-terminal domain
delta-Endotoxin ...
Endotoxin_N
FAMILY NOT NAMED
777. Fungi__Cordyceps_confragosa_RCEF_1005_OAA82568.1
Trans...
Non cytoplasmic domain
delta-Endotoxin (insectocide), N-terminal domain
FAMILY NOT NAMED
Endotoxin_N
unkn...
778. Fungi__Cordyceps_confragosa_RCEF_1005_OAA79057.1
Trans...
Cytoplasmic domain
delta-Endotoxin (insectocide), N-terminal domain
FAMILY NOT NAMED
Endotoxin_N
unkn...
779. Fungi__Cordyceps_confragosa_RCEF_1005_OAA78799.1
delta-Endotoxin (insectocide), N-terminal domain
Endotoxin_N
780. Fungi__Beauveria_brongniartii_RCEF_3172_OAA49100.1
Trans...
Cytoplasmic domain
delta-Endotoxin (insectocide), N-terminal domain
FAMILY NOT NAMED
Endotoxin_N
unkn...
781. Fungi__Beauveria_brongniartii_RCEF_3172_OAA39912.1
No...
Cytoplasmic domain
S..
delta-Endotoxin (insectocide), N-terminal domain
Endotoxin_N
782. Fungi__Beauveria_brongniartii_RCEF_3172_OAA39610.1
N..
Tra...
Cytoplasmic domain
delta-Endotoxin (insectocide), N-terminal domain
FAMILY NOT NAMED
Endotoxin_N
783. Fungi__Beauveria_brongniartii_RCEF_3172_OAA38507.1
delta-Endotoxin (insectocide), N-terminal domain
d.
FAMILY NOT NAMED
Endotoxin_N
unkn...

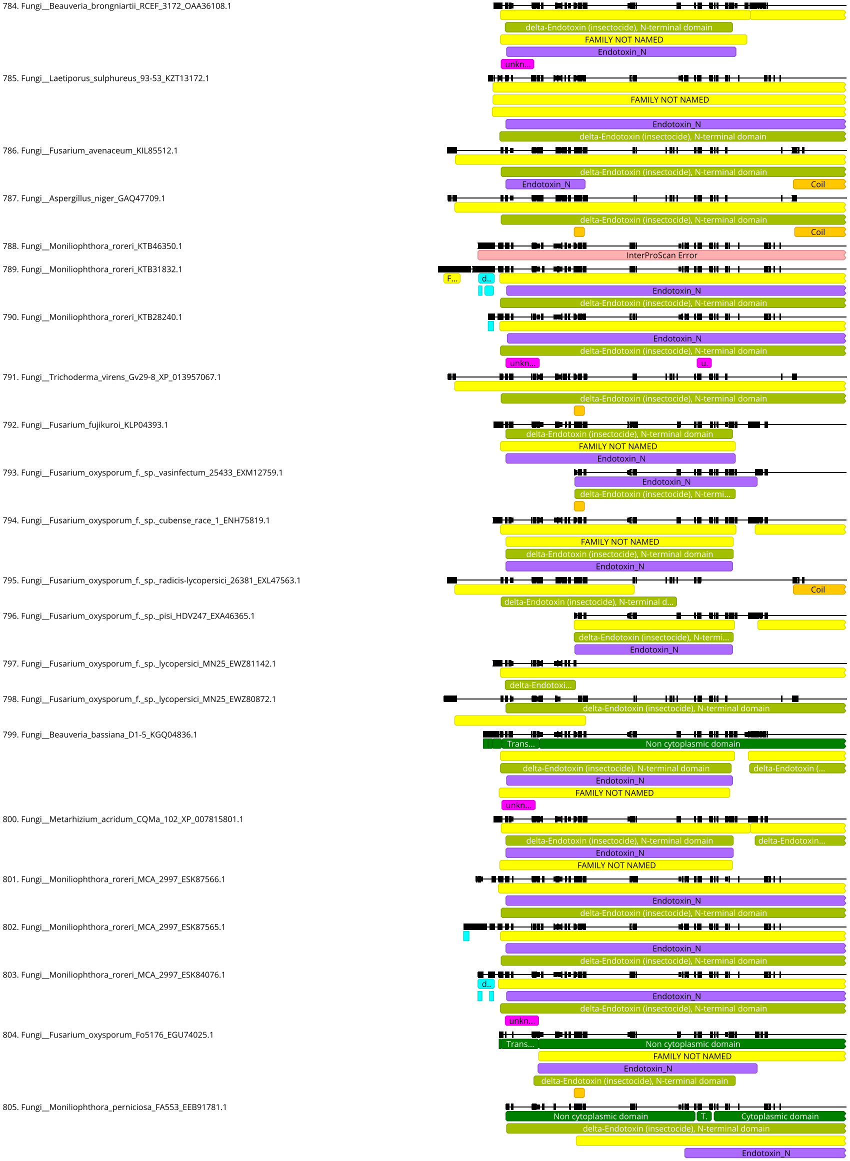

784. Fungi__Beauveria_brongniartii_RCEF_3172_OAA36108.1
delta-Endotoxin (insectocide), N-terminal domain
FAMILY NOT NAMED
Endotoxin_N
unkn...
785. Fungi__Laetiporus_sulphureus_93-53_KZT13172.1
FAMILY NOT NAMED
Endotoxin_N
delta-Endotoxin (insectocide), N-terminal domain
786. Fungi__Fusarium_avenaceum_KIL85512.1
delta-Endotoxin (insectocide), N-terminal domain
Endotoxin_N
Coil
787. Fungi__Aspergillus_niger_GAQ47709.1
delta-Endotoxin (insectocide), N-terminal domain
Coil
788. Fungi__Moniliophthora_roreri_KTB46350.1
InterProScan Error
789. Fungi__Moniliophthora_roreri_KTB31832.1
F...
d..
Endotoxin_N
delta-Endotoxin (insectocide), N-terminal domain
790. Fungi__Moniliophthora_roreri_KTB28240.1
Endotoxin_N
delta-Endotoxin (insectocide), N-terminal domain
unkn...
u.
791. Fungi__Trichoderma_virens_Gv29-8_XP_013957067.1
delta-Endotoxin (insectocide), N-terminal domain
792. Fungi__Fusarium_fujikuroi_KLP04393.1
delta-Endotoxin (insectocide), N-terminal domain
FAMILY NOT NAMED
Endotoxin_N
793. Fungi__Fusarium_oxysporum_f._sp._vasinfectum_25433_EXM12759.1
Endotoxin_N
delta-Endotoxin (insectocide), N-termi...
794. Fungi__Fusarium_oxysporum_f._sp._cubense_race_1_ENH75819.1
FAMILY NOT NAMED
delta-Endotoxin (insectocide), N-terminal domain
Endotoxin_N
795. Fungi__Fusarium_oxysporum_f._sp._radicis-lycopersici_26381_EXL47563.1
Coil
delta-Endotoxin (insectocide), N-terminal d...
796. Fungi__Fusarium_oxysporum_f._sp._pisi_HDV247_EXA46365.1
delta-Endotoxin (insectocide), N-termi...
Endotoxin_N
797. Fungi__Fusarium_oxysporum_f._sp._lycopersici_MN25_EWZ81142.1
delta-Endotoxi...
798. Fungi__Fusarium_oxysporum_f._sp._lycopersici_MN25_EWZ80872.1
delta-Endotoxin (insectocide), N-terminal domain
799. Fungi__Beauveria_bassiana_D1-5_KGQ04836.1
Trans...
Non cytoplasmic domain
delta-Endotoxin (insectocide), N-terminal domain
delta-Endotoxin (...
Endotoxin_N
FAMILY NOT NAMED
unkn...
800. Fungi__Metarhizium_acridum_CQMa_102_XP_007815801.1
delta-Endotoxin (insectocide), N-terminal domain
delta-Endotoxin...
Endotoxin_N
FAMILY NOT NAMED
801. Fungi__Moniliophthora_roreri_MCA_2997_ESK87566.1
Endotoxin_N
delta-Endotoxin (insectocide), N-terminal domain
802. Fungi__Moniliophthora_roreri_MCA_2997_ESK87565.1
Endotoxin_N
delta-Endotoxin (insectocide), N-terminal domain
803. Fungi__Moniliophthora_roreri_MCA_2997_ESK84076.1
d..
Endotoxin_N
delta-Endotoxin (insectocide), N-terminal domain
unkn...
804. Fungi__Fusarium_oxysporum_Fo5176_EGU74025.1
Trans...
Non cytoplasmic domain
FAMILY NOT NAMED
Endotoxin_N
delta-Endotoxin (insectocide), N-terminal domain
805. Fungi__Moniliophthora_perniciosa_FA553_EEB91781.1
Non cytoplasmic domain
T.
Cytoplasmic domain
delta-Endotoxin (insectocide), N-terminal domain
Endotoxin_N

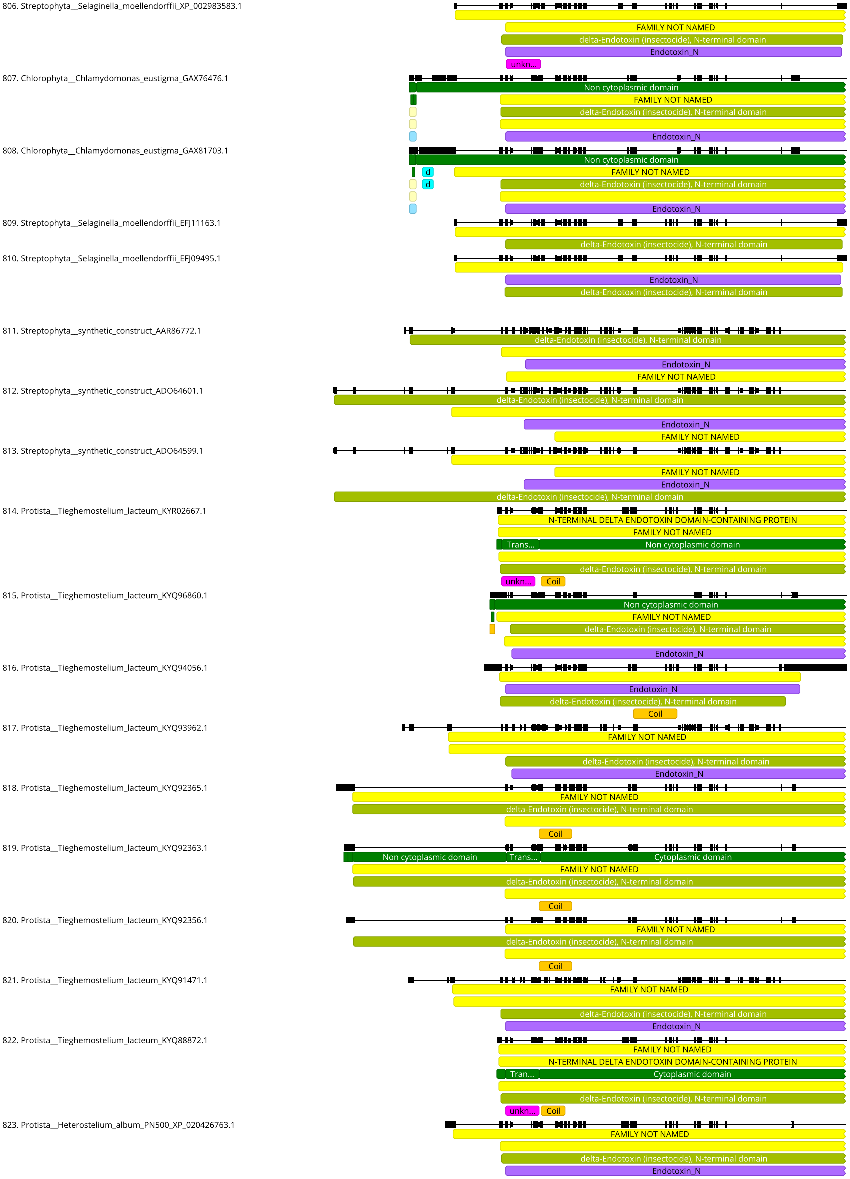
806. Streptophyta__Selaginella_moellendorffii_XP_002983583.1
FAMILY NOT NAMED
delta-Endotoxin (insectocide), N-terminal domain
Endotoxin_N
unkn...
807. Chlorophyta__Chlamydomonas_eustigma_GAX76476.1
Non cytoplasmic domain
FAMILY NOT NAMED
delta-Endotoxin (insectocide), N-terminal domain
Endotoxin_N
808. Chlorophyta__Chlamydomonas_eustigma_GAX81703.1
Non cytoplasmic domain
d
FAMILY NOT NAMED
d
delta-Endotoxin (insectocide), N-terminal domain
Endotoxin_N
809. Streptophyta__Selaginella_moellendorffii_EFJ11163.1
delta-Endotoxin (insectocide), N-terminal domain
810. Streptophyta__Selaginella_moellendorffii_EFJ09495.1
Endotoxin_N
delta-Endotoxin (insectocide), N-terminal domain
811. Streptophyta__synthetic_construct_AAR86772.1
delta-Endotoxin (insectocide), N-terminal domain
Endotoxin_N
FAMILY NOT NAMED
812. Streptophyta__synthetic_construct_ADO64601.1
delta-Endotoxin (insectocide), N-terminal domain
Endotoxin_N
FAMILY NOT NAMED
813. Streptophyta__synthetic_construct_ADO64599.1
FAMILY NOT NAMED
Endotoxin_N
delta-Endotoxin (insectocide), N-terminal domain
814. Protista__Tieghemostelium_lacteum_KYR02667.1
N-TERMINAL DELTA ENDOTOXIN DOMAIN-CONTAINING PROTEIN
FAMILY NOT NAMED
Trans...
Non cytoplasmic domain
delta-Endotoxin (insectocide), N-terminal domain
unkn...
Coil
815. Protista__Tieghemostelium_lacteum_KYQ96860.1
Non cytoplasmic domain
FAMILY NOT NAMED
delta-Endotoxin (insectocide), N-terminal domain
Endotoxin_N
816. Protista__Tieghemostelium_lacteum_KYQ94056.1
Endotoxin_N
delta-Endotoxin (insectocide), N-terminal domain
Coil
817. Protista__Tieghemostelium_lacteum_KYQ93962.1
FAMILY NOT NAMED
delta-Endotoxin (insectocide), N-terminal domain
Endotoxin_N
818. Protista__Tieghemostelium_lacteum_KYQ92365.1
FAMILY NOT NAMED
delta-Endotoxin (insectocide), N-terminal domain
Coil
819. Protista__Tieghemostelium_lacteum_KYQ92363.1
Non cytoplasmic domain
Trans...
Cytoplasmic domain
FAMILY NOT NAMED
delta-Endotoxin (insectocide), N-terminal domain
Coil
820. Protista__Tieghemostelium_lacteum_KYQ92356.1
FAMILY NOT NAMED
delta-Endotoxin (insectocide), N-terminal domain
Coil
821. Protista__Tieghemostelium_lacteum_KYQ91471.1
FAMILY NOT NAMED
delta-Endotoxin (insectocide), N-terminal domain
Endotoxin_N
822. Protista__Tieghemostelium_lacteum_KYQ88872.1
FAMILY NOT NAMED
N-TERMINAL DELTA ENDOTOXIN DOMAIN-CONTAINING PROTEIN
Tran...
Cytoplasmic domain
delta-Endotoxin (insectocide), N-terminal domain
unkn...
Coil
823. Protista__Heterostelium_album_PN500_XP_020426763.1
FAMILY NOT NAMED
delta-Endotoxin (insectocide), N-terminal domain
Endotoxin_N

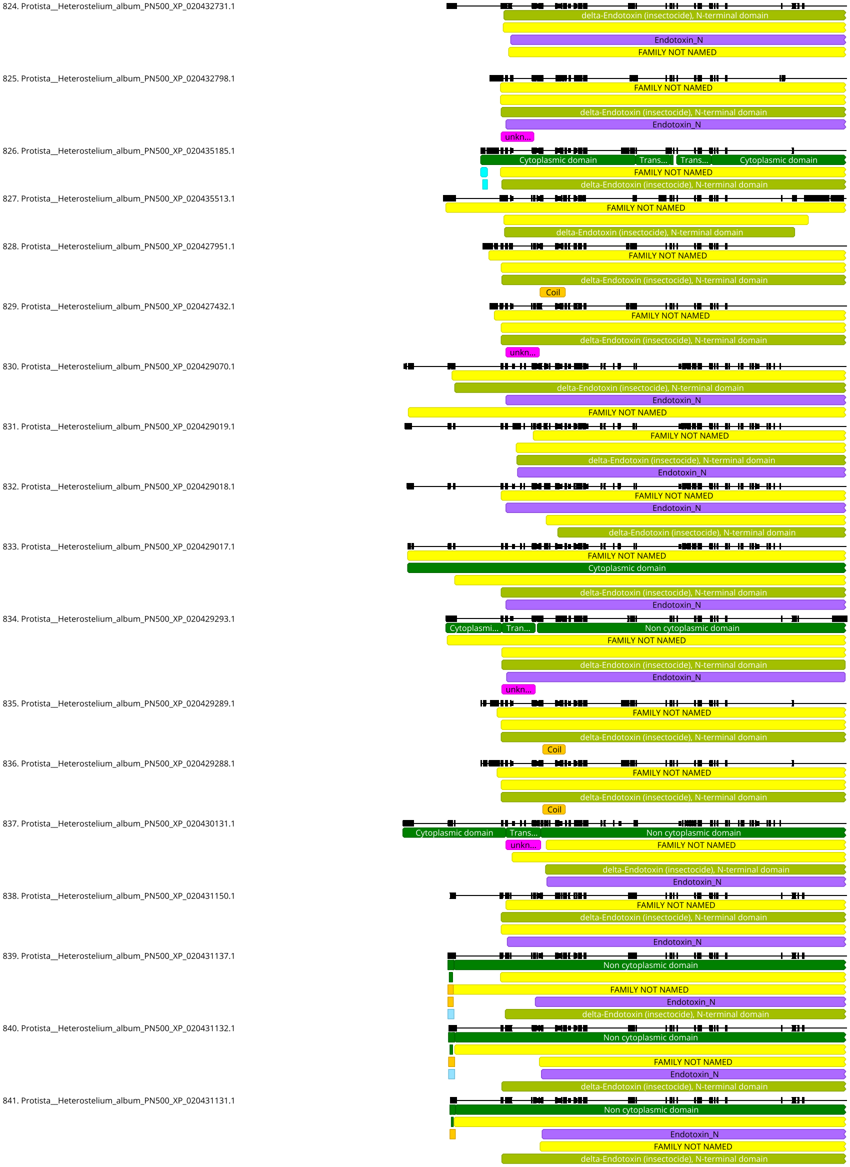
824. Protista__Heterostelium_album_PN500_XP_020432731.1
delta-Endotoxin (insectocide), N-terminal domain
Endotoxin_N
FAMILY NOT NAMED
825. Protista__Heterostelium_album_PN500_XP_020432798.1
FAMILY NOT NAMED
delta-Endotoxin (insectocide), N-terminal domain
Endotoxin_N
unkn...
826. Protista__Heterostelium_album_PN500_XP_020435185.1
Cytoplasmic domain
Trans...
Trans...
Cytoplasmic domain
FAMILY NOT NAMED
delta-Endotoxin (insectocide), N-terminal domain
827. Protista__Heterostelium_album_PN500_XP_020435513.1
FAMILY NOT NAMED
delta-Endotoxin (insectocide), N-terminal domain
828. Protista__Heterostelium_album_PN500_XP_020427951.1
FAMILY NOT NAMED
delta-Endotoxin (insectocide), N-terminal domain
Coil
829. Protista__Heterostelium_album_PN500_XP_020427432.1
FAMILY NOT NAMED
delta-Endotoxin (insectocide), N-terminal domain
unkn...
830. Protista__Heterostelium_album_PN500_XP_020429070.1
delta-Endotoxin (insectocide), N-terminal domain
Endotoxin_N
FAMILY NOT NAMED
831. Protista__Heterostelium_album_PN500_XP_020429019.1
FAMILY NOT NAMED
delta-Endotoxin (insectocide), N-terminal domain
Endotoxin_N
832. Protista__Heterostelium_album_PN500_XP_020429018.1
FAMILY NOT NAMED
Endotoxin_N
delta-Endotoxin (insectocide), N-terminal domain
833. Protista__Heterostelium_album_PN500_XP_020429017.1
FAMILY NOT NAMED
Cytoplasmic domain
delta-Endotoxin (insectocide), N-terminal domain
Endotoxin_N
834. Protista__Heterostelium_album_PN500_XP_020429293.1
Cytoplasmi...
Tran...
Non cytoplasmic domain
FAMILY NOT NAMED
delta-Endotoxin (insectocide), N-terminal domain
Endotoxin_N
unkn...
835. Protista__Heterostelium_album_PN500_XP_020429289.1
FAMILY NOT NAMED
delta-Endotoxin (insectocide), N-terminal domain
Coil
836. Protista__Heterostelium_album_PN500_XP_020429288.1
FAMILY NOT NAMED
delta-Endotoxin (insectocide), N-terminal domain
Coil
837. Protista__Heterostelium_album_PN500_XP_020430131.1
Cytoplasmic domain
Trans...
Non cytoplasmic domain
unkn...
FAMILY NOT NAMED
delta-Endotoxin (insectocide), N-terminal domain
Endotoxin_N
838. Protista__Heterostelium_album_PN500_XP_020431150.1
FAMILY NOT NAMED
delta-Endotoxin (insectocide), N-terminal domain
Endotoxin_N
839. Protista__Heterostelium_album_PN500_XP_020431137.1
Non cytoplasmic domain
FAMILY NOT NAMED
Endotoxin_N
delta-Endotoxin (insectocide), N-terminal domain
840. Protista__Heterostelium_album_PN500_XP_020431132.1
Non cytoplasmic domain
FAMILY NOT NAMED
Endotoxin_N
delta-Endotoxin (insectocide), N-terminal domain
841. Protista__Heterostelium_album_PN500_XP_020431131.1
Non cytoplasmic domain
Endotoxin_N
FAMILY NOT NAMED
delta-Endotoxin (insectocide), N-terminal domain

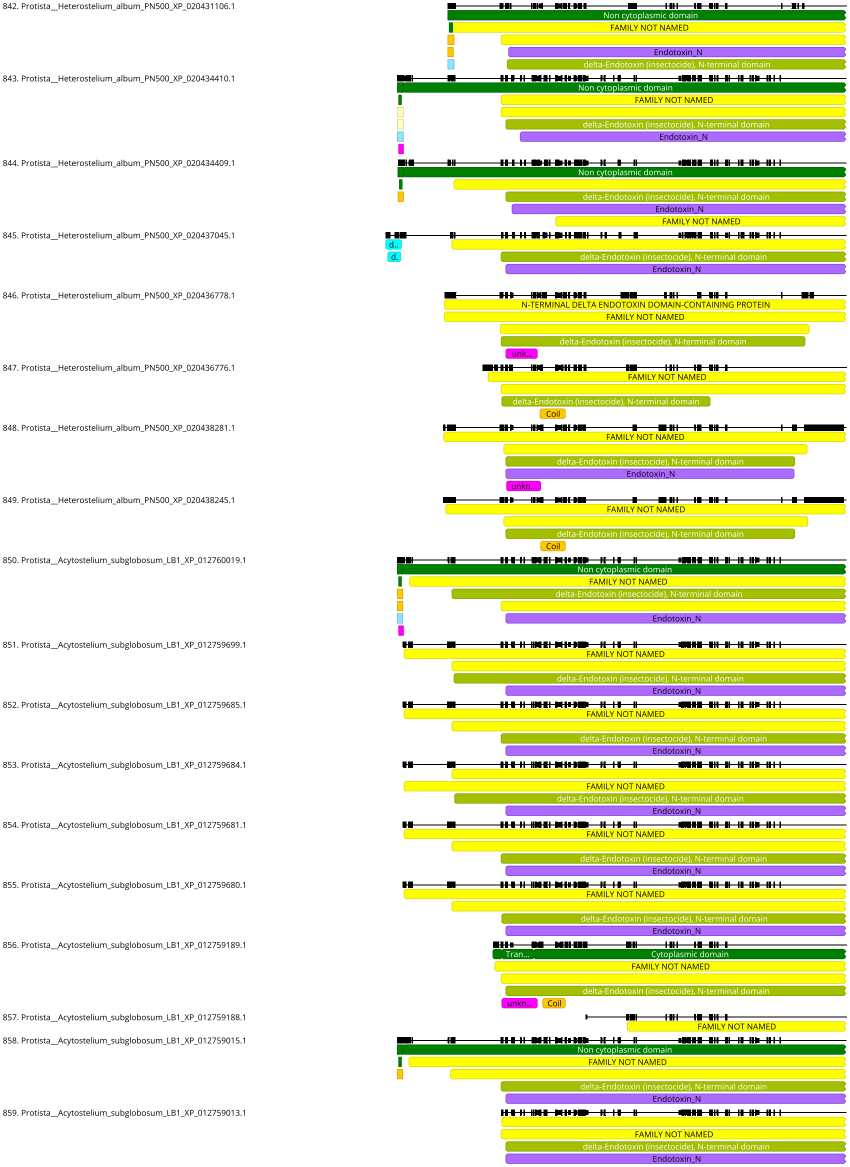
842. Protista__Heterostelium_album_PN500_XP_020431106.1
Non cytoplasmic domain
FAMILY NOT NAMED
Endotoxin_N
delta-Endotoxin (insectocide), N-terminal domain
843. Protista__Heterostelium_album_PN500_XP_020434410.1
Non cytoplasmic domain
FAMILY NOT NAMED
delta-Endotoxin (insectocide), N-terminal domain
Endotoxin_N
844. Protista__Heterostelium_album_PN500_XP_020434409.1
Non cytoplasmic domain
delta-Endotoxin (insectocide), N-terminal domain
Endotoxin_N
FAMILY NOT NAMED
845. Protista__Heterostelium_album_PN500_XP_020437045.1
d..
d.
delta-Endotoxin (insectocide), N-terminal domain
Endotoxin_N
846. Protista__Heterostelium_album_PN500_XP_020436778.1
N-TERMINAL DELTA ENDOTOXIN DOMAIN-CONTAINING PROTEIN
FAMILY NOT NAMED
delta-Endotoxin (insectocide), N-terminal domain
unk...
847. Protista__Heterostelium_album_PN500_XP_020436776.1
FAMILY NOT NAMED
delta-Endotoxin (insectocide), N-terminal domain
Coil
848. Protista__Heterostelium_album_PN500_XP_020438281.1
FAMILY NOT NAMED
delta-Endotoxin (insectocide), N-terminal domain
Endotoxin_N
unkn...
849. Protista__Heterostelium_album_PN500_XP_020438245.1
FAMILY NOT NAMED
delta-Endotoxin (insectocide), N-terminal domain
Coil
850. Protista__Acytostelium_subglobosum_LB1_XP_012760019.1
Non cytoplasmic domain
FAMILY NOT NAMED
delta-Endotoxin (insectocide), N-terminal domain
Endotoxin_N
851. Protista__Acytostelium_subglobosum_LB1_XP_012759699.1
FAMILY NOT NAMED
delta-Endotoxin (insectocide), N-terminal domain
Endotoxin_N
852. Protista__Acytostelium_subglobosum_LB1_XP_012759685.1
FAMILY NOT NAMED
delta-Endotoxin (insectocide), N-terminal domain
Endotoxin_N
853. Protista__Acytostelium_subglobosum_LB1_XP_012759684.1
FAMILY NOT NAMED
delta-Endotoxin (insectocide), N-terminal domain
Endotoxin_N
854. Protista__Acytostelium_subglobosum_LB1_XP_012759681.1
FAMILY NOT NAMED
delta-Endotoxin (insectocide), N-terminal domain
Endotoxin_N
855. Protista__Acytostelium_subglobosum_LB1_XP_012759680.1
FAMILY NOT NAMED
delta-Endotoxin (insectocide), N-terminal domain
Endotoxin_N
856. Protista__Acytostelium_subglobosum_LB1_XP_012759189.1
Tran...
Cytoplasmic domain
FAMILY NOT NAMED
delta-Endotoxin (insectocide), N-terminal domain
unkn...
Coil
857. Protista__Acytostelium_subglobosum_LB1_XP_012759188.1
FAMILY NOT NAMED
858. Protista__Acytostelium_subglobosum_LB1_XP_012759015.1
Non cytoplasmic domain
FAMILY NOT NAMED
delta-Endotoxin (insectocide), N-terminal domain
Endotoxin_N
859. Protista__Acytostelium_subglobosum_LB1_XP_012759013.1
FAMILY NOT NAMED
delta-Endotoxin (insectocide), N-terminal domain
Endotoxin_N

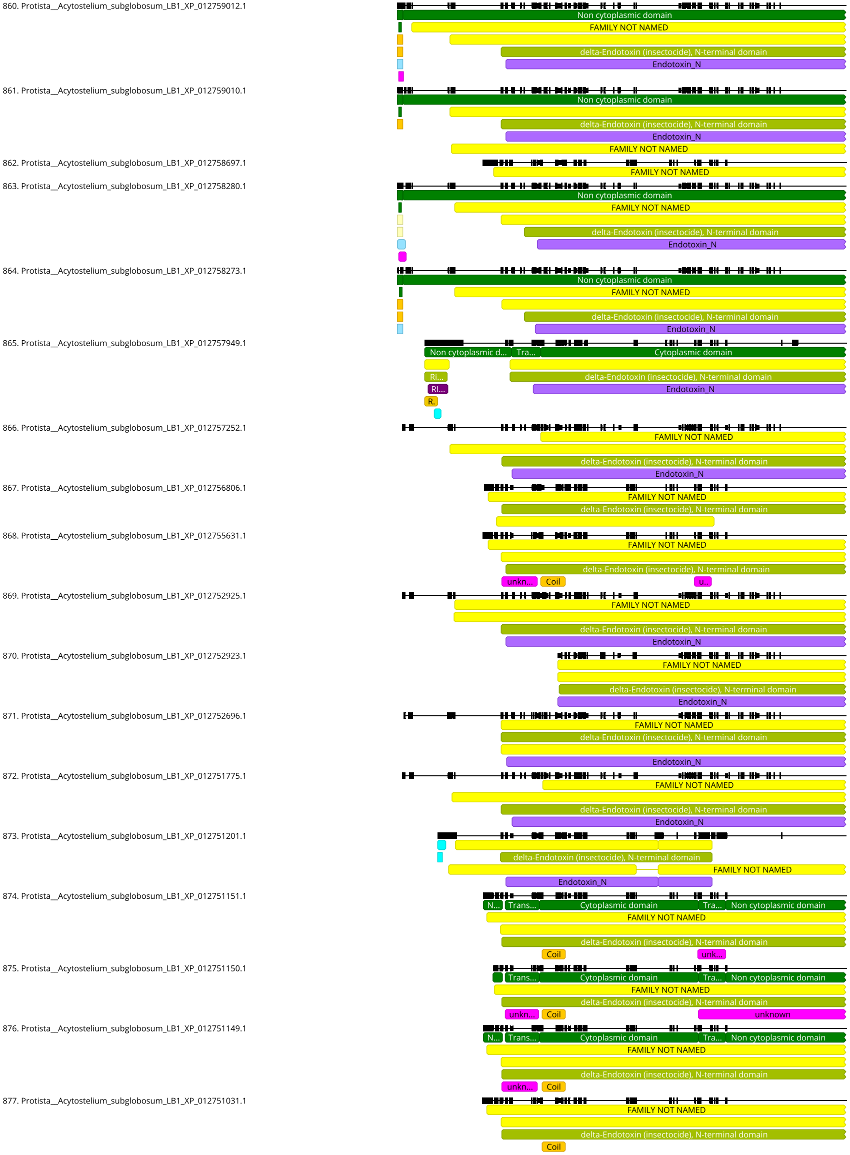
860. Protista__Acytostelium_subglobosum_LB1_XP_012759012.1
Non cytoplasmic domain
FAMILY NOT NAMED
delta-Endotoxin (insectocide), N-terminal domain
Endotoxin_N
861. Protista__Acytostelium_subglobosum_LB1_XP_012759010.1
Non cytoplasmic domain
delta-Endotoxin (insectocide), N-terminal domain
Endotoxin_N
FAMILY NOT NAMED
862. Protista__Acytostelium_subglobosum_LB1_XP_012758697.1
FAMILY NOT NAMED
863. Protista__Acytostelium_subglobosum_LB1_XP_012758280.1
Non cytoplasmic domain
FAMILY NOT NAMED
delta-Endotoxin (insectocide), N-terminal domain
Endotoxin_N
864. Protista__Acytostelium_subglobosum_LB1_XP_012758273.1
Non cytoplasmic domain
FAMILY NOT NAMED
delta-Endotoxin (insectocide), N-terminal domain
Endotoxin_N
865. Protista__Acytostelium_subglobosum_LB1_XP_012757949.1
Non cytoplasmic d...
Tra...
Cytoplasmic domain
Ri...
delta-Endotoxin (insectocide), N-terminal domain
Rl...
Endotoxin_N
R.
866. Protista__Acytostelium_subglobosum_LB1_XP_012757252.1
FAMILY NOT NAMED
delta-Endotoxin (insectocide), N-terminal domain
Endotoxin_N
867. Protista__Acytostelium_subglobosum_LB1_XP_012756806.1
FAMILY NOT NAMED
delta-Endotoxin (insectocide), N-terminal domain
868. Protista__Acytostelium_subglobosum_LB1_XP_012755631.1
FAMILY NOT NAMED
delta-Endotoxin (insectocide), N-terminal domain
unkn...
Coil
u..
869. Protista__Acytostelium_subglobosum_LB1_XP_012752925.1
FAMILY NOT NAMED
delta-Endotoxin (insectocide), N-terminal domain
Endotoxin_N
870. Protista__Acytostelium_subglobosum_LB1_XP_012752923.1
FAMILY NOT NAMED
delta-Endotoxin (insectocide), N-terminal domain
Endotoxin_N
871. Protista__Acytostelium_subglobosum_LB1_XP_012752696.1
FAMILY NOT NAMED
delta-Endotoxin (insectocide), N-terminal domain
Endotoxin_N
872. Protista__Acytostelium_subglobosum_LB1_XP_012751775.1
FAMILY NOT NAMED
delta-Endotoxin (insectocide), N-terminal domain
Endotoxin_N
873. Protista__Acytostelium_subglobosum_LB1_XP_012751201.1
delta-Endotoxin (insectocide), N-terminal domain
FAMILY NOT NAMED
Endotoxin_N
874. Protista__Acytostelium_subglobosum_LB1_XP_012751151.1
N...
Trans...
Cytoplasmic domain
Tra...
Non cytoplasmic domain
FAMILY NOT NAMED
delta-Endotoxin (insectocide), N-terminal domain
Coil
unk...
875. Protista__Acytostelium_subglobosum_LB1_XP_012751150.1
Trans...
Cytoplasmic domain
Tra...
Non cytoplasmic domain
FAMILY NOT NAMED
delta-Endotoxin (insectocide), N-terminal domain
unkn...
Coil
unknown
876. Protista__Acytostelium_subglobosum_LB1_XP_012751149.1
N...
Trans...
Cytoplasmic domain
Tra...
Non cytoplasmic domain
FAMILY NOT NAMED
delta-Endotoxin (insectocide), N-terminal domain
unkn...
Coil
877. Protista__Acytostelium_subglobosum_LB1_XP_012751031.1
FAMILY NOT NAMED
delta-Endotoxin (insectocide), N-terminal domain
Coil

878. Protista__Acytostelium_subglobosum_LB1_XP_012751030.1
879. Protista__Acytostelium_subglobosum_LB1_XP_012750528.1
880. Protista__Acytostelium_subglobosum_LB1_XP_012749234.1
881. Protista__Acytostelium_subglobosum_LB1_XP_012749103.1
882. Protista__Acytostelium_subglobosum_LB1_XP_012749016.1
883. Protista__Acytostelium_subglobosum_LB1_XP_012748975.1
884. Protista__Acytostelium_subglobosum_LB1_XP_012748660.1
885. Protista__Acytostelium_subglobosum_LB1_XP_012748658.1
886. Protista__Chondrus_crispus_XP_005716346.1
887. Protista__Cavenderia_fasciculata_XP_004362835.1
888. Protista__Cavenderia_fasciculata_XP_004362538.1
889. Protista__Cavenderia_fasciculata_XP_004361802.1
890. Protista__Cavenderia_fasciculata_XP_004360924.1
891. Protista__Cavenderia_fasciculata_XP_004358759.1
892. Protista__Cavenderia_fasciculata_XP_004358155.1
893. Protista__Cavenderia_fasciculata_XP_004357975.1
894. Protista__Cavenderia_fasciculata_XP_004366826.1
895. Protista__Cavenderia_fasciculata_XP_004356210.1
896. Protista__Cavenderia_fasciculata_XP_004356193.1
897. Protista__Cavenderia_fasciculata_XP_004355686.1
898. Protista__Cavenderia_fasciculata_XP_004354858.1
899. Protista__Cavenderia_fasciculata_XP_004352345.1

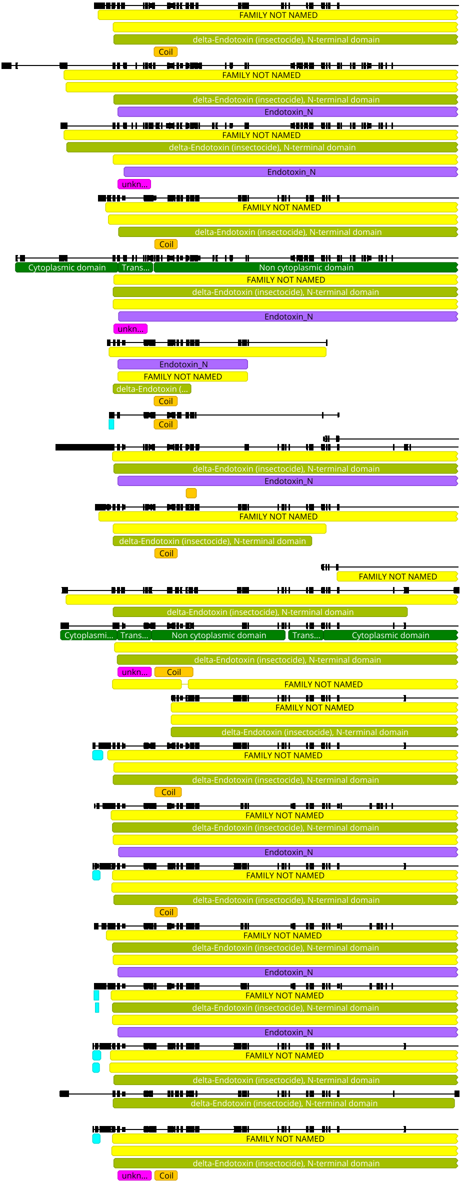

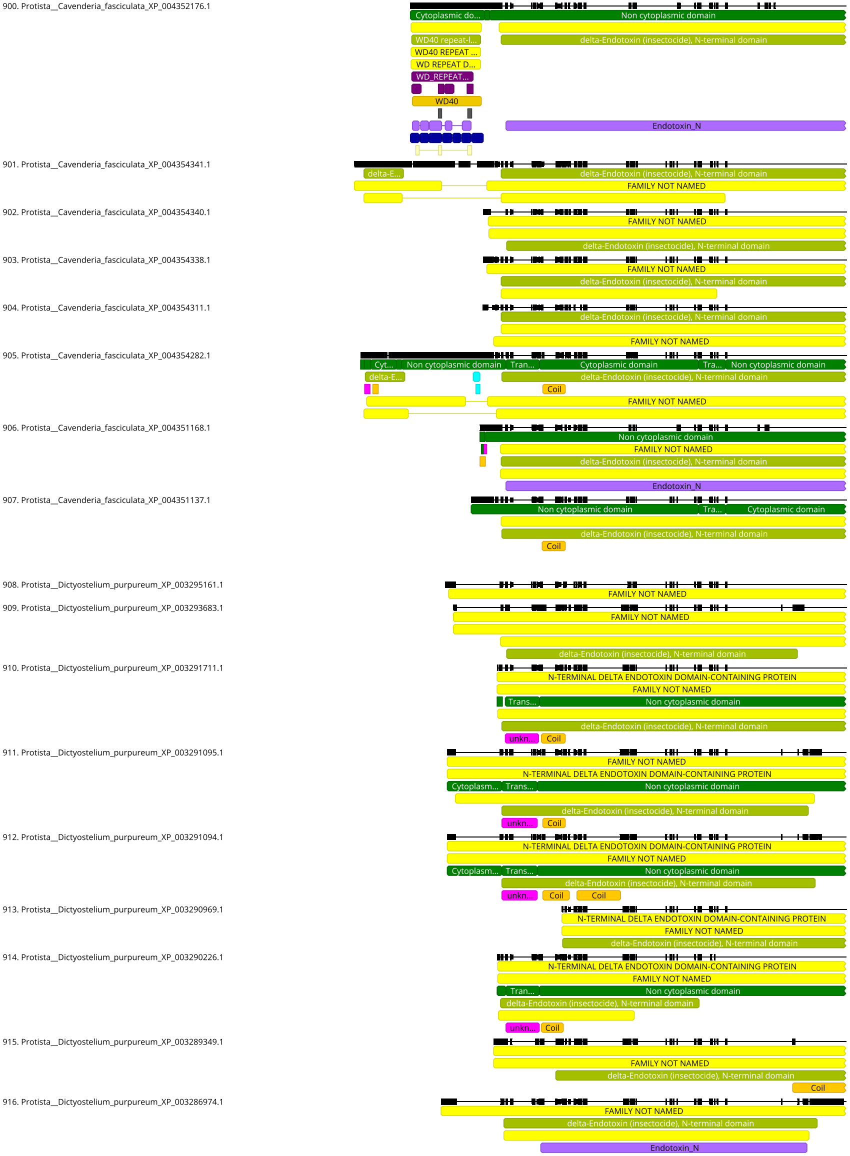

900. Protista__Cavendera_fasciculata_XP_004352176.1
Cytoplasmic do...
Non cytoplasmic domain
WD40 repeat-l...
delta-Endotoxin (insectocide), N-terminal domain
WD40 REPEAT ...
WD REPEAT D...
WD_REPEAT...
WD40
Endotoxin_N
901. Protista__Cavendera_fasciculata_XP_004354341.1
delta-E...
delta-Endotoxin (insectocide), N-terminal domain
FAMILY NOT NAMED
902. Protista__Cavendera_fasciculata_XP_004354340.1
FAMILY NOT NAMED
delta-Endotoxin (insectocide), N-terminal domain
903. Protista__Cavendera_fasciculata_XP_004354338.1
FAMILY NOT NAMED
delta-Endotoxin (insectocide), N-terminal domain
904. Protista__Cavendera_fasciculata_XP_004354311.1
delta-Endotoxin (insectocide), N-terminal domain
FAMILY NOT NAMED
905. Protista__Cavendera_fasciculata_XP_004354282.1
Cyt...
Non cytoplasmic domain
Tran...
Cytoplasmic domain
Tra...
Non cytoplasmic domain
delta-E...
delta-Endotoxin (insectocide), N-terminal domain
Coil
FAMILY NOT NAMED
906. Protista__Cavendera_fasciculata_XP_004351168.1
Non cytoplasmic domain
FAMILY NOT NAMED
delta-Endotoxin (insectocide), N-terminal domain
Endotoxin_N
907. Protista__Cavendera_fasciculata_XP_004351137.1
Non cytoplasmic domain
Tra...
Cytoplasmic domain
delta-Endotoxin (insectocide), N-terminal domain
Coil
908. Protista__Dictyostelium_purpureum_XP_003295161.1
FAMILY NOT NAMED
909. Protista__Dictyostelium_purpureum_XP_003293683.1
FAMILY NOT NAMED
delta-Endotoxin (insectocide), N-terminal domain
910. Protista__Dictyostelium_purpureum_XP_003291711.1
N-TERMINAL DELTA ENDOTOXIN DOMAIN-CONTAINING PROTEIN
FAMILY NOT NAMED
Trans...
Non cytoplasmic domain
delta-Endotoxin (insectocide), N-terminal domain
unkn...
Coil
911. Protista__Dictyostelium_purpureum_XP_003291095.1
FAMILY NOT NAMED
N-TERMINAL DELTA ENDOTOXIN DOMAIN-CONTAINING PROTEIN
Cytoplasm...
Trans...
Non cytoplasmic domain
delta-Endotoxin (insectocide), N-terminal domain
unkn...
Coil
912. Protista__Dictyostelium_purpureum_XP_003291094.1
N-TERMINAL DELTA ENDOTOXIN DOMAIN-CONTAINING PROTEIN
FAMILY NOT NAMED
Cytoplasm...
Trans...
Non cytoplasmic domain
delta-Endotoxin (insectocide), N-terminal domain
unkn...
Coil
Coil
913. Protista__Dictyostelium_purpureum_XP_003290969.1
N-TERMINAL DELTA ENDOTOXIN DOMAIN-CONTAINING PROTEIN
FAMILY NOT NAMED
delta-Endotoxin (insectocide), N-terminal domain
914. Protista__Dictyostelium_purpureum_XP_003290226.1
N-TERMINAL DELTA ENDOTOXIN DOMAIN-CONTAINING PROTEIN
FAMILY NOT NAMED
Tran...
Non cytoplasmic domain
delta-Endotoxin (insectocide), N-terminal domain
unkn...
Coil
915. Protista__Dictyostelium_purpureum_XP_003289349.1
FAMILY NOT NAMED
delta-Endotoxin (insectocide), N-terminal domain
Coil
916. Protista__Dictyostelium_purpureum_XP_003286974.1
FAMILY NOT NAMED
delta-Endotoxin (insectocide), N-terminal domain
Endotoxin_N

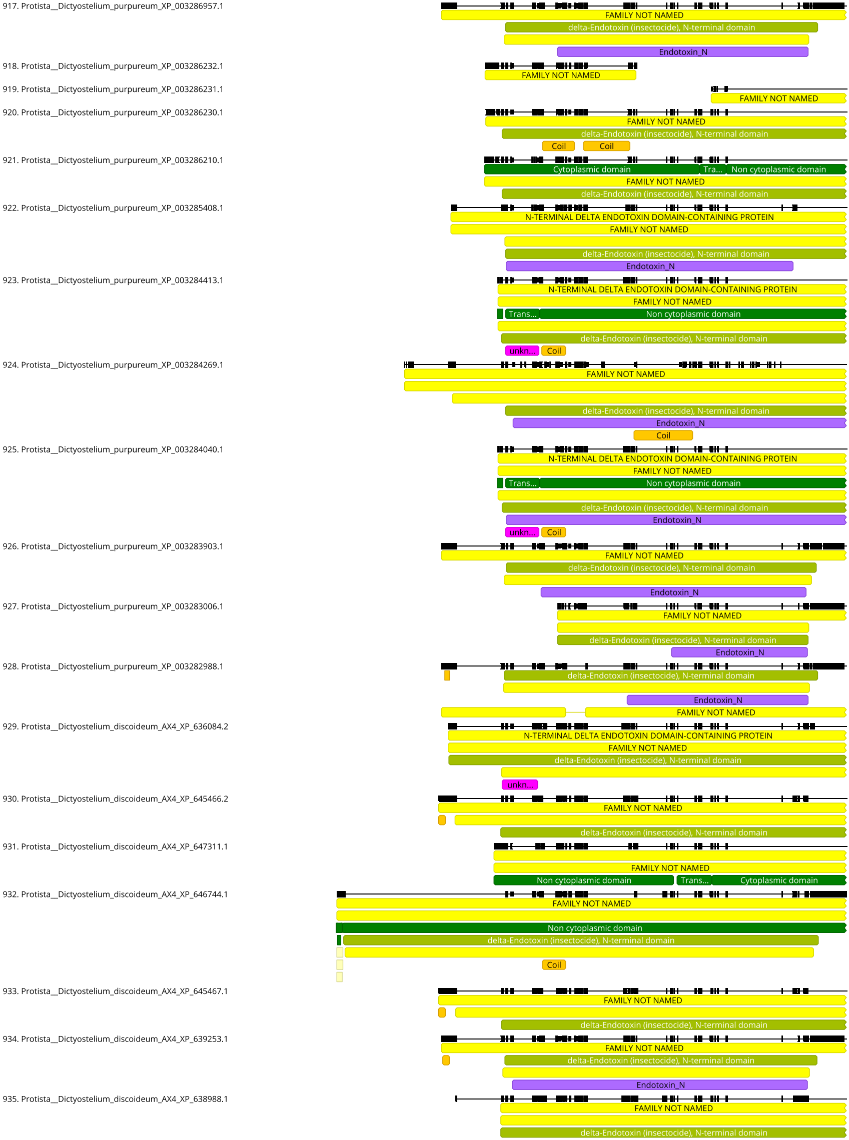
917. Protista__Dictyostelium_purpureum_XP_003286957.1
FAMILY NOT NAMED
delta-Endotoxin (insectocide), N-terminal domain
Endotoxin_N
918. Protista__Dictyostelium_purpureum_XP_003286232.1
FAMILY NOT NAMED
919. Protista__Dictyostelium_purpureum_XP_003286231.1
FAMILY NOT NAMED
920. Protista__Dictyostelium_purpureum_XP_003286230.1
FAMILY NOT NAMED
delta-Endotoxin (insectocide), N-terminal domain
Coil
Coil
921. Protista__Dictyostelium_purpureum_XP_003286210.1
Cytoplasmic domain
Tra...
Non cytoplasmic domain
FAMILY NOT NAMED
delta-Endotoxin (insectocide), N-terminal domain
922. Protista__Dictyostelium_purpureum_XP_003285408.1
N-TERMINAL DELTA ENDOTOXIN DOMAIN-CONTAINING PROTEIN
FAMILY NOT NAMED
delta-Endotoxin (insectocide), N-terminal domain
Endotoxin_N
923. Protista__Dictyostelium_purpureum_XP_003284413.1
N-TERMINAL DELTA ENDOTOXIN DOMAIN-CONTAINING PROTEIN
FAMILY NOT NAMED
Trans...
Non cytoplasmic domain
delta-Endotoxin (insectocide), N-terminal domain
unkn...
Coil
924. Protista__Dictyostelium_purpureum_XP_003284269.1
FAMILY NOT NAMED
delta-Endotoxin (insectocide), N-terminal domain
Endotoxin_N
Coil
925. Protista__Dictyostelium_purpureum_XP_003284040.1
N-TERMINAL DELTA ENDOTOXIN DOMAIN-CONTAINING PROTEIN
FAMILY NOT NAMED
Trans...
Non cytoplasmic domain
delta-Endotoxin (insectocide), N-terminal domain
Endotoxin_N
unkn...
Coil
926. Protista__Dictyostelium_purpureum_XP_003283903.1
FAMILY NOT NAMED
delta-Endotoxin (insectocide), N-terminal domain
Endotoxin_N
927. Protista__Dictyostelium_purpureum_XP_003283006.1
FAMILY NOT NAMED
delta-Endotoxin (insectocide), N-terminal domain
Endotoxin_N
928. Protista__Dictyostelium_purpureum_XP_003282988.1
delta-Endotoxin (insectocide), N-terminal domain
Endotoxin_N
FAMILY NOT NAMED
929. Protista__Dictyostelium_discoideum_AX4_XP_636084.2
N-TERMINAL DELTA ENDOTOXIN DOMAIN-CONTAINING PROTEIN
FAMILY NOT NAMED
delta-Endotoxin (insectocide), N-terminal domain
unkn...
930. Protista__Dictyostelium_discoideum_AX4_XP_645466.2
FAMILY NOT NAMED
delta-Endotoxin (insectocide), N-terminal domain
931. Protista__Dictyostelium_discoideum_AX4_XP_647311.1
FAMILY NOT NAMED
Non cytoplasmic domain
Trans...
Cytoplasmic domain
932. Protista__Dictyostelium_discoideum_AX4_XP_646744.1
FAMILY NOT NAMED
Non cytoplasmic domain
delta-Endotoxin (insectocide), N-terminal domain
Coil
933. Protista__Dictyostelium_discoideum_AX4_XP_645467.1
FAMILY NOT NAMED
delta-Endotoxin (insectocide), N-terminal domain
934. Protista__Dictyostelium_discoideum_AX4_XP_639253.1
FAMILY NOT NAMED
delta-Endotoxin (insectocide), N-terminal domain
Endotoxin_N
935. Protista__Dictyostelium_discoideum_AX4_XP_638988.1
FAMILY NOT NAMED
delta-Endotoxin (insectocide), N-terminal domain

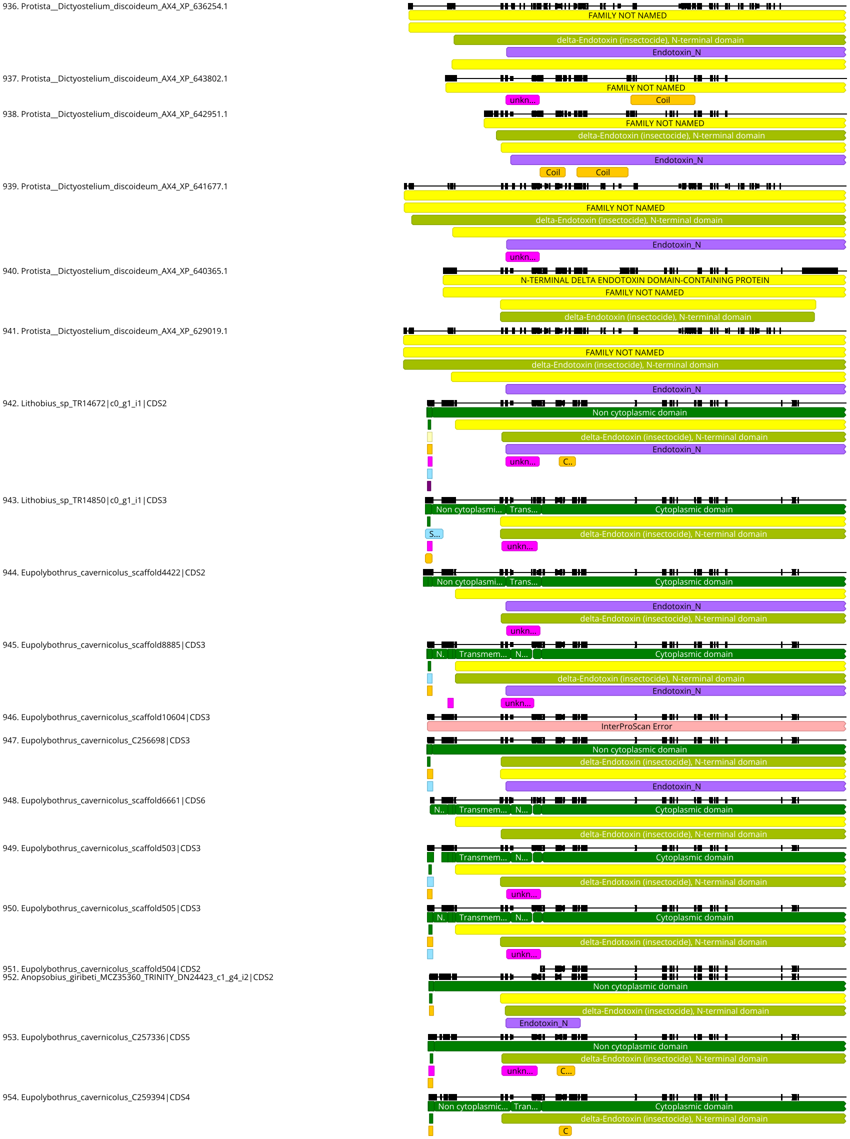
936. Protista__Dictyostelium_discoideum_AX4_XP_636254.1
FAMILY NOT NAMED
delta-Endotoxin (insectocide), N-terminal domain
Endotoxin_N
937. Protista__Dictyostelium_discoideum_AX4_XP_643802.1
FAMILY NOT NAMED
unkn...
Coil
938. Protista__Dictyostelium_discoideum_AX4_XP_642951.1
FAMILY NOT NAMED
delta-Endotoxin (insectocide), N-terminal domain
Endotoxin_N
Coil
Coil
939. Protista__Dictyostelium_discoideum_AX4_XP_641677.1
FAMILY NOT NAMED
delta-Endotoxin (insectocide), N-terminal domain
Endotoxin_N
unkn...
940. Protista__Dictyostelium_discoideum_AX4_XP_640365.1
N-TERMINAL DELTA ENDOTOXIN DOMAIN-CONTAINING PROTEIN
FAMILY NOT NAMED
delta-Endotoxin (insectocide), N-terminal domain
941. Protista__Dictyostelium_discoideum_AX4_XP_629019.1
FAMILY NOT NAMED
delta-Endotoxin (insectocide), N-terminal domain
Endotoxin_N
942. Lithobius_sp_TR14672|c0_g1_i1|CDS2
Non cytoplasmic domain
delta-Endotoxin (insectocide), N-terminal domain
Endotoxin_N
unkn...
C..
943. Lithobius_sp_TR14850|c0_g1_i1|CDS3
Non cytoplasmi...
Trans...
Cytoplasmic domain
S...
delta-Endotoxin (insectocide), N-terminal domain
unkn...
944. Eupolybothrus_cavernicolus_scaffold4422|CDS2
Non cytoplasmi...
Trans...
Cytoplasmic domain
Endotoxin_N
delta-Endotoxin (insectocide), N-terminal domain
unkn...
945. Eupolybothrus_cavernicolus_scaffold8885|CDS3
N.
Transmem...
N...
Cytoplasmic domain
delta-Endotoxin (insectocide), N-terminal domain
Endotoxin_N
unkn...
946. Eupolybothrus_cavernicolus_scaffold10604|CDS3
InterProScan Error
947. Eupolybothrus_cavernicolus_C256698|CDS3
Non cytoplasmic domain
delta-Endotoxin (insectocide), N-terminal domain
Endotoxin_N
948. Eupolybothrus_cavernicolus_scaffold6661|CDS6
N..
Transmem...
N...
Cytoplasmic domain
delta-Endotoxin (insectocide), N-terminal domain
949. Eupolybothrus_cavernicolus_scaffold503|CDS3
Transmem...
N...
Cytoplasmic domain
delta-Endotoxin (insectocide), N-terminal domain
unkn...
950. Eupolybothrus_cavernicolus_scaffold505|CDS3
N.
Transmem...
N...
Cytoplasmic domain
delta-Endotoxin (insectocide), N-terminal domain
unkn...
951. Eupolybothrus_cavernicolus_scaffold504|CDS2
952. Anopsobius_giribeti_MCZ35360_TRINITY_DN24423_c1_g4_i2|CDS2
Non cytoplasmic domain
delta-Endotoxin (insectocide), N-terminal domain
Endotoxin_N
953. Eupolybothrus_cavernicolus_C257336|CDS5
Non cytoplasmic domain
delta-Endotoxin (insectocide), N-terminal domain
unkn...
C...
954. Eupolybothrus_cavernicolus_C259394|CDS4
Non cytoplasmic...
Tran...
Cytoplasmic domain
delta-Endotoxin (insectocide), N-terminal domain
C

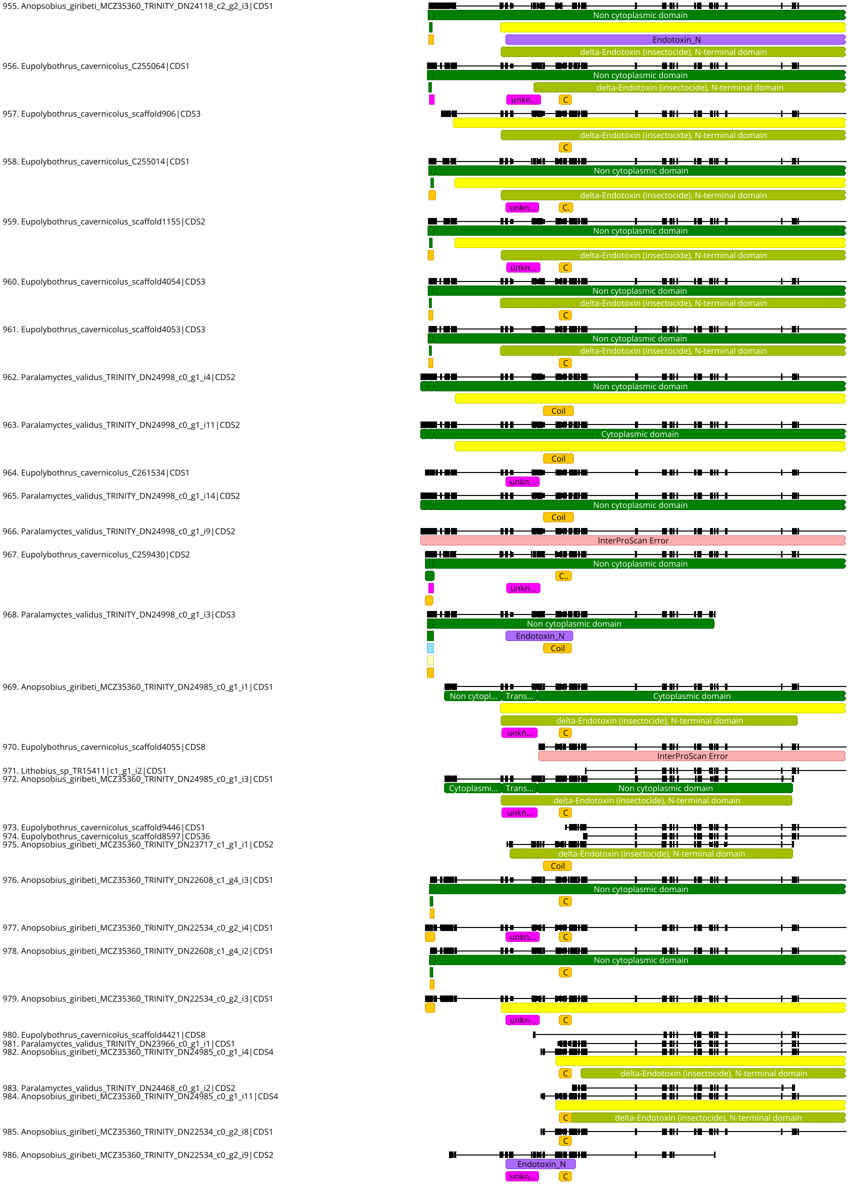
955. Anopsobius_giribeti_MCZ35360_TRINITY_DN24118_c2_g2_i3|CDS1
Non cytoplasmic domain
Endotoxin_N
delta-Endotoxin (insectocide), N-terminal domain
956. Eupolybothrus_cavernicolus_C255064|CDS1
Non cytoplasmic domain
delta-Endotoxin (insectocide), N-terminal domain
unkn...
C
957. Eupolybothrus_cavernicolus_scaffold906|CDS3
delta-Endotoxin (insectocide), N-terminal domain
C
958. Eupolybothrus_cavernicolus_C255014|CDS1
Non cytoplasmic domain
delta-Endotoxin (insectocide), N-terminal domain
unkn...
C.
959. Eupolybothrus_cavernicolus_scaffold1155|CDS2
Non cytoplasmic domain
delta-Endotoxin (insectocide), N-terminal domain
unkn...
C
960. Eupolybothrus_cavernicolus_scaffold4054|CDS3
Non cytoplasmic domain
delta-Endotoxin (insectocide), N-terminal domain
C
961. Eupolybothrus_cavernicolus_scaffold4053|CDS3
Non cytoplasmic domain
delta-Endotoxin (insectocide), N-terminal domain
C
962. Paralamyctes_validus_TRINITY_DN24998_c0_g1_i4|CDS2
Non cytoplasmic domain
Coil
963. Paralamyctes_validus_TRINITY_DN24998_c0_g1_i11|CDS2
Cytoplasmic domain
Coil
964. Eupolybothrus_cavernicolus_C261534|CDS1
unkn...
965. Paralamyctes_validus_TRINITY_DN24998_c0_g1_i14|CDS2
Non cytoplasmic domain
Coil
966. Paralamyctes_validus_TRINITY_DN24998_c0_g1_i9|CDS2
InterProScan Error
967. Eupolybothrus_cavernicolus_C259430|CDS2
Non cytoplasmic domain
C..
unkn...
968. Paralamyctes_validus_TRINITY_DN24998_c0_g1_i3|CDS3
Non cytoplasmic domain
Endotoxin_N
Coil
969. Anopsobius_giribeti_MCZ35360_TRINITY_DN24985_c0_g1_i1|CDS1
Non cytopl...
Trans...
Cytoplasmic domain
delta-Endotoxin (insectocide), N-terminal domain
unkn...
C
970. Eupolybothrus_cavernicolus_scaffold4055|CDS8
InterProScan Error
971. Lithobius_sp_TR15411|c1_g1_i2|CDS1
972. Anopsobius_giribeti_MCZ35360_TRINITY_DN24985_c0_g1_i3|CDS1
Cytoplasmi...
Trans...
Non cytoplasmic domain
delta-Endotoxin (insectocide), N-terminal domain
unkn...
C
973. Eupolybothrus_cavernicolus_scaffold9446|CDS1
974. Eupolybothrus_cavernicolus_scaffold8597|CDS36
975. Anopsobius_giribeti_MCZ35360_TRINITY_DN23717_c1_g1_i1|CDS2
delta-Endotoxin (insectocide), N-terminal domain
Coil
976. Anopsobius_giribeti_MCZ35360_TRINITY_DN22608_c1_g4_i3|CDS1
Non cytoplasmic domain
C
977. Anopsobius_giribeti_MCZ35360_TRINITY_DN22534_c0_g2_i4|CDS1
unkn...
C
978. Anopsobius_giribeti_MCZ35360_TRINITY_DN22608_c1_g4_i2|CDS1
Non cytoplasmic domain
C
979. Anopsobius_giribeti_MCZ35360_TRINITY_DN22534_c0_g2_i3|CDS1
unkn...
C
980. Eupolybothrus_cavernicolus_scaffold4421|CDS8
981. Paralamyctes_validus_TRINITY_DN23966_c0_g1_i1|CDS1
982. Anopsobius_giribeti_MCZ35360_TRINITY_DN24985_c0_g1_i4|CDS4
C
delta-Endotoxin (insectocide), N-terminal domain
983. Paralamyctes_validus_TRINITY_DN24468_c0_g1_i2|CDS2
984. Anopsobius_giribeti_MCZ35360_TRINITY_DN24985_c0_g1_i11|CDS4
C
delta-Endotoxin (insectocide), N-terminal domain
985. Anopsobius_giribeti_MCZ35360_TRINITY_DN22534_c0_g2_i8|CDS1
C
986. Anopsobius_giribeti_MCZ35360_TRINITY_DN22534_c0_g2_i9|CDS2
Endotoxin_N
unkn...
C

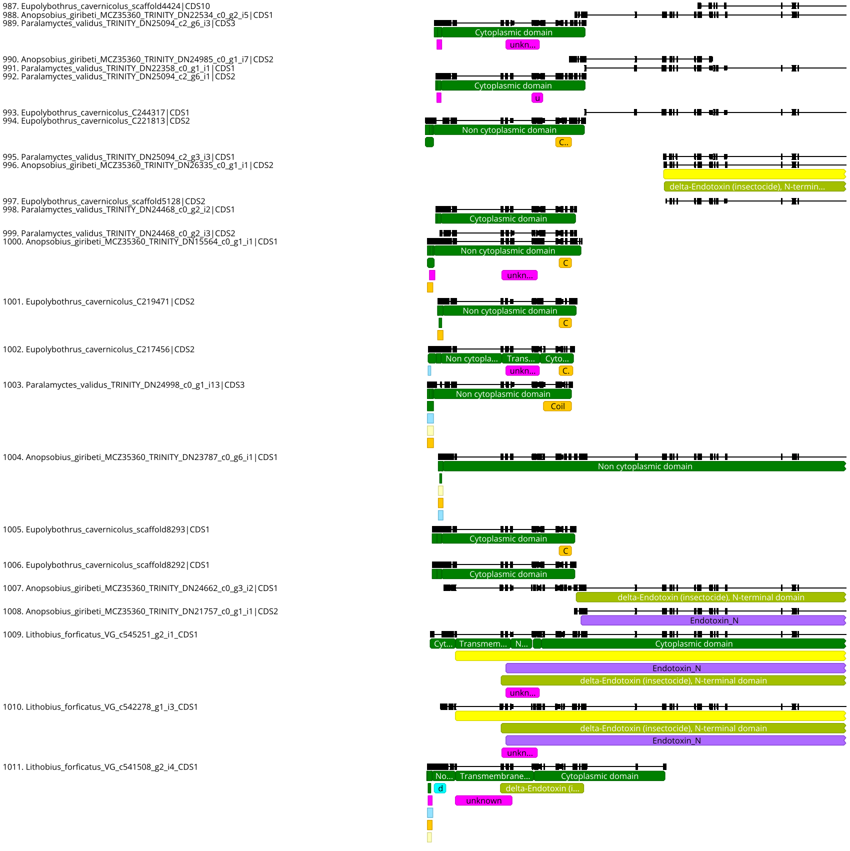
987. Eupolybothrus_cavernicolus_scaffold4424|CDS10
988. Anopsobius_giribeti_MCZ35360_TRINITY_DN22534_c0_g2_i5|CDS1
989. Paralamyctes_validus_TRINITY_DN25094_c2_g6_i3|CDS3
Cytoplasmic domain
unkn...
990. Anopsobius_giribeti_MCZ35360_TRINITY_DN24985_c0_g1_i7|CDS2
991. Paralamyctes_validus_TRINITY_DN22358_c0_g1_i1|CDS1
992. Paralamyctes_validus_TRINITY_DN25094_c2_g6_i1|CDS2
Cytoplasmic domain
u
993. Eupolybothrus_cavernicolus_C244317|CDS1
994. Eupolybothrus_cavernicolus_C221813|CDS2
Non cytoplasmic domain
C..
995. Paralamyctes_validus_TRINITY_DN25094_c2_g3_i3|CDS1
996. Anopsobius_giribeti_MCZ35360_TRINITY_DN26335_c0_g1_i1|CDS2
delta-Endotoxin (insectocide), N-termin...
997. Eupolybothrus_cavernicolus_scaffold5128|CDS2
998. Paralamyctes_validus_TRINITY_DN24468_c0_g2_i2|CDS1
Cytoplasmic domain
999. Paralamyctes_validus_TRINITY_DN24468_c0_g2_i3|CDS2
1000. Anopsobius_giribeti_MCZ35360_TRINITY_DN15564_c0_g1_i1|CDS1
Non cytoplasmic domain
C
unkn...
1001. Eupolybothrus_cavernicolus_C219471|CDS2
Non cytoplasmic domain
C
1002. Eupolybothrus_cavernicolus_C217456|CDS2
Non cytopla...
Trans...
Cyto...
unkn...
C.
1003. Paralamyctes_validus_TRINITY_DN24998_c0_g1_i13|CDS3
Non cytoplasmic domain
Coil
1004. Anopsobius_giribeti_MCZ35360_TRINITY_DN23787_c0_g6_i1|CDS1
Non cytoplasmic domain
1005. Eupolybothrus_cavernicolus_scaffold8293|CDS1
Cytoplasmic domain
C
1006. Eupolybothrus_cavernicolus_scaffold8292|CDS1
Cytoplasmic domain
1007. Anopsobius_giribeti_MCZ35360_TRINITY_DN24662_c0_g3_i2|CDS1
delta-Endotoxin (insectocide), N-terminal domain
1008. Anopsobius_giribeti_MCZ35360_TRINITY_DN21757_c0_g1_i1|CDS2
Endotoxin_N
1009. Lithobius_forficatus_VG_c545251_g2_i1_CDS1
Cyt...
Transmem...
N...
Cytoplasmic domain
Endotoxin_N
delta-Endotoxin (insectocide), N-terminal domain
unkn...
1010. Lithobius_forficatus_VG_c542278_g1_i3_CDS1
delta-Endotoxin (insectocide), N-terminal domain
Endotoxin_N
unkn...
1011. Lithobius_forficatus_VG_c541508_g2_i4_CDS1
No...
Transmembrane...
Cytoplasmic domain
d
delta-Endotoxin (i...
unknown
