# Supplementary material for: Phylogenetic analyses suggest centipede venom arsenals were repeatedly stocked by horizontal gene transfer
Source: Nat Commun. 2021 Feb 5;12:818. doi: 10.1038/s41467-021-21093-8 (PMC7864903; doi:10.1038/s41467-021-21093-8)

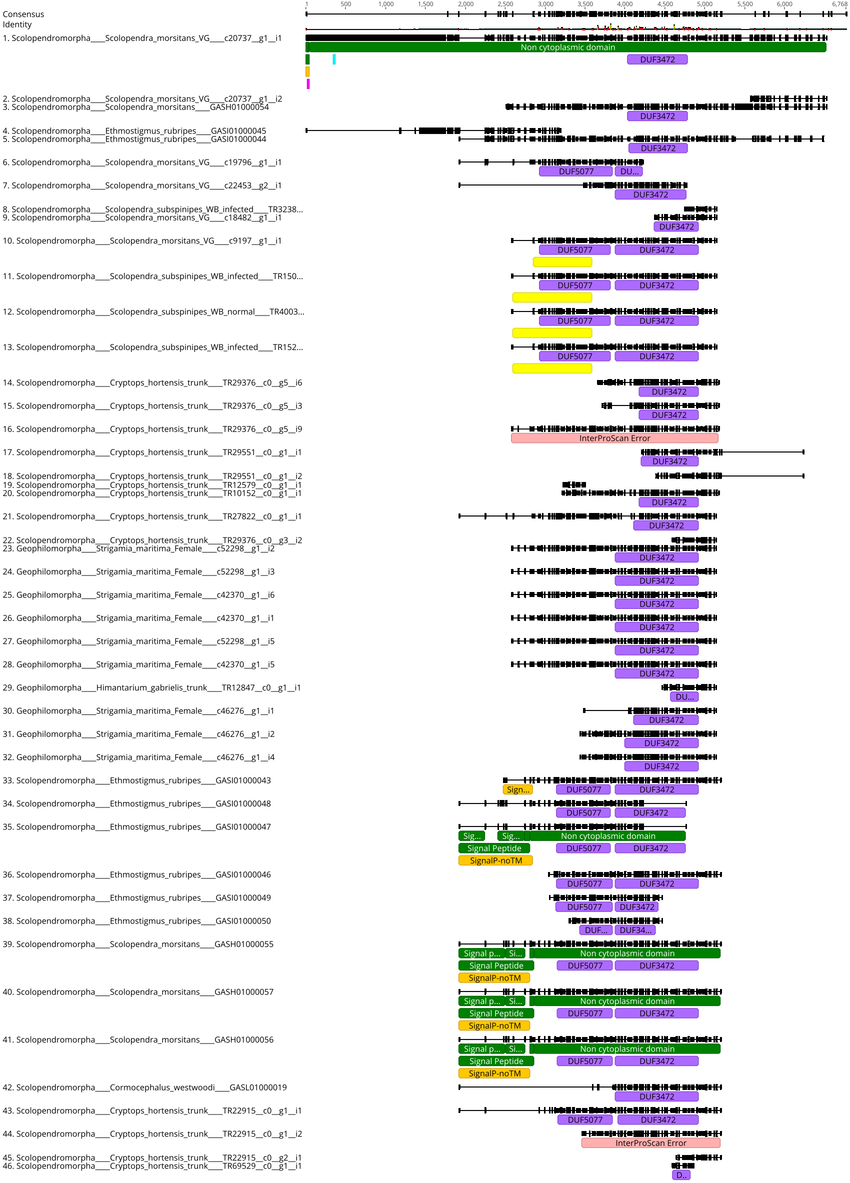
Consensus
Identity
1. Scolopendromorpha___Scolopendra_morsitans_VG___c20737__g1__i1
Non cytoplasmic domain
DUF3472
2. Scolopendromorpha___Scolopendra_morsitans_VG___c20737__g1__i2
3. Scolopendromorpha___Scolopendra_morsitans___GASH01000054
DUF3472
4. Scolopendromorpha___Ethmostigmus_rubripes___GASI01000045
5. Scolopendromorpha___Ethmostigmus_rubripes___GASI01000044
DUF3472
6. Scolopendromorpha___Scolopendra_morsitans_VG___c19796__g1__i1
DUF5077
DU...
7. Scolopendromorpha___Scolopendra_morsitans_VG___c22453__g2__i1
DUF3472
8. Scolopendromorpha___Scolopendra_subspinipes_WB_infected___TR3238...
9. Scolopendromorpha___Scolopendra_morsitans_VG___c18482__g1__i1
DUF3472
10. Scolopendromorpha___Scolopendra_morsitans_VG___c9197__g1__i1
DUF5077
DUF3472
11. Scolopendromorpha___Scolopendra_subspinipes_WB_infected___TR150...
DUF5077
DUF3472
12. Scolopendromorpha___Scolopendra_subspinipes_WB_normal___TR4003...
DUF5077
DUF3472
13. Scolopendromorpha___Scolopendra_subspinipes_WB_infected___TR152...
DUF5077
DUF3472
14. Scolopendromorpha___Cryptops_hortensis_trunk___TR29376__c0__g5__i6
DUF3472
15. Scolopendromorpha___Cryptops_hortensis_trunk___TR29376__c0__g5__i3
DUF3472
16. Scolopendromorpha___Cryptops_hortensis_trunk___TR29376__c0__g5__i9
InterProScan Error
17. Scolopendromorpha___Cryptops_hortensis_trunk___TR29551__c0__g1__i1
DUF3472
18. Scolopendromorpha___Cryptops_hortensis_trunk___TR29551__c0__g1__i2
19. Scolopendromorpha___Cryptops_hortensis_trunk___TR12579__c0__g1__i1
20. Scolopendromorpha___Cryptops_hortensis_trunk___TR10152__c0__g1__i1
DUF3472
21. Scolopendromorpha___Cryptops_hortensis_trunk___TR27822__c0__g1__i1
DUF3472
22. Scolopendromorpha___Cryptops_hortensis_trunk___TR29376__c0__g3__i2
23. Geophilomorpha___Strigamia_maritima_Female___c52298__g1__i2
DUF3472
24. Geophilomorpha___Strigamia_maritima_Female___c52298__g1__i3
DUF3472
25. Geophilomorpha___Strigamia_maritima_Female___c42370__g1__i6
DUF3472
26. Geophilomorpha___Strigamia_maritima_Female___c42370__g1__i1
DUF3472
27. Geophilomorpha___Strigamia_maritima_Female___c52298__g1__i5
DUF3472
28. Geophilomorpha___Strigamia_maritima_Female___c42370__g1__i5
DUF3472
29. Geophilomorpha___Himantarium_gabrielis_trunk___TR12847__c0__g1__i1
DU...
30. Geophilomorpha___Strigamia_maritima_Female___c46276__g1__i1
DUF3472
31. Geophilomorpha___Strigamia_maritima_Female___c46276__g1__i2
DUF3472
32. Geophilomorpha___Strigamia_maritima_Female___c46276__g1__i4
DUF3472
33. Scolopendromorpha___Ethmostigmus_rubripes___GASI01000043
Sign...
DUF5077
DUF3472
34. Scolopendromorpha___Ethmostigmus_rubripes___GASI01000048
DUF5077
DUF3472
35. Scolopendromorpha___Ethmostigmus_rubripes___GASI01000047
Sig...
Sig...
Non cytoplasmic domain
Signal Peptide
DUF5077
DUF3472
SignalP-noTM
36. Scolopendromorpha___Ethmostigmus_rubripes___GASI01000046
DUF5077
DUF3472
37. Scolopendromorpha___Ethmostigmus_rubripes___GASI01000049
DUF5077
DUF3472
38. Scolopendromorpha___Ethmostigmus_rubripes___GASI01000050
DUF...
DUF34...
39. Scolopendromorpha___Scolopendra_morsitans___GASH01000055
Signal p...
Si...
Non cytoplasmic domain
Signal Peptide
DUF5077
DUF3472
SignalP-noTM
40. Scolopendromorpha___Scolopendra_morsitans___GASH01000057
Signal p...
Si...
Non cytoplasmic domain
Signal Peptide
DUF5077
DUF3472
SignalP-noTM
41. Scolopendromorpha___Scolopendra_morsitans___GASH01000056
Signal p...
Si...
Non cytoplasmic domain
Signal Peptide
DUF5077
DUF3472
SignalP-noTM
42. Scolopendromorpha___Cormocephalus_westwoodi___GASL01000019
DUF3472
43. Scolopendromorpha___Cryptops_hortensis_trunk___TR22915__c0__g1__i1
DUF5077
DUF3472
44. Scolopendromorpha___Cryptops_hortensis_trunk___TR22915__c0__g1__i2
InterProScan Error
45. Scolopendromorpha___Cryptops_hortensis_trunk___TR22915__c0__g2__i1
46. Scolopendromorpha___Cryptops_hortensis_trunk___TR69529__c0__g1__i1
D..

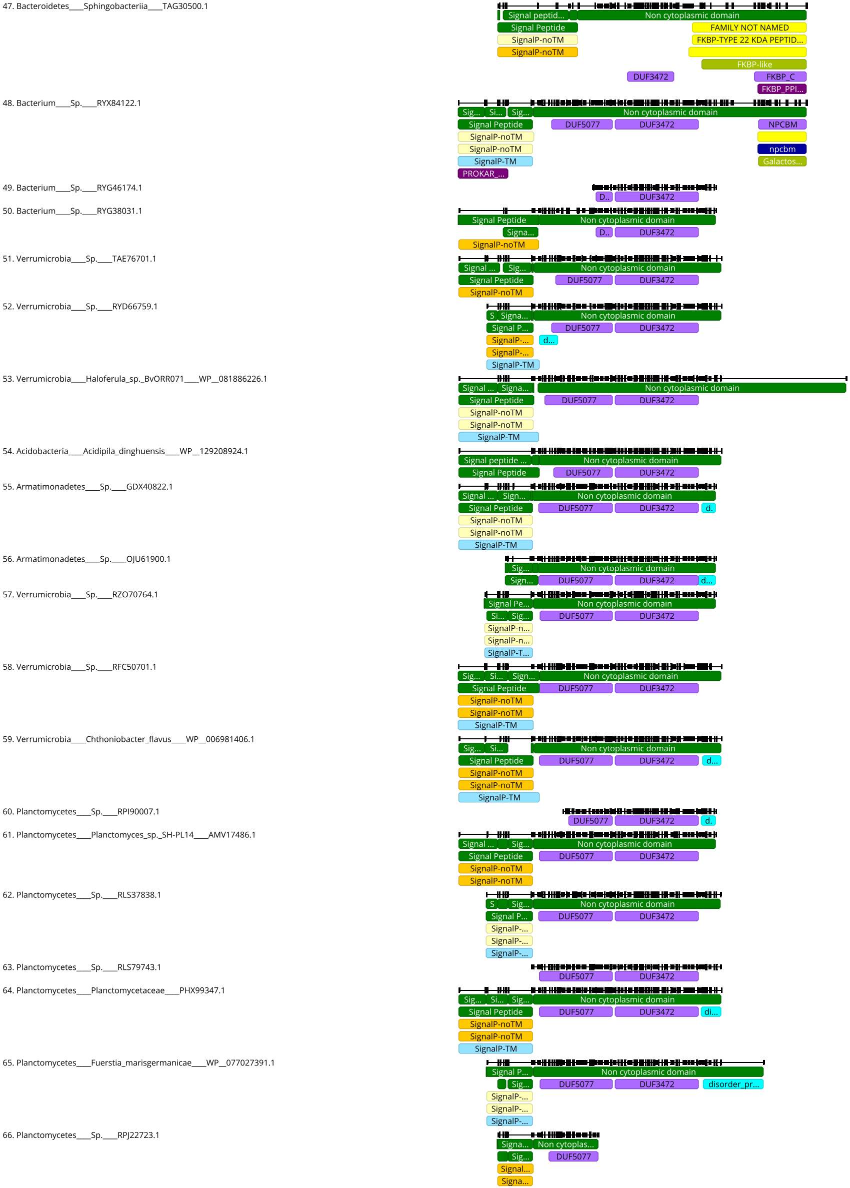
47. Bacteroidetes___Sphingobacteriia___TAG30500.1
Signal peptid...
Non cytoplasmic domain
Signal Peptide
FAMILY NOT NAMED
SignalP-noTM
FKBP-TYPE 22 KDA PEPTID...
SignalP-noTM
FKBP-like
DUF3472
FKBP_C
FKBP_PPI...
48. Bacterium___Sp.___RYX84122.1
Sig...
Si...
Sig...
Non cytoplasmic domain
Signal Peptide
DUF5077
DUF3472
NPCBM
SignalP-noTM
SignalP-noTM
npcbm
SignalP-TM
Galactos...
PROKAR_...
49. Bacterium___Sp.___RYG46174.1
D..
DUF3472
50. Bacterium___Sp.___RYG38031.1
Signal Peptide
Non cytoplasmic domain
Signa...
D..
DUF3472
SignalP-noTM
51. Verrumicrobia___Sp.___TAE76701.1
Signal ...
Sig...
Non cytoplasmic domain
Signal Peptide
DUF5077
DUF3472
SignalP-noTM
52. Verrumicrobia___Sp.___RYD66759.1
S
Signa...
Non cytoplasmic domain
Signal P...
DUF5077
DUF3472
SignalP-...
d...
SignalP-...
SignalP-TM
53. Verrumicrobia___Haloferula_sp._BvORR071___WP__081886226.1
Signal ...
Signa...
Non cytoplasmic domain
Signal Peptide
DUF5077
DUF3472
SignalP-noTM
SignalP-noTM
SignalP-TM
54. Acidobacteria___Acidipila_dinghuensis___WP__129208924.1
Signal peptide ...
Non cytoplasmic domain
Signal Peptide
DUF5077
DUF3472
55. Armatimonadetes___Sp.___GDX40822.1
Signal ...
Sign...
Non cytoplasmic domain
Signal Peptide
DUF5077
DUF3472
d.
SignalP-noTM
SignalP-noTM
SignalP-TM
56. Armatimonadetes___Sp.___OJU61900.1
Sig...
Non cytoplasmic domain
Sign...
DUF5077
DUF3472
d...
57. Verrumicrobia___Sp.___RZO70764.1
Signal Pe...
Non cytoplasmic domain
Si...
Sig...
DUF5077
DUF3472
SignalP-n...
SignalP-n...
SignalP-T...
58. Verrumicrobia___Sp.___RFC50701.1
Sig...
Si...
Sign...
Non cytoplasmic domain
Signal Peptide
DUF5077
DUF3472
SignalP-noTM
SignalP-noTM
SignalP-TM
59. Verrumicrobia___Chthoniobacter_flavus___WP__006981406.1
Sig...
Si...
Non cytoplasmic domain
Signal Peptide
DUF5077
DUF3472
d...
SignalP-noTM
SignalP-noTM
SignalP-TM
60. Planctomycetes___Sp.___RPI90007.1
DUF5077
DUF3472
d.
61. Planctomycetes___Planctomyces_sp._SH-PL14___AMV17486.1
Signal ...
Sig...
Non cytoplasmic domain
Signal Peptide
DUF5077
DUF3472
SignalP-noTM
SignalP-noTM
62. Planctomycetes___Sp.___RLS37838.1
S
Sig...
Non cytoplasmic domain
Signal P...
DUF5077
DUF3472
SignalP-...
SignalP-...
SignalP-...
63. Planctomycetes___Sp.___RLS79743.1
DUF5077
DUF3472
64. Planctomycetes___Planctomycetaceae___PHX99347.1
Sig...
Si...
Sig...
Non cytoplasmic domain
Signal Peptide
DUF5077
DUF3472
di...
SignalP-noTM
SignalP-noTM
SignalP-TM
65. Planctomycetes___Fuerstia_marisgermanicae___WP__077027391.1
Signal P...
Non cytoplasmic domain
Sig...
DUF5077
DUF3472
disorder_pr...
SignalP-...
SignalP-...
SignalP-...
66. Planctomycetes___Sp.___RPJ22723.1
Signa...
Non cytoplas...
Sig...
DUF5077
Signal...
Signa...

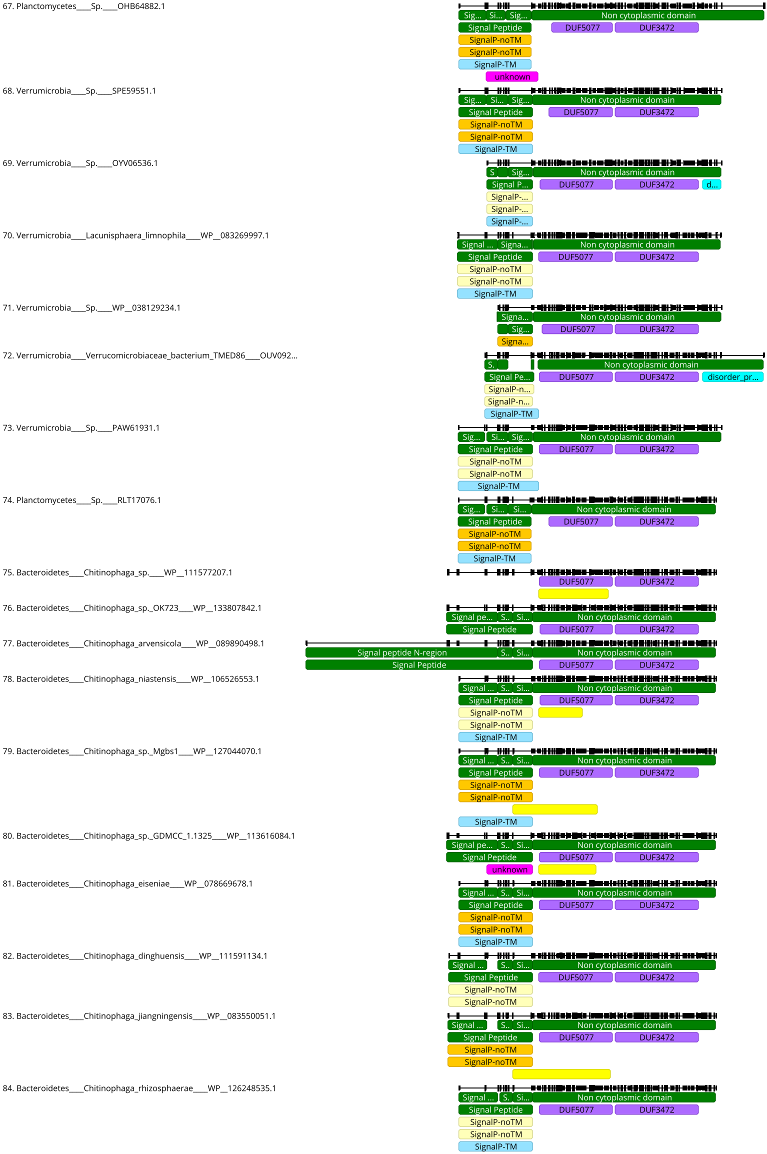

67. Planctomycetes___Sp.___OHB64882.1
Sig... Si... Sig... Non cytoplasmic domain
Signal Peptide
DUF5077
DUF3472
SignalP-noTM
SignalP-noTM
SignalP-TM
unknown
68. Verrumicrobia___Sp.___SPE59551.1
Sig... Si... Sig... Non cytoplasmic domain
Signal Peptide
DUF5077
DUF3472
SignalP-noTM
SignalP-noTM
SignalP-TM
69. Verrumicrobia___Sp.___OYV06536.1
S Sig... Non cytoplasmic domain
Signal P...
DUF5077
DUF3472
d...
SignalP-...
SignalP-...
SignalP-...
70. Verrumicrobia___Lacunisphaera_limnophila___WP__083269997.1
Signal ... Signa... Non cytoplasmic domain
Signal Peptide
DUF5077
DUF3472
SignalP-noTM
SignalP-noTM
SignalP-TM
71. Verrumicrobia___Sp.___WP__038129234.1
Signa... Non cytoplasmic domain
Sig...
DUF5077
DUF3472
Signa...
72. Verrumicrobia___Verrucomicrobiaceae_bacterium_TMED86___OUV092...
S. Non cytoplasmic domain
Signal Pe...
DUF5077
DUF3472
disorder_pr...
SignalP-n...
SignalP-n...
SignalP-TM
73. Verrumicrobia___Sp.___PAW61931.1
Sig... Si... Sig... Non cytoplasmic domain
Signal Peptide
DUF5077
DUF3472
SignalP-noTM
SignalP-noTM
SignalP-TM
74. Planctomycetes___Sp.___RLT17076.1
Sig... Si... Si... Non cytoplasmic domain
Signal Peptide
DUF5077
DUF3472
SignalP-noTM
SignalP-noTM
SignalP-TM
75. Bacteroidetes___Chitinophaga_sp.___WP__111577207.1
DUF5077
DUF3472
76. Bacteroidetes___Chitinophaga_sp._OK723___WP__133807842.1
Signal pe... S.. Si... Non cytoplasmic domain
Signal Peptide
DUF5077
DUF3472
77. Bacteroidetes___Chitinophaga_arvensicola___WP__089890498.1
Signal peptide N-region S.. Si... Non cytoplasmic domain
Signal Peptide
DUF5077
DUF3472
78. Bacteroidetes___Chitinophaga_niastensis___WP__106526553.1
Signal ... S.. Si... Non cytoplasmic domain
Signal Peptide
DUF5077
DUF3472
SignalP-noTM
SignalP-noTM
SignalP-TM
79. Bacteroidetes___Chitinophaga_sp._Mgbs1___WP__127044070.1
Signal ... S.. Si... Non cytoplasmic domain
Signal Peptide
DUF5077
DUF3472
SignalP-noTM
SignalP-noTM
SignalP-TM
80. Bacteroidetes___Chitinophaga_sp._GDMCC_1.1325___WP__113616084.1
Signal pe... S.. Si... Non cytoplasmic domain
Signal Peptide
DUF5077
DUF3472
unknown
81. Bacteroidetes___Chitinophaga_eiseniae___WP__078669678.1
Signal ... S.. Si... Non cytoplasmic domain
Signal Peptide
DUF5077
DUF3472
SignalP-noTM
SignalP-noTM
SignalP-TM
82. Bacteroidetes___Chitinophaga_dinghuensis___WP__111591134.1
Signal ... S.. Si... Non cytoplasmic domain
Signal Peptide
DUF5077
DUF3472
SignalP-noTM
SignalP-noTM
83. Bacteroidetes___Chitinophaga_jiangningensis___WP__083550051.1
Signal ... S.. Si... Non cytoplasmic domain
Signal Peptide
DUF5077
DUF3472
SignalP-noTM
SignalP-noTM
84. Bacteroidetes___Chitinophaga_rhizosphaerae___WP__126248535.1
Signal ... S. Si... Non cytoplasmic domain
Signal Peptide
DUF5077
DUF3472
SignalP-noTM
SignalP-noTM
SignalP-TM

85. Bacteroidetes___Chitinophaga_sp._XJ-2___WP__109695949.1

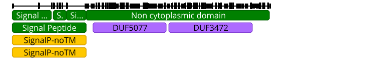

86. Bacteroidetes___Chitinophaga_barathri___WP__120519397.1

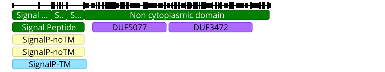

87. Bacteroidetes___Chitinophaga_sp._ZY74___WP__123846167.1

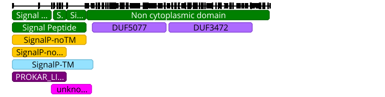

88. Bacteroidetes___Chitinophaga_sp._T22___WP__119077767.1

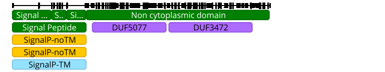

89. Bacteroidetes___Chitinophaga_niabensis___SIO45708.1

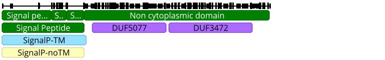

90. Bacteroidetes___Chitinophaga_sp._GDMCC_1.1288___WP__116975596.1

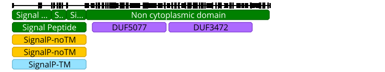

91. Bacteroidetes___Chitinophaga_terrae___WP__089763293.1

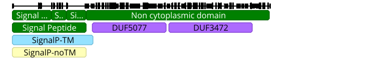

92. Bacteroidetes___Chitinophaga_caeni___WP__098193028.1

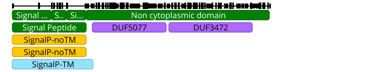

93. Bacteroidetes___Chitinophaga_skermanii___RAI98673.1

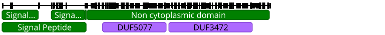

94. Bacteroidetes___Flexithrix_dorotheae___WP__020527010.1

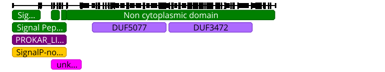

95. Bacteroidetes___Sp.___PWU02726.1

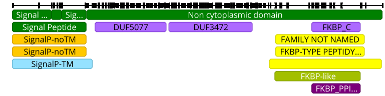

96. Bacteroidetes___Sediminibacterium_goheungense___WP__133475008.1

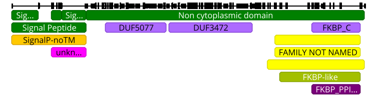

97. Bacteroidetes___Chitinophagaceae___TAJ66198.1

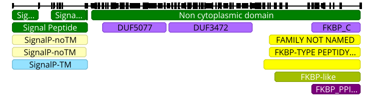

98. Bacteroidetes___Sphingobacteriia___TAH06835.1

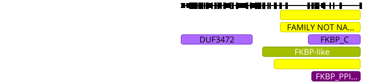

99. Bacteroidetes___Sediminibacterium_salmoneum___WP__051417159.1

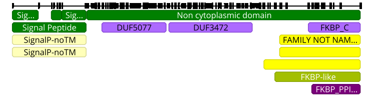

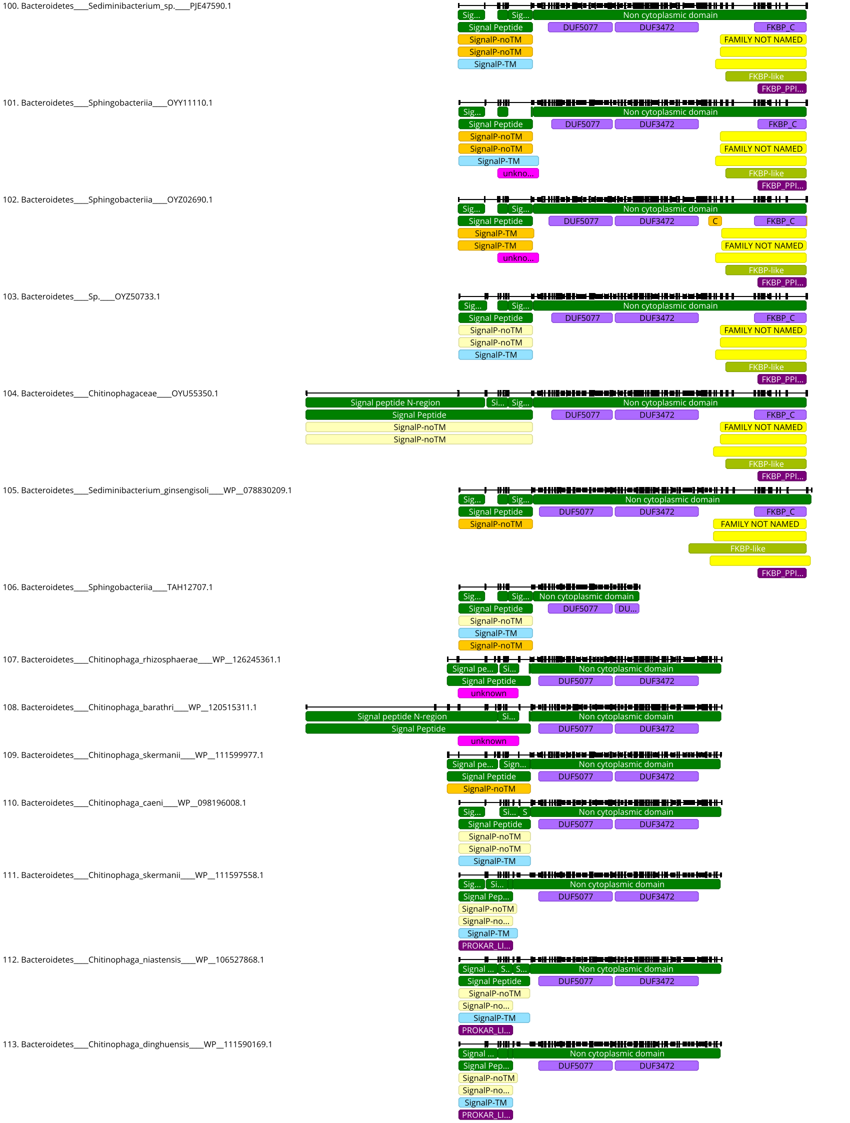
100. Bacteroidetes___Sediminibacterium_sp.___PJE47590.1
Sig... Sig... Non cytoplasmic domain
Signal Peptide DUF5077 DUF3472 FKBP_C
SignalP-noTM FAMILY NOT NAMED
SignalP-noTM
SignalP-TM
FKBP-like
FKBP_PPI...
101. Bacteroidetes___Sphingobacteriia___OYY11110.1
Sig... Non cytoplasmic domain
Signal Peptide DUF5077 DUF3472 FKBP_C
SignalP-noTM
SignalP-noTM FAMILY NOT NAMED
SignalP-TM
unkno... FKBP-like
FKBP_PPI...
102. Bacteroidetes___Sphingobacteriia___OYZ02690.1
Sig... Sig... Non cytoplasmic domain
Signal Peptide DUF5077 DUF3472 C FKBP_C
SignalP-TM
SignalP-TM FAMILY NOT NAMED
unkno...
FKBP-like
FKBP_PPI...
103. Bacteroidetes___Sp.___OYZ50733.1
Sig... Sig... Non cytoplasmic domain
Signal Peptide DUF5077 DUF3472 FKBP_C
SignalP-noTM FAMILY NOT NAMED
SignalP-noTM
SignalP-TM
FKBP-like
FKBP_PPI...
104. Bacteroidetes___Chitinophagaceae___OYU55350.1
Signal peptide N-region Si... Sig... Non cytoplasmic domain
Signal Peptide DUF5077 DUF3472 FKBP_C
SignalP-noTM FAMILY NOT NAMED
SignalP-noTM
FKBP-like
FKBP_PPI...
105. Bacteroidetes___Sediminibacterium_ginsengisoli___WP__078830209.1
Sig... Sig... Non cytoplasmic domain
Signal Peptide DUF5077 DUF3472 FKBP_C
SignalP-noTM FAMILY NOT NAMED
FKBP-like
FKBP_PPI...
106. Bacteroidetes___Sphingobacteriia___TAH12707.1
Sig... Sig... Non cytoplasmic domain
Signal Peptide DUF5077 DU...
SignalP-noTM
SignalP-TM
SignalP-noTM
107. Bacteroidetes___Chitinophaga_rhizosphaerae___WP__126245361.1
Signal pe... Si... Non cytoplasmic domain
Signal Peptide DUF5077 DUF3472
unknown
108. Bacteroidetes___Chitinophaga_barathri___WP__120515311.1
Signal peptide N-region Si... Non cytoplasmic domain
Signal Peptide DUF5077 DUF3472
unknown
109. Bacteroidetes___Chitinophaga_skermanii___WP__111599977.1
Signal pe... Sign... Non cytoplasmic domain
Signal Peptide DUF5077 DUF3472
SignalP-noTM
110. Bacteroidetes___Chitinophaga_caeni___WP__098196008.1
Sig... Si... S Non cytoplasmic domain
Signal Peptide DUF5077 DUF3472
SignalP-noTM
SignalP-noTM
SignalP-TM
111. Bacteroidetes___Chitinophaga_skermanii___WP__111597558.1
Sig... Si... Non cytoplasmic domain
Signal Pep... DUF5077 DUF3472
SignalP-noTM
SignalP-no...
SignalP-TM
PROKAR_LI...
112. Bacteroidetes___Chitinophaga_niastensis___WP__106527868.1
Signal ... S... S... Non cytoplasmic domain
Signal Peptide DUF5077 DUF3472
SignalP-noTM
SignalP-no...
SignalP-TM
PROKAR_LI...
113. Bacteroidetes___Chitinophaga_dinghuensis___WP__111590169.1
Signal ... Non cytoplasmic domain
Signal Pep... DUF5077 DUF3472
SignalP-noTM
SignalP-no...
SignalP-TM
PROKAR_LI...

114. Bacteroidetes___Chitinophaga_jiangningensis___WP__073083257.1
115. Bacteroidetes___Chitinophaga_caeni___WP__098195791.1
116. Streptophyta___Selaginella_moellendorffii___XP__002971429.1
117. Bacteroidetes___Chitinophaga_sp._CF118___WP__090099799.1
118. Bacteroidetes___Chitinophaga_sp._YR573___WP__089788083.1
119. Bacteroidetes___Mucilaginibacter_sp._YR332___WP__121226936.1
120. Bacteroidetes___Mucilaginibacter_pineti___WP__091146115.1
121. Bacteroidetes___Sphingobacteriales___OJW60798.1
122. Bacteroidetes___Flavisolibacter_sp._X7X___WP__121354570.1
123. Bacteroidetes___Chitinophagaceae___WP__086102221.1
124. Bacteroidetes___Chitinophagaceae_K23C18032701___WP__116847522.1
125. Bacteroidetes___Chitinophaga_parva___PUZ26194.1
126. Bacteroidetes___Mucilaginibacter_pineti___WP__091155940.1
127. Lithobiomorpha___Lithobius_sp.___TR14653__c0__g1__i1
128. Lithobiomorpha___Lithobius_sp.___TR14653__c0__g1__i6
129. Lithobiomorpha___Lithobius_sp.___TR20185__c0__g1__i1
130. Lithobiomorpha___Lithobius_forficatus_VG___c542310__g2__i2
131. Lithobiomorpha___Lithobius_forficatus_WB___GBKE01005448.1
132. Lithobiomorpha___Eupolybothrus_cavernicolus___scaffold10118
133. Lithobiomorpha___Eupolybothrus_cavernicolus___c240070
134. Bacterium___Sp.___RYX84168.1
135. Verrumicrobia___Sp.___RME90600.1
136. Acidobacteria___Sulfopaludibacter_sp._SbA3___SPE28318.1

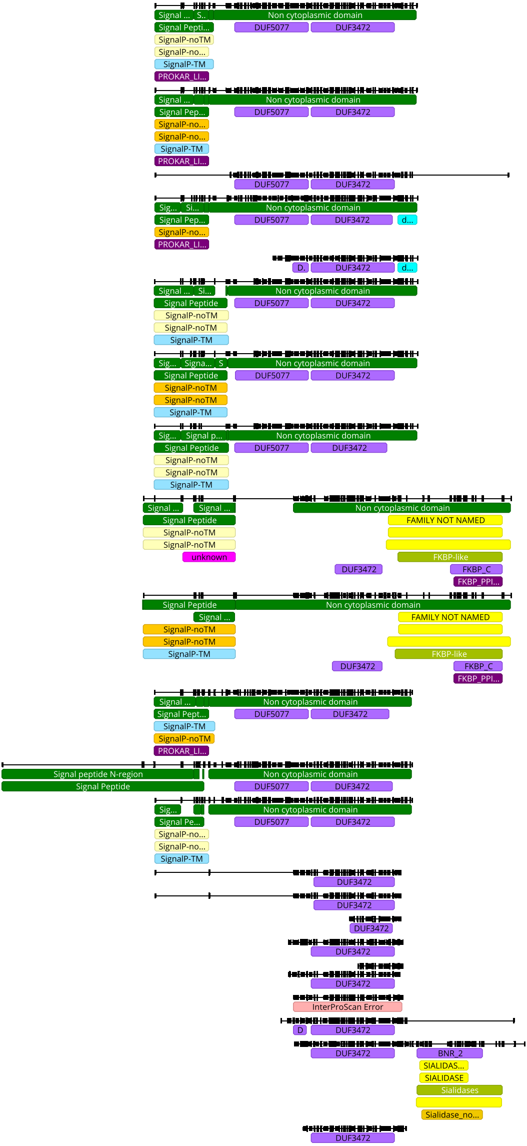

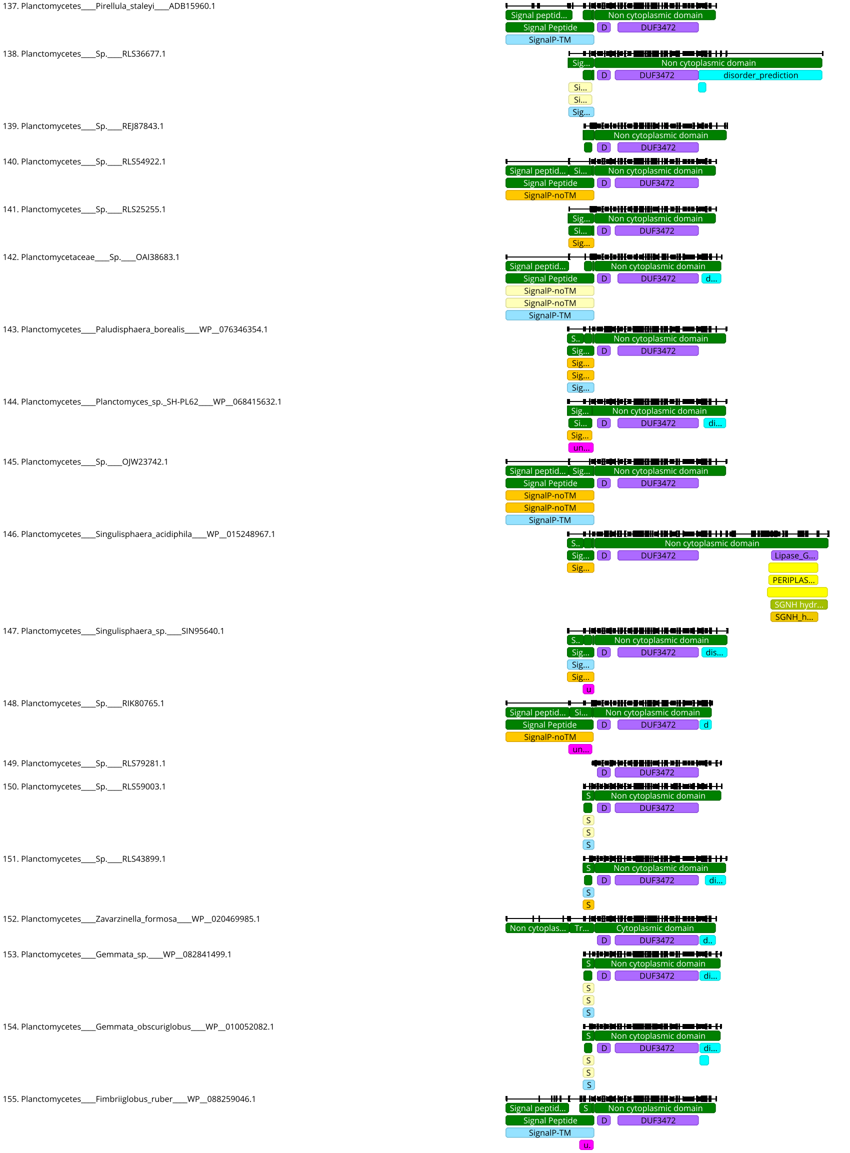
137. Planctomycetes___Pirellula_staleyi___ADB15960.1
Signal peptid... Non cytoplasmic domain
Signal Peptide D DUF3472
SignalP-TM
138. Planctomycetes___Sp.___RLS36677.1
Sig... Non cytoplasmic domain
D DUF3472 disorder_prediction
Si...
Si...
Sig...
139. Planctomycetes___Sp.___REJ87843.1
Non cytoplasmic domain
D DUF3472
140. Planctomycetes___Sp.___RLS54922.1
Signal peptid... Si... Non cytoplasmic domain
Signal Peptide D DUF3472
SignalP-noTM
141. Planctomycetes___Sp.___RLS25255.1
Sig... Non cytoplasmic domain
Si... D DUF3472
Sig...
142. Planctomycetaceae___Sp.___OAI38683.1
Signal peptid... Non cytoplasmic domain
Signal Peptide D DUF3472 d...
SignalP-noTM
SignalP-noTM
SignalP-TM
143. Planctomycetes___Paludisphaera_borealis___WP__076346354.1
S.. Non cytoplasmic domain
Sig... D DUF3472
Sig...
Sig...
Sig...
144. Planctomycetes___Planctomyces_sp._SH-PL62___WP__068415632.1
Sig... Non cytoplasmic domain
Si... D DUF3472 di...
Sig...
un...
145. Planctomycetes___Sp.___OJW23742.1
Signal peptid... Sig... Non cytoplasmic domain
Signal Peptide D DUF3472
SignalP-noTM
SignalP-noTM
SignalP-TM
146. Planctomycetes___Singulisphaera_acidiphila___WP__015248967.1
S.. Non cytoplasmic domain
Sig... D DUF3472 Lipase_G...
Sig...
PERIPLAS...
SGNH hydr...
SGNH_h...
147. Planctomycetes___Singulisphaera_sp.___SIN95640.1
S.. Non cytoplasmic domain
Sig... D DUF3472 dis...
Sig...
Sig...
u
148. Planctomycetes___Sp.___RIK80765.1
Signal peptid... Si... Non cytoplasmic domain
Signal Peptide D DUF3472 d
SignalP-noTM
un...
149. Planctomycetes___Sp.___RLS79281.1
D DUF3472
150. Planctomycetes___Sp.___RLS59003.1
S Non cytoplasmic domain
D DUF3472
S
S
S
151. Planctomycetes___Sp.___RLS43899.1
S Non cytoplasmic domain
D DUF3472 di...
S
S
152. Planctomycetes___Zavarzinella_formosa___WP__020469985.1
Non cytoplas... Tr... Cytoplasmic domain
D DUF3472 d..
153. Planctomycetes___Gemmata_sp.___WP__082841499.1
S Non cytoplasmic domain
D DUF3472 di...
S
S
S
154. Planctomycetes___Gemmata_obscuriglobus___WP__010052082.1
S Non cytoplasmic domain
D DUF3472 di...
S
S
S
155. Planctomycetes___Fimbriiglobus_ruber___WP__088259046.1
Signal peptid... S Non cytoplasmic domain
Signal Peptide D DUF3472
SignalP-TM
u.

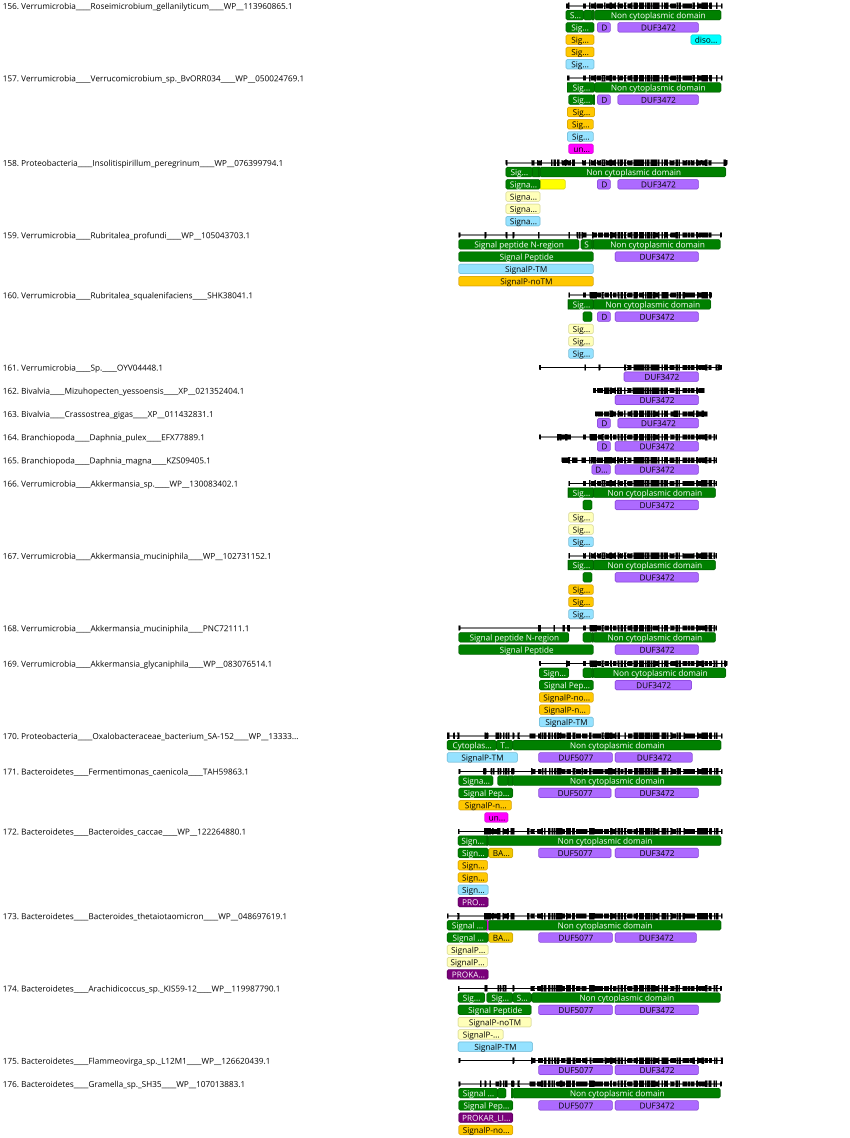

156. Verrumicrobia___Roseimicrobium_gellanilyticum___WP__113960865.1
S...
Non cytoplasmic domain
Sig...
D
DUF3472
Sig...
diso...
Sig...
Sig...
157. Verrumicrobia___Verrucomicrobium_sp._BvORR034___WP__050024769.1
Sig...
Non cytoplasmic domain
Sig...
D
DUF3472
Sig...
Sig...
Sig...
un...
158. Proteobacteria___Insolitispirillum_peregrinum___WP__076399794.1
Sig...
Non cytoplasmic domain
Signa...
D
DUF3472
Signa...
Signa...
Signa...
159. Verrumicrobia___Rubritalea_profundi___WP__105043703.1
Signal peptide N-region
S
Non cytoplasmic domain
Signal Peptide
DUF3472
SignalP-TM
SignalP-noTM
160. Verrumicrobia___Rubritalea_squalenifaciens___SHK38041.1
Sig...
Non cytoplasmic domain
D
DUF3472
Sig...
Sig...
Sig...
161. Verrumicrobia___Sp.___OYV04448.1
DUF3472
162. Bivalvia___Mizuhopecten_yessoensis___XP__021352404.1
DUF3472
163. Bivalvia___Crassostrea_gigas___XP__011432831.1
D
DUF3472
164. Branchiopoda___Daphnia_pulex___EFX77889.1
D
DUF3472
165. Branchiopoda___Daphnia_magna___KZS09405.1
D...
DUF3472
166. Verrumicrobia___Akkermansia_sp.___WP__130083402.1
Sig...
Non cytoplasmic domain
DUF3472
Sig...
Sig...
Sig...
167. Verrumicrobia___Akkermansia_muciniphila___WP__102731152.1
Sig...
Non cytoplasmic domain
DUF3472
Sig...
Sig...
Sig...
168. Verrumicrobia___Akkermansia_muciniphila___PNC72111.1
Signal peptide N-region
Non cytoplasmic domain
Signal Peptide
DUF3472
169. Verrumicrobia___Akkermansia_glycaniphila___WP__083076514.1
Sign...
Non cytoplasmic domain
Signal Pep...
DUF3472
SignalP-no...
SignalP-n...
SignalP-TM
170. Proteobacteria___Oxalobacteraceae_bacterium_SA-152___WP__13333...
Cytoplas...
T...
Non cytoplasmic domain
SignalP-TM
DUF5077
DUF3472
171. Bacteroidetes___Fermentimonas_caenicola___TAH59863.1
Signa...
Non cytoplasmic domain
Signal Pep...
DUF5077
DUF3472
SignalP-n...
un...
172. Bacteroidetes___Bacteroides_caccae___WP__122264880.1
Sign...
Non cytoplasmic domain
Sign...
BA...
DUF5077
DUF3472
Sign...
Sign...
Sign...
PRO...
173. Bacteroidetes___Bacteroides_thetaiotaomicron___WP__048697619.1
Signal ...
Non cytoplasmic domain
Signal ...
BA...
DUF5077
DUF3472
SignalP...
SignalP...
PROKA...
174. Bacteroidetes___Arachidicoccus_sp._KIS59-12___WP__119987790.1
Sig...
Sig...
S...
Non cytoplasmic domain
Signal Peptide
DUF5077
DUF3472
SignalP-noTM
SignalP-...
SignalP-TM
175. Bacteroidetes___Flammeovirga_sp._L12M1___WP__126620439.1
DUF5077
DUF3472
176. Bacteroidetes___Gramella_sp._SH35___WP__107013883.1
Signal ...
Non cytoplasmic domain
Signal Pep...
DUF5077
DUF3472
PROKAR_LI...
SignalP-no...

177. Bacteroidetes___Sp.___PTM08412.1

178. Bacteroidetes___Sp.___PSR11427.1

179. Bacteroidetes___Lewinella_persica___WP__020569455.1

180. Bacteroidetes___Marinifilum_flexuosum___WP__120240594.1

181. Bacteroidetes___Marinilabiliaceae___WP__130641095.1

182. Bacteroidetes___Flavobacteriaceae_BH-SD17___WP__123896717.1

183. Bacteroidetes___Gelidibacter_gilvus___WP__129017482.1

184. Bacteroidetes___Gelidibacter_mesophilus___WP__051209875.1

185. Bacteroidetes___Gelidibacter_sediminis___WP__133756516.1

186. Bacteroidetes___Lutibacter_sp._SM1352___WP__117881812.1

187. Bacteroidetes___Polaribacter_sp._ALD11___WP__100944855.1

188. Bacteroidetes___Polaribacter_atrinae___WP__068449239.1

189. Bacteroidetes___Aquimarina_sediminis___WP__103071825.1

190. Bacteroidetes___Aquimarina_sp._Aq135___WP__108870280.1

191. Bacteroidetes___Aquimarina_sp.___WP__101450127.1

192. Bacteroidetes___Aquimarina_sp._Aq349___WP__108808350.1

193. Bacteroidetes___Aquimarina_sp._AU58___WP__109853543.1

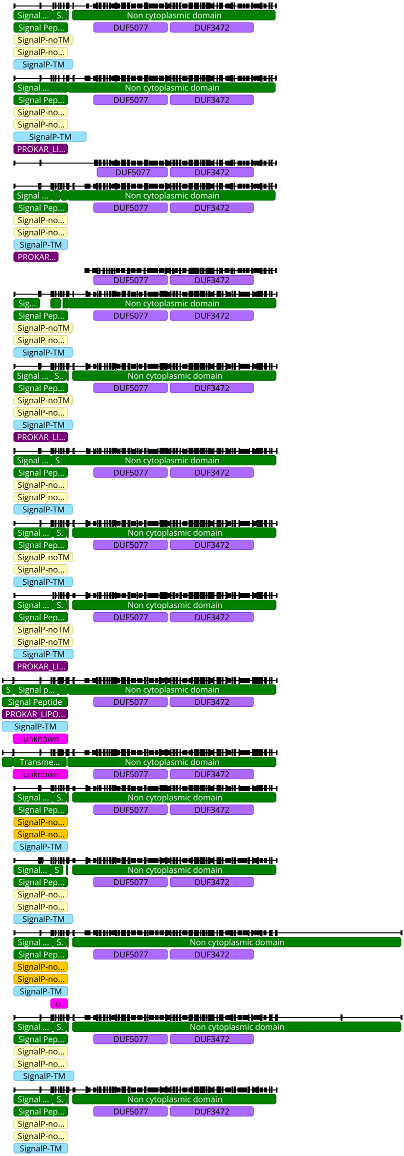

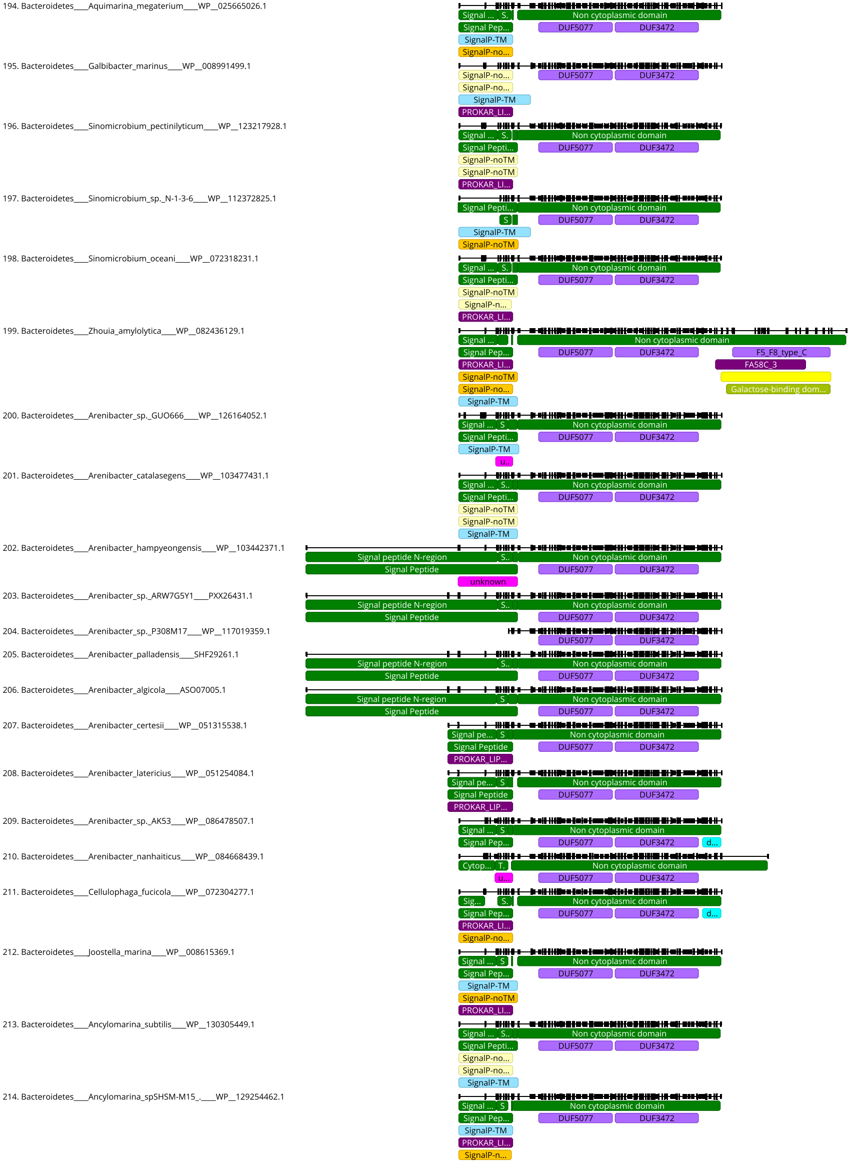
194. Bacteroidetes___Aquimarina_megaterium___WP__025665026.1
Signal ... S. Non cytoplasmic domain
Signal Pep... DUF5077 DUF3472
SignalP-TM
SignalP-no...
195. Bacteroidetes___Galbibacter_marinus___WP__008991499.1
SignalP-no... DUF5077 DUF3472
SignalP-no...
SignalP-TM
PROKAR_LI...
196. Bacteroidetes___Sinomicrobium_pectinilyticum___WP__123217928.1
Signal ... S. Non cytoplasmic domain
Signal Pepti... DUF5077 DUF3472
SignalP-noTM
SignalP-noTM
PROKAR_LI...
197. Bacteroidetes___Sinomicrobium_sp._N-1-3-6___WP__112372825.1
Signal Pepti... Non cytoplasmic domain
S DUF5077 DUF3472
SignalP-TM
SignalP-noTM
198. Bacteroidetes___Sinomicrobium_oceani___WP__072318231.1
Signal ... S. Non cytoplasmic domain
Signal Pepti... DUF5077 DUF3472
SignalP-noTM
SignalP-n...
PROKAR_LI...
199. Bacteroidetes___Zhouia_amylolytica___WP__082436129.1
Signal ... Non cytoplasmic domain
Signal Pep... DUF5077 DUF3472 F5_F8_type_C
PROKAR_LI... FA58C_3
SignalP-noTM
SignalP-no... Galactose-binding dom...
SignalP-TM
200. Bacteroidetes___Arenibacter_sp._GUO666___WP__126164052.1
Signal ... S Non cytoplasmic domain
Signal Pepti... DUF5077 DUF3472
SignalP-TM
u..
201. Bacteroidetes___Arenibacter_catalasegens___WP__103477431.1
Signal ... S.. Non cytoplasmic domain
Signal Pepti... DUF5077 DUF3472
SignalP-noTM
SignalP-noTM
SignalP-TM
202. Bacteroidetes___Arenibacter_hampyeongensis___WP__103442371.1
Signal peptide N-region S.. Non cytoplasmic domain
Signal Peptide DUF5077 DUF3472
unknown
203. Bacteroidetes___Arenibacter_sp._ARW7G5Y1___PXX26431.1
Signal peptide N-region S.. Non cytoplasmic domain
Signal Peptide DUF5077 DUF3472
204. Bacteroidetes___Arenibacter_sp._P308M17___WP__117019359.1
DUF5077 DUF3472
205. Bacteroidetes___Arenibacter_palladensis___SHF29261.1
Signal peptide N-region S.. Non cytoplasmic domain
Signal Peptide DUF5077 DUF3472
206. Bacteroidetes___Arenibacter_algicola___ASO07005.1
Signal peptide N-region S Non cytoplasmic domain
Signal Peptide DUF5077 DUF3472
207. Bacteroidetes___Arenibacter_certesii___WP__051315538.1
Signal pe... S Non cytoplasmic domain
Signal Peptide DUF5077 DUF3472
PROKAR_LIP...
208. Bacteroidetes___Arenibacter_latericius___WP__051254084.1
Signal pe... S Non cytoplasmic domain
Signal Peptide DUF5077 DUF3472
PROKAR_LIP...
209. Bacteroidetes___Arenibacter_sp._AK53___WP__086478507.1
Signal ... S Non cytoplasmic domain
Signal Pep... DUF5077 DUF3472 d...
210. Bacteroidetes___Arenibacter_nanhaiticus___WP__084668439.1
Cytop... T. Non cytoplasmic domain
u... DUF5077 DUF3472
211. Bacteroidetes___Cellulophaga_fucicola___WP__072304277.1
Sig... S. Non cytoplasmic domain
Signal Pep... DUF5077 DUF3472 d...
PROKAR_LI...
SignalP-no...
212. Bacteroidetes___Joostella_marina___WP__008615369.1
Signal ... S Non cytoplasmic domain
Signal Pep... DUF5077 DUF3472
SignalP-TM
SignalP-noTM
PROKAR_LI...
213. Bacteroidetes___Ancylomarina_subtilis___WP__130305449.1
Signal ... S.. Non cytoplasmic domain
Signal Pepti... DUF5077 DUF3472
SignalP-no...
SignalP-no...
SignalP-TM
214. Bacteroidetes___Ancylomarina_spSHSM-M15_.___WP__129254462.1
Signal ... S Non cytoplasmic domain
Signal Pep... DUF5077 DUF3472
SignalP-TM
PROKAR_LI...
SignalP-n...

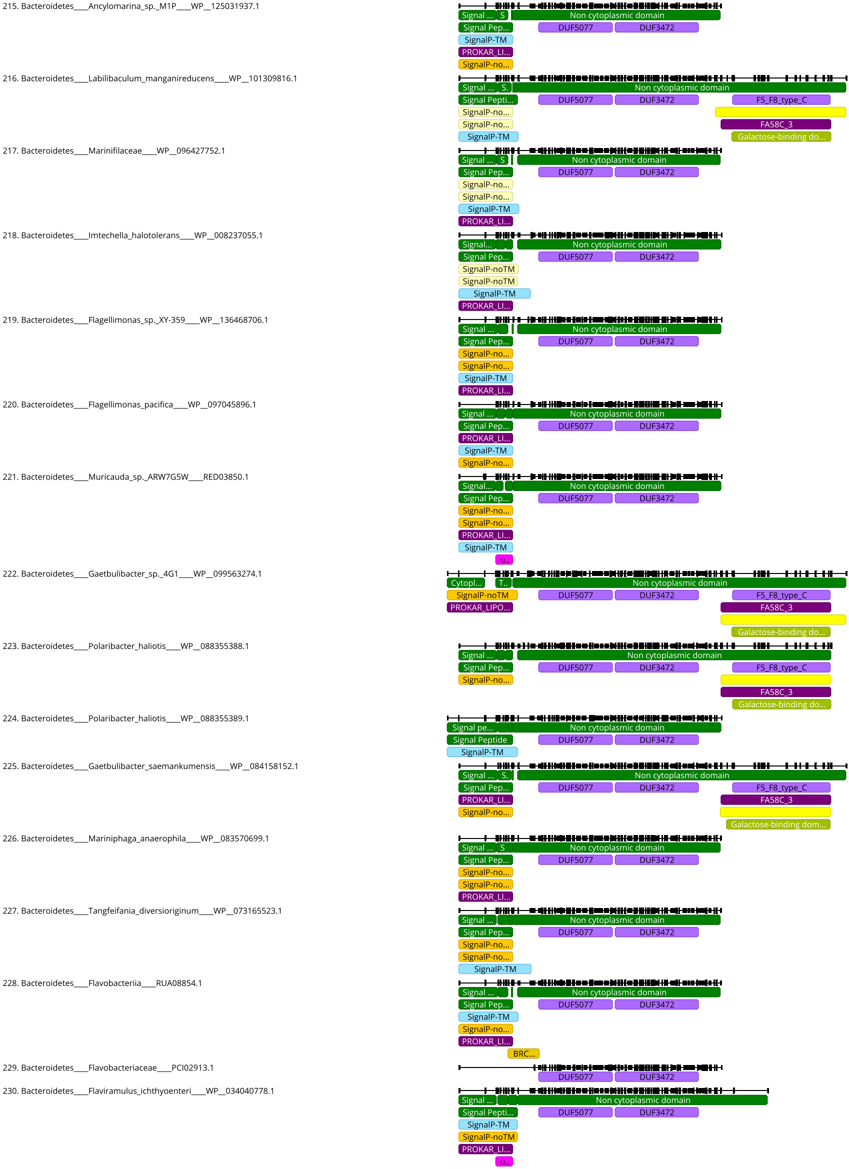
215. Bacteroidetes___Ancylomarina_sp._M1P___WP__125031937.1
Signal ...
S
Non cytoplasmic domain
Signal Pep...
DUF5077
DUF3472
SignalP-TM
PROKAR_LI...
SignalP-no...
216. Bacteroidetes___Labilibaculum_manganireducens___WP__101309816.1
Signal ...
S.
Non cytoplasmic domain
Signal Pepti...
DUF5077
DUF3472
F5_F8_type_C
SignalP-no...
SignalP-no...
FA58C_3
SignalP-TM
Galactose-binding do...
217. Bacteroidetes___Marinifilaceae___WP__096427752.1
Signal ...
S
Non cytoplasmic domain
Signal Pep...
DUF5077
DUF3472
SignalP-no...
SignalP-no...
SignalP-TM
PROKAR_LI...
218. Bacteroidetes___Imtechella_halotolerans___WP__008237055.1
Signal...
Non cytoplasmic domain
Signal Pep...
DUF5077
DUF3472
SignalP-noTM
SignalP-noTM
SignalP-TM
PROKAR_LI...
219. Bacteroidetes___Flagellimonas_sp._XY-359___WP__136468706.1
Signal ...
Non cytoplasmic domain
Signal Pep...
DUF5077
DUF3472
SignalP-no...
SignalP-no...
SignalP-TM
PROKAR_LI...
220. Bacteroidetes___Flagellimonas_pacifica___WP__097045896.1
Signal ...
Non cytoplasmic domain
Signal Pep...
DUF5077
DUF3472
PROKAR_LI...
SignalP-TM
SignalP-no...
221. Bacteroidetes___Muricauda_sp._ARW7G5W___RED03850.1
Signal...
Non cytoplasmic domain
Signal Pep...
DUF5077
DUF3472
SignalP-no...
SignalP-no...
PROKAR_LI...
SignalP-TM
u..
222. Bacteroidetes___Gaetbulibacter_sp._4G1___WP__099563274.1
Cytopl...
T..
Non cytoplasmic domain
SignalP-noTM
DUF5077
DUF3472
F5_F8_type_C
PROKAR_LIPO...
FA58C_3
Galactose-binding do...
223. Bacteroidetes___Polaribacter_haliotis___WP__088355388.1
Signal ...
Non cytoplasmic domain
Signal Pep...
DUF5077
DUF3472
F5_F8_type_C
SignalP-no...
FA58C_3
Galactose-binding do...
224. Bacteroidetes___Polaribacter_haliotis___WP__088355389.1
Signal pe...
Non cytoplasmic domain
Signal Peptide
DUF5077
DUF3472
SignalP-TM
225. Bacteroidetes___Gaetbulibacter_saemankumensis___WP__084158152.1
Signal ...
S.
Non cytoplasmic domain
Signal Pep...
DUF5077
DUF3472
F5_F8_type_C
PROKAR_LI...
FA58C_3
SignalP-no...
Galactose-binding dom...
226. Bacteroidetes___Mariniphaga_anaerophila___WP__083570699.1
Signal ...
S
Non cytoplasmic domain
Signal Pep...
DUF5077
DUF3472
SignalP-no...
SignalP-no...
PROKAR_LI...
227. Bacteroidetes___Tangfeifania_diversioriginum___WP__073165523.1
Signal ...
Non cytoplasmic domain
Signal Pep...
DUF5077
DUF3472
SignalP-no...
SignalP-no...
SignalP-TM
228. Bacteroidetes___Flavobacteriia___RUA08854.1
Signal ...
Non cytoplasmic domain
Signal Pep...
DUF5077
DUF3472
SignalP-TM
SignalP-no...
PROKAR_LI...
BRC...
229. Bacteroidetes___Flavobacteriaceae___PCI02913.1
DUF5077
DUF3472
230. Bacteroidetes___Flaviramulus_ichthyoenteri___WP__034040778.1
Signal ...
Non cytoplasmic domain
Signal Pepti...
DUF5077
DUF3472
SignalP-TM
SignalP-noTM
PROKAR_LI...
u..

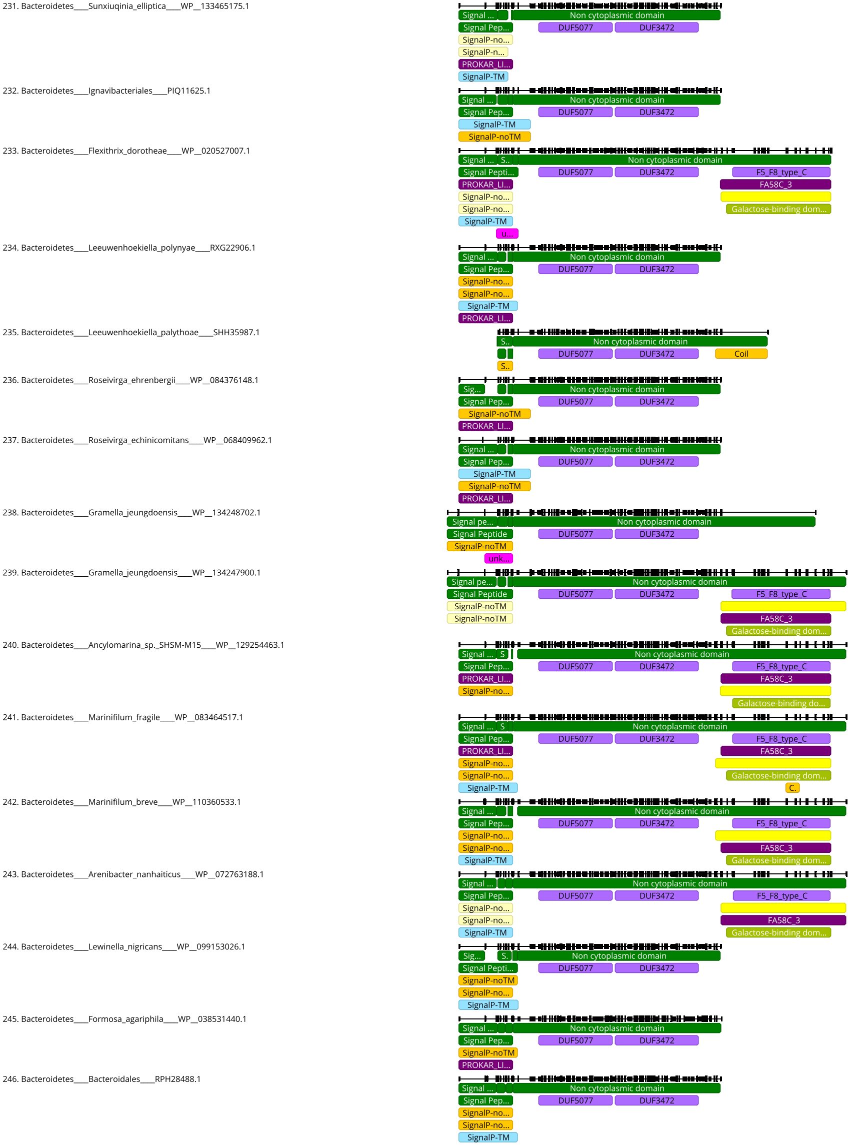
231. Bacteroidetes___Sunxiuqinia_elliptica___WP__133465175.1
Signal ...
Non cytoplasmic domain
Signal Pep...
DUF5077
DUF3472
SignalP-no...
SignalP-n...
PROKAR_LI...
SignalP-TM
232. Bacteroidetes___Ignavibacteriales___PIQ11625.1
Signal ...
Non cytoplasmic domain
Signal Pep...
DUF5077
DUF3472
SignalP-TM
SignalP-noTM
233. Bacteroidetes___Flexithrix_dorotheae___WP__020527007.1
Signal ...
S..
Non cytoplasmic domain
Signal Pepti...
DUF5077
DUF3472
F5_F8_type_C
PROKAR_LI...
FA58C_3
SignalP-no...
SignalP-no...
Galactose-binding dom...
SignalP-TM
u...
234. Bacteroidetes___Leeuwenhoekiella_polynyae___RXG22906.1
Signal ...
Non cytoplasmic domain
Signal Pep...
DUF5077
DUF3472
SignalP-no...
SignalP-no...
SignalP-TM
PROKAR_LI...
235. Bacteroidetes___Leeuwenhoekiella_palythoae___SHH35987.1
S..
Non cytoplasmic domain
DUF5077
DUF3472
Coil
S..
236. Bacteroidetes___Roseivirga_ehrenbergii___WP__084376148.1
Sig...
Non cytoplasmic domain
Signal Pep...
DUF5077
DUF3472
SignalP-noTM
PROKAR_LI...
237. Bacteroidetes___Roseivirga_echinicomitans___WP__068409962.1
Signal ...
Non cytoplasmic domain
Signal Pep...
DUF5077
DUF3472
SignalP-TM
SignalP-noTM
PROKAR_LI...
238. Bacteroidetes___Gramella_jeungdoensis___WP__134248702.1
Signal pe...
Non cytoplasmic domain
Signal Peptide
DUF5077
DUF3472
SignalP-noTM
unk...
239. Bacteroidetes___Gramella_jeungdoensis___WP__134247900.1
Signal pe...
Non cytoplasmic domain
Signal Peptide
DUF5077
DUF3472
F5_F8_type_C
SignalP-noTM
SignalP-noTM
FA58C_3
Galactose-binding dom...
240. Bacteroidetes___Ancylomarina_sp._SHSM-M15___WP__129254463.1
Signal ...
S
Non cytoplasmic domain
Signal Pep...
DUF5077
DUF3472
F5_F8_type_C
PROKAR_LI...
FA58C_3
SignalP-no...
Galactose-binding do...
241. Bacteroidetes___Marinifilum_fragile___WP__083464517.1
Signal ...
S
Non cytoplasmic domain
Signal Pep...
DUF5077
DUF3472
F5_F8_type_C
PROKAR_LI...
FA58C_3
SignalP-no...
SignalP-no...
Galactose-binding dom...
SignalP-TM
C.
242. Bacteroidetes___Marinifilum_breve___WP__110360533.1
Signal ...
Non cytoplasmic domain
Signal Pep...
DUF5077
DUF3472
F5_F8_type_C
SignalP-no...
SignalP-no...
FA58C_3
SignalP-TM
Galactose-binding dom...
243. Bacteroidetes___Arenibacter_nanhaiticus___WP__072763188.1
Signal ...
Non cytoplasmic domain
Signal Pep...
DUF5077
DUF3472
F5_F8_type_C
SignalP-no...
SignalP-no...
FA58C_3
SignalP-TM
Galactose-binding dom...
244. Bacteroidetes___Lewinella_nigricans___WP__099153026.1
Sig...
S.
Non cytoplasmic domain
Signal Pepti...
DUF5077
DUF3472
SignalP-noTM
SignalP-no...
SignalP-TM
245. Bacteroidetes___Formosa_agariphila___WP__038531440.1
Signal ...
Non cytoplasmic domain
Signal Pep...
DUF5077
DUF3472
SignalP-noTM
PROKAR_LI...
246. Bacteroidetes___Bacteroidales___RPH28488.1
Signal ...
Non cytoplasmic domain
Signal Pep...
DUF5077
DUF3472
SignalP-no...
SignalP-no...
SignalP-TM

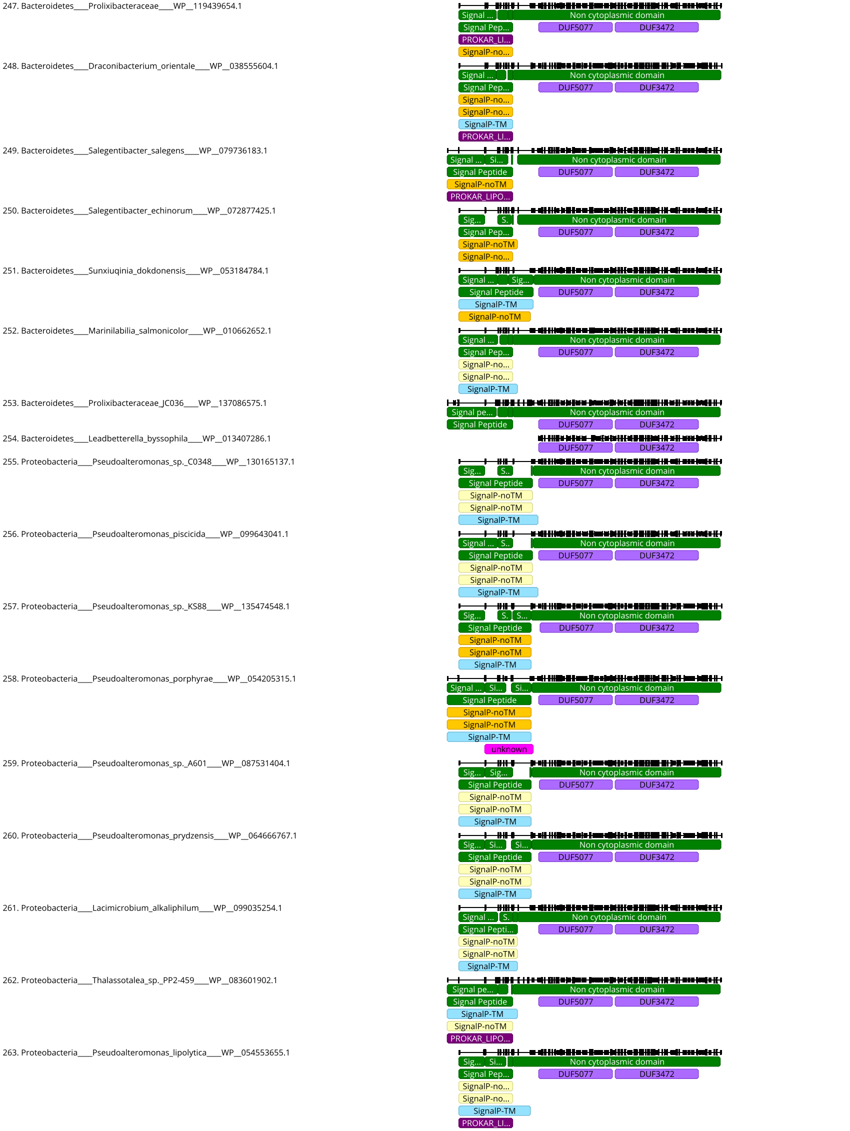
247. Bacteroidetes___Prolixibacteraceae___WP__119439654.1
Signal ...
Non cytoplasmic domain
Signal Pep...
DUF5077
DUF3472
PROKAR_LI...
SignalP-no...
248. Bacteroidetes___Draconibacterium_orientale___WP__038555604.1
Signal ...
Non cytoplasmic domain
Signal Pep...
DUF5077
DUF3472
SignalP-no...
SignalP-no...
SignalP-TM
PROKAR_LI...
249. Bacteroidetes___Salegentibacter_salegens___WP__079736183.1
Signal ...
Si...
Non cytoplasmic domain
Signal Peptide
DUF5077
DUF3472
SignalP-noTM
PROKAR_LIPO...
250. Bacteroidetes___Salegentibacter_echinorum___WP__072877425.1
Sig...
S..
Non cytoplasmic domain
Signal Pep...
DUF5077
DUF3472
SignalP-noTM
SignalP-no...
251. Bacteroidetes___Sunxiuqinia_dokdonensis___WP__053184784.1
Signal ...
Sig...
Non cytoplasmic domain
Signal Peptide
DUF5077
DUF3472
SignalP-TM
SignalP-noTM
252. Bacteroidetes___Marinilabilia_salmonicolor___WP__010662652.1
Signal ...
Non cytoplasmic domain
Signal Pep...
DUF5077
DUF3472
SignalP-no...
SignalP-no...
SignalP-TM
253. Bacteroidetes___Prolixibacteraceae_JC036___WP__137086575.1
Signal pe...
Non cytoplasmic domain
Signal Peptide
DUF5077
DUF3472
254. Bacteroidetes___Leadbetterella_byssophila___WP__013407286.1
DUF5077
DUF3472
255. Proteobacteria___Pseudoalteromonas_sp._C0348___WP__130165137.1
Sig...
S..
Non cytoplasmic domain
Signal Peptide
DUF5077
DUF3472
SignalP-noTM
SignalP-noTM
SignalP-TM
256. Proteobacteria___Pseudoalteromonas_piscicida___WP__099643041.1
Signal ...
S..
Non cytoplasmic domain
Signal Peptide
DUF5077
DUF3472
SignalP-noTM
SignalP-noTM
SignalP-TM
257. Proteobacteria___Pseudoalteromonas_sp._KS88___WP__135474548.1
Sig...
S.
S...
Non cytoplasmic domain
Signal Peptide
DUF5077
DUF3472
SignalP-noTM
SignalP-noTM
SignalP-TM
258. Proteobacteria___Pseudoalteromonas_porphyrae___WP__054205315.1
Signal ...
Si...
Si...
Non cytoplasmic domain
Signal Peptide
DUF5077
DUF3472
SignalP-noTM
SignalP-noTM
SignalP-TM
unknown
259. Proteobacteria___Pseudoalteromonas_sp._A601___WP__087531404.1
Sig...
Sig...
Non cytoplasmic domain
Signal Peptide
DUF5077
DUF3472
SignalP-noTM
SignalP-noTM
SignalP-TM
260. Proteobacteria___Pseudoalteromonas_prydzensis___WP__064666767.1
Sig...
Si...
Si...
Non cytoplasmic domain
Signal Peptide
DUF5077
DUF3472
SignalP-noTM
SignalP-noTM
SignalP-TM
261. Proteobacteria___Lacimicrobium_alkaliphilum___WP__099035254.1
Signal ...
S.
Non cytoplasmic domain
Signal Pepti...
DUF5077
DUF3472
SignalP-noTM
SignalP-noTM
SignalP-TM
262. Proteobacteria___Thalassotalea_sp._PP2-459___WP__083601902.1
Signal pe...
Non cytoplasmic domain
Signal Peptide
DUF5077
DUF3472
SignalP-TM
SignalP-noTM
PROKAR_LIPO...
263. Proteobacteria___Pseudoalteromonas_lipolytica___WP__054553655.1
Sig...
Si...
Non cytoplasmic domain
Signal Pep...
DUF5077
DUF3472
SignalP-no...
SignalP-no...
SignalP-TM
PROKAR_LI...

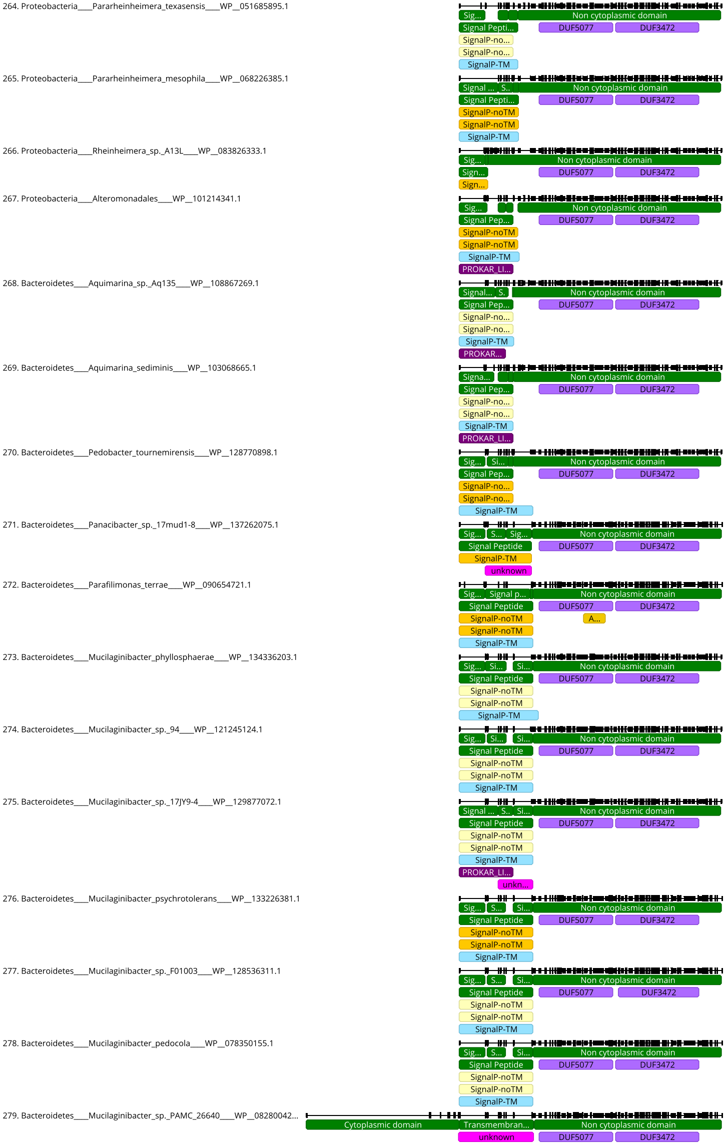
264. Proteobacteria___Pararheinheimera_texasensis___WP__051685895.1
Sig... Non cytoplasmic domain
Signal Pepti... DUF5077 DUF3472
SignalP-no...
SignalP-no...
SignalP-TM
265. Proteobacteria___Pararheinheimera_mesophila___WP__068226385.1
Signal ... S.. Non cytoplasmic domain
Signal Pepti... DUF5077 DUF3472
SignalP-noTM
SignalP-noTM
SignalP-TM
266. Proteobacteria___Rheinheimera_sp._A13L___WP__083826333.1
Sig... Non cytoplasmic domain
Sign... DUF5077 DUF3472
Sign...
267. Proteobacteria___Alteromonadales___WP__101214341.1
Sig... Non cytoplasmic domain
Signal Pep... DUF5077 DUF3472
SignalP-noTM
SignalP-noTM
SignalP-TM
PROKAR_LI...
268. Bacteroidetes___Aquimarina_sp._Aq135___WP__108867269.1
Signal... S. Non cytoplasmic domain
Signal Pep... DUF5077 DUF3472
SignalP-no...
SignalP-no...
SignalP-TM
PROKAR...
269. Bacteroidetes___Aquimarina_sediminis___WP__103068665.1
Signa... Non cytoplasmic domain
Signal Pep... DUF5077 DUF3472
SignalP-no...
SignalP-no...
SignalP-TM
PROKAR_LI...
270. Bacteroidetes___Pedobacter_tournemirensis___WP__128770898.1
Sig... Si... Non cytoplasmic domain
Signal Pep... DUF5077 DUF3472
SignalP-no...
SignalP-no...
SignalP-TM
271. Bacteroidetes___Panacibacter_sp._17mud1-8___WP__137262075.1
Sig... S... Sig... Non cytoplasmic domain
Signal Peptide DUF5077 DUF3472
SignalP-TM
unknown
272. Bacteroidetes___Parafilimonas_terrae___WP__090654721.1
Sig... Signal p... Non cytoplasmic domain
Signal Peptide DUF5077 DUF3472
SignalP-noTM A...
SignalP-noTM
SignalP-TM
273. Bacteroidetes___Mucilaginibacter_phyllosphaerae___WP__134336203.1
Sig... Si... Si... Non cytoplasmic domain
Signal Peptide DUF5077 DUF3472
SignalP-noTM
SignalP-noTM
SignalP-TM
274. Bacteroidetes___Mucilaginibacter_sp._94___WP__121245124.1
Sig... Si... Si... Non cytoplasmic domain
Signal Peptide DUF5077 DUF3472
SignalP-noTM
SignalP-noTM
SignalP-TM
275. Bacteroidetes___Mucilaginibacter_sp._17JY9-4___WP__129877072.1
Signal ... S.. Si... Non cytoplasmic domain
Signal Peptide DUF5077 DUF3472
SignalP-noTM
SignalP-noTM
SignalP-TM
PROKAR_LI...
unkn...
276. Bacteroidetes___Mucilaginibacter_psychrotolerans___WP__133226381.1
Sig... S... Si... Non cytoplasmic domain
Signal Peptide DUF5077 DUF3472
SignalP-noTM
SignalP-noTM
SignalP-TM
277. Bacteroidetes___Mucilaginibacter_sp._F01003___WP__128536311.1
Sig... S... Si... Non cytoplasmic domain
Signal Peptide DUF5077 DUF3472
SignalP-noTM
SignalP-noTM
SignalP-TM
278. Bacteroidetes___Mucilaginibacter_pedocola___WP__078350155.1
Sig... S... Si... Non cytoplasmic domain
Signal Peptide DUF5077 DUF3472
SignalP-noTM
SignalP-noTM
SignalP-TM
279. Bacteroidetes___Mucilaginibacter_sp._PAMC_26640___WP__08280042...
Cytoplasmic domain Transmembran... Non cytoplasmic domain
unknown DUF5077 DUF3472

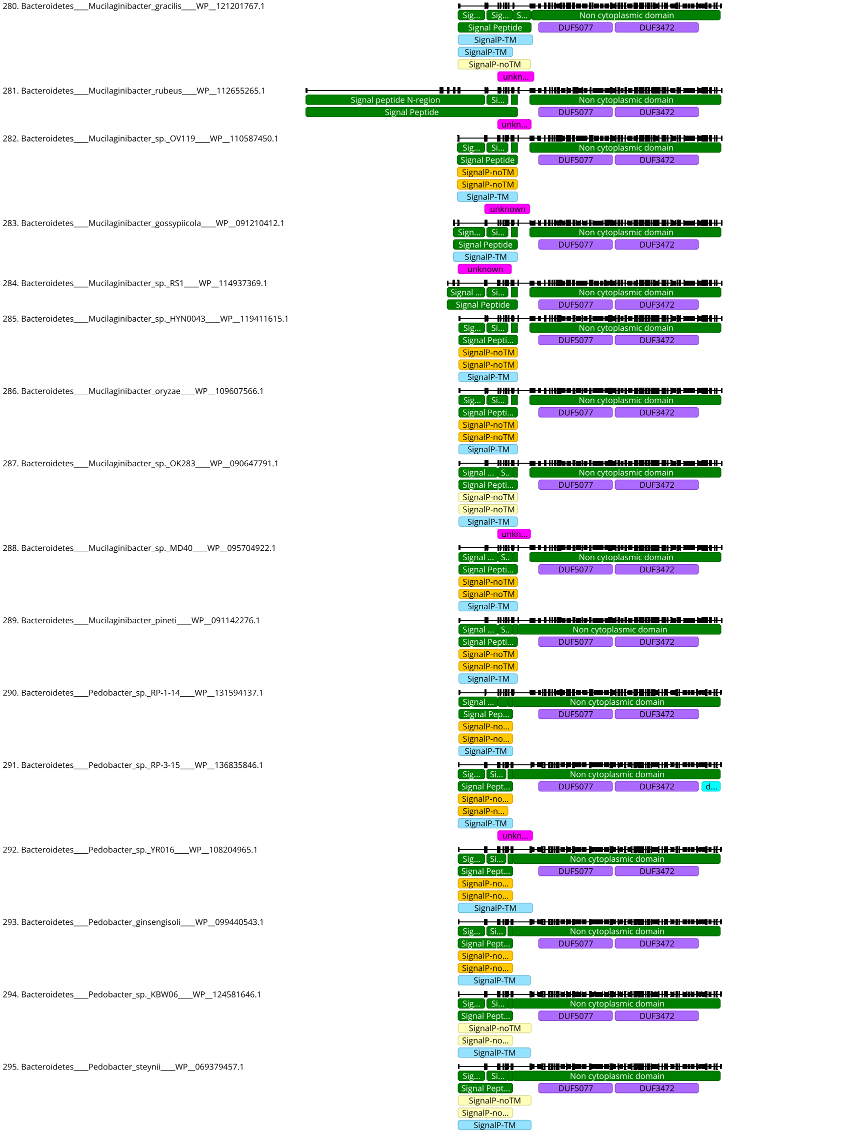
280. Bacteroidetes___Mucilaginibacter_gracilis___WP__121201767.1
Sig... Sig... S... Non cytoplasmic domain
Signal Peptide DUF5077 DUF3472
SignalP-TM
SignalP-TM
SignalP-noTM
unkn...
281. Bacteroidetes___Mucilaginibacter_rubeus___WP__112655265.1
Signal peptide N-region Si... Non cytoplasmic domain
Signal Peptide DUF5077 DUF3472
unkn...
282. Bacteroidetes___Mucilaginibacter_sp._OV119___WP__110587450.1
Sig... Si... Non cytoplasmic domain
Signal Peptide DUF5077 DUF3472
SignalP-noTM
SignalP-noTM
SignalP-TM
unknown
283. Bacteroidetes___Mucilaginibacter_gossypiicola___WP__091210412.1
Sign... Si... Non cytoplasmic domain
Signal Peptide DUF5077 DUF3472
SignalP-TM
unknown
284. Bacteroidetes___Mucilaginibacter_sp._RS1___WP__114937369.1
Signal ... Si... Non cytoplasmic domain
Signal Peptide DUF5077 DUF3472
285. Bacteroidetes___Mucilaginibacter_sp._HYN0043___WP__119411615.1
Sig... Si... Non cytoplasmic domain
Signal Pepti... DUF5077 DUF3472
SignalP-noTM
SignalP-noTM
SignalP-TM
286. Bacteroidetes___Mucilaginibacter_oryzae___WP__109607566.1
Sig... Si... Non cytoplasmic domain
Signal Pepti... DUF5077 DUF3472
SignalP-noTM
SignalP-noTM
SignalP-TM
287. Bacteroidetes___Mucilaginibacter_sp._OK283___WP__090647791.1
Signal ... S... Non cytoplasmic domain
Signal Pepti... DUF5077 DUF3472
SignalP-noTM
SignalP-noTM
SignalP-TM
unkn...
288. Bacteroidetes___Mucilaginibacter_sp._MD40___WP__095704922.1
Signal ... S... Non cytoplasmic domain
Signal Pepti... DUF5077 DUF3472
SignalP-noTM
SignalP-noTM
SignalP-TM
289. Bacteroidetes___Mucilaginibacter_pineti___WP__091142276.1
Signal ... S... Non cytoplasmic domain
Signal Pepti... DUF5077 DUF3472
SignalP-noTM
SignalP-noTM
SignalP-TM
290. Bacteroidetes___Pedobacter_sp._RP-1-14___WP__131594137.1
Signal ... Non cytoplasmic domain
Signal Pep... DUF5077 DUF3472
SignalP-no...
SignalP-no...
SignalP-TM
291. Bacteroidetes___Pedobacter_sp._RP-3-15___WP__136835846.1
Sig... Si... Non cytoplasmic domain
Signal Pept... DUF5077 DUF3472 d...
SignalP-no...
SignalP-n...
SignalP-TM
unkn...
292. Bacteroidetes___Pedobacter_sp._YR016___WP__108204965.1
Sig... Si... Non cytoplasmic domain
Signal Pept... DUF5077 DUF3472
SignalP-no...
SignalP-no...
SignalP-TM
293. Bacteroidetes___Pedobacter_ginsengisoli___WP__099440543.1
Sig... Si... Non cytoplasmic domain
Signal Pept... DUF5077 DUF3472
SignalP-no...
SignalP-no...
SignalP-TM
294. Bacteroidetes___Pedobacter_sp._KBW06___WP__124581646.1
Sig... Si... Non cytoplasmic domain
Signal Pept... DUF5077 DUF3472
SignalP-noTM
SignalP-no...
SignalP-TM
295. Bacteroidetes___Pedobacter_steynii___WP__069379457.1
Sig... Si... Non cytoplasmic domain
Signal Pept... DUF5077 DUF3472
SignalP-noTM
SignalP-no...
SignalP-TM

296. Bacteroidetes___Pedobacter_sp._PACM_27299___WP__062550573.1

297. Bacteroidetes___Pedobacter_nyackensis___WP__084288130.1

298. Bacteroidetes___Pedobacter_yulinensis___WP__107213935.1

299. Bacteroidetes___Mucilaginibacter_polytrichastri___WP__074489003.1

300. Bacteroidetes___Mucilaginibacter_sp._OK268___WP__090464273.1

301. Bacteroidetes___Mucilaginibacter_mallensis___WP__091370395.1

302. Bacteroidetes___Pedobacter_sp._OK701___WP__133779470.1

303. Bacteroidetes___Pedobacter_sp._HMWF019___WP__108552413.1

304. Bacteroidetes___Pedobacter_cryoconitis___WP__111631908.1

305. Bacteroidetes___Pedobacter_lusitanus___WP__041886098.1

306. Bacteroidetes___Solitalea_longa___WP__103789043.1

307. Bacteroidetes___Solitalea_canadensis___WP__014680973.1

308. Bacteroidetes___Chitinophaga___WP__111578057.1

309. Bacteroidetes___Chitinophaga_sp._MD30___WP__095838908.1

310. Bacteroidetes___Pedobacter_sp._KBW01___WP__124558601.1

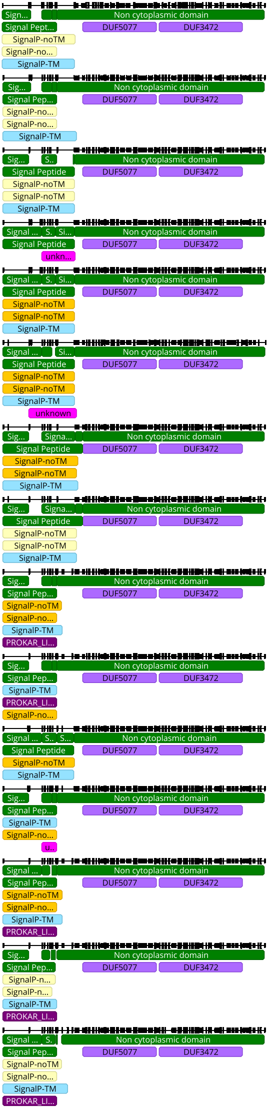

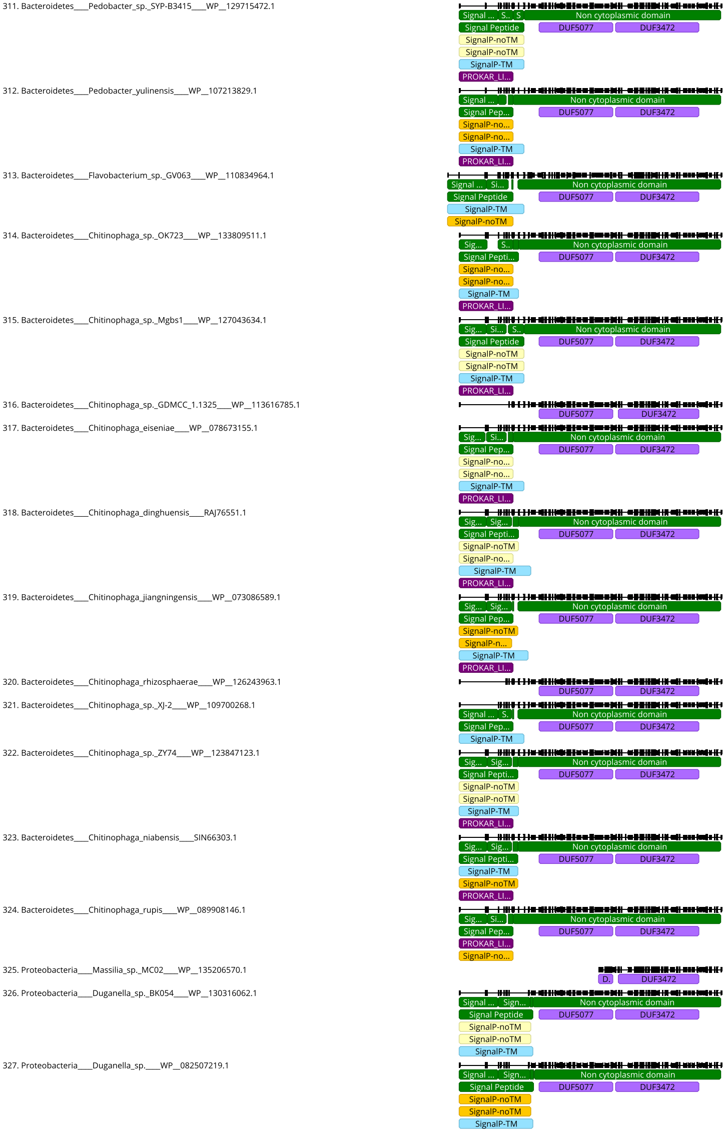

311. Bacteroidetes___Pedobacter_sp._SYP-B3415___WP__129715472.1
Signal ... S.. S Non cytoplasmic domain
Signal Peptide DUF5077 DUF3472
SignalP-noTM
SignalP-noTM
SignalP-TM
PROKAR_LI...
312. Bacteroidetes___Pedobacter_yulinensis___WP__107213829.1
Signal ... Non cytoplasmic domain
Signal Pep... DUF5077 DUF3472
SignalP-no...
SignalP-no...
SignalP-TM
PROKAR_LI...
313. Bacteroidetes___Flavobacterium_sp._GV063___WP__110834964.1
Signal ... Si... Non cytoplasmic domain
Signal Peptide DUF5077 DUF3472
SignalP-TM
SignalP-noTM
314. Bacteroidetes___Chitinophaga_sp._OK723___WP__133809511.1
Sig... S.. Non cytoplasmic domain
Signal Pepti... DUF5077 DUF3472
SignalP-no...
SignalP-no...
SignalP-TM
PROKAR_LI...
315. Bacteroidetes___Chitinophaga_sp._Mgbs1___WP__127043634.1
Sig... Si... S.. Non cytoplasmic domain
Signal Peptide DUF5077 DUF3472
SignalP-noTM
SignalP-noTM
SignalP-TM
PROKAR_LI...
316. Bacteroidetes___Chitinophaga_sp._GDMCC_1.1325___WP__113616785.1
DUF5077 DUF3472
317. Bacteroidetes___Chitinophaga_eiseniae___WP__078673155.1
Sig... Si... Non cytoplasmic domain
Signal Pep... DUF5077 DUF3472
SignalP-no...
SignalP-no...
SignalP-TM
PROKAR_LI...
318. Bacteroidetes___Chitinophaga_dinghuensis___RAJ76551.1
Sig... Sig... Non cytoplasmic domain
Signal Pepti... DUF5077 DUF3472
SignalP-noTM
SignalP-no...
SignalP-TM
PROKAR_LI...
319. Bacteroidetes___Chitinophaga_jiangningensis___WP__073086589.1
Sig... Sig... Non cytoplasmic domain
Signal Pep... DUF5077 DUF3472
SignalP-noTM
SignalP-n...
SignalP-TM
PROKAR_LI...
320. Bacteroidetes___Chitinophaga_rhizosphaerae___WP__126243963.1
DUF5077 DUF3472
321. Bacteroidetes___Chitinophaga_sp._XJ-2___WP__109700268.1
Signal ... S.. Non cytoplasmic domain
Signal Pep... DUF5077 DUF3472
SignalP-TM
322. Bacteroidetes___Chitinophaga_sp._ZY74___WP__123847123.1
Sig... Sig... Non cytoplasmic domain
Signal Pepti... DUF5077 DUF3472
SignalP-noTM
SignalP-noTM
SignalP-TM
PROKAR_LI...
323. Bacteroidetes___Chitinophaga_niabensis___SIN66303.1
Sig... Sig... Non cytoplasmic domain
Signal Pepti... DUF5077 DUF3472
SignalP-TM
SignalP-noTM
PROKAR_LI...
324. Bacteroidetes___Chitinophaga_rupis___WP__089908146.1
Sig... Si... Non cytoplasmic domain
Signal Pep... DUF5077 DUF3472
PROKAR_LI...
SignalP-no...
325. Proteobacteria___Massilia_sp._MC02___WP__135206570.1
D. DUF3472
326. Proteobacteria___Duganella_sp._BK054___WP__130316062.1
Signal ... Sign... Non cytoplasmic domain
Signal Peptide DUF5077 DUF3472
SignalP-noTM
SignalP-noTM
SignalP-TM
327. Proteobacteria___Duganella_sp.___WP__082507219.1
Signal ... Sign... Non cytoplasmic domain
Signal Peptide DUF5077 DUF3472
SignalP-noTM
SignalP-noTM
SignalP-TM

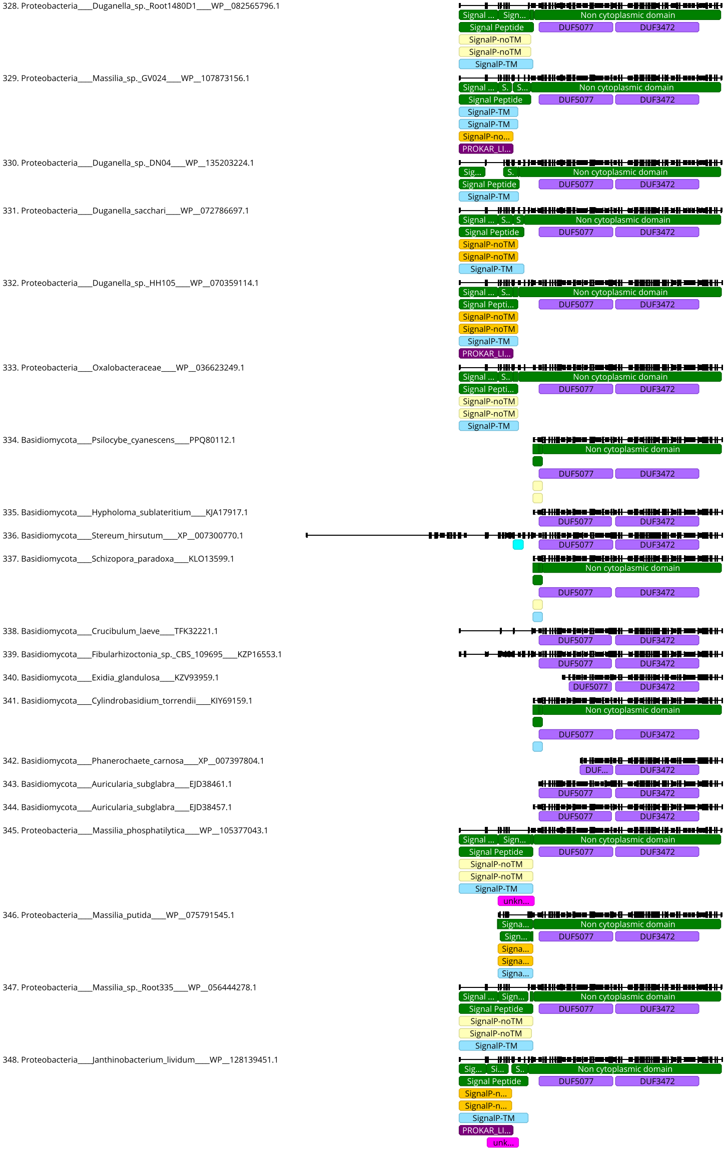
328. Proteobacteria____Duganella_sp._Root1480D1____WP__082565796.1
Signal ...
Sign...
Non cytoplasmic domain
Signal Peptide
DUF5077
DUF3472
SignalP-noTM
SignalP-noTM
SignalP-TM
329. Proteobacteria____Massilia_sp._GV024____WP__107873156.1
Signal ...
S.
S...
Non cytoplasmic domain
Signal Peptide
DUF5077
DUF3472
SignalP-TM
SignalP-TM
SignalP-no...
PROKAR_LI...
330. Proteobacteria____Duganella_sp._DN04____WP__135203224.1
Sig...
S.
Non cytoplasmic domain
Signal Peptide
DUF5077
DUF3472
SignalP-TM
331. Proteobacteria____Duganella_sacchari____WP__072786697.1
Signal ...
S..
S
Non cytoplasmic domain
Signal Peptide
DUF5077
DUF3472
SignalP-noTM
SignalP-noTM
SignalP-TM
332. Proteobacteria____Duganella_sp._HH105____WP__070359114.1
Signal ...
S..
Non cytoplasmic domain
Signal Pepti...
DUF5077
DUF3472
SignalP-noTM
SignalP-noTM
SignalP-TM
PROKAR_LI...
333. Proteobacteria____Oxalobacteraceae____WP__036623249.1
Signal ...
S..
Non cytoplasmic domain
Signal Pepti...
DUF5077
DUF3472
SignalP-noTM
SignalP-noTM
SignalP-TM
334. Basidiomycota____Psilocybe_cyanescens____PPQ80112.1
Non cytoplasmic domain
DUF5077
DUF3472
335. Basidiomycota____Hypholoma_sublateritium____KJA17917.1
DUF5077
DUF3472
336. Basidiomycota____Stereum_hirsutum____XP__007300770.1
DUF5077
DUF3472
337. Basidiomycota____Schizopora_paradoxa____KLO13599.1
Non cytoplasmic domain
DUF5077
DUF3472
338. Basidiomycota____Crucibulum_laeve____TFK32221.1
DUF5077
DUF3472
339. Basidiomycota____Fibularhizoctonia_sp._CBS_109695____KZP16553.1
DUF5077
DUF3472
340. Basidiomycota____Exidia_glandulosa____KZV93959.1
DUF5077
DUF3472
341. Basidiomycota____Cylindrobasidium_torrendii____KIY69159.1
Non cytoplasmic domain
DUF5077
DUF3472
342. Basidiomycota____Phanerochaete_carnosa____XP__007397804.1
DUF...
DUF3472
343. Basidiomycota____Auricularia_subglabra____EJD38461.1
DUF5077
DUF3472
344. Basidiomycota____Auricularia_subglabra____EJD38457.1
DUF5077
DUF3472
345. Proteobacteria____Massilia_phosphatilytica____WP__105377043.1
Signal ...
Sign...
Non cytoplasmic domain
Signal Peptide
DUF5077
DUF3472
SignalP-noTM
SignalP-noTM
SignalP-TM
unkn...
346. Proteobacteria____Massilia_putida____WP__075791545.1
Signa...
Non cytoplasmic domain
Sign...
DUF5077
DUF3472
Signa...
Signa...
Signa...
347. Proteobacteria____Massilia_sp._Root335____WP__056444278.1
Signal ...
Sign...
Non cytoplasmic domain
Signal Peptide
DUF5077
DUF3472
SignalP-noTM
SignalP-noTM
SignalP-TM
348. Proteobacteria____Janthinobacterium_lividum____WP__128139451.1
Sig...
Si...
S..
Non cytoplasmic domain
Signal Peptide
DUF5077
DUF3472
SignalP-n...
SignalP-n...
SignalP-TM
PROKAR_LI...
unk...

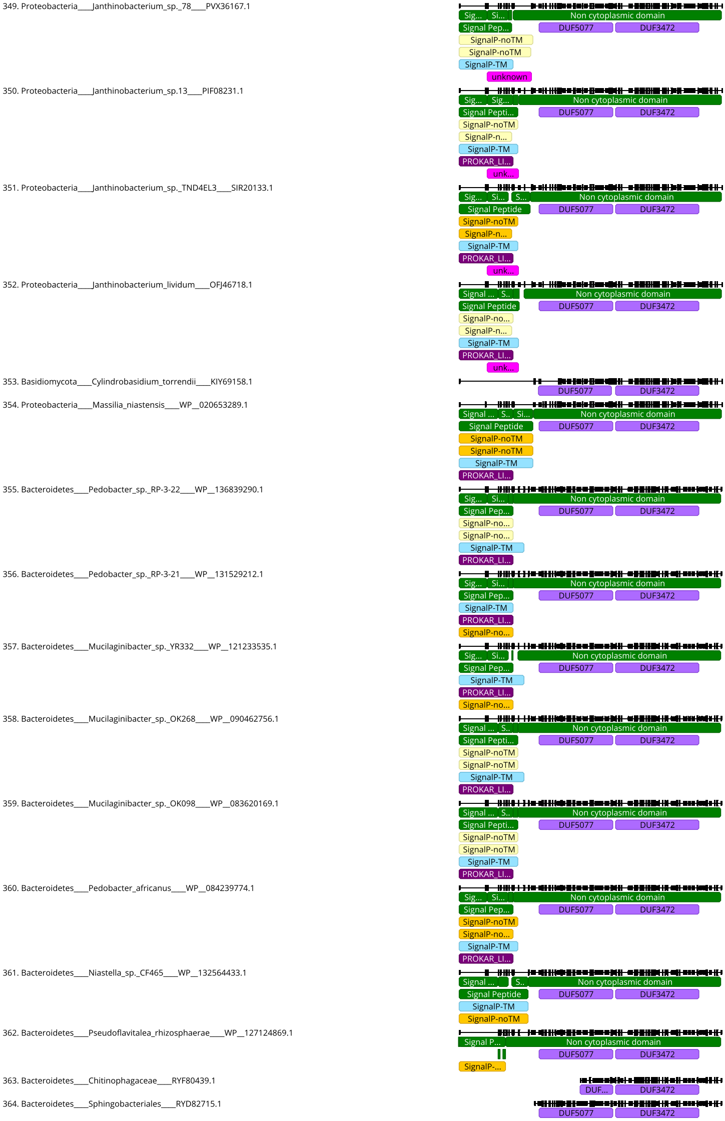
349. Proteobacteria____Janthinobacterium_sp._78____PVX36167.1
Sig... Si... Non cytoplasmic domain
Signal Pep... DUF5077 DUF3472
SignalP-noTM
SignalP-noTM
SignalP-TM
unknown
350. Proteobacteria____Janthinobacterium_sp.13____PIF08231.1
Sig... Sig... Non cytoplasmic domain
Signal Pepti... DUF5077 DUF3472
SignalP-noTM
SignalP-n...
SignalP-TM
PROKAR_LI...
unk...
351. Proteobacteria____Janthinobacterium_sp._TND4EL3____SIR20133.1
Sig... Si... S... Non cytoplasmic domain
Signal Peptide DUF5077 DUF3472
SignalP-noTM
SignalP-n...
SignalP-TM
PROKAR_LI...
unk...
352. Proteobacteria____Janthinobacterium_lividum____OFJ46718.1
Signal ... S.. Non cytoplasmic domain
Signal Peptide DUF5077 DUF3472
SignalP-no...
SignalP-n...
SignalP-TM
PROKAR_LI...
unk...
353. Basidiomycota____Cylindrobasidium_torrendii____KIY69158.1
DUF5077 DUF3472
354. Proteobacteria____Massilia_niastensis____WP__020653289.1
Signal ... S.. Si... Non cytoplasmic domain
Signal Peptide DUF5077 DUF3472
SignalP-noTM
SignalP-noTM
SignalP-TM
PROKAR_LI...
355. Bacteroidetes____Pedobacter_sp._RP-3-22____WP__136839290.1
Sig... Si... Non cytoplasmic domain
Signal Pep... DUF5077 DUF3472
SignalP-no...
SignalP-no...
SignalP-TM
PROKAR_LI...
356. Bacteroidetes____Pedobacter_sp._RP-3-21____WP__131529212.1
Sig... Si... Non cytoplasmic domain
Signal Pep... DUF5077 DUF3472
SignalP-TM
PROKAR_LI...
SignalP-no...
357. Bacteroidetes____Mucilaginibacter_sp._YR332____WP__121233535.1
Sig... Si... Non cytoplasmic domain
Signal Pep... DUF5077 DUF3472
SignalP-TM
PROKAR_LI...
SignalP-no...
358. Bacteroidetes____Mucilaginibacter_sp._OK268____WP__090462756.1
Signal ... S.. Non cytoplasmic domain
Signal Pepti... DUF5077 DUF3472
SignalP-noTM
SignalP-noTM
SignalP-TM
PROKAR_LI...
359. Bacteroidetes____Mucilaginibacter_sp._OK098____WP__083620169.1
Signal ... S.. Non cytoplasmic domain
Signal Pepti... DUF5077 DUF3472
SignalP-noTM
SignalP-noTM
SignalP-TM
PROKAR_LI...
360. Bacteroidetes____Pedobacter_africanus____WP__084239774.1
Sig... Si... Non cytoplasmic domain
Signal Pep... DUF5077 DUF3472
SignalP-noTM
SignalP-no...
SignalP-TM
PROKAR_LI...
361. Bacteroidetes____Niastella_sp._CF465____WP__132564433.1
Signal ... S.. Non cytoplasmic domain
Signal Peptide DUF5077 DUF3472
SignalP-TM
SignalP-noTM
362. Bacteroidetes____Pseudoflavitalea_rhizosphaerae____WP__127124869.1
Signal P... Non cytoplasmic domain
DUF5077 DUF3472
SignalP-...
363. Bacteroidetes____Chitinophagaceae____RYF80439.1
DUF... DUF3472
364. Bacteroidetes____Sphingobacteriales____RYD82715.1
DUF5077 DUF3472

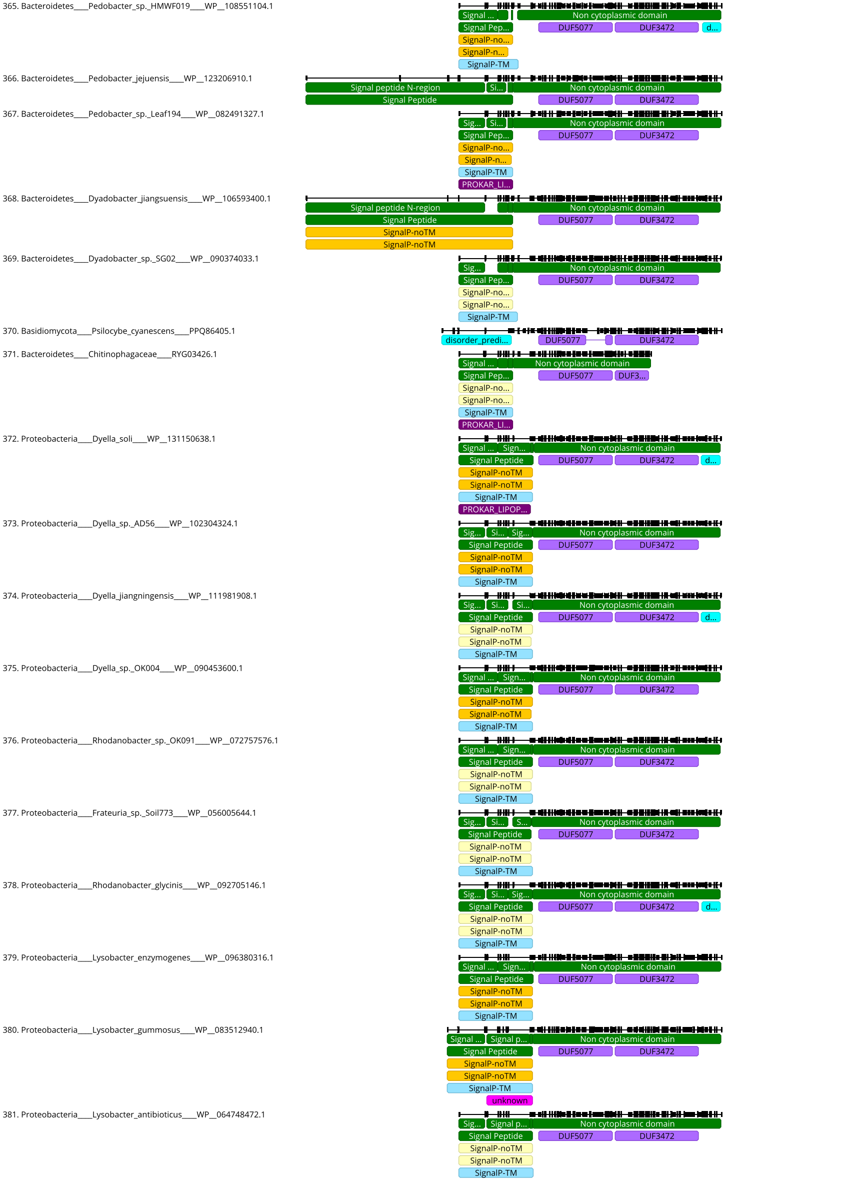

365. Bacteroidetes___Pedobacter_sp._HMWF019___WP__108551104.1
Signal ...
Non cytoplasmic domain
Signal Pep...
DUF5077
DUF3472
d...
SignalP-no...
SignalP-n...
SignalP-TM
366. Bacteroidetes___Pedobacter_jejuensis___WP__123206910.1
Signal peptide N-region
Si...
Non cytoplasmic domain
Signal Peptide
DUF5077
DUF3472
367. Bacteroidetes___Pedobacter_sp._Leaf194___WP__082491327.1
Sig...
Si...
Non cytoplasmic domain
Signal Pep...
DUF5077
DUF3472
SignalP-no...
SignalP-n...
SignalP-TM
PROKAR_LI...
368. Bacteroidetes___Dyadobacter_jiangsuensis___WP__106593400.1
Signal peptide N-region
Non cytoplasmic domain
Signal Peptide
DUF5077
DUF3472
SignalP-noTM
SignalP-noTM
369. Bacteroidetes___Dyadobacter_sp._SG02___WP__090374033.1
Sig...
Non cytoplasmic domain
Signal Pep...
DUF5077
DUF3472
SignalP-no...
SignalP-no...
SignalP-TM
370. Basidiomycota___Psilocybe_cyanescens___PPQ86405.1
disorder_predi...
DUF5077
DUF3472
371. Bacteroidetes___Chitinophagaceae___RYG03426.1
Signal ...
Non cytoplasmic domain
Signal Pep...
DUF5077
DUF3...
SignalP-no...
SignalP-no...
SignalP-TM
PROKAR_LI...
372. Proteobacteria___Dyella_soli___WP__131150638.1
Signal ...
Sign...
Non cytoplasmic domain
Signal Peptide
DUF5077
DUF3472
d...
SignalP-noTM
SignalP-noTM
SignalP-TM
PROKAR_LIPOP...
373. Proteobacteria___Dyella_sp._AD56___WP__102304324.1
Sig...
Si...
Sig...
Non cytoplasmic domain
Signal Peptide
DUF5077
DUF3472
SignalP-noTM
SignalP-noTM
SignalP-TM
374. Proteobacteria___Dyella_jiangningensis___WP__111981908.1
Sig...
Si...
Si...
Non cytoplasmic domain
Signal Peptide
DUF5077
DUF3472
d...
SignalP-noTM
SignalP-noTM
SignalP-TM
375. Proteobacteria___Dyella_sp._OK004___WP__090453600.1
Signal ...
Sign...
Non cytoplasmic domain
Signal Peptide
DUF5077
DUF3472
SignalP-noTM
SignalP-noTM
SignalP-TM
376. Proteobacteria___Rhodanobacter_sp._OK091___WP__072757576.1
Signal ...
Sign...
Non cytoplasmic domain
Signal Peptide
DUF5077
DUF3472
SignalP-noTM
SignalP-noTM
SignalP-TM
377. Proteobacteria___Frateuria_sp._Soil773___WP__056005644.1
Sig...
Si...
S...
Non cytoplasmic domain
Signal Peptide
DUF5077
DUF3472
SignalP-noTM
SignalP-noTM
SignalP-TM
378. Proteobacteria___Rhodanobacter_glycinis___WP__092705146.1
Sig...
Si...
Sig...
Non cytoplasmic domain
Signal Peptide
DUF5077
DUF3472
d...
SignalP-noTM
SignalP-noTM
SignalP-TM
379. Proteobacteria___Lysobacter_enzymogenes___WP__096380316.1
Signal ...
Sign...
Non cytoplasmic domain
Signal Peptide
DUF5077
DUF3472
SignalP-noTM
SignalP-noTM
SignalP-TM
380. Proteobacteria___Lysobacter_gummosus___WP__083512940.1
Signal ...
Signal p...
Non cytoplasmic domain
Signal Peptide
DUF5077
DUF3472
SignalP-noTM
SignalP-noTM
SignalP-TM
unknown
381. Proteobacteria___Lysobacter_antibioticus___WP__064748472.1
Sig...
Signal p...
Non cytoplasmic domain
Signal Peptide
DUF5077
DUF3472
SignalP-noTM
SignalP-noTM
SignalP-TM

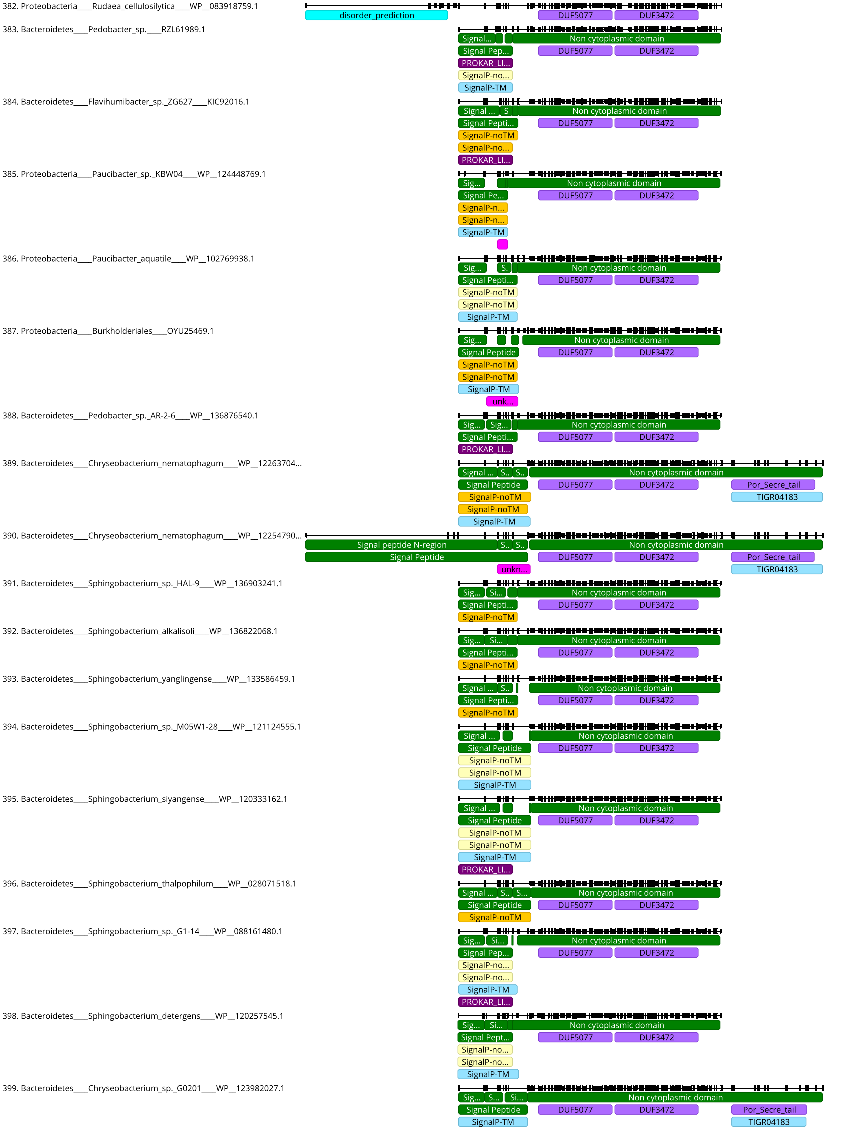
382. Proteobacteria____Rudaea_cellulosilytica____WP__083918759.1
disorder_prediction
DUF5077
DUF3472
383. Bacteroidetes____Pedobacter_sp.____RZL61989.1
Signal...
Non cytoplasmic domain
Signal Pep...
DUF5077
DUF3472
PROKAR_LI...
SignalP-no...
SignalP-TM
384. Bacteroidetes____Flavihumibacter_sp._ZG627____KIC92016.1
Signal ...
S
Non cytoplasmic domain
Signal Pepti...
DUF5077
DUF3472
SignalP-noTM
SignalP-no...
PROKAR_LI...
385. Proteobacteria____Paucibacter_sp._KBW04____WP__124448769.1
Sig...
Non cytoplasmic domain
Signal Pe...
DUF5077
DUF3472
SignalP-n...
SignalP-n...
SignalP-TM
386. Proteobacteria____Paucibacter_aquatile____WP__102769938.1
Sig...
S.
Non cytoplasmic domain
Signal Pepti...
DUF5077
DUF3472
SignalP-noTM
SignalP-noTM
SignalP-TM
387. Proteobacteria____Burkholderiales____OYU25469.1
Sig...
Non cytoplasmic domain
Signal Peptide
DUF5077
DUF3472
SignalP-noTM
SignalP-noTM
SignalP-TM
unk...
388. Bacteroidetes____Pedobacter_sp._AR-2-6____WP__136876540.1
Sig...
Sig...
Non cytoplasmic domain
Signal Pepti...
DUF5077
DUF3472
PROKAR_LI...
389. Bacteroidetes____Chryseobacterium_nematophagum____WP__12263704...
Signal ...
S..
S..
Non cytoplasmic domain
Signal Peptide
DUF5077
DUF3472
Por_Secre_tail
SignalP-noTM
TIGR04183
SignalP-noTM
SignalP-TM
390. Bacteroidetes____Chryseobacterium_nematophagum____WP__12254790...
Signal peptide N-region
S..
S..
Non cytoplasmic domain
Signal Peptide
DUF5077
DUF3472
Por_Secre_tail
unkn...
TIGR04183
391. Bacteroidetes____Sphingobacterium_sp._HAL-9____WP__136903241.1
Sig...
Si...
Non cytoplasmic domain
Signal Pepti...
DUF5077
DUF3472
SignalP-noTM
392. Bacteroidetes____Sphingobacterium_alkalisoli____WP__136822068.1
Sig...
Si...
Non cytoplasmic domain
Signal Pepti...
DUF5077
DUF3472
SignalP-noTM
393. Bacteroidetes____Sphingobacterium_yanglingense____WP__133586459.1
Signal ...
S..
Non cytoplasmic domain
Signal Pepti...
DUF5077
DUF3472
SignalP-noTM
394. Bacteroidetes____Sphingobacterium_sp._M05W1-28____WP__121124555.1
Signal ...
Non cytoplasmic domain
Signal Peptide
DUF5077
DUF3472
SignalP-noTM
SignalP-noTM
SignalP-TM
395. Bacteroidetes____Sphingobacterium_siyangense____WP__120333162.1
Signal ...
Non cytoplasmic domain
Signal Peptide
DUF5077
DUF3472
SignalP-noTM
SignalP-noTM
SignalP-TM
PROKAR_LI...
396. Bacteroidetes____Sphingobacterium_thalpophilum____WP__028071518.1
Signal ...
S..
S...
Non cytoplasmic domain
Signal Peptide
DUF5077
DUF3472
SignalP-noTM
397. Bacteroidetes____Sphingobacterium_sp._G1-14____WP__088161480.1
Sig...
Si...
Non cytoplasmic domain
Signal Pep...
DUF5077
DUF3472
SignalP-no...
SignalP-no...
SignalP-TM
PROKAR_LI...
398. Bacteroidetes____Sphingobacterium_detergens____WP__120257545.1
Sig...
Si...
Non cytoplasmic domain
Signal Pept...
DUF5077
DUF3472
SignalP-no...
SignalP-no...
SignalP-TM
399. Bacteroidetes____Chryseobacterium_sp._G0201____WP__123982027.1
Sig...
S...
Si...
Non cytoplasmic domain
Signal Peptide
DUF5077
DUF3472
Por_Secre_tail
SignalP-TM
TIGR04183

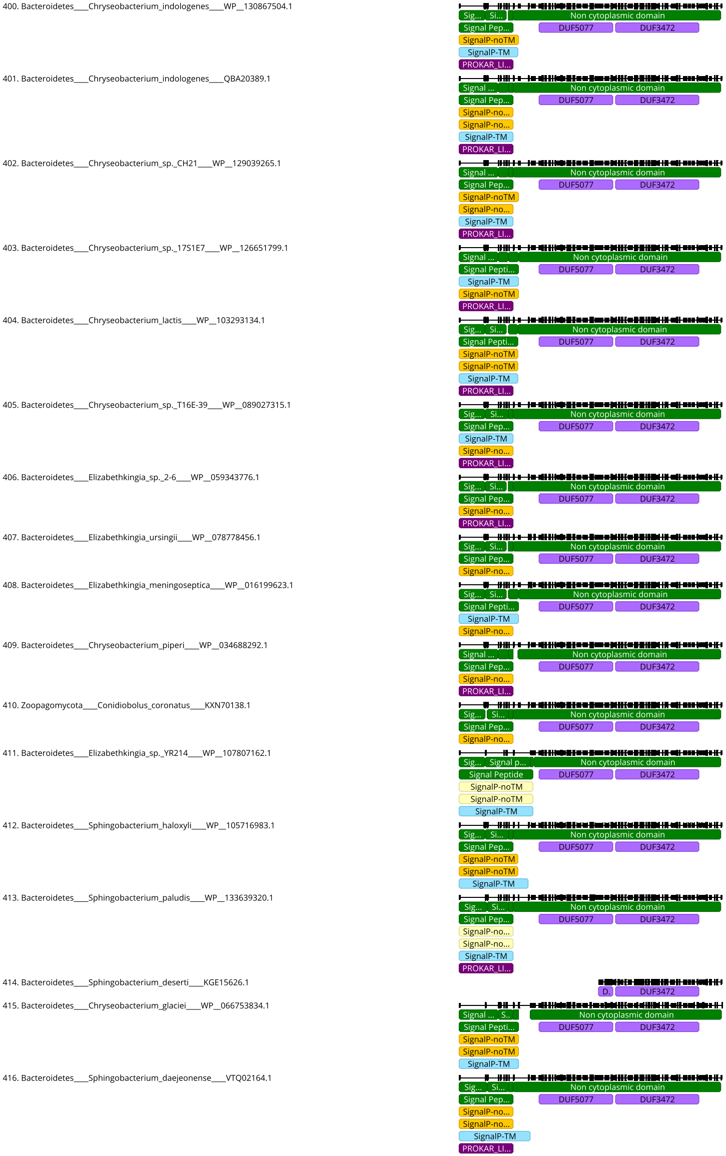
400. Bacteroidetes___Chryseobacterium_indologenes___WP__130867504.1
Sig... Si... Non cytoplasmic domain
Signal Pep... DUF5077 DUF3472
SignalP-noTM
SignalP-TM
PROKAR_LI...
401. Bacteroidetes___Chryseobacterium_indologenes___QBA20389.1
Signal ... Non cytoplasmic domain
Signal Pep... DUF5077 DUF3472
SignalP-no...
SignalP-no...
SignalP-TM
PROKAR_LI...
402. Bacteroidetes___Chryseobacterium_sp._CH21___WP__129039265.1
Signal ... Non cytoplasmic domain
Signal Pep... DUF5077 DUF3472
SignalP-noTM
SignalP-no...
SignalP-TM
PROKAR_LI...
403. Bacteroidetes___Chryseobacterium_sp._17S1E7___WP__126651799.1
Signal ... Non cytoplasmic domain
Signal Pepti... DUF5077 DUF3472
SignalP-TM
SignalP-noTM
PROKAR_LI...
404. Bacteroidetes___Chryseobacterium_lactis___WP__103293134.1
Sig... Si... Non cytoplasmic domain
Signal Pepti... DUF5077 DUF3472
SignalP-noTM
SignalP-noTM
SignalP-TM
PROKAR_LI...
405. Bacteroidetes___Chryseobacterium_sp._T16E-39___WP__089027315.1
Sig... Si... Non cytoplasmic domain
Signal Pep... DUF5077 DUF3472
SignalP-TM
SignalP-no...
PROKAR_LI...
406. Bacteroidetes___Elizabethkingia_sp._2-6___WP__059343776.1
Sig... Si... Non cytoplasmic domain
Signal Pep... DUF5077 DUF3472
SignalP-no...
PROKAR_LI...
407. Bacteroidetes___Elizabethkingia_ursingii___WP__078778456.1
Sig... Si... Non cytoplasmic domain
Signal Pep... DUF5077 DUF3472
SignalP-no...
408. Bacteroidetes___Elizabethkingia_meningoseptica___WP__016199623.1
Sig... Si... Non cytoplasmic domain
Signal Pepti... DUF5077 DUF3472
SignalP-TM
SignalP-no...
409. Bacteroidetes___Chryseobacterium_piperi___WP__034688292.1
Signal ... Non cytoplasmic domain
Signal Pep... DUF5077 DUF3472
SignalP-no...
PROKAR_LI...
410. Zoopagomycota___Conidiobolus_coronatus___KXN70138.1
Sig... Si... Non cytoplasmic domain
Signal Pep... DUF5077 DUF3472
SignalP-no...
411. Bacteroidetes___Elizabethkingia_sp._YR214___WP__107807162.1
Sig... Signal p... Non cytoplasmic domain
Signal Peptide DUF5077 DUF3472
SignalP-noTM
SignalP-noTM
SignalP-TM
412. Bacteroidetes___Sphingobacterium_haloxyli___WP__105716983.1
Sig... Si... Non cytoplasmic domain
Signal Pep... DUF5077 DUF3472
SignalP-noTM
SignalP-noTM
SignalP-TM
413. Bacteroidetes___Sphingobacterium_paludis___WP__133639320.1
Sig... Si... Non cytoplasmic domain
Signal Pep... DUF5077 DUF3472
SignalP-no...
SignalP-no...
SignalP-TM
PROKAR_LI...
414. Bacteroidetes___Sphingobacterium_deserti___KGE15626.1
D. DUF3472
415. Bacteroidetes___Chryseobacterium_glaciei___WP__066753834.1
Signal ... S.. Non cytoplasmic domain
Signal Pepti... DUF5077 DUF3472
SignalP-noTM
SignalP-noTM
SignalP-TM
416. Bacteroidetes___Sphingobacterium_daejeonense___VTQ02164.1
Sig... Si... Non cytoplasmic domain
Signal Pep... DUF5077 DUF3472
SignalP-no...
SignalP-no...
SignalP-TM
PROKAR_LI...

417. Bacteroidetes___Sphingobacterium_sp._JB170___WP__086949048.1

418. Bacteroidetes___Sphingobacterium_cellulitidis___WP__094255906.1

419. Bacteroidetes___Sphingobacterium_lactis___WP__103907392.1

420. Bacteroidetes___Chitinophaga_caeni___WP__098194367.1

421. Bacteroidetes___Sphingobacterium_sp._CZ-2___WP__134777587.1

422. Bacteroidetes___Chryseobacterium_sp._YR477___WP__052186721.1

423. Bacteroidetes___Chryseobacterium_sp._KLBC_52___WP__114819687.1

424. Bacteroidetes___Chryseobacterium_sp._RU37D___SIQ54442.1

425. Bacteroidetes___Sphingobacterium_alimentarium___WP__132778043.1

426. Bacteroidetes___Empedobacter_falsenii___WP__125350328.1

427. Bacteroidetes___Sphingobacterium_sp._T2___WP__052258805.1

428. Bacteroidetes___Sphingobacterium_nematocida___WP__079644953.1

429. Copepoda___Eucyclops_serrulatus___TR106736__c0__g2__i3

430. Copepoda___Eucyclops_serrulatus___TR107317__c0__g1__i2

431. Hydrozoa___Hydra_vulgaris___XP__002157558.3

432. Bacteroidetes___Cytophaga_sp._DMA-K-7a___WP__132115003.1

433. Bacteroidetes___Dyadobacter_sp._AR-3-8___TKT93938.1

434. Bacteroidetes___Dyadobacter_sp._AR-3-6___WP__131959551.1

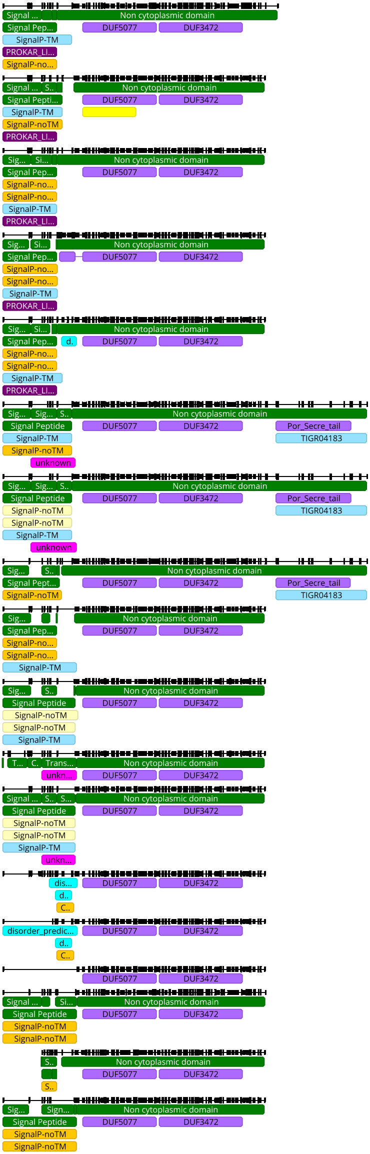

435. Bacteroidetes___Dyadobacter_tibetensis___WP__025762782.1
436. Bacteroidetes___Dyadobacter_jejuensis___WP__109672101.1
437. Bacteroidetes___Pseudarcicella_hirudinis___WP__092018071.1
438. Bacteroidetes___Arsenicibacter_rosenii___WP__071502151.1
439. Bacteroidetes___Runella_slithyformis___AEI49798.1
440. Bacteroidetes___Runella_sp._YX9___WP__114460489.1
441. Bacteroidetes___Runella_sp._2___WP__122932041.1
442. Bacteroidetes___Emticicia_sp._MM___WP__064196456.1
443. Bacteroidetes___Cytophagales_sp.___TAH18264.1
444. Bacteroidetes___Larkinella_sp._ZZJ9___WP__114408658.1
445. Bacteroidetes___Larkinella_sp._BK230___WP__134024719.1
446. Bacteroidetes___Larkinella_arboricola___WP__111628891.1
447. Bacteroidetes___Larkinella_rosea___WP__124873807.1
448. Bacteroidetes___Larkinella_knui___WP__124909598.1
449. Bacteroidetes___Spirosoma_oryzae___WP__106136702.1
450. Bacteroidetes___Fibrella_sp._ES10-3-2-2___WP__085414209.1
451. Bacteroidetes___Spirosoma_rigui___WP__080241671.1

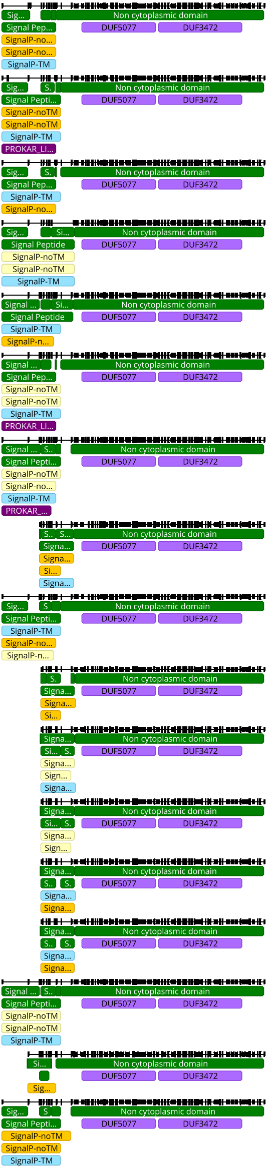

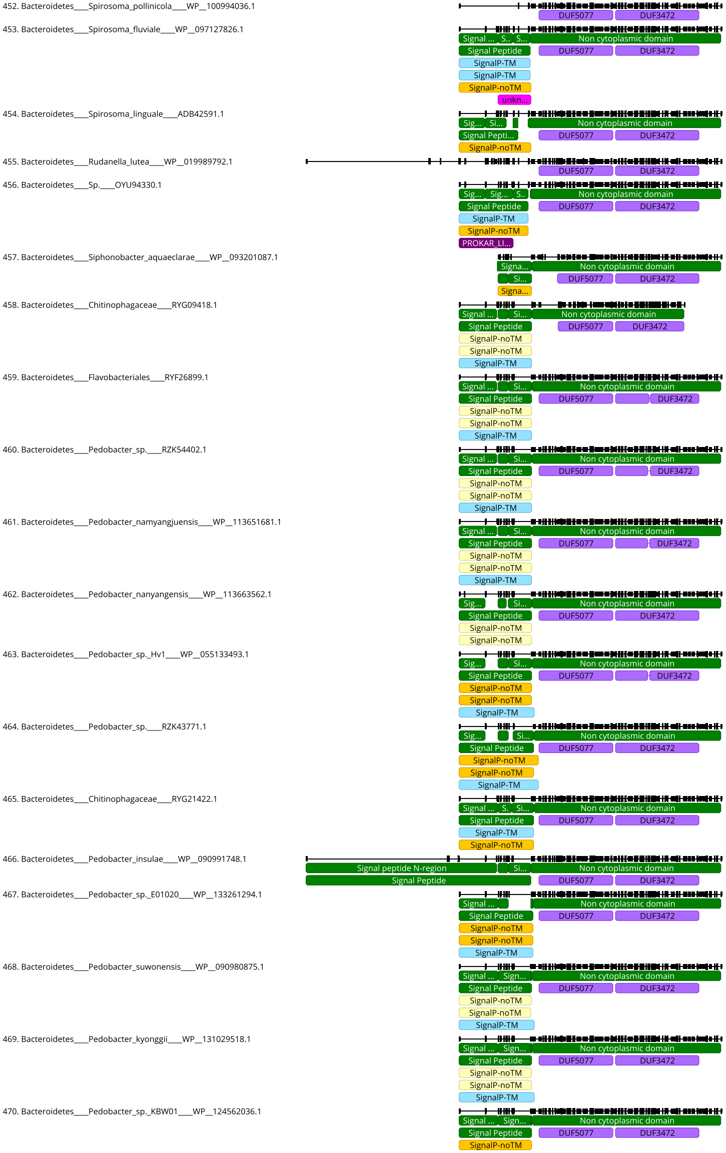

452. Bacteroidetes___Spirosoma_pollinicola___WP__100994036.1
DUF5077
DUF3472
453. Bacteroidetes___Spirosoma_fluviale___WP__097127826.1
Signal ...
S..
S...
Non cytoplasmic domain
Signal Peptide
DUF5077
DUF3472
SignalP-TM
SignalP-TM
SignalP-noTM
unkn...
454. Bacteroidetes___Spirosoma_linguale___ADB42591.1
Sig...
Si...
Non cytoplasmic domain
Signal Pepti...
DUF5077
DUF3472
SignalP-noTM
455. Bacteroidetes___Rudanella_lutea___WP__019989792.1
DUF5077
DUF3472
456. Bacteroidetes___Sp.___OYU94330.1
Sig...
Sig...
S..
Non cytoplasmic domain
Signal Peptide
DUF5077
DUF3472
SignalP-TM
SignalP-noTM
PROKAR_LI...
457. Bacteroidetes___Siphonobacter_aquaeclarae___WP__093201087.1
Signa...
Non cytoplasmic domain
Si...
DUF5077
DUF3472
Signa...
458. Bacteroidetes___Chitinophagaceae___RYG09418.1
Signal ...
Si...
Non cytoplasmic domain
Signal Peptide
DUF5077
DUF3472
SignalP-noTM
SignalP-noTM
SignalP-TM
459. Bacteroidetes___Flavobacteriales___RYF26899.1
Signal ...
Si...
Non cytoplasmic domain
Signal Peptide
DUF5077
DUF3472
SignalP-noTM
SignalP-noTM
SignalP-TM
460. Bacteroidetes___Pedobacter_sp.___RZK54402.1
Signal ...
Si...
Non cytoplasmic domain
Signal Peptide
DUF5077
DUF3472
SignalP-noTM
SignalP-noTM
SignalP-TM
461. Bacteroidetes___Pedobacter_namyangjuensis___WP__113651681.1
Signal ...
Si...
Non cytoplasmic domain
Signal Peptide
DUF5077
DUF3472
SignalP-noTM
SignalP-noTM
SignalP-TM
462. Bacteroidetes___Pedobacter_nanyangensis___WP__113663562.1
Sig...
Si...
Non cytoplasmic domain
Signal Peptide
DUF5077
DUF3472
SignalP-noTM
SignalP-noTM
463. Bacteroidetes___Pedobacter_sp._Hv1___WP__055133493.1
Sig...
Si...
Non cytoplasmic domain
Signal Peptide
DUF5077
DUF3472
SignalP-noTM
SignalP-noTM
SignalP-TM
464. Bacteroidetes___Pedobacter_sp.___RZK43771.1
Sig...
Si...
Non cytoplasmic domain
Signal Peptide
DUF5077
DUF3472
SignalP-noTM
SignalP-noTM
SignalP-TM
465. Bacteroidetes___Chitinophagaceae___RYG21422.1
Signal ...
S.
Si...
Non cytoplasmic domain
Signal Peptide
DUF5077
DUF3472
SignalP-TM
SignalP-noTM
466. Bacteroidetes___Pedobacter_insulae___WP__090991748.1
Signal peptide N-region
Si...
Non cytoplasmic domain
Signal Peptide
DUF5077
DUF3472
467. Bacteroidetes___Pedobacter_sp._E01020___WP__133261294.1
Signal ...
Non cytoplasmic domain
Signal Peptide
DUF5077
DUF3472
SignalP-noTM
SignalP-noTM
SignalP-TM
468. Bacteroidetes___Pedobacter_suwonensis___WP__090980875.1
Signal ...
Sign...
Non cytoplasmic domain
Signal Peptide
DUF5077
DUF3472
SignalP-noTM
SignalP-noTM
SignalP-TM
469. Bacteroidetes___Pedobacter_kyonggii___WP__131029518.1
Signal ...
Sign...
Non cytoplasmic domain
Signal Peptide
DUF5077
DUF3472
SignalP-noTM
SignalP-noTM
SignalP-TM
470. Bacteroidetes___Pedobacter_sp._KBW01___WP__124562036.1
Signal ...
Sign...
Non cytoplasmic domain
Signal Peptide
DUF5077
DUF3472
SignalP-noTM

471. Bacteroidetes____Pedobacter_sp._Leaf216____WP__055903036.1

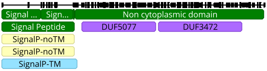

472. Bacteroidetes____Pedobacter_sp._JDX94____WP__115402240.1

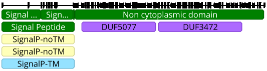

473. Bacteroidetes____Pedobacter_sp._R-8____WP__109929126.1

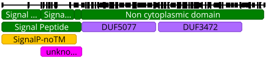

474. Bacteroidetes____Pedobacter_rhizosphaerae____WP__090889365.1

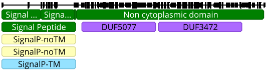

475. Bacteroidetes____Flavobacterium_sp._LB3P52____WP__131917199.1

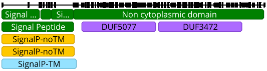

476. Bacteroidetes____Flavobacterium_sp._103____WP__116798004.1

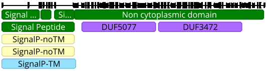

477. Bacteroidetes____Flavobacterium_fluvii____WP__073370380.1

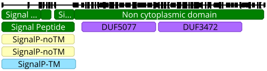

478. Bacteroidetes____Flavobacterium_cutihirudinis____WP__115887313.1

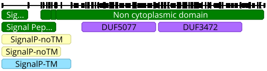

479. Bacteroidetes____Flavobacterium_sp._A5.7____WP__132990066.1

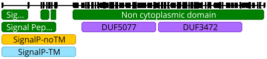

480. Bacteroidetes____Flavobacterium_nitrogenifigens____WP__111378677.1

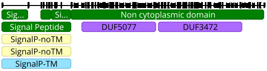

481. Bacteroidetes____Flavobacterium_sp._URHB0058____WP__026981994.1

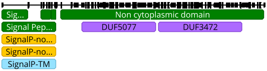

482. Bacteroidetes____Flavobacterium_sp._Root420____WP__056186260.1

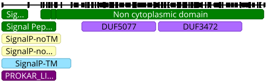

483. Bacteroidetes____Flavobacterium_sp._90____WP__121325946.1

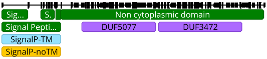

484. Bacteroidetes____Flavobacterium_sp._GV063____WP__110837240.1

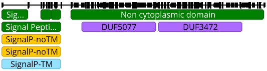

485. Bacteroidetes____Flavobacterium_hibernum____WP__041519326.1

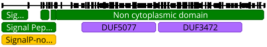

486. Bacteroidetes____Flavobacterium_sp._245____WP__133525014.1

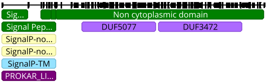

487. Bacteroidetes___Flavobacterium_sp._JRR20__7___WP__121318803.1

488. Bacteroidetes___Flavobacterium_sp._YO64___WP__129053625.1

489. Bacteroidetes___Flavobacterium_saccharophilum___SHL91891.1

490. Bacteroidetes___Flavobacterium_sp._HYN0086___WP__113678084.1

491. Bacteroidetes___Flavobacterium_crocinum___WP__109192565.1

492. Bacteroidetes___Flavobacterium_denitrificans___WP__026729131.1

493. Bacteroidetes___Flavobacterium_sp._WG21___WP__017497324.1

494. Bacteroidetes___Flavobacterium_sp._ABG___WP__047775717.1

495. Bacteroidetes___Flavobacterium_araucananum___WP__089481373.1

496. Bacteroidetes___Flavobacterium_weaverense___WP__121925968.1

497. Bacteroidetes___Flavobacterium_sp._TSA-D2___WP__132112489.1

498. Bacteroidetes___Flavobacterium_granuli___WP__072942011.1

499. Bacteroidetes___Flavobacterium_gillisiae___SEA92715.1

500. Bacteroidetes___Flavobacterium_sp._KMS___WP__039111045.1

501. Bacteroidetes___Flavobacterium_sp._TAB_87___KVV16368.1

502. Bacteroidetes___Flavobacterium_sp._LB2P22___WP__132071482.1

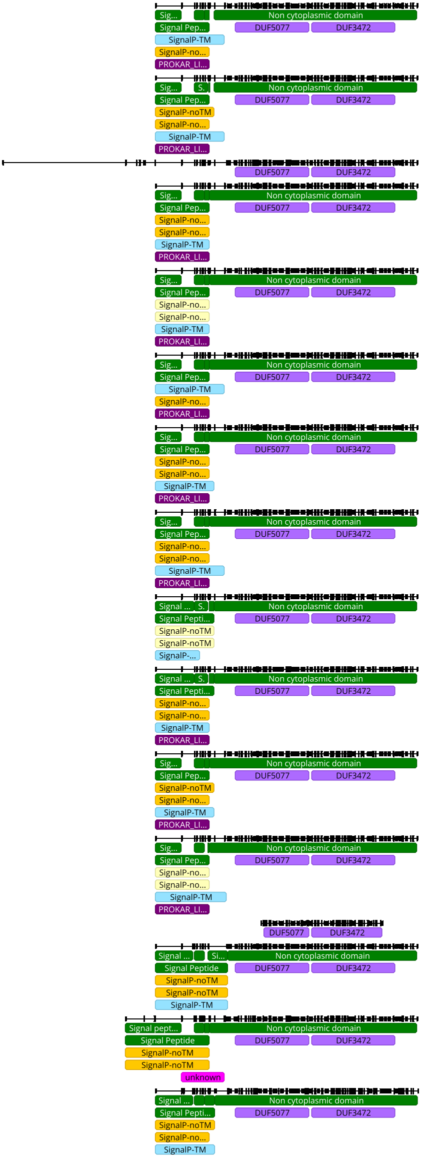

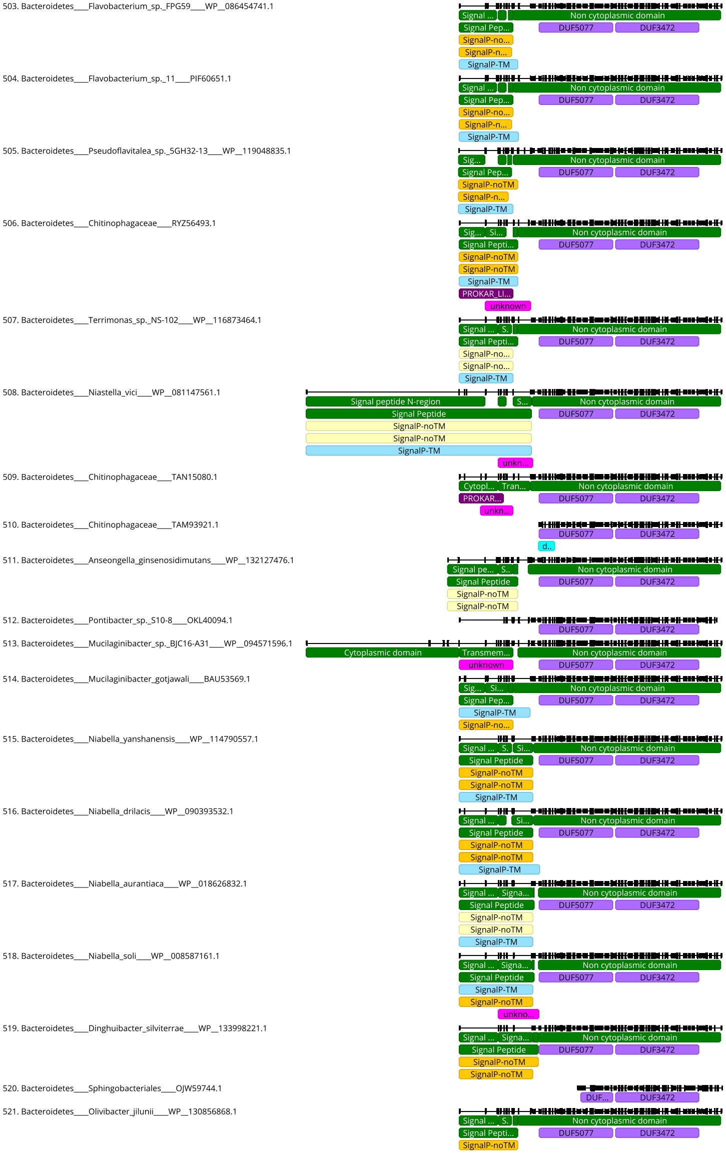
503. Bacteroidetes___Flavobacterium_sp._FPG59___WP__086454741.1
Signal ...
Non cytoplasmic domain
Signal Pep...
DUF5077
DUF3472
SignalP-no...
SignalP-n...
SignalP-TM
504. Bacteroidetes___Flavobacterium_sp._11___PIF60651.1
Signal ...
Non cytoplasmic domain
Signal Pep...
DUF5077
DUF3472
SignalP-no...
SignalP-n...
SignalP-TM
505. Bacteroidetes___Pseudoflavitalea_sp._5GH32-13___WP__119048835.1
Sig...
Non cytoplasmic domain
Signal Pep...
DUF5077
DUF3472
SignalP-noTM
SignalP-n...
SignalP-TM
506. Bacteroidetes___Chitinophagaceae___RYZ56493.1
Sig...
Si...
Non cytoplasmic domain
Signal Pepti...
DUF5077
DUF3472
SignalP-noTM
SignalP-noTM
SignalP-TM
PROKAR_LI...
unknown
507. Bacteroidetes___Terrimonas_sp._NS-102___WP__116873464.1
Signal ...
S.
Non cytoplasmic domain
Signal Pepti...
DUF5077
DUF3472
SignalP-no...
SignalP-no...
SignalP-TM
508. Bacteroidetes___Niastella_vici___WP__081147561.1
Signal peptide N-region
S...
Non cytoplasmic domain
Signal Peptide
DUF5077
DUF3472
SignalP-noTM
SignalP-noTM
SignalP-TM
unkn...
509. Bacteroidetes___Chitinophagaceae___TAN15080.1
Cytopl...
Tran...
Non cytoplasmic domain
PROKAR...
DUF5077
DUF3472
unkn...
510. Bacteroidetes___Chitinophagaceae___TAM93921.1
DUF5077
DUF3472
d..
511. Bacteroidetes___Anseongella_ginsenosidimutans___WP__132127476.1
Signal pe...
S..
Non cytoplasmic domain
Signal Peptide
DUF5077
DUF3472
SignalP-noTM
SignalP-noTM
512. Bacteroidetes___Pontibacter_sp._S10-8___OKL40094.1
DUF5077
DUF3472
513. Bacteroidetes___Mucilaginibacter_sp._BJC16-A31___WP__094571596.1
Cytoplasmic domain
Transmem...
Non cytoplasmic domain
unknown
DUF5077
DUF3472
514. Bacteroidetes___Mucilaginibacter_gotjawali___BAU53569.1
Sig...
Si...
Non cytoplasmic domain
Signal Pep...
DUF5077
DUF3472
SignalP-TM
SignalP-no...
515. Bacteroidetes___Niabella_yanshanensis___WP__114790557.1
Signal ...
S.
Si...
Non cytoplasmic domain
Signal Peptide
DUF5077
DUF3472
SignalP-noTM
SignalP-noTM
SignalP-TM
516. Bacteroidetes___Niabella_drilacis___WP__090393532.1
Signal ...
Si...
Non cytoplasmic domain
Signal Peptide
DUF5077
DUF3472
SignalP-noTM
SignalP-noTM
SignalP-TM
517. Bacteroidetes___Niabella_aurantiaca___WP__018626832.1
Signal ...
Signa...
Non cytoplasmic domain
Signal Peptide
DUF5077
DUF3472
SignalP-noTM
SignalP-noTM
SignalP-TM
518. Bacteroidetes___Niabella_soli___WP__008587161.1
Signal ...
Signa...
Non cytoplasmic domain
Signal Peptide
DUF5077
DUF3472
SignalP-TM
SignalP-noTM
unkno...
519. Bacteroidetes___Dinghuibacter_silviterrae___WP__133998221.1
Signal ...
Signa...
Non cytoplasmic domain
Signal Peptide
DUF5077
DUF3472
SignalP-noTM
SignalP-noTM
520. Bacteroidetes___Sphingobacteriales___OJW59744.1
DUF...
DUF3472
521. Bacteroidetes___Olivibacter_jilunii___WP__130856868.1
Signal ...
S.
Non cytoplasmic domain
Signal Pepti...
DUF5077
DUF3472
SignalP-noTM

522. Bacteroidetes___Olivibacter_sp._XZL3___WP__134090823.1

523. Bacteroidetes___Pseudosphingobacterium_domesticum___WP__09333...

524. Bacteroidetes___Pedobacter_sp._SM1810___WP__118193357.1

525. Bacteroidetes___Sphingobacterium_sp._JUb78___WP__132772335.1

526. Bacteroidetes___Sphingobacterium_sp._ML3W___WP__038695904.1

527. Bacteroidetes___Sphingobacterium_faecium___PTX09114.1

528. Bacteroidetes___Sphingobacterium_sp._PM2-P1-29___CDS93590.1

529. Bacteroidetes___Sphingobacterium_spiritivorum___SUJ06496.1

530. Bacteroidetes___Sphingobacteriaceae___WP__033564800.1

531. Bacteroidetes___Sphingobacterium_psychroaquaticum___SMG50001.1

532. Bacteroidetes___Sphingobacterium_wenxiniae___SFT13372.1

533. Bacteroidetes___Chitinophaga_parva___WP__108688473.1

534. Bacteroidetes___Sphingobacteriales___RTL59027.1

535. Bacteroidetes___Sphingobacteriales___OQY92019.1

536. Bacteroidetes___Vibrionimonas_magnilacihabitans___WP__105470646.1

537. Bacteroidetes___Lacibacter_sp._TTM-7___WP__129131688.1

538. Bacteroidetes___Flavihumibacter_sp._CACIAM_22H1___KYP16330.1

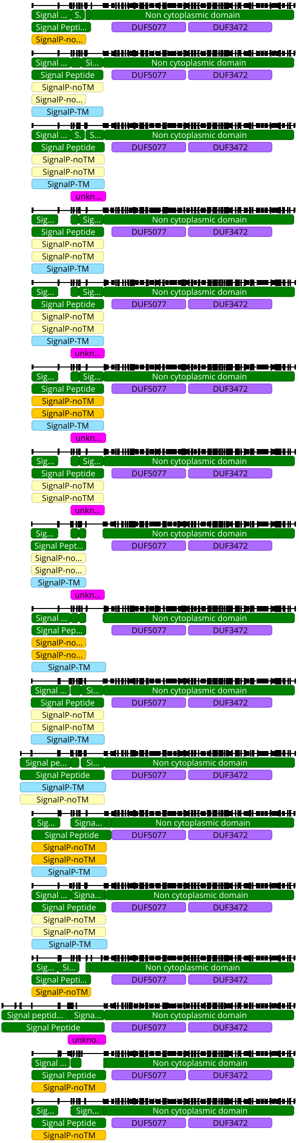

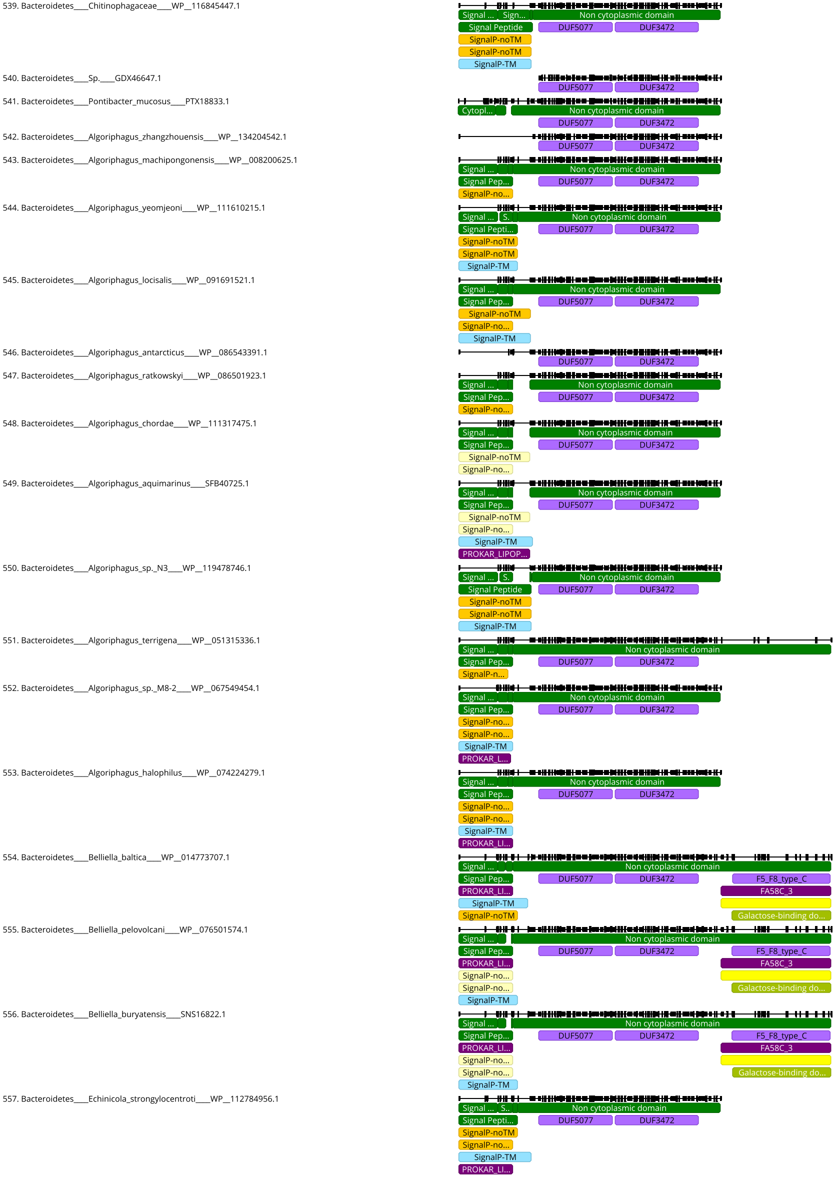
539. Bacteroidetes___Chitinophagaceae___WP__116845447.1
Signal ...
Sign...
Non cytoplasmic domain
Signal Peptide
DUF5077
DUF3472
SignalP-noTM
SignalP-noTM
SignalP-TM
540. Bacteroidetes___Sp.___GDX46647.1
DUF5077
DUF3472
541. Bacteroidetes___Pontibacter_mucosus___PTX18833.1
Cytopl...
Non cytoplasmic domain
DUF5077
DUF3472
542. Bacteroidetes___Algoriphagus_zhangzhouensis___WP__134204542.1
DUF5077
DUF3472
543. Bacteroidetes___Algoriphagus_machipongonensis___WP__008200625.1
Signal ...
Non cytoplasmic domain
Signal Pep...
DUF5077
DUF3472
SignalP-no...
544. Bacteroidetes___Algoriphagus_yeomjeoni___WP__111610215.1
Signal ...
S.
Non cytoplasmic domain
Signal Pepti...
DUF5077
DUF3472
SignalP-noTM
SignalP-noTM
SignalP-TM
545. Bacteroidetes___Algoriphagus_locisalis___WP__091691521.1
Signal ...
Non cytoplasmic domain
Signal Pep...
DUF5077
DUF3472
SignalP-noTM
SignalP-no...
SignalP-TM
546. Bacteroidetes___Algoriphagus_antarcticus___WP__086543391.1
DUF5077
DUF3472
547. Bacteroidetes___Algoriphagus_ratkowskyi___WP__086501923.1
Signal ...
Non cytoplasmic domain
Signal Pep...
DUF5077
DUF3472
SignalP-no...
548. Bacteroidetes___Algoriphagus_chordae___WP__111317475.1
Signal ...
Non cytoplasmic domain
Signal Pep...
DUF5077
DUF3472
SignalP-noTM
SignalP-no...
549. Bacteroidetes___Algoriphagus_aquimarinus___SFB40725.1
Signal ...
Non cytoplasmic domain
Signal Pep...
DUF5077
DUF3472
SignalP-noTM
SignalP-no...
SignalP-TM
PROKAR_LIPOP...
550. Bacteroidetes___Algoriphagus_sp._N3___WP__119478746.1
Signal ...
S.
Non cytoplasmic domain
Signal Peptide
DUF5077
DUF3472
SignalP-noTM
SignalP-noTM
SignalP-TM
551. Bacteroidetes___Algoriphagus_terrigena___WP__051315336.1
Signal ...
Non cytoplasmic domain
Signal Pep...
DUF5077
DUF3472
SignalP-n...
552. Bacteroidetes___Algoriphagus_sp._M8-2___WP__067549454.1
Signal ...
Non cytoplasmic domain
Signal Pep...
DUF5077
DUF3472
SignalP-no...
SignalP-no...
SignalP-TM
PROKAR_L...
553. Bacteroidetes___Algoriphagus_halophilus___WP__074224279.1
Signal ...
Non cytoplasmic domain
Signal Pep...
DUF5077
DUF3472
SignalP-no...
SignalP-no...
SignalP-TM
PROKAR_LI...
554. Bacteroidetes___Belliella_baltica___WP__014773707.1
Signal ...
Non cytoplasmic domain
Signal Pep...
DUF5077
DUF3472
F5_F8_type_C
PROKAR_LI...
FA58C_3
SignalP-TM
SignalP-noTM
Galactose-binding do...
555. Bacteroidetes___Belliella_pelovolcani___WP__076501574.1
Signal ...
Non cytoplasmic domain
Signal Pep...
DUF5077
DUF3472
F5_F8_type_C
PROKAR_LI...
FA58C_3
SignalP-no...
SignalP-no...
Galactose-binding do...
SignalP-TM
556. Bacteroidetes___Belliella_buryatensis___SNS16822.1
Signal ...
Non cytoplasmic domain
Signal Pep...
DUF5077
DUF3472
F5_F8_type_C
PROKAR_LI...
FA58C_3
SignalP-no...
SignalP-no...
Galactose-binding do...
SignalP-TM
557. Bacteroidetes___Echinicola_strongylocentroti___WP__112784956.1
Signal ...
S..
Non cytoplasmic domain
Signal Pepti...
DUF5077
DUF3472
SignalP-noTM
SignalP-no...
SignalP-TM
PROKAR_LI...

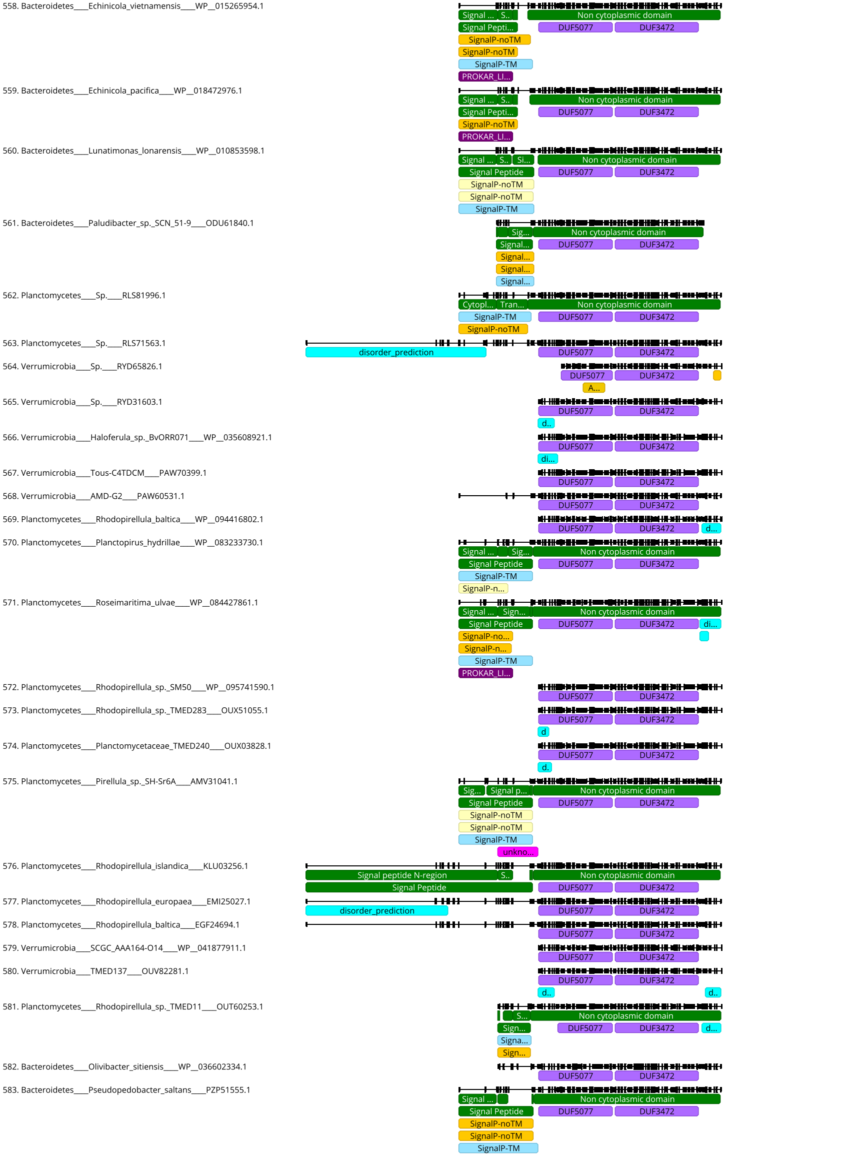
558. Bacteroidetes___Echinicola_vietnamensis___WP__015265954.1
Signal ... S.. Non cytoplasmic domain
Signal Pepti... DUF5077 DUF3472
SignalP-noTM
SignalP-noTM
SignalP-TM
PROKAR_LI...
559. Bacteroidetes___Echinicola_pacifica___WP__018472976.1
Signal ... S.. Non cytoplasmic domain
Signal Pepti... DUF5077 DUF3472
SignalP-noTM
PROKAR_LI...
560. Bacteroidetes___Lunatimonas_lonarensis___WP__010853598.1
Signal ... S.. Si... Non cytoplasmic domain
Signal Peptide DUF5077 DUF3472
SignalP-noTM
SignalP-noTM
SignalP-TM
561. Bacteroidetes___Paludibacter_sp._SCN_51-9___ODU61840.1
Sig... Non cytoplasmic domain
Signal... DUF5077 DUF3472
Signal...
Signal...
Signal...
562. Planctomycetes___Sp.___RLS81996.1
Cytopl... Tran... Non cytoplasmic domain
SignalP-TM DUF5077 DUF3472
SignalP-noTM
563. Planctomycetes___Sp.___RLS71563.1
disorder_prediction DUF5077 DUF3472
564. Verrumicrobia___Sp.___RYD65826.1
DUF5077 DUF3472
A...
565. Verrumicrobia___Sp.___RYD31603.1
DUF5077 DUF3472
d..
566. Verrumicrobia___Haloferula_sp._BvORR071___WP__035608921.1
DUF5077 DUF3472
di...
567. Verrumicrobia___Tous-C4TDCM___PAW70399.1
DUF5077 DUF3472
568. Verrumicrobia___AMD-G2___PAW60531.1
DUF5077 DUF3472
569. Planctomycetes___Rhodopirellula_baltica___WP__094416802.1
DUF5077 DUF3472 d...
570. Planctomycetes___Planctopirus_hydrillae___WP__083233730.1
Signal ... Sig... Non cytoplasmic domain
Signal Peptide DUF5077 DUF3472
SignalP-TM
SignalP-n...
571. Planctomycetes___Roseimaritima_ulvae___WP__084427861.1
Signal ... Sign... Non cytoplasmic domain
Signal Peptide DUF5077 DUF3472 di...
SignalP-no...
SignalP-n...
SignalP-TM
PROKAR_LI...
572. Planctomycetes___Rhodopirellula_sp._SM50___WP__095741590.1
DUF5077 DUF3472
573. Planctomycetes___Rhodopirellula_sp._TMED283___OUX51055.1
DUF5077 DUF3472
d
574. Planctomycetes___Planctomycetaceae_TMED240___OUX03828.1
DUF5077 DUF3472
d.
575. Planctomycetes___Pirellula_sp._SH-Sr6A___AMV31041.1
Sig... Signal p... Non cytoplasmic domain
Signal Peptide DUF5077 DUF3472
SignalP-noTM
SignalP-noTM
SignalP-TM
unkno...
576. Planctomycetes___Rhodopirellula_islandica___KLU03256.1
Signal peptide N-region S.. Non cytoplasmic domain
Signal Peptide DUF5077 DUF3472
577. Planctomycetes___Rhodopirellula_europaea___EMI25027.1
disorder_prediction DUF5077 DUF3472
578. Planctomycetes___Rhodopirellula_baltica___EGF24694.1
DUF5077 DUF3472
579. Verrumicrobia___SCGC_AAA164-O14___WP__041877911.1
DUF5077 DUF3472
580. Verrumicrobia___TMED137___OUV82281.1
DUF5077 DUF3472
d.. d..
581. Planctomycetes___Rhodopirellula_sp._TMED11___OUT60253.1
S... Non cytoplasmic domain
Sign... DUF5077 DUF3472 d...
Signa...
Sign...
582. Bacteroidetes___Olivibacter_sitiensis___WP__036602334.1
DUF5077 DUF3472
583. Bacteroidetes___Pseudopedobacter_saltans___PZP51555.1
Signal ... Non cytoplasmic domain
Signal Peptide DUF5077 DUF3472
SignalP-noTM
SignalP-noTM
SignalP-TM

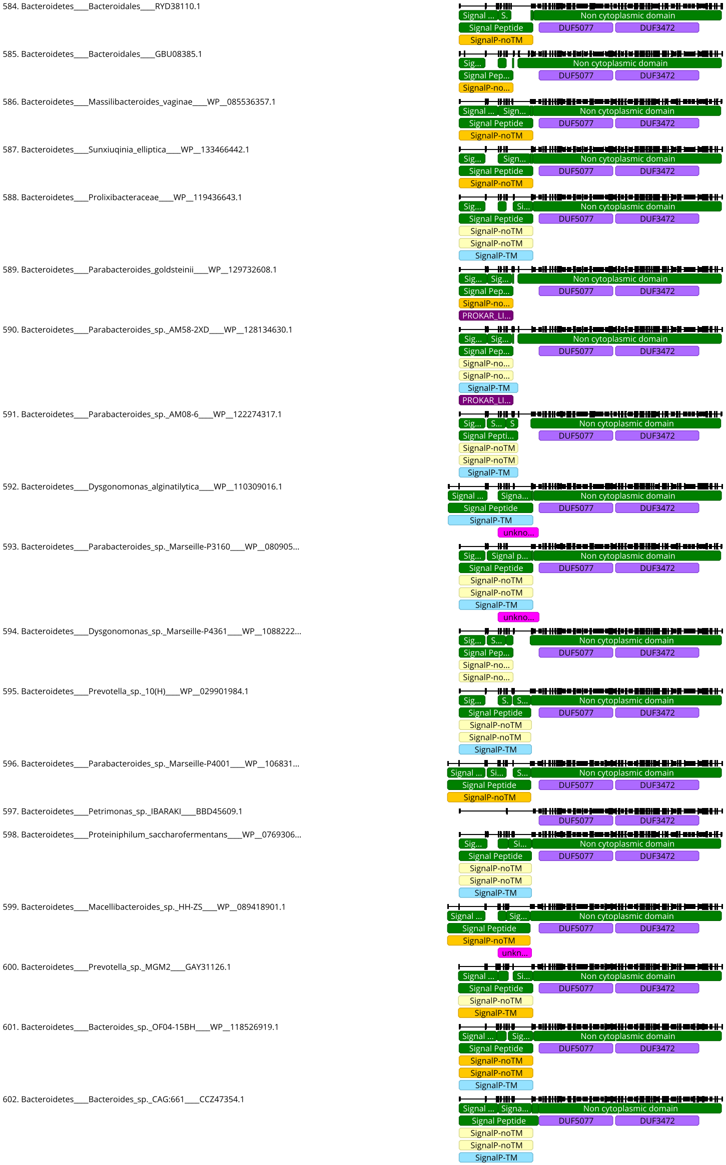
584. Bacteroidetes___Bacteroidales___RYD38110.1
Signal ... S. Non cytoplasmic domain
Signal Peptide DUF5077 DUF3472
SignalP-noTM
585. Bacteroidetes___Bacteroidales___GBU08385.1
Sig... Non cytoplasmic domain
Signal Pep... DUF5077 DUF3472
SignalP-no...
586. Bacteroidetes___Massilibacteroides_vaginae___WP__085536357.1
Signal ... Sign... Non cytoplasmic domain
Signal Peptide DUF5077 DUF3472
SignalP-noTM
587. Bacteroidetes___Sunxiuqinia_elliptica___WP__133466442.1
Sig... Sign... Non cytoplasmic domain
Signal Peptide DUF5077 DUF3472
SignalP-noTM
588. Bacteroidetes___Prolixibacteraceae___WP__119436643.1
Sig... Si... Non cytoplasmic domain
Signal Peptide DUF5077 DUF3472
SignalP-noTM
SignalP-noTM
SignalP-TM
589. Bacteroidetes___Parabacteroides_goldsteinii___WP__129732608.1
Sig... Sig... Non cytoplasmic domain
Signal Pep... DUF5077 DUF3472
SignalP-no...
PROKAR_LI...
590. Bacteroidetes___Parabacteroides_sp._AM58-2XD___WP__128134630.1
Sig... Sig... Non cytoplasmic domain
Signal Pep... DUF5077 DUF3472
SignalP-no...
SignalP-no...
SignalP-TM
PROKAR_LI...
591. Bacteroidetes___Parabacteroides_sp._AM08-6___WP__122274317.1
Sig... S... S Non cytoplasmic domain
Signal Pepti... DUF5077 DUF3472
SignalP-noTM
SignalP-noTM
SignalP-TM
592. Bacteroidetes___Dysgonomonas_alginatilytica___WP__110309016.1
Signal ... Signa... Non cytoplasmic domain
Signal Peptide DUF5077 DUF3472
SignalP-TM
unkno...
593. Bacteroidetes___Parabacteroides_sp._Marseille-P3160___WP__080905...
Sig... Signal p... Non cytoplasmic domain
Signal Peptide DUF5077 DUF3472
SignalP-noTM
SignalP-noTM
SignalP-TM
unkno...
594. Bacteroidetes___Dysgonomonas_sp._Marseille-P4361___WP__1088222...
Sig... S... Non cytoplasmic domain
Signal Pep... DUF5077 DUF3472
SignalP-no...
SignalP-no...
595. Bacteroidetes___Prevotella_sp._10(H)___WP__029901984.1
Sig... S. S... Non cytoplasmic domain
Signal Peptide DUF5077 DUF3472
SignalP-noTM
SignalP-noTM
SignalP-TM
596. Bacteroidetes___Parabacteroides_sp._Marseille-P4001___WP__106831...
Signal ... Si... S... Non cytoplasmic domain
Signal Peptide DUF5077 DUF3472
SignalP-noTM
597. Bacteroidetes___Petrimonas_sp._IBARAKI___BBD45609.1
DUF5077 DUF3472
598. Bacteroidetes___Proteiniphilum_saccharofermentans___WP__0769306...
Sig... Si... Non cytoplasmic domain
Signal Peptide DUF5077 DUF3472
SignalP-noTM
SignalP-noTM
SignalP-TM
599. Bacteroidetes___Macellibacteroides_sp._HH-ZS___WP__089418901.1
Signal ... Sig... Non cytoplasmic domain
Signal Peptide DUF5077 DUF3472
SignalP-noTM
unkn...
600. Bacteroidetes___Prevotella_sp._MGM2___GAY31126.1
Signal ... Si... Non cytoplasmic domain
Signal Peptide DUF5077 DUF3472
SignalP-noTM
SignalP-TM
601. Bacteroidetes___Bacteroides_sp._OF04-15BH___WP__118526919.1
Signal ... Sig... Non cytoplasmic domain
Signal Peptide DUF5077 DUF3472
SignalP-noTM
SignalP-noTM
SignalP-TM
602. Bacteroidetes___Bacteroides_sp._CAG:661___CCZ47354.1
Signal ... Signa... Non cytoplasmic domain
Signal Peptide DUF5077 DUF3472
SignalP-noTM
SignalP-noTM
SignalP-TM

603. Bacteroidetes___Bacteroides_sp._CAG:598___CCX62870.1

604. Bacteroidetes___Odoribacter_sp.OF09-27XD___WP__118773131.1

605. Bacteroidetes___Bacteroides_neonati___WP__042368425.1

606. Bacteroidetes___Bacteroides_sp._KCTC_15687___WP__125041105.1

607. Bacteroidetes___Bacteroidessp._43__46___OKZ21583.1

608. Bacteroidetes___Prevotella_sp._CAG:617___CDF22282.1

609. Bacteroidetes___Prevotella_sp._CAG:891___CDE87178.1

610. Bacteroidetes___Mucinivorans_hirudinis___CDN33116.1

611. Bacteroidetes___Sphingobacterium_detergens___WP__120257125.1

612. Bacteroidetes___Sphingobacterium_sp._HMA12___WP__104382861.1

613. Bacteroidetes___Microscilla_marina___WP__004155978.1

614. Bacteroidetes___Acetobacteroides_hydrogenigenes___WP__13183863...

615. Bacteroidetes___Bacteroidales___WP__082253572.1

616. Bacteroidetes___Salinibacter_ruber___WP__118839465.1

617. Bacteroidetes___Salinibacter_altiplanensis___WP__103026544.1

618. Bacteroidetes___Salinibacter_sp._10B___WP__105013206.1

619. Bacteroidetes___Haliscomenobacter_hydrossis___AEE52249.1

620. Bacteroidetes___Flavihumibacter_petaseus___GAO41358.1

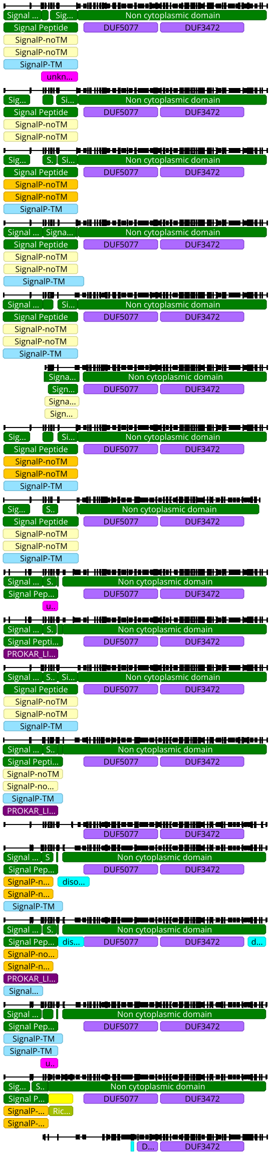

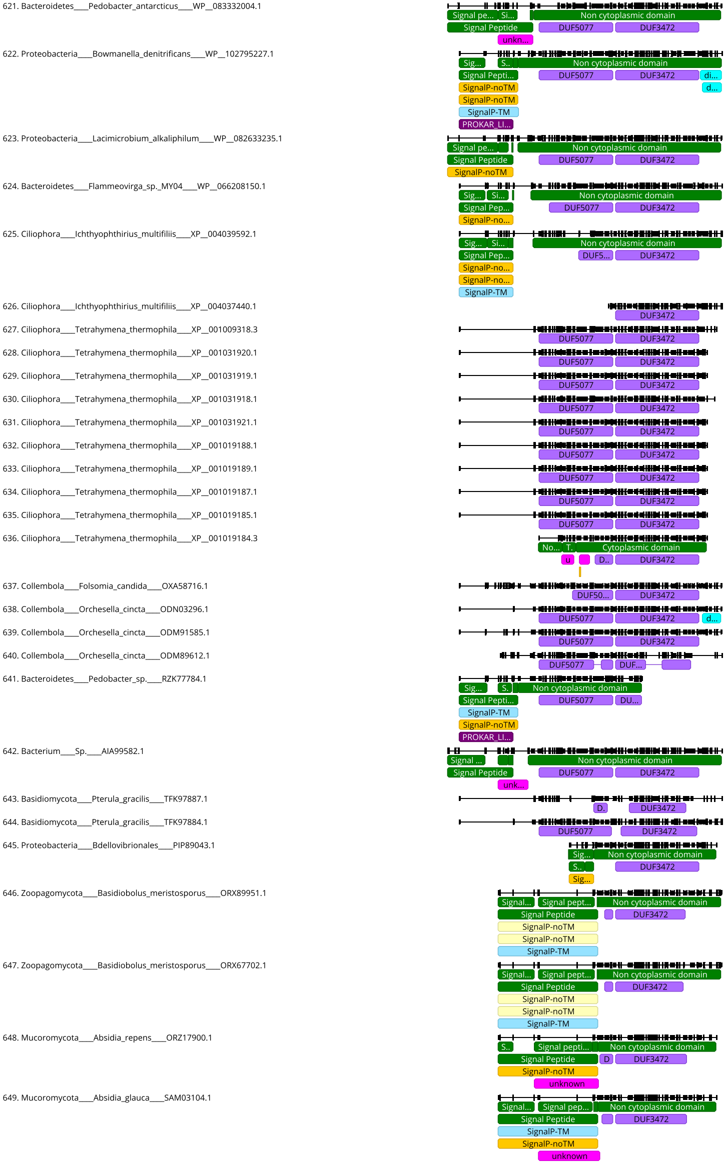

621. Bacteroidetes____Pedobacter_antarcticus____WP__083332004.1
Signal pe... Si... Non cytoplasmic domain
Signal Peptide DUF5077 DUF3472
unkn...
622. Proteobacteria____Bowmanella_denitrificans____WP__102795227.1
Sig... S.. Non cytoplasmic domain
Signal Pepti... DUF5077 DUF3472 di...
SignalP-noTM d...
SignalP-noTM
SignalP-TM
PROKAR_LI...
623. Proteobacteria____Lacimicrobium_alkaliphilum____WP__082633235.1
Signal pe... Non cytoplasmic domain
Signal Peptide DUF5077 DUF3472
SignalP-noTM
624. Bacteroidetes____Flammeovirga_sp._MY04____WP__066208150.1
Sig... Si... Non cytoplasmic domain
Signal Pep... DUF5077 DUF3472
SignalP-no...
625. Ciliophora____Ichthyophthirius_multifiliis____XP__004039592.1
Sig... Si... Non cytoplasmic domain
Signal Pep... DUF5... DUF3472
SignalP-no...
SignalP-no...
SignalP-TM
626. Ciliophora____Ichthyophthirius_multifiliis____XP__004037440.1
DUF3472
627. Ciliophora____Tetrahymena_thermophila____XP__001009318.3
DUF5077 DUF3472
628. Ciliophora____Tetrahymena_thermophila____XP__001031920.1
DUF5077 DUF3472
629. Ciliophora____Tetrahymena_thermophila____XP__001031919.1
DUF5077 DUF3472
630. Ciliophora____Tetrahymena_thermophila____XP__001031918.1
DUF5077 DUF3472
631. Ciliophora____Tetrahymena_thermophila____XP__001031921.1
DUF5077 DUF3472
632. Ciliophora____Tetrahymena_thermophila____XP__001019188.1
DUF5077 DUF3472
633. Ciliophora____Tetrahymena_thermophila____XP__001019189.1
DUF5077 DUF3472
634. Ciliophora____Tetrahymena_thermophila____XP__001019187.1
DUF5077 DUF3472
635. Ciliophora____Tetrahymena_thermophila____XP__001019185.1
DUF5077 DUF3472
636. Ciliophora____Tetrahymena_thermophila____XP__001019184.3
No... T. Cytoplasmic domain
u D.. DUF3472
637. Collembola____Folsomia_candida____OXA58716.1
DUF50... DUF3472
638. Collembola____Orchesella_cincta____ODN03296.1
DUF5077 DUF3472 d...
639. Collembola____Orchesella_cincta____ODM91585.1
DUF5077 DUF3472
640. Collembola____Orchesella_cincta____ODM89612.1
DUF5077 DUF...
641. Bacteroidetes____Pedobacter_sp.____RZK77784.1
Sig... S. Non cytoplasmic domain
Signal Pepti... DUF5077 DU...
SignalP-TM
SignalP-noTM
PROKAR_LI...
642. Bacterium____Sp.____AIA99582.1
Signal ... Non cytoplasmic domain
Signal Peptide DUF5077 DUF3472
unk...
643. Basidiomycota____Pterula_gracilis____TFK97887.1
D. DUF3472
644. Basidiomycota____Pterula_gracilis____TFK97884.1
DUF5077 DUF3472
645. Proteobacteria____Bdellovibrionales____PIP89043.1
Sig... Non cytoplasmic domain
S.. DUF3472
Sig...
646. Zoopagomycota____Basidiobolus_meristosporus____ORX89951.1
Signal... Signal pept... Non cytoplasmic domain
Signal Peptide DUF3472
SignalP-noTM
SignalP-noTM
SignalP-TM
647. Zoopagomycota____Basidiobolus_meristosporus____ORX67702.1
Signal... Signal pept... Non cytoplasmic domain
Signal Peptide DUF3472
SignalP-noTM
SignalP-noTM
SignalP-TM
648. Mucoromycota____Absidia_repens____ORZ17900.1
S.. Signal pepti... Non cytoplasmic domain
Signal Peptide D DUF3472
SignalP-noTM
unknown
649. Mucoromycota____Absidia_glauca____SAM03104.1
Signal... Signal pep... Non cytoplasmic domain
Signal Peptide DUF3472
SignalP-TM
SignalP-noTM
unknown

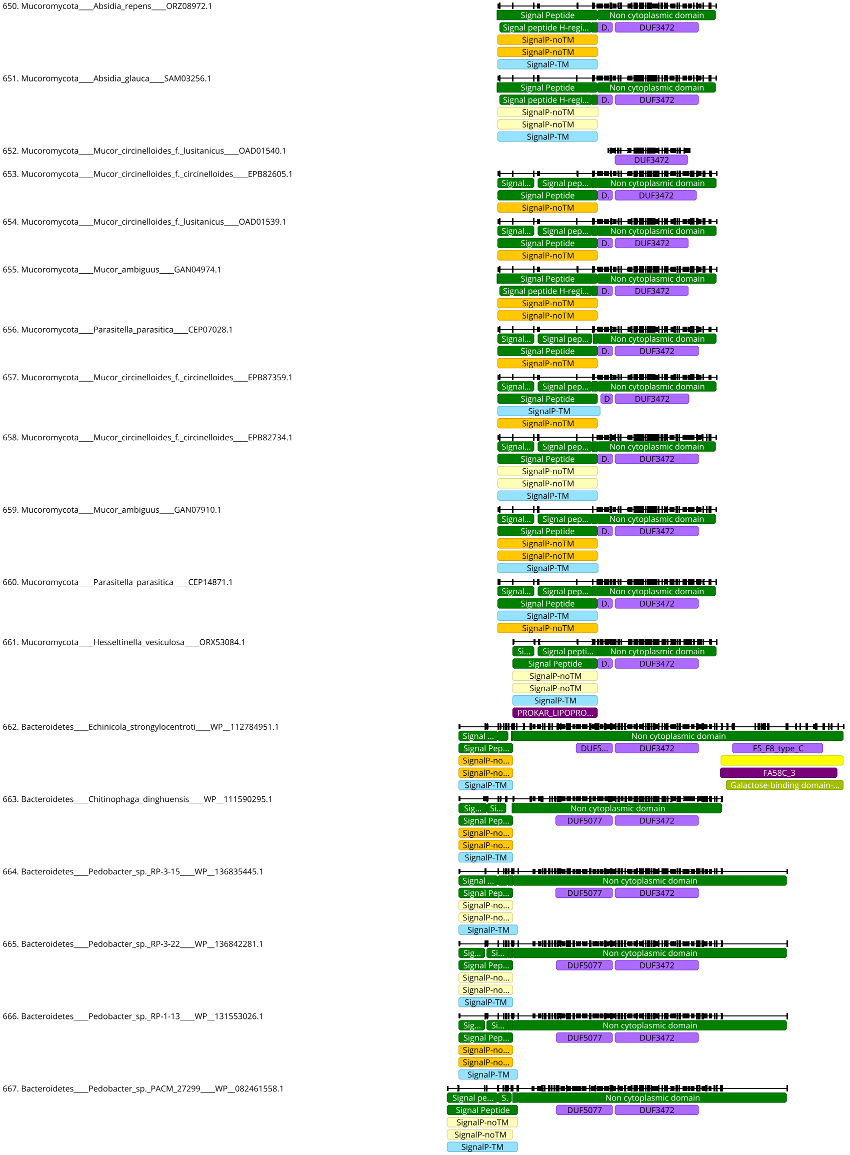
650. Mucoromycota___Absidia_repens___ORZ08972.1
Signal Peptide
Non cytoplasmic domain
Signal peptide H-regi...
D.
DUF3472
SignalP-noTM
SignalP-noTM
SignalP-TM
651. Mucoromycota___Absidia_glauca___SAM03256.1
Signal Peptide
Non cytoplasmic domain
Signal peptide H-regi...
D.
DUF3472
SignalP-noTM
SignalP-noTM
SignalP-TM
652. Mucoromycota___Mucor_circinelloides_f._lusitanicus___OAD01540.1
DUF3472
653. Mucoromycota___Mucor_circinelloides_f._circinelloides___EPB82605.1
Signal...
Signal pep...
Non cytoplasmic domain
Signal Peptide
D.
DUF3472
SignalP-noTM
654. Mucoromycota___Mucor_circinelloides_f._lusitanicus___OAD01539.1
Signal...
Signal pep...
Non cytoplasmic domain
Signal Peptide
D.
DUF3472
SignalP-noTM
655. Mucoromycota___Mucor_ambiguus___GAN04974.1
Signal Peptide
Non cytoplasmic domain
Signal peptide H-regi...
D.
DUF3472
SignalP-noTM
SignalP-noTM
656. Mucoromycota___Parasitella_parasitica___CEP07028.1
Signal...
Signal pep...
Non cytoplasmic domain
Signal Peptide
D.
DUF3472
SignalP-noTM
657. Mucoromycota___Mucor_circinelloides_f._circinelloides___EPB87359.1
Signal...
Signal pep...
Non cytoplasmic domain
Signal Peptide
D
DUF3472
SignalP-TM
SignalP-noTM
658. Mucoromycota___Mucor_circinelloides_f._circinelloides___EPB82734.1
Signal...
Signal pep...
Non cytoplasmic domain
Signal Peptide
D.
DUF3472
SignalP-noTM
SignalP-noTM
SignalP-TM
659. Mucoromycota___Mucor_ambiguus___GAN07910.1
Signal...
Signal pep...
Non cytoplasmic domain
Signal Peptide
D.
DUF3472
SignalP-noTM
SignalP-noTM
SignalP-TM
660. Mucoromycota___Parasitella_parasitica___CEP14871.1
Signal...
Signal pep...
Non cytoplasmic domain
Signal Peptide
D.
DUF3472
SignalP-TM
SignalP-noTM
661. Mucoromycota___Hesseltinella_vesiculosa___ORX53084.1
Si...
Signal pepti...
Non cytoplasmic domain
Signal Peptide
D.
DUF3472
SignalP-noTM
SignalP-noTM
SignalP-TM
PROKAR_LIPOPRO...
662. Bacteroidetes___Echinicola_strongylocentroti___WP__112784951.1
Signal ...
Non cytoplasmic domain
Signal Pep...
DUF5...
DUF3472
F5_F8_type_C
SignalP-no...
SignalP-no...
FA58C_3
SignalP-TM
Galactose-binding domain-...
663. Bacteroidetes___Chitinophaga_dinghuensis___WP__111590295.1
Sig...
Si...
Non cytoplasmic domain
Signal Pep...
DUF5077
DUF3472
SignalP-no...
SignalP-no...
SignalP-TM
664. Bacteroidetes___Pedobacter_sp._RP-3-15___WP__136835445.1
Signal ...
Non cytoplasmic domain
Signal Pep...
DUF5077
DUF3472
SignalP-no...
SignalP-no...
SignalP-TM
665. Bacteroidetes___Pedobacter_sp._RP-3-22___WP__136842281.1
Sig...
Si...
Non cytoplasmic domain
Signal Pep...
DUF5077
DUF3472
SignalP-no...
SignalP-no...
SignalP-TM
666. Bacteroidetes___Pedobacter_sp._RP-1-13___WP__131553026.1
Sig...
Si...
Non cytoplasmic domain
Signal Pep...
DUF5077
DUF3472
SignalP-no...
SignalP-no...
SignalP-TM
667. Bacteroidetes___Pedobacter_sp._PACM_27299___WP__082461558.1
Signal pe...
S.
Non cytoplasmic domain
Signal Peptide
DUF5077
DUF3472
SignalP-noTM
SignalP-noTM
SignalP-TM

668. Bacteroidetes___Pedobacter_insulae___SFH40004.1

669. Bacteroidetes___Niabella_aurantiaca___WP__018627346.1

670. Bacteroidetes___Niabella_drilacis___SDC67727.1

671. Bacteroidetes___Prevotella_sp._10(H)___WP__081852614.1

672. Bacteroidetes___Sphingobacterium_spiritivorum___WP__115168955.1

673. Bacteroidetes___Sphingobacterium_nematocida___WP__079642968.1

674. Bacteroidetes___Elizabethkingia_miricola___WP__086980541.1

675. Bacteroidetes___Arachidicoccus_rhizosphaerae___WP__091395110.1

676. Bacteroidetes___Parabacteroides_sp._Marseille-P3160___WP__080903...

677. Verrumicrobia___Opitutae___GBL30550.1

678. Verrumicrobia___Opitutae___GBL23218.1

679. Bacteroidetes___Chitinophaga_ginsengisoli___WP__106602872.1

680. Bacteroidetes___Siphonobacter_sp._BAB-5385___WP__094815794.1

681. Bacteroidetes___Chitinophaga_costaii___WP__089711646.1

682. Bacteroidetes___Prevotella_stercorea___WP__118355700.1

683. Bacteroidetes___Prevotella_sp._CAG:520___CDB05745.1

684. Bacteroidetes___Prevotella_sp._CAG:5226___CDA44058.1

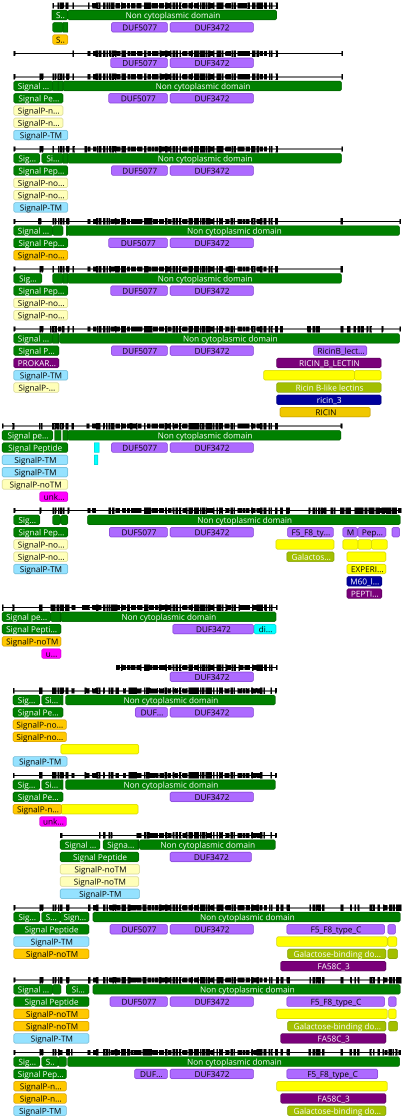

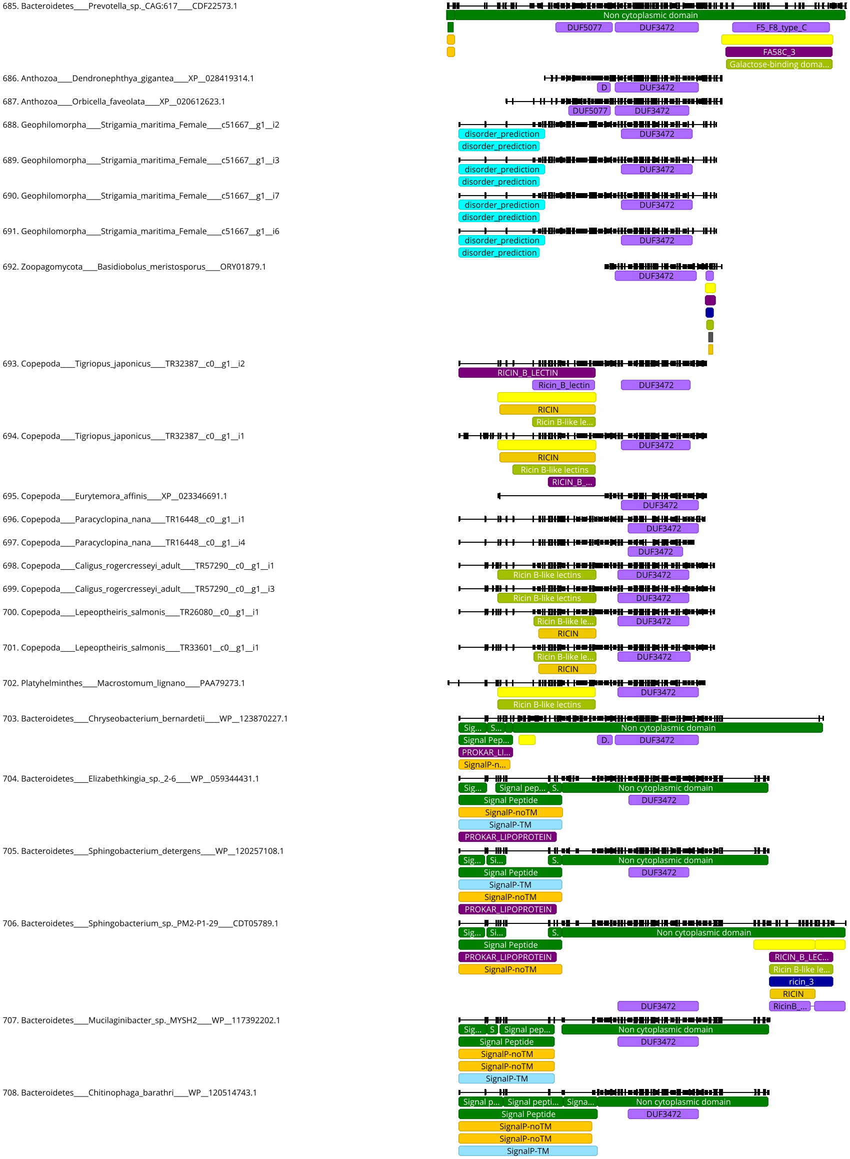

685. Bacteroidetes___Prevotella_sp._CAG:617___CDF22573.1
Non cytoplasmic domain
DUF5077
DUF3472
F5_F8_type_C
FA58C_3
Galactose-binding doma...
686. Anthozoa___Dendronephthya_gigantea___XP__028419314.1
D
DUF3472
687. Anthozoa___Orbicella_faveolata___XP__020612623.1
DUF5077
DUF3472
688. Geophilomorpha___Strigamia_maritima_Female___c51667__g1__i2
disorder_prediction
DUF3472
disorder_prediction
689. Geophilomorpha___Strigamia_maritima_Female___c51667__g1__i3
disorder_prediction
DUF3472
disorder_prediction
690. Geophilomorpha___Strigamia_maritima_Female___c51667__g1__i7
disorder_prediction
DUF3472
disorder_prediction
691. Geophilomorpha___Strigamia_maritima_Female___c51667__g1__i6
disorder_prediction
DUF3472
disorder_prediction
692. Zoopagomycota___Basidiobolus_meristosporus___ORY01879.1
DUF3472
693. Copepoda___Tigriopus_japonicus___TR32387__c0__g1__i2
RICIN_B_LECTIN
Ricin_B_lectin
DUF3472
RICIN
Ricin B-like le...
694. Copepoda___Tigriopus_japonicus___TR32387__c0__g1__i1
DUF3472
RICIN
Ricin B-like lectins
RICIN_B_...
695. Copepoda___Eurytemora_affinis___XP__023346691.1
DUF3472
696. Copepoda___Paracyclopina_nana___TR16448__c0__g1__i1
DUF3472
697. Copepoda___Paracyclopina_nana___TR16448__c0__g1__i4
DUF3472
698. Copepoda___Caligus_rogercresseyi_adult___TR57290__c0__g1__i1
Ricin B-like lectins
DUF3472
699. Copepoda___Caligus_rogercresseyi_adult___TR57290__c0__g1__i3
Ricin B-like lectins
DUF3472
700. Copepoda___Lepeoptheiris_salmonis___TR26080__c0__g1__i1
Ricin B-like le...
DUF3472
RICIN
701. Copepoda___Lepeoptheiris_salmonis___TR33601__c0__g1__i1
Ricin B-like le...
DUF3472
RICIN
702. Platyhelminthes___Macrostomum_lignano___PAA79273.1
DUF3472
Ricin B-like lectins
703. Bacteroidetes___Chryseobacterium_bernardetii___WP__123870227.1
Sig...
S...
Non cytoplasmic domain
Signal Pep...
D.
DUF3472
PROKAR_LI...
SignalP-n...
704. Bacteroidetes___Elizabethkingia_sp._2-6___WP__059344431.1
Sig...
Signal pep...
S.
Non cytoplasmic domain
Signal Peptide
DUF3472
SignalP-noTM
SignalP-TM
PROKAR_LIPOPROTEIN
705. Bacteroidetes___Sphingobacterium_detergens___WP__120257108.1
Sig...
Si...
S.
Non cytoplasmic domain
Signal Peptide
DUF3472
SignalP-TM
SignalP-noTM
PROKAR_LIPOPROTEIN
706. Bacteroidetes___Sphingobacterium_sp._PM2-P1-29___CDT05789.1
Sig...
Si...
S.
Non cytoplasmic domain
Signal Peptide
PROKAR_LIPOPROTEIN
RICIN_B_LEC...
SignalP-noTM
Ricin B-like le...
ricin_3
RICIN
DUF3472
RicinB_...
707. Bacteroidetes___Mucilaginibacter_sp._MYSH2___WP__117392202.1
Sig...
S
Signal pep...
Non cytoplasmic domain
Signal Peptide
DUF3472
SignalP-noTM
SignalP-noTM
SignalP-TM
708. Bacteroidetes___Chitinophaga_barathri___WP__120514743.1
Signal p...
Signal pepti...
Signa...
Non cytoplasmic domain
Signal Peptide
DUF3472
SignalP-noTM
SignalP-noTM
SignalP-TM

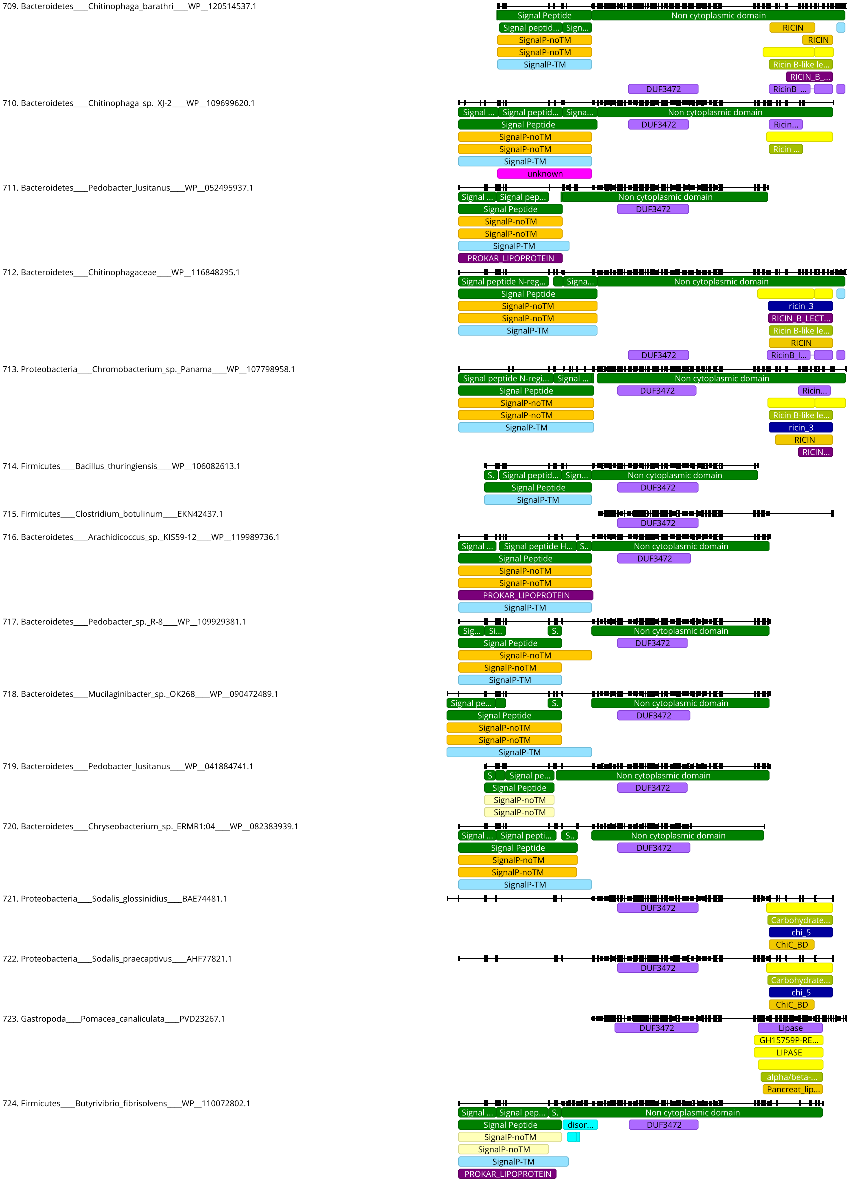
709. Bacteroidetes___Chitinophaga_barathri___WP__120514537.1
Signal Peptide
Non cytoplasmic domain
Signal peptid...
Sign...
RICIN
SignalP-noTM
RICIN
SignalP-noTM
SignalP-TM
Ricin B-like le...
RICIN_B_...
DUF3472
RicinB_...
710. Bacteroidetes___Chitinophaga_sp._XJ-2___WP__109699620.1
Signal ...
Signal peptid...
Signa...
Non cytoplasmic domain
Signal Peptide
DUF3472
Ricin...
SignalP-noTM
SignalP-noTM
Ricin ...
SignalP-TM
unknown
711. Bacteroidetes___Pedobacter_lusitanus___WP__052495937.1
Signal ...
Signal pep...
Non cytoplasmic domain
Signal Peptide
DUF3472
SignalP-noTM
SignalP-noTM
SignalP-TM
PROKAR_LIPOPROTEIN
712. Bacteroidetes___Chitinophagaceae___WP__116848295.1
Signal peptide N-reg...
Signa...
Non cytoplasmic domain
Signal Peptide
SignalP-noTM
ricin_3
SignalP-noTM
RICIN_B_LECT...
SignalP-TM
Ricin B-like le...
RICIN
DUF3472
RicinB_l...
713. Proteobacteria___Chromobacterium_sp._Panama___WP__107798958.1
Signal peptide N-regi...
Signal ...
Non cytoplasmic domain
Signal Peptide
DUF3472
Ricin...
SignalP-noTM
SignalP-noTM
Ricin B-like le...
SignalP-TM
ricin_3
RICIN
RICIN...
714. Firmicutes___Bacillus_thuringiensis___WP__106082613.1
S.
Signal peptid...
Sign...
Non cytoplasmic domain
Signal Peptide
DUF3472
SignalP-TM
715. Firmicutes___Clostridium_botulinum___EKN42437.1
DUF3472
716. Bacteroidetes___Arachidicoccus_sp._KIS59-12___WP__119989736.1
Signal ...
Signal peptide H...
S..
Non cytoplasmic domain
Signal Peptide
DUF3472
SignalP-noTM
SignalP-noTM
PROKAR_LIPOPROTEIN
SignalP-TM
717. Bacteroidetes___Pedobacter_sp._R-8___WP__109929381.1
Sig...
Si...
S.
Non cytoplasmic domain
Signal Peptide
DUF3472
SignalP-noTM
SignalP-noTM
SignalP-TM
718. Bacteroidetes___Mucilaginibacter_sp._OK268___WP__090472489.1
Signal pe...
S.
Non cytoplasmic domain
Signal Peptide
DUF3472
SignalP-noTM
SignalP-noTM
SignalP-TM
719. Bacteroidetes___Pedobacter_lusitanus___WP__041884741.1
S
Signal pe...
Non cytoplasmic domain
Signal Peptide
DUF3472
SignalP-noTM
SignalP-noTM
720. Bacteroidetes___Chryseobacterium_sp._ERMR1:04___WP__082383939.1
Signal ...
Signal pepti...
S..
Non cytoplasmic domain
Signal Peptide
DUF3472
SignalP-noTM
SignalP-noTM
SignalP-TM
721. Proteobacteria___Sodalis_glossinidius___BAE74481.1
DUF3472
Carbohydrate...
chi_5
ChiC_BD
722. Proteobacteria___Sodalis_praecaptivus___AHF77821.1
DUF3472
Carbohydrate...
chi_5
ChiC_BD
723. Gastropoda___Pomacea_canaliculata___PVD23267.1
DUF3472
Lipase
GH15759P-RE...
LIPASE
alpha/beta-...
Pancreat_lip...
724. Firmicutes___Butyrivibrio_fibrisolvens___WP__110072802.1
Signal ...
Signal pep...
S.
Non cytoplasmic domain
Signal Peptide
disor...
DUF3472
SignalP-noTM
SignalP-noTM
SignalP-TM
PROKAR_LIPOPROTEIN

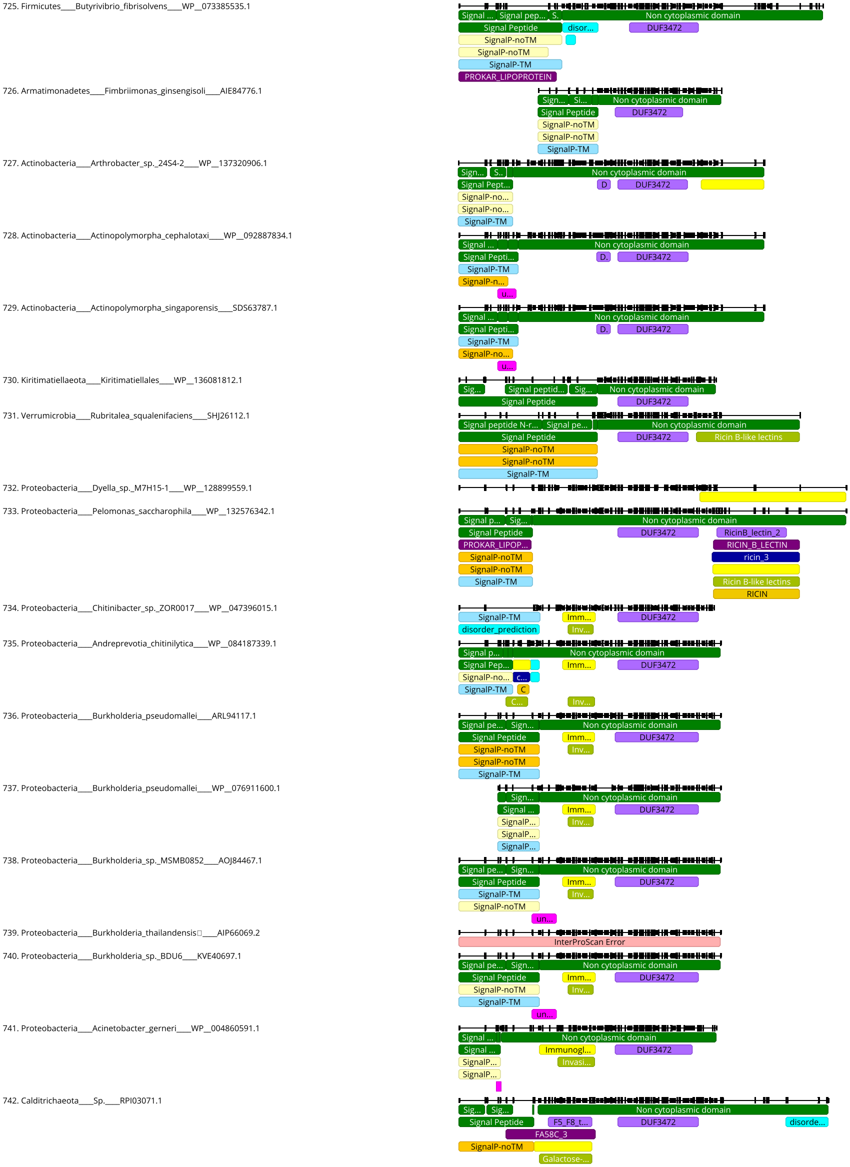

725. Firmicutes___Butyrivibrio_fibrisolvens___WP__073385535.1
Signal ...
Signal pep...
S.
Non cytoplasmic domain
Signal Peptide
disor...
DUF3472
SignalP-noTM
SignalP-noTM
SignalP-TM
PROKAR_LIPOPROTEIN
726. Armatimonadetes___Fimbriimonas_ginsengisoli___AIE84776.1
Sign...
Si...
Non cytoplasmic domain
Signal Peptide
DUF3472
SignalP-noTM
SignalP-noTM
SignalP-TM
727. Actinobacteria___Arthrobacter_sp._24S4-2___WP__137320906.1
Sign...
S..
Non cytoplasmic domain
Signal Pept...
D
DUF3472
SignalP-no...
SignalP-no...
SignalP-TM
728. Actinobacteria___Actinopolymorpha_cephalotaxi___WP__092887834.1
Signal ...
Non cytoplasmic domain
Signal Pepti...
D.
DUF3472
SignalP-TM
SignalP-n...
u...
729. Actinobacteria___Actinopolymorpha_singaporensis___SDS63787.1
Signal ...
Non cytoplasmic domain
Signal Pepti...
D.
DUF3472
SignalP-TM
SignalP-no...
u...
730. Kiritimatiellaeota___Kiritimatiellales___WP__136081812.1
Sig...
Signal peptid...
Sig...
Non cytoplasmic domain
Signal Peptide
DUF3472
731. Verrumicrobia___Rubritalea_squalenifaciens___SHJ26112.1
Signal peptide N-r...
Signal pe...
Non cytoplasmic domain
Signal Peptide
DUF3472
Ricin B-like lectins
SignalP-noTM
SignalP-noTM
SignalP-TM
732. Proteobacteria___Dyella_sp._M7H15-1___WP__128899559.1
733. Proteobacteria___Pelomonas_saccharophila___WP__132576342.1
Signal p...
Sig...
Non cytoplasmic domain
Signal Peptide
DUF3472
RicinB_lectin_2
PROKAR_LIPOP...
RICIN_B_LECTIN
SignalP-noTM
ricin_3
SignalP-noTM
SignalP-TM
Ricin B-like lectins
RICIN
734. Proteobacteria___Chitinibacter_sp._ZOR0017___WP__047396015.1
SignalP-TM
Imm...
DUF3472
disorder_prediction
Inv...
735. Proteobacteria___Andreprevotia_chitinilytica___WP__084187339.1
Signal p...
Non cytoplasmic domain
Signal Pep...
Imm...
DUF3472
SignalP-no...
c...
SignalP-TM
C
C...
Inv...
736. Proteobacteria___Burkholderia_pseudomallei___ARL94117.1
Signal pe...
Sign...
Non cytoplasmic domain
Signal Peptide
Imm...
DUF3472
SignalP-noTM
Inv...
SignalP-noTM
SignalP-TM
737. Proteobacteria___Burkholderia_pseudomallei___WP__076911600.1
Sign...
Non cytoplasmic domain
Signal ...
Imm...
DUF3472
SignalP...
Inv...
SignalP...
SignalP...
738. Proteobacteria___Burkholderia_sp._MSMB0852___AOJ84467.1
Signal pe...
Sign...
Non cytoplasmic domain
Signal Peptide
Imm...
DUF3472
SignalP-TM
Inv...
SignalP-noTM
un...
739. Proteobacteria___Burkholderia_thailandensis___AIP66069.2
InterProScan Error
740. Proteobacteria___Burkholderia_sp._BDU6___KVE40697.1
Signal pe...
Sign...
Non cytoplasmic domain
Signal Peptide
Imm...
DUF3472
SignalP-noTM
Inv...
SignalP-TM
un...
741. Proteobacteria___Acinetobacter_gerneri___WP__004860591.1
Signal ...
Non cytoplasmic domain
Signal ...
Immunogl...
DUF3472
SignalP...
Invasi...
SignalP...
742. Calditrichaeota___Sp.___RPI03071.1
Sig...
Sig...
Non cytoplasmic domain
Signal Peptide
F5_F8_t...
DUF3472
disorde...
FA58C_3
SignalP-noTM
Galactose-...

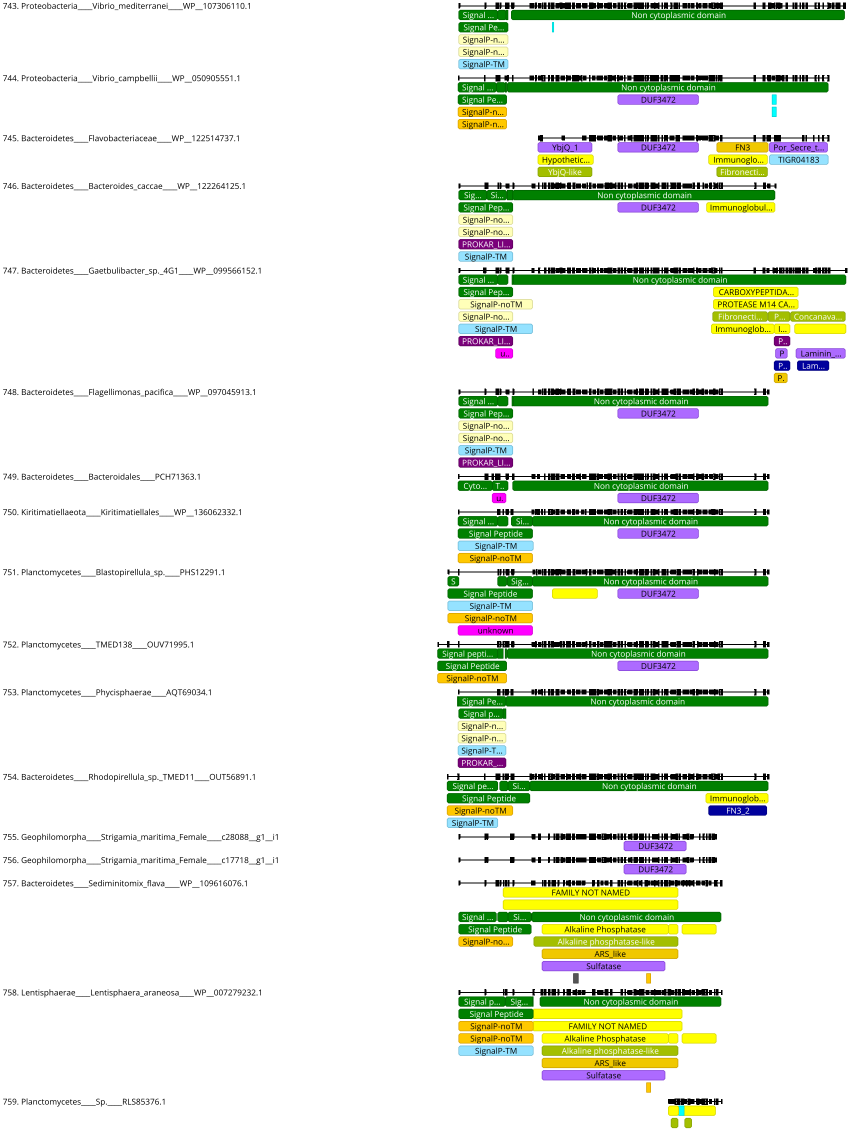
743. Proteobacteria___Vibrio_mediterranei___WP__107306110.1
Signal ...
Non cytoplasmic domain
Signal Pe...
SignalP-n...
SignalP-n...
SignalP-TM
744. Proteobacteria___Vibrio_campbellii___WP__050905551.1
Signal ...
Non cytoplasmic domain
Signal Pe...
DUF3472
SignalP-n...
SignalP-n...
745. Bacteroidetes___Flavobacteriaceae___WP__122514737.1
YbjQ_1
DUF3472
FN3
Por_Secre_t...
Hypothetic...
Immunoglo...
TIGR04183
YbjQ-like
Fibronecti...
746. Bacteroidetes___Bacteroides_caccae___WP__122264125.1
Sig...
Si...
Non cytoplasmic domain
Signal Pep...
DUF3472
Immunoglobul...
SignalP-no...
SignalP-no...
PROKAR_LI...
SignalP-TM
747. Bacteroidetes___Gaetbulibacter_sp._4G1___WP__099566152.1
Signal ...
Non cytoplasmic domain
Signal Pep...
CARBOXYPEPTIDA...
SignalP-noTM
PROTEASE M14 CA...
SignalP-no...
Fibronecti...
P...
Concanava...
SignalP-TM
Immunoglob...
I...
PROKAR_LI...
P..
u..
P
Laminin_...
P..
Lam...
P.
748. Bacteroidetes___Flagellimonas_pacifica___WP__097045913.1
Signal ...
Non cytoplasmic domain
Signal Pep...
DUF3472
SignalP-no...
SignalP-no...
SignalP-TM
PROKAR_LI...
749. Bacteroidetes___Bacteroidales___PCH71363.1
Cyto...
T...
Non cytoplasmic domain
u..
DUF3472
750. Kiritimatiellaeota___Kiritimatiellales___WP__136062332.1
Signal ...
Si...
Non cytoplasmic domain
Signal Peptide
DUF3472
SignalP-TM
SignalP-noTM
751. Planctomycetes___Blastopirellula_sp.___PHS12291.1
S
Sig...
Non cytoplasmic domain
Signal Peptide
DUF3472
SignalP-TM
SignalP-noTM
unknown
752. Planctomycetes___TMED138___OUV71995.1
Signal pepti...
Non cytoplasmic domain
Signal Peptide
DUF3472
SignalP-noTM
753. Planctomycetes___Phycisphaerae___AQT69034.1
Signal Pe...
Non cytoplasmic domain
Signal p...
SignalP-n...
SignalP-n...
SignalP-T...
PROKAR_...
754. Bacteroidetes___Rhodopirellula_sp._TMED11___OUT56891.1
Signal pe...
Si...
Non cytoplasmic domain
Signal Peptide
Immunoglob...
SignalP-noTM
FN3_2
SignalP-TM
755. Geophilomorpha___Strigamia_maritima_Female___c28088__g1__i1
DUF3472
756. Geophilomorpha___Strigamia_maritima_Female___c17718__g1__i1
DUF3472
757. Bacteroidetes___Sediminitomix_flava___WP__109616076.1
FAMILY NOT NAMED
Signal ...
Si...
Non cytoplasmic domain
Signal Peptide
Alkaline Phosphatase
SignalP-no...
Alkaline phosphatase-like
ARS_like
Sulfatase
758. Lentisphaerae___Lentisphaera_araneosa___WP__007279232.1
Signal p...
Sig...
Non cytoplasmic domain
Signal Peptide
SignalP-noTM
FAMILY NOT NAMED
SignalP-noTM
Alkaline Phosphatase
SignalP-TM
Alkaline phosphatase-like
ARS_like
Sulfatase
759. Planctomycetes___Sp.___RLS85376.1

760. Planctomycetes____Rubinisphaera_brasiliensis____WP__013628018.1

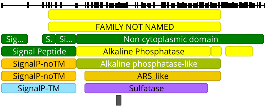

761. Bacteroidetes____Flavobacteriaceae_TMED147____RPG69513.1

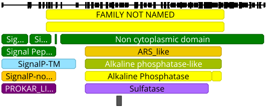

762. Bacteroidetes____Sp.____PWH11469.1

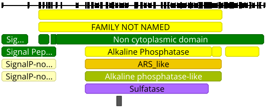

763. Bacteroidetes____Maribacter_sedimenticola____WP__089259898.1

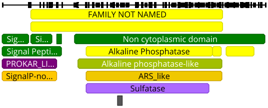

764. Bacteroidetes____Maribacter_dokdonensis____WP__074674387.1

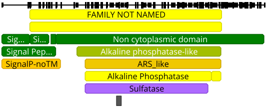

765. Bacteroidetes____Zobellia_uliginosa____WP__051914805.1

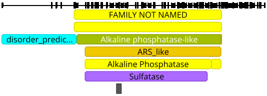

766. Bacteroidetes____Flavobacteriaceae_bacterium____PKA99785.1

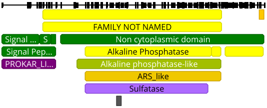

767. Bacteroidetes____Arenibacter_palladensis____SHG03679.1

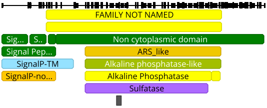

768. Bacteroidetes____Tangfeifania_diversioriginum____SHI79143.1

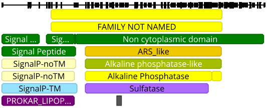

769. Bacteroidetes____Cyclobacterium_lianum____WP__084097340.1

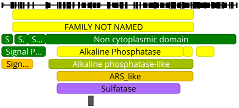

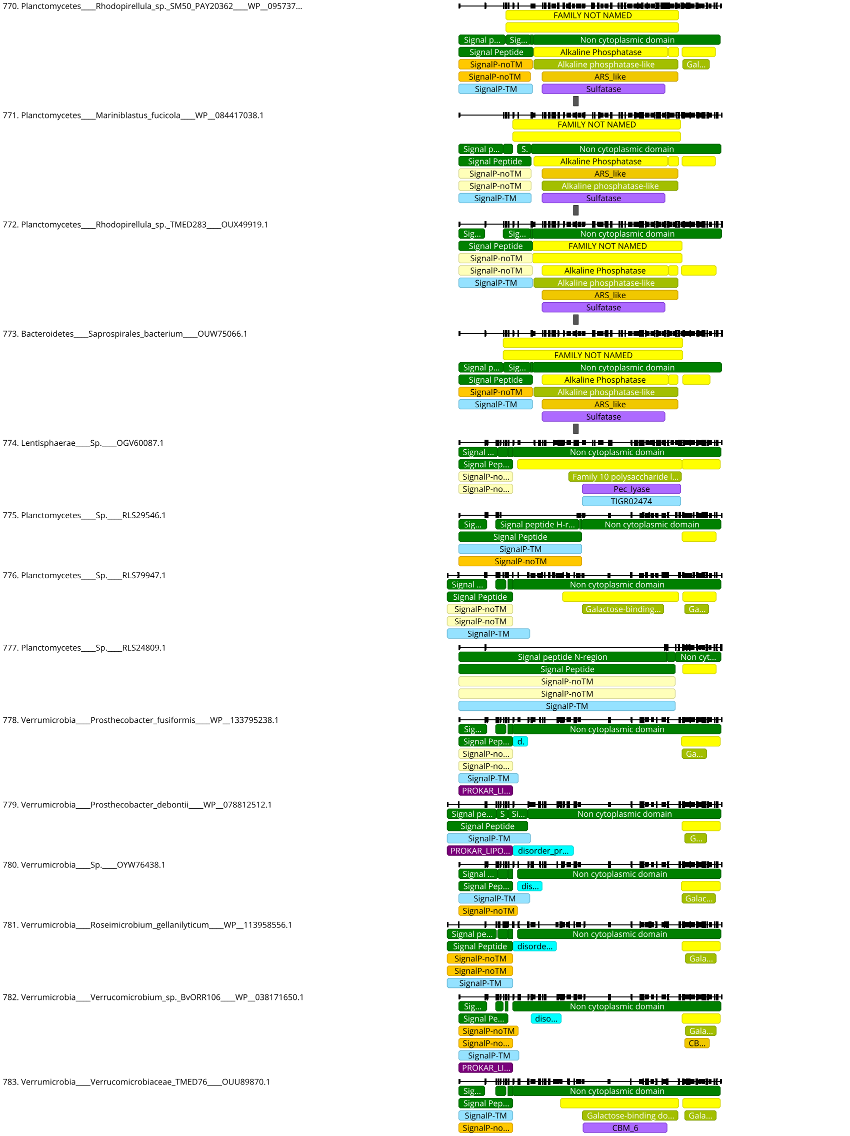
770. Planctomycetes___Rhodopirellula_sp._SM50_PAY20362___WP__095737...
FAMILY NOT NAMED
Signal p...
Sig...
Non cytoplasmic domain
Signal Peptide
Alkaline Phosphatase
SignalP-noTM
Alkaline phosphatase-like
Gal...
SignalP-noTM
ARS_like
SignalP-TM
Sulfatase
771. Planctomycetes___Mariniblastus_fucicola___WP__084417038.1
FAMILY NOT NAMED
Signal p...
S.
Non cytoplasmic domain
Signal Peptide
Alkaline Phosphatase
SignalP-noTM
ARS_like
SignalP-noTM
Alkaline phosphatase-like
SignalP-TM
Sulfatase
772. Planctomycetes___Rhodopirellula_sp._TMED283___OUX49919.1
Sig...
Sig...
Non cytoplasmic domain
Signal Peptide
FAMILY NOT NAMED
SignalP-noTM
SignalP-noTM
Alkaline Phosphatase
SignalP-TM
Alkaline phosphatase-like
ARS_like
Sulfatase
773. Bacteroidetes___Saprospirales_bacterium___OUW75066.1
FAMILY NOT NAMED
Signal p...
Sig...
Non cytoplasmic domain
Signal Peptide
Alkaline Phosphatase
SignalP-noTM
Alkaline phosphatase-like
SignalP-TM
ARS_like
Sulfatase
774. Lentisphaerae___Sp.___OGV60087.1
Signal ...
Non cytoplasmic domain
Signal Pep...
SignalP-no...
Family 10 polysaccharide l...
SignalP-no...
Pec_lyase
TIGR02474
775. Planctomycetes___Sp.___RLS29546.1
Sig...
Signal peptide H-r...
Non cytoplasmic domain
Signal Peptide
SignalP-TM
SignalP-noTM
776. Planctomycetes___Sp.___RLS79947.1
Signal ...
Non cytoplasmic domain
Signal Peptide
SignalP-noTM
Galactose-binding...
Ga...
SignalP-noTM
SignalP-TM
777. Planctomycetes___Sp.___RLS24809.1
Signal peptide N-region
Non cyt...
Signal Peptide
SignalP-noTM
SignalP-noTM
SignalP-TM
778. Verrumicrobia___Prosthecobacter_fusiformis___WP__133795238.1
Sig...
Non cytoplasmic domain
Signal Pep...
d.
SignalP-no...
Ga...
SignalP-no...
SignalP-TM
PROKAR_LI...
779. Verrumicrobia___Prosthecobacter_debontii___WP__078812512.1
Signal pe...
S
Si...
Non cytoplasmic domain
Signal Peptide
SignalP-TM
G...
PROKAR_LIPO...
disorder_pr...
780. Verrumicrobia___Sp.___OYW76438.1
Signal ...
Non cytoplasmic domain
Signal Pep...
dis...
SignalP-TM
Galac...
SignalP-noTM
781. Verrumicrobia___Roseimicrobium_gellanilyticum___WP__113958556.1
Signal pe...
Non cytoplasmic domain
Signal Peptide
disorde...
SignalP-noTM
Gala...
SignalP-noTM
SignalP-TM
782. Verrumicrobia___Verrucomicrobium_sp._BvORR106___WP__038171650.1
Sig...
Non cytoplasmic domain
Signal Pe...
diso...
SignalP-noTM
Gala...
SignalP-no...
CB...
SignalP-TM
PROKAR_LI...
783. Verrumicrobia___Verrucomicrobiaceae_TMED76___OUU89870.1
Sig...
Non cytoplasmic domain
Signal Pep...
SignalP-TM
Galactose-binding do...
Gala...
SignalP-no...
CBM_6

784. Bacteroidetes___Sphingobacterium_sp._B29___WP__083663536.1

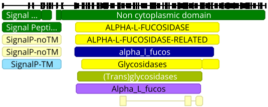

785. Bacteroidetes___Pedobacter_caeni___WP__073228603.1

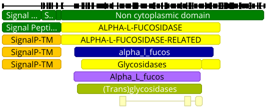

786. Bacteroidetes___Sphingobacterium_psychroaquaticum___WP__085472...

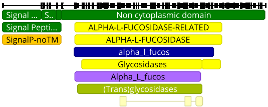

787. Bacteroidetes___Pedobacter_sp._YR016___WP__108204548.1

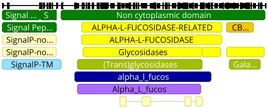

788. Firmicutes___Paenibacillus_ginsengarvi___WP__120751916.1

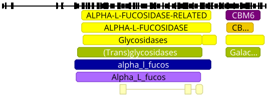

789. Armatimonadetes___Sp.___KXK37236.1

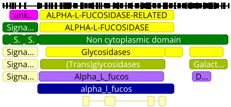

790. Armatimonadetes___Sp.___RIJ99962.1

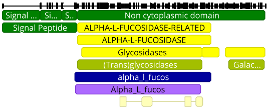

791. Armatimonadetes___Fimbriimonas_ginsengisoli___WP__025225458.1

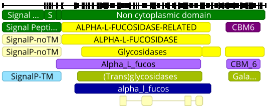

792. Bacterium___Sp.___RYG26229.1

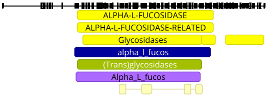

793. Armatimonadetes___Sp.___OJU61908.1

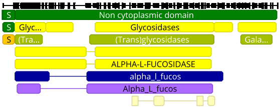

794. Armatimonadetes___Sp.___OWU65232.1

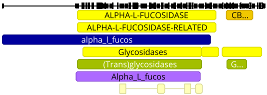

795. Verrumicrobia___Sp.___RME89401.1

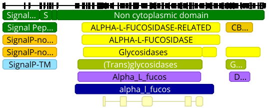

796. Desantisbacteria___Sp.___PIV55928.1

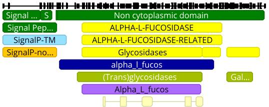

797. Verrumicrobia___Sp.___OQW96982.1

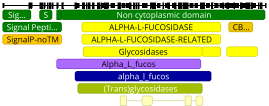

798. Bacteroidetes___Pseudoflavitalea_sp._5GH32-13___WP__119049835.1

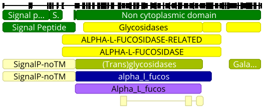

799. Armatimonadetes___Sp.___OJU62946.1

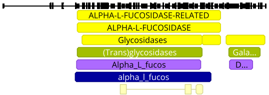

800. Armatimonadetes___Sp.___OIP03115.1

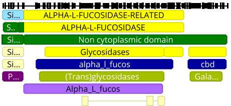

801. Bacteroidetes___Saccharicrinis_fermentans___WP__081735867.1

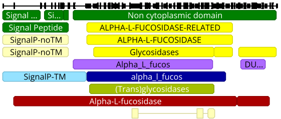

802. Bacteroidetes___Algibacter_alginicilyticus___WP__098946181.1

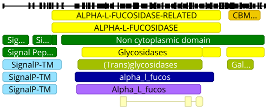

803. Bacteroidetes___Arcticibacter_svalbardensis___WP__052017030.1

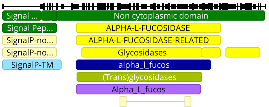

804. Planctomycetes___Sp.___RMG01079.1

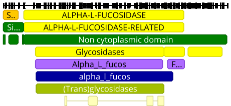

805. Lentisphaerae___Sp.___OGV66970.1

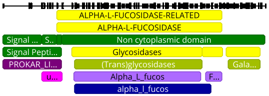

806. Verrumicrobia___Sp.___TAE90453.1

807. Verrumicrobia___Sp.___TAE89078.1

808. Armatimonadetes___Capsulimonas_corticalis___WP__119321738.1

809. Bacterium___Sp.___RYG36185.1

810. Armatimonadetes___Sp.___OJU62848.1

811. Verrucomicrobia___Sp.___TAE91276.1

812. Verrucomicrobia___Sp.___TAE74425.1

813. Lentisphaerae___Lentisphaera_araneosa___WP__007279467.1

814. Lentisphaerae___Lentisphaera_araneosa___WP__007278952.1

815. Bacterium___Sp.___WP__120394777.1

816. Bacteroidetes___Sp.___KXK19710.1

817. Verrumicrobia___Verrucomicrobiaceae___RZO16897.1

818. Bacteroidetes___Bacteroides_cellulosilyticus___WP__129616490.1

819. Acidobacteria___Sp.___RPJ59100.1

820. Acidobacteria___Sp.___RPI26164.1

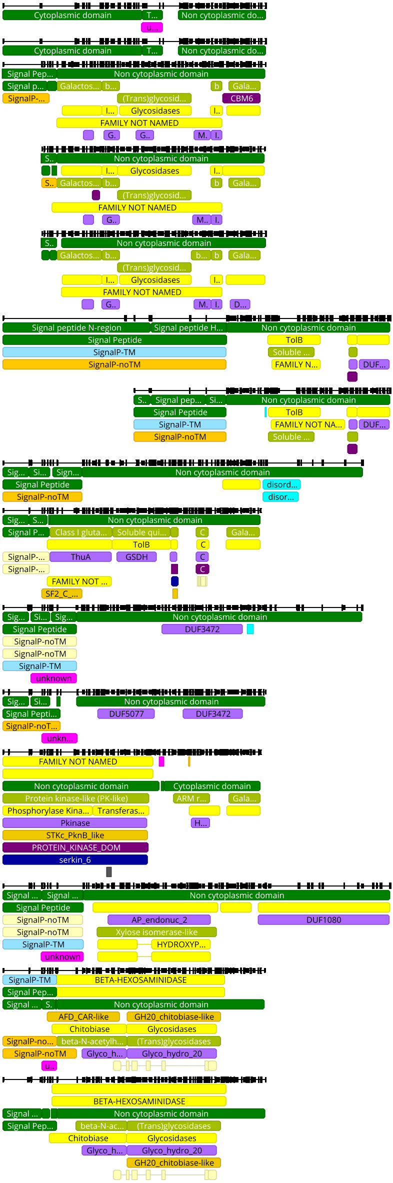

Supplement: Supplementary file 12 — Supplementary Data 8 [file DUF3472.pdf › Supplementary Data 8/DUF3472.pdf]
