# Supplementary material for: Phylogenetic analyses suggest centipede venom arsenals were repeatedly stocked by horizontal gene transfer
Source: Nat Commun. 2021 Feb 5;12:818. doi: 10.1038/s41467-021-21093-8 (PMC7864903; doi:10.1038/s41467-021-21093-8)

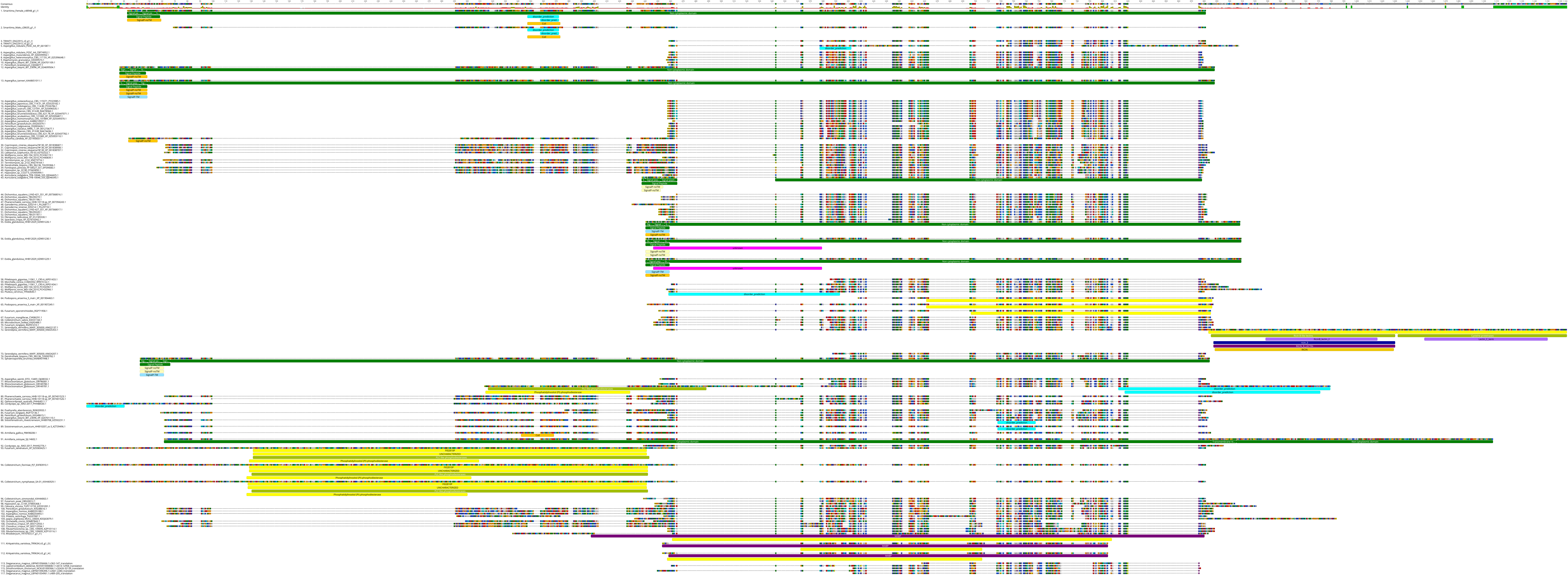

Supplement: Supplementary file 12 — Supplementary Data 8 [file unchar05.pdf › Supplementary Data 8/unchar05.pdf]
