# Supplementary material for: Phylogenetic analyses suggest centipede venom arsenals were repeatedly stocked by horizontal gene transfer
Source: Nat Commun. 2021 Feb 5;12:818. doi: 10.1038/s41467-021-21093-8 (PMC7864903; doi:10.1038/s41467-021-21093-8)
Supplement: Supplementary file 12 — Supplementary Data 8 [file BPftx.pdf › Supplementary Data 8/BPftx.pdf]

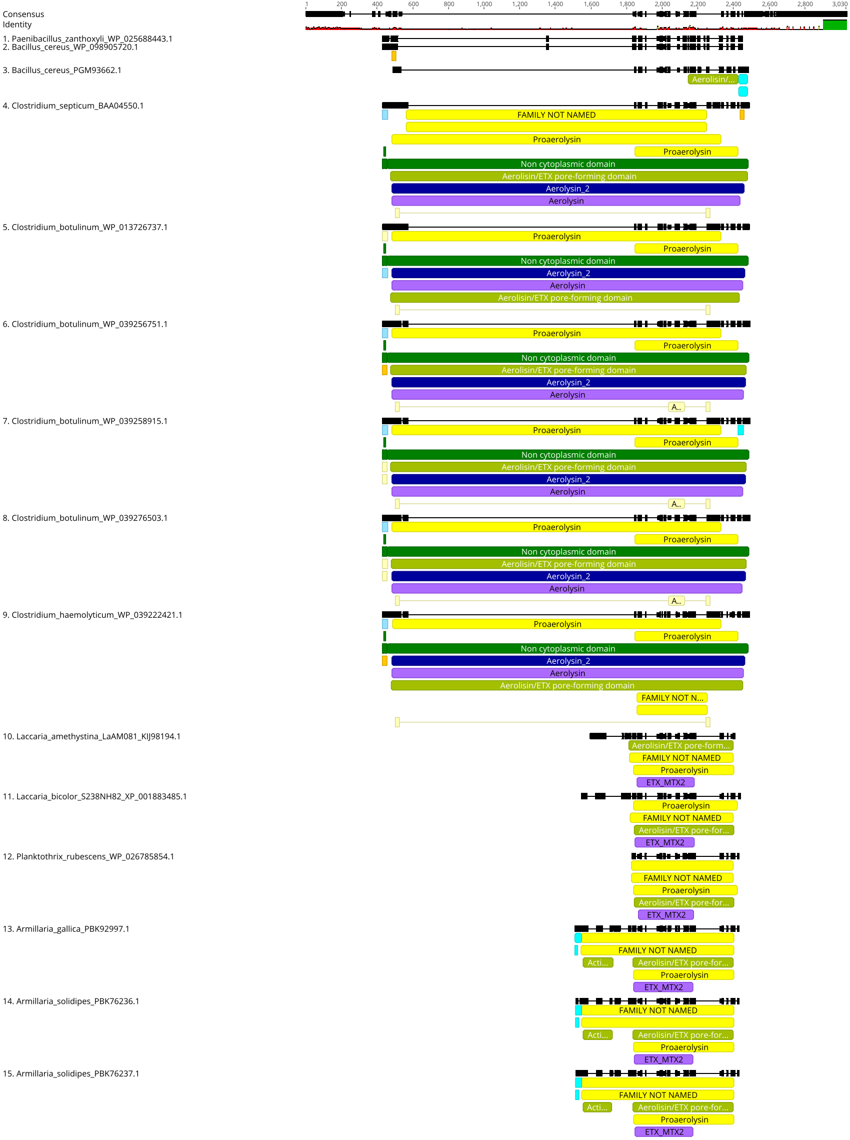

Consensus
Identity
1. Paenibacillus_zanthoxyli_WP_025688443.1
2. Bacillus_cereus_WP_098905720.1
3. Bacillus_cereus_PGM93662.1
Aerolisin/...
4. Clostridium_septicum_BAA04550.1
FAMILY NOT NAMED
Proaerolysin
Proaerolysin
Non cytoplasmic domain
Aerolisin/ETX pore-forming domain
Aerolysin_2
Aerolysin
5. Clostridium_botulinum_WP_013726737.1
Proaerolysin
Proaerolysin
Non cytoplasmic domain
Aerolysin_2
Aerolysin
Aerolisin/ETX pore-forming domain
6. Clostridium_botulinum_WP_039256751.1
Proaerolysin
Proaerolysin
Non cytoplasmic domain
Aerolisin/ETX pore-forming domain
Aerolysin_2
Aerolysin
A..
7. Clostridium_botulinum_WP_039258915.1
Proaerolysin
Proaerolysin
Non cytoplasmic domain
Aerolisin/ETX pore-forming domain
Aerolysin_2
Aerolysin
A..
8. Clostridium_botulinum_WP_039276503.1
Proaerolysin
Proaerolysin
Non cytoplasmic domain
Aerolisin/ETX pore-forming domain
Aerolysin_2
Aerolysin
A..
9. Clostridium_haemolyticum_WP_039222421.1
Proaerolysin
Proaerolysin
Non cytoplasmic domain
Aerolysin_2
Aerolysin
Aerolisin/ETX pore-forming domain
FAMILY NOT N...
10. Laccaria_amethystina_LaAM081_KIJ98194.1
Aerolisin/ETX pore-form...
FAMILY NOT NAMED
Proaerolysin
ETX_MTX2
11. Laccaria_bicolor_S238NH82_XP_001883485.1
Proaerolysin
FAMILY NOT NAMED
Aerolisin/ETX pore-for...
ETX_MTX2
12. Planktothrix_rubescens_WP_026785854.1
FAMILY NOT NAMED
Proaerolysin
Aerolisin/ETX pore-for...
ETX_MTX2
13. Armillaria_gallica_PBK92997.1
FAMILY NOT NAMED
Acti...
Aerolisin/ETX pore-for...
Proaerolysin
ETX_MTX2
14. Armillaria_solidipes_PBK76236.1
FAMILY NOT NAMED
Acti...
Aerolisin/ETX pore-for...
Proaerolysin
ETX_MTX2
15. Armillaria_solidipes_PBK76237.1
FAMILY NOT NAMED
Acti...
Aerolisin/ETX pore-for...
Proaerolysin
ETX_MTX2

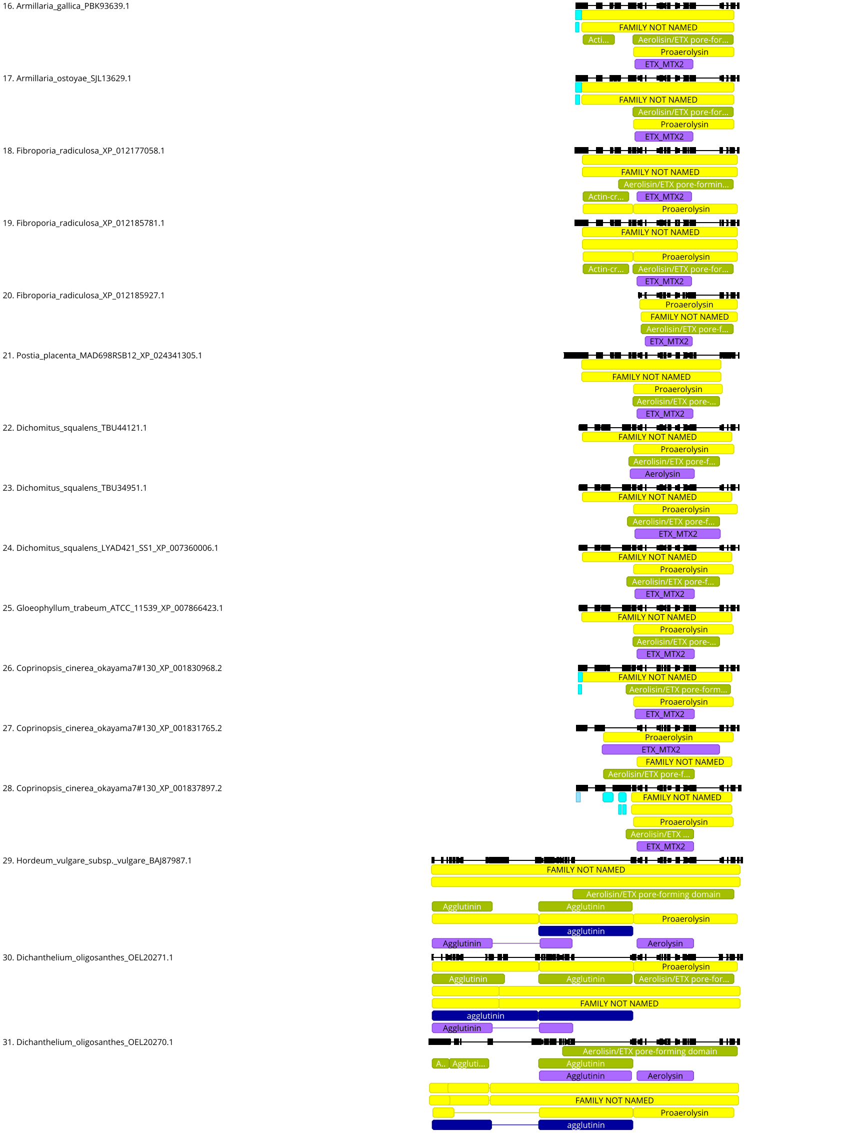

16. Armillaria_gallica_PBK93639.1
FAMILY NOT NAMED
Acti...
Aerolisin/ETX pore-for...
Proaerolysin
ETX_MTX2
17. Armillaria_ostoyae_SJL13629.1
FAMILY NOT NAMED
Aerolisin/ETX pore-for...
Proaerolysin
ETX_MTX2
18. Fibroporia_radiculosa_XP_012177058.1
FAMILY NOT NAMED
Aerolisin/ETX pore-formin...
Actin-cr...
ETX_MTX2
Proaerolysin
19. Fibroporia_radiculosa_XP_012185781.1
FAMILY NOT NAMED
Proaerolysin
Actin-cr...
Aerolisin/ETX pore-for...
ETX_MTX2
20. Fibroporia_radiculosa_XP_012185927.1
Proaerolysin
FAMILY NOT NAMED
Aerolisin/ETX pore-f...
ETX_MTX2
21. Postia_placenta_MAD698RSB12_XP_024341305.1
FAMILY NOT NAMED
Proaerolysin
Aerolisin/ETX pore-...
ETX_MTX2
22. Dichomitus_squalens_TBU44121.1
FAMILY NOT NAMED
Proaerolysin
Aerolisin/ETX pore-f...
Aerolysin
23. Dichomitus_squalens_TBU34951.1
FAMILY NOT NAMED
Proaerolysin
Aerolisin/ETX pore-f...
ETX_MTX2
24. Dichomitus_squalens_LYAD421_SS1_XP_007360006.1
FAMILY NOT NAMED
Proaerolysin
Aerolisin/ETX pore-f...
ETX_MTX2
25. Gloeophyllum_trabeum_ATCC_11539_XP_007866423.1
FAMILY NOT NAMED
Proaerolysin
Aerolisin/ETX pore-...
ETX_MTX2
26. Coprinopsis_cinerea_okayama7#130_XP_001830968.2
FAMILY NOT NAMED
Aerolisin/ETX pore-form...
Proaerolysin
ETX_MTX2
27. Coprinopsis_cinerea_okayama7#130_XP_001831765.2
Proaerolysin
ETX_MTX2
FAMILY NOT NAMED
Aerolisin/ETX pore-f...
28. Coprinopsis_cinerea_okayama7#130_XP_001837897.2
FAMILY NOT NAMED
Proaerolysin
Aerolisin/ETX ...
ETX_MTX2
29. Hordeum_vulgare_subsp._vulgare_BAJ87987.1
FAMILY NOT NAMED
Aerolisin/ETX pore-forming domain
Agglutinin
Agglutinin
Proaerolysin
agglutinin
Agglutinin
Aerolysin
30. Dichanthelium_oligosanthes_OEL20271.1
Proaerolysin
Agglutinin
Agglutinin
Aerolisin/ETX pore-for...
FAMILY NOT NAMED
agglutinin
Agglutinin
31. Dichanthelium_oligosanthes_OEL20270.1
Aerolisin/ETX pore-forming domain
A..
Aggluti...
Agglutinin
Agglutinin
Aerolysin
FAMILY NOT NAMED
Proaerolysin
agglutinin

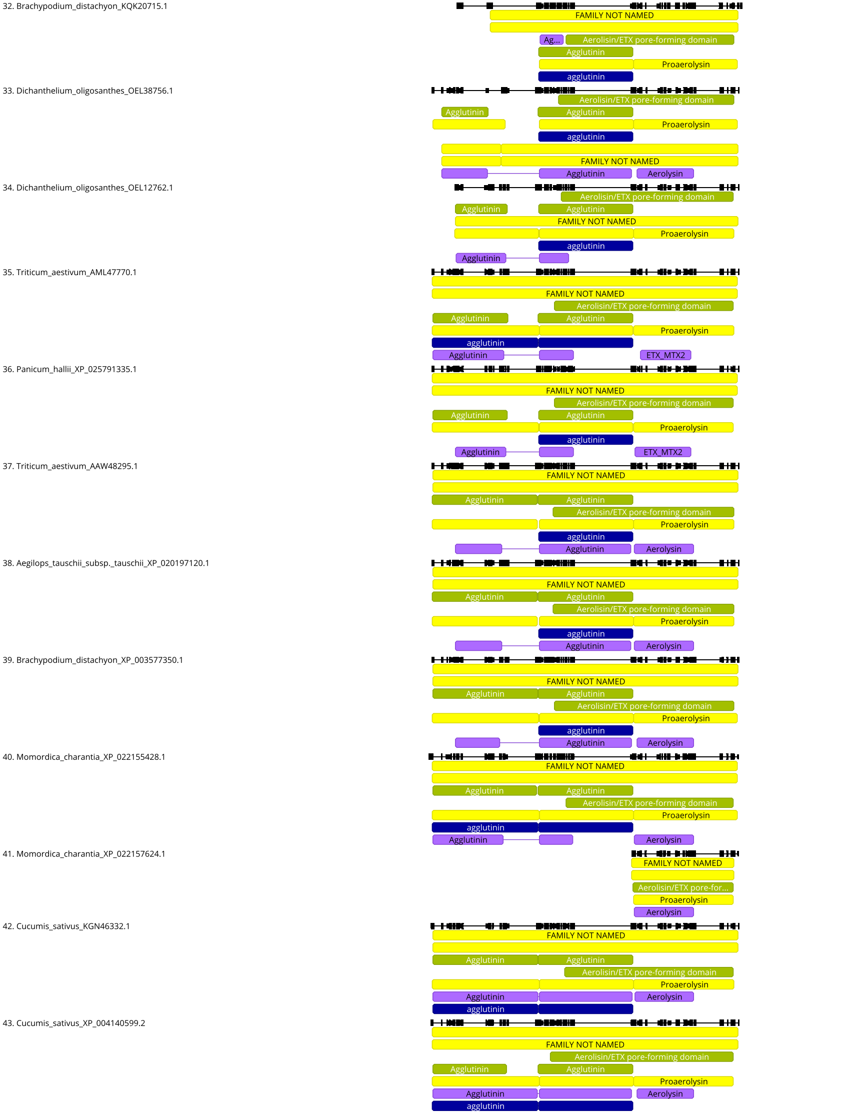
32. Brachypodium_distachyon_KQK20715.1
FAMILY NOT NAMED
Ag...
Aerolisin/ETX pore-forming domain
Agglutinin
Proaerolysin
agglutinin
33. Dichanthelium_oligosanthes_OEL38756.1
Aerolisin/ETX pore-forming domain
Agglutinin
Agglutinin
Proaerolysin
agglutinin
FAMILY NOT NAMED
Agglutinin
Aerolysin
34. Dichanthelium_oligosanthes_OEL12762.1
Aerolisin/ETX pore-forming domain
Agglutinin
Agglutinin
FAMILY NOT NAMED
Proaerolysin
agglutinin
Agglutinin
35. Triticum_aestivum_AML47770.1
FAMILY NOT NAMED
Aerolisin/ETX pore-forming domain
Agglutinin
Agglutinin
Proaerolysin
agglutinin
Agglutinin
ETX_MTX2
36. Panicum_hallii_XP_025791335.1
FAMILY NOT NAMED
Aerolisin/ETX pore-forming domain
Agglutinin
Agglutinin
Proaerolysin
agglutinin
Agglutinin
ETX_MTX2
37. Triticum_aestivum_AAW48295.1
FAMILY NOT NAMED
Agglutinin
Agglutinin
Aerolisin/ETX pore-forming domain
Proaerolysin
agglutinin
Agglutinin
Aerolysin
38. Aegilops_tauschii_subsp._tauschii_XP_020197120.1
FAMILY NOT NAMED
Agglutinin
Agglutinin
Aerolisin/ETX pore-forming domain
Proaerolysin
agglutinin
Agglutinin
Aerolysin
39. Brachypodium_distachyon_XP_003577350.1
FAMILY NOT NAMED
Agglutinin
Agglutinin
Aerolisin/ETX pore-forming domain
Proaerolysin
agglutinin
Agglutinin
Aerolysin
40. Momordica_charantia_XP_022155428.1
FAMILY NOT NAMED
Agglutinin
Agglutinin
Aerolisin/ETX pore-forming domain
Proaerolysin
agglutinin
Agglutinin
Aerolysin
41. Momordica_charantia_XP_022157624.1
FAMILY NOT NAMED
Aerolisin/ETX pore-for...
Proaerolysin
Aerolysin
42. Cucumis_sativus_KGN46332.1
FAMILY NOT NAMED
Agglutinin
Agglutinin
Aerolisin/ETX pore-forming domain
Proaerolysin
Agglutinin
Aerolysin
agglutinin
43. Cucumis_sativus_XP_004140599.2
FAMILY NOT NAMED
Aerolisin/ETX pore-forming domain
Agglutinin
Agglutinin
Proaerolysin
Agglutinin
Aerolysin
agglutinin

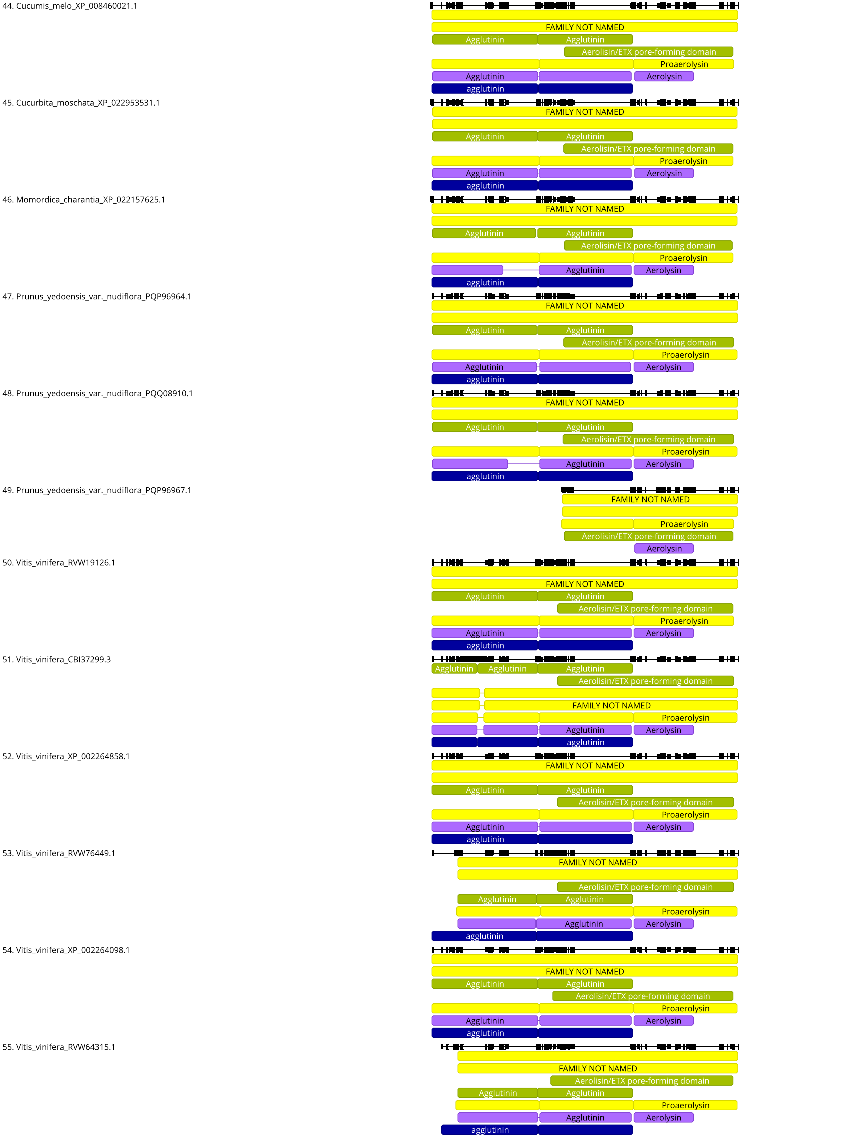
44. Cucumis_melo_XP_008460021.1
FAMILY NOT NAMED
Agglutinin
Agglutinin
Aerolisin/ETX pore-forming domain
Proaerolysin
Agglutinin
Aerolysin
agglutinin
45. Cucurbita_moschata_XP_022953531.1
FAMILY NOT NAMED
Agglutinin
Agglutinin
Aerolisin/ETX pore-forming domain
Proaerolysin
Agglutinin
Aerolysin
agglutinin
46. Momordica_charantia_XP_022157625.1
FAMILY NOT NAMED
Agglutinin
Agglutinin
Aerolisin/ETX pore-forming domain
Proaerolysin
Agglutinin
Aerolysin
agglutinin
47. Prunus_yedoensis_var._nudiflora_PQP96964.1
FAMILY NOT NAMED
Agglutinin
Agglutinin
Aerolisin/ETX pore-forming domain
Proaerolysin
Agglutinin
Aerolysin
agglutinin
48. Prunus_yedoensis_var._nudiflora_PQQ08910.1
FAMILY NOT NAMED
Agglutinin
Agglutinin
Aerolisin/ETX pore-forming domain
Proaerolysin
Agglutinin
Aerolysin
agglutinin
49. Prunus_yedoensis_var._nudiflora_PQP96967.1
FAMILY NOT NAMED
Proaerolysin
Aerolisin/ETX pore-forming domain
Aerolysin
50. Vitis_vinifera_RVW19126.1
FAMILY NOT NAMED
Agglutinin
Agglutinin
Aerolisin/ETX pore-forming domain
Proaerolysin
Agglutinin
Aerolysin
agglutinin
51. Vitis_vinifera_CBI37299.3
Agglutinin
Agglutinin
Agglutinin
Aerolisin/ETX pore-forming domain
FAMILY NOT NAMED
Proaerolysin
Agglutinin
Aerolysin
agglutinin
52. Vitis_vinifera_XP_002264858.1
FAMILY NOT NAMED
Agglutinin
Agglutinin
Aerolisin/ETX pore-forming domain
Proaerolysin
Agglutinin
Aerolysin
agglutinin
53. Vitis_vinifera_RVW76449.1
FAMILY NOT NAMED
Aerolisin/ETX pore-forming domain
Agglutinin
Agglutinin
Proaerolysin
Agglutinin
Aerolysin
agglutinin
54. Vitis_vinifera_XP_002264098.1
FAMILY NOT NAMED
Agglutinin
Agglutinin
Aerolisin/ETX pore-forming domain
Proaerolysin
Agglutinin
Aerolysin
agglutinin
55. Vitis_vinifera_RVW64315.1
FAMILY NOT NAMED
Aerolisin/ETX pore-forming domain
Agglutinin
Agglutinin
Proaerolysin
Agglutinin
Aerolysin
agglutinin

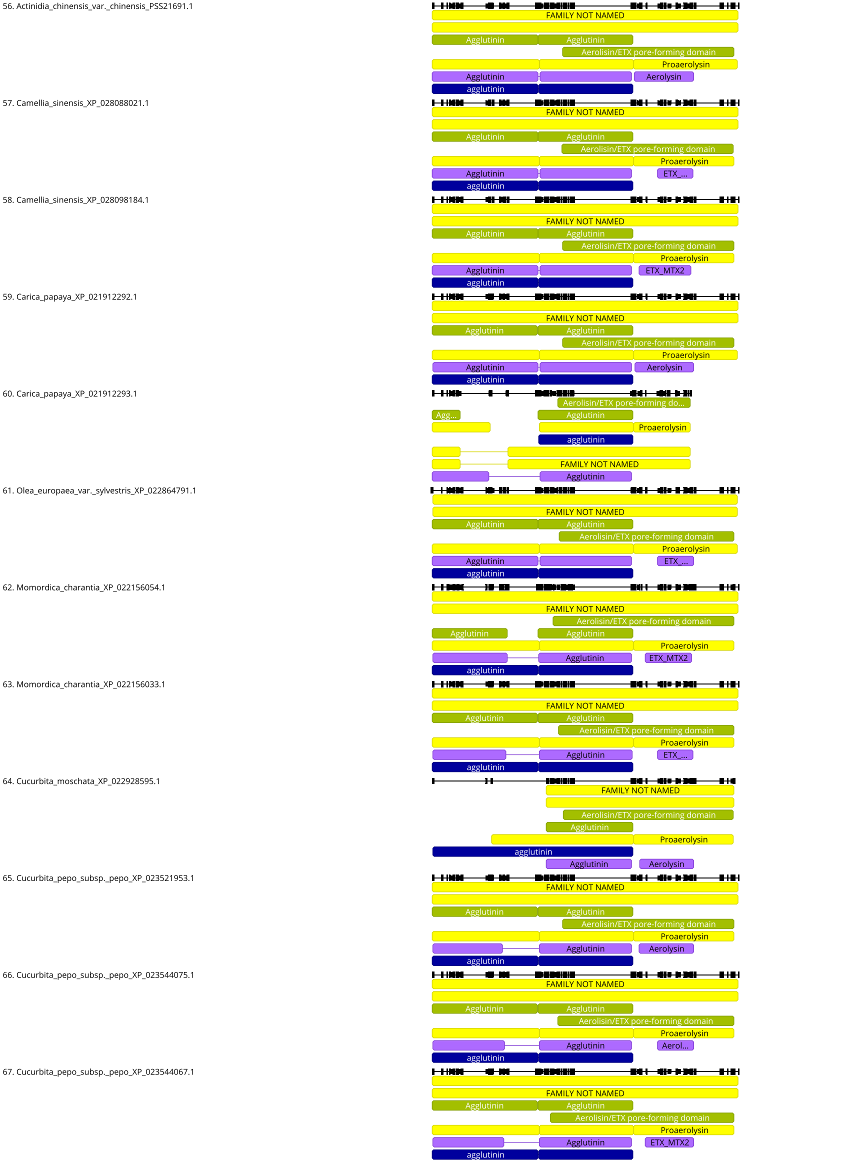
56. Actinidia_chinensis_var._chinensis_PSS21691.1
FAMILY NOT NAMED
Agglutinin
Agglutinin
Aerolisin/ETX pore-forming domain
Proaerolysin
Agglutinin
Aerolysin
agglutinin
57. Camellia_sinensis_XP_028088021.1
FAMILY NOT NAMED
Agglutinin
Agglutinin
Aerolisin/ETX pore-forming domain
Proaerolysin
Agglutinin
ETX_...
agglutinin
58. Camellia_sinensis_XP_028098184.1
FAMILY NOT NAMED
Agglutinin
Agglutinin
Aerolisin/ETX pore-forming domain
Proaerolysin
Agglutinin
ETX_MTX2
agglutinin
59. Carica_papaya_XP_021912292.1
FAMILY NOT NAMED
Agglutinin
Agglutinin
Aerolisin/ETX pore-forming domain
Proaerolysin
Agglutinin
Aerolysin
agglutinin
60. Carica_papaya_XP_021912293.1
Aerolisin/ETX pore-forming do...
Agg...
Agglutinin
Proaerolysin
agglutinin
FAMILY NOT NAMED
Agglutinin
61. Olea_europaea_var._sylvestris_XP_022864791.1
FAMILY NOT NAMED
Agglutinin
Agglutinin
Aerolisin/ETX pore-forming domain
Proaerolysin
Agglutinin
ETX_...
agglutinin
62. Momordica_charantia_XP_022156054.1
FAMILY NOT NAMED
Aerolisin/ETX pore-forming domain
Agglutinin
Agglutinin
Proaerolysin
Agglutinin
ETX_MTX2
agglutinin
63. Momordica_charantia_XP_022156033.1
FAMILY NOT NAMED
Agglutinin
Agglutinin
Aerolisin/ETX pore-forming domain
Proaerolysin
Agglutinin
ETX_...
agglutinin
64. Cucurbita_moschata_XP_022928595.1
FAMILY NOT NAMED
Aerolisin/ETX pore-forming domain
Agglutinin
Proaerolysin
agglutinin
Agglutinin
Aerolysin
65. Cucurbita_pepo_subsp._pepo_XP_023521953.1
FAMILY NOT NAMED
Agglutinin
Agglutinin
Aerolisin/ETX pore-forming domain
Proaerolysin
Agglutinin
Aerolysin
agglutinin
66. Cucurbita_pepo_subsp._pepo_XP_023544075.1
FAMILY NOT NAMED
Agglutinin
Agglutinin
Aerolisin/ETX pore-forming domain
Proaerolysin
Agglutinin
Aerol...
agglutinin
67. Cucurbita_pepo_subsp._pepo_XP_023544067.1
FAMILY NOT NAMED
Agglutinin
Agglutinin
Aerolisin/ETX pore-forming domain
Proaerolysin
Agglutinin
ETX_MTX2
agglutinin

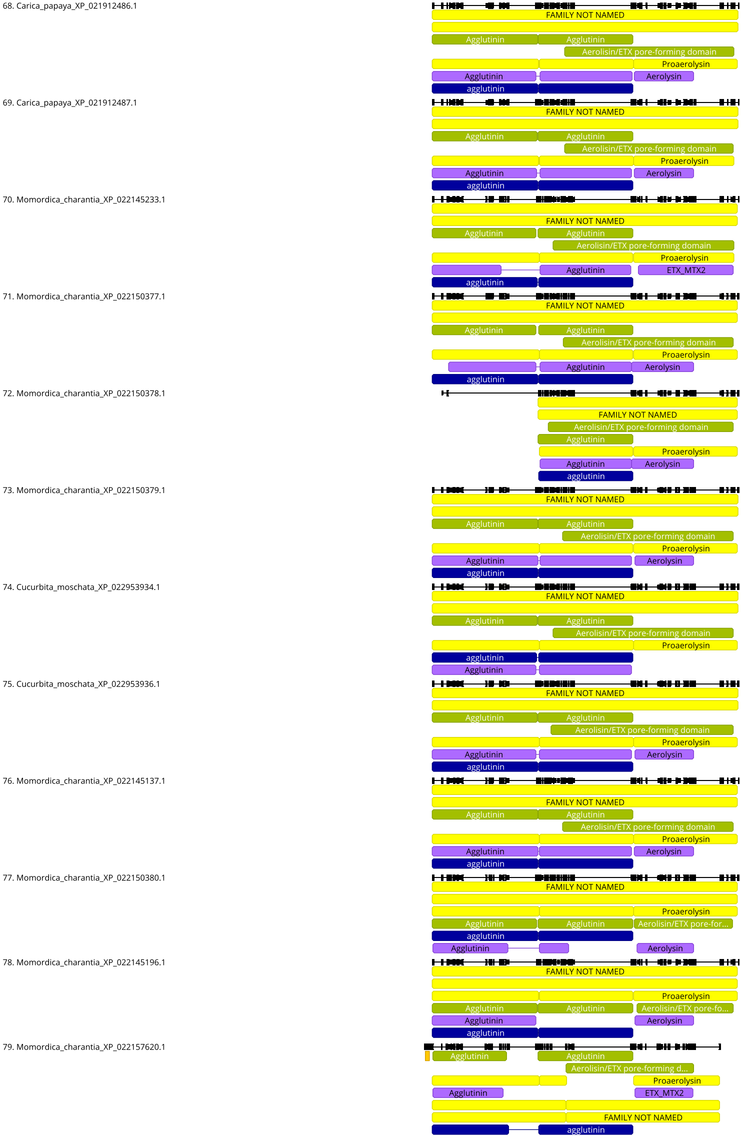
68. Carica_papaya_XP_021912486.1
FAMILY NOT NAMED
Agglutinin
Agglutinin
Aerolisin/ETX pore-forming domain
Proaerolysin
Agglutinin
Aerolysin
agglutinin
69. Carica_papaya_XP_021912487.1
FAMILY NOT NAMED
Agglutinin
Agglutinin
Aerolisin/ETX pore-forming domain
Proaerolysin
Agglutinin
Aerolysin
agglutinin
70. Momordica_charantia_XP_022145233.1
FAMILY NOT NAMED
Agglutinin
Agglutinin
Aerolisin/ETX pore-forming domain
Proaerolysin
Agglutinin
ETX_MTX2
agglutinin
71. Momordica_charantia_XP_022150377.1
FAMILY NOT NAMED
Agglutinin
Agglutinin
Aerolisin/ETX pore-forming domain
Proaerolysin
Agglutinin
Aerolysin
agglutinin
72. Momordica_charantia_XP_022150378.1
FAMILY NOT NAMED
Aerolisin/ETX pore-forming domain
Agglutinin
Proaerolysin
Agglutinin
Aerolysin
agglutinin
73. Momordica_charantia_XP_022150379.1
FAMILY NOT NAMED
Agglutinin
Agglutinin
Aerolisin/ETX pore-forming domain
Proaerolysin
Agglutinin
Aerolysin
agglutinin
74. Cucurbita_moschata_XP_022953934.1
FAMILY NOT NAMED
Agglutinin
Agglutinin
Aerolisin/ETX pore-forming domain
Proaerolysin
agglutinin
Agglutinin
75. Cucurbita_moschata_XP_022953936.1
FAMILY NOT NAMED
Agglutinin
Agglutinin
Aerolisin/ETX pore-forming domain
Proaerolysin
Agglutinin
Aerolysin
agglutinin
76. Momordica_charantia_XP_022145137.1
FAMILY NOT NAMED
Agglutinin
Agglutinin
Aerolisin/ETX pore-forming domain
Proaerolysin
Agglutinin
Aerolysin
agglutinin
77. Momordica_charantia_XP_022150380.1
FAMILY NOT NAMED
Proaerolysin
Agglutinin
Agglutinin
Aerolisin/ETX pore-for...
agglutinin
Agglutinin
Aerolysin
78. Momordica_charantia_XP_022145196.1
FAMILY NOT NAMED
Proaerolysin
Agglutinin
Agglutinin
Aerolisin/ETX pore-fo...
Agglutinin
Aerolysin
agglutinin
79. Momordica_charantia_XP_022157620.1
Agglutinin
Agglutinin
Aerolisin/ETX pore-forming d...
Proaerolysin
Agglutinin
ETX_MTX2
FAMILY NOT NAMED
agglutinin

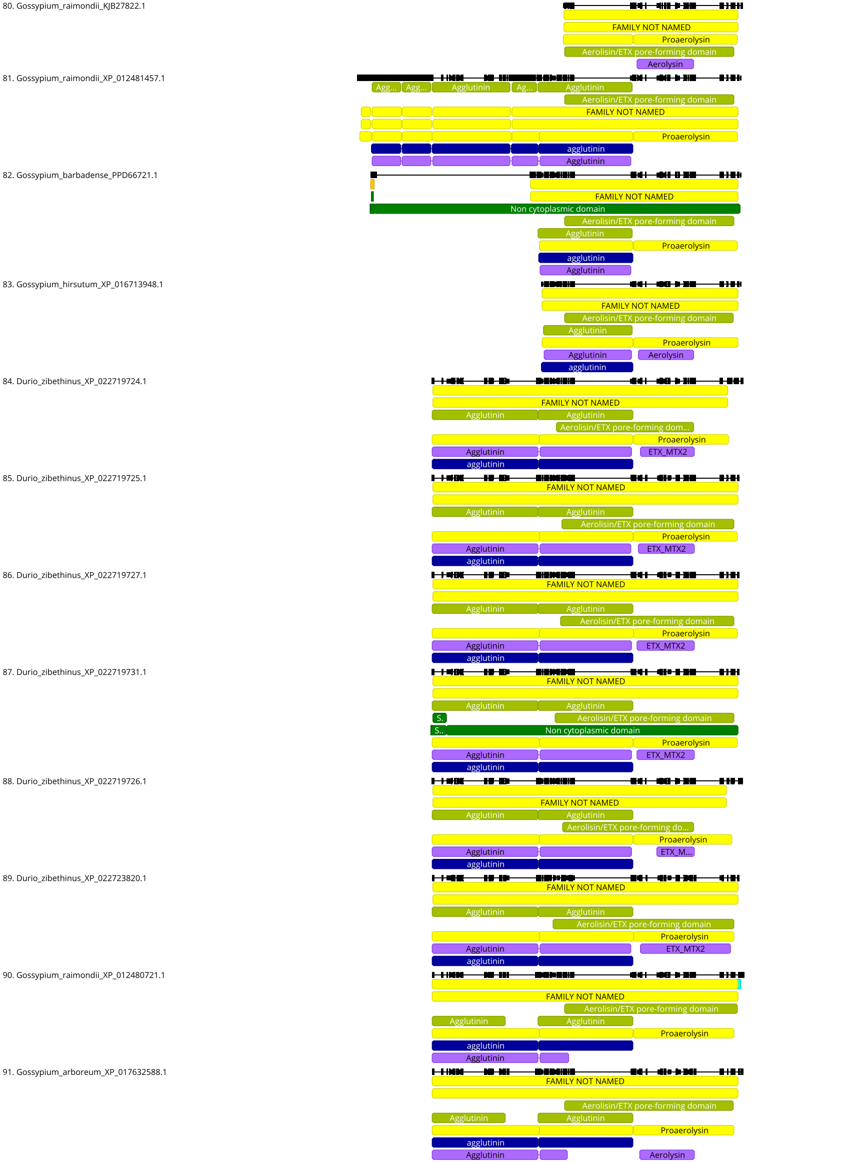
80. Gossypium_raimondii_KJB27822.1
FAMILY NOT NAMED
Proaerolysin
Aerolisin/ETX pore-forming domain
Aerolysin
81. Gossypium_raimondii_XP_012481457.1
Agg...
Agg...
Agglutinin
Ag...
Agglutinin
Aerolisin/ETX pore-forming domain
FAMILY NOT NAMED
Proaerolysin
agglutinin
Agglutinin
82. Gossypium_barbadense_PPD66721.1
FAMILY NOT NAMED
Non cytoplasmic domain
Aerolisin/ETX pore-forming domain
Agglutinin
Proaerolysin
agglutinin
Agglutinin
83. Gossypium_hirsutum_XP_016713948.1
FAMILY NOT NAMED
Aerolisin/ETX pore-forming domain
Agglutinin
Proaerolysin
Agglutinin
Aerolysin
agglutinin
84. Durio_zibethinus_XP_022719724.1
FAMILY NOT NAMED
Agglutinin
Agglutinin
Aerolisin/ETX pore-forming dom...
Proaerolysin
Agglutinin
ETX_MTX2
agglutinin
85. Durio_zibethinus_XP_022719725.1
FAMILY NOT NAMED
Agglutinin
Agglutinin
Aerolisin/ETX pore-forming domain
Proaerolysin
Agglutinin
ETX_MTX2
agglutinin
86. Durio_zibethinus_XP_022719727.1
FAMILY NOT NAMED
Agglutinin
Agglutinin
Aerolisin/ETX pore-forming domain
Proaerolysin
Agglutinin
ETX_MTX2
agglutinin
87. Durio_zibethinus_XP_022719731.1
FAMILY NOT NAMED
Agglutinin
Agglutinin
S.
Aerolisin/ETX pore-forming domain
S..
Non cytoplasmic domain
Proaerolysin
Agglutinin
ETX_MTX2
agglutinin
88. Durio_zibethinus_XP_022719726.1
FAMILY NOT NAMED
Agglutinin
Agglutinin
Aerolisin/ETX pore-forming do...
Proaerolysin
Agglutinin
ETX_M...
agglutinin
89. Durio_zibethinus_XP_022723820.1
FAMILY NOT NAMED
Agglutinin
Agglutinin
Aerolisin/ETX pore-forming domain
Proaerolysin
Agglutinin
ETX_MTX2
agglutinin
90. Gossypium_raimondii_XP_012480721.1
FAMILY NOT NAMED
Aerolisin/ETX pore-forming domain
Agglutinin
Agglutinin
Proaerolysin
agglutinin
Agglutinin
91. Gossypium_arboreum_XP_017632588.1
FAMILY NOT NAMED
Aerolisin/ETX pore-forming domain
Agglutinin
Agglutinin
Proaerolysin
agglutinin
Agglutinin
Aerolysin

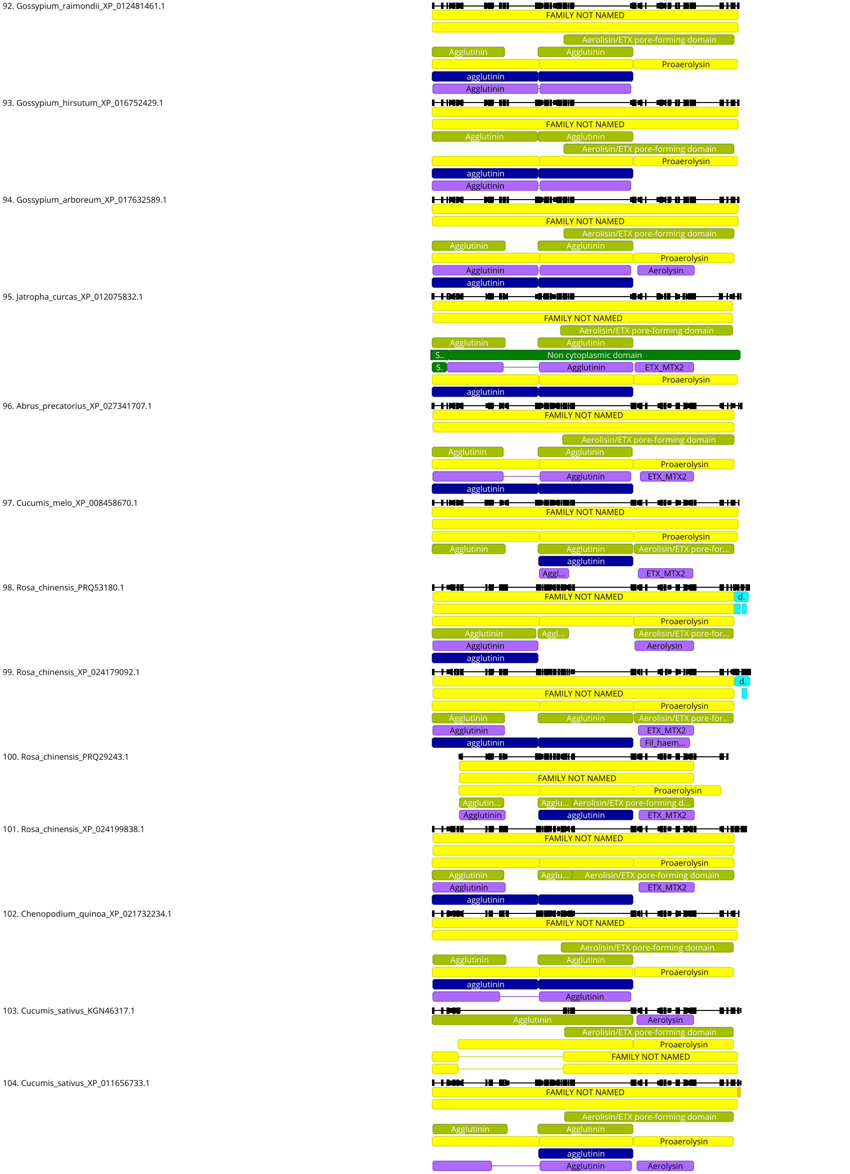
92. Gossypium_raimondii_XP_012481461.1
FAMILY NOT NAMED
Aerolisin/ETX pore-forming domain
Agglutinin
Agglutinin
Proaerolysin
agglutinin
Agglutinin
93. Gossypium_hirsutum_XP_016752429.1
FAMILY NOT NAMED
Agglutinin
Agglutinin
Aerolisin/ETX pore-forming domain
Proaerolysin
agglutinin
Agglutinin
94. Gossypium_arboreum_XP_017632589.1
FAMILY NOT NAMED
Aerolisin/ETX pore-forming domain
Agglutinin
Agglutinin
Proaerolysin
Agglutinin
Aerolysin
agglutinin
95. Jatropha_curcas_XP_012075832.1
FAMILY NOT NAMED
Aerolisin/ETX pore-forming domain
Agglutinin
Agglutinin
S..
Non cytoplasmic domain
S.
Agglutinin
ETX_MTX2
Proaerolysin
agglutinin
96. Abrus_precatorius_XP_027341707.1
FAMILY NOT NAMED
Aerolisin/ETX pore-forming domain
Agglutinin
Agglutinin
Proaerolysin
Agglutinin
ETX_MTX2
agglutinin
97. Cucumis_melo_XP_008458670.1
FAMILY NOT NAMED
Proaerolysin
Agglutinin
Agglutinin
Aerolisin/ETX pore-for...
agglutinin
Aggl...
ETX_MTX2
98. Rosa_chinensis_PRQ53180.1
FAMILY NOT NAMED
d.
Proaerolysin
Agglutinin
Aggl...
Aerolisin/ETX pore-for...
Agglutinin
Aerolysin
agglutinin
99. Rosa_chinensis_XP_024179092.1
d.
FAMILY NOT NAMED
Proaerolysin
Agglutinin
Agglutinin
Aerolisin/ETX pore-for...
Agglutinin
ETX_MTX2
agglutinin
Fil_haem...
100. Rosa_chinensis_PRQ29243.1
FAMILY NOT NAMED
Proaerolysin
Agglutin...
Agglu...
Aerolisin/ETX pore-forming d...
Agglutinin
agglutinin
ETX_MTX2
101. Rosa_chinensis_XP_024199838.1
FAMILY NOT NAMED
Proaerolysin
Agglutinin
Agglu...
Aerolisin/ETX pore-forming domain
Agglutinin
ETX_MTX2
agglutinin
102. Chenopodium_quinoa_XP_021732234.1
FAMILY NOT NAMED
Aerolisin/ETX pore-forming domain
Agglutinin
Agglutinin
Proaerolysin
agglutinin
Agglutinin
103. Cucumis_sativus_KGN46317.1
Agglutinin
Aerolysin
Aerolisin/ETX pore-forming domain
Proaerolysin
FAMILY NOT NAMED
104. Cucumis_sativus_XP_011656733.1
FAMILY NOT NAMED
Aerolisin/ETX pore-forming domain
Agglutinin
Agglutinin
Proaerolysin
agglutinin
Agglutinin
Aerolysin

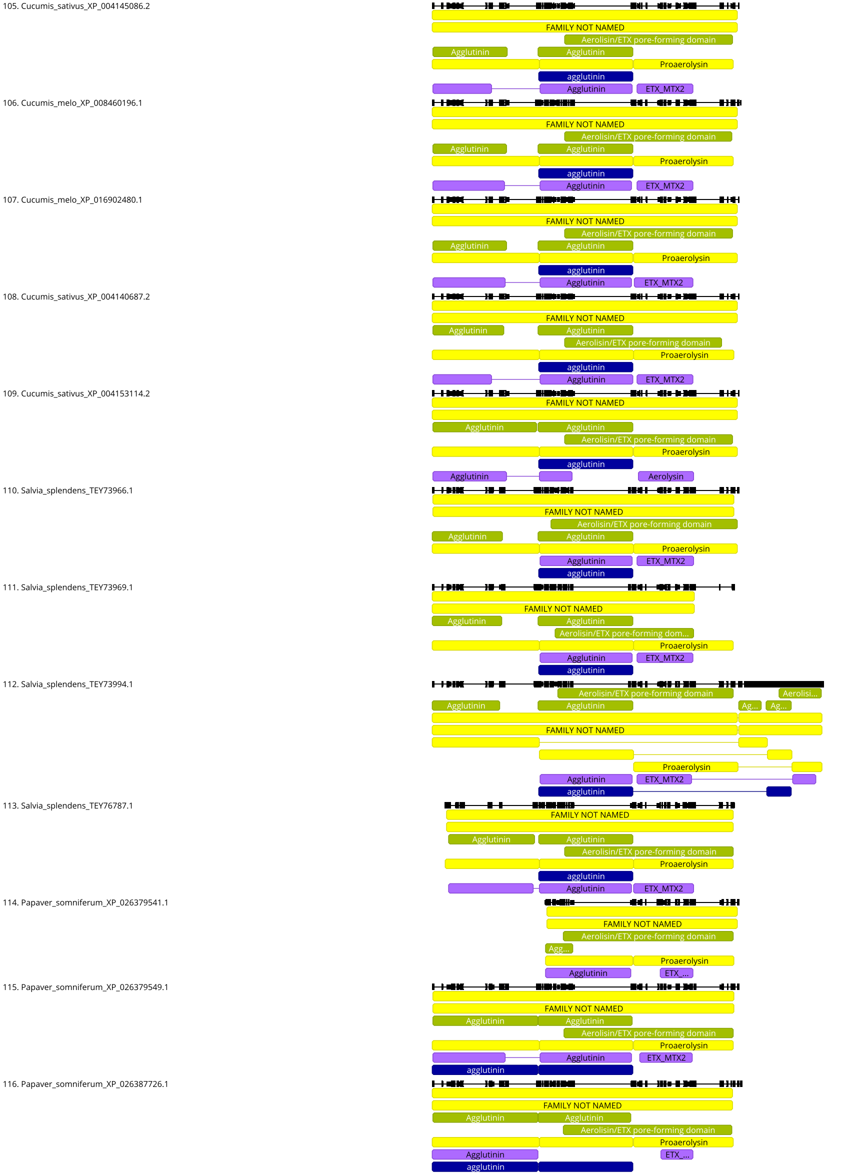
105. Cucumis_sativus_XP_004145086.2
FAMILY NOT NAMED
Aerolisin/ETX pore-forming domain
Agglutinin
Agglutinin
Proaerolysin
agglutinin
Agglutinin
ETX_MTX2
106. Cucumis_melo_XP_008460196.1
FAMILY NOT NAMED
Aerolisin/ETX pore-forming domain
Agglutinin
Agglutinin
Proaerolysin
agglutinin
Agglutinin
ETX_MTX2
107. Cucumis_melo_XP_016902480.1
FAMILY NOT NAMED
Aerolisin/ETX pore-forming domain
Agglutinin
Agglutinin
Proaerolysin
agglutinin
Agglutinin
ETX_MTX2
108. Cucumis_sativus_XP_004140687.2
FAMILY NOT NAMED
Agglutinin
Agglutinin
Aerolisin/ETX pore-forming domain
Proaerolysin
agglutinin
Agglutinin
ETX_MTX2
109. Cucumis_sativus_XP_004153114.2
FAMILY NOT NAMED
Agglutinin
Agglutinin
Aerolisin/ETX pore-forming domain
Proaerolysin
agglutinin
Agglutinin
Aerolysin
110. Salvia_splendens_TEY73966.1
FAMILY NOT NAMED
Aerolisin/ETX pore-forming domain
Agglutinin
Agglutinin
Proaerolysin
Agglutinin
ETX_MTX2
agglutinin
111. Salvia_splendens_TEY73969.1
FAMILY NOT NAMED
Agglutinin
Agglutinin
Aerolisin/ETX pore-forming dom...
Proaerolysin
Agglutinin
ETX_MTX2
agglutinin
112. Salvia_splendens_TEY73994.1
Aerolisin/ETX pore-forming domain
Aerolisi...
Agglutinin
Agglutinin
Ag...
Ag...
FAMILY NOT NAMED
Proaerolysin
Agglutinin
ETX_MTX2
agglutinin
113. Salvia_splendens_TEY76787.1
FAMILY NOT NAMED
Agglutinin
Agglutinin
Aerolisin/ETX pore-forming domain
Proaerolysin
agglutinin
Agglutinin
ETX_MTX2
114. Papaver_somniferum_XP_026379541.1
FAMILY NOT NAMED
Aerolisin/ETX pore-forming domain
Agg...
Proaerolysin
Agglutinin
ETX_...
115. Papaver_somniferum_XP_026379549.1
FAMILY NOT NAMED
Agglutinin
Agglutinin
Aerolisin/ETX pore-forming domain
Proaerolysin
Agglutinin
ETX_MTX2
agglutinin
116. Papaver_somniferum_XP_026387726.1
FAMILY NOT NAMED
Agglutinin
Agglutinin
Aerolisin/ETX pore-forming domain
Proaerolysin
Agglutinin
ETX_...
agglutinin

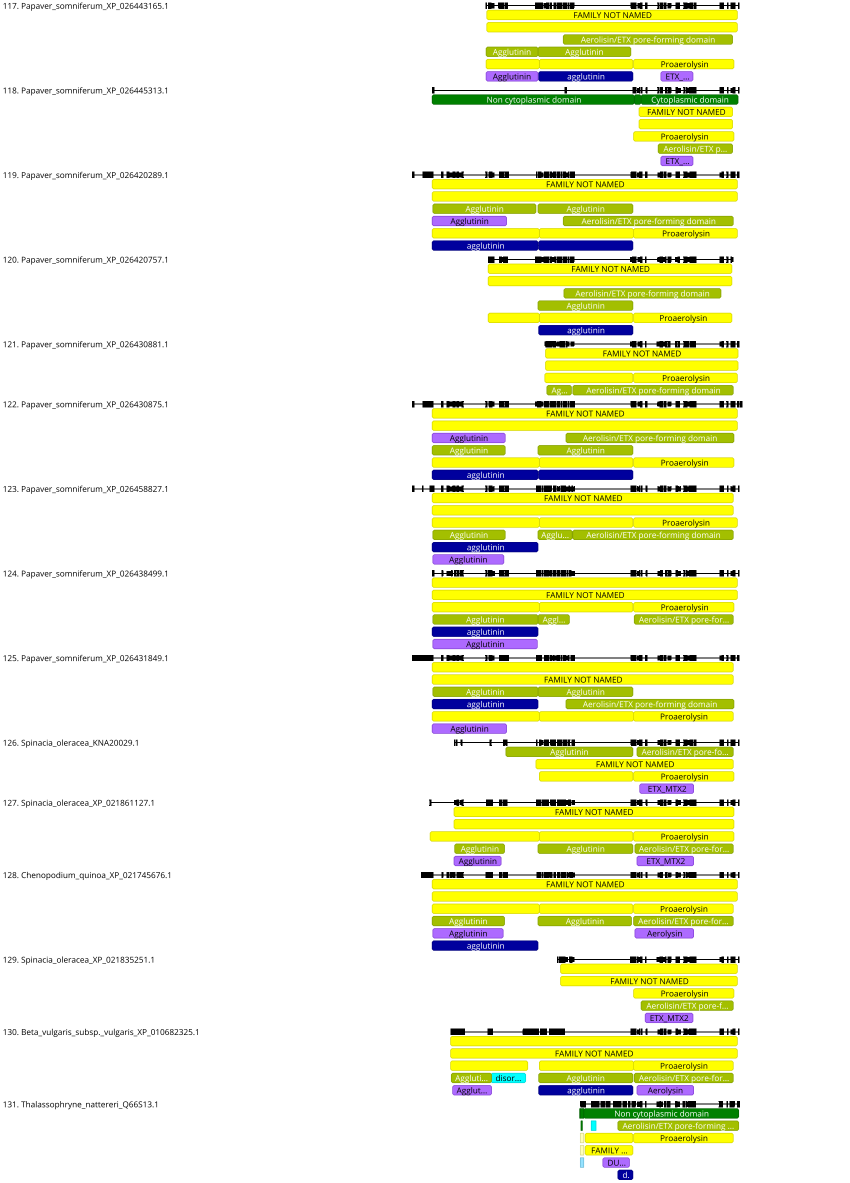
117. Papaver_somniferum_XP_026443165.1
FAMILY NOT NAMED
Aerolisin/ETX pore-forming domain
Agglutinin
Agglutinin
Proaerolysin
Agglutinin
agglutinin
ETX_...
118. Papaver_somniferum_XP_026445313.1
Non cytoplasmic domain
Cytoplasmic domain
FAMILY NOT NAMED
Proaerolysin
Aerolisin/ETX p...
ETX_...
119. Papaver_somniferum_XP_026420289.1
FAMILY NOT NAMED
Agglutinin
Agglutinin
Agglutinin
Aerolisin/ETX pore-forming domain
Proaerolysin
agglutinin
120. Papaver_somniferum_XP_026420757.1
FAMILY NOT NAMED
Aerolisin/ETX pore-forming domain
Agglutinin
Proaerolysin
agglutinin
121. Papaver_somniferum_XP_026430881.1
FAMILY NOT NAMED
Proaerolysin
Ag...
Aerolisin/ETX pore-forming domain
122. Papaver_somniferum_XP_026430875.1
FAMILY NOT NAMED
Agglutinin
Aerolisin/ETX pore-forming domain
Agglutinin
Agglutinin
Proaerolysin
agglutinin
123. Papaver_somniferum_XP_026458827.1
FAMILY NOT NAMED
Proaerolysin
Agglutinin
Agglu...
Aerolisin/ETX pore-forming domain
agglutinin
Agglutinin
124. Papaver_somniferum_XP_026438499.1
FAMILY NOT NAMED
Proaerolysin
Agglutinin
Aggl...
Aerolisin/ETX pore-for...
agglutinin
Agglutinin
125. Papaver_somniferum_XP_026431849.1
FAMILY NOT NAMED
Agglutinin
Agglutinin
agglutinin
Aerolisin/ETX pore-forming domain
Proaerolysin
Agglutinin
126. Spinacia_oleracea_KNA20029.1
Agglutinin
Aerolisin/ETX pore-fo...
FAMILY NOT NAMED
Proaerolysin
ETX_MTX2
127. Spinacia_oleracea_XP_021861127.1
FAMILY NOT NAMED
Proaerolysin
Agglutinin
Agglutinin
Aerolisin/ETX pore-for...
Agglutinin
ETX_MTX2
128. Chenopodium_quinoa_XP_021745676.1
FAMILY NOT NAMED
Proaerolysin
Agglutinin
Agglutinin
Aerolisin/ETX pore-for...
Agglutinin
Aerolysin
agglutinin
129. Spinacia_oleracea_XP_021835251.1
FAMILY NOT NAMED
Proaerolysin
Aerolisin/ETX pore-f...
ETX_MTX2
130. Beta_vulgaris_subsp._vulgaris_XP_010682325.1
FAMILY NOT NAMED
Proaerolysin
Aggluti...
disor...
Agglutinin
Aerolisin/ETX pore-for...
Agglut...
agglutinin
Aerolysin
131. Thalassophryne_nattereri_Q66S13.1
Non cytoplasmic domain
Aerolisin/ETX pore-forming ...
Proaerolysin
FAMILY ...
DU...
d.

132. Cyprinodon_variegatus_XP_015234320.1

133. Poecilia_reticulata_XP_008417566.1

134. Fundulus_heteroclitus_XP_012713937.2

135. Poecilia_formosa_XP_007543104.1

136. Poecilia_mexicana_XP_014861318.1

137. Poecilia_latipinna_XP_014872285.1

138. Poecilia_reticulata_XP_008417563.1

139. Poecilia_mexicana_XP_014861317.1

140. Poecilia_latipinna_XP_014872293.1

141. Poecilia_formosa_XP_016516735.1

142. Poecilia_latipinna_XP_014872284.1

143. Xiphophorus_maculatus_XP_023199097.1

144. Xiphophorus_maculatus_XP_023199098.1

145. Xiphophorus_maculatus_XP_023198850.1

146. Xiphophorus_couchianus_XP_027890011.1

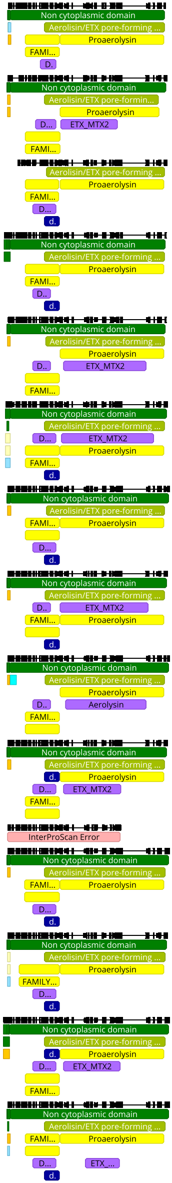

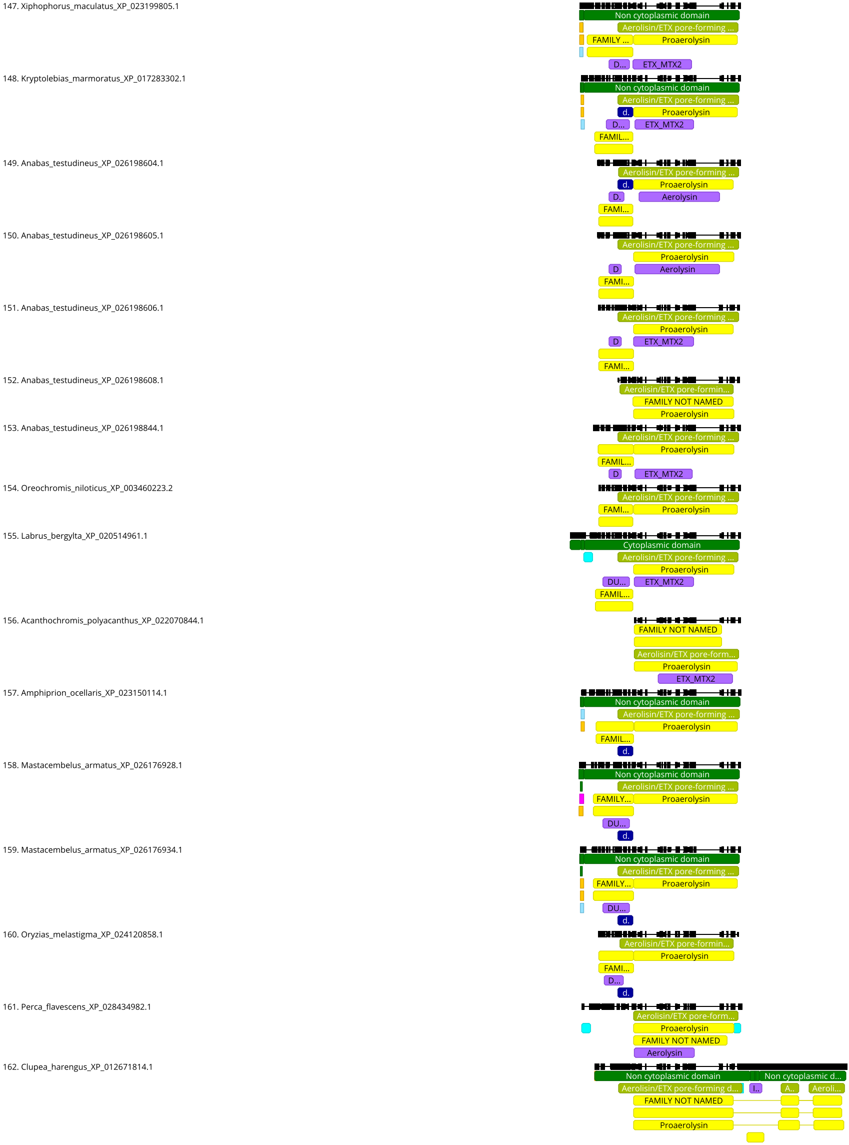
147. Xiphophorus_maculatus_XP_023199805.1
Non cytoplasmic domain
Aerolisin/ETX pore-forming ...
FAMILY ...
Proaerolysin
D...
ETX_MTX2
148. Kryptolebias_marmoratus_XP_017283302.1
Non cytoplasmic domain
Aerolisin/ETX pore-forming ...
d.
Proaerolysin
D...
ETX_MTX2
FAMIL...
149. Anabas_testudineus_XP_026198604.1
Aerolisin/ETX pore-forming ...
d.
Proaerolysin
D.
Aerolysin
FAMI...
150. Anabas_testudineus_XP_026198605.1
Aerolisin/ETX pore-forming ...
Proaerolysin
D
Aerolysin
FAMI...
151. Anabas_testudineus_XP_026198606.1
Aerolisin/ETX pore-forming ...
Proaerolysin
D
ETX_MTX2
FAMI...
152. Anabas_testudineus_XP_026198608.1
Aerolisin/ETX pore-formin...
FAMILY NOT NAMED
Proaerolysin
153. Anabas_testudineus_XP_026198844.1
Aerolisin/ETX pore-forming ...
Proaerolysin
FAMIL...
D
ETX_MTX2
154. Oreochromis_niloticus_XP_003460223.2
Aerolisin/ETX pore-forming ...
FAMI...
Proaerolysin
155. Labrus_bergylta_XP_020514961.1
Cytoplasmic domain
Aerolisin/ETX pore-forming ...
Proaerolysin
DU...
ETX_MTX2
FAMIL...
156. Acanthochromis_polyacanthus_XP_022070844.1
FAMILY NOT NAMED
Aerolisin/ETX pore-form...
Proaerolysin
ETX_MTX2
157. Amphiprion_ocellaris_XP_023150114.1
Non cytoplasmic domain
Aerolisin/ETX pore-forming ...
Proaerolysin
FAMIL...
d.
158. Mastacembelus_armatus_XP_026176928.1
Non cytoplasmic domain
Aerolisin/ETX pore-forming ...
FAMILY...
Proaerolysin
DU...
d.
159. Mastacembelus_armatus_XP_026176934.1
Non cytoplasmic domain
Aerolisin/ETX pore-forming ...
FAMILY...
Proaerolysin
DU...
d.
160. Oryzias_melastigma_XP_024120858.1
Aerolisin/ETX pore-formin...
Proaerolysin
FAMI...
D...
d.
161. Perca_flavescens_XP_028434982.1
Aerolisin/ETX pore-form...
Proaerolysin
FAMILY NOT NAMED
Aerolysin
162. Clupea_harengus_XP_012671814.1
Non cytoplasmic domain
Non cytoplasmic d...
Aerolisin/ETX pore-forming d...
I..
A..
Aeroli...
FAMILY NOT NAMED
Proaerolysin

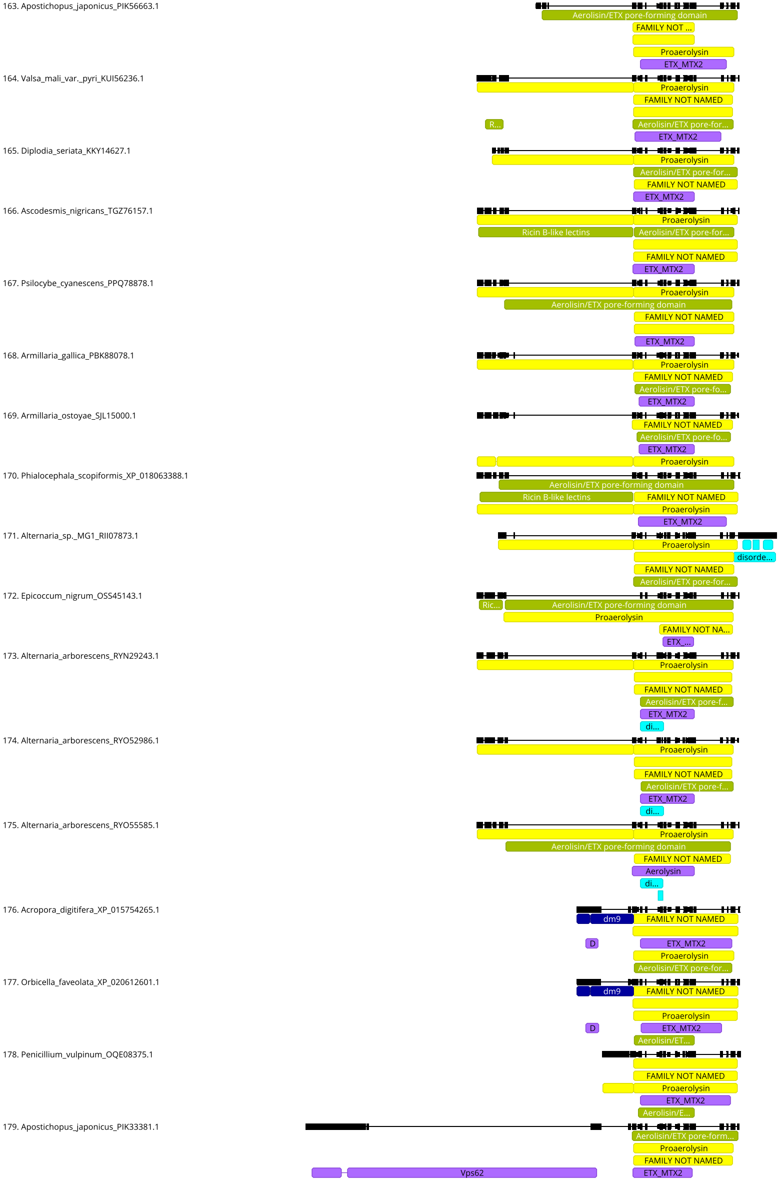

163. Apostichopus_japonicus_PIK56663.1
Aerolisin/ETX pore-forming domain
FAMILY NOT ...
Proaerolysin
ETX_MTX2
164. Valsa_mali_var._pyri_KUI56236.1
Proaerolysin
FAMILY NOT NAMED
R...
Aerolisin/ETX pore-for...
ETX_MTX2
165. Diplodia_seriata_KKY14627.1
Proaerolysin
Aerolisin/ETX pore-for...
FAMILY NOT NAMED
ETX_MTX2
166. Ascodesmis_nigricans_TGZ76157.1
Proaerolysin
Ricin B-like lectins
Aerolisin/ETX pore-for...
FAMILY NOT NAMED
ETX_MTX2
167. Psilocybe_cyanescens_PPQ78878.1
Proaerolysin
Aerolisin/ETX pore-forming domain
FAMILY NOT NAMED
ETX_MTX2
168. Armillaria_gallica_PBK88078.1
Proaerolysin
FAMILY NOT NAMED
Aerolisin/ETX pore-fo...
ETX_MTX2
169. Armillaria_ostoyae_SJL15000.1
FAMILY NOT NAMED
Aerolisin/ETX pore-fo...
ETX_MTX2
Proaerolysin
170. Phialocephala_scopiformis_XP_018063388.1
Aerolisin/ETX pore-forming domain
Ricin B-like lectins
FAMILY NOT NAMED
Proaerolysin
ETX_MTX2
171. Alternaria_sp._MG1_RII07873.1
Proaerolysin
disorde...
FAMILY NOT NAMED
Aerolisin/ETX pore-for...
172. Epicoccum_nigrum_OSS45143.1
Ric...
Aerolisin/ETX pore-forming domain
Proaerolysin
FAMILY NOT NA...
ETX_...
173. Alternaria_arborescens_RYN29243.1
Proaerolysin
FAMILY NOT NAMED
Aerolisin/ETX pore-f...
ETX_MTX2
di...
174. Alternaria_arborescens_RYO52986.1
Proaerolysin
FAMILY NOT NAMED
Aerolisin/ETX pore-f...
ETX_MTX2
di...
175. Alternaria_arborescens_RYO55585.1
Proaerolysin
Aerolisin/ETX pore-forming domain
FAMILY NOT NAMED
Aerolysin
di...
176. Acropora_digitifera_XP_015754265.1
dm9
FAMILY NOT NAMED
D
ETX_MTX2
Proaerolysin
Aerolisin/ETX pore-for...
177. Orbicella_faveolata_XP_020612601.1
dm9
FAMILY NOT NAMED
Proaerolysin
D
ETX_MTX2
Aerolisin/ET...
178. Penicillium_vulpinum_OQE08375.1
FAMILY NOT NAMED
Proaerolysin
ETX_MTX2
Aerolisin/E...
179. Apostichopus_japonicus_PIK33381.1
Aerolisin/ETX pore-form...
Proaerolysin
FAMILY NOT NAMED
Vps62
ETX_MTX2

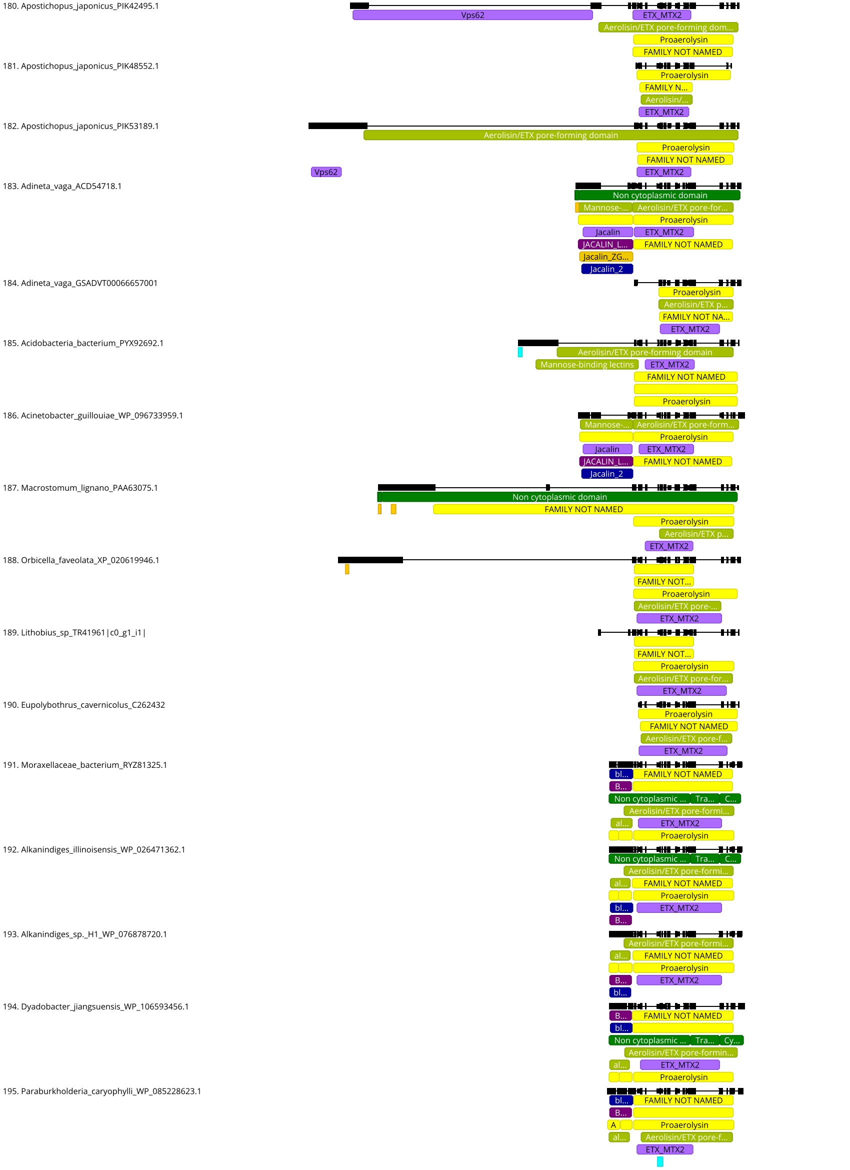

180. Apostichopus_japonicus_PIK42495.1
Vps62
ETX_MTX2
Aerolisin/ETX pore-forming dom...
Proaerolysin
FAMILY NOT NAMED
181. Apostichopus_japonicus_PIK48552.1
Proaerolysin
FAMILY N...
Aerolisin/...
ETX_MTX2
182. Apostichopus_japonicus_PIK53189.1
Aerolisin/ETX pore-forming domain
Proaerolysin
FAMILY NOT NAMED
Vps62
ETX_MTX2
183. Adineta_vaga_ACD54718.1
Non cytoplasmic domain
Mannose-...
Aerolisin/ETX pore-for...
Proaerolysin
Jacalin
ETX_MTX2
JACALIN_L...
FAMILY NOT NAMED
Jacalin_ZG...
Jacalin_2
184. Adineta_vaga_GSADVT00066657001
Proaerolysin
Aerolisin/ETX p...
FAMILY NOT NA...
ETX_MTX2
185. Acidobacteria_bacterium_PYX92692.1
Aerolisin/ETX pore-forming domain
Mannose-binding lectins
ETX_MTX2
FAMILY NOT NAMED
Proaerolysin
186. Acinetobacter_guillouiae_WP_096733959.1
Mannose-...
Aerolisin/ETX pore-form...
Proaerolysin
Jacalin
ETX_MTX2
JACALIN_L...
FAMILY NOT NAMED
Jacalin_2
187. Macrostomum_lignano_PAA63075.1
Non cytoplasmic domain
FAMILY NOT NAMED
Proaerolysin
Aerolisin/ETX p...
ETX_MTX2
188. Orbicella_faveolata_XP_020619946.1
FAMILY NOT...
Proaerolysin
Aerolisin/ETX pore-...
ETX_MTX2
189. Lithobius_sp_TR41961|c0_g1_i1|
FAMILY NOT...
Proaerolysin
Aerolisin/ETX pore-for...
ETX_MTX2
190. Eupolybothrus_cavernicolus_C262432
Proaerolysin
FAMILY NOT NAMED
Aerolisin/ETX pore-f...
ETX_MTX2
191. Moraxellaceae_bacterium_RYZ81325.1
bl...
FAMILY NOT NAMED
B...
Non cytoplasmic ...
Tra...
C...
Aerolisin/ETX pore-formi...
al...
ETX_MTX2
Proaerolysin
192. Alkanindiges_illinoisensis_WP_026471362.1
Non cytoplasmic ...
Tra...
C...
Aerolisin/ETX pore-formi...
al...
FAMILY NOT NAMED
Proaerolysin
bl...
ETX_MTX2
B...
193. Alkanindiges_sp._H1_WP_076878720.1
Aerolisin/ETX pore-formi...
al...
FAMILY NOT NAMED
Proaerolysin
B...
ETX_MTX2
bl...
194. Dyadobacter_jiangsuensis_WP_106593456.1
B...
FAMILY NOT NAMED
bl...
Non cytoplasmic ...
Tra...
Cy...
Aerolisin/ETX pore-formin...
al...
ETX_MTX2
Proaerolysin
195. Paraburkholderia_caryophylli_WP_085228623.1
bl...
FAMILY NOT NAMED
B...
A
Proaerolysin
al...
Aerolisin/ETX pore-f...
ETX_MTX2

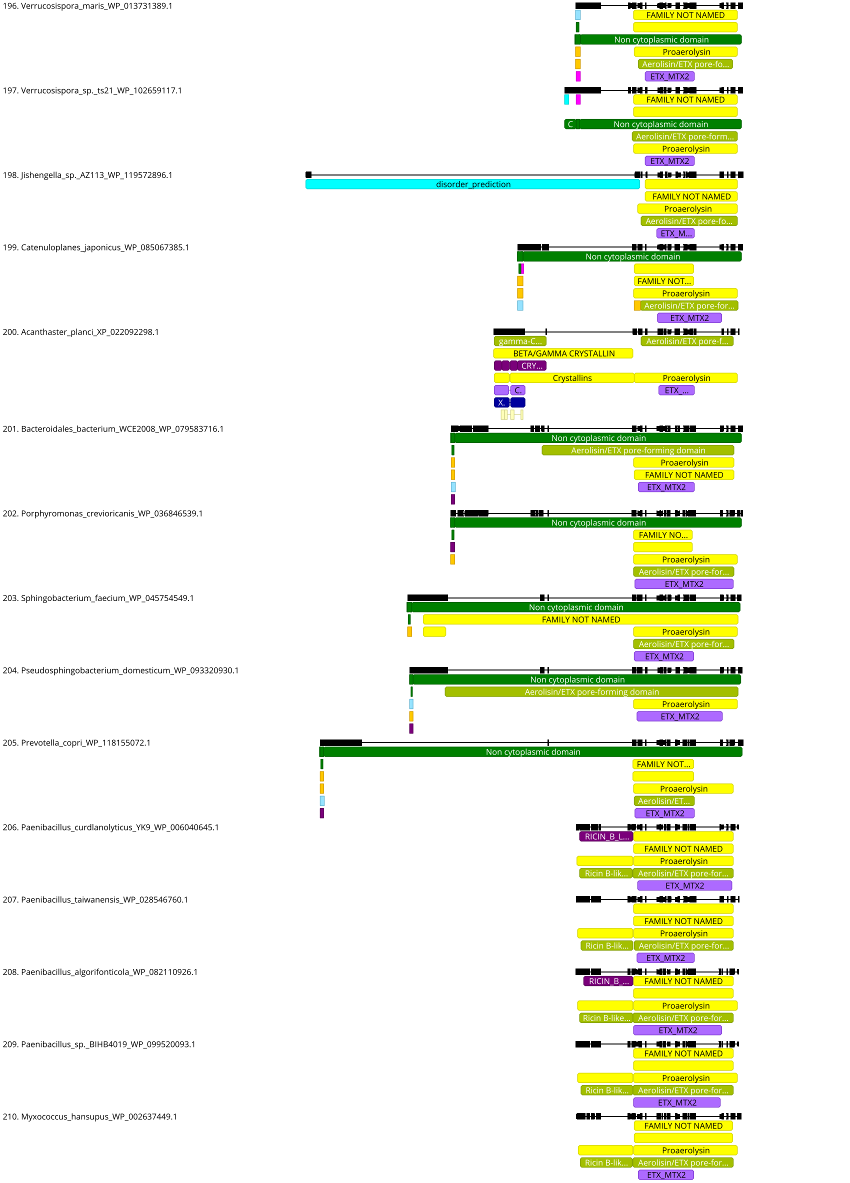
196. Verrucosispora_maris_WP_013731389.1
FAMILY NOT NAMED
Non cytoplasmic domain
Proaerolysin
Aerolisin/ETX pore-fo...
ETX_MTX2
197. Verrucosispora_sp._ts21_WP_102659117.1
FAMILY NOT NAMED
C
Non cytoplasmic domain
Aerolisin/ETX pore-form...
Proaerolysin
ETX_MTX2
198. Jishengella_sp._AZ113_WP_119572896.1
disorder_prediction
FAMILY NOT NAMED
Proaerolysin
Aerolisin/ETX pore-fo...
ETX_M...
199. Catenuloplanes_japonicus_WP_085067385.1
Non cytoplasmic domain
FAMILY NOT...
Proaerolysin
Aerolisin/ETX pore-for...
ETX_MTX2
200. Acanthaster_planci_XP_022092298.1
gamma-C...
Aerolisin/ETX pore-f...
BETA/GAMMA CRYSTALLIN
CRY...
Crystallins
Proaerolysin
C.
ETX_...
X.
201. Bacteroidales_bacterium_WCE2008_WP_079583716.1
Non cytoplasmic domain
Aerolisin/ETX pore-forming domain
Proaerolysin
FAMILY NOT NAMED
ETX_MTX2
202. Porphyromonas_crevioricanis_WP_036846539.1
Non cytoplasmic domain
FAMILY NO...
Proaerolysin
Aerolisin/ETX pore-for...
ETX_MTX2
203. Sphingobacterium_faecium_WP_045754549.1
Non cytoplasmic domain
FAMILY NOT NAMED
Proaerolysin
Aerolisin/ETX pore-for...
ETX_MTX2
204. Pseudosphingobacterium_domesticum_WP_093320930.1
Non cytoplasmic domain
Aerolisin/ETX pore-forming domain
Proaerolysin
ETX_MTX2
205. Prevotella_copri_WP_118155072.1
Non cytoplasmic domain
FAMILY NOT...
Proaerolysin
Aerolisin/ET...
ETX_MTX2
206. Paenibacillus_curdlanolyticus_YK9_WP_006040645.1
RICIN_B_L...
FAMILY NOT NAMED
Proaerolysin
Ricin B-lik...
Aerolisin/ETX pore-for...
ETX_MTX2
207. Paenibacillus_taiwanensis_WP_028546760.1
FAMILY NOT NAMED
Proaerolysin
Ricin B-lik...
Aerolisin/ETX pore-for...
ETX_MTX2
208. Paenibacillus_algorifonticola_WP_082110926.1
RICIN_B_...
FAMILY NOT NAMED
Proaerolysin
Ricin B-like...
Aerolisin/ETX pore-for...
ETX_MTX2
209. Paenibacillus_sp._BIHB4019_WP_099520093.1
FAMILY NOT NAMED
Proaerolysin
Ricin B-lik...
Aerolisin/ETX pore-for...
ETX_MTX2
210. Myxococcus_hansupus_WP_002637449.1
FAMILY NOT NAMED
Proaerolysin
Ricin B-lik...
Aerolisin/ETX pore-for...
ETX_MTX2

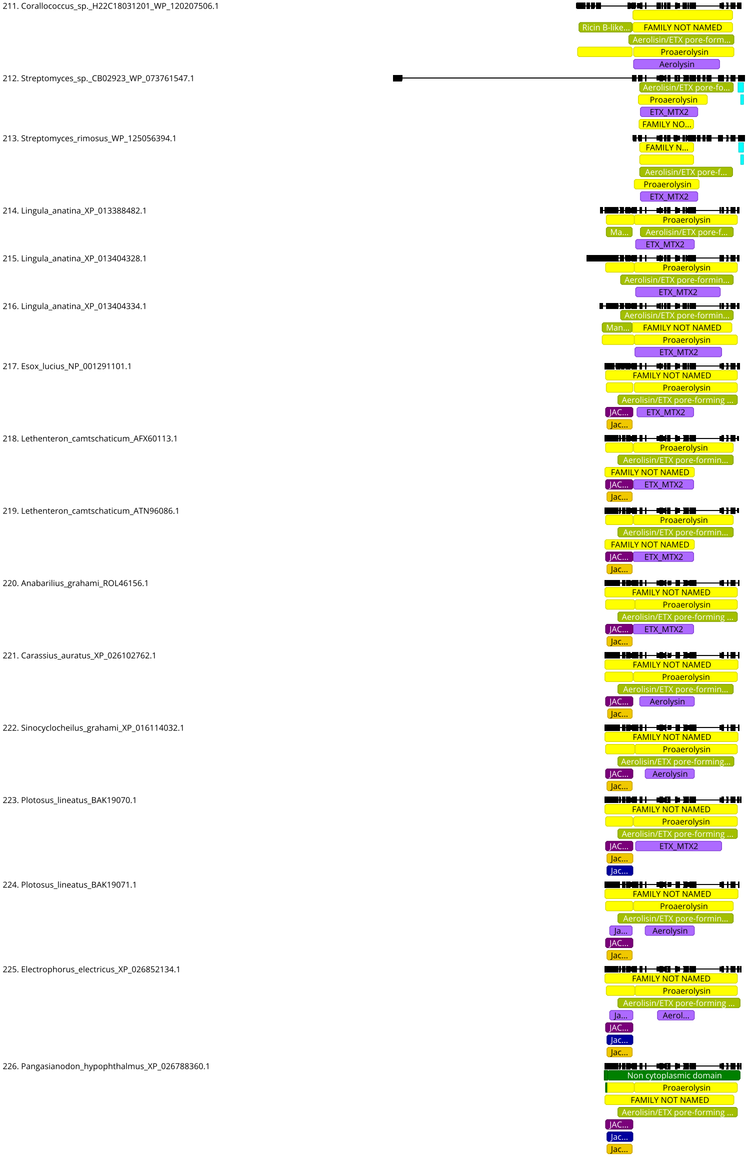
211. Corallococcus_sp._H22C18031201_WP_120207506.1
Ricin B-like...
FAMILY NOT NAMED
Aerolisin/ETX pore-form...
Proaerolysin
Aerolysin
212. Streptomyces_sp._CB02923_WP_073761547.1
Aerolisin/ETX pore-fo...
Proaerolysin
ETX_MTX2
FAMILY NO...
213. Streptomyces_rimosus_WP_125056394.1
FAMILY N...
Aerolisin/ETX pore-f...
Proaerolysin
ETX_MTX2
214. Lingula_anatina_XP_013388482.1
Proaerolysin
Ma...
Aerolisin/ETX pore-f...
ETX_MTX2
215. Lingula_anatina_XP_013404328.1
Proaerolysin
Aerolisin/ETX pore-formin...
ETX_MTX2
216. Lingula_anatina_XP_013404334.1
Aerolisin/ETX pore-formin...
Man...
FAMILY NOT NAMED
Proaerolysin
ETX_MTX2
217. Esox_lucius_NP_001291101.1
FAMILY NOT NAMED
Proaerolysin
Aerolisin/ETX pore-forming ...
JAC...
ETX_MTX2
Jac...
218. Lethenteron_camtschaticum_AFX60113.1
Proaerolysin
Aerolisin/ETX pore-formin...
FAMILY NOT NAMED
JAC...
ETX_MTX2
Jac...
219. Lethenteron_camtschaticum_ATN96086.1
Proaerolysin
Aerolisin/ETX pore-formin...
FAMILY NOT NAMED
JAC...
ETX_MTX2
Jac...
220. Anabarilius_grahami_ROL46156.1
FAMILY NOT NAMED
Proaerolysin
Aerolisin/ETX pore-forming ...
JAC...
ETX_MTX2
Jac...
221. Carassius_auratus_XP_026102762.1
FAMILY NOT NAMED
Proaerolysin
Aerolisin/ETX pore-formin...
JAC...
Aerolysin
Jac...
222. Sinocyclocheilus_grahami_XP_016114032.1
FAMILY NOT NAMED
Proaerolysin
Aerolisin/ETX pore-forming...
JAC...
Aerolysin
Jac...
223. Plotosus_lineatus_BAK19070.1
FAMILY NOT NAMED
Proaerolysin
Aerolisin/ETX pore-forming ...
JAC...
ETX_MTX2
Jac...
Jac...
224. Plotosus_lineatus_BAK19071.1
FAMILY NOT NAMED
Proaerolysin
Aerolisin/ETX pore-formin...
Ja...
Aerolysin
JAC...
Jac...
225. Electrophorus_electricus_XP_026852134.1
FAMILY NOT NAMED
Proaerolysin
Aerolisin/ETX pore-forming ...
Ja...
Aerol...
JAC...
Jac...
Jac...
226. Pangasianodon_hypophthalmus_XP_026788360.1
Non cytoplasmic domain
Proaerolysin
FAMILY NOT NAMED
Aerolisin/ETX pore-forming ...
JAC...
Jac...
Jac...

227. Ictalurus_punctatus_XP_017352097.1

FAMILY NOT NAMED
Proaerolysin
Aerolisin/ETX pore-forming ...
JAC...
Jac...

228. Tachysurus_fulvidraco_XP_027023473.1

Proaerolysin
FAMILY NOT NAMED
Aerolisin/ETX pore-forming ...
JAC...
Ja...

229. Tachysurus_fulvidraco_XP_027023477.1

Proaerolysin
FAMILY NOT NAMED
Aerolisin/ETX pore-forming...
JAC...
Ja...

230. Tachysurus_fulvidraco_XP_027023616.1

FAMILY NOT NAMED
Proaerolysin
Aerolisin/ETX pore-forming ...
JAC...
Ja...

231. Pygocentrus_nattereri_XP_017560326.1

Non cytoplasmic domain
Proaerolysin
FAMILY NOT NAMED
Aerolisin/ETX pore-formin...
Ja... Aerolysin
JAC...
Jac...
Jac...

232. Paramormyrops_kingsleyae_XP_023671347.1

FAMILY NOT NAMED
Proaerolysin
Aerolisin/ETX pore-formin...
JAC... Aerolysin
Jac...

233. Paramormyrops_kingsleyae_XP_023660707.1

FAMILY NOT NAMED
Proaerolysin
Aerolisin/ETX pore-formin...
JAC...
Jac...

234. Lepisosteus_oculatus_XP_006628335.1

FAMILY NOT NAMED
Proaerolysin
Aerolisin/ETX pore-forming...
JAC...
Jac...

235. Anabas_testudineus_XP_026224000.1

FAMILY NOT NAMED
Proaerolysin
Aerolisin/ETX pore-formin...
JAC...
Jac...

236. Anabas_testudineus_XP_026224001.1

FAMILY NOT NAMED
Proaerolysin
Aerolisin/ETX pore-formin...
JAC...
Ja...

237. Oryzias_javanicus_RVE75191.1

Proaerolysin
FAMILY NOT NAMED
Aerolisin/ETX pore-formin...
JAC... Aerolysin
Jac...

238. Oryzias_melastigma_XP_024144712.1

Proaerolysin
FAMILY NOT NAMED
Aerolisin/ETX pore-formin...
JAC... Aeroly...
Jac...

239. Oryzias_melastigma_XP_024144711.1

FAMILY NOT NAMED
Proaerolysin
Aerolisin/ETX pore-formin...
JAC... Aeroly...
Jac...

240. Oryzias_melastigma_XP_024121381.1

Aerolisin/ETX pore-for...
Proaerolysin
FAMILY NOT NAMED
Aerolysin

241. Oryzias_latipes_XP_004086107.1

FAMILY NOT NAMED
Proaerolysin
Aerolisin/ETX pore-formin...
JAC... Aeroly...
Jac...

242. Oryzias_latipes_XP_020555873.1

FAMILY NOT NAMED
Proaerolysin
Aerolisin/ETX pore-formin...
JAC... Aerolysin
Jac...

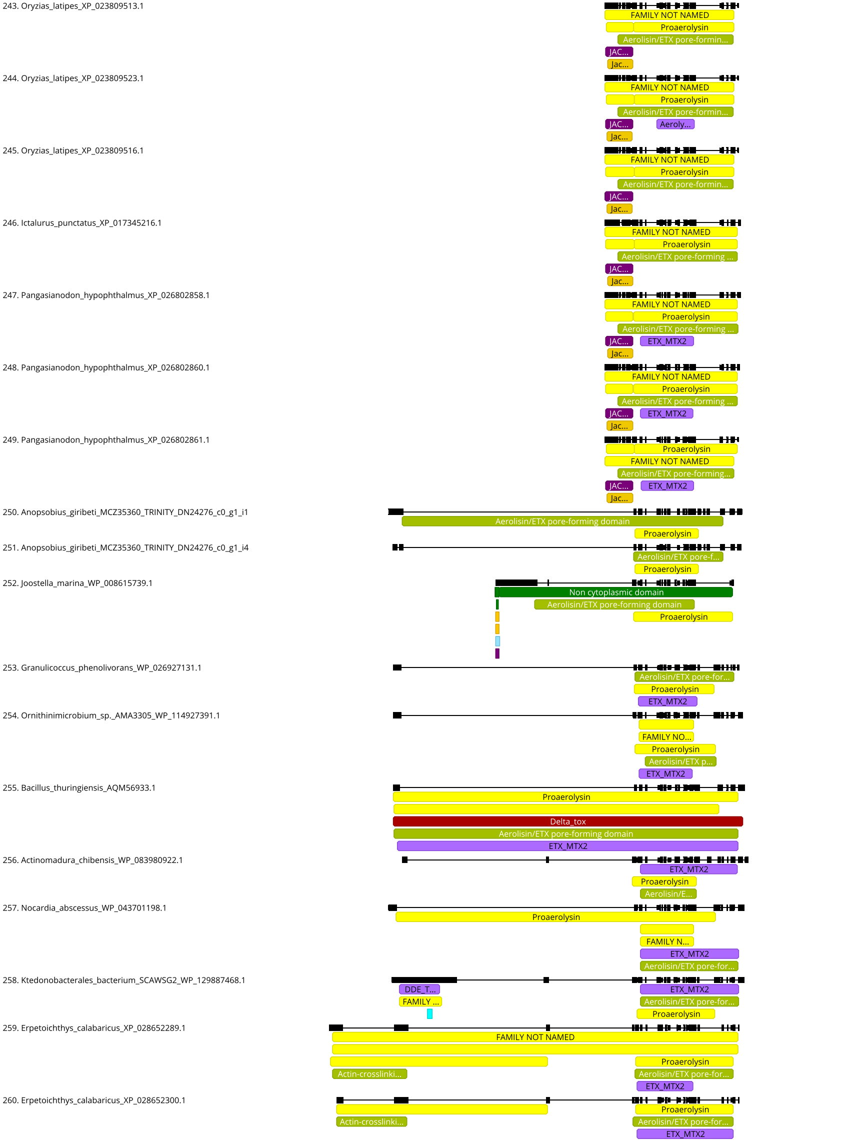
243. Oryzias_latipes_XP_023809513.1
FAMILY NOT NAMED
Proaerolysin
Aerolisin/ETX pore-formin...
JAC...
Jac...
244. Oryzias_latipes_XP_023809523.1
FAMILY NOT NAMED
Proaerolysin
Aerolisin/ETX pore-formin...
JAC...
Aeroly...
Jac...
245. Oryzias_latipes_XP_023809516.1
FAMILY NOT NAMED
Proaerolysin
Aerolisin/ETX pore-formin...
JAC...
Jac...
246. Ictalurus_punctatus_XP_017345216.1
FAMILY NOT NAMED
Proaerolysin
Aerolisin/ETX pore-forming ...
JAC...
Jac...
247. Pangasianodon_hypophthalmus_XP_026802858.1
FAMILY NOT NAMED
Proaerolysin
Aerolisin/ETX pore-forming ...
JAC...
ETX_MTX2
Jac...
248. Pangasianodon_hypophthalmus_XP_026802860.1
FAMILY NOT NAMED
Proaerolysin
Aerolisin/ETX pore-forming ...
JAC...
ETX_MTX2
Jac...
249. Pangasianodon_hypophthalmus_XP_026802861.1
Proaerolysin
FAMILY NOT NAMED
Aerolisin/ETX pore-forming...
JAC...
ETX_MTX2
Jac...
250. Anopsobius_giribeti_MCZ35360_TRINITY_DN24276_c0_g1_i1
Aerolisin/ETX pore-forming domain
Proaerolysin
251. Anopsobius_giribeti_MCZ35360_TRINITY_DN24276_c0_g1_i4
Aerolisin/ETX pore-f...
Proaerolysin
252. Joostella_marina_WP_008615739.1
Non cytoplasmic domain
Aerolisin/ETX pore-forming domain
Proaerolysin
253. Granulicoccus_phenolivorans_WP_026927131.1
Aerolisin/ETX pore-for...
Proaerolysin
ETX_MTX2
254. Ornithinimicrobium_sp._AMA3305_WP_114927391.1
FAMILY NO...
Proaerolysin
Aerolisin/ETX p...
ETX_MTX2
255. Bacillus_thuringiensis_AQM56933.1
Proaerolysin
Delta_tox
Aerolisin/ETX pore-forming domain
ETX_MTX2
256. Actinomadura_chibensis_WP_083980922.1
ETX_MTX2
Proaerolysin
Aerolisin/E...
257. Nocardia_abscessus_WP_043701198.1
Proaerolysin
FAMILY N...
ETX_MTX2
Aerolisin/ETX pore-for...
258. Ktedonobacterales_bacterium_SCAWSG2_WP_129887468.1
DDE_T...
ETX_MTX2
FAMILY ...
Aerolisin/ETX pore-for...
Proaerolysin
259. Erpetoichthys_calabaricus_XP_028652289.1
FAMILY NOT NAMED
Proaerolysin
Actin-crosslinki...
Aerolisin/ETX pore-for...
ETX_MTX2
260. Erpetoichthys_calabaricus_XP_028652300.1
Proaerolysin
Actin-crosslinki...
Aerolisin/ETX pore-for...
ETX_MTX2

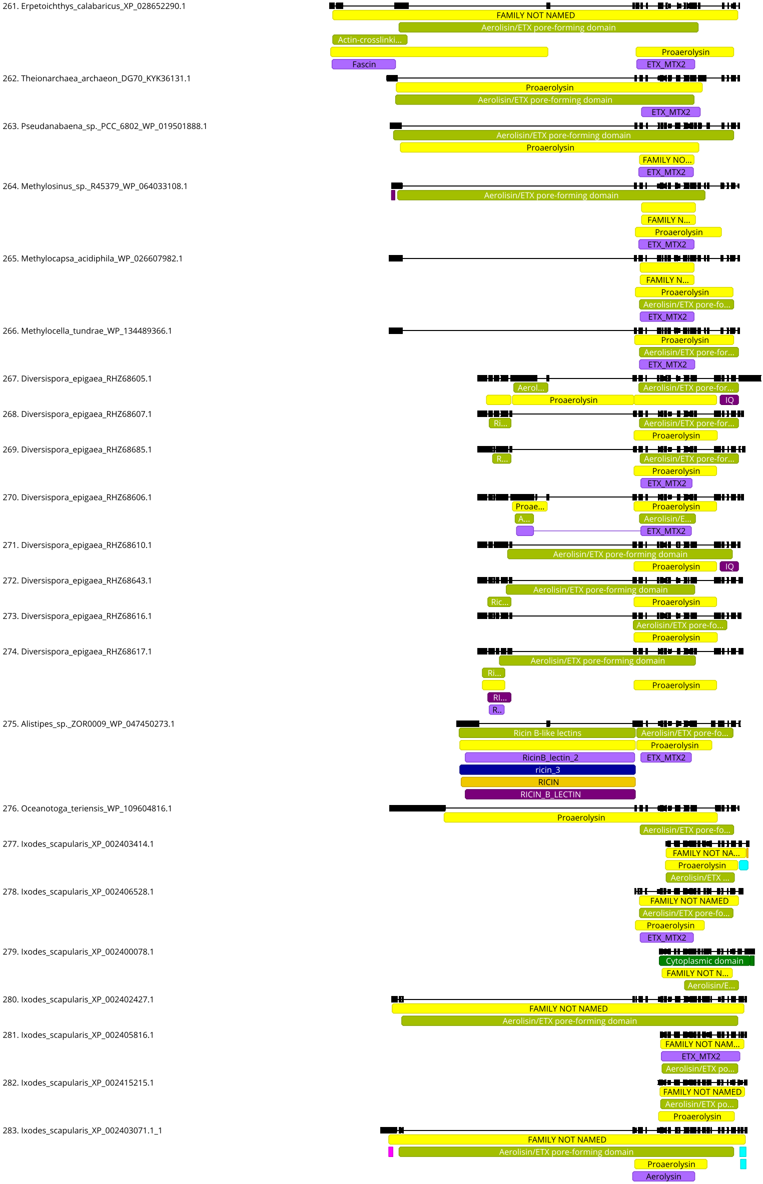
261. Erpetoichthys_calabaricus_XP_028652290.1
FAMILY NOT NAMED
Aerolisin/ETX pore-forming domain
Actin-crosslinki...
Proaerolysin
Fascin
ETX_MTX2
262. Theionarchaea_archaeon_DG70_KYK36131.1
Proaerolysin
Aerolisin/ETX pore-forming domain
ETX_MTX2
263. Pseudanabaena_sp._PCC_6802_WP_019501888.1
Aerolisin/ETX pore-forming domain
Proaerolysin
FAMILY NO...
ETX_MTX2
264. Methylosinus_sp._R45379_WP_064033108.1
Aerolisin/ETX pore-forming domain
FAMILY N...
Proaerolysin
ETX_MTX2
265. Methylocapsa_acidiphila_WP_026607982.1
FAMILY N...
Proaerolysin
Aerolisin/ETX pore-fo...
ETX_MTX2
266. Methylocella_tundrae_WP_134489366.1
Proaerolysin
Aerolisin/ETX pore-for...
ETX_MTX2
267. Diversispora_epigaea_RHZ68605.1
Aerol...
Aerolisin/ETX pore-for...
Proaerolysin
IQ
268. Diversispora_epigaea_RHZ68607.1
Ri...
Aerolisin/ETX pore-for...
Proaerolysin
269. Diversispora_epigaea_RHZ68685.1
R...
Aerolisin/ETX pore-for...
Proaerolysin
ETX_MTX2
270. Diversispora_epigaea_RHZ68606.1
Proae...
Proaerolysin
A...
Aerolisin/E...
ETX_MTX2
271. Diversispora_epigaea_RHZ68610.1
Aerolisin/ETX pore-forming domain
Proaerolysin
IQ
272. Diversispora_epigaea_RHZ68643.1
Aerolisin/ETX pore-forming domain
Ric...
Proaerolysin
273. Diversispora_epigaea_RHZ68616.1
Aerolisin/ETX pore-fo...
Proaerolysin
274. Diversispora_epigaea_RHZ68617.1
Aerolisin/ETX pore-forming domain
Ri...
Proaerolysin
RI...
R..
275. Alistipes_sp._ZOR0009_WP_047450273.1
Ricin B-like lectins
Aerolisin/ETX pore-fo...
Proaerolysin
RicinB_lectin_2
ETX_MTX2
ricin_3
RICIN
RICIN_B_LECTIN
276. Oceanotoga_teriensis_WP_109604816.1
Proaerolysin
Aerolisin/ETX pore-fo...
277. Ixodes_scapularis_XP_002403414.1
FAMILY NOT NA...
Proaerolysin
Aerolisin/ETX ...
278. Ixodes_scapularis_XP_002406528.1
FAMILY NOT NAMED
Aerolisin/ETX pore-fo...
Proaerolysin
ETX_MTX2
279. Ixodes_scapularis_XP_002400078.1
Cytoplasmic domain
FAMILY NOT N...
Aerolisin/E...
280. Ixodes_scapularis_XP_002402427.1
FAMILY NOT NAMED
Aerolisin/ETX pore-forming domain
281. Ixodes_scapularis_XP_002405816.1
FAMILY NOT NAM...
ETX_MTX2
Aerolisin/ETX po...
282. Ixodes_scapularis_XP_002415215.1
FAMILY NOT NAMED
Aerolisin/ETX po...
Proaerolysin
283. Ixodes_scapularis_XP_002403071.1_1
FAMILY NOT NAMED
Aerolisin/ETX pore-forming domain
Proaerolysin
Aerolysin

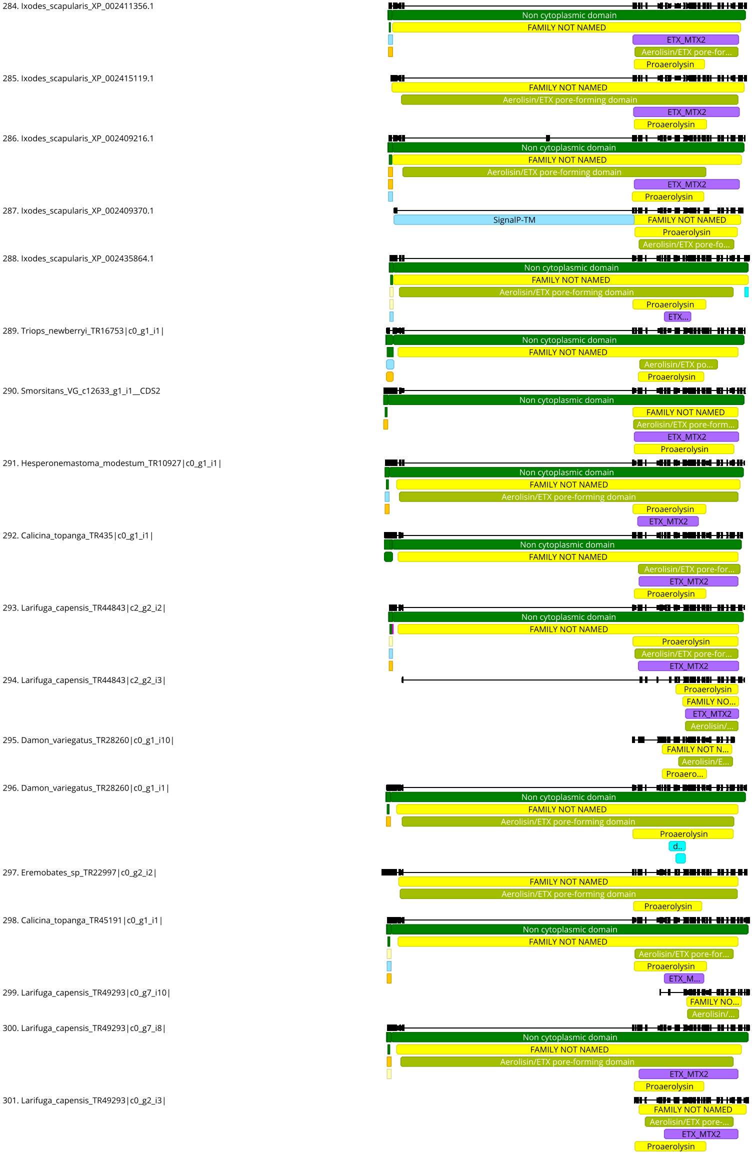
284. Ixodes_scapularis_XP_002411356.1
Non cytoplasmic domain
FAMILY NOT NAMED
ETX_MTX2
Aerolisin/ETX pore-for...
Proaerolysin
285. Ixodes_scapularis_XP_002415119.1
FAMILY NOT NAMED
Aerolisin/ETX pore-forming domain
ETX_MTX2
Proaerolysin
286. Ixodes_scapularis_XP_002409216.1
Non cytoplasmic domain
FAMILY NOT NAMED
Aerolisin/ETX pore-forming domain
ETX_MTX2
Proaerolysin
287. Ixodes_scapularis_XP_002409370.1
SignalP-TM
FAMILY NOT NAMED
Proaerolysin
Aerolisin/ETX pore-fo...
288. Ixodes_scapularis_XP_002435864.1
Non cytoplasmic domain
FAMILY NOT NAMED
Aerolisin/ETX pore-forming domain
Proaerolysin
ETX...
289. Triops_newberryi_TR16753|c0_g1_i1|
Non cytoplasmic domain
FAMILY NOT NAMED
Aerolisin/ETX po...
Proaerolysin
290. Smorsitans_VG_c12633_g1_i1__CDS2
Non cytoplasmic domain
FAMILY NOT NAMED
Aerolisin/ETX pore-form...
ETX_MTX2
Proaerolysin
291. Hesperonemastoma_modestum_TR10927|c0_g1_i1|
Non cytoplasmic domain
FAMILY NOT NAMED
Aerolisin/ETX pore-forming domain
Proaerolysin
ETX_MTX2
292. Calicina_topanga_TR435|c0_g1_i1|
Non cytoplasmic domain
FAMILY NOT NAMED
Aerolisin/ETX pore-for...
ETX_MTX2
Proaerolysin
293. Larifuga_capensis_TR44843|c2_g2_i2|
Non cytoplasmic domain
FAMILY NOT NAMED
Proaerolysin
Aerolisin/ETX pore-for...
ETX_MTX2
294. Larifuga_capensis_TR44843|c2_g2_i3|
Proaerolysin
FAMILY NO...
ETX_MTX2
Aerolisin/...
295. Damon_variegatus_TR28260|c0_g1_i10|
FAMILY NOT N...
Aerolisin/E...
Proaero...
296. Damon_variegatus_TR28260|c0_g1_i1|
Non cytoplasmic domain
FAMILY NOT NAMED
Aerolisin/ETX pore-forming domain
Proaerolysin
d..
297. Eremobates_sp_TR22997|c0_g2_i2|
FAMILY NOT NAMED
Aerolisin/ETX pore-forming domain
Proaerolysin
298. Calicina_topanga_TR45191|c0_g1_i1|
Non cytoplasmic domain
FAMILY NOT NAMED
Aerolisin/ETX pore-for...
Proaerolysin
ETX_M...
299. Larifuga_capensis_TR49293|c0_g7_i10|
FAMILY NO...
Aerolisin/...
300. Larifuga_capensis_TR49293|c0_g7_i8|
Non cytoplasmic domain
FAMILY NOT NAMED
Aerolisin/ETX pore-forming domain
ETX_MTX2
Proaerolysin
301. Larifuga_capensis_TR49293|c0_g2_i3|
FAMILY NOT NAMED
Aerolisin/ETX pore-...
ETX_MTX2
Proaerolysin

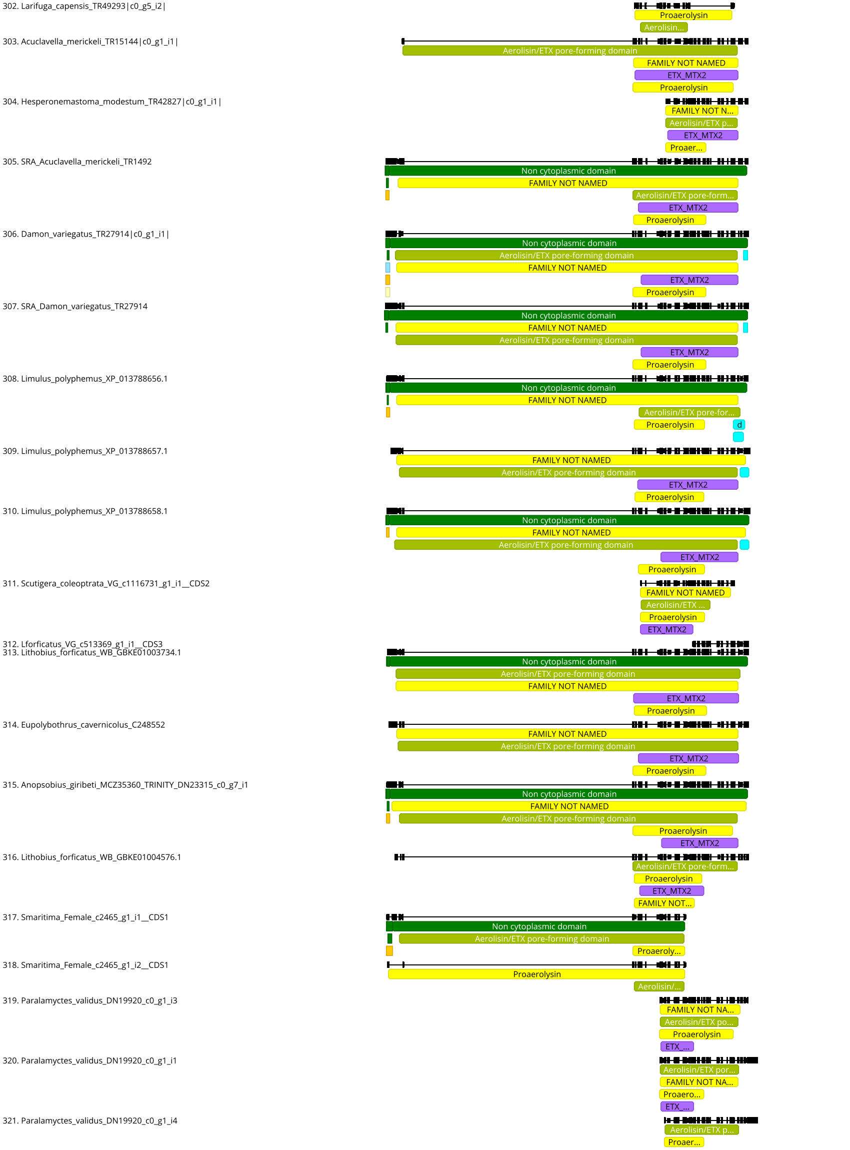
302. Larifuga_capensis_TR49293|c0_g5_i2|
Proaerolysin
Aerolisin...
303. Acuclavella_merickeli_TR15144|c0_g1_i1|
Aerolisin/ETX pore-forming domain
FAMILY NOT NAMED
ETX_MTX2
Proaerolysin
304. Hesperonemastoma_modestum_TR42827|c0_g1_i1|
FAMILY NOT N...
Aerolisin/ETX p...
ETX_MTX2
Proaer...
305. SRA_Acuclavella_merickeli_TR1492
Non cytoplasmic domain
FAMILY NOT NAMED
Aerolisin/ETX pore-form...
ETX_MTX2
Proaerolysin
306. Damon_variegatus_TR27914|c0_g1_i1|
Non cytoplasmic domain
Aerolisin/ETX pore-forming domain
FAMILY NOT NAMED
ETX_MTX2
Proaerolysin
307. SRA_Damon_variegatus_TR27914
Non cytoplasmic domain
FAMILY NOT NAMED
Aerolisin/ETX pore-forming domain
ETX_MTX2
Proaerolysin
308. Limulus_polyphemus_XP_013788656.1
Non cytoplasmic domain
FAMILY NOT NAMED
Aerolisin/ETX pore-for...
Proaerolysin
d
309. Limulus_polyphemus_XP_013788657.1
FAMILY NOT NAMED
Aerolisin/ETX pore-forming domain
ETX_MTX2
Proaerolysin
310. Limulus_polyphemus_XP_013788658.1
Non cytoplasmic domain
FAMILY NOT NAMED
Aerolisin/ETX pore-forming domain
ETX_MTX2
Proaerolysin
311. Scutigera_coleoptrata_VG_c1116731_g1_i1__CDS2
FAMILY NOT NAMED
Aerolisin/ETX ...
Proaerolysin
ETX_MTX2
312. Lforficatus_VG_c513369_g1_i1__CDS3
313. Lithobius_forficatus_WB_GBKE01003734.1
Non cytoplasmic domain
Aerolisin/ETX pore-forming domain
FAMILY NOT NAMED
ETX_MTX2
Proaerolysin
314. Eupolybothrus_cavernicolus_C248552
FAMILY NOT NAMED
Aerolisin/ETX pore-forming domain
ETX_MTX2
Proaerolysin
315. Anopsobius_giribeti_MCZ35360_TRINITY_DN23315_c0_g7_i1
Non cytoplasmic domain
FAMILY NOT NAMED
Aerolisin/ETX pore-forming domain
Proaerolysin
ETX_MTX2
316. Lithobius_forficatus_WB_GBKE01004576.1
Aerolisin/ETX pore-form...
Proaerolysin
ETX_MTX2
FAMILY NOT...
317. Smaritima_Female_c2465_g1_i1__CDS1
Non cytoplasmic domain
Aerolisin/ETX pore-forming domain
Proaeroly...
318. Smaritima_Female_c2465_g1_i2__CDS1
Proaerolysin
Aerolisin/...
319. Paralamyctes_validus_DN19920_c0_g1_i3
FAMILY NOT NA...
Aerolisin/ETX po...
Proaerolysin
ETX_...
320. Paralamyctes_validus_DN19920_c0_g1_i1
Aerolisin/ETX por...
FAMILY NOT NA...
Proaero...
ETX_...
321. Paralamyctes_validus_DN19920_c0_g1_i4
Aerolisin/ETX p...
Proaer...

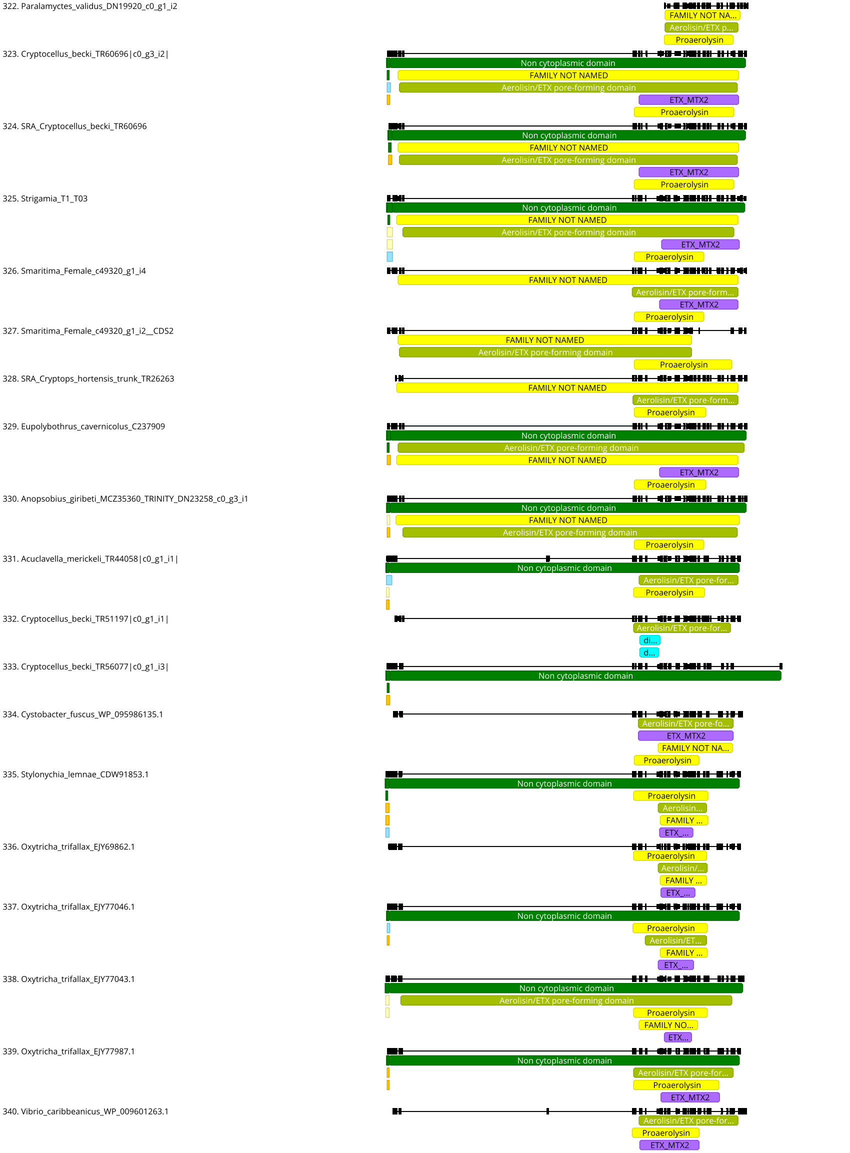
322. Paralamyctes_validus_DN19920_c0_g1_i2
FAMILY NOT NA...
Aerolisin/ETX p...
Proaerolysin
323. Cryptocellus_becki_TR60696|c0_g3_i2|
Non cytoplasmic domain
FAMILY NOT NAMED
Aerolisin/ETX pore-forming domain
ETX_MTX2
Proaerolysin
324. SRA_Cryptocellus_becki_TR60696
Non cytoplasmic domain
FAMILY NOT NAMED
Aerolisin/ETX pore-forming domain
ETX_MTX2
Proaerolysin
325. Strigamia_T1_T03
Non cytoplasmic domain
FAMILY NOT NAMED
Aerolisin/ETX pore-forming domain
ETX_MTX2
Proaerolysin
326. Smaritima_Female_c49320_g1_i4
FAMILY NOT NAMED
Aerolisin/ETX pore-form...
ETX_MTX2
Proaerolysin
327. Smaritima_Female_c49320_g1_i2__CDS2
FAMILY NOT NAMED
Aerolisin/ETX pore-forming domain
Proaerolysin
328. SRA_Cryptops_hortensis_trunk_TR26263
FAMILY NOT NAMED
Aerolisin/ETX pore-form...
Proaerolysin
329. Eupolybothrus_cavernicolus_C237909
Non cytoplasmic domain
Aerolisin/ETX pore-forming domain
FAMILY NOT NAMED
ETX_MTX2
Proaerolysin
330. Anopsobius_giribeti_MCZ35360_TRINITY_DN23258_c0_g3_i1
Non cytoplasmic domain
FAMILY NOT NAMED
Aerolisin/ETX pore-forming domain
Proaerolysin
331. Acuclavella_merickeli_TR44058|c0_g1_i1|
Non cytoplasmic domain
Aerolisin/ETX pore-for...
Proaerolysin
332. Cryptocellus_becki_TR51197|c0_g1_i1|
Aerolisin/ETX pore-for...
di...
d...
333. Cryptocellus_becki_TR56077|c0_g1_i3|
Non cytoplasmic domain
334. Cystobacter_fuscus_WP_095986135.1
Aerolisin/ETX pore-fo...
ETX_MTX2
FAMILY NOT NA...
Proaerolysin
335. Stylonychia_lemnae_CDW91853.1
Non cytoplasmic domain
Proaerolysin
Aerolisin...
FAMILY ...
ETX_...
336. Oxytricha_trifallax_EJY69862.1
Proaerolysin
Aerolisin/...
FAMILY ...
ETX_...
337. Oxytricha_trifallax_EJY77046.1
Non cytoplasmic domain
Proaerolysin
Aerolisin/ET...
FAMILY ...
ETX_...
338. Oxytricha_trifallax_EJY77043.1
Non cytoplasmic domain
Aerolisin/ETX pore-forming domain
Proaerolysin
FAMILY NO...
ETX...
339. Oxytricha_trifallax_EJY77987.1
Non cytoplasmic domain
Aerolisin/ETX pore-for...
Proaerolysin
ETX_MTX2
340. Vibrio_caribbeanicus_WP_009601263.1
Aerolisin/ETX pore-for...
Proaerolysin
ETX_MTX2

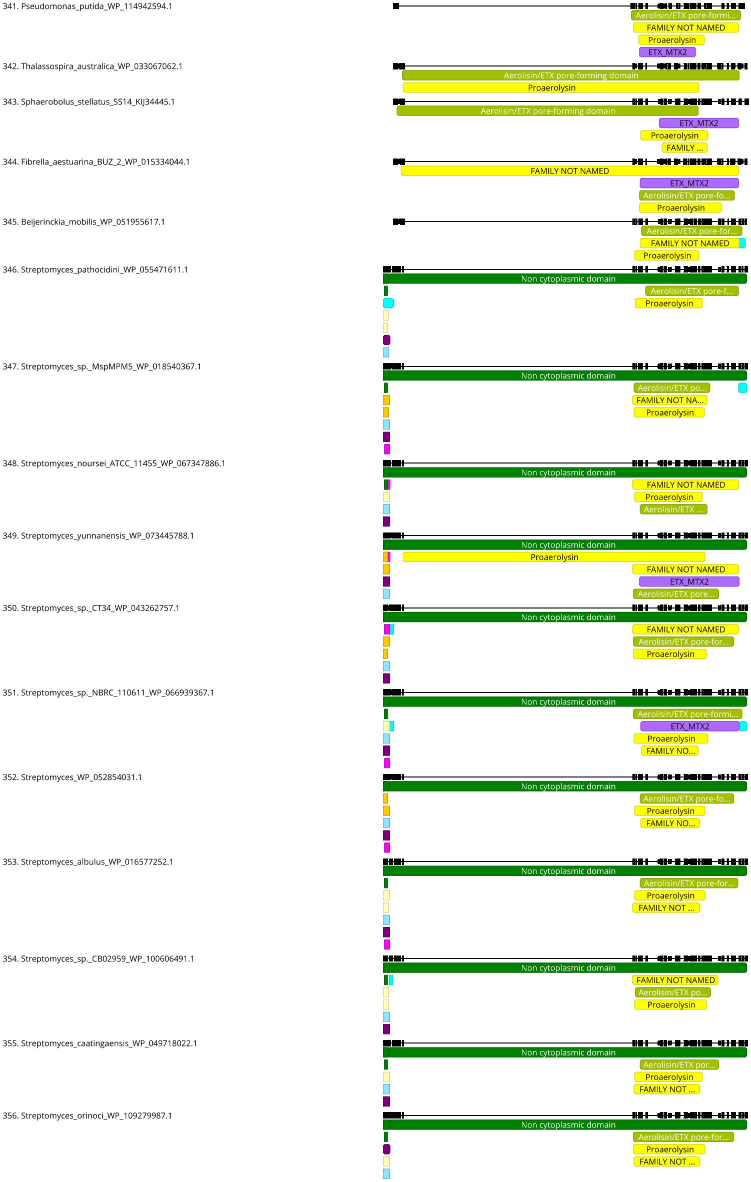

341. Pseudomonas_putida_WP_114942594.1
Aerolisin/ETX pore-formi...
FAMILY NOT NAMED
Proaerolysin
ETX_MTX2
342. Thalassospira_australica_WP_033067062.1
Aerolisin/ETX pore-forming domain
Proaerolysin
343. Sphaerobolus_stellatus_SS14_KIJ34445.1
Aerolisin/ETX pore-forming domain
ETX_MTX2
Proaerolysin
FAMILY ...
344. Fibrella_aestuarina_BUZ_2_WP_015334044.1
FAMILY NOT NAMED
ETX_MTX2
Aerolisin/ETX pore-fo...
Proaerolysin
345. Beijerinckia_mobilis_WP_051955617.1
Aerolisin/ETX pore-for...
FAMILY NOT NAMED
Proaerolysin
346. Streptomyces_pathocidini_WP_055471611.1
Non cytoplasmic domain
Aerolisin/ETX pore-f...
Proaerolysin
347. Streptomyces_sp._MspMPM5_WP_018540367.1
Non cytoplasmic domain
Aerolisin/ETX po...
FAMILY NOT NA...
Proaerolysin
348. Streptomyces_noursei_ATCC_11455_WP_067347886.1
Non cytoplasmic domain
FAMILY NOT NAMED
Proaerolysin
Aerolisin/ETX ...
349. Streptomyces_yunnanensis_WP_073445788.1
Non cytoplasmic domain
Proaerolysin
FAMILY NOT NAMED
ETX_MTX2
Aerolisin/ETX pore...
350. Streptomyces_sp._CT34_WP_043262757.1
Non cytoplasmic domain
FAMILY NOT NAMED
Aerolisin/ETX pore-for...
Proaerolysin
351. Streptomyces_sp._NBRC_110611_WP_066939367.1
Non cytoplasmic domain
Aerolisin/ETX pore-formi...
ETX_MTX2
Proaerolysin
FAMILY NO...
352. Streptomyces_WP_052854031.1
Non cytoplasmic domain
Aerolisin/ETX pore-fo...
Proaerolysin
FAMILY NO...
353. Streptomyces_albulus_WP_016577252.1
Non cytoplasmic domain
Aerolisin/ETX pore-for...
Proaerolysin
FAMILY NOT ...
354. Streptomyces_sp._CB02959_WP_100606491.1
Non cytoplasmic domain
FAMILY NOT NAMED
Aerolisin/ETX po...
Proaerolysin
355. Streptomyces_caatingaensis_WP_049718022.1
Non cytoplasmic domain
Aerolisin/ETX por...
Proaerolysin
FAMILY NOT ...
356. Streptomyces_orinoci_WP_109279987.1
Non cytoplasmic domain
Aerolisin/ETX pore-for...
Proaerolysin
FAMILY NOT ...

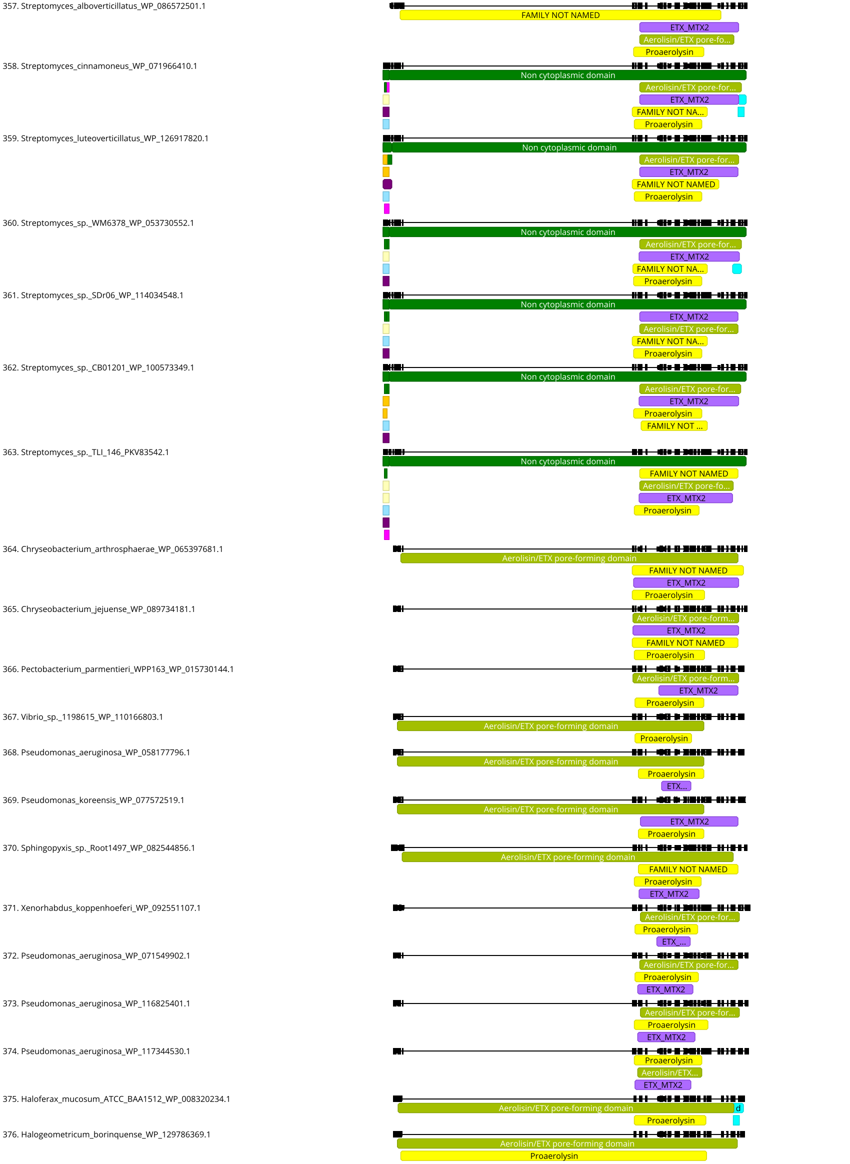
357. Streptomyces_alboverticillatus_WP_086572501.1
FAMILY NOT NAMED
ETX_MTX2
Aerolisin/ETX pore-fo...
Proaerolysin
358. Streptomyces_cinnamoneus_WP_071966410.1
Non cytoplasmic domain
Aerolisin/ETX pore-for...
ETX_MTX2
FAMILY NOT NA...
Proaerolysin
359. Streptomyces_luteoverticillatus_WP_126917820.1
Non cytoplasmic domain
Aerolisin/ETX pore-for...
ETX_MTX2
FAMILY NOT NAMED
Proaerolysin
360. Streptomyces_sp._WM6378_WP_053730552.1
Non cytoplasmic domain
Aerolisin/ETX pore-for...
ETX_MTX2
FAMILY NOT NA...
Proaerolysin
361. Streptomyces_sp._SDr06_WP_114034548.1
Non cytoplasmic domain
ETX_MTX2
Aerolisin/ETX pore-for...
FAMILY NOT NA...
Proaerolysin
362. Streptomyces_sp._CB01201_WP_100573349.1
Non cytoplasmic domain
Aerolisin/ETX pore-for...
ETX_MTX2
Proaerolysin
FAMILY NOT ...
363. Streptomyces_sp._TLI_146_PKV83542.1
Non cytoplasmic domain
FAMILY NOT NAMED
Aerolisin/ETX pore-fo...
ETX_MTX2
Proaerolysin
364. Chryseobacterium_arthrosphaerae_WP_065397681.1
Aerolisin/ETX pore-forming domain
FAMILY NOT NAMED
ETX_MTX2
Proaerolysin
365. Chryseobacterium_jejuense_WP_089734181.1
Aerolisin/ETX pore-form...
ETX_MTX2
FAMILY NOT NAMED
Proaerolysin
366. Pectobacterium_parmentieri_WPP163_WP_015730144.1
Aerolisin/ETX pore-form...
ETX_MTX2
Proaerolysin
367. Vibrio_sp._1198615_WP_110166803.1
Aerolisin/ETX pore-forming domain
Proaerolysin
368. Pseudomonas_aeruginosa_WP_058177796.1
Aerolisin/ETX pore-forming domain
Proaerolysin
ETX...
369. Pseudomonas_koreensis_WP_077572519.1
Aerolisin/ETX pore-forming domain
ETX_MTX2
Proaerolysin
370. Sphingopyxis_sp._Root1497_WP_082544856.1
Aerolisin/ETX pore-forming domain
FAMILY NOT NAMED
Proaerolysin
ETX_MTX2
371. Xenorhabdus_koppenhoeferi_WP_092551107.1
Aerolisin/ETX pore-for...
Proaerolysin
ETX_...
372. Pseudomonas_aeruginosa_WP_071549902.1
Aerolisin/ETX pore-for...
Proaerolysin
ETX_MTX2
373. Pseudomonas_aeruginosa_WP_116825401.1
Aerolisin/ETX pore-for...
Proaerolysin
ETX_MTX2
374. Pseudomonas_aeruginosa_WP_117344530.1
Proaerolysin
Aerolisin/ETX...
ETX_MTX2
375. Haloferax_mucosum_ATCC_BAA1512_WP_008320234.1
Aerolisin/ETX pore-forming domain
d
Proaerolysin
376. Halogeometricum_borinquense_WP_129786369.1
Aerolisin/ETX pore-forming domain
Proaerolysin

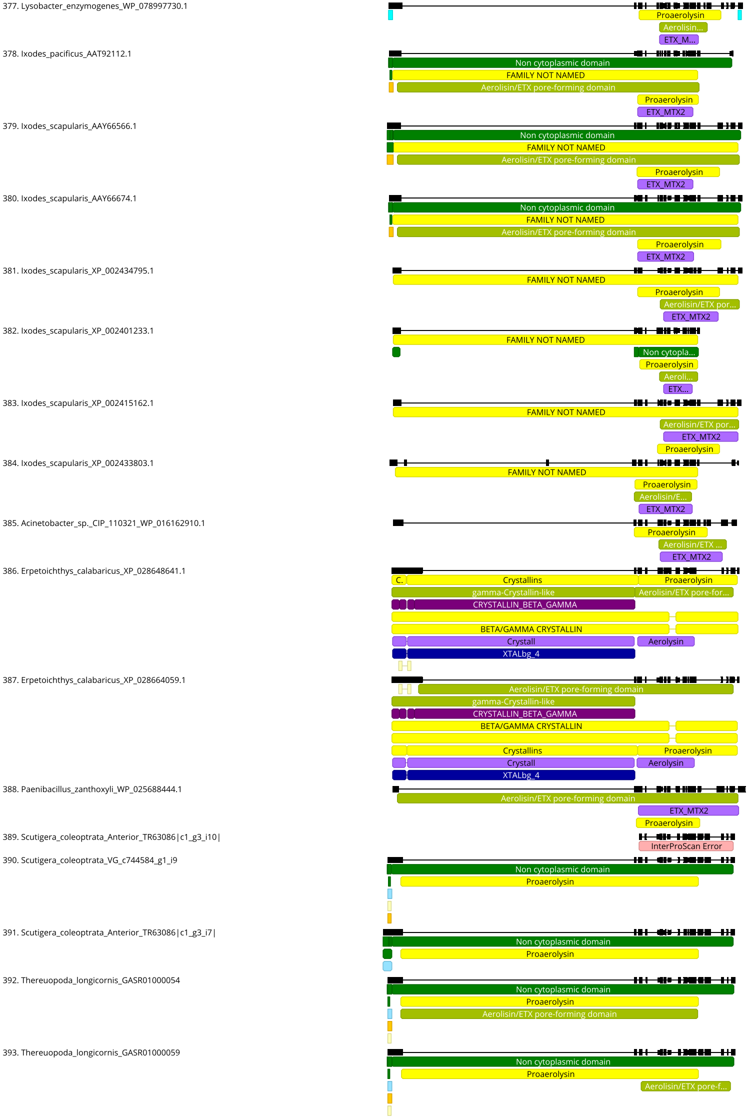
377. Lysobacter_enzymogenes_WP_078997730.1
Proaerolysin
Aerolisin...
ETX_M...
378. Ixodes_pacificus_AAT92112.1
Non cytoplasmic domain
FAMILY NOT NAMED
Aerolisin/ETX pore-forming domain
Proaerolysin
ETX_MTX2
379. Ixodes_scapularis_AAY66566.1
Non cytoplasmic domain
FAMILY NOT NAMED
Aerolisin/ETX pore-forming domain
Proaerolysin
ETX_MTX2
380. Ixodes_scapularis_AAY66674.1
Non cytoplasmic domain
FAMILY NOT NAMED
Aerolisin/ETX pore-forming domain
Proaerolysin
ETX_MTX2
381. Ixodes_scapularis_XP_002434795.1
FAMILY NOT NAMED
Proaerolysin
Aerolisin/ETX por...
ETX_MTX2
382. Ixodes_scapularis_XP_002401233.1
FAMILY NOT NAMED
Non cytopla...
Proaerolysin
Aeroli...
ETX...
383. Ixodes_scapularis_XP_002415162.1
FAMILY NOT NAMED
Aerolisin/ETX por...
ETX_MTX2
Proaerolysin
384. Ixodes_scapularis_XP_002433803.1
FAMILY NOT NAMED
Proaerolysin
Aerolisin/E...
ETX_MTX2
385. Acinetobacter_sp._CIP_110321_WP_016162910.1
Proaerolysin
Aerolisin/ETX ...
ETX_MTX2
386. Erpetoichthys_calabaricus_XP_028648641.1
C.
Crystallins
Proaerolysin
gamma-Crystallin-like
Aerolisin/ETX pore-for...
CRYSTALLIN_BETA_GAMMA
BETA/GAMMA CRYSTALLIN
Crystall
Aerolysin
XTALbg_4
387. Erpetoichthys_calabaricus_XP_028664059.1
Aerolisin/ETX pore-forming domain
gamma-Crystallin-like
CRYSTALLIN_BETA_GAMMA
BETA/GAMMA CRYSTALLIN
Crystallins
Proaerolysin
Crystall
Aerolysin
XTALbg_4
388. Paenibacillus_zanthoxyli_WP_025688444.1
Aerolisin/ETX pore-forming domain
ETX_MTX2
Proaerolysin
389. Scutigera_coleoptrata_Anterior_TR63086|c1_g3_i10|
InterProScan Error
390. Scutigera_coleoptrata_VG_c744584_g1_i9
Non cytoplasmic domain
Proaerolysin
391. Scutigera_coleoptrata_Anterior_TR63086|c1_g3_i7|
Non cytoplasmic domain
Proaerolysin
392. Thereuopoda_longicornis_GASR01000054
Non cytoplasmic domain
Proaerolysin
Aerolisin/ETX pore-forming domain
393. Thereuopoda_longicornis_GASR01000059
Non cytoplasmic domain
Proaerolysin
Aerolisin/ETX pore-f...

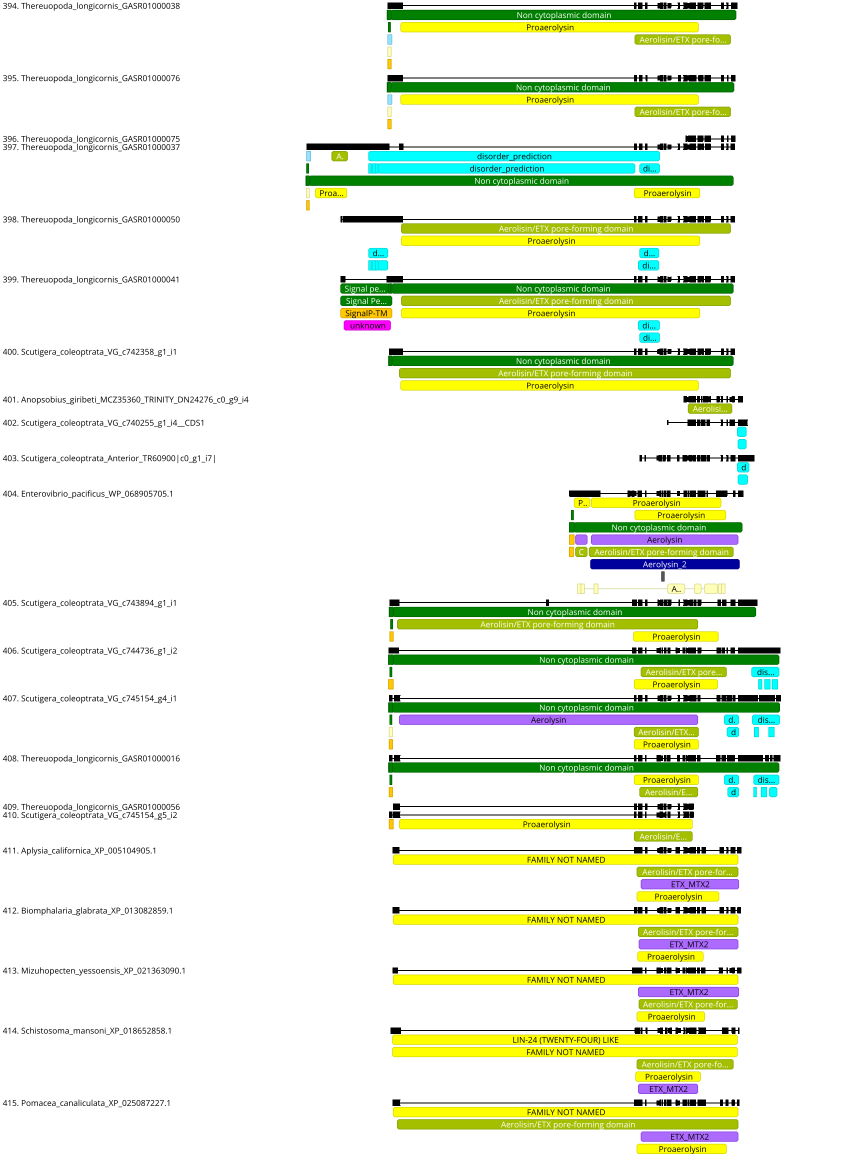

394. Thereuopoda_longicornis_GASR01000038
Non cytoplasmic domain
Proaerolysin
Aerolisin/ETX pore-fo...
395. Thereuopoda_longicornis_GASR01000076
Non cytoplasmic domain
Proaerolysin
Aerolisin/ETX pore-fo...
396. Thereuopoda_longicornis_GASR01000075
397. Thereuopoda_longicornis_GASR01000037
A.
disorder_prediction
disorder_prediction
di...
Non cytoplasmic domain
Proa...
Proaerolysin
398. Thereuopoda_longicornis_GASR01000050
Aerolisin/ETX pore-forming domain
Proaerolysin
d...
d...
di...
399. Thereuopoda_longicornis_GASR01000041
Signal pe...
Non cytoplasmic domain
Signal Pe...
Aerolisin/ETX pore-forming domain
SignalP-TM
Proaerolysin
unknown
di...
di...
400. Scutigera_coleoptrata_VG_c742358_g1_i1
Non cytoplasmic domain
Aerolisin/ETX pore-forming domain
Proaerolysin
401. Anopsobius_giribeti_MCZ35360_TRINITY_DN24276_c0_g9_i4
Aerolisi...
402. Scutigera_coleoptrata_VG_c740255_g1_i4__CDS1
403. Scutigera_coleoptrata_Anterior_TR60900|c0_g1_i7|
d
404. Enterovibrio_pacificus_WP_068905705.1
P..
Proaerolysin
Proaerolysin
Non cytoplasmic domain
Aerolysin
C
Aerolisin/ETX pore-forming domain
Aerolysin_2
A..
405. Scutigera_coleoptrata_VG_c743894_g1_i1
Non cytoplasmic domain
Aerolisin/ETX pore-forming domain
Proaerolysin
406. Scutigera_coleoptrata_VG_c744736_g1_i2
Non cytoplasmic domain
Aerolisin/ETX pore...
dis...
Proaerolysin
407. Scutigera_coleoptrata_VG_c745154_g4_i1
Non cytoplasmic domain
Aerolysin
d.
dis...
Aerolisin/ETX...
d
Proaerolysin
408. Thereuopoda_longicornis_GASR01000016
Non cytoplasmic domain
Proaerolysin
d.
dis...
Aerolisin/E...
d
409. Thereuopoda_longicornis_GASR01000056
410. Scutigera_coleoptrata_VG_c745154_g5_i2
Proaerolysin
Aerolisin/E...
411. Aplysia_californica_XP_005104905.1
FAMILY NOT NAMED
Aerolisin/ETX pore-for...
ETX_MTX2
Proaerolysin
412. Biomphalaria_glabrata_XP_013082859.1
FAMILY NOT NAMED
Aerolisin/ETX pore-for...
ETX_MTX2
Proaerolysin
413. Mizuhopecten_yessoensis_XP_021363090.1
FAMILY NOT NAMED
ETX_MTX2
Aerolisin/ETX pore-for...
Proaerolysin
414. Schistosoma_mansoni_XP_018652858.1
LIN-24 (TWENTY-FOUR) LIKE
FAMILY NOT NAMED
Aerolisin/ETX pore-fo...
Proaerolysin
ETX_MTX2
415. Pomacea_canaliculata_XP_025087227.1
FAMILY NOT NAMED
Aerolisin/ETX pore-forming domain
ETX_MTX2
Proaerolysin

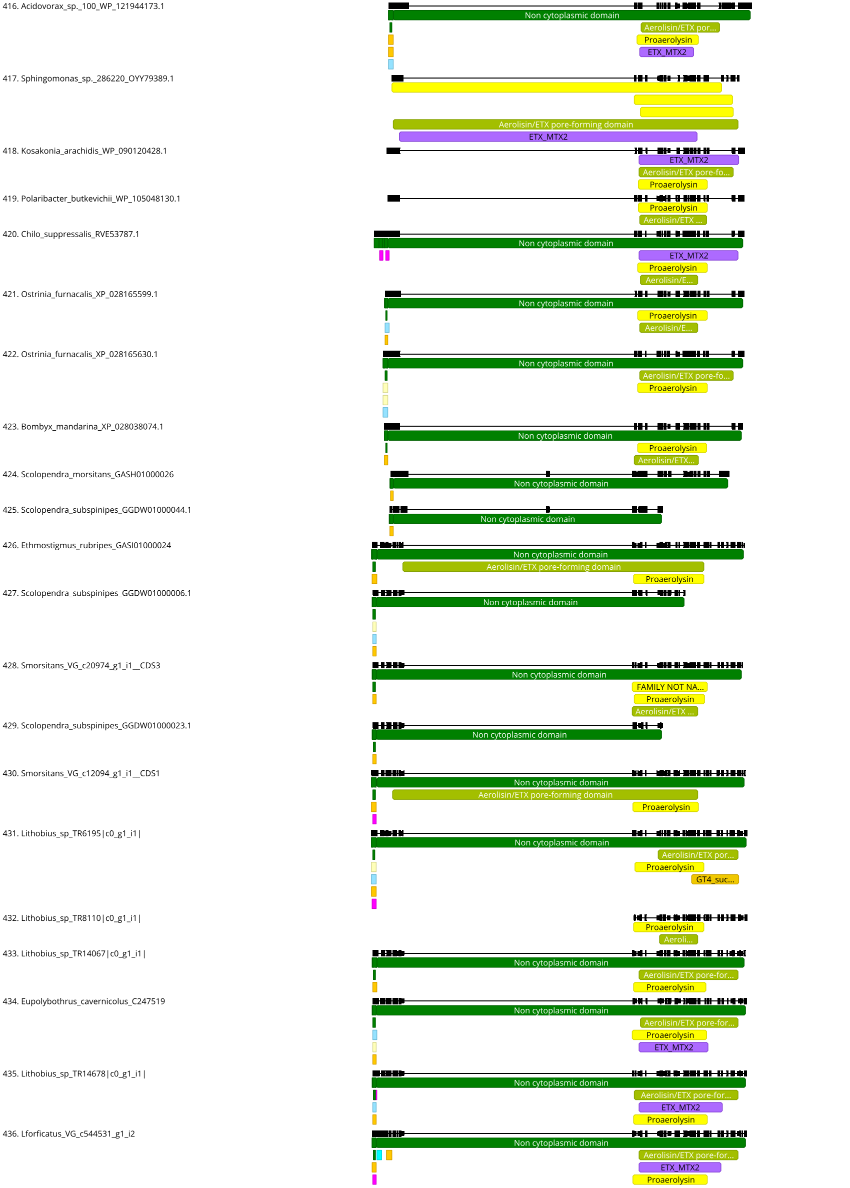

416. Acidovorax_sp._100_WP_121944173.1
Non cytoplasmic domain
Aerolisin/ETX por...
Proaerolysin
ETX_MTX2
417. Sphingomonas_sp._286220_OYY79389.1
Aerolisin/ETX pore-forming domain
ETX_MTX2
418. Kosakonia_arachidis_WP_090120428.1
ETX_MTX2
Aerolisin/ETX pore-fo...
Proaerolysin
419. Polaribacter_butkevichii_WP_105048130.1
Proaerolysin
Aerolisin/ETX ...
420. Chilo_suppressalis_RVE53787.1
Non cytoplasmic domain
ETX_MTX2
Proaerolysin
Aerolisin/E...
421. Ostrinia_furnacalis_XP_028165599.1
Non cytoplasmic domain
Proaerolysin
Aerolisin/E...
422. Ostrinia_furnacalis_XP_028165630.1
Non cytoplasmic domain
Aerolisin/ETX pore-fo...
Proaerolysin
423. Bombyx_mandarina_XP_028038074.1
Non cytoplasmic domain
Proaerolysin
Aerolisin/ETX...
424. Scolopendra_morsitans_GASH01000026
Non cytoplasmic domain
425. Scolopendra_subspinipes_GGDW01000044.1
Non cytoplasmic domain
426. Ethmostigmus_rubripes_GASI01000024
Non cytoplasmic domain
Aerolisin/ETX pore-forming domain
Proaerolysin
427. Scolopendra_subspinipes_GGDW01000006.1
Non cytoplasmic domain
428. Smorsitans_VG_c20974_g1_i1__CDS3
Non cytoplasmic domain
FAMILY NOT NA...
Proaerolysin
Aerolisin/ETX ...
429. Scolopendra_subspinipes_GGDW01000023.1
Non cytoplasmic domain
430. Smorsitans_VG_c12094_g1_i1__CDS1
Non cytoplasmic domain
Aerolisin/ETX pore-forming domain
Proaerolysin
431. Lithobius_sp_TR6195|c0_g1_i1|
Non cytoplasmic domain
Aerolisin/ETX por...
Proaerolysin
GT4_suc...
432. Lithobius_sp_TR8110|c0_g1_i1|
Proaerolysin
Aeroli...
433. Lithobius_sp_TR14067|c0_g1_i1|
Non cytoplasmic domain
Aerolisin/ETX pore-for...
Proaerolysin
434. Eupolybothrus_cavernicolus_C247519
Non cytoplasmic domain
Aerolisin/ETX pore-for...
Proaerolysin
ETX_MTX2
435. Lithobius_sp_TR14678|c0_g1_i1|
Non cytoplasmic domain
Aerolisin/ETX pore-for...
ETX_MTX2
Proaerolysin
436. Lforficatus_VG_c544531_g1_i2
Non cytoplasmic domain
Aerolisin/ETX pore-for...
ETX_MTX2
Proaerolysin

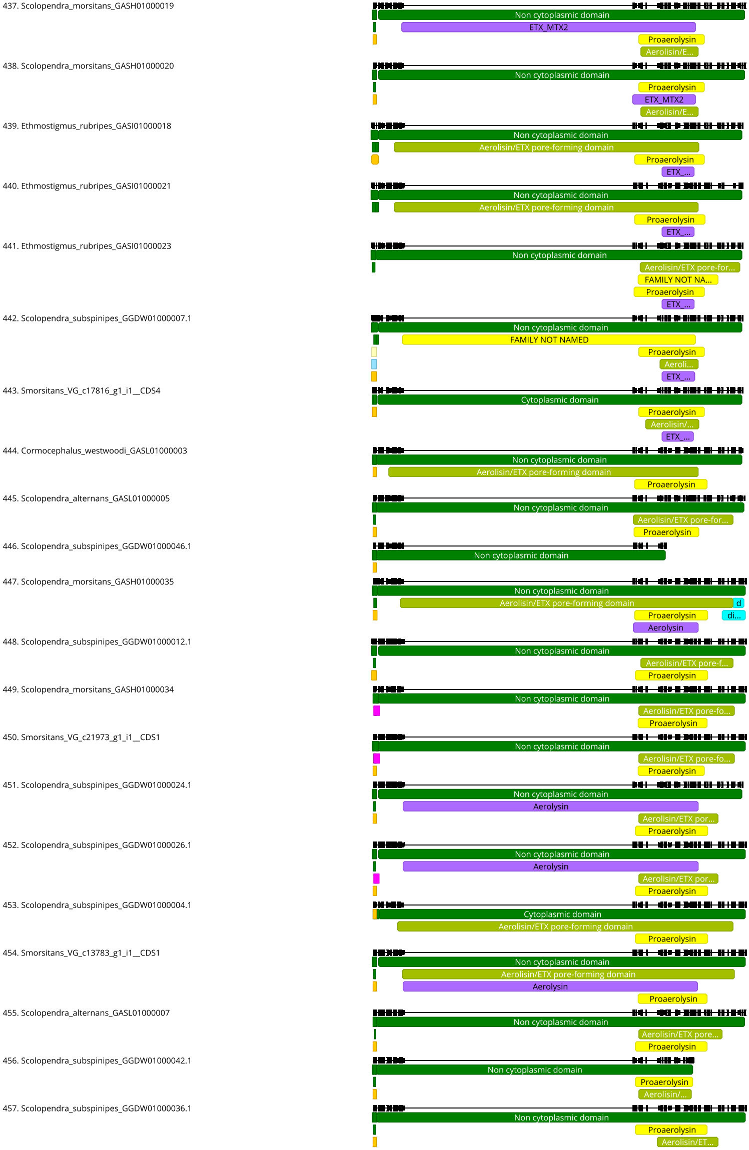
437. Scolopendra_morsitans_GASH01000019
Non cytoplasmic domain
ETX_MTX2
Proaerolysin
Aerolisin/E...
438. Scolopendra_morsitans_GASH01000020
Non cytoplasmic domain
Proaerolysin
ETX_MTX2
Aerolisin/E...
439. Ethmostigmus_rubripes_GASI01000018
Non cytoplasmic domain
Aerolisin/ETX pore-forming domain
Proaerolysin
ETX_...
440. Ethmostigmus_rubripes_GASI01000021
Non cytoplasmic domain
Aerolisin/ETX pore-forming domain
Proaerolysin
ETX_...
441. Ethmostigmus_rubripes_GASI01000023
Non cytoplasmic domain
Aerolisin/ETX pore-for...
FAMILY NOT NA...
Proaerolysin
ETX_...
442. Scolopendra_subspinipes_GGDW01000007.1
Non cytoplasmic domain
FAMILY NOT NAMED
Proaerolysin
Aeroli...
ETX_...
443. Smorsitans_VG_c17816_g1_i1__CDS4
Cytoplasmic domain
Proaerolysin
Aerolisin/...
ETX_...
444. Cormocephalus_westwoodi_GASL01000003
Non cytoplasmic domain
Aerolisin/ETX pore-forming domain
Proaerolysin
445. Scolopendra_alternans_GASL01000005
Non cytoplasmic domain
Aerolisin/ETX pore-for...
Proaerolysin
446. Scolopendra_subspinipes_GGDW01000046.1
Non cytoplasmic domain
447. Scolopendra_morsitans_GASH01000035
Non cytoplasmic domain
Aerolisin/ETX pore-forming domain
d
Proaerolysin
di...
Aerolysin
448. Scolopendra_subspinipes_GGDW01000012.1
Non cytoplasmic domain
Aerolisin/ETX pore-f...
Proaerolysin
449. Scolopendra_morsitans_GASH01000034
Non cytoplasmic domain
Aerolisin/ETX pore-fo...
Proaerolysin
450. Smorsitans_VG_c21973_g1_i1__CDS1
Non cytoplasmic domain
Aerolisin/ETX pore-fo...
Proaerolysin
451. Scolopendra_subspinipes_GGDW01000024.1
Non cytoplasmic domain
Aerolysin
Aerolisin/ETX por...
Proaerolysin
452. Scolopendra_subspinipes_GGDW01000026.1
Non cytoplasmic domain
Aerolysin
Aerolisin/ETX por...
Proaerolysin
453. Scolopendra_subspinipes_GGDW01000004.1
Cytoplasmic domain
Aerolisin/ETX pore-forming domain
Proaerolysin
454. Smorsitans_VG_c13783_g1_i1__CDS1
Non cytoplasmic domain
Aerolisin/ETX pore-forming domain
Aerolysin
Proaerolysin
455. Scolopendra_alternans_GASL01000007
Non cytoplasmic domain
Aerolisin/ETX pore...
Proaerolysin
456. Scolopendra_subspinipes_GGDW01000042.1
Non cytoplasmic domain
Proaerolysin
Aerolisin/...
457. Scolopendra_subspinipes_GGDW01000036.1
Non cytoplasmic domain
Proaerolysin
Aerolisin/ET...

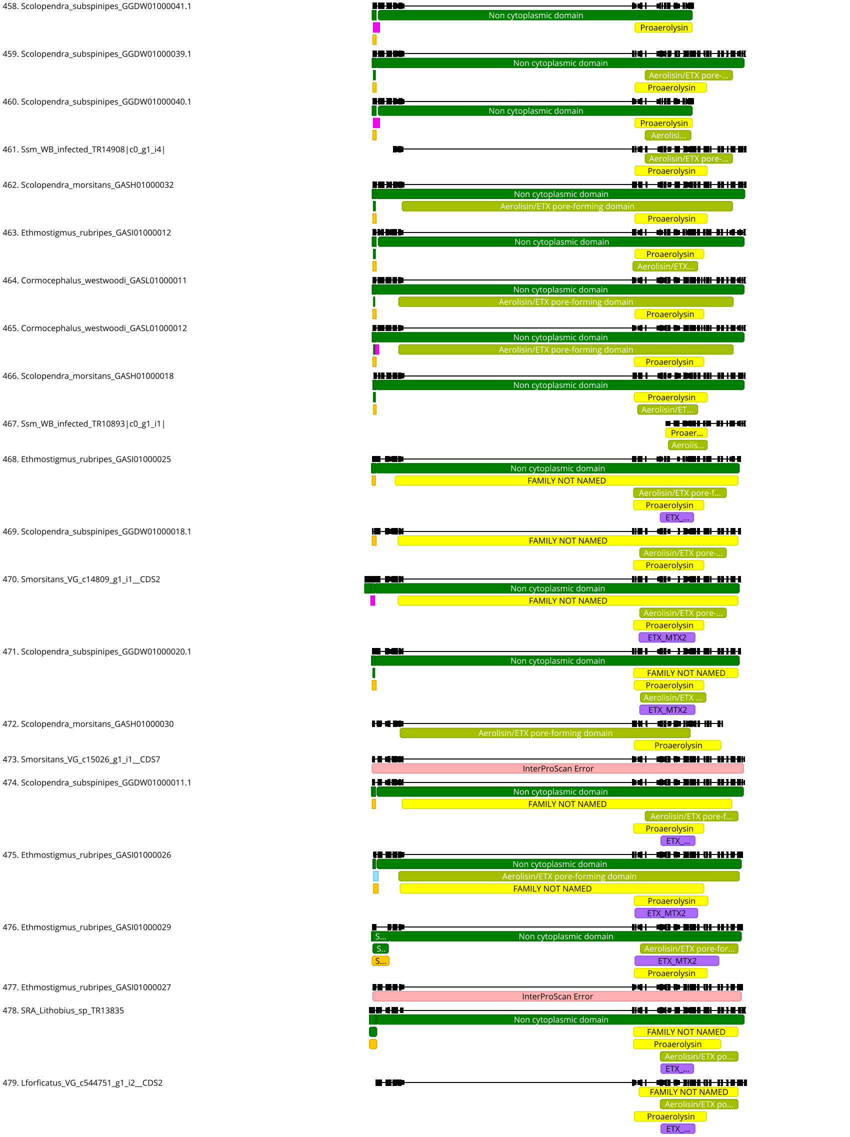
458. Scolopendra_subspinipes_GGDW01000041.1
Non cytoplasmic domain
Proaerolysin
459. Scolopendra_subspinipes_GGDW01000039.1
Non cytoplasmic domain
Aerolisin/ETX pore-...
Proaerolysin
460. Scolopendra_subspinipes_GGDW01000040.1
Non cytoplasmic domain
Proaerolysin
Aerolisi...
461. Ssm_WB_infected_TR14908|c0_g1_i4|
Aerolisin/ETX pore-...
Proaerolysin
462. Scolopendra_morsitans_GASH01000032
Non cytoplasmic domain
Aerolisin/ETX pore-forming domain
Proaerolysin
463. Ethmostigmus_rubripes_GASI01000012
Non cytoplasmic domain
Proaerolysin
Aerolisin/ETX...
464. Cormocephalus_westwoodi_GASL01000011
Non cytoplasmic domain
Aerolisin/ETX pore-forming domain
Proaerolysin
465. Cormocephalus_westwoodi_GASL01000012
Non cytoplasmic domain
Aerolisin/ETX pore-forming domain
Proaerolysin
466. Scolopendra_morsitans_GASH01000018
Non cytoplasmic domain
Proaerolysin
Aerolisin/ET...
467. Ssm_WB_infected_TR10893|c0_g1_i1|
Proaer...
Aerolis...
468. Ethmostigmus_rubripes_GASI01000025
Non cytoplasmic domain
FAMILY NOT NAMED
Aerolisin/ETX pore-f...
Proaerolysin
ETX_...
469. Scolopendra_subspinipes_GGDW01000018.1
FAMILY NOT NAMED
Aerolisin/ETX pore-...
Proaerolysin
470. Smorsitans_VG_c14809_g1_i1__CDS2
Non cytoplasmic domain
FAMILY NOT NAMED
Aerolisin/ETX pore-...
Proaerolysin
ETX_MTX2
471. Scolopendra_subspinipes_GGDW01000020.1
Non cytoplasmic domain
FAMILY NOT NAMED
Proaerolysin
Aerolisin/ETX ...
ETX_MTX2
472. Scolopendra_morsitans_GASH01000030
Aerolisin/ETX pore-forming domain
Proaerolysin
473. Smorsitans_VG_c15026_g1_i1__CDS7
InterProScan Error
474. Scolopendra_subspinipes_GGDW01000011.1
Non cytoplasmic domain
FAMILY NOT NAMED
Aerolisin/ETX pore-f...
Proaerolysin
ETX_...
475. Ethmostigmus_rubripes_GASI01000026
Non cytoplasmic domain
Aerolisin/ETX pore-forming domain
FAMILY NOT NAMED
Proaerolysin
ETX_MTX2
476. Ethmostigmus_rubripes_GASI01000029
S...
Non cytoplasmic domain
S..
Aerolisin/ETX pore-for...
S...
ETX_MTX2
Proaerolysin
477. Ethmostigmus_rubripes_GASI01000027
InterProScan Error
478. SRA_Lithobius_sp_TR13835
Non cytoplasmic domain
FAMILY NOT NAMED
Proaerolysin
Aerolisin/ETX po...
ETX_...
479. Lforficatus_VG_c544751_g1_i2__CDS2
FAMILY NOT NAMED
Aerolisin/ETX po...
Proaerolysin
ETX_...

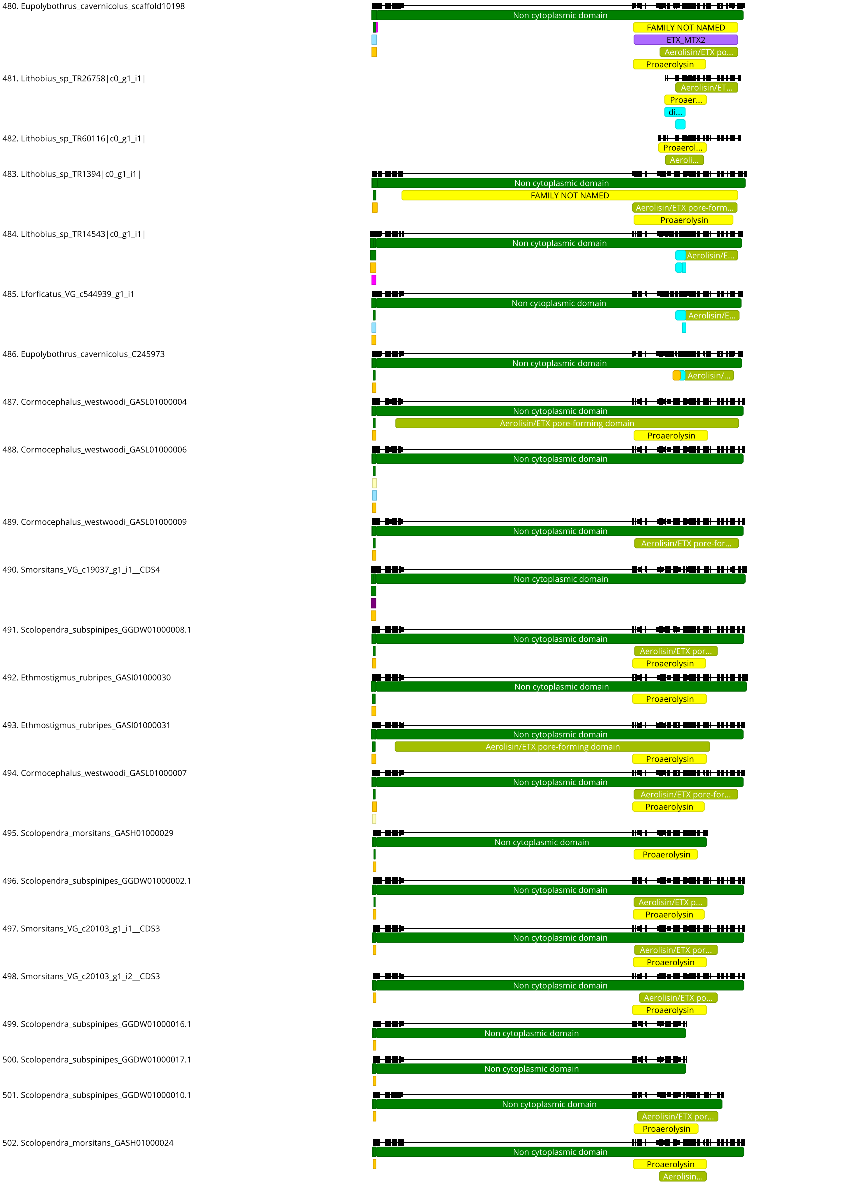
480. Eupolybothrus_cavernicolus_scaffold10198
Non cytoplasmic domain
FAMILY NOT NAMED
ETX_MTX2
Aerolisin/ETX po...
Proaerolysin
481. Lithobius_sp_TR26758|c0_g1_i1|
Aerolisin/ET...
Proaer...
di...
482. Lithobius_sp_TR60116|c0_g1_i1|
Proaerol...
Aeroli...
483. Lithobius_sp_TR1394|c0_g1_i1|
Non cytoplasmic domain
FAMILY NOT NAMED
Aerolisin/ETX pore-form...
Proaerolysin
484. Lithobius_sp_TR14543|c0_g1_i1|
Non cytoplasmic domain
Aerolisin/E...
485. Lforficatus_VG_c544939_g1_i1
Non cytoplasmic domain
Aerolisin/E...
486. Eupolybothrus_cavernicolus_C245973
Non cytoplasmic domain
Aerolisin/...
487. Cormocephalus_westwoodi_GASL01000004
Non cytoplasmic domain
Aerolisin/ETX pore-forming domain
Proaerolysin
488. Cormocephalus_westwoodi_GASL01000006
Non cytoplasmic domain
489. Cormocephalus_westwoodi_GASL01000009
Non cytoplasmic domain
Aerolisin/ETX pore-for...
490. Smorsitans_VG_c19037_g1_i1__CDS4
Non cytoplasmic domain
491. Scolopendra_subspinipes_GGDW01000008.1
Non cytoplasmic domain
Aerolisin/ETX por...
Proaerolysin
492. Ethmostigmus_rubripes_GASI01000030
Non cytoplasmic domain
Proaerolysin
493. Ethmostigmus_rubripes_GASI01000031
Non cytoplasmic domain
Aerolisin/ETX pore-forming domain
Proaerolysin
494. Cormocephalus_westwoodi_GASL01000007
Non cytoplasmic domain
Aerolisin/ETX pore-for...
Proaerolysin
495. Scolopendra_morsitans_GASH01000029
Non cytoplasmic domain
Proaerolysin
496. Scolopendra_subspinipes_GGDW01000002.1
Non cytoplasmic domain
Aerolisin/ETX p...
Proaerolysin
497. Smorsitans_VG_c20103_g1_i1__CDS3
Non cytoplasmic domain
Aerolisin/ETX por...
Proaerolysin
498. Smorsitans_VG_c20103_g1_i2__CDS3
Non cytoplasmic domain
Aerolisin/ETX po...
Proaerolysin
499. Scolopendra_subspinipes_GGDW01000016.1
Non cytoplasmic domain
500. Scolopendra_subspinipes_GGDW01000017.1
Non cytoplasmic domain
501. Scolopendra_subspinipes_GGDW01000010.1
Non cytoplasmic domain
Aerolisin/ETX por...
Proaerolysin
502. Scolopendra_morsitans_GASH01000024
Non cytoplasmic domain
Proaerolysin
Aerolisin...

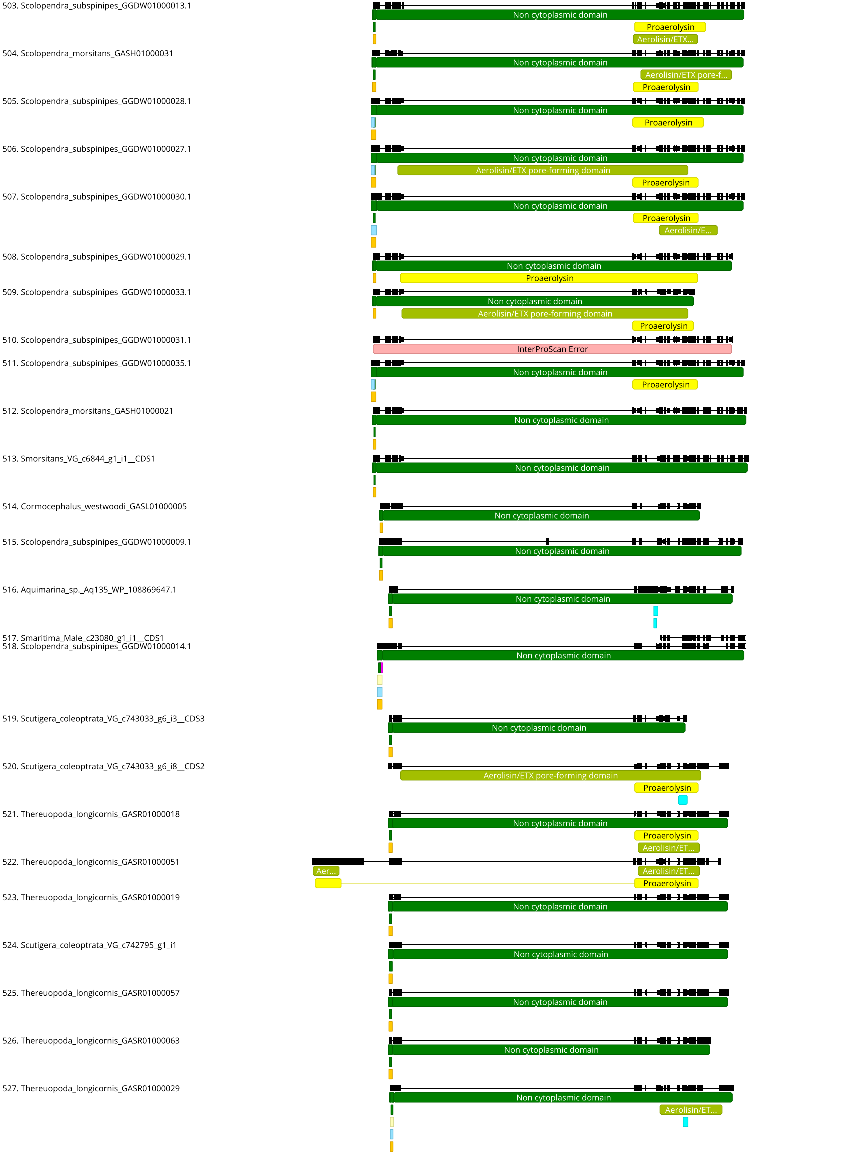
503. Scolopendra_subspinipes_GGDW01000013.1
Non cytoplasmic domain
Proaerolysin
Aerolisin/ETX...
504. Scolopendra_morsitans_GASH01000031
Non cytoplasmic domain
Aerolisin/ETX pore-f...
Proaerolysin
505. Scolopendra_subspinipes_GGDW01000028.1
Non cytoplasmic domain
Proaerolysin
506. Scolopendra_subspinipes_GGDW01000027.1
Non cytoplasmic domain
Aerolisin/ETX pore-forming domain
Proaerolysin
507. Scolopendra_subspinipes_GGDW01000030.1
Non cytoplasmic domain
Proaerolysin
Aerolisin/E...
508. Scolopendra_subspinipes_GGDW01000029.1
Non cytoplasmic domain
Proaerolysin
509. Scolopendra_subspinipes_GGDW01000033.1
Non cytoplasmic domain
Aerolisin/ETX pore-forming domain
Proaerolysin
510. Scolopendra_subspinipes_GGDW01000031.1
InterProScan Error
511. Scolopendra_subspinipes_GGDW01000035.1
Non cytoplasmic domain
Proaerolysin
512. Scolopendra_morsitans_GASH01000021
Non cytoplasmic domain
513. Smorsitans_VG_c6844_g1_i1__CDS1
Non cytoplasmic domain
514. Cormocephalus_westwoodi_GASL01000005
Non cytoplasmic domain
515. Scolopendra_subspinipes_GGDW01000009.1
Non cytoplasmic domain
516. Aquimarina_sp._Aq135_WP_108869647.1
Non cytoplasmic domain
517. Smaritima_Male_c23080_g1_i1__CDS1
518. Scolopendra_subspinipes_GGDW01000014.1
Non cytoplasmic domain
519. Scutigera_coleoptrata_VG_c743033_g6_i3__CDS3
Non cytoplasmic domain
520. Scutigera_coleoptrata_VG_c743033_g6_i8__CDS2
Aerolisin/ETX pore-forming domain
Proaerolysin
521. Thereuopoda_longicornis_GASR01000018
Non cytoplasmic domain
Proaerolysin
Aerolisin/ET...
522. Thereuopoda_longicornis_GASR01000051
Aer...
Aerolisin/ET...
Proaerolysin
523. Thereuopoda_longicornis_GASR01000019
Non cytoplasmic domain
524. Scutigera_coleoptrata_VG_c742795_g1_i1
Non cytoplasmic domain
525. Thereuopoda_longicornis_GASR01000057
Non cytoplasmic domain
526. Thereuopoda_longicornis_GASR01000063
Non cytoplasmic domain
527. Thereuopoda_longicornis_GASR01000029
Non cytoplasmic domain
Aerolisin/ET...

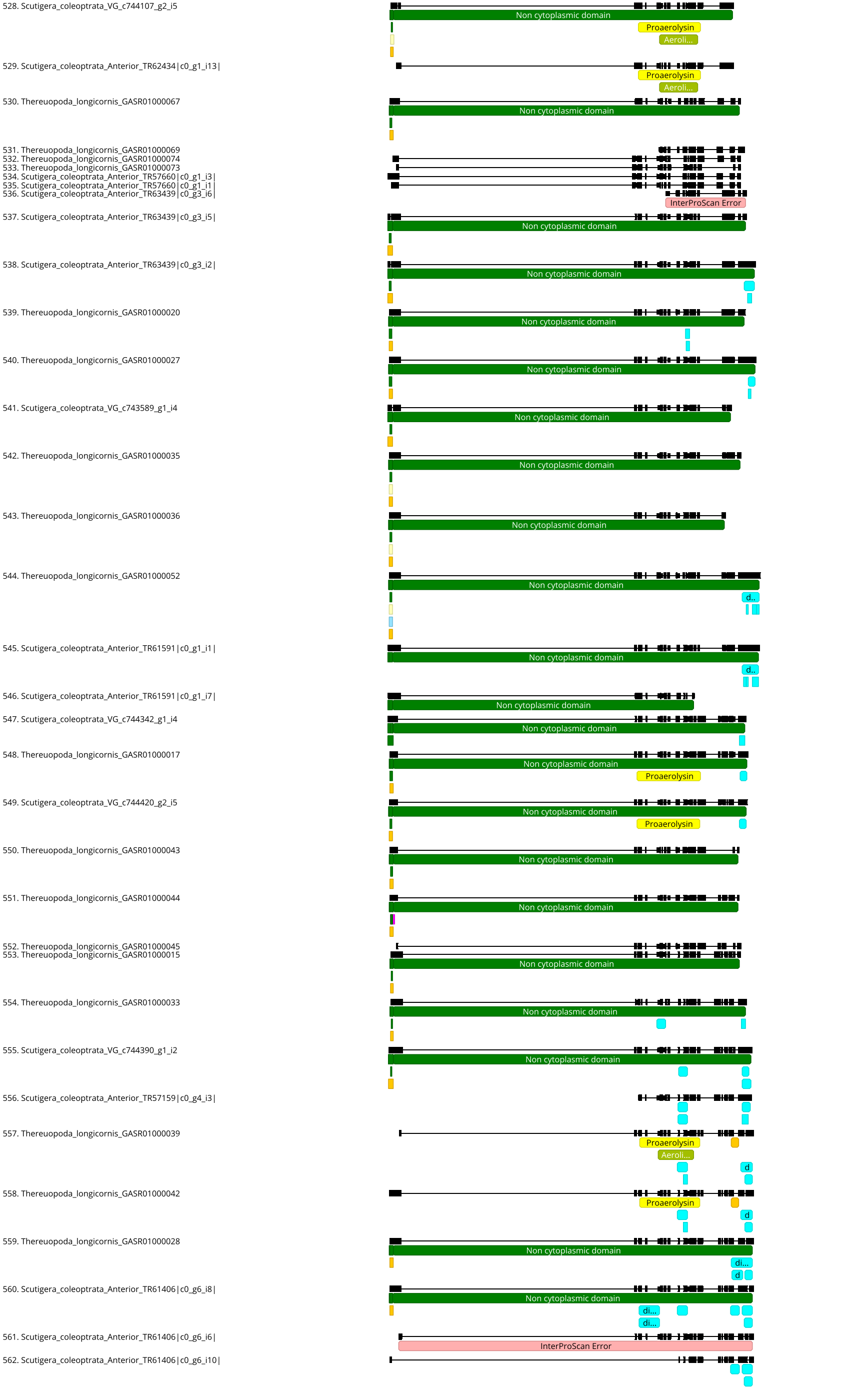
528. Scutigera_coleoptrata_VG_c744107_g2_i5
Non cytoplasmic domain
Proaerolysin
Aeroli...
529. Scutigera_coleoptrata_Anterior_TR62434|c0_g1_i13|
Proaerolysin
Aeroli...
530. Thereuopoda_longicornis_GASR01000067
Non cytoplasmic domain
531. Thereuopoda_longicornis_GASR01000069
532. Thereuopoda_longicornis_GASR01000074
533. Thereuopoda_longicornis_GASR01000073
534. Scutigera_coleoptrata_Anterior_TR57660|c0_g1_i3|
535. Scutigera_coleoptrata_Anterior_TR57660|c0_g1_i1|
536. Scutigera_coleoptrata_Anterior_TR63439|c0_g3_i6|
InterProScan Error
537. Scutigera_coleoptrata_Anterior_TR63439|c0_g3_i5|
Non cytoplasmic domain
538. Scutigera_coleoptrata_Anterior_TR63439|c0_g3_i2|
Non cytoplasmic domain
539. Thereuopoda_longicornis_GASR01000020
Non cytoplasmic domain
540. Thereuopoda_longicornis_GASR01000027
Non cytoplasmic domain
541. Scutigera_coleoptrata_VG_c743589_g1_i4
Non cytoplasmic domain
542. Thereuopoda_longicornis_GASR01000035
Non cytoplasmic domain
543. Thereuopoda_longicornis_GASR01000036
Non cytoplasmic domain
544. Thereuopoda_longicornis_GASR01000052
Non cytoplasmic domain
d..
545. Scutigera_coleoptrata_Anterior_TR61591|c0_g1_i1|
Non cytoplasmic domain
d..
546. Scutigera_coleoptrata_Anterior_TR61591|c0_g1_i7|
Non cytoplasmic domain
547. Scutigera_coleoptrata_VG_c744342_g1_i4
Non cytoplasmic domain
548. Thereuopoda_longicornis_GASR01000017
Non cytoplasmic domain
Proaerolysin
549. Scutigera_coleoptrata_VG_c744420_g2_i5
Non cytoplasmic domain
Proaerolysin
550. Thereuopoda_longicornis_GASR01000043
Non cytoplasmic domain
551. Thereuopoda_longicornis_GASR01000044
Non cytoplasmic domain
552. Thereuopoda_longicornis_GASR01000045
553. Thereuopoda_longicornis_GASR01000015
Non cytoplasmic domain
554. Thereuopoda_longicornis_GASR01000033
Non cytoplasmic domain
555. Scutigera_coleoptrata_VG_c744390_g1_i2
Non cytoplasmic domain
556. Scutigera_coleoptrata_Anterior_TR57159|c0_g4_i3|
557. Thereuopoda_longicornis_GASR01000039
Proaerolysin
Aeroli...
d
558. Thereuopoda_longicornis_GASR01000042
Proaerolysin
d
559. Thereuopoda_longicornis_GASR01000028
Non cytoplasmic domain
di...
d
560. Scutigera_coleoptrata_Anterior_TR61406|c0_g6_i8|
Non cytoplasmic domain
di...
di...
561. Scutigera_coleoptrata_Anterior_TR61406|c0_g6_i6|
InterProScan Error
562. Scutigera_coleoptrata_Anterior_TR61406|c0_g6_i10|
