# Supplementary material for: Phylogenetic analyses suggest centipede venom arsenals were repeatedly stocked by horizontal gene transfer
Source: Nat Commun. 2021 Feb 5;12:818. doi: 10.1038/s41467-021-21093-8 (PMC7864903; doi:10.1038/s41467-021-21093-8)
Supplement: Supplementary file 2 — Peer Review File [file 41467_2021_21093_MOESM2_ESM.pdf]

Reviewer #1 (Remarks to the Author):

The manuscript by Undheim and Jenner describes the likely multiple horizontal gene transfers (HGTs) of toxin-encoding genes to centipedes from bacteria, oomycetes and fungi. Altogether the authors reveal that at the minimum, the number of transfer events is 10, with eight of them having clear directionality from the non-centipede donor to centipedes. Interestingly, some of the protein families were transferred multiple times into centipede venom. Importantly, the claims of the authors are not based only on transcriptomics and phylogeny, but also include proteomics data that clearly demonstrate the production of members of those protein families in the venom at the protein level. All in all, this is an important study that highlights an important biological phenomenon in venom evolution that was overlooked for a long time. However, I have one significant reservation: I am not 100% convinced that the novelty of this study is at the level that justifies publication in a high impact journal at the level of Nature Communications. My (slight) reservation stems from the fact that HGTs were shown before to contribute to venom evolution of other animals (mostly arthropods and cnidarians as the authors mention in the introduction), even if at a lesser magnitude. Still, the finding that HGTs play such a pivotal role in the venom evolution of centipede venom is definitely novel and interesting. This is of course up to the editor to decide what novelty level is required to meet the criteria of Nature Communications.
Specific comments:
1. Line 74: I guess this is a typo: please change "last" to "least".
2. Line 105: This is weird and not helpful at all to base such a claim on personal communication. Either provide the information, or remove this.
3. Lines 185-187: This is a weak argument and the authors have much better ones. The selection for polyA-tailed RNA is far from perfect and results in enrichment rather than full selectivity. If the authors really wish to go in this direction they should amplify several toxin-encoding transcripts with a specific primer and poly-dT primer (basically following a 3' RACE protocol).
4. Lines 249-250: see my previous point.
5. The authors are making an argument that for some centipede sequences with bacterial homology they cannot check the presence of introns because they have no homologs specifically in Strigamia maritima and this is the only species with a sequenced genome. I would argue that for proving their point the authors could run a PCR on genomic DNA from another species that does express those proteins in its venom and show the presence of introns. This experimental task is not too challenging if the authors have access to at least a handful of species and showing the presence of introns in a couple of cases should already be informative.
6. Lines 324: I'm not sure that using the terminology of a venom expressing a protein is the most accurate one. Please rephrase.
7. Lines 414-415: This statement is somewhat confusing While arthropods are involved in many of the cases there are also other groups such as cnidarians where multiple HGT events of toxin-encoding genes were shown (the authors actually refer to these studies in other parts of their manuscript). Please rephrase accordingly.

Reviewer #2 (Remarks to the Author):

The manuscript reported novel results that the centipede venom components were evolved by horizontal gene transfer (HGT). Authors provided evidences that the DNA sequences used in the research were not contaminated. However, the construction of the phylogenetic trees seems imperfect which weaken the analyses.

Major points:
1. What the definition of ß-PFTx? ß-PFTxs in the manuscript were classified as aerolysin-like proteins (ALPs）. The definition of ß-PFTx determine the scales of ALPs take into calculation. Are all ALPs included or only orthologous genes of ß-PFTx previously found in centipede venoms used? The methods and criteria of ALPs need detailed in the manuscript.

2. ALPs belong to a superfamily and some ALP subgroups were not satisfactory classified. Since the similarities of some ALP subgroup members are lower, if using all ALP sequence to construct phylogenetic trees might be reflect subgroup relationship other than evolution relationship.
3. “Initial identification of HGT candidates” might retrieve more sequences by HMMER search.
4. “Construction of phylogenetic datasets”, the used sequences to construct of HMM were from non-metazoan might be not suitable.

Reviewer #3 (Remarks to the Author):

This paper presents very thorough phylogenetic analyses to show evidence for multiple horizontal gene transfer events (HGT) contributing proteins to centipede venom. This is a cool, exciting result, showing the importance of non-standard evolutionary mechanisms providing the fuel for key evolutionary innovations. The paper is well written, easy to read and presents a number of interesting ideas and results. I thank the authors for the care they took in preparing a well written, enjoyable to read manuscript.

Here are my main thoughts on how the manuscript could be improved:

1) There is a lot of work behind the analyses based on many data sets. Ultimately the force of the arguments depends on the quality of the phylogenetic trees, so I wanted to look at the alignments the tree figures are based on. Fortunately the authors provide the alignments in the supplementary files. However, I find it hard to visualize the alignments because they are provided in sequential fasta format. It’s really hard to get a sense of the quality of the alignments without me feeding these files into another program and I don’t see readers being likely to do that, but they should be able to assess the alignments easily. Some look ok but others a little sketchy – many gaps, making me wonder about homology. I would like to see the authors provide a better way for the readers to visualize the alignments, without having to re-analyze the data themselves. Perhaps in addition to the fasta format, the authors should provide figurers of the alignments in interleaved format. Periodically in the paper, the authors mention the presence or absence of various domains in members of the protein families. The supplementary alignment figures could be used to highlight these domains.

2) The direction and number of times of HGTs to centipedes to or from bacteria or other organisms for each protein family is inferred throughout the paper based on what I assume is the most parsimonious reconstruction, but this is not explicitly explained in the methods section. How this is inferred is important to the story, and I think the authors could do more analytically in determining this. For example, why not reconstruct ancestral states at nodes for taxonomic host of the proteins using likelihood or other methods that might provide probabilities of state? And perhaps mark where on the tree HGTs are inferred to have occurred? Related to this I get concerned about mid-point rooting, as it is so important in determining the directionality of evolutionary change, although I don’t know what alternative the authors have. It might be worth the authors mentioning this issue in their discussion.

3) One thing I think important that is missing from this paper, especially given the prominence it may ultimately have, is a summary species phylogeny for the centipedes, showing where and when the HGT events for the various protein families occurred, maybe with a timeline. While overall interesting to read, I found myself sometimes lost in the discussion of various protein families and centipede lineages and I think such a figure (species phylogeny with inferred HGTs mapped on) would be very helpful to the reader.

4) The authors discuss particular centipede species having streamlined venom proteomes, including S. maritma, the only one with a genome. Perhaps the authors could explain what they mean by this, e.g., compare the number of types of proteins found in streamlined vs. non-streamlined?

5) On line 166 the authors mention the great diversity of beta-PFTx transcripts expressed in centipede venom proteomes and provide a reference to a paper. For those unfamiliar with those papers, providing a specific number of transcripts would be helpful.

6) On lines 197-228, the authors repeatedly mention protein domains in different proteins and their presence or absence and how that related to HGT. I got a little lost in keeping track of the details. This might be nicely summarized in a figure, or could be mapped to the alignments I mention above

7) On lines 434-437, the authors mention the four protein families that we reconstructed to be present in the ancestral centipede venom. Other than the beta-PFTx, the authors should clarify which ones they are referring to. This is where the species phylogeny summary of HGT events for these families would be helpful.

8) It would be interesting for the authors to briefly discuss the mechanisms of HGT leading to a venom protein. For example, it’s implied that the genes are transferred to the various animal genomes via their germ line cells. Then there is the problem of regulating expression of a toxin so it selectively expressed in one’s venom glands. I believe the evidence supports that these rare evolutionary events happened, but it is hard to wrap one’s brain around the improbability of this.

Response to reviewers' comments for manuscript NCOMMS-20-28823

Below we respond to all the reviewers' comments, with our answers in *italics*. In the main text of the manuscript we have highlighted in yellow all the changes made.

Reviewer #1 (Remarks to the Author):

The manuscript by Undheim and Jenner describes the likely multiple horizontal gene transfers (HGTs) of toxin-encoding genes to centipedes from bacteria, oomycetes and fungi. Altogether the authors reveal that at the minimum, the number of transfer events is 10, with eight of them having clear directionality from the non-centipede donor to centipedes. Interestingly, some of the protein families were transferred multiple times into centipede venom. Importantly, the claims of the authors are not based only on transcriptomics and phylogeny, but also include proteomics data that clearly demonstrate the production of members of those protein families in the venom at the protein level. All in all, this is an important study that highlights an important biological phenomenon in venom evolution that was overlooked for a long time. However, I have one significant reservation: I am not 100% convinced that the novelty of this study is at the level that justifies publication in a high impact journal at the level of Nature Communications. My (slight) reservation stems from the fact that HGTs were shown before to contribute to venom evolution of other animals (mostly arthropods and cnidarians as the authors mention in the introduction), even if at a lesser magnitude. Still, the finding that HGTs play such a pivotal role in the venom evolution of centipede venom is definitely novel and interesting. This is of course up to the editor to decide what novelty level is required to meet the criteria of Nature Communications.
Specific comments:

1. Line 74: I guess this is a typo: please change "last" to "least".

Changed.

2. Line 105: This is weird and not helpful at all to base such a claim on personal communication. Either provide the information, or remove this.

It was necessary to cite both the published paper and a personal communication from the first author, Dr Clementine Francois, because although the paper reports that only a single scaffold of the Strigamia maritima *genome was a candidate for possible contamination, it did not provide the name of this scaffold. Dr Francois provided us with the identify of this scaffold, which allowed us to check whether the candidate HGT genes mapped to it. As per the requirements of the journal, Dr Francois has provided a written declaration that she gives us permission to include this information in our manuscript.*

3. Lines 185-187: This is a weak argument and the authors have much better ones. The selection for polyA-tailed RNA is far from perfect and results in enrichment rather than full selectivity. If the authors really wish to go in this direction they should amplify several toxin-encoding transcripts with a specific primer and poly-dT primer (basically following a 3' RACE protocol).

We presented this information as ancillary information, but we agree with the reviewer that we have much better evidence for our conclusions. We have therefore removed these sentences, and modified the following sentence to underline that on current evidence

symbionts cannot be categorically rejected. For further discussion of additional labwork, see point 5 below.

4. Lines 249-250: see my previous point.

As for the comment above, we have removed these sentences.

5. The authors are making an argument that for some centipede sequences with bacterial homology they cannot check the presence of introns because they have no homologs specifically in Strigamia maritima and this is the only species with a sequenced genome. I would argue that for proving their point the authors could run a PCR on genomic DNA from another species that does express those proteins in its venom and show the presence of introns. This experimental task is not too challenging if the authors have access to at least a handful of species and showing the presence of introns in a couple of cases should already be informative.

As the reviewer realized, this experimental work could be done if we had access to suitable material from the relevant species. Unfortunately, we currently don't have access to fresh material for most species in which we detected these two genes (centiPAD and PCPDP-like protein). With the exception of Lithobius forficatus *these species were collected by ourselves and other authors in continental Europe, Australia, and New Zealand. With us being based in Norway and the UK, recollecting these species, especially under ongoing COVID restrictions, has been impossible. However, we recollected* L. forficatus, *which expresses both of these genes in its venom, in Norway to do the suggested experiments to try to demonstrate the presence of introns. In summary, we were able to confirm that: i) both genes are present in Norwegian populations of* L. forficatus *(a country we had not sampled before); ii) these genes are expressed in genomic DNA isolated from leg muscle, and are therefore not restricted to mRNA and proteins detected in the venom glands and forcipules. However, we only managed to amplify data from the 3' end of the coding sequences of both genes. Failure to amplify the 5' end of the coding sequences suggests that these regions could contain long introns, but it is of course not proof of this. For more detail, please see the full protocol below in square brackets.*

Further experiments are needed to generate conclusive evidence for the presence or absence of introns in these two genes. However, because of official advise to work from home at both our institutions, and severe restrictions on lab access, it has taken us this long to complete this work so far. Unfortunately, this situation will likely remain in place for the foreseeable future. We therefore want to make the following final point. Although the presence of introns in these genes would allow us to categorically reject that they are bacterial products, our combined phylogenetic, transcriptomic, and proteomic evidence virtually excludes the possility that we are dealing with bacterial contamination, as discussed in our manuscript. We are explicit in the text that symbionts cannot categorically be rejected as sources of these genes, but on the balance of available evidence we argue it is more likely that we are dealing with horizontally transferred genes. Importantly, the literature on HGT (e.g. Verster et al 2019, Mol. Biol. Evol. 36: 2105; Artamonova et al 2015, Environmental Microbiology *17: 2203;* Flot et al 2013, *Nature 500: 453*) *shows that by no means all horizontally transferred bacterial genes in animals have introns, especially not if the transfers were relatively recent events, which we explicitly argue for the centiPADs. Only future genome sequencing can shrink uncertainty to zero. In either case, HGT or symbionts as the source of centipede venom proteins are both significantly new discoveries.*

In view of these arguments, and given the current impossibility of us performing further labwork in a timely manner, we have decided to submit this revision with these caveats explicitly acknowledged. We don't wish to unduly delay publication of what we and the reviewers agree are interesting and important new insights.

[Protocol used: PCRs were performed using primers designed based on contigs identified in the venom. We used Primer-BLAST (https://www.ncbi.nlm.nih.gov/tools/primer-blast/) to design forward and reverse primers for amplifying overlapping regions of each gene that corresponded to no more than 1000 bp of the coding sequence. To minimise the chance of non-specific amplification, we also searched primer candidates against the full transcriptome of L. forficatus. *As our DNA template, we extracted DNA from leg muscle tissue from a single specimen collected near Oslo, Norway, using the Qiagen MagAttract HMW DNA Kit. PCR was carried out using the Qiagen UltraRun LongRange PCR Kit using standard protocols (with and without "Q solution") aimed for the amplification of >20 kb amplicons. This approach resulted in the amplification of two primer pairs for the centiPAD-encoding gene and one primer pair for the PCPDPLP-encoding gene. The amplicons included the last 631 bp of the CDS and 83 bp of the 3' UTR of the centiPAD transcript and 900 bp of 3' end of the CDS for the PCPDPLP-encoding transcript. However, each amplicon corresponded to the predicted length of each region without the addition of introns, while none of the three primer pairs designed for each gene to amplify the 5' end of the CDS (including the signal peptide and for centiPAD 5' UTR) yielded any PCR product.]*

6. Lines 324: I'm not sure that using the terminology of a venom expressing a protein is the most accurate one. Please rephrase.

Rephrased.

7. Lines 414-415: This statement is somewhat confusing While arthropods are involved in many of the cases there are also other groups such as cnidarians where multiple HGT events of toxin-encoding genes were shown (the authors actually refer to these studies in other parts of their manuscript). Please rephrase accordingly.

The statement is correct as it is. The reviewer is right that multiple HGT events are known for pore-forming toxins into cnidarians, but importantly, these do not represent HGT events into their venoms. The hydralysins and aerolysins in question are expressed exclusively outside the animals' venom systems, in ecto- and endodermal cells of the pharynx and gastrovascular system, as detailed in the cited literature.

Reviewer #2 (Remarks to the Author):

The manuscript reported novel results that the centipede venom components were evolved by horizontal gene transfer (HGT). Authors provided evidences that the DNA sequences used in the research were not contaminated. However, the construction of the phylogenetic trees seems imperfect which weaken the analyses.

The reviewer's concerns seem to arise from not fully grasping the strategy that we adopted in constructing our datasets. We trust that our answers below remove any uncertainty about our approach.

Major points:
1. What the definition of ß-PFTx? ß-PFTxs in the manuscript were classified as aerolysin-like proteins (ALPs）. The definition of ß-PFTx determine the scales of ALPs take into calculation. Are all ALPs included or only orthologous genes of ß-PFTx previously found in centipede venoms used? The methods and criteria of ALPs need detailed in the manuscript.

The reviewer seems to suggest that our dataset was constructed under the constraint that we only included aerolysin-like sequences that are classified as ß-PFTx, while other aerolysin-like sequences may have been excluded. This is not the case. In the text we note that the centipede ß-PFTx sequences belong to the aerolysin-like toxin superfamily because they contain an aerolysin domain (IPR005830), which is characteristic of this superfamily. However, it is the origin of centipede ß-PFTx's—not ALPs—that are of interest to our study. Therefore, as stated in the Methods section, we compiled our phylogenetic dataset for ß-PFTx by conducting HMMER searches against NCBI's nr database as well as our custom transcriptome and genome datasets with a HMMER profile that was constructed from an alignment that included all full-length centipede sequences, as well as selected metazoan outgroup taxa. Our search for putatively homologous sequences was therefore not constrained in any way to only ß-PFTx sequences, or sequences classified as aerolysin-like. We deliberately constructed this and our other phylogenetic datasets in a bottom-up approach that ensured that no relevant sequences were excluded, and without narrowing the selection of sequences by any top-down constraints based on a priori sequence annotations or sequence classifications.

2. ALPs belong to a superfamily and some ALP subgroups were not satisfactory classified. Since the similarities of some ALP subgroup members are lower, if using all ALP sequence to construct phylogenetic trees might be reflect subgroup relationship other than evolution relationship.

As we explain for the previous point, our dataset was constructed without any top-down constraints based on either the classification or annotation of putatively homologous sequences. Our bottom-up approach ensures that no potentially relevant sequences are excluded a priori.

3. "Initial identification of HGT candidates" might retrieve more sequences by HMMER search.

This is precisely what we did, as we describe in the following paragraph of the Methods section titled "Construction of phylogenetic datasets."

4. "Construction of phylogenetic datasets", the used sequences to construct of HMM were from non- metazoan might be not suitable.

We are unsure what the reviewer means here because of a lack of detail. However, only in the case of one of the genes we analysed, PCPDP-like protein, did we include non-metazoans in our HMMER profile to ensure a more powerful search for putatively homologous sequences, which is precisely the reviewer's concern in the previous points. We did this because the gene is only found in a single centipede species.

Reviewer #3 (Remarks to the Author):

This paper presents very thorough phylogenetic analyses to show evidence for multiple horizontal gene transfer events (HGT) contributing proteins to centipede venom. This is a cool, exciting result, showing the importance of non-standard evolutionary mechanisms providing the fuel for key evolutionary innovations. The paper is well written, easy to read and presents a number of interesting ideas and results. I thank the authors for the care they took in preparing a well written, enjoyable to read manuscript.

Here are my main thoughts on how the manuscript could be improved:

1) There is a lot of work behind the analyses based on many data sets. Ultimately the force of the arguments depends on the quality of the phylogenetic trees, so I wanted to look at the alignments the tree figures are based on. Fortunately the authors provide the alignments in the supplementary files. However, I find it hard to visualize the alignments because they are provided in sequential fasta format. It's really hard to get a sense of the quality of the alignments without me feeding these files into another program and I don't see readers being likely to do that, but they should be able to assess the alignments easily. Some look ok but others a little sketchy – many gaps, making me wonder about homology. I would like to see the authors provide a better way for the readers to visualize the alignments, without having to re-analyze the data themselves. Perhaps in addition to the fasta format, the authors should provide figurers of the alignments in interleaved format.

We acknowledge that this was a suboptimal way to provide alignments, which we adopted to reduce the number of supplementary files. To facilitate easier viewing we have now separated each alignment into its own fasta file (in Supplementary Data 4).

Regarding the reviewer's opinion that some of the alignments look 'a little sketchy' because of alignment gaps, we note that gaps do not provide useful information about sequence homology. After all, it is because indel events are so ubiquitous in homologous sequences that sequence alignment is a necessary step in molecular phylogenetic analyses in the first place. Since our datasets include such distantly related taxa the presence of gaps is expected. Moreover, an indel in just one sequence will create a gap throughout an entire alignment. This is illustrated in the alignment of unchar05, where the larger gaps are the result of an indel in single sequences that are aligned well in other regions.

Because the reviewer does not refer to any specific alignment, we offer a general view: We have robustly established the homology of the centipede sequences to those of non-metazoan donor taxa by the following steps: 1) employing the rigorous, statistical similarity thresholds for the detection of putatively homologous sequences used by BLAST and HMMER; 2) followed by performing phylogenetic analyses that robustly nest candidate centipede HGT sequences in paraphyletic backbones of non-metazoan taxa; 3) and additionally showing the shared presence of conserved protein domains or cysteine patterns shared between centipede and donor sequences, which add further support to our conclusions even where sequence divergence has been pronounced. We have now added a new supplementary file (Supplementary Data 8) with protein domain annotations mapped to all our alignments to facilitate visualisation of this (see next point).

Periodically in the paper, the authors mention the presence or absence of various domains in members of the protein families. The supplementary alignment figures could be used to highlight these domains.

We have added a new supplementary file to show the domain annotations for all alignments. We inserted this sentence into the Methods section: "We used InterProScan[70] as implemented in Geneious v11.1.5 (https://www.geneious.com) to generate protein domain annotations for all alignments (see Supplementary Data 8)."

2) The direction and number of times of HGTs to centipedes to or from bacteria or other organisms for each protein family is inferred throughout the paper based on what I assume is the most parsimonious reconstruction, but this is not explicitly explained in the methods section. How this is inferred is important to the story, and I think the authors could do more analytically in determining this. For example, why not reconstruct ancestral states at nodes for taxonomic host of the proteins using likelihood or other methods that might provide probabilities of state? And perhaps mark where on the tree HGTs are inferred to have occurred?

We appreciate the point the reviewer is trying to make here, but our analyses follow established protocol for determining where and when HGT may have occurred. We used model-based maximum likelihood methods to discover the relationships between host and putative donor taxa for each of the gene families. The results indicate where and how many times HGT may have taken place for each family by showing where donor sequences are placed in the tree. This does not involve a separate ancestral state reconstruction step, either with parsimony or model-based methods. Importantly, it is nonsensical to perform model-based maximum likelihood or Bayesian ancestral state reconstructions on such data. Model-based approaches assign probabilities to reconstructed ancestral states by taking into account the topology and branch lengths of the tree because these are relevant parameters for understanding the evolution of characters that evolved along the branches of the tree. However, such parameters for the host tree are completely uninformative for reconstructing the horizontal transfer of donor genes because these did not evolve along the host tree, but were received from an unrelated clade of donor sequences. Hence, while the topology and branch lengths of the host tree can be informative for reconstructing the vertical evolution of host genes along that tree, they are not informative for reconstructing where and when donor sequences were horizontally transferred into the host tree. Instead, we reconstructed where and when HGT events occurred with broadly sampled phylogenetic analyses of host and putative donor taxa, which is best practice for this kind of analysis. We performed tree topology tests to ensure we did not inflate the number of independent HGTs due to topological uncertainty.

Related to this I get concerned about mid-point rooting, as it is so important in determining the directionality of evolutionary change, although I don't know what alternative the authors have. It might be worth the authors mentioning this issue in their discussion.

We used midpoint rooting because it was impossible to designate outgroups to justify taxonomic rooting. We therefore inserted this sentence into the relevant part of the Methods section: "Because taxonomic outgroups could not be designated we used midpoint rooting to root the trees." *We also note that rooting the trees with centipedes (denying HGT into centipedes) would result in profoundly unlikely scenarios of often multiple HGT events from centipedes into large numbers of other eukaryotes and prokaryotes.*

3) One thing I think important that is missing from this paper, especially given the prominence it may ultimately have, is a summary species phylogeny for the centipedes, showing where and when the HGT events for the various protein families occurred, maybe with a timeline. While overall interesting to read, I found myself sometimes lost in the discussion of various protein families and centipede lineages and I think such a figure (species phylogeny with inferred HGTs mapped on) would be very helpful to the reader.

We agree with the reviewer, and have prepared a new summary figure (Figure 6) that shows where in the phylogeny of centipedes the different HGTs have occurred.

4) The authors discuss particular centipede species having streamlined venom proteomes, including S. maritma, the only one with a genome. Perhaps the authors could explain what they mean by this, e.g., compare the number of types of proteins found in streamlined vs. non-streamlined?

Since the relevant sentence (lines 401-403) does not contribute essential information to the point we are making here, we have removed it.

5) On line 166 the authors mention the great diversity of beta-PFTx transcripts expressed in centipede venom proteomes and provide a reference to a paper. For those unfamiliar with those papers, providing a specific number of transcripts would be helpful.

To keep the text concise we prefer to keep this as it is. The relevant sentence references the number of transcripts confirmed in venom proteomes, which differs between species and ranges from less than half a dozen to several dozen transcipts, as well as the relative abundance in venom as revealed by 2D-PAGE gels, and which again differs between species.

6) On lines 197-228, the authors repeatedly mention protein domains in different proteins and their presence or absence and how that related to HGT. I got a little lost in keeping track of the details. This might be nicely summarized in a figure, or could be mapped to the alignments I mention above

We have rephrased and refocused the text about the relevant clade to make it easier to understand. Also, as noted above under point 1, to make this clearer we now provide Supplementary Data 8 with all the protein domain annotations mapped to our alignments.

7) On lines 434-437, the authors mention the four protein families that we reconstructed to be present in the ancestral centipede venom. Other than the beta-PFTx, the authors should clarify which ones they are referring to. This is where the species phylogeny summary of HGT events for these families would be helpful.

We added the names of the three other protein families to the relevant sentence.

8) It would be interesting for the authors to briefly discuss the mechanisms of HGT leading to a venom protein. For example, it’s implied that the genes are transferred to the various animal genomes via their germ line cells. Then there is the problem of regulating expression of a toxin so it selectively expressed in one’s venom glands. I believe the evidence supports that these rare evolutionary events happened, but it is hard to wrap one’s brain around the improbability of this.

Although we agree that the mechanisms of HGT are fascinating and remain elusive in many ways, our paper did not study these, and there is unfortunately no space within the word limits of our paper to digress on this topic.

Reviewer #1 (Remarks to the Author):

I believe this is an interesting work that significantly contributes to our understanding of venom evolution. It is very clear that the authors have considerably revised the manuscript. It is regrettable that the PCR amplification of the intron-containing fragments failed, but I fully understand that under the current conditions of COVID-19 lab work is severely limited.
My only remaining remark is regarding aerolysin-like molecules being part of the cnidarian venom. In my opinion, the venom system of Cnidaria is so diffuse that gland cells even in the internal part of the animal can be considered part of the venom as noticeably cnidarians have nematocysts that puncture the prey even after it is internalized (see for example Schlesinger et al 2009 Proceedings of the Royal Society B 276: 1063-1067). Thus, the gland cell-derived toxins may still be part of the venom. Please rephrase accordingly. Other than that I do not have any remaining comments.

Reviewer #2 (Remarks to the Author):

Authors well organized the manuscript according to the points rised by reviewers. However, the novelty of the manuscript to be published in high-level journal of NC need be properly evaluated.

Response to reviewers

Reviewer #1 (Remarks to the Author):

I believe this is an interesting work that significantly contributes to our understanding of venom evolution. It is very clear that the authors have considerably revised the manuscript. It is regrettable that the PCR amplification of the intron-containing fragments failed, but I fully understand that under the current conditions of COVID-19 lab work is severely limited.
My only remaining remark is regarding aerolysin-like molecules being part of the cnidarian venom. In my opinion, the venom system of Cnidaria is so diffuse that gland cells even in the internal part of the animal can be considered part of the venom as noticeably cnidarians have nematocysts that puncture the prey even after it is internalized (see for example Schlesinger et al 2009 Proceedings of the Royal Society B 276: 1063-1067). Thus, the gland cell-derived toxins may still be part of the venom. Please rephrase accordingly. Other than that I do not have any remaining comments.

We have rephrased the relevant sentence in the second paragraph of the Introduction to reflect this, while noting that cnidarian venom workers themselves continue to debate where to draw the line between the venom and digestive systems.

Reviewer #2 (Remarks to the Author):

Authors well organized the manuscript according to the points rised by reviewers. However, the novelty of the manuscript to be published in high-level journal of NC need be properly evaluated.
